# State of the Art and Prospects for Halide Perovskite Nanocrystals

**DOI:** 10.1021/acsnano.0c08903

**Published:** 2021-06-17

**Authors:** Amrita Dey, Junzhi Ye, Apurba De, Elke Debroye, Seung Kyun Ha, Eva Bladt, Anuraj S. Kshirsagar, Ziyu Wang, Jun Yin, Yue Wang, Li Na Quan, Fei Yan, Mengyu Gao, Xiaoming Li, Javad Shamsi, Tushar Debnath, Muhan Cao, Manuel A. Scheel, Sudhir Kumar, Julian A. Steele, Marina Gerhard, Lata Chouhan, Ke Xu, Xian-gang Wu, Yanxiu Li, Yangning Zhang, Anirban Dutta, Chuang Han, Ilka Vincon, Andrey L. Rogach, Angshuman Nag, Anunay Samanta, Brian A. Korgel, Chih-Jen Shih, Daniel R. Gamelin, Dong Hee Son, Haibo Zeng, Haizheng Zhong, Handong Sun, Hilmi Volkan Demir, Ivan G. Scheblykin, Iván Mora-Seró, Jacek K. Stolarczyk, Jin Z. Zhang, Jochen Feldmann, Johan Hofkens, Joseph M. Luther, Julia Pérez-Prieto, Liang Li, Liberato Manna, Maryna I. Bodnarchuk, Maksym V. Kovalenko, Maarten B. J. Roeffaers, Narayan Pradhan, Omar F. Mohammed, Osman M. Bakr, Peidong Yang, Peter Müller-Buschbaum, Prashant V. Kamat, Qiaoliang Bao, Qiao Zhang, Roman Krahne, Raquel E. Galian, Samuel D. Stranks, Sara Bals, Vasudevanpillai Biju, William A. Tisdale, Yong Yan, Robert L. Z. Hoye, Lakshminarayana Polavarapu

**Affiliations:** 1 Chair for Photonics and Optoelectronics, Nano-Institute Munich, Department of Physics, Ludwig-Maximilians-Universität (LMU), Königinstrasse 10, 80539 Munich, Germany; 2 Cavendish Laboratory, University of Cambridge, 19 JJ Thomson Avenue, Cambridge CB3 0HE, United Kingdom; 3 School of Chemistry, University of Hyderabad, Hyderabad 500 046, India; 4 Department of Chemistry, KU Leuven, 3001 Leuven, Belgium; 5 Department of Chemical Engineering, Massachusetts Institute of Technology, Cambridge, Massachusetts 02139, United States; 6 EMAT, University of Antwerp, Groenenborgerlaan 171, 2020 Antwerp, Belgium; 7 NANOlab Center of Excellence, University of Antwerp, 2020 Antwerp, Belgium; 8 Department of Chemistry, Indian Institute of Science Education and Research (IISER), Pune 411008, India; 9 School of Science and Technology for Optoelectronic Information ,Yantai University, Yantai, Shandong Province 264005, China; 10 Division of Physical Science and Engineering, King Abdullah University of Science and Technology, Thuwal 23955-6900, Kingdom of Saudi Arabia; 11 MIIT Key Laboratory of Advanced Display Materials and Devices, Institute of Optoelectronics & Nanomaterials, College of Materials Science and Engineering, Nanjing University of Science and Technology, Nanjing 210094, China; 12 Division of Physics and Applied Physics, School of Physical and Mathematical Sciences, Nanyang Technological University, Singapore 637371; 13 Department of Chemistry, University of California, Berkeley, Berkeley, California 94720, United States; 14 Materials Sciences Division, Lawrence Berkeley National Laboratory, Berkeley, California 94720, United States; 15 LUMINOUS! Center of Excellence for Semiconductor Lighting and Displays, TPI-The Photonics Institute, School of Electrical and Electronic Engineering, Nanyang Technological University, Singapore 639798; 16 Department of Materials Science and Engineering, University of California, Berkeley, California 94720, United States; 17 Institute of Functional Nano & Soft Materials (FUNSOM), Jiangsu Key Laboratory for Carbon-Based Functional Materials and Devices, Soochow University, Suzhou 215123, China; 18 Lehrstuhl für Funktionelle Materialien, Physik Department, Technische Universität München, James-Franck-Str. 1, 85748 Garching, Germany; 19 Heinz Maier-Leibnitz Zentrum (MLZ), Technische Universität München, Lichtenbergstr. 1, D-85748 Garching, Germany; 20 MACS Department of Microbial and Molecular Systems, KU Leuven, 3001 Leuven, Belgium; 21 Chemical Physics and NanoLund Lund University, PO Box 124, 22100 Lund, Sweden; 22 Graduate School of Environmental Science and Research Institute for Electronic Science, Hokkaido University, Sapporo, Hokkaido 001-0020, Japan; 23 Department of Chemistry and Biochemistry, University of California, Santa Cruz, California 95064, United States; 24 Beijing Key Laboratory of Nanophotonics and Ultrafine Optoelectronic Systems, School of Materials Science & Engineering, Beijing Institute of Technology, 5 Zhongguancun South Street, Haidian District, Beijing 100081, China; 25 Department of Materials Science and Engineering, and Centre for Functional Photonics (CFP), City University of Hong Kong, 83 Tat Chee Avenue, Kowloon, Hong Kong S.A.R.; 26 McKetta Department of Chemical Engineering and Texas Materials Institute, The University of Texas at Austin, Austin, Texas 78712-1062, United States; 27 School of Materials Sciences, Indian Association for the Cultivation of Science, Kolkata 700032, India; 28 Department of Chemistry and Biochemistry, San Diego State University, San Diego, California 92182, United States; 29 Department of Chemistry, University of Washington, Seattle, Washington 98195, United States; 30 Department of Chemistry, Texas A&M University, College Station, Texas 77843, United States; 31 Centre for Disruptive Photonic Technologies (CDPT), Nanyang Technological University, Singapore 637371; 32 Division of Physics and Applied Physics, School of Physical and Mathematical Sciences, Nanyang Technological University, Singapore 639798; 33 Department of Electrical and Electronics Engineering, Department of Physics, UNAM-Institute of Materials Science and Nanotechnology, Bilkent University, Ankara 06800, Turkey; 34 Institute of Advanced Materials (INAM), Universitat Jaume I, 12071 Castelló, Spain; 35 Max Planck Institute for Polymer Research, Mainz 55128, Germany; 36 National Renewable Energy Laboratory, Golden, Colorado 80401, United States; 37 Institute of Molecular Science, University of Valencia, c/Catedrático José Beltrán 2, Paterna, Valencia 46980, Spain; 38 School of Environmental Science and Engineering, Shanghai Jiao Tong University, Shanghai 200240, China; 39 Nanochemistry Department, Istituto Italiano di Tecnologia, Via Morego 30, Genova 16163, Italy; 40 Institute of Inorganic Chemistry and § Institute of Chemical and Bioengineering, Department of Chemistry and Applied Bioscience, ETH Zurich, Vladimir Prelog Weg 1, CH-8093 Zürich, Switzerland; 41 Laboratory for Thin Films and Photovoltaics, Empa−Swiss Federal Laboratories for Materials Science and Technology, Überlandstrasse 129, CH-8600 Dübendorf, Switzerland; 42 Kavli Energy NanoScience Institute, Berkeley, California 94720, United States; 43 Institute for Chemical and Bioengineering, Department of Chemistry and Applied Biosciences, ETH-Zurich, CH-8093 Zürich, Switzerland; 44 Notre Dame Radiation Laboratory, Department of Chemistry and Biochemistry, University of Notre Dame, Notre Dame, Indiana 46556, United States; 45 Department of Materials Science and Engineering and ARC Centre of Excellence in Future Low-Energy Electronics Technologies (FLEET), Monash University, Clayton, Victoria 3800, Australia; 46 Istituto Italiano di Tecnologia, Via Morego 30, 16163 Genova, Italy; 47 Department of Chemical Engineering and Biotechnology, University of Cambridge, Cambridge CB3 0AS, United Kingdom; 48 Department of Materials, Imperial College London, Exhibition Road, London SW7 2AZ, United Kingdom; 49 CINBIO, Universidade de Vigo, Materials Chemistry and Physics group, Departamento de Química Física, Campus Universitario As Lagoas, Marcosende, 36310 Vigo, Spain; 50 Advanced Membranes and Porous Materials Center, King Abdullah University of Science and Technology, Thuwal 23955-6900, Kingdom of Saudi Arabia; 51 KAUST Catalysis Center, King Abdullah University of Science and Technology, Thuwal 23955-6900, Kingdom of Saudi Arabia; 52 Multiscale Crystal Materials Research Center, Shenzhen Institute of Advanced Technology, Chinese Academy of Sciences, Shenzhen 518055, China

**Keywords:** metal-halide perovskite nanocrystals, perovskite nanoplatelets, perovskite nanocubes, perovskite nanowires, lead-free perovskite nanocrystals, light-emitting devices, photovoltaics, lasers, photocatalysts, photodetectors

## Abstract

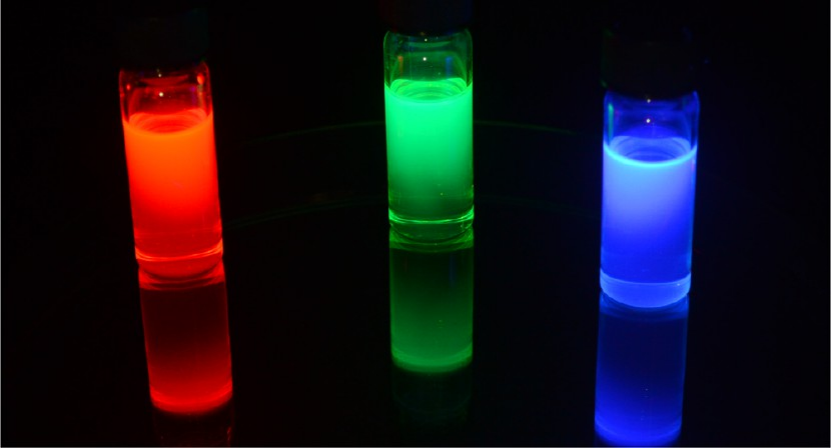

Metal-halide perovskites have rapidly emerged as one of the most promising materials of the 21st century, with many exciting properties and great potential for a broad range of applications, from photovoltaics to optoelectronics and photocatalysis. The ease with which metal-halide perovskites can be synthesized in the form of brightly luminescent colloidal nanocrystals, as well as their tunable and intriguing optical and electronic properties, has attracted researchers from different disciplines of science and technology. In the last few years, there has been a significant progress in the shape-controlled synthesis of perovskite nanocrystals and understanding of their properties and applications. In this comprehensive review, researchers having expertise in different fields (chemistry, physics, and device engineering) of metal-halide perovskite nanocrystals have joined together to provide a state of the art overview and future prospects of metal-halide perovskite nanocrystal research.

The earliest research work on metal-halide perovskites (MHPs) was conducted in the late 1800s by Wells,^1^ while the detailed structural characterization was carried out by Weber in the 1900s.^2−4^ Their potential applications in electronic and optical devices attracted attention in the late 1990s and the early 2000s, long before captivating the broad scientific community.^5,6^ In 2009, Kojima *et al*.^7^ demonstrated the use of lead-halide perovskites (LHPs) as visible-light sensitizers in solar cells, but it took another 3 years to fully grasp their potential for highly efficient photovoltaics.^8,9^ Since then, the number of researchers working on MHPs has been increasing significantly over the years, accompanied by a substantial increase in research output in this area. The high efficiency of LHP photovoltaic cells is attributed to long charge carrier diffusion lengths along with low Urbach energies, high photoluminescence quantum yields, and high absorption coefficients.^10,11^ These significant features are of interest not only for the device communities but also for the chemistry, physics, and materials research communities. Over the last decade, numerous advances have been made toward the fundamental understanding as well as potential applications of MHPs. The certified power conversion efficiency (PCE) of single-junction perovskite-based solar cells has surpassed 25% in a short span of time, demonstrating an order of magnitude higher rate of improvement compared to other photovoltaic technologies.^12^ MHPs have recently emerged at the forefront of materials research not only because of their impressive photovoltaic performance but also due to their attractive optical and electronic properties.^10,11,13−30^ Over the years, they have already shown great promise in a wide range of technological applications encompassing photovoltaics (PVs), light-emitting diodes (LEDs), lasers, transistors, photodetectors, and photocatalysts.^27,31−45^ The optical and electronic properties of MHPs were shown to be strongly dependent on their dimensionality (both structural and morphological).^6,14,16,18,22,30,46−50^

Three-dimensional (3D) MHPs refer to a class of crystalline compounds adopting the generic chemical formula ABX_3_, where the cation “B” has six nearest-neighbor anions “X”, while the cation “A” sits in a cavity formed by eight corner-sharing BX_6_ octahedra.^10,51,52^ MHPs are generally classified into either organic–inorganic hybrid (OIH) or inorganic perovskites depending on whether the A-site cation is organic or inorganic. OIH perovskites generally have methylammonium (MA) or formamidinium (FA) as the monovalent A-site cation, lead, tin, or germanium as the divalent B cation and chlorine, bromine, iodine, or their combinations as the halide ion (X). On the other hand, inorganic perovskites have cesium (Cs) or rubidium (Rb) as the A cation. The ideal structure of the perovskite, which is illustrated in Figure 1A, is based on a cubic lattice. However, the deviation from the ideal perovskite structure in ABX_3_ materials can be predicted through the Goldschmidt tolerance factor *t* (*t* = (*r*_A_ + *r*_X_)/[√2(*r*_B_ + *r*_X_)]), where *r*_A_, *r*_B_, and *r*_X_ are the ionic radii of the corresponding ions, and *t* is defined as the ratio of the distance A–X to the distance B–X. Unlike classical semiconductors (such as Ge, Si, GaAs, CdS, CdSe, InP), high-quality MHPs can be prepared by simply mixing the corresponding precursor solutions at room temperature (RT) under ambient conditions due to their inherent ionic character.^29,53,54^ The optical properties of MHPs are easily tunable across the visible spectrum of light by simply varying the halide composition.^30,55−57^ While the bulk properties of MHP are significant, decreasing the size of the crystals to the nanoscale reveals their size-dependent optical and electronic properties. For instance, nanosized crystals (nanocrystals, NCs) of MHP exhibit quantum-confinement effects that can be exploited to tune the optical properties,^14,16,19,22^ much like in other semiconductors.^58,59^ The structural dimensionality of MHPs is easily tunable from 3D to 2D using long-chain alkylammonium cations in their synthesis (Figure 1B). The emission wavelength and exciton binding energies of these layered perovskites are controllable by the number of octahedral layers between the long-chain organic layers (*n* = 1 to ∞).^49,60,61^ The tunable emission wavelength, narrow emission, and low nonradiative losses of MHPs make them potential candidates for LEDs. In addition, the long charge carrier diffusion lengths in MHPs facilitate efficient recombination of electrically injected charge carriers. Bulk perovskites suffer from low photoluminescence quantum yields (PLQYs) due to inherent defects, particularly those present at grain boundaries, surfaces, and interfaces.^15,62,63^ On the other hand, MHP NCs appeared as extremely efficient light emitters with near-unity PLQY. The early reports on colloidal halide perovskites emerged in 2012–2014.^64−66^ Despite limited control over the size, shape, and colloidal stability, those early papers showed that such fine perovskite particles exhibit much enhanced emissivity, as evidenced by a PLQY of ∼20% for MAPbBr_3_ colloids.^66^ In late 2014, Gonzalez-Carrero *et al*.^25^ reported an improved synthesis of highly luminescent MAPbBr_3_ colloids in toluene. Although the particles were found to be polydisperse and irregularly shaped, as seen from the transmission electron microscopy (TEM) images, they exhibited an impressive PLQY of 80% and stood in drastic contrast to classical colloidal quantum dots (QDs), such as those made of CdSe and InP, which must be epitaxially overcoated with wider-band-gap inorganic shells, such as CdS or ZnS, for imparting high PLQY values.^67^ The most relevant colloidal synthesis of well-defined colloidal LHP NCs, which enabled exquisite control over the size and size distribution and thermodynamic stability of colloids, was the one by Protesescu *et al.* in January 2015 using the hot-injection (HI) method, which delivered monodisperse CsPbX_3_ NCs.^14^ These CsPbX_3_ NCs not only exhibited PLQY values up to 100% but also showed quantum-size effects similar to classical QDs. In March 2015, Zhang *et al*. introduced the ligand-assisted reprecipitation (LARP) approach for the room-temperature synthesis of MAPbX_3_ NCs with color-tunable emission and PLQY up 70%.^29^ In the same year, Tyagi *et al*.^19^ and Sichert *et al.*(16) simultaneously reported the preparation of MAPbBr_3_ perovskite nanoplatelets (NPls). The precise control of the number of monolayers in the platelets, down to monolayer, demonstrated in the latter report and achieved by changing the ratio of the organic cations in LARP, enabled a careful assessment of the quantum-confinement effects in the platelets.^16^ Later, the synthesis methodology initially proposed for CsPbX_3_ NCs in reference (14) was used also in the early reports on FAPbX_3_ (X = Br, I) and CsFAPbI_3_ NCs.^68,69^ After these seminal reports on uniform perovskite NCs, there has been a surge in MHP NC research. Over the years, numerous efforts have been devoted to control the size and shape of MHP NCs by varying the ligands, reaction temperatures, and precursors. A wide range of morphologies such as nanocubes, nanowires (NWs), nanorods (NRs), NPls, nanosheets (NSs), multifaced nanocrystals,^70−72^ and QDs (nanocubes with sized in the strong quantum-confinement regime) have been reported.^18,23,36,48,52,56,73−76^ These NCs exhibit either bulk-like (3D) or quantum-confined (2D or 0D) properties depending on their dimensions. For instance, the thickness of the NPls is precisely tunable down to a single layer of edge-sharing octahedra (Figure 1B, strongly quantum-confined region). Over the years, the syntheses of LHP NCs have been optimized toward monodispersity, with near-unity PLQY and colloidal stability.^52,77,78^ Their size/shape and composition (A, B, and X) are also tunable by post-synthetic shape transformations and ion exchange, respectively.^52,55,57,73,79^ Furthermore, their optical properties are tunable by self-assembly into superlattices.^80−83^ Although low-band-gap, iodine-based MHPs are also defect-tolerant, surface defects caused by the detachment of ligands and surface atoms (B and X) can strongly affect their PLQYs.^60,84^ To overcome these effects, post-synthetic surface treatment methods have been developed.^52,60,85,86^ In general, a post-synthetic treatment of LHP NCs with ligand molecules or metal halides leads to a significant improvement in their PLQY.^60,84,87,88^ Additional properties could be achieved in perovskite NCs by post-synthetic treatments with functional molecules. The controlled synthesis of LHP NCs makes it easy for the researchers to test these fascinating NCs as active materials in a wide range of applications, including LEDs,^40^ lasers,^89^ solar cells,^90,91^ photodetectors,^37^ transistors^92,93^ and for photocatalysis.^43^ On the other hand, despite the rapid progress in various aspects of LHP NCs, their stability is one of the major roadblocks in advancing the field toward real-world applications. To address this issue, researchers have implemented both *in situ* synthesis as well as post-synthetic surface coating strategies,^41,94^ but by these approaches, the perovskite NCs are often protected with a layer of organic ligands, acting as a dielectric surface coating, which is a major concern for the injection and transport of charge carriers. Therefore, perovskite NCs coated with dielectric shells can only be used as down-converters in LEDs. Another major obstacle for applying LHP NCs in consumer products such as LEDs and solar cells is the toxicity of lead. Therefore, researchers have been testing various other metals to replace this lead with less toxic alternatives. The replacement of divalent Pb^2+^ with trivalent Bi^3+^ or Sb^3+^ leads to the formation of vacancy ordered triple perovskites (A_3_B_2_X_9_), which have a 0D or 2D structure, with exciton binding energies higher than those of the 3D perovskites.^95−97^ On the other hand, the perovskite crystal structure can be preserved by adding a monovalent B-site cation, as well (*e.g*., Ag), which leads to the formation of double perovskites, as illustrated in Figure 1C, which have been facing their own challenges in terms of wide band gaps and low PLQYs thus far.

**Figure 1:**
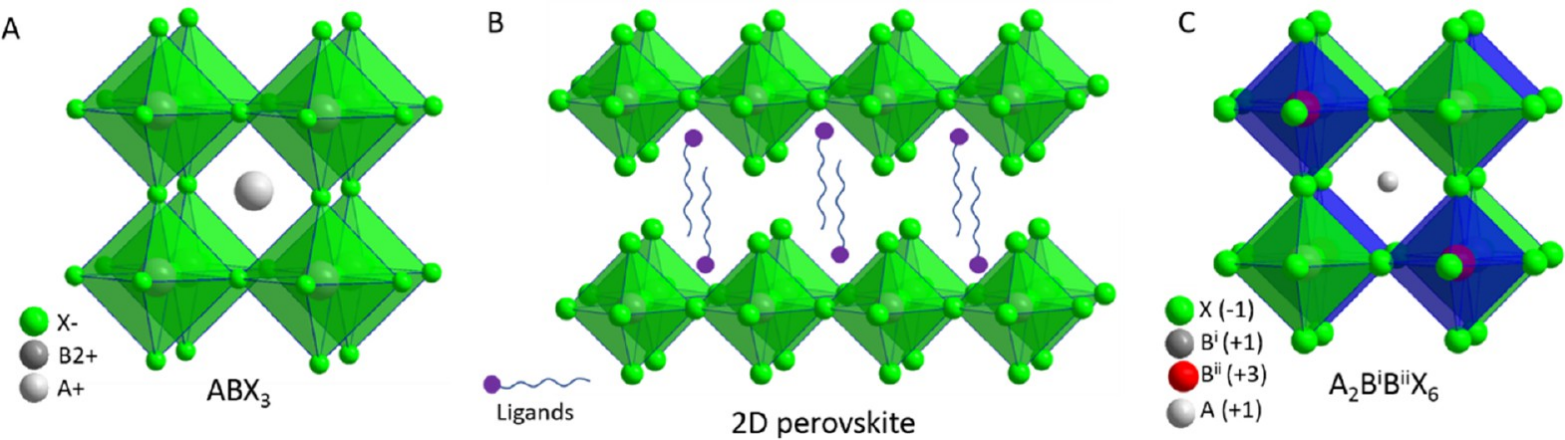
Illustrations of cubic crystal structure of (A) 3D perovskites, (B) 2D-layered perovskites, and (C) 3D double perovskite.

As illustrated in Figure 2, currently, MHP NCs are undergoing further chemical engineering in connection with shape-controlled synthesis using different precursors and ligands, surface functionalization to induce additional functionality (for example, chirality), metal-ion doping, and search for Pb-free NCs alternatives, phase stability (thermal and moisture), and self-assembly. All of these research lines are aimed toward improving and stabilizing their optical properties. Over the years, numerous excellent reviews have been published on MHP NCs, regarding their colloidal chemistry, optical properties (linear and nonlinear), and potential applications.^21−23,36,37,41,46,52,92−94,98−116^ However, there is no extensive literature review covering the entire spectrum of research into aspects of MHP NCs, from synthesis and fundamental properties to device applications and related challenges. It has already been over 5 years since MHP NC research has started, and it has quickly emerged as an important field in contemporary nanoscience and nanotechnology, a field that is still rapidly growing. We have therefore identified the need for a comprehensive literature review on current research lines and future prospects of MHP NCs, not only to guide currently active researchers of this field but also to inspire a younger generation of researchers to join this exciting research field. To realize this, we have put together our expertise to provide a broad overview of currently available knowledge on various aspects of MHP NCs. This review article provides comprehensive and up to date developments in the synthetic methods for the shape-controlled synthesis of MHP NCs (both Pb and Pb-free), their surface chemistry, post-synthetic surface passivation, surface functionalization, self-assembly, and optical properties along with potential applications.

**Figure 2:**
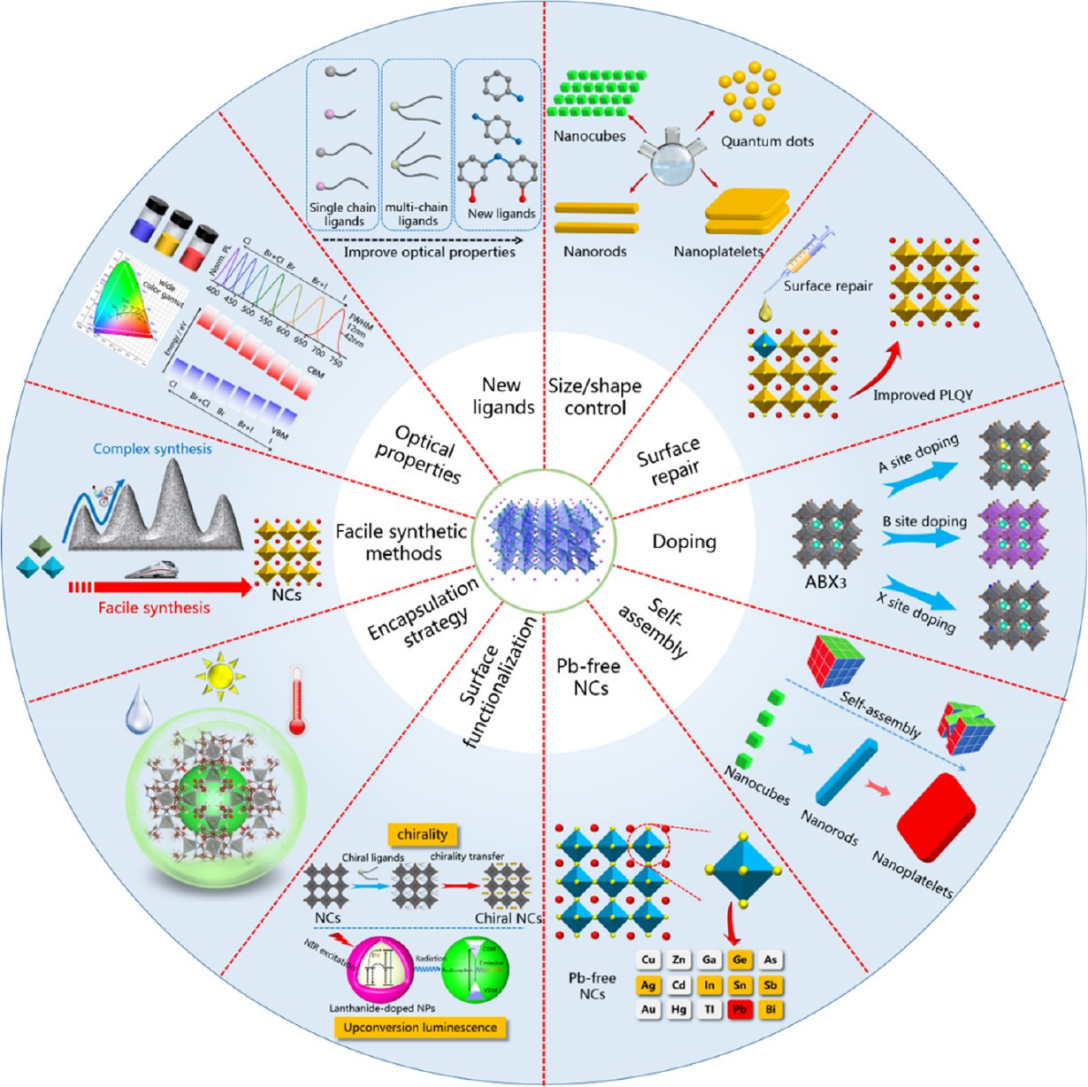
Schematic overview of the current research directions on the chemistry of colloidal MHP NCs.

We have organized this review into 11 main parts. (1) Colloidal synthesis of LHP NCs includes a brief history of colloidal synthesis of LHP NCs and a discussion on general approaches developed over the years for their shape/size-controlled (nanocubes, nanoplatelets and nanowires) synthesis and post-synthetic ion exchange for compositional tuning, along with post-synthetic shape transformations. We also discuss *in situ* synthesis approaches to obtain LHP NCs on a substrate. (2) Surface chemistry and post-synthetic surface treatment of LHP NCs improves their optical properties and provides our current understanding of ligand chemistry on LHP NC surface and passivation. (3) We discuss recent advances on 0D Cs_4_PbBr_6_ NCs, regarding their syntheses, phase transformations and origin of their green photoluminescence. (4) Surface coating strategies are used to enhance the stability of LHP NCs toward humidity, heat and harsh environments. (5) We then discuss various possible metal combinations to synthesize Pb-free perovskite NCs. (6) We provide a summary of LHP NCs doped (A- and B-sites) with various other metal ions to improve their optical properties as well as their phase stability. Special emphasis is paid to Mn^2+^-doped LHP NCs. (7) We provide a summary of self-assembly strategies employed for the fabrication of LHP nanocube superlattices. (8) We discuss the characterization of LHP NCs and their assembly by TEM and X-ray scattering techniques. In this section, we describe the challenges associated with characterization of LHP NCs by TEM due to electron-beam-induced degradation. In addition, we discuss X-ray scattering analysis of LHP NC degradation. (9) We discuss the optical properties of MHP NCs, such as their PL, quantum-confinement effects, chirality, and ultrafast charge carrier dynamics. (10) We also discuss the optical studies of quantum dots and nano- and microcrystals at the single-particle level. (11) In the last section, we offer an up to date research progress on various potential applications of MHP NCs, including lasers, LEDs, photodetectors, field-effect transistors (FETs), photovoltaics, and photocatalysis. In addition, an outlook is provided at the end of each section, along with an overall outlook at the end of the article.

## Shape-Controlled Synthesis of MHP NCs

### Evolution of Different Synthesis Methods

**Figure 3:**
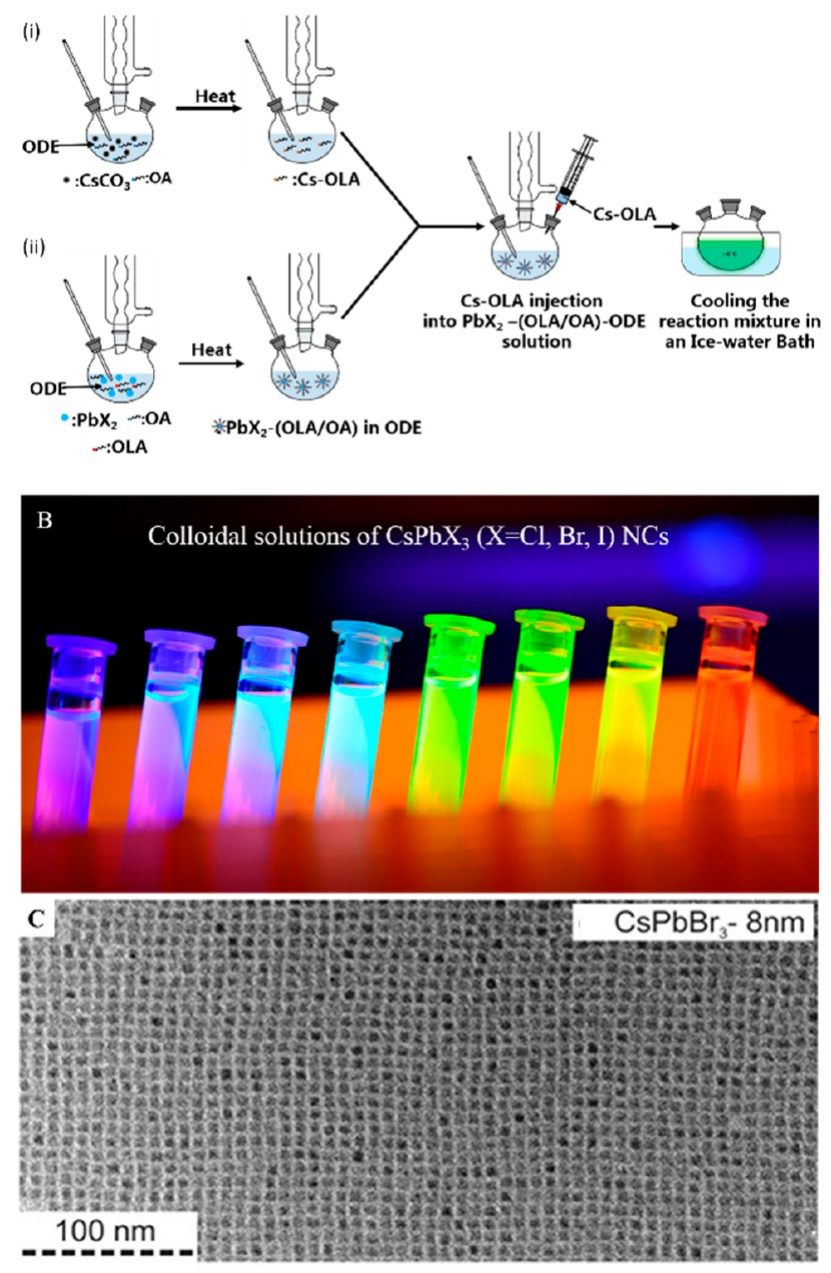
(A) Schematic illustrations of HI synthesis of colloidal CsPbX_3_ NCs. The synthesis relies on the injection of presynthesized Cs-oleate into a reaction solution (PbX_2_ dissolved in 1-octadecene using oleylamine and oleic acid) at high temperature. (B) Photographs of the colloidal solutions of CsPbX_3_ NCs synthesized by the HI method. Photo courtesy of Dr. Loredana Protesescu. (C) TEM images of the corresponding CsPbBr_3_ NCs. Panel C is reprinted from ref (14). Copyright 2015 American Chemical Society. Further permissions related to the material excerpted should be directed to the ACS.

The success of colloidal MHP NCs has resided mainly in the ability to synthesize them with excellent control over their shape, size, and composition, as well as with high quality.^14,22,23,36,47,52,85,98,105,117^ Part of this success stems from the fact that these systems, as soon as they were approached, had largely benefited from the knowledge on conventional colloidal nanocrystals that had accumulated over the past few decades, especially on their synthesis, the study of their fundamental properties, and their device applications.^58,118−124^ On the other hand, MHPs have been known for a very long time, but their connection with the NC world has come only in relatively recent times. As a matter of fact, the fabrication and optical properties of layered MHPs were reported long before (in the 1990s) the realization of their great potential for applications in devices, especially for photovoltaics.^125−128^ Along the line of conventional colloidal QD photovoltaics (PVs), Im *et al.* explored MAPbI_3_ NCs in a TiO_2_ matrix as a potential sensitizer for PVs in 2011.^129^ In their work, the NCs were synthesized on a nanocrystalline TiO_2_ surface by spin-coating the perovskite precursor solution. This was probably one of the early works to inspire the colloidal chemistry research community to investigate the solution-phase synthesis of colloidal MHP NCs. In 2014, Schmidt *et al.* reported the synthesis of MAPbBr_3_ perovskite nano/microcrystals.^66^ Their synthesis relied on the use of medium-length alkyl chain organic ammonium cations (octylammonium bromide and octadecylammonium bromide) as capping ligands to obtain colloidal MAPbBr_3_ NCs *via* the solvent (acetone)-induced reprecipitation of MABr and PbBr_2_ precursors. The prepared MAPbB_3_ nano/microcrystals exhibited green emission with a PLQY of ∼20%. The ligands played a critical role in limiting the crystallization to obtain colloidal NCs, as otherwise the precursors would precipitate out to form non-emissive or (weakly emissive) large bulk crystals. Interestingly, a similar concept had been employed previously to obtain 2D-layered halide perovskites on substrates and perovskite colloidal dispersions.^130^ In a subsequent work, Gonzalez-Carrero *et al*.^25^ further improved the PLQY of these NCs to 83% by optimizing the ligand concentration. However, the morphology of the perovskite colloids was unclear until the colloidal synthesis of well-defined CsPbX_3_ NCs reported by Protesescu *et al.* in 2015.^14^ They synthesized the CsPbX_3_ NCs by adapting a hot-injection strategy (Figure 3). Interestingly, HI has been used for more than two decades for CdSe^58^ and since then also for other conventional colloidal NCs (Pb chalcogenides, In pnictides, *etc*.).

Protesescu *et al*. were able to tune the size of the NCs by varying the reaction temperature and thus explored the quantum size effects in this class of NCs. This work lays the foundation for the shape-controlled synthesis of MHP NCs. This pioneering work clearly highlighted that LHP NCs have narrow emission spectra width with high PLQYs (up to 90%), and the PL peak position is precisely tunable across the visible spectrum (400–700 nm) of light by varying the halide (Cl, Br, I) composition and NC size (Figure 3). It is significant that LHP NCs, unlike conventional colloidal semiconductor QDs, exhibit such high PLQYs without any surface passivation. Later in 2015, Sichert *et al*.^16^ demonstrated the synthesis of organic–inorganic hybrid perovskite NPls with thickness control down to a monolayer by varying the ratio of long and short-chain ligands in the reprecipitation reaction. For such thin NPls, the quantum-confinement effects strongly affected their absorption and PL properties. The outstanding optical properties of both organic–inorganic and all-inorganic LHPs unveiled by these initial reports have greatly attracted the interest of researchers from various disciplines.

**Figure 4:**
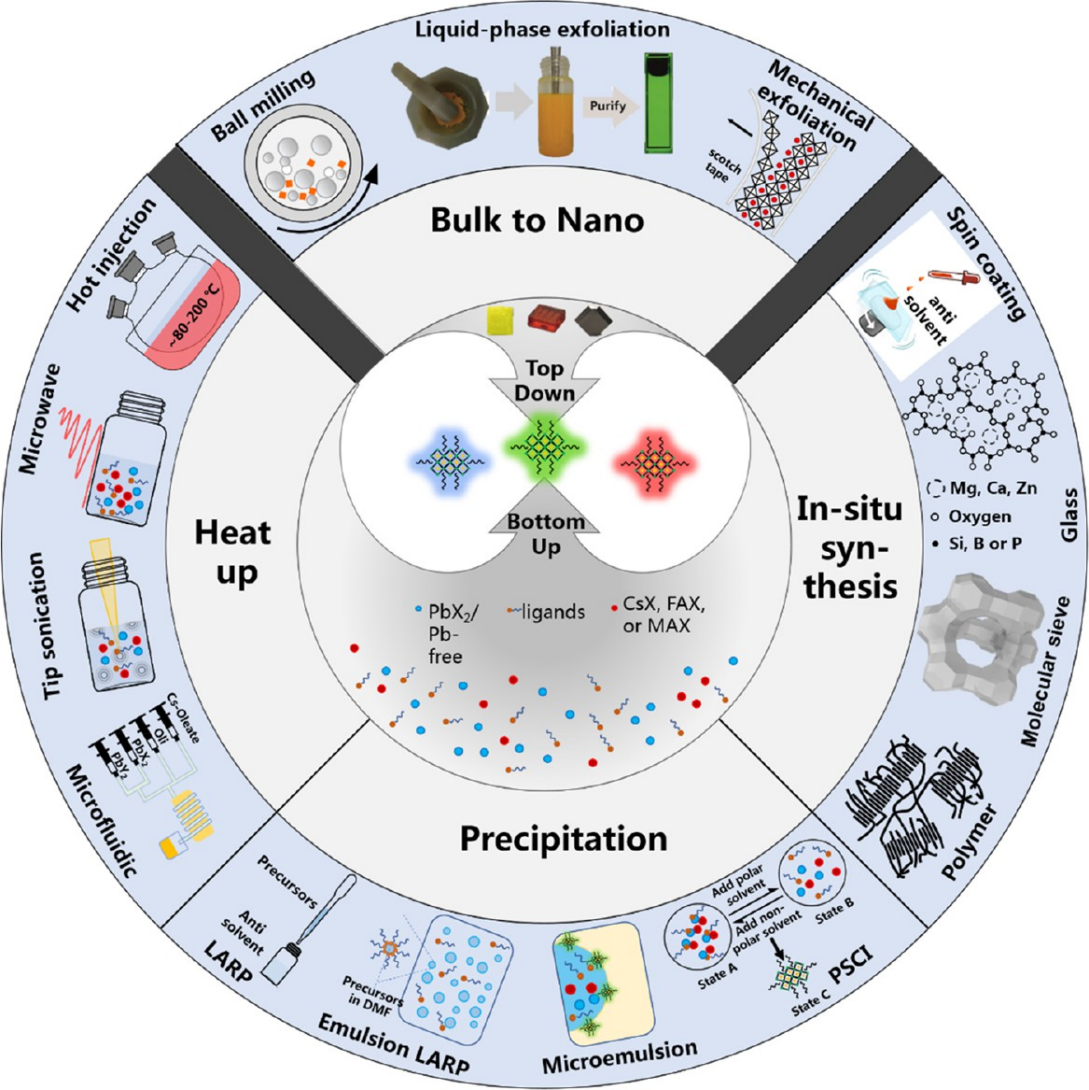
Schematic overview of various synthetic methods for MHP NCs. These methods can be generally classified into either “top-down” or “bottom-up”. The bottom-up methods can be further classified into three different subcategories (heat-up, precipitation, and *in situ* synthesis) depending on the type of reaction. PSCI, polar solvent-controlled ionization; LARP, ligand-assisted reprecipitation.

Over the last few years, significant efforts have been devoted to developing facile and reliable synthesis methods for MHPs. As schematically illustrated in Figure 4, these methods can be mainly classified into either “bottom-up” or “top-down” approaches based on the growth process.^131,132^ The bottom-up approaches can be further subclassified into three different categories based on the nature of the synthesis: (1) heat-up, (2) reprecipitation, and (3) *in situ* synthesis. Among all the strategies illustrated in Figure 4, HI and LARP have been the most frequently used methods for the synthesis of MHP NCs. As illustrated in Figure 3A, the HI synthesis of CsPbX_3_ NCs generally relies on the injection of presynthesized Cs-oleate into a reaction mixture containing PbX_2_ ligands in 1-octadecene at high temperatures and inert atmospheres, followed by immediate quenching of the reaction with an ice bath.

This method generally produces high-quality monodisperse CsPbX_3_ NCs with high PLQY, and this can also be adapted to the synthesis of Pb-free perovskite NCs using suitable precursors (refer to NANOCRYSTALS OF LEAD-FREE PEROVSKITE-INSPIRED MATERIALS). Over the years, the HI synthesis of MHP NCs has undergone further optimization with different precursors and ligands to achieve better stability and shape control. However, this method is tedious and requires high temperatures and inert atmospheres, which limits cost-effective mass production. Alternatively, researchers have adapted a few other methods such as tip sonication,^30^ microwave irradiation,^133^ ball-milling,^131^ and solvothermal methods^134^ for the synthesis of MHP NCs at atmospheric conditions. These are single-step bottom-up synthesis approaches, in which all the precursors and ligands are mixed in a solvent and then reacted by applying heat (solvothermal synthesis, which is very similar to HI) or by tip sonication or microwave irradiation at atmospheric conditions. Nevertheless, the temperature in the reaction medium increases during ultrasonication or microwave irradiation, promoting the reaction.

**Figure 5:**
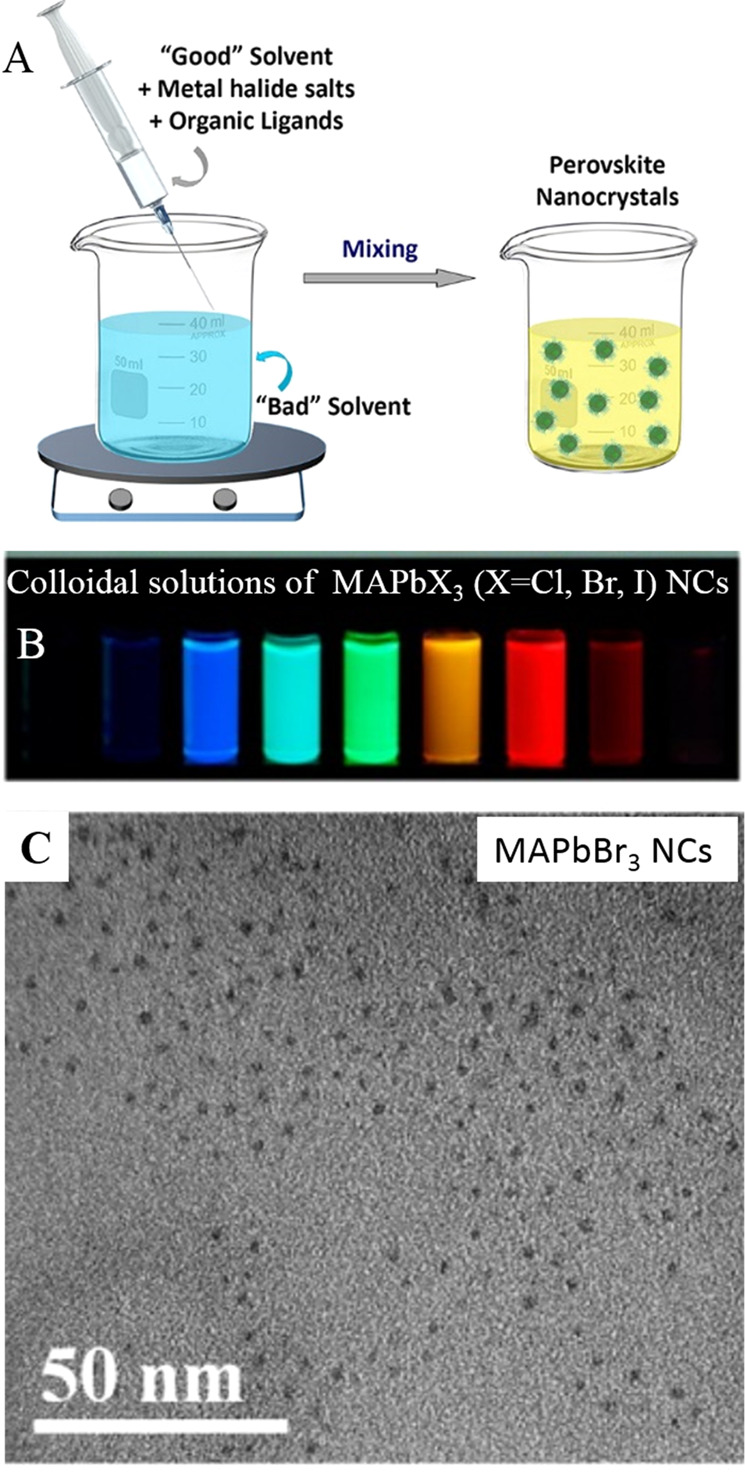
(A) Schematic illustrations of the synthesis of colloidal MAPbX_3_ NCs by the LARP approach. Reprinted from ref (52). Copyright 2019 American Chemical Society. Further permissions related to the material excerpted should be directed to the ACS. The synthesis relies on dropping precursor powders and ligands dissolved in a good solvent (such as DMF or DMSO) into a poor solvent (such as toluene or hexane). (B) Photographs of the colloidal solutions of MAPbX_3_ NCs synthesized by the HI method. (C) TEM images of the corresponding MAPbBr_3_ NCs. Panels B and C are reprinted from ref (29). Copyright 2015 American Chemical Society.

The inherent ionic nature of perovskites has enabled the synthesis of high-quality MHP NCs by the LARP approach in ambient atmosphere at room temperature. The reprecipitation approach has been known for centuries, and it has been used to prepare organic nanoparticles.^135−137^ This approach relies on the spontaneous crystallization of substances upon reaching a supersaturated state, which can be achieved by lowering the temperature, by solvent evaporation, or by the addition of a poor solvent in which the solubility of the substance is low. If this is carried out in the presence of ligands, nucleation and growth of the precipitate can be controlled, and this is called the LARP process. In early 2015, Zhang *et al*.^29^ initially employed this LARP approach to synthesize strongly luminescent colloidal MAPbX_3_ (X = Cl, Br, I) NCs at room temperature. In this approach, a solution of perovskite precursors (such as MAX, FAX, CsX, along with PbX_2_) and ligands (alkylamines and alkyl carboxylic acids) dissolved in a good solvent such as dimethylformamide (DMF) or dimethyl sulfoxide (DMSO) is dropped into a poor solvent (such as toluene or hexane), inducing the instantaneous formation of ligand-capped colloidal perovskite NCs (Figure 5A; see movie S1). The LARP approach generally yields either spherical NCs (Figure 5C) or nanoplatelets.^16,19^ The size of the MAPbBr_3_ NCs is tunable by varying the temperature at which LARP is carried out, as shown by Huang *et al*.^138^ Yet, there is still a debate on whether the spherical NCs are perovskites or Pb clusters that result from electron-beam-induced degradation of perovskite NCs (movie S2).^16,30,139^ The LARP approach has been further updated into emulsion synthesis, which enabled the purification of MAPbBr_3_ NCs by precipitation into solid-state light-emitting powder form.^138^ This can be redissolved into solvents for processing thin-film devices.^140,141^ This LARP approach has also been extended to all-inorganic MHP NCs.^53,77^ However, the level of shape control achieved by LARP is still lagging far behind that of the HI synthesis. As illustrated in Figure 2, currently, the synthesis of MHP NCs is undergoing further fine-tuning in connection with shape control using different precursors and ligands, surface functionalization to induce additional functionalities (for example, chirality), and metal-ion doping, moving the focus toward Pb-free NCs, phase stability (thermal and moisture), and self-assembly. All these research lines are aimed toward improving the optical properties of NCs or finding alternative, less toxic compositions while keeping optical performances high. Despite significant advances in the synthesis of MHP NCs, only limited shape control has been achieved, as mainly NCs, NPls, and NWs have been frequently reported. In the following, we discuss the state of the art synthesis of these three morphologies.

### Nanocubes

Nanocubes are the most explored MHP NCs in terms of their synthesis, characterization, and investigation for potential applications.^14,52,53,89,142^ Over the last 5 years, there has been significant progress toward the development of reliable and scalable synthetic approaches for MHP nanocubes with tunable composition and high PLQY.^14,30,52,53,134,143,144^ As a result, these nanocubes have already shown great promise for LEDs, lasers, and solar cells, as compared with other MHP morphologies and nanostructures.^42,89,90,142^ In general, perovskite precursors often tend to precipitate to form NCs with cubic shapes at high reaction temperatures, while they tend to crystallize into nanoplatelet morphologies at relatively low reaction temperatures. This temperature dependence is now better understood in terms of acid/base equilibria regulating the protonation/deprotonation of the alkylamine ligands used in the synthesis competing with Cs^+^ ions for their inclusion to the facets of the growing NCs.^145^ In fact, CsPbX_3_ perovskite nanocubes were initially synthesized using a well-known HI method, and it is still the most frequently used method to synthesize MHP NCs (Figure 3 and movie S3: large-scale synthesis of CsPbBr_3_ nanocubes; the hot injection is realized here by creating a reduced pressure in the flask and opening the valve of the dropping funnel).^14^ In this method, PbX_2_ precursors were first dissolved in octadecene, followed by the injection of Cs-oleate at high temperature and inert atmosphere. It is worth mentioning that the reaction has to be quickly quenched with an ice bath upon the injection of Cs-oleate; otherwise, a prolonged reaction time leads to the formation of nanowires as side products (the reader should consult the nanowires section for additional details).^75^ This method generally yields monodisperse CsPbX_3_ nanocubes, and the halide composition of the nanocubes is easily tunable by varying the ratio of PbX_2_ precursors in the reaction medium. Although the initial studies suggested that these CsPbX_3_ nanocubes exhibit cubic structures,^14,30,53^ CsPbBr_3_ nanocubes were later found to have an orthorhombic crystal structure.^143,146,147^ The Br- and I-based perovskite NCs generally feature high PLQY (near-unity has been reported), while the Cl-based NCs suffer from lower PLQYs.^14,30,57^ Nevertheless, recent studies have shown that post-synthetic treatment with metal chloride salts can significantly improve the PLQY of CsPbCl_3_ nanocubes up to near-unity.^87,148^ However, it is still unclear whether metal ion doping or the surface passivation with chloride ions or both leads to the observed PLQY enhancement.^86^

**Figure 6:**
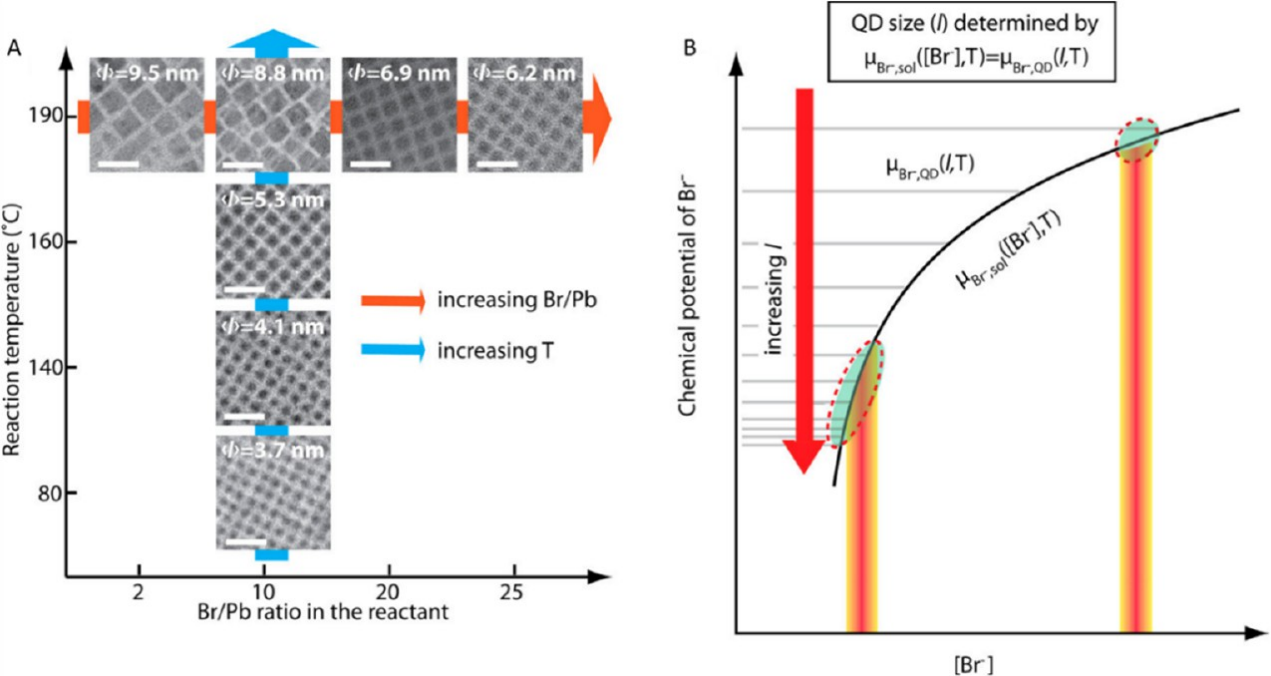
Size control of CsPbBr_3_ perovskite nanocubes *via* thermodynamic equilibrium in hot-injection synthesis. (A) Dependence of the size of CsPbBr_3_ nanocube on the Br to Pb ratio in the reaction medium and the reaction temperature. (B) Proposed model illustrating the determination of the nanocube size *via* equilibrium of Br^–^ between the nanocube lattice and the reaction medium. The nanocube size for a given concentration of Br^–^ ([Br^–^]) and temperature (*T*) is determined at which the chemical potentials (μ_Br^–^_) of Br^–^ in the reaction medium become equal. The inverse correlation between the nanocube size and the concentration of Br^–^ at a given temperature (*T*) can be clearly seen from the two marked (dotted circles) areas. Reproduced from ref (149). Copyright 2018 American Chemical Society.

In addition, the size of the CsPbX_3_ perovskite nanocubes is also tunable over a limited range *via* hot-injection synthesis. However, unlike conventional colloidal NCs, the size of the perovskite NCs is tunable by controlling the reaction temperature rather than the growth kinetics because of their fast (1–3 s) nucleation and growth. In general, the size of the perovskite nanocubes decreases with decreasing reaction temperature. For instance, Protesescu *et al*. synthesized monodisperse nanocubes of size range of 4–15 nm by hot-injection synthesis *via* temperature control (140–200 °C).^14^ Nevertheless, it should be noted that precursors crystallize into nanoplatelets at low reaction temperatures (<130 °C).^18^ For precise control over the size of quantum-confined CsPbX_3_ nanocubes, Dong *et al*.^149^ proposed a strategy based on the halide ion equilibrium between the nanocubes and the reaction medium, along with temperature control (Figure 6). In principle, the halide (X) to Pb ratio should be higher for small (strongly quantum-confined) CsPbX_3_ nanocubes. As the Br^–^ ions diffuse in and out of the crystal lattice with a low kinetic barrier, the size of the resulting nanocube depends on the variation of the Br^–^ equilibrium between the nanocube and the reaction medium. Therefore, at a given temperature, the increase in the Br/Pb ratio for a fixed amount of Cs^+^ and Pb^2+^ in the reaction medium leads to a decrease in the nanocube size (Figure 6A). Similarly, for a fixed Br/Pb ratio, the size of the nanocube decreases with decreasing reaction temperature (Figure 6A). This model was proposed based on the Br^–^ equilibrium between the nanocube lattice and the reaction medium and is consistent with the experimentally observed (from TEM analysis shown Figure 6A) correlation between nanocube size and Br/Pb ratio (Figure 6B). This method has received considerable attention regarding the preparation and study of the optical properties of size controlled quantum-confined nanocubes.^150−153^ In addition, several other potential methods have also been reported for the growth of size-controlled quantum-confined CsPbBr_3_ nanocubes.^79,145,154^ For instance, Pradhan and co-workers showed that the size of the CsPbBr_3_ nanocubes can be reduced down to ∼3.5 nm by increasing the amount of oleylamine–HBr (OLA–HBr) in the reaction medium at a fixed temperature (160 °C).^79^ To achieve a better understanding of the role of ligands (OLA and OA) in controlling the shape and size of perovskite NCs, Almeida and co-workers performed a systematic synthetic study by varying the ratio between OLA and OA and correlated with the size, shape, and distribution of the resultant CsPbBr_3_ NCs.^145^ They found that a high concentration of oleylammonium species in the reaction medium leads to the formation of nanoplatelets, whereas a low concentration results in nanocubes. In addition, they were able to prepare monodisperse CsPbBr_3_ nanocubes with sizes ranging from 4.0 to 16.4 nm by varying the OLA/OA ratio along with reaction temperature. Despite the successful synthesis of small nanocubes (<20 nm), precise control over the size of CsPbX_3_ nanocubes with sizes above 20 nm is still challenging.

**Figure 7:**
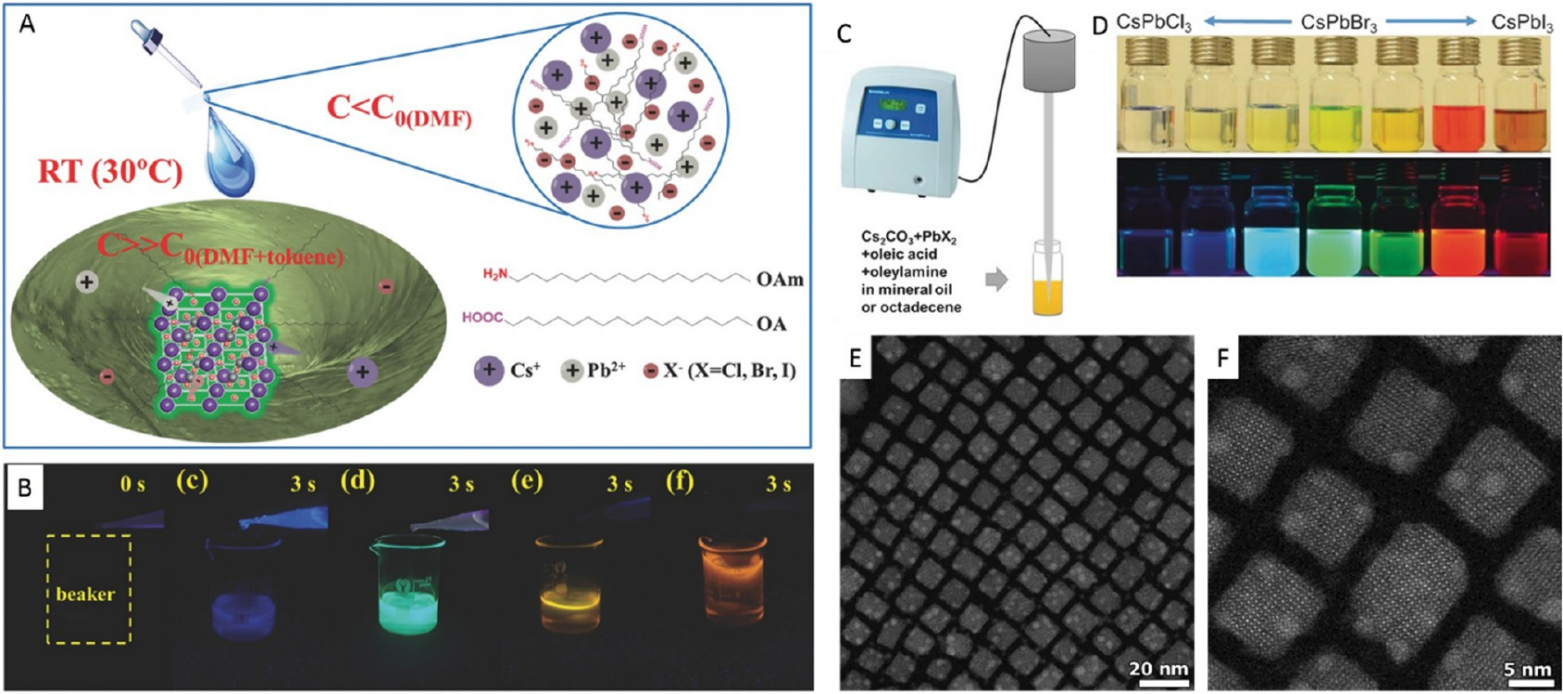
Highly luminescent CsPbX_3_ (X = Cl, Br, and I) nanocubes *via* supersaturated recrystallization at RT and single-step ultrasonication approaches. (A) Schematic illustration of the RT synthesis of CsPbX_3_ nanocubes. The precursors (Cs^+^, Pb^2+^, and X^–^ ions) crystallize into perovskite nanocubes under ambient conditions within 10 s after having been transferred from a good solvent (DMF) to a bad solvent (toluene). (B) Photographs of pure toluene (0 s) and the colloidal solutions of CsPbX_3_ nanocubes with different halide compositions formed within 3 s after the injection of corresponding DMF precursors into pure toluene under UV illumination in darkness. Panels A and B are reprinted with permission from ref (53). Copyright 2016 John Wiley & Sons, Inc. (C) Schematic illustration of the single-step synthesis of CsPbX_3_ perovskite nanocubes. (D) Photograph of the colloidal dispersions of CsPbX_3_ NCs with different halide (X = Cl, Br, and I) compositions under room light (top) and UV light (bottom). (E,F) Different magnification high-angle annular dark-field scanning transmission electron microscopy images of CsPbBr_3_ nanocubes obtained by ultrasonication approach. Panels C and D are adapted from ref (30). Copyright 2016 John Wiley & Sons, Inc.

Although the hot-injection method has been extensively used for the synthesis of inorganic perovskite nanocubes, it is tedious and generally carried out under inert conditions. Moreover, it requires an additional synthesis step for the Cs-oleate precursor. To overcome these limitations, several alternative methods, such as microwave irradiation,^133^ ultrasonication,^30^ solvothermal synthesis^134^, and LARP^53^ have been reported. For instance, Zeng and co-workers reported the early work on the RT synthesis of highly luminescent CsPbX_3_ perovskite nanocubes using the LARP method (Figure 7A,B).^53^ In this method, CsBr and PbBr_2_ precursors were first dissolved in DMF or DMSO along with OLA and OA ligands. The precursor solution was then added to toluene at RT to trigger the precipitation of brightly luminescent perovskite nanocubes within a few seconds, as shown in Figure 7B. The authors reported a PLQY of 95% for CsPbBr_3_ nanocubes prepared by this method. The emission color was easily tunable by the halide composition in the precursor solution in DMF. Nevertheless, this method required the use of polar solvents that can influence the stability of the prepared NCs. In 2016, Tong *et al*.^30^ reported the polar-solvent-free single-step synthesis of CsPbX_3_ nanocubes with controllable halide composition by ultrasonicating the precursor salts in the presence of ligands (Figure 7C,D). This is one of the easiest and fastest methods to obtain perovskite NCs. The emission color of the prepared nanocubes is easily tunable by varying the ratio of different halide precursors in the reaction medium. The nanocubes prepared by this approach are nearly monodisperse and exhibit high PLQY. This method was further extended to the preparation of perovskite nanowires^22^ and nanorods.^155^ In 2017, Chen *et al.* reported the solvothermal synthesis CsPbX_3_ NCs.^134^ In this method, the precursors and ligands were loaded in a Teflon-lined autoclave and then heated at 160 °C for 30 min. The obtained nanocubes appeared to be rather monodisperse with a PLQY up to 80%. Zhai *et al.* further extended this method to CsPbBr_3_ nanoplatelets using presynthesized Cs-oleate as the precursor.^156^

**Figure 8:**
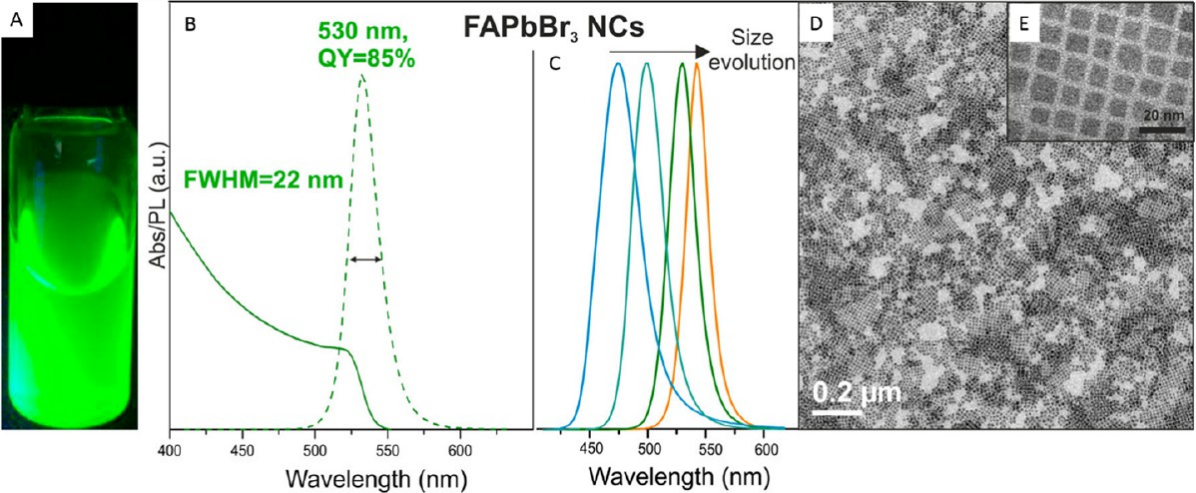
Synthesis of FAPbBr_3_ nanocubes by hot injection. (A) Photograph of colloidal solution of FAPbBr_3_ nanocubes in toluene under UV light illumination. (B) UV–vis absorption and PL spectra of FAPbBr_3_ nanocubes with a PL peak maximum at 530 nm. (C) PL spectra for FAPbBr_3_ NCs of different sizes. The emission peak red shifts with increasing size from 5 to >50 nm. (D,E) TEM images of FAPbBr_3_ nanocubes at two different magnifications. Reproduced from ref (68). Copyright 2016 American Chemical Society. Further permissions related to the material excerpted should be directed to the ACS.

In comparison to the many studies on inorganic perovskite NCs, organic–inorganic hybrid perovskite nanocubes have been rarely reported.^68,157−161^ In 2016, Vybornyi *et al*.^158^ demonstrated a polar-solvent-free colloidal synthesis of MAPbBr_3_ perovskite NCs by the HI method. They were able to tune the morphology from nanocubes to nanoplatelets and nanowires by varying the reaction parameters. In 2019, Zhang *et al.* extended this method to the synthesis of monodisperse MAPbI_3_ nanocubes.^160^ The main problem associated with these MA-based perovskites is their chemical decomposition, which limits their applications. Alternatively, Protesescu *et al.*(68) reported stable and bright green emissive FAPbBr_3_ nanocubes by the hot-injection method (Figure 8). In this method, FA and Pb acetate precursors were first dissolved in octadecene in the presence of OA, followed by the injection of presynthesized oleylammonium bromide (OLABr) at 130 °C. This method is slightly different from the typical hot-injection method used for the synthesis of CsPbX_3_ NCs, where PbBr_2_ was used as precursor for both Pb and Br. This hot-injection method, in which FA-oleate was injected into PbBr_2_–OA–OLA solution, produced FAPbBr_3_ nanocubes with a much broader size distribution. The nanocubes prepared by this method are rather monodisperse (12 nm) with the PL peak at 530 nm and QY of 85% (Figure 8B). In addition, the authors demonstrated that the size of the FAPbBr_3_ nanocubes can be tuned from 5 to 50 nm by adjusting either the amount of OLABr or the reaction temperature, and thus the emission peak is tunable from 470 to 545 nm (Figure 8C).^68^ The purification process after the synthesis of perovskite NCs is critical in order to recover monodisperse NCs. Very recently, Li *et al*.^162^ proposed size-selective precipitation using a mixture of ethyl acetate and methyl acetate (2:1 volume ratio) to obtain strongly confined nanocubes of different sizes. The precipitation process can be repeated multiple times to obtain FAPbBr_3_ nanocubes of different sizes. Hybrid perovskite NCs have also often been prepared by the LARP method, and the resulting NCs possess either spherical or nanoplatelet morphology.^29,66^ However, there is still debate on whether the spherical particles obtained by the LARP method are perovskites or whether they are the e-beam-induced degradation product of perovskite NPls (see Electron Microscopy section). In 2017, Levchuk *et al*.^159^ reported the RT synthesis of brightly luminescent FAPbX_3_ nanocubes by the LARP method. The synthesis relies on the rapid injection of a precursor solution (PbX_2_ and FAX dissolved in DMF along with OA and OLA) into chloroform. The obtained nanocubes exhibit PLQYs up to 85%. They were able to tune the morphology from nanocubes to NPls of different thicknesses by varying the OLA/OA ratio. However, the cubic morphology of the particles obtained in this approach is not as perfect as that of the nanocubes synthesized by the hot-injection method. A few months later, Minh *et al*.^163^ reported a RT synthesis of FAPbX_3_ nanocubes by LARP method, in which presynthesized PbX_2_–DMSO complexes were used as precursors. In this approach, the precursors (FAX and PbX_2_–DMSO complex) were first dissolved in DMF along with OLA, followed by injection of the precursor solution into a mixture of toluene and OA. They were able to tune the size distribution of the nanocubes by varying the amount of OLA used in the reprecipitation reaction. The quality of the nanocubes prepared by this approach appeared to be as good as that of the nanocubes prepared by hot injection. Such a purification approach is also useful for the size-selective separation of inorganic perovskite nanocubes, as demonstrated by Forde *et al.*(154) Very recently, Zu *et al*. reported the synthesis of FAPbBr_3_ NCs by the LARP approach using sulfobetaine-18 (SBE-18) as the capping ligand.^164^ The authors claimed that the FAPbBr_3_ nanocubes prepared using SBE-18 ligands (PLQY ≈ 90.6%, fwhm ≈ 20.5 nm) exhibited PLQYs (as well as green color purity) higher than those of OLA/OA-capped FAPbBr_3_ nanocubes (PLQY ≈ 83.2%, fwhm ≈ 24 nm) prepared under similar conditions.

**Figure 9:**
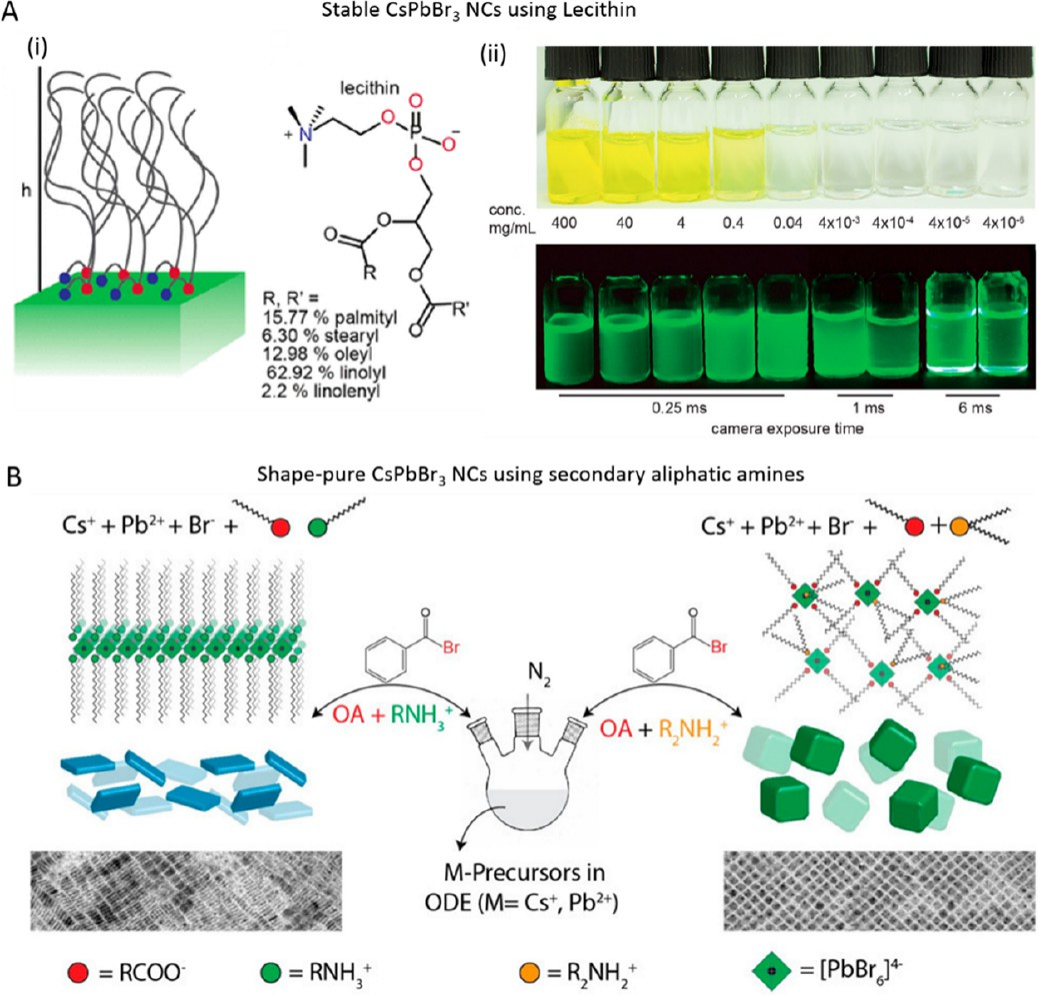
(A) Schematic illustration showing the synthesis of CsPbBr_3_ NCs using primary (left) and secondary (right) aliphatic amines. The TEM images showing the resultant products in the respective reactions. Reproduced from ref (170). Copyright 2019 American Chemical Society. Further permissions related to the material excerpted should be directed to the ACS. (B) (i) Schematic of lecithin ligands forming a brushlike structure on the NC surface, and the “h” indicates the brush height (left) and chemical structure of lecithin and statistical occurrence of side chains (R, R′) in soy lecithin (right). (ii) Photographs of the colloidal solutions of lecithin-capped CsPbBr_3_ NCs at various concentrations under daylight (top) and UV light (bottom). Reproduced from ref (143). Copyright 2018 American Chemical Society. Further permissions related to the material excerpted should be directed to the ACS.

In general, capping agents play a critical role in controlling the shape of NCs during colloidal synthesis, the properties of the NCs, as well as their colloidal stability.^165−167^ Recently, there has been a growing interest in the exploration of different ligands for shape-controlled synthesis and stability of perovskite NCs with high PLQYs.^168−174^ For instance, in 2017, Liu *et al*.^175^ reported the use of trioctylphosphine–PbI_2_ (TOP–PbI_2_) as a precursor for the synthesis of phase-stable CsPbI_3_ nanocubes with near-unity PLQY. Their approach relies on the injection of presynthesized TOP–PbI_2_ precursor into a reaction mixture containing Cs_2_CO_3_, OA, and OLA in octadecene (ODE) at different temperatures that are set to achieve a desired size for nanocubes. The authors found that these CsPbI_3_ nanocubes exhibited higher stability as well higher PLQY compared to those of the nanocubes prepared without the use of the TOP ligand. The higher PLQY was attributed to the removal of nonradiative traps upon strong binding of TOP to the nanocube surface. Around the same time, Wu *et al*.^169^ further showed that the incorporation of a highly branched capping ligand, trioctylphosphine oxide (TOPO), along with traditional oleic acid/oleylamine ligand, leads to monodisperse CsPbX_3_ nanocubes at high temperature (260 °C). Otherwise, the reaction led to large aggregates at such temperatures in the absence of TOPO. More importantly, the authors found that the TOPO-protected CsPbBr_3_ nanocubes exhibited superior stability in ethanol as compared to that of OA/OLA-capped CsPbBr_3_ nanocubes, regardless of the reaction temperatures at which they were synthesized. The most important factor in the selection of ligands is that they should bind strongly to the NC surface so that they do not detach during the washing process. However, this is not the case for OA/OLA-capped perovskite NCs, as their optical properties and applications are often hampered by the colloidal and structural instability caused by the desorption of ligands. To address this issue, Krieg *et al*.^171^ proposed zwitterionic capping ligands to enhance the stability and durability of CsPbBr_3_ nanocubes, and the authors named the corresponding NCs as “CsPbX_3_ (X = Cl, Br, I) nanocrystals 2.0”. The Cs and Pb precursors used in their synthesis are different from the ones used in the hot-injection synthesis of OA/OLA-capped CsPbX_3_ NCs. The synthesis used by Kreig *et al.* is based on the injection of presynthesized TOP-X_2_ into a mixture of presynthesized Cs-2-ethylhexanoate solution, Pb(II)-ethylhexanoate solution, and zwitterionic ligand (3-*N*,*N*-(dimethyloctadecylammonio)propanesulfonate) at 160 °C. Interestingly, the authors claimed that the morphology and optical properties of these nanocubes were preserved after several washing cycles. The enhanced stability of zwitterionic ligand-capped CsPbX_3_ NCs was attributed to the simultaneous coordination of each ligand molecule to the surface cations and anions of NC. In a subsequent work, the same group introduced another zwitterionic capping ligand, namely, soy-lecithin, a mass-produced natural phospholipid, to protect the surface of CsPbX_3_ (X = Cl, Br) nanocubes through tight binding to the cations and anions at the surface (Figure 9A-i).^170^ The ligand enabled the high-yield synthesis of CsPbX_3_ nanocubes with a long-term colloidal and structural stability in a broad range of colloidal concentrations (from a few mg mL^–1^ to >400 mg mL^–1^), as shown in Figure 9A-ii. They attributed such high colloidal stability to an increased particle–particle repulsion caused by branched chains and ligand polydispersity. In addition, the authors demonstrated the fabrication of micrometer-thick and homogeneous dense CsPbBr_3_ nanocube films in a single spin-coating step using ultraconcentrated colloidal solutions. Very recently, Wang *et al*.^173^ demonstrated the potential application of polyzwitterionic ligands for phase transfer of CsPbBr_3_ nanocubes from a nonpolar solvent to a polar solvent through ligand exchange. Such polyzwitterionic ligands on the NC surface enabled the stabilization of CsPbBr_3_ NCs in a wide range of solvents. These studies suggest that the long-chain molecules with multiple functional groups can serve as potential ligands for perovskite NCs with long-term colloidal stability. A similar ligand binding strategy was applied to obtain stable CsPbI_3_ NCs with near-unity PLQY using 2,2′-iminodibenzoic acid as the bidentate ligand.^172^

In addition, several groups showed that the chain length of alkylamines and carboxylic acids ligands plays an important role in the morphology of perovskite NCs.^143,176,177^ For instance, Pan *et al.* systematically studied the influence of the chain length of alkylamine and carboxylic acid ligands used in hot injection.^177^ They found an increase in the size of the CsPbBr_3_ nanocubes when the chain length of the carboxylic acid was shortened at high reaction temperatures. On the other hand, the replacement of OLA with a short-chain amine leads to a change in the morphology from nanocubes to nanoplatelets. However, it is not uncommon to have a small percentage of nanoplatelets in nanocube samples or *vice versa*. Very recently, Imran *et al*. reported the synthesis of shape-pure, nearly monodisperse nanocubes using secondary aliphatic amine ligands (Figure 9B).^143^ Interestingly, their synthesis yielded only nanocubes, regardless of the length of the alkyl chains, oleic acid concentration, and reaction temperature. As illustrated in Figure 9B, they proposed that the secondary ammonium ions do not bind to the surface of CsPbBr_3_ NCs as effectively as primary ammonium ions (oleylammonium in this case) due to steric hindrance, which limits the formation of nanoplatelets. This was further supported by the fact that the surface coverage (6–8%) of secondary ammonium cations is much lower than that of oleate molecules (92–94%), as revealed by nuclear magnetic resonance (NMR) measurements and X-ray photoelectron spectroscopy (XPS).

**Figure 10:**
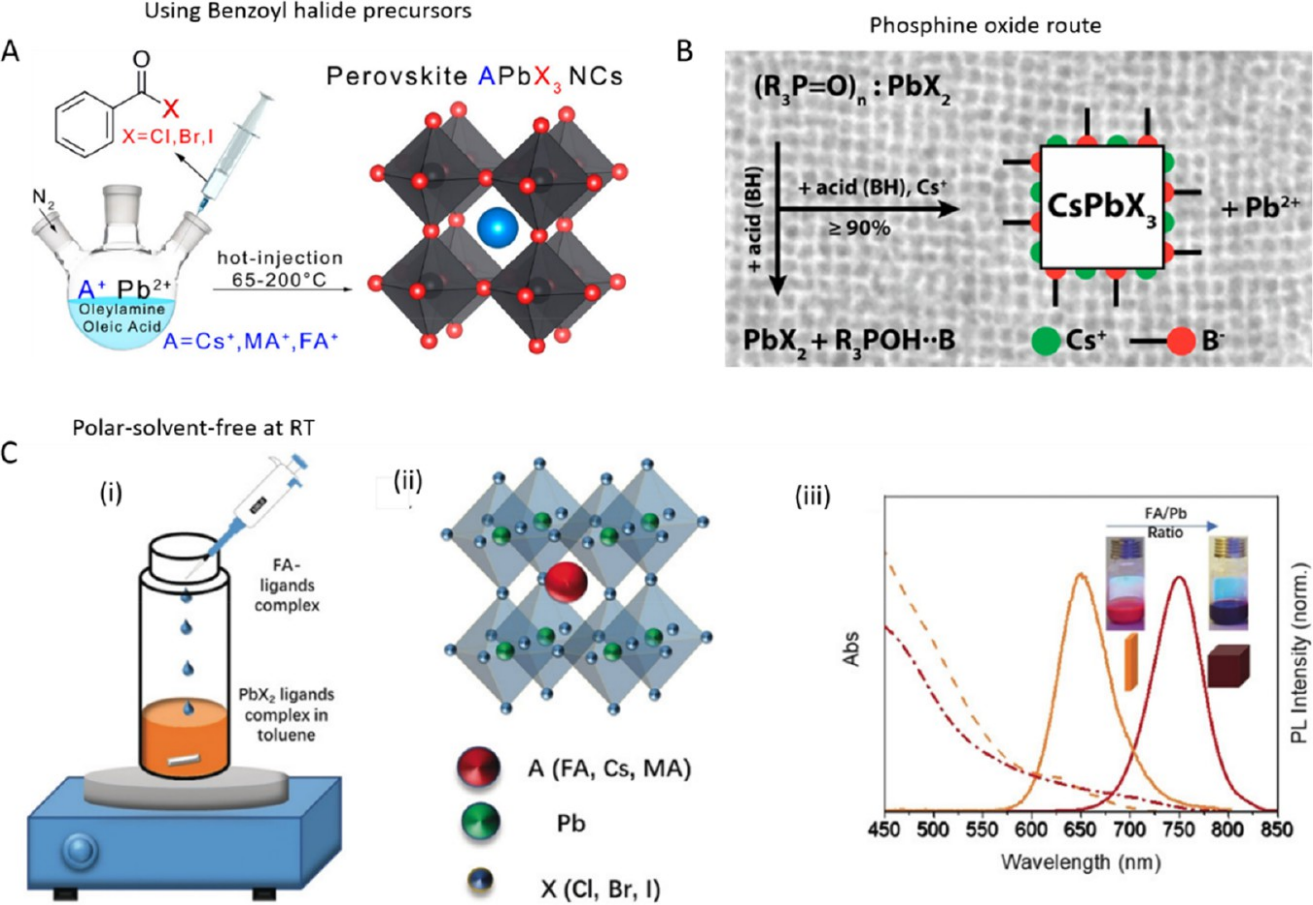
Reaction schemes of the colloidal synthesis of halide perovskite NCs using (A) benzoyl halide precursors. Reproduced from ref (178). Copyright 2018. American Chemical Society. Further permissions related to the material excerpted should be directed to the ACS. (B) trioctylphosphine oxide (TOPO) instead of aliphatic amines. Reprinted with permission under Creative Common [CC-BY] license from ref (179). Copyright 2018 American Chemical Society. (C) Schematic illustration of the polar-solvent-free synthesis of halide perovskite NCs at room temperature by spontaneous crystallization (i) and perovskite crystal structure (ii). The shape of the NCs depends on the precursor ratio (iii). Reprinted with permission under a Creative Commons CC BY license from ref (54). Copyright 2019 John Wiley & Sons, Inc.

Currently, colloidal syntheses of CsPbX_3_ NCs are undergoing further optimization using a variety of precursors and ligands, and many general methods are being developed for better control over their shape, composition, and polydispersity.^54,161,167,179−182^ In most synthesis methods that are in use for perovskite NCs, PbX_2_ salts are employed as precursors for both Pb and halide ions. This limits the precise control over the reactant species and thus the final chemical composition of colloidal perovskite NCs. To overcome this, Imran *et al*.^178^ reported the use of benzoyl halides as the halide precursors for monodisperse APbX_3_ NCs (in which A = Cs^+^, CH_3_NH_3_^+^, or CH(NH_2_)_2_^+^). Their method relied on the injection of benzoyl halide precursor into the reaction medium containing cesium carbonate (organic cation for hybrid perovskite NCs) and lead acetate trihydrate along with ligands at high temperature (Figure 10A; also note that a similar approach using instead tris-trimethylsilyl bromide or chloride as halide precursor was employed by Creutz *et al.* in the synthesis of double halide perovskite NCs).^183^ This approach enabled one to independently tune the amount of both cations (A^+^ and Pb^2+^) and halide (X^–^) precursors in the synthesis. Interestingly, this method produced nearly monodisperse MAPbX_3_ nanocubes, which seems difficult to obtain using other synthesis methods. In addition, the same group developed an amine-free synthesis of CsPbBr_3_ nanocubes by complete replacement of the routinely used aliphatic amines with TOPO (Figure 10B).^179^ Their synthesis relied on the injection of Cs-oleate into a reaction mixture containing PbBr_2_ along with TOPO and OA. This reaction yielded only nanocubes regardless of the tested reaction conditions. This was attributed to the absence of primary amines in the reaction medium. The TOPO helped to dissolve the PbBr_2_ in the reaction medium as well as to establish an acid–base equilibrium with OA in a way similar to the OA–OLA system (Figure 10B).^179^ Therefore, the acidity of the reaction environment controlled the reactivity of the PbX_2_ precursor and thus regulated the size of the NCs. Interestingly, only Cs-oleate ligands were present on the surface of the NCs, and they were bound dynamically to the NC surface; therefore, an optimum concentration of ligands was necessary to achieve high PLQY. Despite achieving excellent control over the shape purity and polydispersity of ABX_3_ perovskite NCs, most discussed synthesis methods require inert atmosphere and high temperature. In contrast, Polavarapu and co-workers demonstrated a polar-solvent-free synthesis for ABX_3_ NCs at ambient conditions through spontaneous crystallization of precursor–ligand complexes in a nonpolar organic medium (Figure 10C-i).^54^ Furthermore, the shape of perovskite NCs was controllable from nanocubes to nanoplatelets by varying the ratio of monovalent (*e.g*., formamidinium (FA^+^) and Cs^+^) to divalent (Pb^2+^) cation–ligand complexes (Figure 10C-iii). The authors demonstrated the versatility of this method by applying it to perovskite NCs of different compositions.

#### Isolation and Purification of Colloidal MHP Nanocubes

Colloidal ligand-stabilized NCs are usually extracted from crude reaction mixtures and purified by antisolvent precipitation.^58^ When the capping ligand layer is hydrophobic, a miscible polar solvent is used to flocculate the NCs, which are then isolated by centrifugation. This precipitative washing procedure removes excess ligand, residual reactants, and molecular byproducts and is an important step when the NCs are to be used in devices, such solar cells or light-emitting diodes that require charge transport through a deposited layer of nanocrystals.

Metal-halide perovskite nanocubes can degrade during the purification process. Bound ligands are in dynamic equilibrium with free ligands, and polar solvents can lower the kinetic barrier to ligand exchange and enhance ligand desorption.^84^ “Overwashing” can lead to irreversible aggregation, changes in morphology, a significant drop in photoluminescence, or even more significantly, changes in crystal phase or composition.^184,185^ For example, perovskite CsPbI_3_ nanocubes often transform to the yellow non-perovskite phase,^185,186^ and CH_3_NH_3_PbI_3_ (MAPI) nanocubes decompose into PbI_2_.^187^

Of course, one way to minimize degradation is to avoid the use of polar solvents, hence simply allowing the nanocubes to settle by centrifuging the crude reaction mixture at high speeds.^158,181,188^ This mostly works, but it often leaves a significant amount of nanocubes suspended in the supernatant, which are then discarded. A considerable residue of unbound ligand and low volatility reaction solvent (*i.e.*, octadecene) is also retained in the nanocube precipitate.^84,189^ This residue is a problem for device applications. It also creates challenges during characterization. TEM is difficult with so much excess hydrocarbon impurity, and free ligand contamination strongly interferes with the signal from bound ligand in analytical techniques like Fourier transform infrared (FTIR) spectroscopy and NMR spectroscopy.

**Figure 11:**
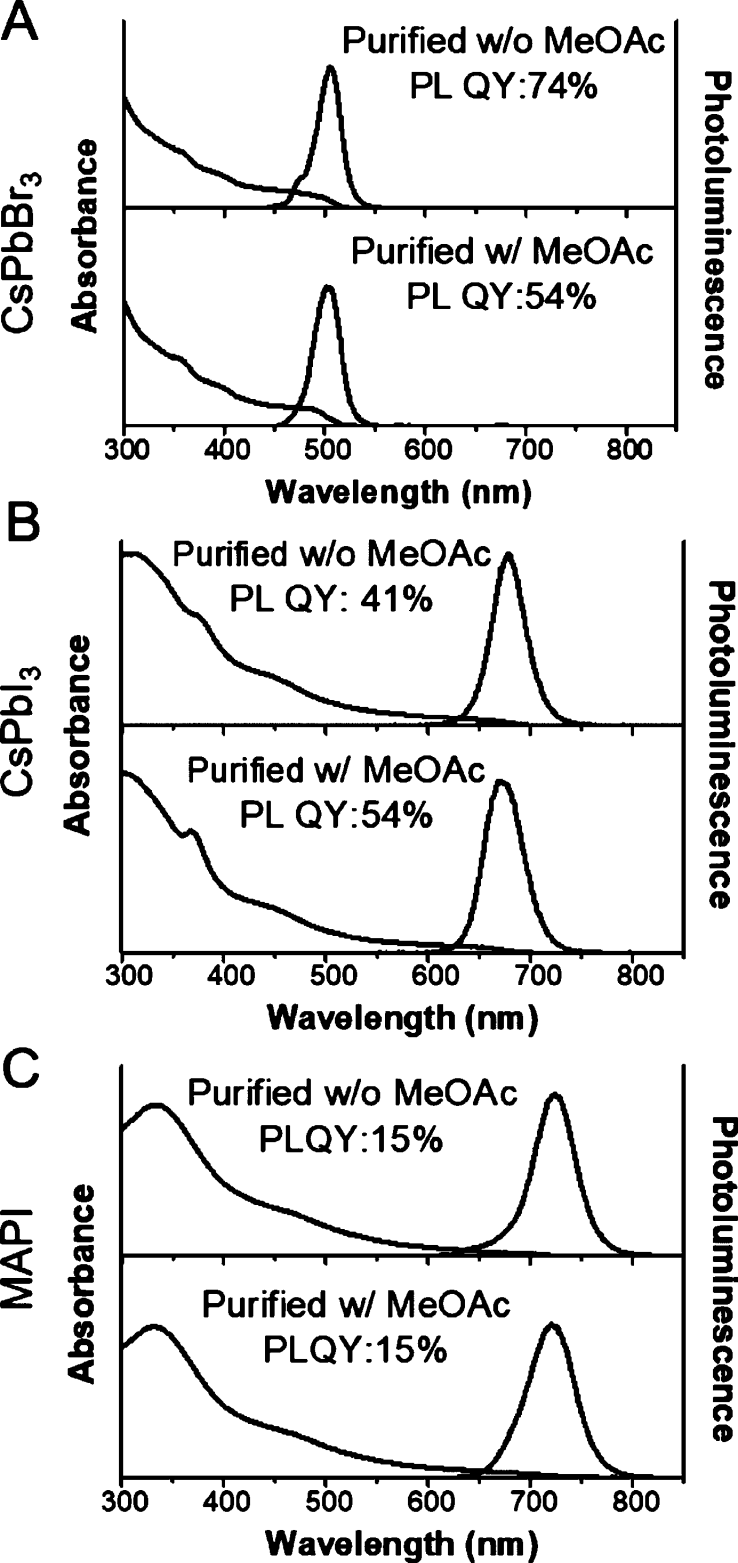
UV–vis absorbance and PL emission spectra of (A) CsPbBr_3_, (B) CsPbI_3_, and (C) MAPbI_3_ (MAPI) nanocubes in hexane that were isolated from crude reaction mixtures by centrifugation with or without the addition of methyl acetate (MeOAc). The nanocubes were isolated using an equal volume of MeOAc added to the crude reaction mixtures, followed by centrifugation at 8000 rpm (8228*g*) for 5 min. Poorly capped nanocubes were removed from the sample by dispersing the nanocubes in hexane and centrifuging again at 8500 rpm (9289*g*) for 5 min. The excitation wavelength was 350 nm for CsPbBr_3_ and 470 nm for CsPbI_3_ and MAPI nanocubes, and PLQYs were determined relative to Rhodamine-B. Adapted from ref (187). Copyright 2020 American Chemical Society. Further permissions related to the material excerpted should be directed to the ACS.

**Figure 12:**
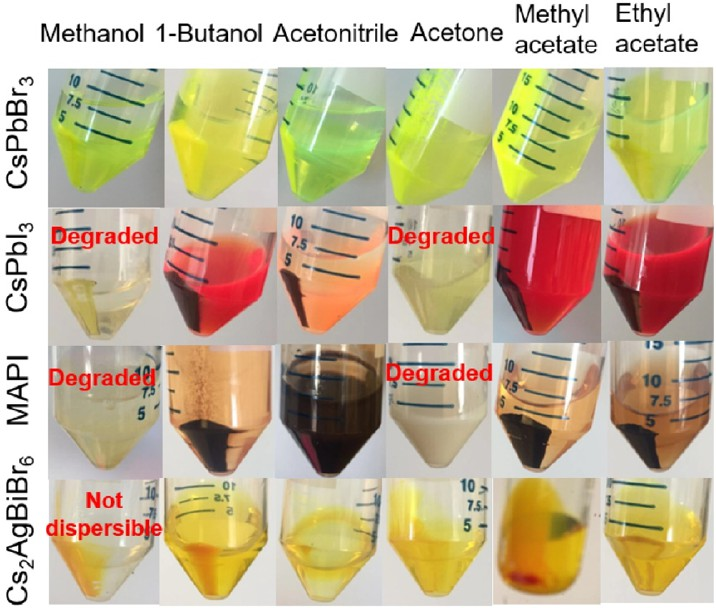
Photographs of centrifuge tubes with CsPbBr_3_, CsPbI_3_, MAPI, and Cs_2_AgBiBr_6_ (see NANOCRYSTALS OF LEAD-FREE PEROVSKITE-INSPIRED MATERIALS section for synthesis of Cs_2_AgBiBr_6_ nanocubes) nanocubes precipitated by centrifugation (8000 rpm (8228*g*) for 5 min) from crude reaction mixtures with six different polar solvents using equivalent volumes of polar solvent and crude reaction mixture. Nanocube concentrations were about 5–10 mg/mL. Some variation in nanocube concentration occurs because of the differences in reaction yields. Based on measured product yields, the concentrations were 4.3 mg/mL for Cs_2_AgBiBr_6_, 9 mg/mL for CsPbI_3_, and 7 mg/mL for CsPbBr_3_ and MAPI. Images are adapted from ref (187). Copyright 2020 American Chemical Society. Further permissions related to the material excerpted should be directed to the ACS.

With some care, a variety of polar antisolvents can be used to precipitate and purify metal-halide perovskite nanocubes without degradation.^184,185,190^ Methyl acetate has been widely used.^160,185,191−193^Figure 11 shows absorbance and PL spectra of CsPbBr_3_, CsPbI_3_, and MAPI nanocubes isolated from reaction mixtures by antisolvent precipitation with methyl acetate. The optical properties of these nanocubes are comparable to those of the nanocubes isolated without methyl acetate. Figure 12 shows images of CsPbBr_3_, CsPbI_3_, MAPI, and Cs_2_AgBiBr_6_ nanocubes that were precipitated with methanol, 1-butanol, acetonitrile, acetone, methyl acetate, and ethyl acetate. A clear and colorless supernatant indicates that all the nanocubes had been precipitated. There are a few situations where nanocubes are still retained in the supernatant, even with the use of the antisolvent. The expected colors of CsPbBr_3_, CsPbI_3_, MAPI, and Cs_2_AgBiBr_6_ nanocubes are yellow-green, dark red, dark brown, and golden-orange, respectively. Precipitation of CsPbI_3_ and MAPI nanocubes with methanol and acetone turned the color of the precipitate into pale yellow or milky white. Methanol and acetone are not compatible with CsPbI_3_ and MAPI nanocubes, and in general, these two polar solvents should be avoided when purifying iodide-containing nanocubes, including FAPbI_3_. Methanol and acetonitrile are not completely miscible with octadecene, and a liquid–liquid phase separation results that retains some nanocubes in the supernatant, which cannot be isolated. CsPbBr_3_ nanocubes are the most stable of these metal-halide perovskite NCs and were found to be compatible with all of the polar antisolvents shown in Figure 12. Cs_2_AgBiBr_6_ nanocubes are also relatively stable, although methanol does lead to irreversible aggregation and should be avoided.

In addition to the antisolvent chemistry, the conditions used to precipitate the nanocubes are important. Some of these conditions may seem trivial, like centrifugation time, for example.^184,185,194,195^ For CsPbI_3_ nanocubes, 5–10 min of centrifugation at 8000 rpm (8228*g*) works well. Longer centrifugation times can result in drastically different results, yielding CsPbI_3_ nanocubes with very poor dispersibility, low PLQYs, and nanocubes largely transformed to the yellow phase. The precipitate should be separated from the supernatant immediately after centrifugation. Degradation of the sample can continue to occur when the nanocubes remain in the presence of a large volume of polar solvent. The volume ratio of antisolvent to solvent is important. For example, when CsPbI_3_ nanocubes are dispersed in a crude reaction mixture of octadecene or redispersed in hexane at a concentration of about 10 mg/mL, an antisolvent to solvent volume ratio in the range of 1–2 is usually appropriate. This is not quite enough antisolvent to precipitate all of the nanocubes in the sample, but more antisolvent can end up degrading the nanocubes. An antisolvent/solvent ratio of 3, for example, will precipitate nearly all of the nanocubes, but the nanocubes will not be able to be redispersed easily and the PLQYs will be significantly reduced. Anhydrous solvents should be used to minimize degradation induced by water. Although not always necessary, the purification can be carried out in a glovebox under inert conditions. Using that procedure tends to provide nanocubes with longer shelf-life. There is a risk, however, that the sample starts degrading because the extra time spent transferring samples in and out of a glovebox prolongs the exposure of the nanocubes to antisolvent, which can induce such degradation. In general, the purification process should be optimized for each type of nanocube and the synthetic approach that is used. Differences in capping ligand chemistry and concentrations of the crude reaction mixture due to variations in the yields of alternative reactions can all lead to changes in the optimized antisolvent precipitation conditions.

**Figure 13:**
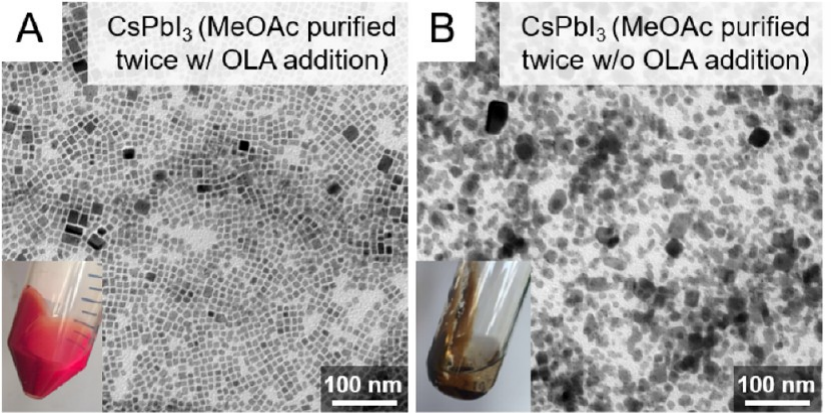
TEM images of CsPbI_3_ nanocubes that were precipitated twice with methyl acetate (A) with and (B) without the addition of oleylamine before the second precipitative washing step. The insets show photographs of the products obtained after centrifugation. The nanocubes in (A) were isolated after adding 10 μL of oleylamine to 3 mL of CsPbI_3_ nanocubes in hexane at a concentration of 10 mg/mL. Both samples in (A) and (B) were centrifuged at 8000 rpm (8228*g*) for 3 min after adding 3 mL of methyl acetate (1:1 v/v methyl acetate/hexane). Adapted from ref (187). Copyright 2020 American Chemical Society. Further permissions related to the material excerpted should be directed to the ACS.

The use of antisolvents to purify metal-halide perovskite nanocubes is essential in some cases. Analytical techniques, like NMR spectroscopy, require samples that are nearly completely free of unbound ligand and other organic impurities. One precipitative washing step is not enough to achieve the necessary level of purity required for these measurements. At least two cycles of precipitative washing are needed.^84^ A second precipitative washing step with antisolvent can degrade iodide-based metal-halide perovskite nanocubes such as CsPbI_3_. To prevent degradation, a small amount of excess ligand (*i.e.*, oleylamine) must be added before the second precipitative wash.^187^Figure 13 shows TEM images and photographs of CsPbI_3_ nanocubes after a second precipitation with methyl acetate. Without additional oleylamine, the CsPbI_3_ nanocube product ends up with a dull brown color, and the nanocubes are still mostly in the perovskite phase but have lost most of their luminescence and their distinct cubic shape. They do not redisperse in hexane. In contrast, the nanocubes in Figure 13A that were precipitated after an addition of oleylamine (10 μL) retain their luminescence and cubic shape and disperse readily in hexane. The NMR spectra of these nanocubes also do not show the presence of any free unbound ligand.^84,145,187^ For some nanocubes, however, the addition of oleylamine before a second precipitative wash can lead to degradation, as in the case of Cs_2_AgBiBr_6_ nanocubes.^196^ Each sample requires optimization of the best purification conditions, but in general, precipitation with polar antisolvents is an effective way to isolate and purify metal-halide perovskite nanocubes.

#### Summary and Outlook of Perovskite Nanocubes

A wide range of synthetic methods has been reported for monodisperse CsPbX_3_ (X = Cl, Br, and I) nanocubes with 80–100% PLQY (for X = Br and I) under optimized conditions. Every method has its own advantages and disadvantages. To date, HI and LARP methods have been extensively explored for the synthesis of inorganic perovskite NCs.^14,53^ In particular, HI synthesis is being heavily explored for shape-controlled synthesis of CsPbX_3_ NCs using different kinds of precursors and ligands. The role of acid–base equilibria of ligands, precursor types, and the chain length of amines in the shape control of CsPbBr_3_ nanocubes have been explored.^143,145,176,177^ In most synthesis methods, long-chain alkylamines have been used as ligands for stabilization of perovskite nanocubes. However, they are problematic for device applications as they block the transport of charge carriers. Therefore, it is important to explore short-chain ligands in future studies for the stabilization of perovskite nanocubes. Although perovskite nanocubes exhibit extremely high PLQYs right after synthesis, their purification leads to a significant reduction in PLQY (∼20–40%) due to the removal of ligands from the NC surface. To overcome this problem, bidentate ligands (or chelating ligands) have been proposed for enhanced stability and to retain high PLQY even after purification of nanocubes.^171,172^ While CsPbBr_3_ nanocubes have been found to be relatively stable over a long time, it is still challenging to obtain strongly luminescent, phase-stable CsPbI_3_ nanocubes. Various ligands have been proposed for improving their cubic phase stability; however, the stability is still not comparable to that of CsPbBr_3_ nanocubes. On the other hand, despite great progress in the synthesis of inorganic perovskite nanocubes, organic–inorganic hybrid nanocubes have been less explored regarding their shape-controlled synthesis, and future studies could be focused in this direction. In addition, more studies are needed in the future to obtain highly luminescent and stable lead-free perovskite nanocubes (see later sections on lead-free NCs).^197^

### Nanoplatelets

#### Origins of Perovskite Nanoplatelets

Two-dimensional (2D) metal-halide perovskite nanoplatelets trace their origin to the synthesis of Ruddlesden–Popper (R–P) phase-layered perovskite crystals. In the 1990s, it was discovered that substituting the usual small A-site cations (*e.g*., MA, FA, Cs) for larger organic cations (*e.g.*, butylammonium) could induce the crystallization of layered structures.^6,127,128,130,198−200^ These layered perovskite crystals consist of alternating inorganic layers of lead-halide octahedra and organic cations; the inorganic metal-halide layer primarily determines the optoelectronic properties, and the large organic cation layer electronically isolates the inorganic layers. Because of quantum-confinement effects, layered perovskites exhibit drastically different properties compared to the bulk 3D phase.^201^ Also, layered perovskites showed enhanced stability compared to 3D counterparts due to a negative enthalpy of formation^49,202,203^ as well as the presence of organic spacer layers that protect inorganic layers from external factors such as oxygen and moisture.^204^

**Figure 14:**
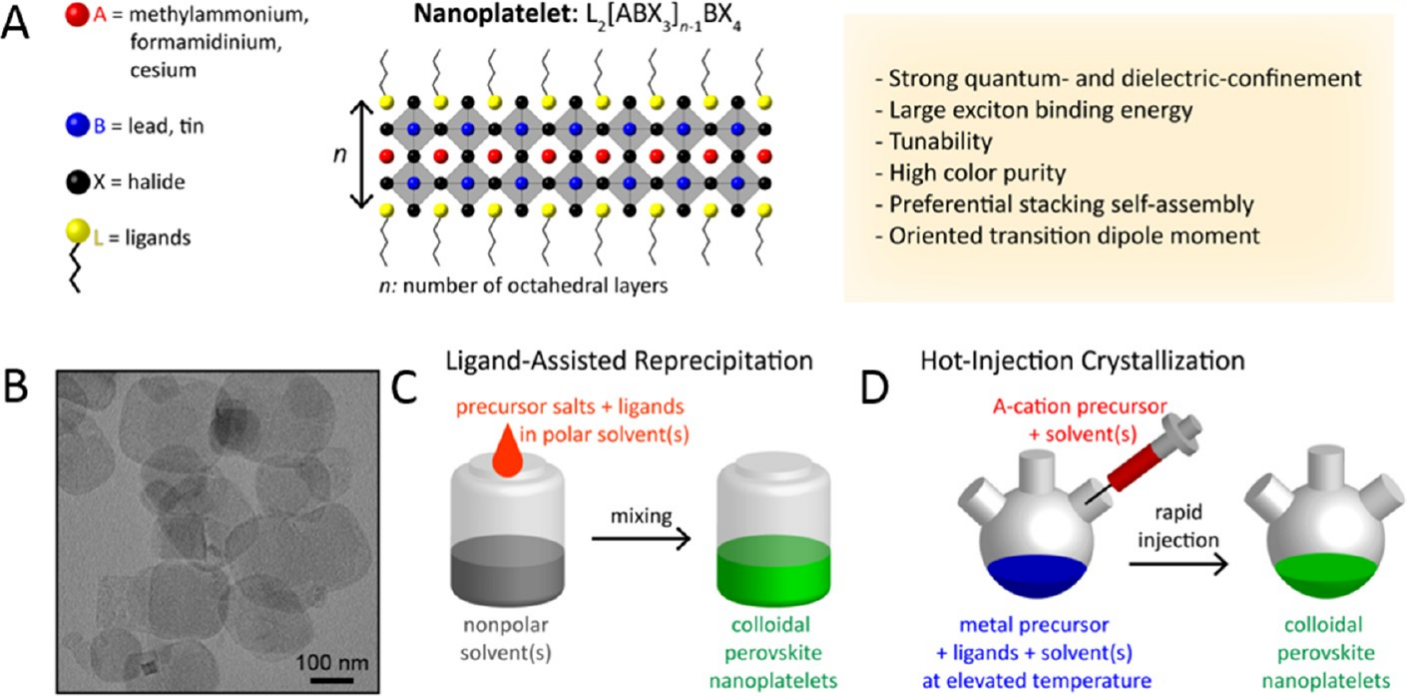
Structure of colloidal perovskite nanoplatelets and synthetic approaches. (A) Perovskite nanoplatelet structure and its distinctive properties. (B) Transmission electron microscopy image of nanoplatelets. Reproduced from ref (209). Copyright 2016 American Chemical Society. (C) Schematic illustration of ligand-assisted reprecipitation method. (D) Schematic illustration of hot-injection crystallization method.

Around 2015, multiple groups reported the synthesis of colloidal perovskite nanoplatelets^16,18,19,48^—2D perovskite crystals much like their R–P predecessors but dispersed in solution. Colloidal perovskite NPls are generally characterized by the chemical formula of L_2_[ABX_3_]_*n*−1_BX_4_ (Figure 14A,B) where *n* indicates the number of inorganic metal-halide octahedral layers in thickness. Thicknesses of NPls are confined to a few unit cells, and NPls can tolerate lateral dimension dispersity as long as thickness homogeneity is ensured.^47^ Surface ligands act as surfactants, entropically stabilizing the 2D crystals in solution, but their role in 2D NC formation is debated.^205,206^ Since layered R–P perovskites can be thought as a crystal of stacked NPls with electronically decoupled inorganic layers, there are many parallels between layered perovskites and perovskite NPls. They seem to be tunable over the same range and composition with identical band gap and optical properties,^18,47,201,207−209^ which implies that previous studies on layered perovskites can also shed light on the properties of colloidal perovskite NPls.

Colloidal perovskite NPls were initially identified as a side product of MAPbBr_3_ NC synthesis,^19^ but very quickly the ability to precisely control thickness was reported.^16,18,48,158^ Following these initial works, subsequent efforts focused on developing refined synthetic protocols for NPls with well-controlled thicknesses and improved material properties. For instance, the color of emission can be tuned by varying thickness and composition.^16,18,48,209−212^ Also, reports on the tunability of surface-capping ligands, ranging from short ligands for optimal charge transport behavior to long and functionalized ligands for enhanced stability, have highlighted the possibility of optimizing surface properties of NPls for specific applications.^212−214^ It has also been reported that the lateral dimension of NPls, which may affect electronic transport in NPl optoelectronic devices, can be tuned from tens of nanometers^18,48,54,158,210,215,216^ to several micrometers^47,209,217,218^ without loss of quantum confinement in the vertical direction.

#### Distinctive Properties of Nanoplatelets

2D nanoplatelets possess distinctive characteristics specific to their 2D shape (Figure 14A). The exciton Bohr radius of lead-halide perovskite materials has been reported to be ∼3 nm or larger, depending on composition.^14,16,47,48,128^ It is synthetically challenging to prepare 0D NCs with such small dimensions; however, perovskite NPls as thin as 0.6 nm in thickness^209,214,219−221^ exhibiting strong quantum and dielectric confinement can be easily fabricated. This strong confinement induces excitonic absorption and emission features to be blue-shifted from those of the bulk perovskite phase by up to 0.7 eV,^47,222^ which enables the synthesis of bluer light-emitting NCs. Spatial confinement of excitons in 2D structures also yields large exciton binding energies, reaching up to several hundred meV,^100,128,130,222^ which can facilitate efficient recombination of excitons. Moreover, monodisperse NPls exhibit superior emission color purity due to atomically precise thickness homogeneity. Achieving monodispersity is of great importance for NPls since band gaps of strongly confined NPls show significantly larger shifts when the thickness changes,^47,209,211^ compared to other weakly confined NCs.^14,223,224^ Nonetheless, monodisperse nanoplatelets have been widely demonstrated.^18,48,60,158,209,210,213,217,221,225,226,54,218^

A 2D structure is ideal for integration into optoelectronic devices. A key feature of 2D NPls is the tendency for the transition dipole moment to preferentially orient within the 2D plane,^227,228^ which is advantageous for optical coupling. Additionally, NPls exhibit face to face stacking^18,54,224^ and preferential face-down assembly on a given substrate.^209,214,218,229^ This tendency—combined with transition dipole anisotropy—leads to preferential emission in the out-of-plane direction.^228^ Moreover, large lateral dimensions of NPls^16,209,217,218^ can potentially be utilized to minimize grain boundaries in-plane and lower the percolation threshold for charge transport.^230^

#### Synthesis of Nanoplatelets

**Figure 15:**
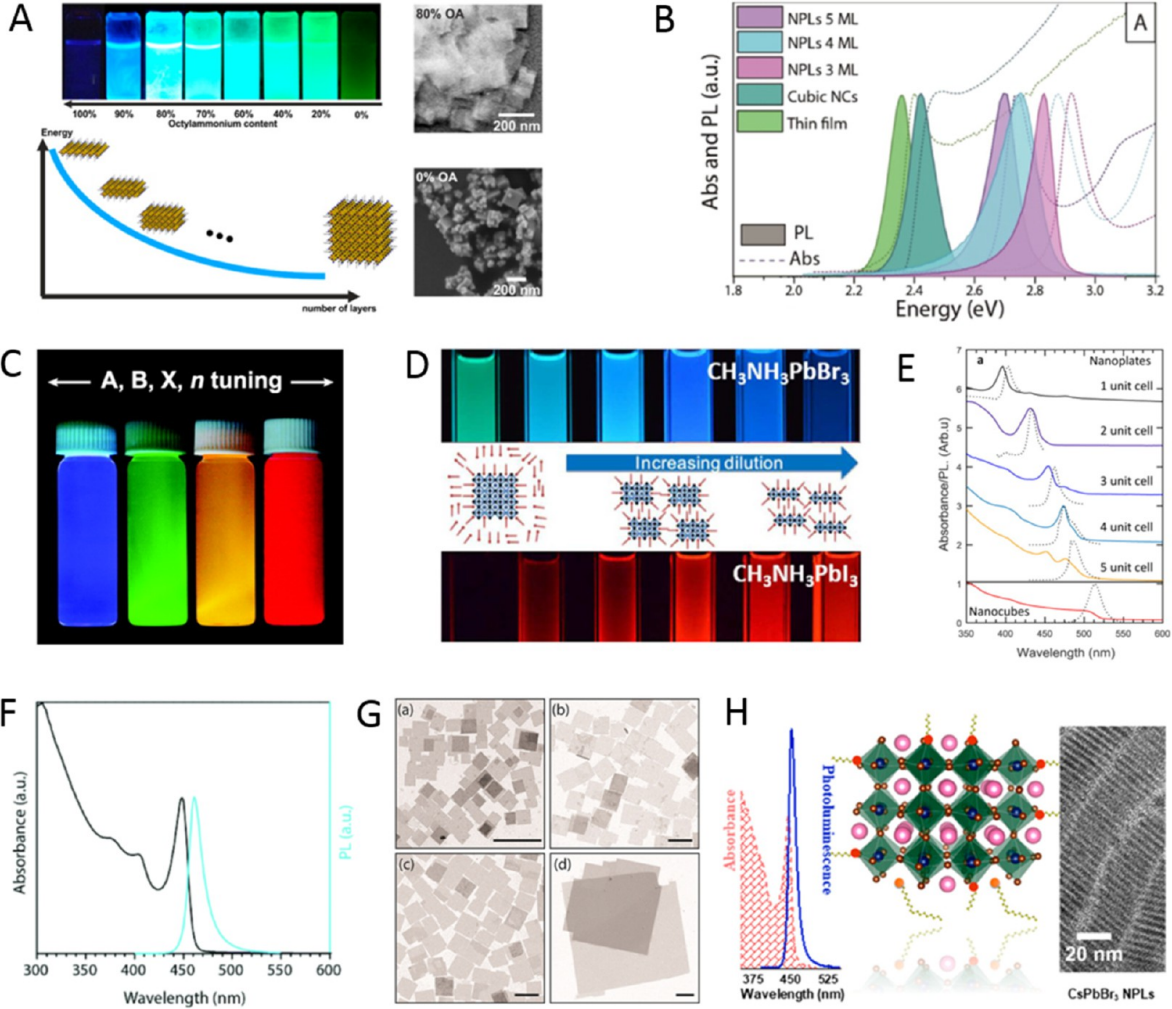
Advancements of colloidal perovskite nanoplatelet synthesis. (A) Synthesis of thickness-controlled MAPbBr_3_ nanoplatelets *via* LARP. (B) Synthesis of CsPbBr_3_ nanoplatelets *via* LARP. (C) Thickness and compositional tunability of nanoplatelets *via* LARP. (D) Dilution-induced nanoplatelet formation *via* LARP. (E) Thickness-controlled CsPbBr_3_ nanoplatelet synthesis *via* HI. (F) *n* = 3 MAPbBr_3_ nanoplatelet synthesis *via* HI. (G) Nanoplatelet lateral dimension control through HI synthesis. (H) Synthesis of hexylphosphonate-capped NPls with enhanced stability *via* heat-up approach. Panel A is reproduced from ref (16). Copyright 2015 American Chemical Society. Panel B is reproduced from ref (48). Copyright 2016 American Chemical Society. Panel C is reproduced from ref (209). Copyright 2016 American Chemical Society. Panel D is reproduced from ref (231). Copyright 2016 American Chemical Society. Panel E is reproduced with permission from ref (18). Copyright 2015 American Chemical Society. Panel F is reproduced under a Creative Common [CC-BY 3.0] license with permission from ref (158). Copyright 2016 Royal Society of Chemistry. Panel G is reproduced from ref (217). Copyright 2016 American Chemical Society. Panel H is reproduced under a Creative Common [CC-BY] license from ref (213). Copyright 2020 American Chemical Society.

Numerous synthetic approaches to perovskite NPls have been developed. In this section, we start with a discussion on the two most widely used techniques—LARP (Figure 14C) and hot-injection crystallization (Figure 14D)—and then introduce other synthetic approaches. The LARP method usually consists of dissolving perovskite NPl precursor salts in relatively polar solvent(s) (*e.g*., DMF and DMSO) and then mixing it with less polar solvent(s) (*e.g*., toluene, hexane) to induce crystallization at room temperature. In 2015, Sichert *et al*. reported the synthesis of thickness-controlled MAPbBr_3_ NPls *via* LARP (Figure 15A).^16^ They first dissolved NPl precursors (MABr, PbBr_2_, and OABr) in DMF, and NPs were then crystallized upon mixing the solution with excess toluene. Precise tuning of NPl thickness was achieved by varying the methylammonium to octylammonium ratio in the precursor solution. Soon after, Akkerman *et al*. reported the synthesis of *n* = 3–5 CsPbBr_3_ NPls with modified LARP process where the addition of acetone into the precursor solution mixture induced destabilization of precursor complexes and initiated NPl crystallization under ambient conditions (Figure 15B).^48^ They also showed that the band gap of the NPls can be continuously tuned by an anion exchange reaction. Later, Weidman *et al.* published *n* = 1 and *n* = 2 perovskite NPls with wide ranging composition (A = MA/FA/Cs, B = Pb/Sn, X = Cl/Br/I, ligand = butylammonium/octylammonium) *via* LARP by simply varying the stoichiometric ratios of precursor solutions (Figure 15C).^209^ Tong *et al.* demonstrated the breakup of large MAPbX_3_ NCs synthesized *via* LARP into NPls by diluting the solution, which triggered osmotic swelling by solvent (Figure 15D).^231^ In addition, Sun *et al.* carried out a systematic study and showed that choosing the right combination of ligand species plays a crucial role in determining the shape of the NCs synthesized *via* LARP.^176^

In general, LARP enables facile synthesis of colloidal perovskite NPls with easily tunable composition and ligands. Moreover, LARP can be highly cost-effective as it delivers colloidal perovskite NPls in ambient atmosphere at room temperature. However, thinner NPls synthesized *via* LARP tend to exhibit lower photoluminescence quantum yield,^16,209,214^ and it is difficult to target thicker (*n* ≥ 3) dispersions with good thickness control.^214,232,233^ Recent works have focused on refining the synthesis and improving material properties—expanding synthetic capability,^60,212,220^ improving thickness selectivity,^214,220^ modulating surface properties by incorporating different ligand species,^212,214^ boosting photoluminescence quantum yield^60,211,225^ and enhancing material stability.^229^ Although significant advancements have been made in the past few years, there is still ample room for further development.

Another widely used synthetic approach is hot-injection crystallization, as described in the previous section. The HI approach is based on the rapid injection of a precursor solution into a solution containing the other precursors, ligands and solvent(s), at elevated temperature. The HI synthesis enables the separation of nucleation and growth of NCs so that it can deliver high-quality NCs.^52^ Also, it does not involve any polar solvent which could potentially be detrimental to colloidal perovskites. Early reports of perovskite NPl synthesis *via* the HI protocol^18,158^ came out a few months after Protesescu *et al.* published the synthesis of CsPbX_3_ quantum dots *via* HI.^14^ Bekenstein *et al.* found that lowering the temperature of cesium precursor injection into lead-halide precursor solution results in the formation of *n* = 1–5 CsPbBr_3_ NPls (Figure 15E).^18^ They also demonstrated NPl band gap tuning *via* halide exchange reaction. Around the same time, Vybornyi *et al.* reported the HI synthesis of *n* = 3 MAPbBr_3_ NPls (Figure 15F).^158^ Along with the previous report from Sichert *et al.* on the synthesis of MAPbBr_3_ NPls *via* LARP,^16^ those early reports revealed the possibility to synthesize perovskite NPls with control over their thickness. However, it was pointed out that lateral dimensions of perovskite NPls synthesized *via* HI (10–100 nm)^18,158,210^ are generally smaller than those of NPls synthesized *via* LARP (100–1000 nm).^16,209,214^ In response to it, Shamsi *et al.* showed that the lateral dimension of CsPbBr_3_ NPls can be increased to several microns by adjusting the ratio of shorter ligands to longer ligands in the synthetic mixture during the HI synthesis (Figure 15G).^217^ Similarly, Zhang *et al.* published the synthesis of micron-sized *n* = 2 FAPbBr_3_ NPls.^218^ Furthermore, Pan *et al.* provided deeper insight into HI synthesis by identifying the key factors that control the shape of the NCs in HI synthesis—reaction temperature and choice of ligands.^177^

Recent works on NPl synthesis *via* HI have focused on refining the synthesis of NPls accompanied by detailed structural characterizations^210^ and understanding the complex dynamics of the HI reaction.^145,215^ However, the HI synthesis is still highly focused on Cs-based NPls,^18,145,177,210,215,217^ and there is only a limited number of reports on organic cation-based NPls.^158,218^ Compared to LARP-synthesized NPls, HI-synthesized NPls are generally smaller in lateral dimensions^18,158,210^ and usually capped by longer ligands,^16,18,209,210^ which could undermine electronic transport properties. Since the HI method requires high temperature and inert atmosphere, scalability and cost-effectiveness could be greater barriers to eventual commercialization for HI than for LARP. Historically, HI-synthesized NPls have shown higher PLQY,^18,48^ though the PLQY of LARP-synthesized NPls has recently become comparable.^60,211,225,231^ Thus, more efforts on further developing HI synthesis of perovskite NPls are needed.

Apart from LARP and HI, other creative approaches to perovskite NPL synthesis have been demonstrated. Shamsi *et al.* showed that quantum-confined CsPbBr_3_ NPls can be synthesized by mixing of cesium-oleate solution with PbBr_2_–ligand complex solution, adding isopropyl alcohol to initiate nucleation and then heating the solution to grow NPls.^234^ A few years later, Shamsi *et al.* slightly modified this heat-up method and demonstrated the synthesis of hexylphosphonate-capped NPls (Figure 15H).^213^ They observed that stronger binding of phosphonate ions compared to conventional alkylammonium ions to NPl surface^177,213^ greatly improved the stability of NPls and suppressed transformation of NPls into thicker, less-confined structures which can result in the loss of desirable optical properties.^229,234,235^ Huang *et al.* reported the scalable synthesis of *n* = 4 FAPbI_3_ NPls by mixing the FA–ligand complex solution with PbX_2_–ligand complex solution in toluene.^54^ This approach was a hybrid of HI and LARP in that it was done under ambient conditions at room temperature but no polar solvent was involved. Another interesting approach is ultrasonication-assisted synthesis: Tong *et al.*(30) and Hintermayr *et al.*(132) reported the synthesis of perovskite NPls by sonicating the dispersion of perovskite precursors in the presence of coordinating ligands. Lastly, Dou *et al.* demonstrated the direct synthesis of atomically thin monolayer of L_2_BX_4_ perovskite on the substrate by drop-casting the solution of precursor salts first dissolved in DMF/chlorobenzene cosolvent.^17^ Even though this was not a “colloidal nanoplatelet” synthesis, it introduces another promising route to deposit a thin layer of 2D perovskites.

#### Outstanding Questions and Future Challenges for Nanoplatelets

Although various synthetic techniques have been developed for colloidal perovskite NPls, a complete understanding of anisotropic perovskite NPl growth is lacking. How can thin 2D structures grow from an isotropic crystal lattice and homogeneous solvent environment? An in-depth study carried out by Riedinger *et al.* on the formation of 2D CdSe NPls from isotropic materials^205^ provides some interesting insight. In that paper, the authors started with experimentally verifying that CdSe NPls can be formed in an isotropic environment in the absence of any molecular mesophases, and then formulated a growth model based on experimental results. General theory of nucleation and growth predicts the growth of a NC to occur through the nucleation of a additional island on one of the facets; when this island reaches a critical size, expansion of the island becomes thermodynamically favorable and leads to the formation of a complete additional layer on that facet. Riedinger *et al.* showed that when specific criteria are met, namely, (1) NC formation occurs through nucleation-limited growth, (2) initial small crystallites can adopt anisotropic 2D shapes due to the random fluctuations in the reaction mixture, and (3) the thickness of this initial crystallite is smaller than the critical island size—certain combinations of volume, surface, and edge formation energies of NCs in the system can lead to a lower nucleation barrier for narrower facets compared to large planar facets. This lower nucleation barrier results in the faster growth on the narrower facet, which can eventually yield anisotropic 2D NPls. Their model also predicts higher narrow-facet nucleation barrier for thicker NPls than thinner NPls, and it is consistent with the observations by Bekenstein *et al.*(18) and Pan *et al.*(177) that thicker perovskite NPls were formed at higher reaction temperatures. Although Riedinger *et al.*(205) studied the CdSe NPl system, their theoretical model is generalizable to any isotropic materials system, including perovskite NPls. It should also be noted that, along with reaction temperature, previous reports listed a careful choice of ligands and precise control of perovskite precursor composition and concentration of precursor solution as other key factors in the shape-controlled synthesis of perovskite NPls.^145,177,236^ We speculate that optimized synthetic conditions in those reports may in fact reflect precisely tuned volume, surface, and edge formation energies of the NC in the system where the formation of anisotropic 2D NPls is favored. More recently, Burlakov *et al.* proposed a CsPbBr_3_ NPl formation mechanism based on the competitive nucleation of an inorganic perovskite layer and an organic ligand layer.^206^ Being consistent with the discussion above, their work also focused on temperature and interaction energies between constituents as primary factors that determine nucleation kinetics. Through a combination of theoretical and experimental work, they showed that, under certain conditions, narrower facets can favor crystal layer nucleation, while wider facets are more effectively passivated by ligand layer formation, which can lead to anisotropic two-dimensional crystal growth. Their theoretical prediction of preferential formation of thinner NPls at low reaction temperature was experimentally verified and is also consistent with the observations by Bekenstein *et al.*(18) and Pan *et al.*(177) Still, this picture is far from complete, and we do not yet have a firm grasp on the mechanism of how anisotropic NPls are formed from isotropic environments.

In addition to open questions regarding nucleation and growth, a detailed understanding of the electronic structure in 2D NPls is still lacking. Furthermore, it is unclear to what extent perovskite NPls actually exist as isolated sheets in solution rather than small crystallites of RP phase.^237^ Spontaneous stacking^158,224^ and slow precipitation of NPls^214^ in concentrated solutions have been observed, which may indicate the existence of large RP-phase crystallites with poor colloidal stability. Thus, a systematic study on the behavior of NPls in colloidal solution is needed for a better solution processability. In addition, efforts are underway to tackle the main drawbacks of perovskite NPls, namely, improving their low PLQY^60,225^ and enhancing the stability.^229^ Additional goals include the synthesis of stable lead-free NPls,^209^ doping NPls to expand their functionality,^238^ and integrating NPls into state-of-the-art optoelectronic devices (see also later sections on these various topics).^211^

### Nanowires

Semiconductor nanowires are fundamental nanoscale building blocks for nanophotonic platforms such as interconnects, waveguides, and optical cavities. Due to the single-crystallinity and well-controlled interfacial engineering, individual NWs or their assemblies are also ideal model systems for the fundamental study of charge transfer and carrier dynamics at the nanoscale. Metal-halide perovskites have demonstrated a significant level of defect tolerance. The ionic nature of halide perovskites makes them interesting systems to understand charge dynamics in defect tolerant materials compared with covalent inorganic semiconductors. In addition, low-temperature synthesis and facile ion exchange chemistry provide additional possibilities for understanding alloy and heterostructure formation to explore nanoscale properties. In this section, we review the synthetic approaches of inorganic halide perovskite NWs, their self-assembly, anion exchange, phase transition, and their various applications, especially in photonics and thermoelectrics.

#### Synthesis of Inorganic Perovskite Nanowires

##### Colloidal Synthesis

**Figure 16:**
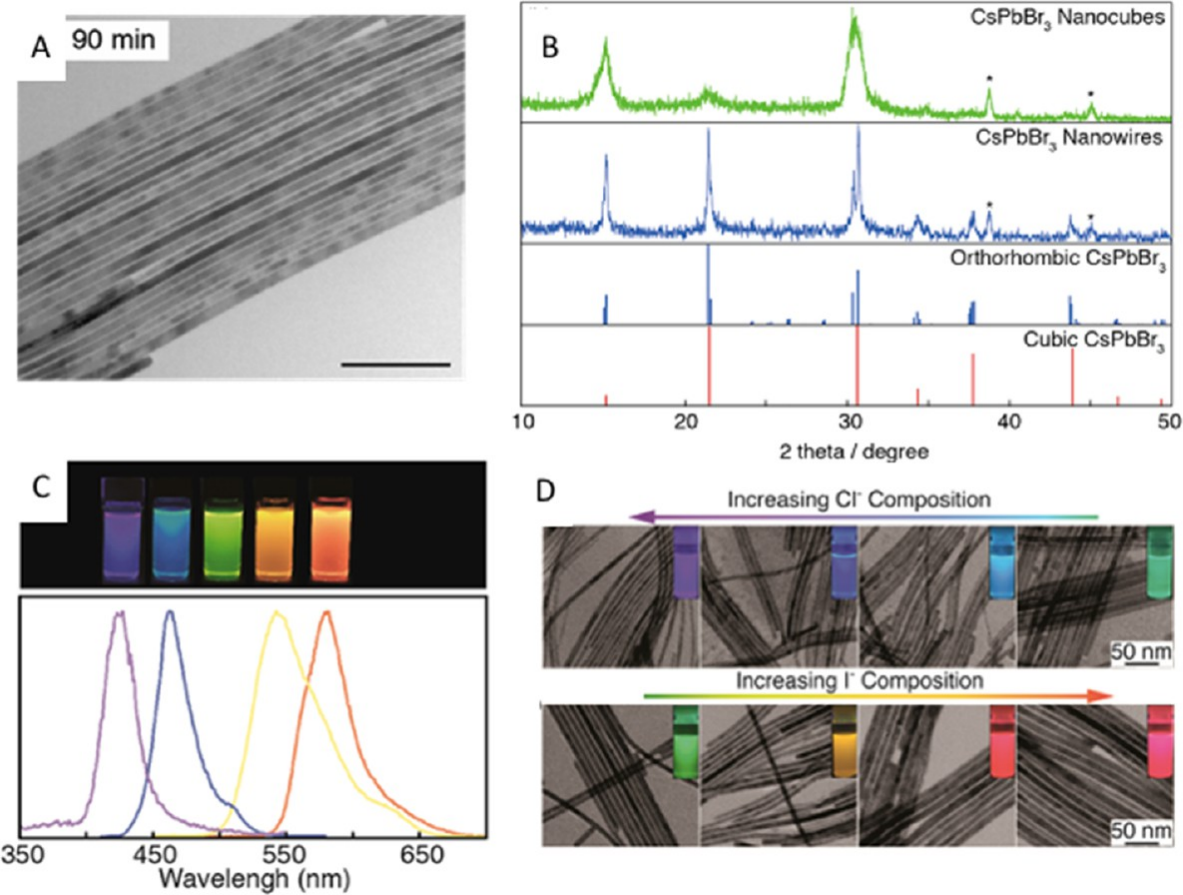
(A) TEM image of colloidally synthesized CsPbBr_3_ nanowires. (B) X-ray diffraction spectrum of CsPbBr_3_ nanocubes and nanowires. Reproduced from ref (75). Copyright 2015 American Chemical Society. (C) Photoluminescence properties of ultrathin CsPbBr_3_ nanowires with different halide compositions. Reproduced from ref (76). Copyright 2016 American Chemical Society. (D) TEM images of anion exchange in CsPbX_3_ perovskite nanowires with various halide contents. Reproduced from ref (56). Copyright 2016 American Chemical Society.

One-dimensional (1D) perovskite NWs have attracted attention because of their large morphology anisotropy and quantum mechanical effects associated with the two confined dimensions. Shortly after the successful synthesis of perovskite NCs,^14^ halide perovskite NWs were synthesized by controlling the reaction conditions to achieve different aspect ratios, chemical compositions and phases. In the synthesis of NWs, the formation of “isotropic” perovskite NCs typically dominates in the early stage of reaction, which is triggered by the rapid injection of cesium precursor (Cs-oleate) into a hot solution of lead precursor (Pb-halide) with the proper choice of organic ligands such as oleic acid, oleylamine, and octylamine.^74,75^ In 2015, Zhang *et al.*(75) reported the solution-phase colloidal synthesis of CsPbBr_3_ perovskite NWs that exhibit orthorhombic crystal structure (Figure 16A,B). Later, it was found that NWs evolve through a linear growth, their aspect ratio quickly increases over time and the NW lengths up to 5 μm are easily reached.^239^ Inspired by this approach, Tong *et al.*(22) reported the synthesis of CsPbBr_3_ NWs by ultrasonication of precursor powders and ligands. They found that, different from a linear growth mechanism in the hot inject synthesis, the initially formed nanocubes gradually transform into NWs through the oriented attachment mechanism. These methods seem to work quite well for CsPbBr_3_ NWs. However, the growth of CsPbI_3_ NWs was found to be characterized by much faster kinetics and less controllable size and phase: although the cubic phase of CsPbI_3_ can be stabilized at high temperature (above 360 °C), especially at the nanoscale, it spontaneously transforms into the room-temperature stable orthorhombic phase characterized by 1D chains of edge-sharing octahedra. A recent study suggested that, at the initial growth stage of orthorhombic CsPbI_3_ NWs, the cubic phase CsPbI_3_ nanocubes show lattice distortion induced by the polar solvent molecules, which triggers hierarchical self-assembly of CsPbI_3_ nanocubes into single-crystalline NWs through an orientated attachment process.^186^ This distinct crystal structure of the CsPbI_3_ NWs leads to their distinctive optical behaviors at room temperature. Unlike the narrow and strong excitonic emission from CsPbBr_3_ NWs, the CsPbI_3_ NWs show a broad and low-energy emission that is attributed to the indirect band gap transition of the orthorhombic phase.^240^

Ultrathin perovskite NWs with a diameter less than the exciton Bohr radius down to atomic level (<3 nm) are additionally interesting due to their potential quantum-confinement effects.^76^ Zhang *et al.* developed a method to improve both purity and yields of ultrathin NWs from colloidal synthesis.^76^ The ultrathin CsPbBr_3_ NWs showed a strong photoluminescence at ∼465 nm, which is significantly blue-shifted compared to the emission wavelength for bulk CsPbBr_3_ (∼530 nm) (Figure 16C,D). A surface treatment with PbBr_2_ precursor led to an increase in both PLQY and stability of the NWs by retarding the ripening process. Similarly Imran *et al.* developed a method to grow CsPbBr_3_ NWs with a width that could be tuned down to the quantum-confinement regime (3.4 ± 0.5 nm), using short carboxylic acids and long alkylamines as the growth medium.^74^ From their study, the increased concentration of short carboxylic acid over the long ligand led to a reduction in the NW width.

To achieve the composition tunability in colloidally synthesized halide perovskites, the facile anion exchange process has been applied to perovskites with different morphologies and is discussed extensively in the Composition Control by Ion Exchange and Suppression of Exchange section of this review.^55,57^ Halide anion exchange chemistry in CsPbX_3_ NWs represents a powerful strategy for attaining band gap tunability across the blue to near-IR wavelength region.^56^ Post-synthetic chemical transformations have been used in halide perovskites to obtain broad compositional tunability. CsPbBr_3_ NWs were used as the starting materials, and the CsPbX_3_ alloy NWs with a wide range of halide compositions can be achieved through anion-exchange reactions using organic or inorganic halide precursors. The anion-exchange reaction in perovskite NCs typically happens at the nanocrystal–solvent interface and at room temperature. The PL of CsPbX_3_ NWs is easily tunable across the entire visible range by varying the halide composition in a similar way to CsPbX_3_ nanocubes.

##### Solvent-Evaporation-Induced Nanowire Growth

**Figure 17:**
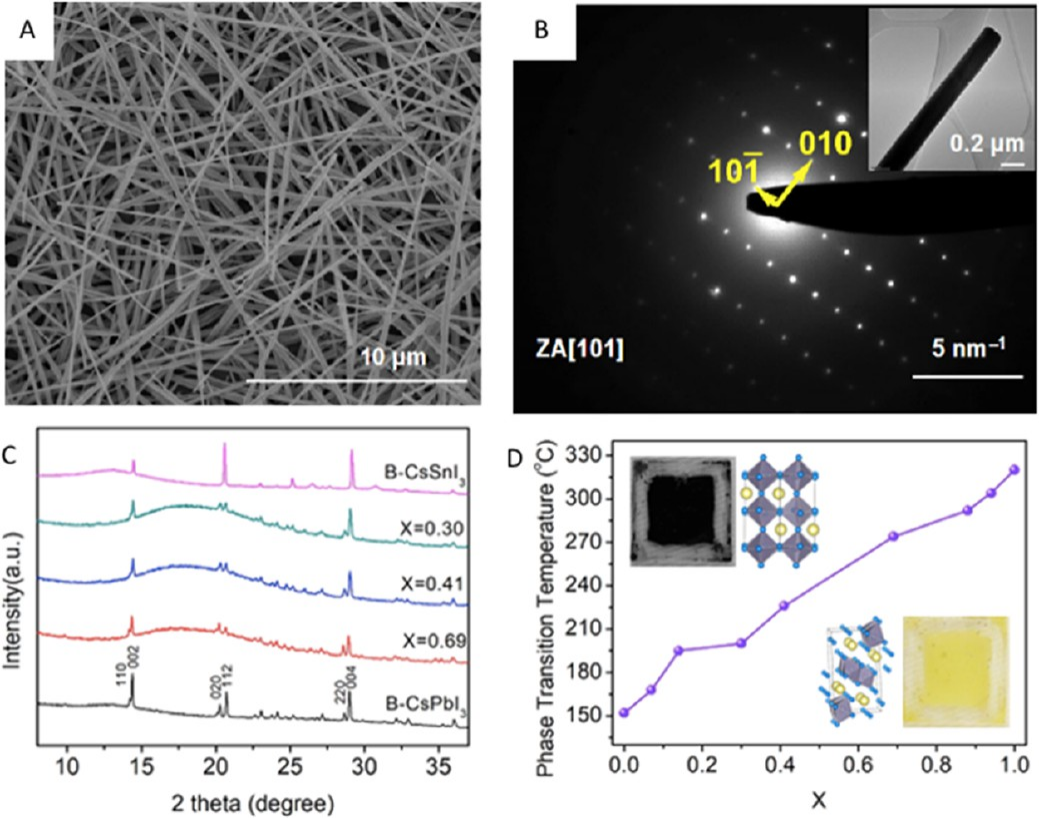
(A) SEM images of CsPbI_3_ nanowires grown on a glass substrate from solvent evaporation method and (B) Selected area electron diffraction pattern of a single nanowire to confirm the single-crystalline orthorhombic CsPbI_3_ phase. Reproduced with permission from ref (240). Copyright 2017 Springer Nature. (C) X-ray diffraction patterns of black phase CsPb_*x*_Sn_1–*x*_I_3_ nanowire mesh. (D) Phase transition temperature of CsPb_*x*_Sn_1–*x*_I_3_ nanowires as a function of Pb content in alloy composition. Reproduced with permission from ref (243). Copyright 2018 American Chemical Society.

In addition to the inorganic perovskite NW synthesis using colloidal methods, single-crystalline micrometer-sized perovskite NWs can be synthesized using the surfactant-free, substrate-assisted dissolution–recrystallization growth method.^240−242^ Here, the polycrystalline thin film of PbX_2_ acts as the seed to initiate the perovskite NW growth by immersing it into a diluted cesium-halide precursor solution. The lead precursor slowly dissolves and recrystallizes with the surrounding cesium precursor to form one-dimensional perovskite single crystals (Figure 17A,B). The appropriate balance between the choice of high halide salt solubility and low perovskite solubility is the key to achieve effective transformation of perovskite NWs from the seeding layer. This method has been applied to perovskites with different phases and compositions.^242^ For example, and as already state earlier, the CsPbI_3_ system can adopt either the non-perovskite yellow phase (double chain orthorhombic structure) or the black perovskite phase through the rapid thermal quenching process.^240^ The synthesis of single-crystalline perovskite alloys with mixed “B”-site cation has been challenging due to the thermodynamically favorable phase separation in solution. Lei *et al.* successfully synthesized single-crystalline CsPb_*x*_Sn_1–*x*_I_3_ NWs (Figure 17C) with the substrate-based solvent evaporation method.^243^ In particular, the yellow phase and the black phase CsPb_*x*_Sn_1–*x*_I_3_ NWs can easily be interconverted by carefully tuning of the quenching temperature. The transition temperature increases from 152 to 320 °C as the Pb concentration increases in CsPb_*x*_Sn_1–*x*_I_3_ NWs (Figure 17D). The electrical conductivity of direct band gap black phase CsPb_*x*_Sn_1–*x*_I_3_ is 3–4 orders of magnitude higher than that of the yellow phase CsPb_*x*_Sn_1–*x*_I_3_ NWs. In addition to the mixed “B”-site cation perovskites, mixed alloyed NWs can also be prepared by adjusting the ratios of halides (I, Br, Cl) or A-site cations (MA, FA, Cs).^244,245^

##### Vapor-Phase Transport and Growth

**Figure 18:**
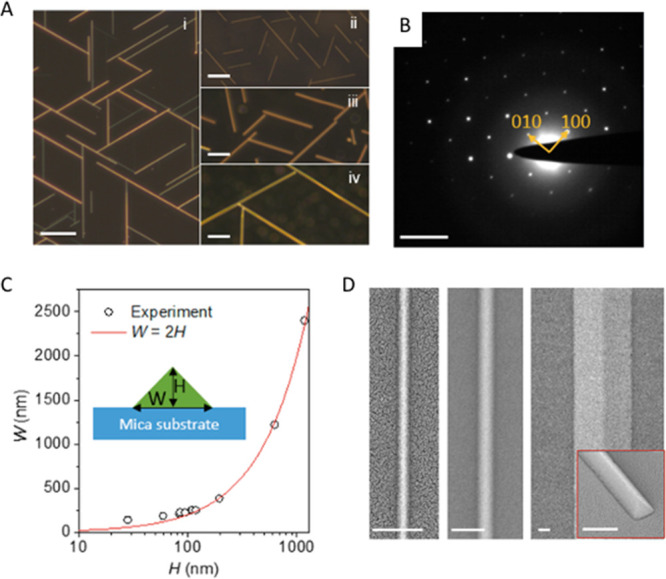
(A) Optical dark-field images of CsPbBr_3_ nanowires grown on the mica substrate from chemical vapor transport method and (B) selected area electron diffraction patterns from CsPbBr_3_ nanowires with a high single crystallinity. (C) Mapping of nanowire geometry on the mica substrate for the width and height. (D) SEM images of individual CsPbBr_3_ nanowire with different lateral widths from left to right (scale bar, 500 nm). Reproduced from ref (249). Copyright 2018 American Chemical Society

For hybrid organic–inorganic perovskites, direct vapor-phase growth is challenging due to the decomposition of the organic cation from the perovskite before vaporization. However, this is not a problem for all-inorganic CsPbX_3_ perovskite systems and they can be easily obtained at ∼450 °C. By precise control of reactant transport and epitaxial substrate selection (mica, sapphire *etc.*), the perovskite NWs can achieve controlled alignment and orientation with tunable compositions.^246−249^ For example, the CsPbBr_3_ NWs can be grown such that they are horizontally aligned on the mica substrate, and the size distribution spans from less than 200 nm to a few microns (Figure 18).^249^ With the same synthetic approach, the growth of perovskite NWs can be controlled in the in-plane direction by the graphoepitaxial effect on sapphire substrate.^250^ A comparative study of epitaxial and graphoepitaxial growth has been conducted with CsPbBr_3_ NWs.^251^ The graphoepitaxial growth of CsPbBr_3_ NWs results in the bidirectional growth and horizontal alignment on a faceted sapphire substrate. The CsPbBr_3_ NWs grown epitaxially on the flat sapphire plane show six isoperiodic directions. Such facile synthesis and controllability of large-scale nanowire networks could potentially facilitate their integration in electronic devices. These single crystals are highly photoluminescent with tunable emission wavelengths, making it possible to observe phase transitions and physical property evolution through an optical approach. Vapor-phase grown single-crystal perovskites can provide an excellent platform for fundamental understanding of the lattice dynamics and transport properties, considering their high crystalline quality, low defect density, and controllable morphologies.

#### Anion Exchange and Phase Transition in Perovskite Nanowires

Compared to many of traditional covalent semiconductors, the soft nature of the crystal lattice and the weak ionic bonding enable higher reconfigurability in halide perovskites. Consequently, a significant ionic migration is expected in the halide perovskite lattice, which is considered as a possible origin for anomalous hysteresis, light-induced phase segregation and photoinstability. A fundamental understanding of the ionic behavior in halide perovskites has been primarily based on conventional charge transport studies, which only revealed long-range diffusion on average at the macroscopic level. By combining anion exchange chemistry with nanofabrication techniques, single-crystalline halide perovskite NW heterostructures have been synthesized.^252,253^ The spatially resolved multicolor CsPbX_3_ (X = I, Br, Cl or alloy of two halides) NWs show a sharp electronic interface of the heterojunctions, which enables a quantitative study of ion interdiffusion and migration dynamics. Unlike the single-crystalline nanostructured perovskite, ionic migrations/diffusions across the grain boundary in polycrystalline thin film are usually faster than inside the lattice.^15,254^ Thus, the high ionic conductivity from polycrystalline thin films may not truly represents the intrinsic properties.

**Figure 19:**
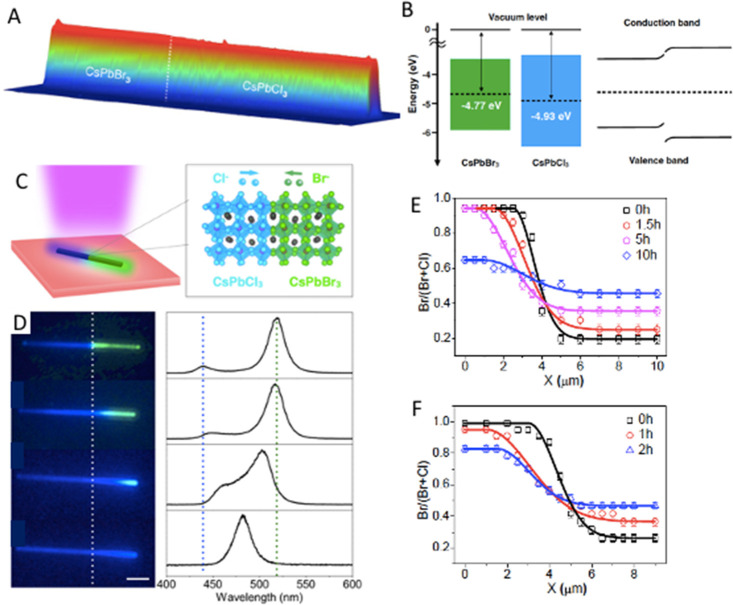
(A) Three-dimensional atomic force microscopy image of CsPbCl_3_–CsPbBr_3_ nanowire heterostructure. (B) Corresponding electronic work functions determined by Kelvin probe force microscopy and the electronic band alignment of CsPbBr_3_–CsPbCl_3_ nanowire. Reproduced with permission from ref (252). Copyright 2017 National Academy of Sciences of the United States of America. (C) Schematic illustration of perovskite nanowire heterostructure of CsPbBr_3_–CsPbCl_3_ nanowire. (D) PL evolution of nanowire heterostructure as a function of anion interdiffusion time due to the heat treatment. (E,F) Halide concentration profiles of perovskite nanowire heterostructure that measured from confocal PL. Reproduced with permission from ref (255). Copyright 2018 National Academy of Sciences of the United States of America.

Heterostructures of single-crystalline CsPbX_3_ perovskite NWs with two different halide species (CsPbBr_3_–CsPbCl_3_) were used as a model system to understand ionic diffusion in halide perovskites (Figure 19A,B).^255^ The heterostructures exhibit two-color PL emission with a sharp interface. The sharp interface, with one-dimensional control, makes these highly crystalline heterojunctions ideal systems to study the intrinsic halide anion interdiffusion because of the well-defined morphology and absence of grain boundary. The changes in surface potential between two components show distinctive electronic properties across the heterostructure NW. The single-crystalline CsPbX_3_ NWs that were grown on epitaxial substrates were also used to study the kinetics of ion exchange.^256^ For example, CsPbCl_3_, MAPbBr_3_, or MAPbI_3_ microplates were grown from the solution-based approaches and transferred on top of aligned CsPbBr_3_ NWs on fluorinated-mica substrates. The corresponding solid-state anion interdiffusion could be studied using time-dependent confocal PL microscopy (Figure 19C–F). The temperature-dependent measurements revealed the interdiffusion coefficient of chloride to bromide, along with an activation energy of 0.44 eV.

The variation in electronic band gaps can be exploited to monitor not only the ion migration, but also solid-state phase transition dynamics. *In situ* characterization of the phase transition dynamics (from perovskite phase [α- or γ-phase] to non-perovskite phase [β-phase]) in CsPbIBr_2_ NWs has been probed in microscopic channels with high spatial resolution, providing an opportunity to determine the underlying relationships between physical crystal structures and their thermal/electronic properties.^257^ To observe the thermally induced phase transition dynamics, cathodoluminescence (CL) (luminescence induced by an electron beam) and secondary electron images were simultaneously collected at high frame rates with low electron dose, using a customized scanning electron microscope. The non-perovskite phase of CsPbIBr_2_ shows a larger, indirect band gap, with a low PL emission intensity, and the perovskite phase of CsPbIBr_2_ shows instead a smaller, direct band gap with a bright PL emission. The difference in emission wavelength yields distinctive contrast in CL imaging, which allows one to track the phase transition dynamics. The phase propagation rates along the NWs were measured by increasing the temperature from 163 to 182 °C. An activation energy of 210 ± 60 kJ/mol was extracted, pointing toward an Arrhenius-like behavior. The microscopic mechanism of phase propagation dynamics was studied from the molecular dynamics simulations, revealing the structurally disordered, liquid-like interface as the origin of the increase in entropy for interphase boundary propagation.

Additionally, p–n junction formation can be fabricated with the single-crystalline CsSnI_3_ NWs by utilizing a localized, thermally driven phase transition.^258^ CsSnI_3_ undergoes a thermally driven phase transition from the double-chain non-perovskite yellow phase to the orthorhombic black perovskite phase at ∼150 °C, and the formation energies of cation and anion vacancies in these two phases are significantly different, which leads to n- and p-type electrical characteristics for yellow and black phases. The carrier mobility of black phase CsSnI_3_ is ∼400 cm^2^ V^–1^ s^–1^, while that of the yellow phase CsPbSnI_3_ is 2 orders of magnitude lower (∼0.9 cm^2^ V^–1^ s^–1^). Also, using the CL microscopy technique, the interface formation and propagation between two phases could be directly monitored.

**Figure 20:**
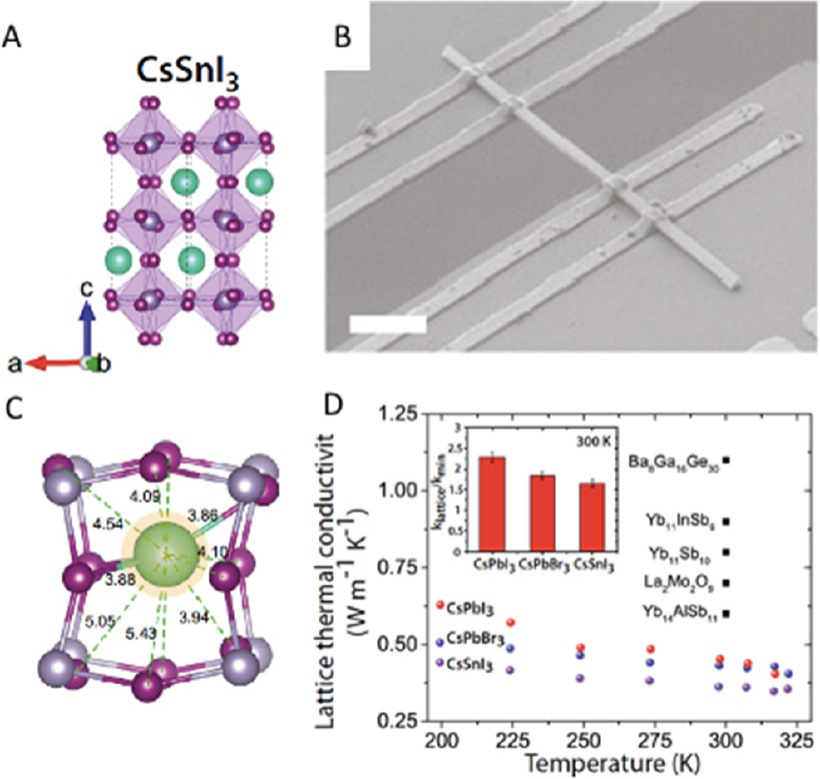
(A) Crystal structures of CsSnI_3_ perovskite. (B) SEM images of single nanowire on microisland device. (C) Inhomogeneous bonding structure of atomic cluster rattling mechanism in CsSnI_3_. (D) Comparison of thermal conductivity in perovskites and other crystals. Reproduced with permission from ref (259). Copyright 2017 National Academy of Sciences of the United States of America.

Perovskite NWs have received considerable attention in lasing (see Lasers section) and optoelectronic devices. Therefore, exploring the thermal transport properties of single-crystalline solids is crucial for developing microelectronic devices. One of the distinctive characteristics of halide perovskite NWs is the coupling between inonic crystal lattice and the confining one dimensional geometry. Combined with the heavy elements (Pb, Sn) in the halide perovskite structure, thermal conductivity in halide perovskites can be greatly reduced, which may significantly boost the thermoelectric performance (Figure 20),^259^ especially when the diameter of NW is smaller than the length of the phonon mean free path. The thermal conductivity has been shown to be ultralow (∼0.5 W m^–1^ K^–1^ at room temperature) in CsPbI_3_, CsPbBr_3_, and CsSnI_3_ perovskite NWs. Interestingly, these NWs exhibit crystal-like thermal conductivity in which the lattice thermal conductivity initially increases and then decreases as the temperature increases. The ultralow thermal conductivity of inorganic perovskite NWs was attributed to the cluster rattling mechanism based on phonon–phonon scattering measurements.^259^ Compared to the inorganic perovskites, a large reduction of thermal conductivity (0.22 W m^–1^ K^–1^) was observed in the organic–inorganic hybrid MAPbBr_3_ NWs.^260^ In addition, temperature-dependent measurements revealed the dynamic disorder of the organic cations in MAPbBr_3_ NWs, which affects the thermal conductivity at low temperature.^260^ On the other hand, the effects of phonon group velocity and the high Umklapp scattering rate are dominant in MAPbI_3_ NWs at high temperatures.^260^

#### Synthesis of Organic–Inorganic Hybrid Perovskite Nanowires

Unlike in the case of colloidal inorganic CsPbX_3_ perovskite NWs, only limited research progress has been made regarding the controlled synthesis and applications of colloidal OIH perovskite nanowires. Most of the studies on OIH perovskite NWs have been focused on growing them on substrates for optoelectronic and photovoltaic applications.^261−266^ In 2014, Horváth *et al.*(266) reported the fabrication of methylammonium lead iodide (CH_3_NH_3_PbI_3_: MAPI) perovskite NWs by a slip-coating method. This method relies on drying a saturated solution of MAPI dissolved in DMF in a confined volume between two glass plates. However, the NWs were rather thick, with a diameter in the range of 50 and 400 nm. In a subsequent work, Im *et al.* demonstrated the fabrication of dense MAPI NWs films for solar cell applications.^267^ The NWs were grown on a TiO_2_ layer substrate by two-step spin-coating using a DMF–isopropyl alcohol (IPA) solution of MAPI precursor. It was found that the amount of DMF and the concentration of MAPI in the precursor solution is critical for NW formation, and the thickness and length of the NWs can be controlled by varying the amount of DMF. In a follow-up work, the same group carried out a detailed analysis of the intermediate structures during the crystallization of NWs, and they found that the intermediate phase MAI-PbI_2_-DMF acts as a structure-directing agent.^268^ Interestingly, it was found that the treatment of perovskite thin films with a mixture of DMF/IPA could also lead to the formation of perovskite NWs through dissolution and recrystallization.^261^ In addition, predesigned templates could also be used to guide the crystallization of perovskite into NWs. For instance, Spina *et al.*(269) demonstrated the fabrication of MAPI NW arrays in open nanofluidic channels, by which it was possible to control the thickness, length, cross-sectional shape, and orientation of the NWs. Similarly, anodized aluminum oxide templates were used for the fabrication of uniform perovskite (CH_3_NH_3_PbI_3_ and CH_3_NH_3_PbBr_3_) NW arrays with a controlled diameter (50–200 nm) on ITO substrates.^270^ The NWs prepared by these template approaches appear to have rather rough surfaces. Similar to the case of inorganic perovskite NWs, it has been shown that high-quality HOI perovskite NWs with smooth surfaces and a rectangular cross section can be prepared on silicon substrates by vapor-phase synthesis.^271^ This is a two-step fabrication process. First, chemical vapor disposition of PbX_2_ precursor powders at high temperature leads to the formation of PbX_2_ NWs, which then convert into MAPbX_3_ by chemical evaporation of MAX in the same reaction chamber.^271^ These OIH perovskite NWs exhibit room-temperature lasing characteristics upon optical pumping.

A few attempts have been made toward the solution-phase synthesis of high-quality OIH perovskite NWs by the LARP approach.^272−274^ This approach was initially applied to obtain brightly luminescent small NCs. However, this reaction generally yields a side product consisting of larger nanocubes and NWs in the sediment. Zhang *et al.*(272) showed that this LARP reaction produces either high-quality larger MAPbBr nanocubes or NWs upon stirring the reaction mixture for longer times (24 h). The morphology is controllable from nanocubes to NWs by adjusting the amount of ligand solution (octylamine). Debroye *et al.*(273) further extended this approach to MAPI NWs. They used both oleylamine (OLA) and oleic acid (OA) as ligands and found that the length of the NWs increases with increasing the amount of OLA in the reaction medium with a fixed amount of OA. This was attributed to the differences in surface binding kinetic of two different ligands to specific crystal facets.^273^ The NWs were found to be single-crystalline and they exhibit longer PL lifetimes. However, the exact mechanism behind the morphology control is still unexplored.

### Synthesis of MHP NCs on Substrates (*In Situ* Synthesis)

Despite the great success of HT and LARP methods in the shape-controlled synthesis of high-quality perovskite NCs, they also suffer from their fragile surface chemistry and instability. In particular, preserving their superior optical properties when processing them into thin films or embedding them into solid matrix has been challenging. To overcome such problems, an *in situ* synthesis strategy (*i.e.*, synthesis on a substrate) has been employed to colloidal synthesis since the 1990s.^275^ Because of the high formation enthalpy of II–VI seminductors, the *in situ* fabrication of conventional quantum dots usually requires high reaction temperature, which affects their optical properties with large full width at half-maximum (fwhm) and low PLQY.^276^ On the other hand, perovskites are ionic semiconductors with low formation enthalpy and are defect-tolerant.^98,277^ These two features make the *in situ* synthesis strategy well-suitable for fabrication of high-quality MHP NC-based nanocomposites for color conversion applications^278^ or MHP NC thin films for electroluminescence devices.^224^ Through this approach, MHP NCs can be directly synthesized in a hard matrix such as porous aluminum oxides,^279^ glasses,^280−282^ molecular sieves,^283^ or in a soft polymeric matrix.^278^ It is worth mentioning that the *in situ* fabricated perovskite NC–polymer composite films have been successfully applied in TCL TV products.^284^

**Figure 21:**
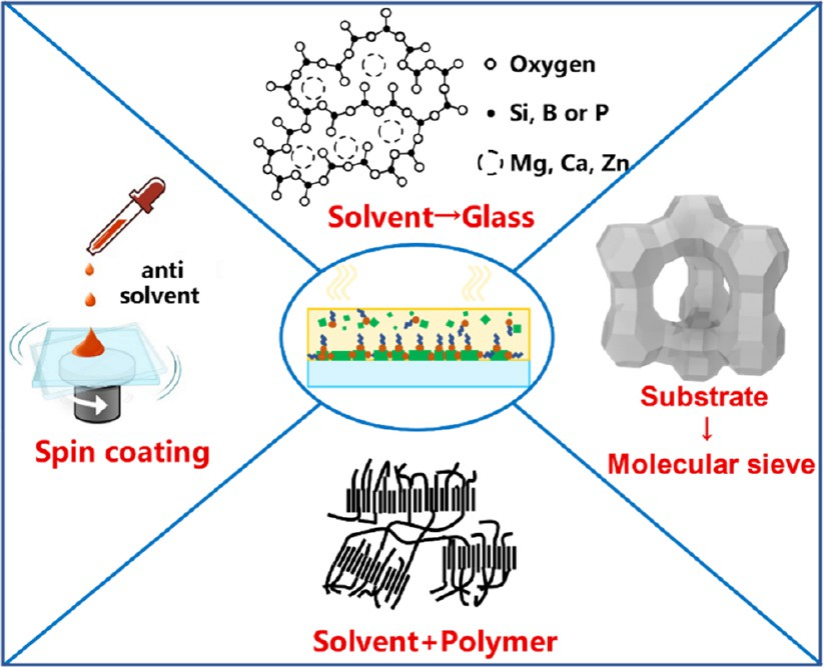
Schematic illustration of four types substrates used in *in situ* synthesis for perovskite NC composites.

Considering the unique advantages of this approach, there has been a growing interest in *in situ* synthesis of perovskite NCs directly on a substrate or in a matrix. As illustrated in Figure 21, mainly four types of substrates have been reported for *in situ* synthesis of perovskite NC composites: (1) glass matrix (for NC-doped glasses, only suitable for inorganic perovskite NCs due to high reaction temperature), (2) molecular sieves, (3) polymer matrix, (4) glass surface (for obtaining perovskite NC films by *in situ* LARP approach). The first three substrates offer a constrained space for perovskites to crystallize it, which can be called nanoconfined crystallization. However, unlike solution-phase colloidal synthesis, the shape of the NCs cannot be controlled with these *in situ* synthesis strategies.

**Figure 22:**
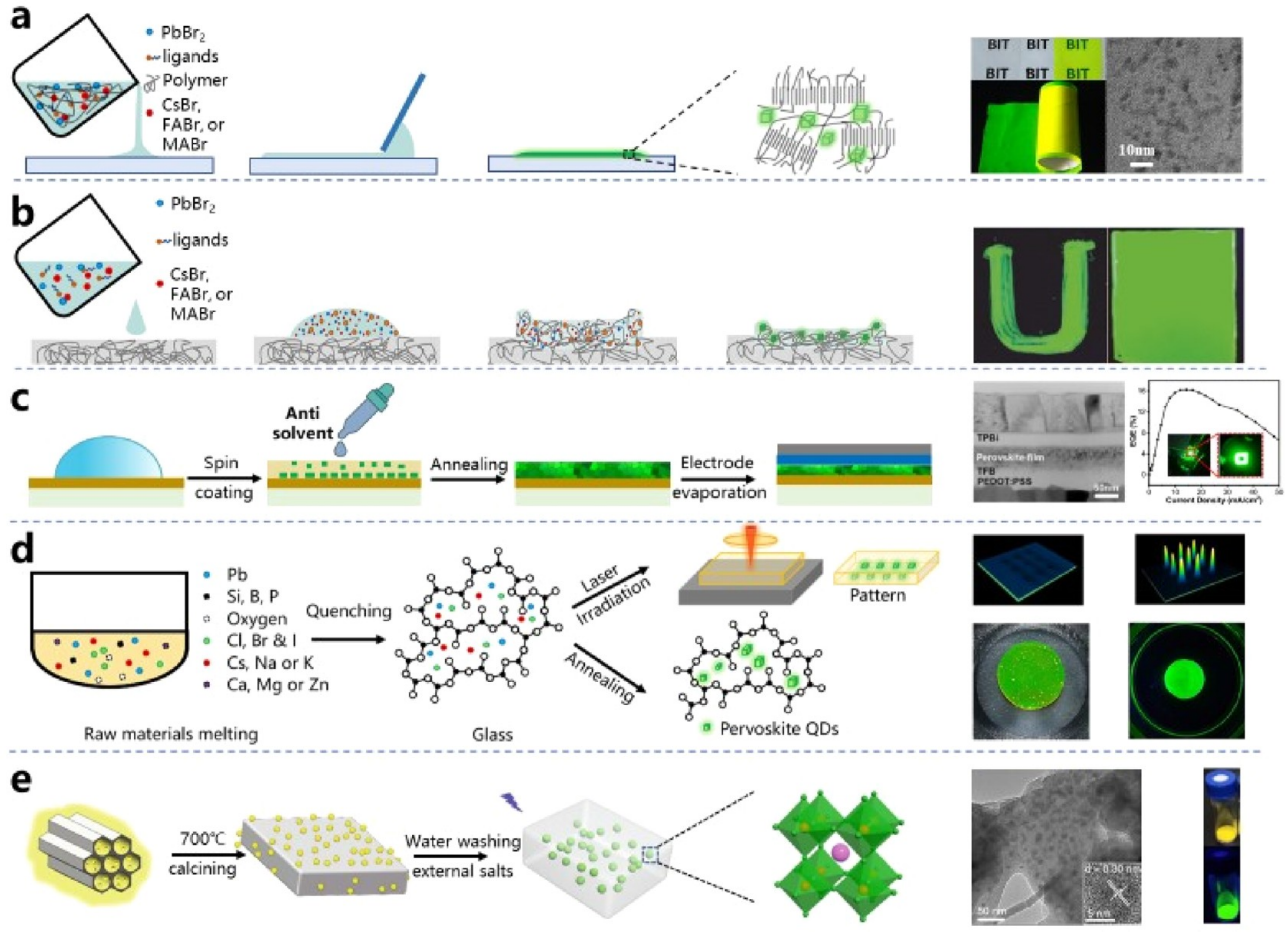
(a) Schematic illustration of the fabrication of perovskite NC composite by blade coating of precursor solution. The insets are the photographs of the luminescent film under sunlight and UV light and the TEM images of sliced films (right side). Reproduced with permission from ref (278). Copyright 2016 John Wiley & Sons, Inc. (b) Schematic illustration showing the fabrication of nanocomposites preparation with swelling–deswelling microencapsulation strategy. The insets are the images of the luminescent nanocomposite prepared by swab painting and spin coating under UV light (right side). Reproduced with permission from ref (285). Copyright 2016 John Wiley & Sons, Inc. (c) Schematic illustration of the fabrication of LED device based on NCs film prepared by *in situ* LARP progress. The insets are the TEM image of a device cross section and the plot of EQE *vs* current density of the device. Reproduced from ref (224). Copyright 2018 American Chemical Society. (d) Schematic illustration of the fabrication of perovskite NC glass composite and photographs of glass substrates having pattered NCs in the glass matrix (by either laser irradiation) and uniformly distributed NCs (by uniform annealing). The top panel on the right side reproduced with permission from ref (282). Copyright 2020 Nature Publishing Group. The bottom panel on the right side is reproduced with permission from ref (281). Copyright 2020 John Wiley & Sons, Inc. (e) Schematic illustration of perovskite NC-embedded molecular sieve phosphors. The insets are the TEM images and the photos of the phosphors under sunlight and UV light (right side). Reproduced with permission from ref (283). Reprinted with permission under a Creative Commons CC BY license. Copyright 2020 The Author(s).

As shown in Figure 22a, Zhong and co-workers developed the *in situ* fabrication strategy to obtain flexible and free-standing perovskite NC–polymer composite films.^278^ The fabrication process exploited the solubility difference between polymer and perovksites, enabling the formation of small size NCs in the polymeric matrix. The as-prepared composite films exhibit improved stability and enhanced PL emission, along with excellent mechanical and also piezoelectric properties. Furthermore, the authors demonstrated the early liquid crystal display backlights based on perovskites. Meanwhile, Wang *et al.*(285) demonstrated a swelling–deswelling microencapsulation strategy for the fabrication of MAPbBr_3_–polymer composites (Figure 22b). In this approach, the introduction of the perovskite precursor solution into the polymer matrix leads to solvent-induced polymer swelling, which then deswells after the removal of the solvent by annealing. In 2018, Zhong’s group demonstrated the *in situ* synthesis of highly luminescent FAPbBr_3_ NC films on ITO-coated glass substrates.^224^ Their approach relies on the crystallization of smooth NC film directly on a substrate by LARP (Figure 22c). The prepared films exhibited bright luminescence with a PLQY up to 78%. They demonstrated that the green LEDs made out of these films exhibit external quantum efficiency up to 16.3%. Figure 22d illustrates a synthesis route for the preparation of a perovskite NC–glass composite. This method relies on heating (at 1300 °C) and then quenching a mixture of perovskite precursors (PbO, CsCO_3_, KX, and so on) and glass melt (SiO_2_, B_2_O_3_, and P_2_O_5_, and so on) to obtain a transparent glass substrate embedded with perovskite precursor.^282^ The precursors in glass matrix can be transformed into perovskite NCs either by laser irradiation or by thermal annealing. By precisely controlling the laser focal point, one can draw reversible fine patterns of perovskite NCs in the glass matrix (inset of Figure 22d). On the other hand, a uniformly doped luminescent glass substrate can be produced by thermal annealing at 400–600 °C (Figure 22d, right side).^281^ A similar strategy could be applied to obtain perovskite NC-doped phosphors using a mixture of perovskite precursors and a molecular sieve, as shown in Figure 22e.^283^ In this approach, highly luminescent perovskite NC-doped phosphor with ultrahigh stability can be achieved by washing away the unbound perovskite NCs.

### Composition Control by Ion Exchange and Suppression of Exchange

#### Anion Exchange

##### Halide Exchange and Mixed-Halide NCs

The band gap and therefore the color of the emission in lead-halide perovskite NCs is mainly defined by halide atom, with CsPbCl_3_ NCs emitting in the blue, CsPbBr_3_ in the green, and CsPbI_3_ in the red visible spectral range. Mixing of the halide composition (Br_*x*_,Cl_1–*x*_; Br_*x*_, I_1–*x*_) provides the possibility of fine-tuning the emission wavelength across the visible range. Mixed-halide composition was already reported in the early report on colloidal lead-halide perovskite NC by Protesescu *et al.*(14) through direct synthesis. This work was quickly followed up by reports on post-synthesis exchange of the halide anions by Kovalenko’s and Manna’s groups. Nedelcu *et al.*(55) and Akkerman *et al.*(57) showed that fast anion exchange between Cl and Br, and Br and I could be reversibly achieved by providing the halide sources to the already synthesized NCs in dry octadecene. This reaction worked for all tested halide sources, from organometallic Grignard reagents (MeMgX) to oleylammonium halides (OLAX) and simple PbX_2_ salts, without affecting the cationic sublattice and by maintaining the cubic crystals structure and the size of the parent NCs. In this way, the anion exchange provided a synthesis strategy for mixed-halide CsPbBr/I and CsPb Br/Cl NCs with good size monodispersity, which translated to improved optical properties such as emission line width and intensity. Gradual halide exchange from Cl to I or *vice versa* was not achieved; in these attempts, the NCs were either shattered^57^ or quickly converted to single halide crystals,^55^ which was attributed to the large difference between the ionic radii of Cl and I atoms. Furthermore, anion exchange was also observed without the use of additional halide sources by direct mixing of CsPbBr_3_ NCs with CsPbI_3_ or CsPbBr_3_ NCs in colloidal solutions. Here, the NCs can serve as halide sources, and fast shuttling of halide anions between NCs occurs until a homogeneous distribution within the sample is reached.

Toward the fabrication of perovskite NCs with tunable emission for lighting application, the anion exchange process was integrated in a microfluidic reactor system for the synthesis of CsPbX/Y NCs with mixed-halide composition by Kang *et al.*(286) Here, the CsPbBr_3_ NCs were fabricated in a first microreactor stage, and then the anion exchange with I and Cl occurred in a second reactor, where the respective halide precursors were added to the flow of the CsPbBr_3_ NCs that were formed in the first reactor. *In situ* control of the flow parameters of the precursors and monitoring of the PL emission enabled fine control of NC size and composition.

Anion exchange reactions also allowed to extend the range of Pb-free double perovskite NC materials. Creutz *et al.*(183) fabricated elpasolite Cs_2_AgBiX_6_ (X = Cl, Br) NCs and then used the anion exchange with I to obtain Cs_2_AgBiI_6_ NCs, which could not be prepared by a direct synthesis route. This Pb-free material is a strong photoabsorber across the visible range and is therefore attractive for photovoltaic applications.

##### *In Situ* Monitoring of Anion Exchange

The bright photoluminescence of the mixed cesium lead halides enabled *in situ* monitoring of the anion exchange dynamics in the NCl samples. Koscher *et al.* measured the PL spectra over time during the anion exchange reaction from CsPbBr_3_ to CsPbCl_3_ and CsPbI_3_ NCs in solution ensuring fast injection by a stopped-flow injector.^287^ The reaction kinetics were analyzed *via* the band gap and PL line width change during the chlorine and iodine exchange. These experiments allowed them to draw a kinetic model for the exchange reaction process, in which distinctly different behaviors were observed for the two reactions. The red shift of the band gap in the exchange from CsPbBr_3_ to CsPbI_3_ followed a monoexponential trend, and this rapid initial alloying was attributed to a surface-limited process. The more complex kinetics for the exchange with chlorine, which manifested with different time intervals with nearly constant band gap change, could be assigned to a diffusion-limited dynamics. Such different behavior was rationalized by the differences in ion sizes and mobilities. The anion exchange reaction in single-crystal perovskite nanoplates (with tens of micrometer lateral size) could be monitored by following the change in PL of individual platelets with a confocal microscope.^288^ Since this study was not done *in situ*, vapor-phase anion exchange reaction on dry CsPbBr_3_ nanoplates was used that ensured rapid quenching of the reaction. At the intermediate stages of the anion exchange from CsPbBr_3_ to CsPbI_3_, a coexistence of red and green emission peaks was observed in the PL spectra. Confocal PL maps recorded on nanoplates with different thicknesses and at different reaction times evidenced a gradual transformation from the edges toward the center of the plate, with dynamics that correspond to a diffusion-controlled mechanism.

The reversible reaction from CsPbCl_3_ to CsPbBr_3_ nanoplatelets was investigated by *in situ* PL spectroscospy by Wang *et al.*,^289^ revealing heterogeneity in the reaction kinetics that depend on the density of the exchanged ions in the crystals. By selecting different fields of view in the micro-PL measurements, the time traces of the emission of individual NCs were recorded, which manifested a strong dependence for the switching times on the concentration of substitutional halide ions used to induce anion exchange.

##### Heterostructure Fabrication *via* Anion Exchange

Anion exchange can be exploited to fabricate heterojunctions in lead-halide perovskite NCs. Huang *et al.* have shown in their progress report^106^ a variety of lateral heterostructures in perovskite nanowires. CsPbBr_3_ nanowires with different diameters were fabricated by wet chemistry, coated with poly(methyl methacrylate) (PMMA), and selected regions were exposed by electron beam lithography. By applying anion exchange with chlorine and iodine precursor solutions, lateral heterojunctions with spatial resolution down to 500 nm were achieved and imaged by confocal fluorescence microscopy (Figure 25). Mixed-halide heterojunctions were also fabricated starting from CsPbBr_3_ nanocubes with an anion exchange to CsPbI_3_ and imaged by variable energy hard X-ray photoelectron spectroscopy.^290^ These measurements elucidate, in contrast to a homogeneous alloy, that the anion exchange progresses *via* the formation of a heterojunction from the outer regions to inner regions of the nanowires, where the surface is rich with the exchanged anions and the core with the native ones. Even in fully exchanged nanocubes, a small core region containing the native (Br) anions was observed.

**Figure 23:**
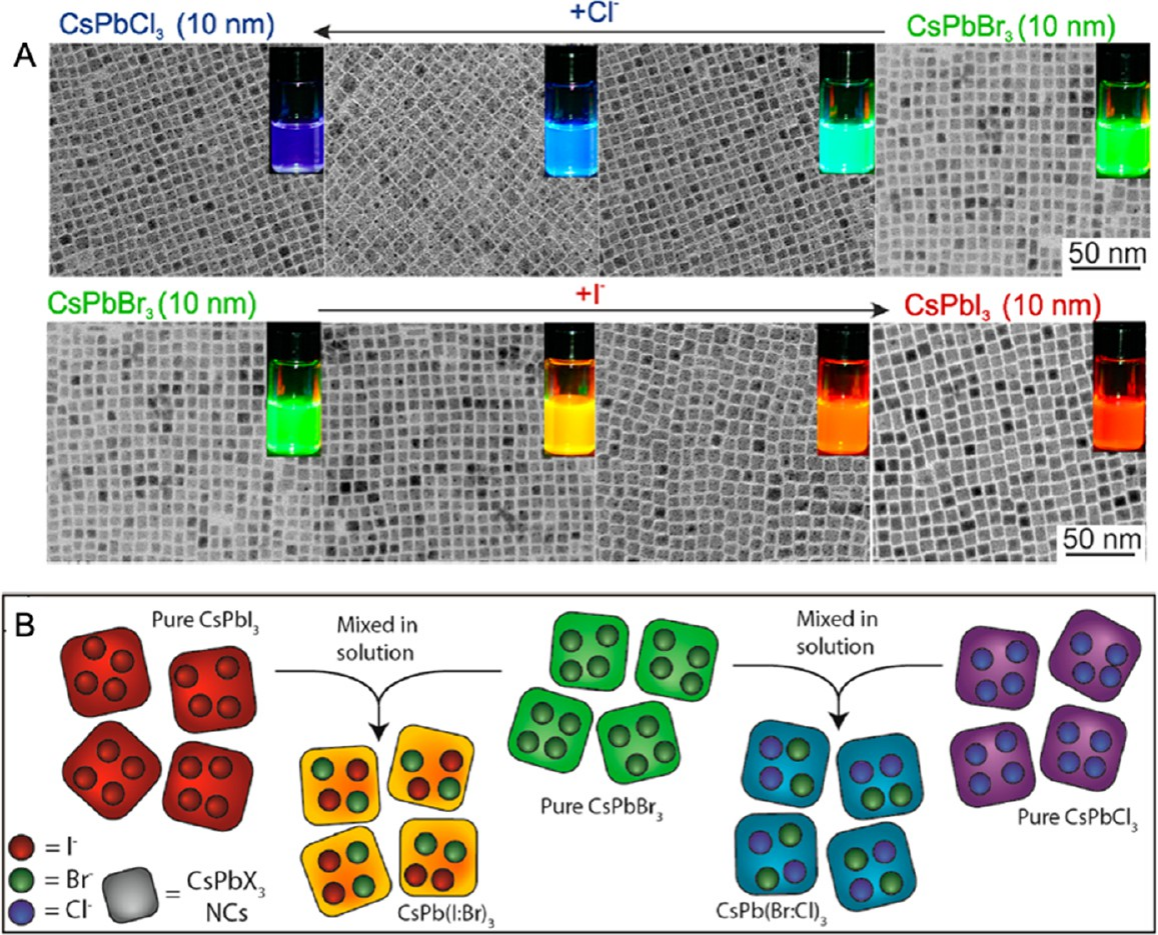
Post-synthesis halide exchange. (A) TEM images of CsPbX_3_ nanocubes with mixed-halide composition. The insets show photographs of the colloidal solutions under ultraviolet light illumination. Reproduced from ref (55). Copyright 2015 American Chemical Society. Further permissions related to the material excerpted should be directed to the ACS. (B) Schematic illustration of the anion exchange reaction that occurs upon mixing NC solutions with different halides. Reproduced from ref (57). Copyright 2015 American Chemical Society. Further permissions related to the material excerpted should be directed to the ACS.

**Figure 24:**
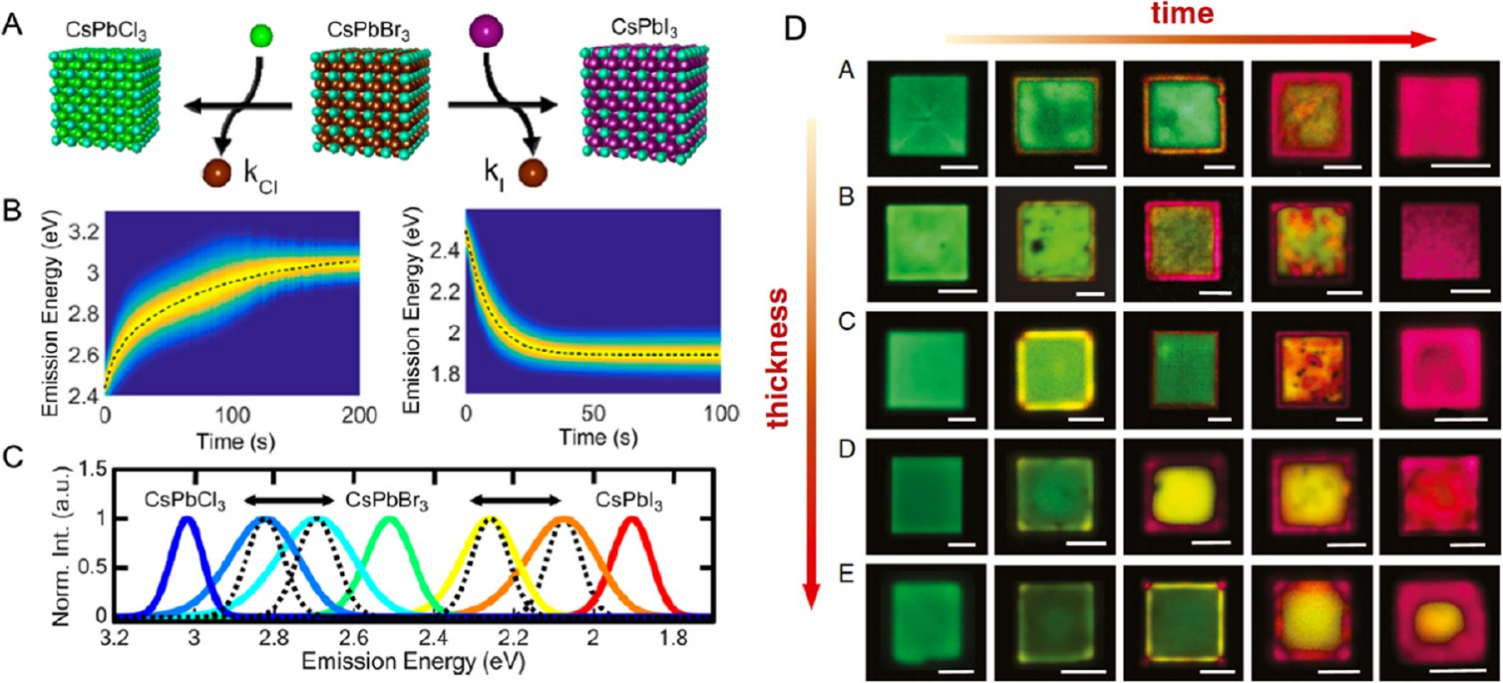
*In situ* photoluminescence monitoring during halide exchange reactions. (A) Schematic illustration of the exchange reaction. (B) PL spectra recorded from colloidal solutions during the anion exchange reactions. (C) PL spectra for the starting CsPbBr_3_ NCs (green) and ending CsPbCl_3_ (dark blue) or CsPbI_3_ (red) along with spectra for mixed-halide compositions (CsPbBr_3–*y*_X_*y*_) in the both the kinetic (solid) and equilibrium (dashed) regime for each band gap shown. Panels A–C are reproduced from ref (287). Copyright 2016 American Chemical Society. (D) Confocal PL mapping of individual nanoplates for different thicknesses and reaction times. Reproduced with permission from ref (288). Copyright 2019 National Academy of Sciences of the United States of Amercia.

**Figure 25:**
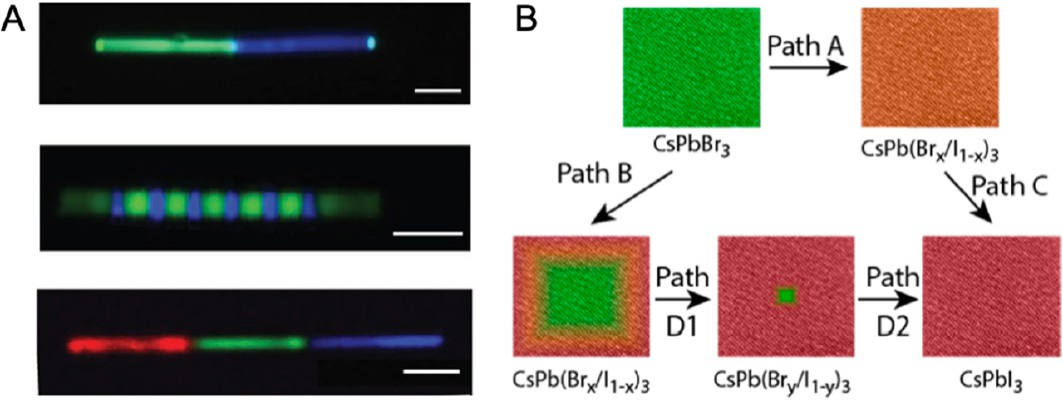
Heterojunctions fabricated by anion exchange reactions. (A) Heterojunctions obtained by masked anion exchange in a CsPbX_3_ nanowire, leading to different halide compositions. Reproduced with permission from ref (252). Copyright 2017 National Academy of Sciences of the United States of America. (B) Schematic illustration of core–shell structures obtained by post-synthesis halide exchange. Reproduced from ref (290). Copyright 2018 American Chemical Society.

Colloidal atomic layer deposition has been employed to fabricate perovskite/metal oxide heterojunctions in NCs.^291^ Here, for the case of alumina-coated CsPbBr_3_ nanocubes, the oxide shell protected the perovskite NC core from anion exchange reactions, which significantly increased the photoluminescence quantum yield and slowed down the kinetics of the anion exchange, which made monitoring by X-ray diffraction possible.

**Figure 26:**
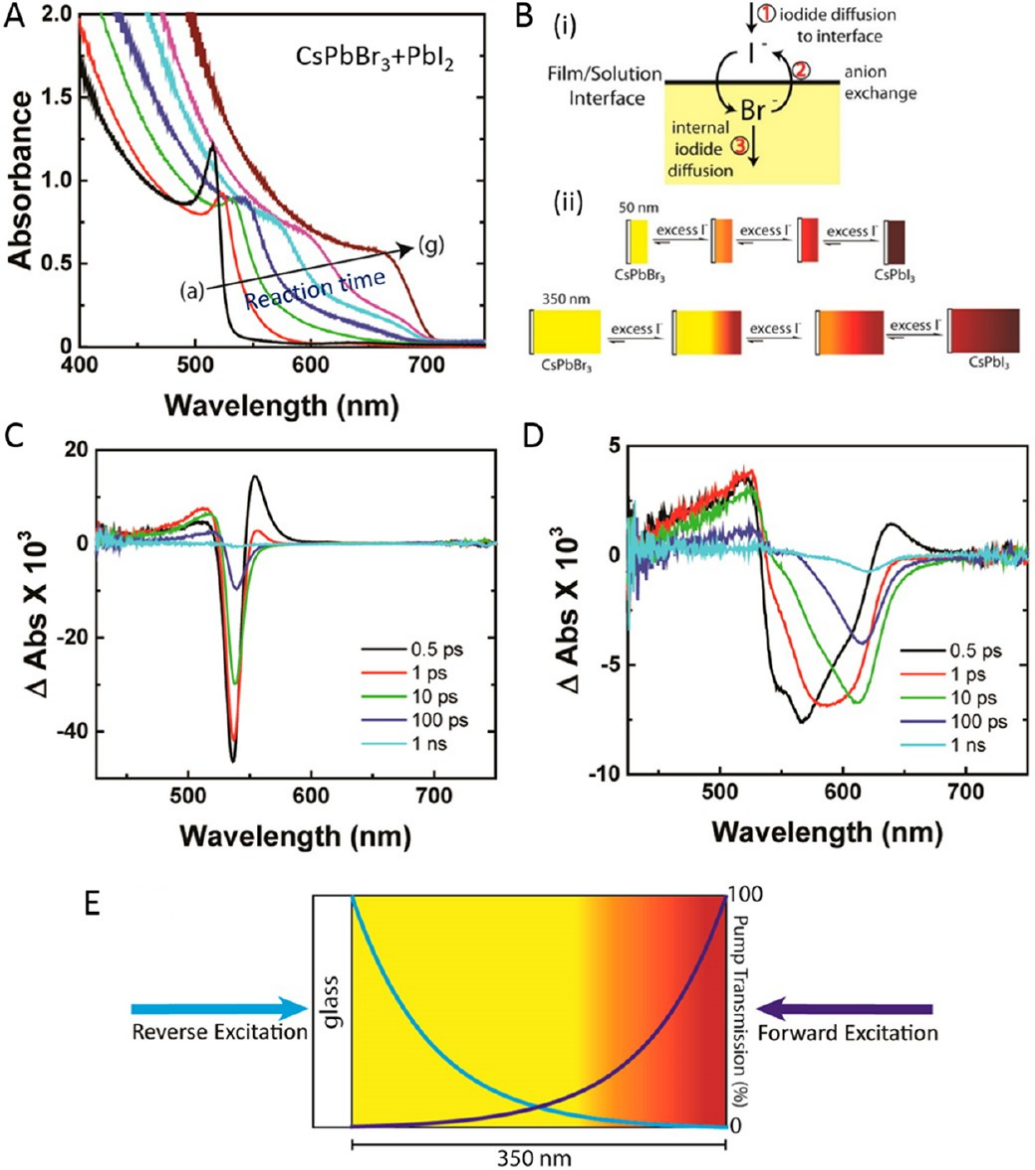
(A) UV–visible extinction spectra of CsPbBr_3_ films (350 nm thick) soaked in PbI_2_ solution at 120 °C for (a) 0, (b) 5, (c) 15, (d) 30, (e) 60, (f) 150, and (g) 480 min. (B) Schematic illustrations showing (i) three-step halide exchange reaction: iodide ions diffuses to the film–solution interface and exchanges with bromide, and the internal iodide diffuses away from the interface; (ii) differences in internal film structure of exchanged films of thickness 75 and 350 nm. (C,D) Transient absorption (TA) spectra of the 15 min soaked 350 nm thick film, acquired upon reverse (C) and forward (D) excitation. The TA spectra acquired under reverse excitation matches well with the steady-state absorption peak (panel A(d)), indicating that the signals originates from within the minimally exchanged portion of the thick film. Forward excitation gives rise a broad bleach spectra moving across the visible spectrum, indicating the excitation of the film surface at the compositional gradient. (E) Schematic representation showing the transient absorption experimental setup for study of thick film. The 387 nm pump can be completely absorbed by the film with an estimated penetration depth of 67 nm, leading to the significant differences in the position of the film where the pump is absorbed when exciting from the forward or reverse direction. Reproduced from ref (292). Copyright 2016 American Chemical Society. Further permissions related to the material excerpted should be directed to the ACS.

In another study, a sintered CsPbBr_3_ nanocrystalline film was converted into a cubic CsPbI_3_ film by exchanging bromide with iodide ions (Figure 26A). This approach enabled a gradient structure to be created with CsPbBr_3_ at one side and CsPbI_3_ on the other side of the film.^292^ The exchange reaction proceeds through three steps, as illustrated in Figure 26B(i). The halide anion exchange rate is most likely governed by the anion exchange at the interface and the internal diffusion of newly formed iodide domain. In thinner films, the iodide ions can diffuse throughout the film, leading to a near-uniform film composition (Figure 26B(ii)). However, in the case of thick films, iodide ions cannot diffuse as fast as the additional iodide ions enter at the interface, causing a compositional gradient across the film (Figure 26B(ii)). Time-resolved transient absorption studies confirmed the migration of charge carriers from the high-band-gap CsPbBr_3_ and CsPbBr_*x*_I_3–*x*_ regions to the iodide-rich region near the film surface with in few picoseconds after excitation (Figure 26C–E). The transient absorption spectra exhibited a narrow bleach upon reverse excitation (Figure 26C), which is consistent with steady state absorption spectra (Figure 26A). However, the bleach peak became broad when the excitation was switched to the forward side, and the peak shifted to lower energies with increasing time delay (Figure 26D). A time constant of 0.5 ps was estimated from the growth of bleaching of the iodide region. These differences in the transient absorption spectra were attributed to the inhomogeneous distribution of anions in thick films as compared to that of thin films after halide ion exchange (Figure 26E). Thus, the gradient films prepared through the halide ion exchange can direct charge carrier-funneling behavior and could improve charge separation and transportation in optoelectronic devices. Because of the miscibility of different halides, such gradient structures are extremely sensitive to temperature and can quickly homogenize at higher temperatures.^293,294^

#### Suppression of Anion Exchange

**Figure 27:**
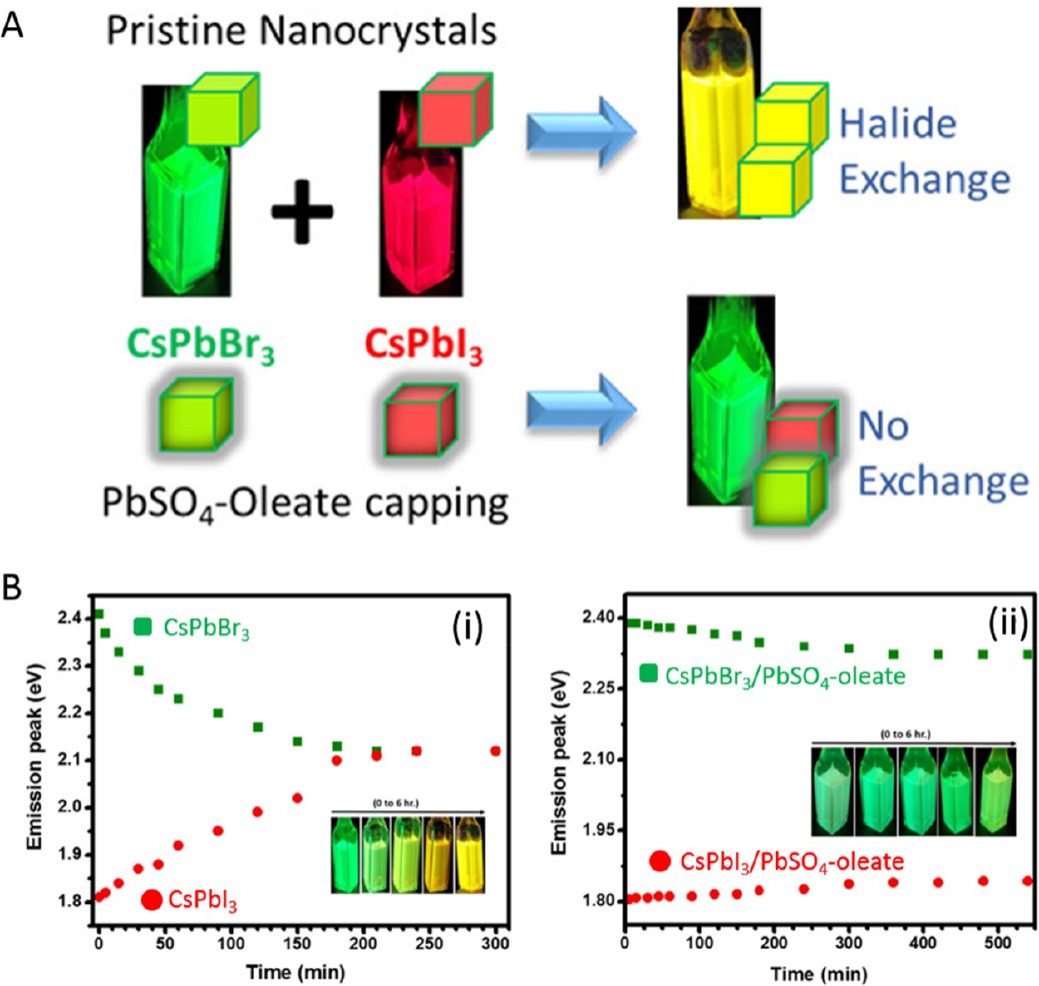
(A) Schematic illustration showing the halide exchange and no exchange with and without PbSO_4_-oleate capping. (B) Changes in photoluminescence peak energy with time of (i) pure CsPbBr_3_ and CsPbI_3_ NCs and (ii) PbSO_4_-oleate-capped CsPbBr_3_ and CsPbI_3_ NCs after mixingthem in hexane solution. The insets show photographs of the colloidal NC mixture under UV light at various mixing times. Reproduced from ref (296). Copyright 2018 American Chemical Society. Further permissions related to the material excerpted should be directed to the ACS.

In many device applications, it is important that the anion exchange be suppressed between different layers of metal-halide perovskites. For example, in an all-perovskite tandem solar cell one would like to maintain the individual mixed-halide compositions in order to retain the aligned band structure of the films. The ease of halide exchange between different lead-halide perovskite films^293,294^ requires therefore the suppression of anion exchange. One such effective strategy is to cap CsPbBr_*x*_I_3–*x*_ NCs with PbSO_4_-oleate (Figure 27A).^295,296^ These capped NCs align linearly and can be deposited as films with a hierarchical nanotube architecture. The suppression of the halide ion can be seen in both NC suspension as well as multilayered films. For example, Figure 27B shows the emission changes during anion exchange and suppression of anion exchange with PbSO_4_-oleate capping of CsPbBr_3_ and CsPbI_3_ NCs. Moreover, Palazon *et al.* found that the CsPbX_3_ NC films exposed to low flux of X-rays do not undergo halide anion exchange.^297^ This is because of the organic shell formed on the surface of NCs through intermolecular C=C bonding within ligands upon exposure to X-rays. This approach enabled the fabrication of fluorescent patterns over millimeter scales with greater stability. By suppressing halide ion exchange, it was possible to mix lead-halide perovskite NCs and have a broader emission in the visible region of the spectrum, including white emission.^296^ Significantly suppressed ion migration has also been achieved in layered perovskites.^298^

#### Cation Exchange

##### “A” Cation Exchange

One of the early observations of “A” cation exchange on halide perovskites was reported in a work describing halide anion exchange reactions in CsPbX_3_ NCs by Akkerman *et al*.^57^ In that work, various halide sources were explored to elicit anion exchange, starting from CsPbBr_3_ NCs and going to CsPbCl_3_ and CsPbI_3_ (and back). On the other hand, exposing the CsPbBr_3_ NCs to methylammonium bromide caused their PL to red shift from 2.43 to 2.36 eV, a value in line with that observed from MAPbBr_3_ NCs. The exchange of Cs^+^ with MA^+^ was corroborated by the X-ray diffraction (XRD) pattern of the sample after the reaction, which indicated a lattice expansion, compatible with the larger size of the MA^+^ cation compared to Cs^+^. It is interesting to note that exchange of the Cs^+^ cation with smaller cations (Rb^+^, K^+^) attempted by Nedelcu *et al.*(55) led instead to the decomposition of the NCs. This was rationalized by hypothesizing that the cation sublattice in halide perovskites is much more rigid than in other compounds, for example, metal chalcogenides, where instead cation exchange occurs easily.^299^ A partial methylammonium (MA^+^) to formamidinium (FA^+^) cation exchange was also observed by Xie *et al.*(300) during the deposition of a film of MaPbI_3_ from a solution containing both MA^+^ and FA^+^ cations: even though MaPbI_3_ was formed first, it evolved in 2 min to FA_0.85_MA_0.15_PbI_3_, a composition that was observed to stabilize the α-phase.

**Figure 28:**
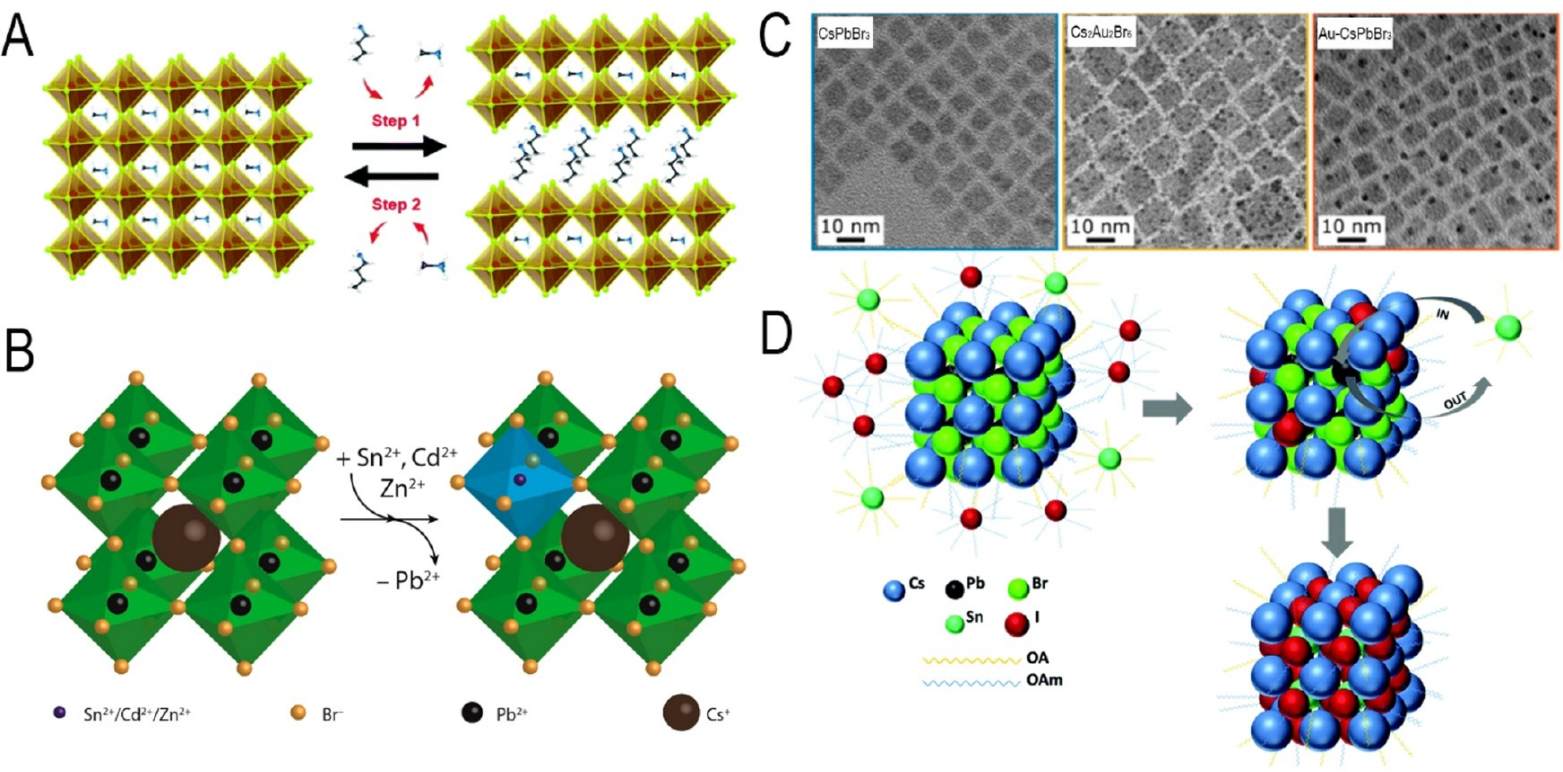
(A) Sketch of the “A” cation induced transformation of 3D to 2D perovskite structures, and back. Reproduced with permission from ref (302). Copyright 2018 Royal Society of Chemistry. (B) Partial “B” cation exchange in CsPbB_3_ NCs. Reprinted under CC-BY-NC-ND license from ref (304). Copyright 2017 American Chemical Society. (C) Competition between Au metal deposition and Pb^2+^ for Au^3+^ cation exchange in CsPbBr_3_ NCs. From left to right: TEM images of starting CsPbBr_3_ NCs, CsPbBr_3_ NCs after Pb^2+^ for Au^3+^ cation exchange, CsPbBr_3_–Au heterostructures. Reproduced from ref (305). Copyright 2017 American Chemical Society. (D) I^–^ anion driven Sn^2+^ cation exchange in in CsPbB_3_ NCs. Reproduced with permission from ref (306). Copyright 2018 Royal Society of Chemistry.

Partial “A” exchange, followed by a phase transformation, was reported by Wang *et al.*,^301^ who treated CsPbBr_3_ NCs with rubidium-oleate. The exchange of Cs^+^ with Rb^+^ ions was limited to the surface of the NCs. Also, it was accompanied by a phase transition to the Rb_4_PbBr_6_ structure, leading to the formation of core/shell CsPbBr_3_/Rb_4_PbBr_6_ NCs with improved stability and enhanced PLQY compared to the core “only” CsPbBr_3_ NCs. Another example of “A” exchange triggering a phase transformation is the one provided by Huang *et al.*,^302^ who started from MAPbBr_3_ NCs and reacted them with phenethylammonium bromide (PEABr). The large size of the PEA^+^ cations makes the 3D perovskite phase unstable, hence their introduction in the lattice causes a transition to the layered phase, accompanied by a blue shift of the emission to 411 nm, as the layered material has a higher band gap than MAPbBr_3_ (Figure 28A).^302^ The reverse reaction took place when MA^+^ ions were added to the 2D NC solution (Figure 28A).^302^ Partial exchange of MA^+^ ions with Cs^+^ ions in films of MAPbI_3_ was found to be essential to preserve the black γ-phase and therefore to avoid the detrimental transition to the higher band gap δ-phase, which is undesirable for photovoltaic applications.^303^ In addition, the resulting films were compact and pin-hole-free and the solar cells fabricated from such films had a power conversion efficiency of 14.1%.

##### “B” Cation Exchange, “Partial” *versus* “Full”

Initial attempts by Nedelcu *et al.*(55) to exchange the “B” cation in NCs were unsuccessful (Ba^2+^, Sn^2+^, Ge^2+^, *etc*.), as the NCs were dissolved. The initial report on successful “B” cation exchange is by van der Stam *et al.*,^304^ who could partially replace Pb^2+^ ions with various bivalent M^2+^ cations (Sn^2+^, Cd^2+^, Zn^2+^), with no major changes in the size and shape of the NCs, except for a small shrinkage due to the contraction of the unit cell, as all these cations have smaller ionic radii compared to Pb^2+^ (Figure 28B). The lattice contraction was also invoked as an explanation for the blue shift in the optical spectra (with preservation of PLQY at values over 50%) following the partial “B” cation exchange. Extensive analysis of the samples showed that the guest cations were homogeneously distributed in the NCs. The extent of the exchange was such that roughly up to 10% of the Pb^2+^ cations could be replaced. These reactions are limited by the low diffusion rate of the cations in the perovskite lattice, especially for the “B” cation.^307^ Although the reaction should be favored by the increase in entropy arising from the formation of a CsPb_1–*x*_M_*x*_Br_3_ solid solution, van der Stam *et al.* argued that the replacement of Pb^2+^ ions with smaller cations progressively builds up compressive strain in the lattice, which tends to counter any further exchange,^304^ thus making the overall process self-limited. Another interesting point made by van der Stam *et al.* is that the cation exchange should be promoted by the presence of halide vacancies (which have low formation energies), so that any exogenous factor limiting the formation of such vacancies should also limit the exchange.^304^ In this regard, the authors considered alkylamine molecules, with their binding ability to Br^–^ ions, as being responsible for preserving a high density of Br vacancies in the NCs, through their ability to remove Br^–^ ions from the NCs. However, when working with large concentrations of MBr_2_ in solution (in the attempt to further promote Pb^2+^ for M^2+^ exchange), the amines lose this “extracting” capability (as there are already too many Br^–^ ions in solution), and the exchange slows down considerably.

Reversible partial “B” cation exchange was observed by Gao *et al.* when reacting CsPbCl_3_ NCs with Mn^2+^ ions, leading to CsPb_1–*x*_Mn_*x*_Cl_3_ NCs or even starting from CsMnCl_3_ NCs and reacting them with Pb^2+^ ions.^308^ This latter case is similar to that of Fang *et al.*,^309^ who also started from rhombohedral CsMnCl_3_ NCs and reacted them with PbCl_2_, thus forming hexagonal Cs_4_Pb_*x*_Mn_1–*x*_Cl_6_ NCs as the intermediate and then cubic CsPb_*x*_Mn_1–*x*_Cl_3_, hence undergoing through successive phase transitions during the exchange.

The hypothesis that only partial “B” cation exchange is possible in halide perovskites was actually challenged by Eperon *et al.*(310), who started from films of formamidinium tin triiodide (CH(NH_2_)_2_SnI_3_, *i.e*., FASnI_3_), which could be either partially or fully converted to FAPbI_3_. The preservation of the morphology of the films proved that this conversion did not proceed through dissolution–recrystallization but was indeed a topotactic exchange reaction. In the same work, the reverse exchange (from Pb to Sn) was demonstrated, as well, and the same processes were extended to colloidal NCs.^310^ The work demonstrated that the “B” cations, at least in selected cases, are actually mobile, thus providing a starting point for possible studies in which transient effects stemming from such B cation mobility may be identified by appropriate experimental tools.

Another notable report on “B” cation exchange is the work of Roman *et al.*(305) (Figure 28C). In their case, the exchange was actually an undesired reaction, as they were attempting to deposit an Au metal domain on top of CsPbBr_3_ NCs by adding Au^3+^ ions, in a reducing environment provided by the surfactant molecules (oleic acid and oleylamine). The exclusive formation of Au-CsPbBr_3_ heterostructures was possible only if PbBr_2_ was added together with the Au^3+^ ions, so that Pb^2+^ could efficiently outcompete the Au^3+^ and Au^+^ ions in the exchange with the Pb^2+^ ions already present in the NCs. Indeed, when no PbBr_2_ was added, a significant side reaction was the replacement of Pb^2+^ ions by couples of Au(I) and Au(III) ions, leading to the formation of double perovskite Cs_2_Au^I^Au^III^Br_6_ NCs with tetragonal crystal structure, decorated by Au domains.

##### Simultaneous Anion–Cation Exchange

There are several reports on concomitant anion–cation exchange. For intsance, Li *et al*.^311^ started from Mn^2+^-doped CsPbCl_3_ NCs (written as CsPb_1–*x*_Cl_3_:*x*Mn^2+^), which were reacted with ZnBr_2_, such that CsPb_1–*x*–*z*_Zn_*z*_(Cl_*y*_Br_1–*y*_)_3_:*x*Mn^2+^ NCs were obtained. Hence, in this type of reaction, the Pb^2+^ (and indeed also Mn^2+^) ions were partially exchanged with Zn^2+^ and the Cl^–^ ions with Br^–^. The motivation in that work was to fabricate a system in which the concentration of Mn^2+^ dopants is still high (so that there is strong emission from Mn^2+^-derived states), and that at the same time the lattice is rich in Br^–^ ions. Apparently, it is not possible to reach high Mn^2+^ doping levels in Br^–^-dominant CsPbX_3_ hosts, but the additional presence of Zn^2+^ ions made it possible.

Various groups have actually observed that the rate of cation exchange is significantly accelerated if also anions are simultaneously exchanged, a process that has been named “anion-driven cation exchange”. In one of the early observations of this type, CsPbBr_3_ NCs were reacted with SnI_2_ and they quickly transformed to CsSnI_3_ (a process which however broadened the size distribution), going through intermediate CsPb_*x*_Sn_1–*x*_(BryI_1–*y*_)_3_ compositions (Figure 28D).^306^ A much lower reactivity was observed instead toward SnBr_2_. An interesting case of anion-driven cation exchange is the one described by Qiao *et al.*(312) who used light to trigger the degradation of dihalomethane in a solution containing CsPbX_3_ NCs (X = Cl, Br) and a sub-micromolar concentration of Mn acetate. The photodegradation reaction released halide ions, which triggered halide and Pb^2+^ to Mn^2+^ exchange at the same time. This process was named “photoinduced doping”.

A different approach, which can be nonetheless still considered as a sort of anion-assisted exchange, is the one described by Zhou *et al.*,^313^ in which CsPbCl_3_ NCs were effectively doped with Mn^2+^ ions when, in the one-pot synthesis of the NCs, trimethylchlorosilane (TMS-Cl) was present in addition to Mn acetate. The authors of the work argued that the high bond dissociation energy of the Mn–O bond in Mn acetate severely limits the availability of Mn^2+^ ions in solution and hence their possibility to be incorporated in the CsPbBr_3_ NCs. On the other hand, the rapid degradation of TMS-Cl frees a large amount of Cl^–^ ions, and as a consequence, octahedral [MnCl_6_]^4–^ complexes are formed in solution (in addition to [PbCl_6_]^4–^ complexes). These units are then directly inserted in the NCs as they nucleate and grow. The general applicability of this reaction scheme was demonstrated by extending the doping to other divalent transition metal cations (Ni^2+^,Cu^2+^, and Zn^2+^).^313^

Doping strategies aim at conferring additional physical properties to the perovskite materials, but they can also impart higher structural, chemical, and photochemical stability (including improved PLQY). A recent case was disclosed by Shapiro *et al.*,^314^ who also exploited an anion-driven cation exchange on CsPbBr_3_ NCs, using NiCl_2_ (or NiBr_2_), and were able to prepare Ni-doped CsPb(BrCl)_3_ NCs, with Ni concentrations tunable from below 1% up to 12% and higher PLQY than that of the starting NCs. When using NiCl_2_, compositional analysis showed that the extent of halide exchange was much higher than that of cation exchange. For example, to a Ni doping of 5.6% corresponded a 50:50 ratio of Br/Cl. This evidenced that, although halide ions are key to ensure the Pb^2+^ to Ni^2+^ exchange, the latter reaction still proceeds at a rate much lower than that of the concomitant anion exchange.

### Post-synthetic Nanocrystal Shape Transformations

**Figure 29:**
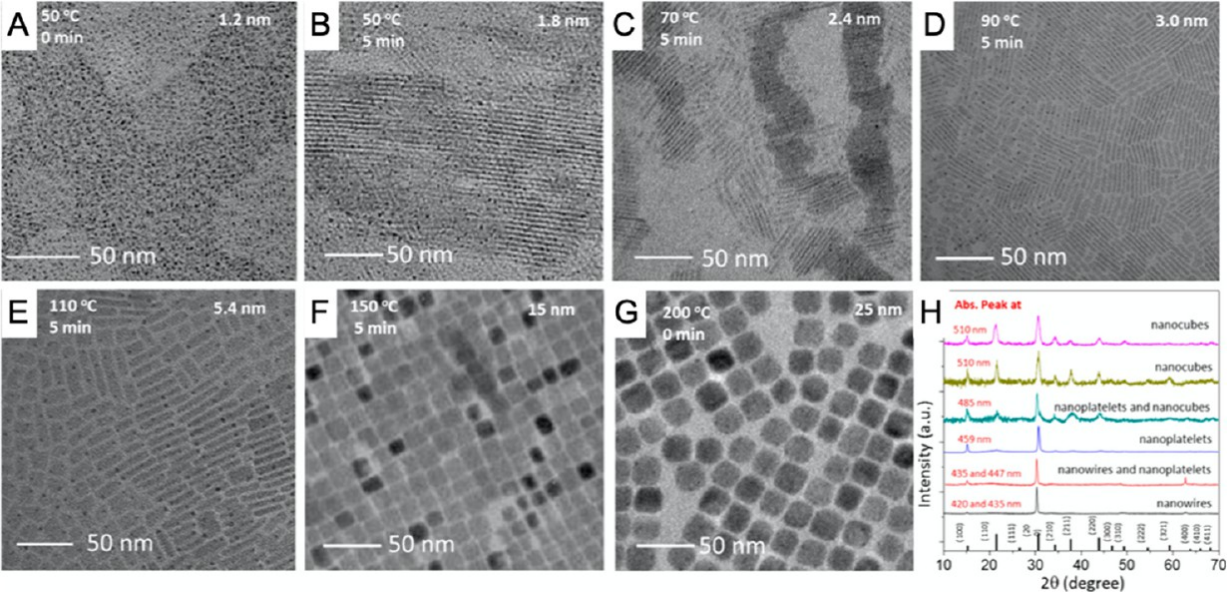
Shape transformations occurring during CsPbBr_3_ NC synthesis for different temperatures and reaction times. (A–G) TEM images. (H) X-ray diffraction data. Reproduced from ref (316). Copyright 2018 American Chemical Society.

Post-synthetic shape transformations provide access to colloidal NCs that are difficult to obtain by direct synthesis. In addition, they help to understand the growth mechanism and the properties of the corresponding NCs. Attempts to improve the properties of nanocube-shaped perovskite NCs by post-synthesis annealing revealed that such heat treatments can lead to changes in the NC shape and size. Yuan *et al.*(315) observed a red shift of the photoluminescence wavelength accompanied by a degradation in intensity upon thermal annealing under vacuum at a temperature of 400 K. TEM imaging revealed an increase in NC size of up to a factor of 2 to 3, and compositional analysis showed that the Pb/Br ratio decreased, thus pointing to more Br-rich surfaces after annealing. The impact of the temperature on the NC growth and shape transformations was elucidated in detail by Pradhan and co-workers (Figure 29).^316^ By stepwise increasing the temperature in their reactions, they demonstrated highly accurate size control and observed shape transformations from thin nanowires to nanoplatelets in the early stages of the reaction that evolved into nanocubes with dimensions up to 25 nm for longer reaction times. Tong *et al.*(22) found that CsPbBr_3_ nanocubes could gradually transform into nanowires through an oriented attachment mechanism under specific reaction conditions. A similar shape transformation was reported by Sun *et al.*,^186^ who showed that cubic crystalline CsPbI_3_ nanocubes transform into nanowires upon their treatment with polar solvents. The authors attributed this to polar solvent induced lattice distortions in cubic crystalline CsPbI_3_ nanocubes, followed by dipole-moment-triggered self-assembly into single-crystalline NWs. Similarly, Pradhan *et al.*(317) showed that post-synthetic aging of colloidal solutions leads to the transformation of CsPb(Br_*x*_I_1–*x*_)_3_ into the corresponding NWs with length up to several micrometers. Such shape transformation can also be triggered by halide-vacancy-driven, ligand-directed self-assembly process, as demonstrated by Bakr and co-workers.^318^ They have shown that the halide vacancy CsPbBr_3_ nanocubes transform into millimeter-long NWs upon ligand exchange with didodecyldimethylammonium sulfide (DDAS).

**Figure 30:**
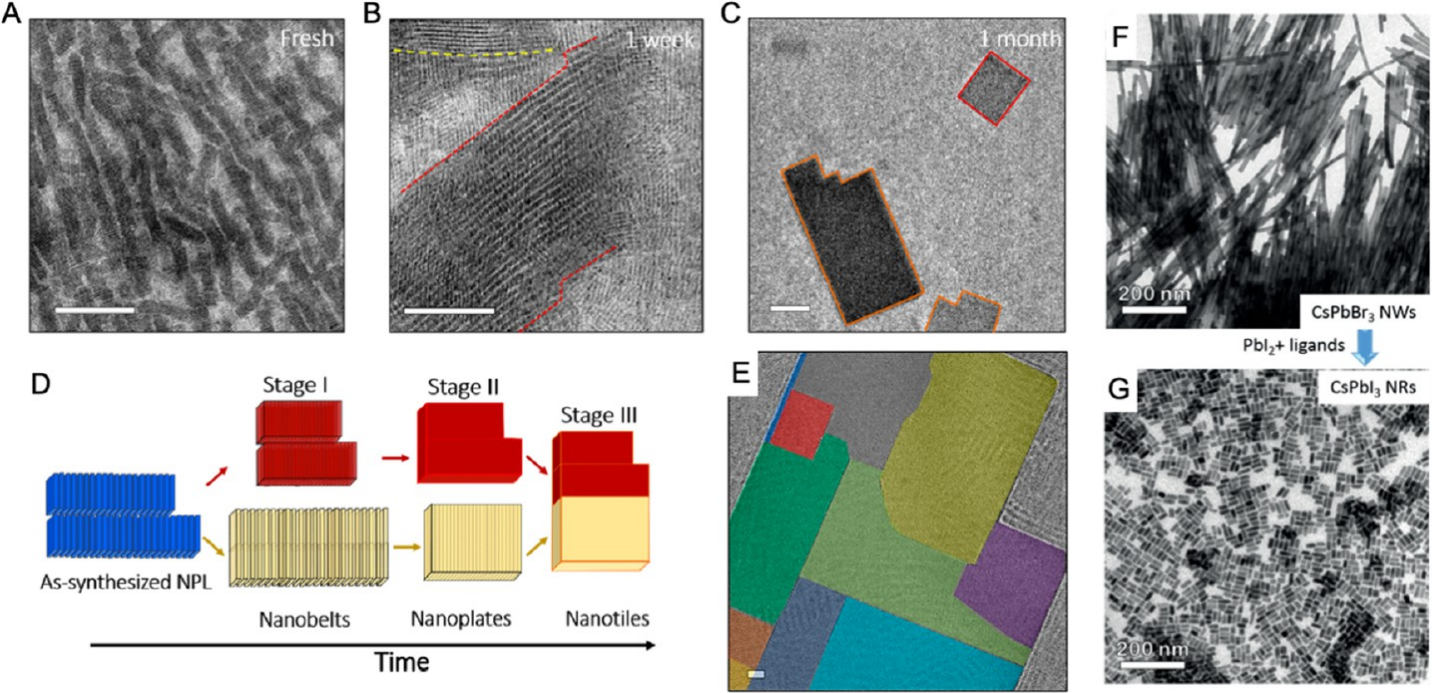
Temperature and chemically induced transformations in shape of CsPbBr_3_ NC structures. (A–C) TEM images recorded from aliquot taken from CsPbBr_3_ nanoplatelet solution at different times. (D) Illustration of the oriented assembly process and transformation from nanoplatelets to nanobelts, nanoplates, and nanotiles. (E) False-colored TEM image of a nanotile. Panels A–E are reproduced under Creative Common CC-BY 4.0 license from ref (319). Copyright 2020 American Chemical Society. (F,G) Chemical cutting of CsPbBr_3_ NWs into CsPbI_3_ nanorods. Reproduced with permission from ref (73). Copyright 2018 John Wiley & Sons, Inc.

The evolution of CsPbBr_3_ nanoplatelets into nanobelts, nanoplates, and nanotiles over time in solution and in films was investigated in detail by Dang *et al.*(319) (Figure 30A–E), who evidenced the formation of nearly defect-free nanobelts at the early stage by oriented attachment and fusion of the nanoplatelets, while at later stages, the nanobelts and nanoplates assembled into mosaic-like nanotiles. The interfaces in such nanotiles were characterized by Ruddlesden–Popper stacking faults due to the presence of CsBr bilayers. This transformation, which occurred in solution at room temperature over several weeks, was also observed in thin NC films and could be accelerated to time frames of less than 1 h by increasing the temperature. Around the same time, Pradhan and co-workers reported a similar shape transformation on a TEM grid at RT.^320^ They found that the polyhedral nanocubes transform into either zigzag-shaped 1D nanostructures by oriented attachment of corners or nanotiles by sidewise fusion, depending on their composition. Interestingly, these transformations could be ceased at any point of time either by applying heat or by adding sufficient ligands. A similar transformation had been reported earlier by Shamsi *et al.*,^234^ who found that the exposure of CsPbBr_3_ nanoplatelet films to intense ultraviolet light led to the transformation into nanobelts. Since the initial nanoplatelets were blue emitting due to quantum-confinement effects, while the larger nanobelts emitted green light, the use of shadow masks in such transformation could lead to color patterned films. The high brightness and stability of the films that were exposed to ultraviolet light enabled the fabrication of solution-processed light-emitting diodes. Such light-induced shape transformations strongly depend on the type of surface ligands. Li *et al.*(321) showed that individual CsPbBr_3_ perovskite NCs capped with 1-alkynyl acids could readily transform either into large cuboid- or peanut-shaped microcrystals under UV irradiation. The shape of the resultant microcrystals depend on the chain length of the 1-alkynyl acid used as surface ligand. The authors proposed that the shape transformation was caused by self-assembly of CsPbBr_3_ nanocubes through ligand-induced homocoupling of surface ligands. In addition, the transformation of nanocubes to nanoplates has also achieved by applying pressure in the GPa range (in a diamond anvil cell) to superlattices of CsPbBr_3_ nanocubes.^322^ The pressure treatment led to the formation of nanoplates with edge lengths that were 2–3 times larger than those of the initial nanocubes and to a blue-shifted emission after pressure release, pointing to quantum confinement in the out-of-plane direction.

Transformations *via* fragmentation of perovskite NCs, instead of assembly, is another possible mechanism. Tong *et al.*(73) demonstrated the chemical cutting of CsPbBr_3_ by a ligand induced fragmentation into CsPbX_3_ nanorods (X = Cl, Br, I) that was triggered by a halide anion exchange reaction (Figure 30F,G). The emission of the resulting perovskite nanorods could be tuned across the visible range, and photon antibunching experiments revealed single-photon emission from such nanorods. Other ligand-induced post-synthesis transformations include the evolution of CsPbBr_3_ nanocubes to NWs and 0D structures, or to nanoplates.^323^ In this latter work, the transformation could be controlled by the choice of the ligands: alkyl carboxylic acids lead to emitting nanoplates, while oleylamine and octylamine initiated the formation of NWs and 0D structures. On the other hand, shape transformations have been rarely reported for OIHP NCs. For instance, Tong *et al.*(231) demonstrated the ligand-induced transformation of 3D nanocubes into 2D NPls upon dilution of colloidal solution. They showed that the thickness of the NPls is tunable by both the ligand concentration as well as the dilution level. In addition, nanoplatelets could be obtained by bottom-up shape transformation of spherical nanodots, as reported by Liu *et al.*(324) They showed that the nanodots obtained by LARP gradually transform into square shape NPls upon aging the nanodot solution for 3 days. They attributed this transformation to dipole–dipole interactions along with realignment of dipolar vectors of nanodots.

### Summary and Outlook of Shape and Composition-Controlled Synthesis of LHP NCs

Numerous methods have been reported for the shape-controlled synthesis of both OIH and inorganic colloidal LHP NCs. Most of the reported methods generally yield either nanocubes, nanoplatelets, or nanowires. Recent studies have demonstrated the synthesis of noncubic LHP NCs at relatively high reaction temperature.^70,71^ However, these methods are yet to be standardized for the routine synthesis of noncubic LHP NCs. The shape of the LHP NCs is controllable from nanocubes to NPl of different thicknesses by varying several parameters, such as reaction temperature,^18^ precursor ratio,^60^ long-chain to short-chain ligands ratio,^16^ and acid–base equilibrium of ligands.^145^ In general, lower reaction temperatures lead to anisotropic growth of NCs, and this results in the formation of LHP NPl at reaction temperatures below 100 °C, and the thickness of NPls decreases with decreasing the reaction temperature.^18^ On the other hand, LHP nanocubes transform into nanowires under prolonged reaction times in both the HI synthesis and the ultrasonication-assisted synthesis.^22,75^ The transformation of nanocubes into nanowires occurs through an oriented attachment mechanism.^22,186,318^ Furthermore, the thickness of the nanowires is tunable down to the strong quantum-confinement regime using short-chain ligands.^74,76^ In addition, shape control is achieved through post-synthetic transformations. For example, it was shown that NPls could be transformed into nanosheets,^319^ and nanowires could be transformed into nanorods.^73^ Despite significant advances in the synthesis of LHP NCs, their growth mechanism is still not well-understood due to the fast nucleation and growth processes, which are therefore hard to follow. A better understanding of their growth mechanism is critical for further advancing the synthesis of LHP NCs of desired shapes through controlled growth rate and directionality using specific ligands. The optical band gap of LPH NCs mainly depends on the extent of the quantum confinement that the NCs exhibit, and this is discussed in detail in the optical properties section (see OPTICAL PROPERTIES). The optical properties of LHP NCs are easily tunable across the visible spectrum of light by halide (Cl, Br, and I) composition, and they can be prepared either by direct synthesis or by applying halide ion exchange reactions. The distinctive feature of LHP NCs is that the halide ion exchange is spontaneous and reversible, and it takes place at room temperature. This means LHP NCs with any halide composition can be easily achieved using presynthesized LHP NCs made of any one of the halide types. For some applications, such a spontaneous halide exchange can be problematic. However, the halide exchange could be suppressed by coating LHP NCs with lead sulfates.^296^ In addition, good progress has been made regarding the cation (A- and B-site) exchange of LHP NCs for enhancement of their stability, for the sake of introducing additional optical properties and replacing Pb with nontoxic metal ions.

## Surface Chemistry of Colloidal Halide Perovskite NCs

With the decrease of particle size down to several nanometers, the fraction of surface atoms in NCs can be higher than 30%. The incomplete coordination of surface atoms usually contributes to the appearance of defect energy levels in the band gap that behave as exciton traps and leads to nonradiative recombination.^325,326^ Therefore, in past decades, researchers from the field of II–VI semiconductor NCs (mainly the cadmium-based NCs) have made great efforts to solve this problem. Finally, PLQY of 100% and perfect monoexponential PL decay were achieved by an elaborate design of synthesis procedures and shell structures.^327,328^ However, lead-halide perovskite NCs with a high QY (∼100%) can be prepared directly and easily even without shells.^14,329,330^ This phenomenon is related to the high defect tolerance of these materials.^98,331^ Theoretical calculations have suggested that the defects with low formation energies are the ones that contribute to shallow states. A detailed discussion on defect tolerance and the distinctive emission properties of lead-halide perovskite NCs is provided in the optical properties section.

After several years of research, it was found that the surface defects, especially the halide vacancies (V_X_), still make great contributions to nonradiative recombination. Then, various passivation strategies were developed to enable a PLQY for perovskite NCs close to 100%. As a whole, these passivation approaches can be divided into two types: post-synthesis passivation and *in situ* passivation, that is, during the synthesis (see below). It should be noted that crystal defects in NCs can be eliminated by a self-purification mechanism.^332^

### Surface Ligands

Lead-halide perovskite nanomaterials typically consist of an all-inorganic or organic–inorganic core, such as CsPbX_3_ and CH_3_NH_3_PbX_3_ (X = Cl, Br, I) NCs, capped with organic ligands, and we will refer to them as LHP@capping NCs. The interest of focusing on surface chemistry of LHP NCs is to better understand the interaction between the ligand anchoring group(s) and the NC surface in LHP@capping NCs with a view to finding the most suitable ligands for surface passivation, thereby manifesting the best of the distinctive properties of the perovskite, thus enhancing their applicability.

Ligands play a crucial role during the synthesis of the NCs, such as in the kinetics of the crystal growth and in regulating the final NC size and shape.^52,333^ In addition, the capping ligands can be designed to prevent the agglomeration of the NCs and determine the extent of the NC–solvent interaction and, consequently, their dispersibility in the medium.^52,333,113^ However, the high dynamic bonding between the NC surface and the capping ligands is at the origin of the chemical instability of LHP@capping NCs; this has become patent during the purification of these nanomaterials.^52,177^ Therefore, enhancing the strength of the ligand coordination to the NC surface can have a positive impact on the colloidal and chemical stability of the NCs and, consequently, on the conservation of their optical properties. Nevertheless, another important feature of the ligand that has to be taken into account is its electrical conductivity, as efficient charge carrier transport is required in NC thin-film-based optoelectronic devices. Lately, this matter has attracted a great deal of interest.^334,335^

Techniques to visualize the dissociation of the ligands from the NC surface, as well as the nature of the ligand anchoring group, are providing relevant information to find the most adequate ligand, or combination of ligands, to exploit the distinctive properties of these materials at the nanoscale.^84,336^ The combination of spectroscopic techniques, such as NMR, FTIR, and XPS, are useful to determine the eventual ligand(s) on the NC surface and the nature of the ligand anchoring group(s). In addition, the combination of NMR spectroscopy and thermogravimetric analysis (TGA) is a suitable strategy to study the composition of LHP@capping.^25^ The ionic nature of these NCs makes them revert back to the NC precursors in a polar solvent, such as deuterated DMSO, thus making it possible to know the structure of the ligand(s) bonded to the surface and to determine the ratio between the LHP@capping components easily by ^1^H NMR.^25^ In addition, the combination of NMR, nuclear Overhauser effect spectroscopy (NOESY), and diffusion-ordered spectroscopy (DOSY) makes it possible to determine if the organic ligand is loosely or tightly bound at the NC surface.^84^ Moreover, the NMR line broadening technique is also of interest for surface chemistry analysis and has been related to poor ligand solvation, a feature of bound ligands.^337^ The broad line has a homogeneous and a heterogeneous component. Solvation of the ligand shell contributes mainly to the heterogeneous line broadening, as was confirmed by dynamic simulations, while the homogeneous contribution depends on the NC size (the bigger the size, the broader the line).^338^ Despite significant understanding of the ligand–NC interaction over the last few years, there are still some issues to be overcome to improve the potential of colloidal perovskite NCs in different technologies. Several contributions will be discussed below.

### Passivation of Surface Defects with Ligands

The type of ligand binding to the surface of common semiconductor NCs has been analyzed using the covalent bond classification introduced by Green *et al.* for organometallic compounds.^339,340^ In this model, the covalent bond of any element is classified according to the total number of electrons involved in the primary bonding in the valence shell of the element (M) and the number of electrons the ligand used to form the bond.

Three types of binding ligands were reported: (a) X-type, which involves a single occupied orbital of the ligand anchoring group and one electron from M (the ligands are neutral species that are radicals, such as H, COR, CR_3_, C_6_H_5_, CN, OCN, ONO; X ligands can derive from anionic precursors, such as halides, hydroxide, alkoxide alkyl species that are one-electron as neutral ligands, but two electron donors as anionic ligands); (b) L-type, which involves an orbital of the ligand filled with two electrons and acts as a donor to the empty orbital of M (the ligands are neutral molecules that are Lewis bases, such as NH_3_, NR_3_, OH_2_, OR_2_, PR_3_, SR_3_); and (c) Z-type, whose anchoring group orbital is empty and can accept an electron pair from M (the ligands are neutral molecules that are Lewis acids, such as BH_3_, BF_3_, BCl_3_).^339,340^

**Scheme 1:**
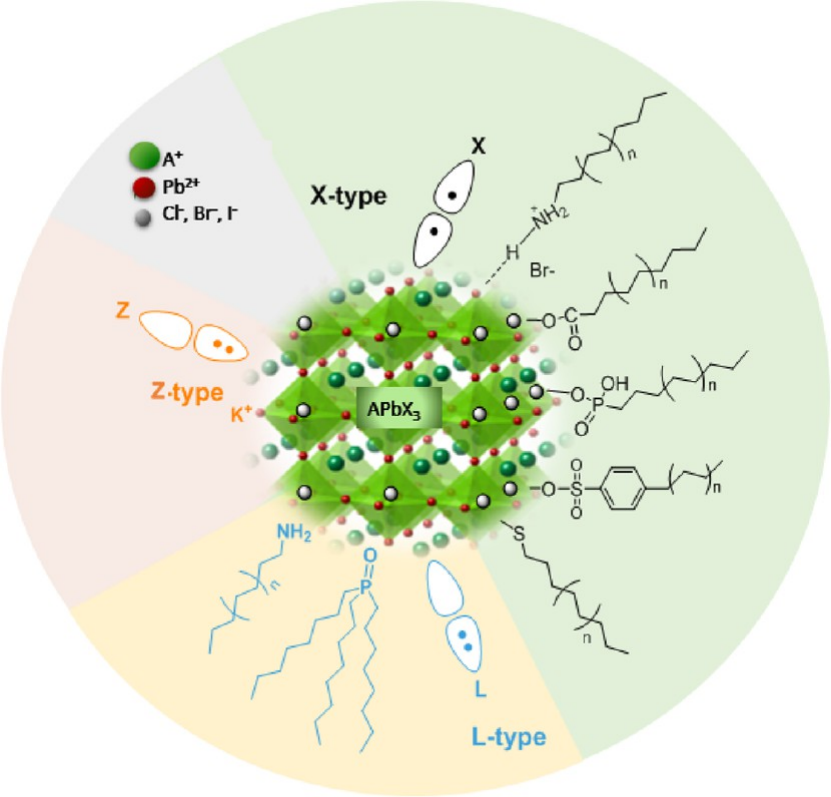
Binding Ligands (X, L, and Z-Type, According to the Covalent Bond Classification) Used as Capping Agents of Colloidal APbX_3_ Perovskite NCs

Regarding the type of ligands in LHP@capping NCs, the most common ligands used are of X- and L-type (see Scheme 1). The binding of ligands to the surface of these NCs is usually highly dynamic and therefore ligands can be lost during the isolation and the subsequent purification steps. Highly emissive LHP@capping NCs are the consequence of an efficient passivation of their surface defects with ligands that anchor to the NC surface with a high binding constant, which are mainly of the X- and L-types, thus providing colloidal and chemical stability. The binding mechanism of the ligands ranked by the covalent bond classification can be summarized as (i) X-type ligand: covalent bond created after one electron donation from the halide anion to the ammonium, or from the carboxylate, phosphate, sulfonate, or thiol/thiolate to the perovskite cations (Pb^2+^, A^+^), or between the charged groups of a zwitterionic molecule and X^–^ and Pb^2+^; (ii) L-type ligand: dative covalent bond created by sharing a lone electron pair from the ligand with the metal center; and (iii) Z-type ligand: dative covalent bond by sharing a lone electron pair from the halide with a Lewis acid, such as the intraction between K^+^ and X^–^.

In 2012, Papavassiliou *et al*. described the preparation of nanocrystalline/microcrystalline materials based on Pb(Br_*x*_Cl_1–*x*_)_3_, Pb(Br_*x*_I_1–*x*_)_3_, Pb(Cl_*x*_I_1–*x*_)_3_ units with *x* = 0–1, which exhibit tunable emission from 400 to 700 nm, from the corresponding quasi-two-dimensional compounds.^64^ Suspensions based on lead bromides materials were obtained using a titration-like method, in which the solutions of (CH_3_NH_3_)(CH_3_C_6_H_4_CH_2_NH_3_)_2_Pb_2_Br_7_, (CH_3_NH_3_)(C_4_H_9_NH_3_)_2_Pb_2_Br_7_, or their precursors in dimethylformamide were injected into toluene or toluene-containing PMMA, at room temperature. The crystalline particles presented sizes ranging between 30 and 160 nm and green emission in the 531–510 nm range with PL quantum yield from 0.13 to 16%. These values were improved up to 25% using (CH_3_NH_3_)(C_4_H_9_NH_3_)_2_Pb_2_Br_7_ as the precursor. The particles prepared in a PMMA matrix increased their stability upon aging for at least 1 year compared with a few hours for the suspension in toluene.

Schmidt *et al*.^66^ reported the preparation of colloidal hybrid perovskite NCs using a nontemplate strategy consisting of adding a mixture of a long-chain ammonium bromide, such as octylammonium bromide (OLABr) and methylammonium bromide (MABr), to an 80 °C solution of oleic acid in ODE, followed by the consecutive addition of PbBr_2_, and immediately afterward, the addition of acetone to induce the crystallization of the perovskite (yellow solid) with a PLQY of 20% in toluene. The electroluminescence (EL) of a thin-film light-emitting device prepared with these colloidal hybrid perovskite NCs showed a noticeable improvement compared with that of bulk film, thus evidencing their potential for optoelectronic applications. A year later, it was demonstrated that more emissive and stable colloidal MAPbBr_3_ NCs (PLQY of 83%) can be obtained in the absence of OA. ^1^H NMR studies of the NCs, by reverting the perovskite back to its precursors in deuterated DMSO, combined with TGA, made it possible to determine the presence of OLABr (X_2_ ligand) on the NC surface, as well as the composition of the nanomaterial (NC plus ligand).^25^ The N 1s XPS spectrum of the NC showed only a band with maximum at 402.6 eV, thus corroborating the presence of alkylammonium to passivate the under-coordinated bromide of the NC surface. Then, bright lead bromide perovskite NCs (PLQY of about 100%) were prepared by following the LARP technique (see below), using the quasi-spherical-shaped 2-adamantylammonium bromide as the only capping ligand.^341^ Though extraordinarily luminescent, these NCs showed a trend to aggregate due to the high interaction between the adamantyl moieties; in fact, they exhibited an average lifetime on the microsecond scale. The high affinity of the adamantyl moiety for the cavity of cucurbit[7]uril enabled the preparation of perovskite NCs with a host–guest complex as capping ligand, which showed a higher photostability under contact with water than the NC passivated with 2-adamantylammonium bromide.^341^ Among various ligands, primary amine/carboxylic acid ligand pairs became the most commonly used pairs of organic ligands for the synthesis of bright colloidal perovskite NCs.^14,141^ The LARP strategy, introduced by Zhang *et al.*,^29^ consisted of a dropwise addition of the capping ligands (octylamine and oleic acid) and the MAPbBr_3_ perovskite precursor solutions into a low polar solvent, followed by centrifugation at room temperature to remove bulk material. The PLQY of MAPbBr_3_ was high and well-preserved after purification (PLQY ∼80%). Similarly, Protesescu *et al.* prepared highly luminescent and monodispersed colloidal CsPbBr_3_ (PLQY of 90%) by a hot-injection methodology using oleylamine and oleic acid as organic ligands.^14^

Table 1 shows the chemical structure of the organic/inorganic ligands mentioned in this section, including acids, such as alkylcarboxylic, alkylphosphonic, alkylsulfonic and alkylphosphonic acids, alkylamines, alkylammonium salts, alkylthiols, and zwitterionic species. De Roo *et al*.^84^ performed ^1^H NMR spectroscopic studies to determine the eventual ligand(s) at the NC surface and also to gain insight into the surface chemistry of CsPbBr_3_ NCs synthesized using oleylamine, oleic acid, a Cs-oleate solution, octadecene, and PbBr_2_. NOESY experiments demonstrated that octadecene and oleic acid did not bind to the NC surface, while oleylammonium bromide was proposed as the capping ligand. It was suggested that the oleylammonium cation might have bound to the surface bromide atoms *via* a hydrogen bridge and the bromide anion might have bound to cesium or lead atoms located on the surface, in agreement with the ionic character of the CsPbBr_3_ NCs.^337^ However, the data were not conclusive as to whether the NCs were stabilized by oleylammonium bromide or oleylammonium oleate, both with a pair of X-type ligands, which corresponds to an NC(X)_2_ binding motif. Three possible combinations of these ligands were then proposed: oleylammonium bromide, oleylammonium oleate, and the unprotonated amine (L-type ligand). As a consequence of the fast exchange between the ligands, it was difficult to determine their individual contribution on the surface of the NCs. The addition of small amounts of excess oleic acid and oleylamine before precipitation preserved the colloidal integrity and PL of the NCs. They corroborated the presence of a tightly bound fraction of oleic acid by means of NMR spectroscopy using dodecylamine/oleic acid as the ligand pair.

**Table 1:**
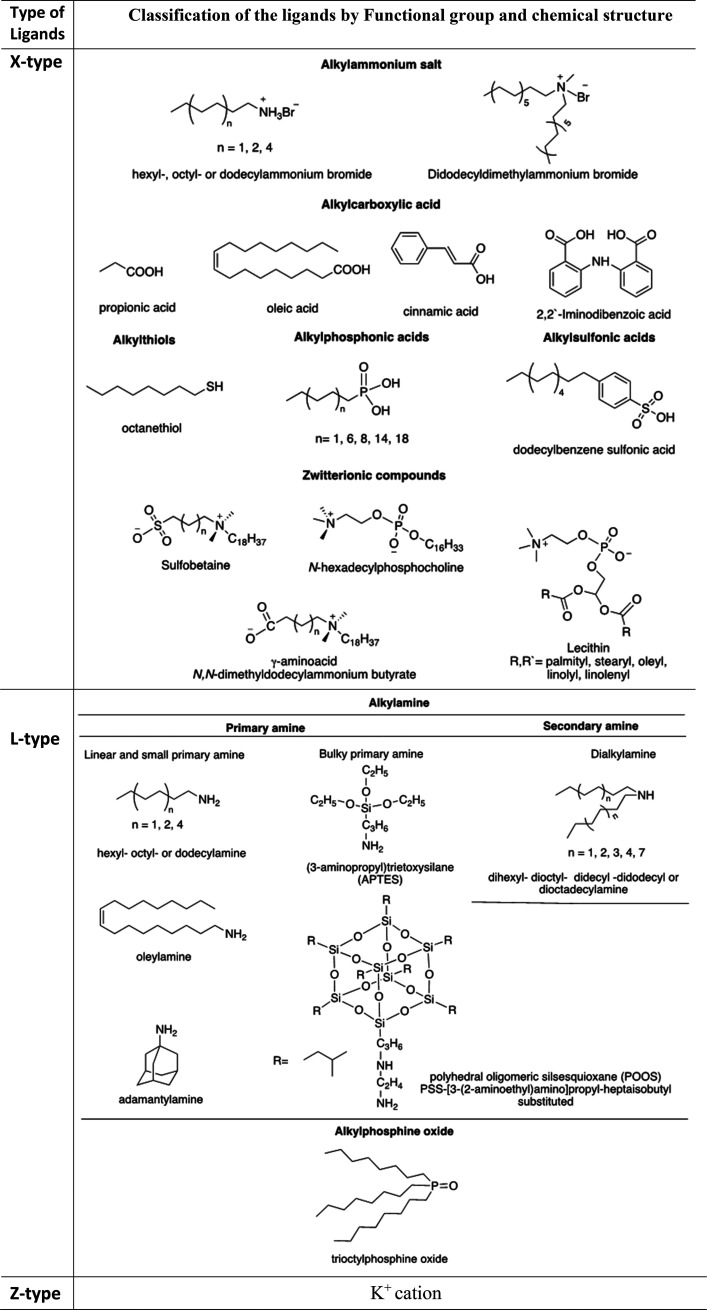
Chemical Structure of Organic/Inorganic Ligands Used To Prepare Colloidal LHP NCs, Categorized According to the Functional Group and Covalent Bond Classification

It was reasoned that oleic acid cannot bind by itself, but it binds as an ion pair with amine, the actual tightly bound ligand pair being oleylammonium oleate. Huang *et al.*(342) have suggested that oleylamine (i) acts as an L-type coordinating agent binding to Pb^2+^ to form a Pb^2+^–oleylamine complex and (ii) reacts with oleic acid to form the oleylammonium oleate salt, and then oleate coordinates to Pb^2+^ due to the high coordination number of the metal cation (between 2 and 10).^343^ Consequently, the N 1s XPS spectrum showed two peaks, at 398.6 and 400 eV, which can be ascribed to the oleylamine and methylammonium/oleylammonium, respectively, while the O 1s XPS showed two peaks at 532.3 and 533.7 eV, which can be attributed to two non-equivalent oxygen atoms of carboxylic acid and to the two chemically equivalent oxygen atoms of oleate, respectively.

González-Carrero *et al.* combined a short primary amine and a short carboxylic acid, such as 2-adamantylamine and propanoic acid, as ligand pairs to produce highly photoluminescent (PLQY ∼100%) colloidal CH_3_NH_3_PbBr_3_ perovskites.^330^ The N 1s XPS spectrum deconvoluted into two peaks centered at 399.8 and 401.5 eV with an area ratio of 0.3; these peaks can be ascribed to 2-adamantylamine and the methylammonium salt, respectively. Both O 1s and C 1s XPS spectra confirmed the presence of carboxylic acid and carboxylate species. The quantification of the perovskite components by XPS showed an atomic ratio of 2.7 and 1.1 for Br/Pb and N/Pb, respectively. This can be considered as a presence of bromide vacancies (V_Br_) in the perovskite more than an excess of lead atoms, as was observed by other researchers.^194^ These LHP@capping nanomaterials showed a low tendency to aggregate in solution due to the reduction of the ligand–ligand interaction between the NCs while preserving the high QY. Those NCs assembled in solid films with thicknesses of hundreds of nanometers also retained a high PLQY, specifically ∼80%.^330^

Primary amines with a branched structure have been used as an L-type ligand, leading to perovskites with a low PLQY. Examples of this type of ligands^344^ are (3-aminopropyl)triethoxysilane (APTES) and polyhedral oligomeric silsesquioxane (POSS) PSS-[3-(2-aminoethyl)amino]propylheptaisobutyl substituted, which have enabled a good control over the size of CH_3_NH_3_PbBr_3_ NCs. Their low PLQY of <20% has been attributed to an inadequate passivation of the nanoparticle surface due to the steric effect of the branched ligands. In addition, CH_3_NH_3_PbBr_3_ NCs have been passivated with a commercial cyclic peptide cyclo(RGDFK), containing five amino acids (arginine, glycine, aspartic acid, phenylalanine, and lysine).^345^ Modeling of PbBr_3_^–^/cyclo(RGDFK) precursor complexes suggested the preferential coordination of the peptide to the PbBr_3_^–^*via* the amine *versus* the guanidine group, which is consistent with the broadening of the −NH_3_^+^ moiety peak (3200 cm^–1^) in the FTIR spectrum of the complex. The low PLQY (∼20%) of the perovskite NCs passivated with cyclo(RGDFK) has been ascribed to charge transfer from the perovskite core to the peptide shell.

Secondary amines of different length, such as dihexyl-, dioctyl-, didecyl-, didodecyl-, and dioctadecylamine, have been used to prepare, in combination with oleic acid, CsPbBr_3_ nanocubes with good emissive properties (48–80%) and a uniform cubic shape that allows their self-assembly in 50-μm-sized superlattices.^143^ Interestingly, the pure-shaped NCs were obtained irrespectively of the length of the amine, oleic acid concentration and temperature. Density functional theory (DFT) calculations suggested that the binding of the dialkylammonium molecules to the [100] facets of CsPbBr_3_ is weak and secondary to that of oleate; otherwise, it would cause a drastic distortion to the lattice.^143^

Different capping agents, such as acids (oleic, phosphonic and sulfonic acids) and thiols, have been proposed to avoid the labile binding of amines (L-type ligand), ammonium/halide, and ammonium/oleate pairs (X_2_-type ligands) to the NC surface. There is some controversy regarding the performance of oleic acid as the CsPbX_3_ NC surface ligand. Yassitepe *et al.* developed an amine-free method to prepare CsPbX_3_ NCs passivated by only oleic acid,^346^ which exhibits strong interaction with the surface, and as a result, the NCs can be washed several times. However, oleic acid does not seem to be a good candidate to provide CsPbX_3_ NCs with a high PLQY. By contrast, Lu *et al.* built colloidal CsPbBr_3_ with oleate as the only ligand (X-type ligand) and produced nanocubes of 11.2 nm with a PLQY of 70%. They showed a colloidal stability over at least 2 months, which is considerably higher than that reported for LHP@amine-oleate-passivated NCs.^347^^1^H NMR spectroscopy corroborated oleate as the surface ligand, which was then effectively replaced by cinnamic acid derivatives, namely, *trans-*cinnamate and *trans*-3,5-difluorocinnamate, as demonstrated by FTIR spectra of the NCs (quantitative removal of the native oleate), as well as by ^19^F NMR and XPS measurements of the difluoro compound (observation of a broad signal and the presence of F signals, respectively). The easy replacement enabled the tuning of the NC optical/electronic properties but decreased its PLQY. Interestingly, LHP@cinnamate NCs showed enhanced photocatalytic activity for α-alkylation of aldehydes. The positive effects of the ligands in terms of the NC photocatalytic response might be due to (a) an increase of the NC photoredox potential, (b) a change in the ligand shell permeability, and (c) a good passivation of the surface defects, thus increasing the lifetime of the photocarriers and/or reducing surface catalytic sites.^347^ Another important factor can be the synergy between the NC surface and its organic ligand to lead to a high substrate preconcentration near the NC surface (for further details, see section “Photocatalysis Using Perovskite NCs”).^348^ More studies are required to determine the contribution of these factors on the performance of the NCs in photocatalysis.

The combination of trioctylphosphine and oleic acid has been used to prepare nanocubes of CsPbBr_3_ NCs (PLQY of ∼60%) with oleate as the only capping ligand;^179^ this synthetic route can be extended to *n*-tetradecylphosphonic acid and diisoctylphosphonic acid. ^31^P NMR spectroscopic studies were performed to determine the role of trioctylphosphine and oleic acid in PbBr_2_ solubility; these studies indicate a competing interaction between the protic acid and PbBr_2_ for the oxygen of TOPO. ^1^H NMR studies give information on the dynamics of the Cs-oleate capping agent by focusing on the broadening and shift of the signals compared to those of the free acid. Negative cross-peaks in the NOESY spectrum corresponded to species with long correlation times with a movement that was slower in solution compared to small free molecules. A diffusion coefficient of 242 μm^2^/s, calculated by DOSY spectroscopy, was highly reduced compared to 725 μm^2^/s in the free acid and corresponded to a 76% of bound oleate species on the NC surface.^179^

Sulfur-containing X-type ligands, alkylthiol and thiocyanates, were proposed to replace the oleylamine/oleic acid pair of ligands and to act as better passivation agents by reducing the surface defects and leading to NCs with a monoexponential PL lifetime and a better PLQY.^349^ A combination of alkylthiols with alkylamines or alkyl acids was used to control the crystal structure from orthorhombic CsPbBr_3_ toward tetragonal CsPb_2_Br_5_ nanowires and nanosheets, which exhibited a high stability at high temperature and under humid conditions.

**Figure 31:**
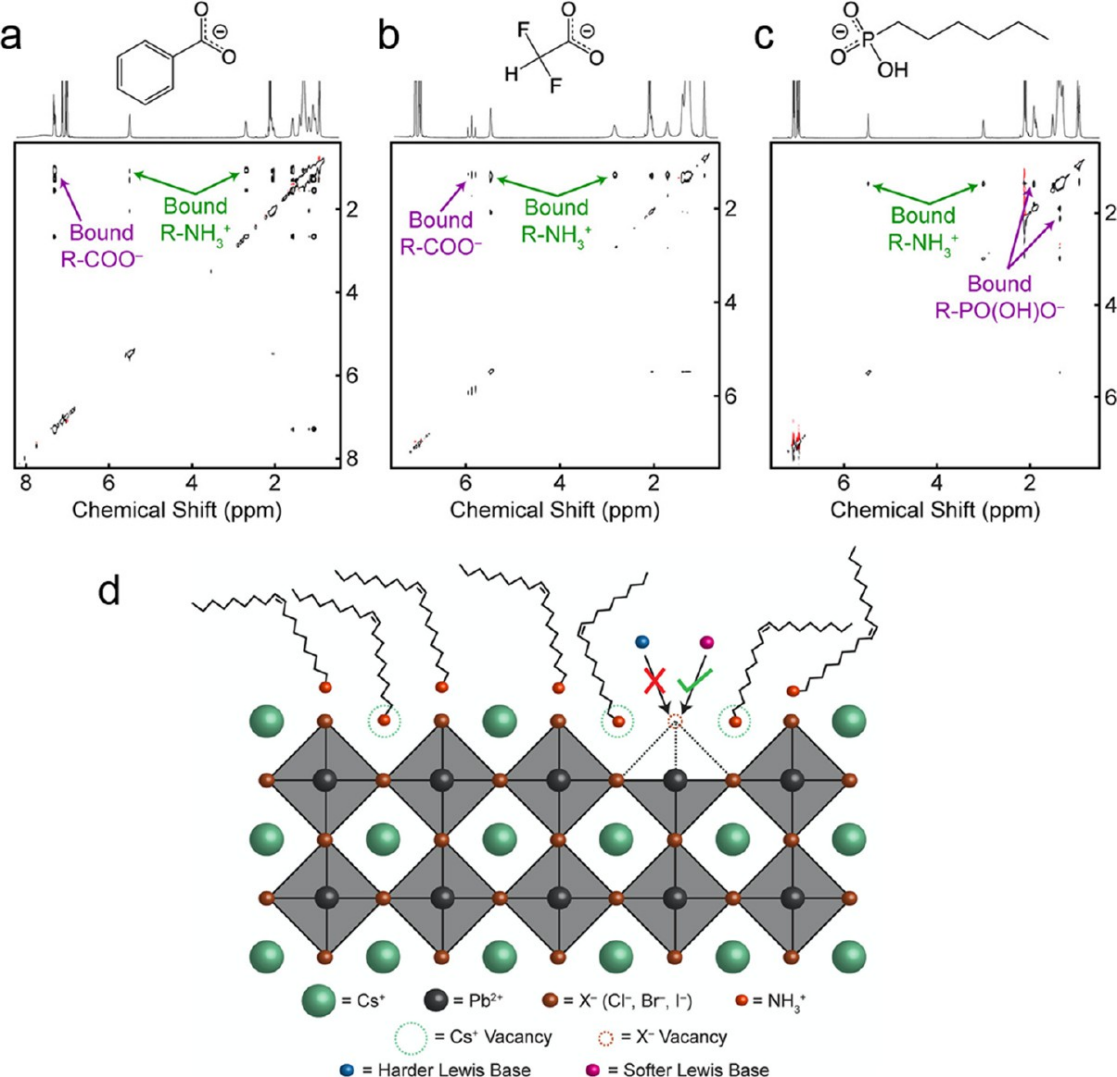
^1^H NOESY NMR spectra of CsPbBr_3_ NC samples exchanged to ligand pairs of oleylammonium and (a) benzoate, (b) difluoroacetate, and (c) hexylphosphonate. All ligand pairs feature negative (black) NOE signals rather than positive (red) NOE signals, thereby corroborating their interaction with the NC surface. (d) Schematic representation of a cesium- and halide-deficient surface terminated by CsX facets, consistent with experimental results. Reproduced from ref (194). Copyright 2018 American Chemical Society.

An exhaustive and systematic surface chemistry study was reported by Alivisatos *et al*. in 2018 to draw together the observations from several reports on this subject.^194^ A methodology to obtain trap-free lead-halide NCs was proposed based on the combination of different techniques, such as NMR, NOESY and fluorescence spectroscopies, with *ab initio* calculations that evidenced that a soft X-type ligand can properly passivate the uncoordinated lead atoms, created by the halide vacancies on the NC surface. A cesium vacancy on the surface can be replaced by the oleylammonium cation. The lower the NC concentration (high dilution), the greater number of surface V_X_, due to low binding of the oleylammonium halide pair. NMR line width was used to determine the number of trap states, related to V_X_, which combined with the PLQY gave the ratio between the radiative and nonradiative rate constant (*k*_r_/*k*_nr_). The *k*_r_/*k*_nr_ ratio value was related to the defect tolerance of the different halide perovskites (9500, 390, and 53 for CsPbI_3_, CsPbBr_3_, and CsPbCl_3_, respectively). Soft Lewis bases that can substitute halide vacancies and coordinate to lead (which is a relatively soft Lewis acid) can be a neutral molecule such as a pyridine and thiophene or an anionic X-type ligand, such as alkylphosphonate, S^2–^, benzoate, fluoroacetate, methanesulfonate, or trioctylphosphine. A ligand exchange strategy was used to introduce different alkyl carboxylates for the oleylammonium-R-COOH ligand pair, such as benzoate, fluoroacetate, and difluoroacetate. Nuclear Overhauser effect NMR spectroscopy was used to confirm the binding of the ligands to the NC surface supported by the negative cross peaks (Figure 31A,B). A good affinity of softer X-type ligands for the NC surface was also confirmed by the negative (black) NOE of oleylammonium hexylphosphonate (Figure 31C). These anionic ligands, X-type Lewis bases, could bind to cesium atoms on the surface, but this is not thermodynamically favorable,^350^ indicating they are binding to the surface lead atoms eliminating the V_X_. By contrast, hard Lewis X-type ligands, such as alkylcarboxylates, carbonates, and nitrates are inefficient passivating ligands (Figure 31D).

Alkylphosphonates as the only organic ligand were initially introduced by Xuan *et al.*(274) CsPbBr_3_ NCs passivated with 1-tetradecylphosphonate were prepared at room temperature with good emissive properties (PLQY of 68%) and extraordinary water and thermal stability using 1-tetradecylphosphonic acid. FTIR showed the replacement of the P=O band at 1230 cm^–1^, belonging to the 1-tetradecylphosphonic acid, by a broad band at 1000–900 cm^–1^, ascribed to Pb–O–P, thus corroborating the anchoring of the alkylphosphonate. Consequently, the O 1s XPS spectrum evidenced the presence of peaks at 530.8 and 530.2 eV corresponding to P–O–Pb and P–O bonds, respectively, confirming the FTIR analysis. Increasing the concentration of the phosphonic acid caused a blue shift in the absorption spectrum, which is consistent with the formation of smaller NCs due to a decrease in the rate of ligand release through the organic shell. The use of a phosphonic acid concentration higher than 7.5 mg mL^–1^ caused the decrease of NC PLQY, which can be associated with the steric hindrance resulting in a high number of uncoordinated surface atoms. Likewise, Zhang *et al.*(351) prepared colloidal CsPbBr_3_ NCs by employing alkyl phosphonic acids as the only surfactant. NMR analysis revealed the presence of both phosphonic acid anhydride and hydrogen phosphonate species on the NCs surface. Theoretical calculations indicated a high affinity of phosphonate ligands for the NC surface and similar stabilization energy of the [001] and [110] facets, thus resulting in the formation of NCs with a truncated octahedron shape that exhibited a nearly 100% PLQY.^351^ A follow-up of this work, by the same group reported the synthesis of CsPbBr_3_ NCs using custom-made oleylphosphonic acid (OLPA). The lower temperature at which OLPA was soluble in the reaction mixture, compared to phosphonic acids with linear chains, allowed the synthesis of NCs (at 100 °C) with sizes down to 5 nm. These NCs were also more colloidally stable upon exposure to air than those of ref (351), and again, this was traced back to the higher solubility of OLPA.

Alkylthiols were used to induce the transformation of CsPbBr_3_ NCs to CsPb_2_Br_5_ nanostructures, and CsPb_2_Br_5_ nanosheets and nanowires were obtained by controlling the ratio between alkylthiols and alkylamine or alkyl acids.^349^ The presence of thiols in the system increased the tolerance to a high temperature and a high humidity environment favored by the good affinity of sulfur to lead atoms. The strong affinity of thiols for the Pb^2+^ sites reduced considerably the density of surface defects, leading to a PLQY close to unity and a monoexponential PL decay kinetics.

**Figure 32:**
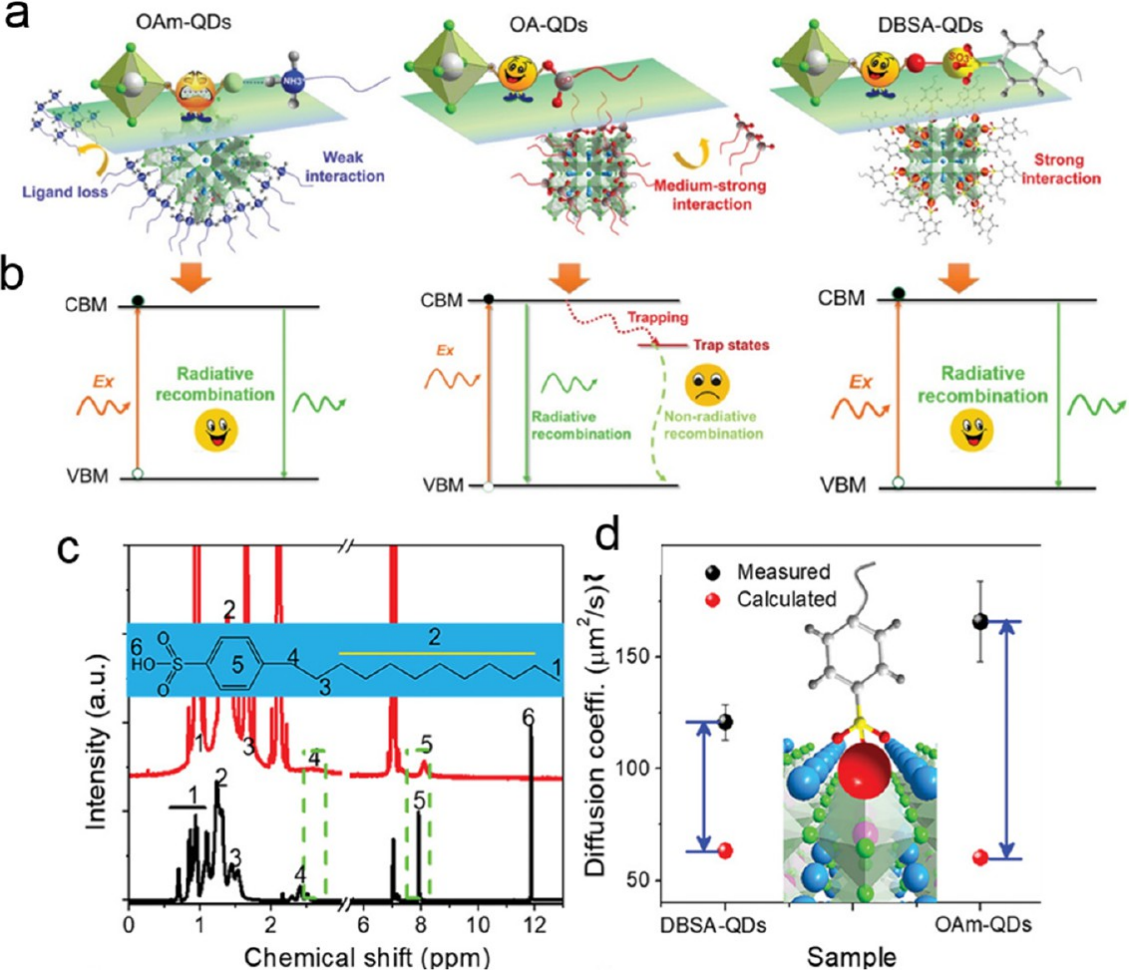
Comparison of different ligand strategies. (a) Binding motif on CsPbBr_3_ QD surface and interaction strength of oleylammonium, oleic acid, and dodecylbenzenesulfonic acid (DBSA) ligands. (b) Effect of different ligands on exciton recombination dynamics. Evidence of strong DBSA–QD interaction. (c) ^1^H NMR full spectra of pure DBSA and perovskite capped with DBSA after three purification cycles. (d) Diffusion coefficients of DBSA and perovskites capped with oleylammonium-capped NCs. Reproduced with permission from ref (352). Copyright 2019 John Wiley & Sons, Inc.

Long chain benzenesulfonic acid, such as dodecylbenzenesulfonic acid, was chosen as an excellent candidate to replace the bromide vacancy on the NC surface (Figure 32a).^352^ In order to eliminate the defect energy levels, ligands with anionic heads with electronic features similar to those of bromide ions should be favorable. A good interaction of alkylsulfonic acid with lead is expected as the calculated binding energy of 1.64 eV in sulfonate-Pb is comparable with 1.47 eV in CH_3_NH_3_Br–Pb. The interaction strength between the ligand and the NC surface was estimated by diffusion-ordered spectroscopy (Figure 32d); the registered diffusion coefficient was smaller than for oleylamine-capped NCs, which is consistent with a stronger interaction between the sulfonate and the NC surface. Such ligand interacts with lead atoms and eliminates the defect energy level successfully, leading to CsPbBr_3_ NCs with a PLQY higher than 90%. This binding was strong enough to resist a washing treatment, as shown by NMR (Figure 32c), keeping the PLQY up to 90% (Figure 32b). The high long-term colloidal stability and photostability under 400 nm irradiation of sulfonate-capped NCs compared to oleylammonium halide-capped NCs is a further evidence of the strong interaction between the sulfonate ligands and the NC surface, which makes these NCs appealing in thin-film technologies.^352^

Zwitterionic long-chain molecules, such as commercially available sulfobetaines, phosphocholines, and γ-amino acids, bind tightly to the CsPbBr_3_ surface due to the fact that (i) they can coordinate simultaneously to the surface cations and anions on the NC surface and (ii) the cationic and anionic groups of their structure cannot be neutralized. The presence of the zwitterionic ligand as the sole ligand at the NC surface was evidenced by complete ionic dissolution of purified NCs in deuterated DMSO, which freed the surface-bound ligands.^171^ In addition, DOSY NMR spectroscopy of the NCs evidenced that the diffusion coefficient related to the broad resonances (corresponding to the zwitterionic ligands anchored to the NC surface) was consistent with that estimated by the Stokes–Einstein equation (2 orders of magnitude slower than that of the free ligand). These NCs can be thoroughly purified, while preserving a PLQY above 90%, and can be densely packed in films, which exhibit high PLQY and good charge transport characteristics.

Inspired by these results, natural lecithin (a zwitterionic phospholipid with branched chains) was proposed as an effective ligand due to its branched chains that increase interparticle repulsion, thus enabling a high effective recovery of the NCs as well as single-nanoparticle spectroscopy when using diluted samples.^170^ In addition to these X-type acid ligands, L-type ligands which possess lone electron pairs can also interact with lead ions with an unoccupied orbital.^77^ However, from the synthesis viewpoint, it is difficult to introduce L-type ligands since many of them cannot dissolve the precursors. Interestingly, Zhang *et al*.^77^ prepared CsPbBr_3_ NCs following the room-temperature antisolvent strategy, using only oleylamine (OLA) as the ligand. In this strategy, the polar solvent dissolves the precursors efficiently, thereby enabling the direct interaction of OLA with the NC surface; both theoretical and experimental results confirmed the significant passivation effect and strong binding energy of OLA. As a consequence, the NCs exhibited a PLQY close to unity and dramatically improved their stability when undergoing purification processes and in the presence of water.

To our knowledge, there are hardly any examples of surface passivation of lead-halide NCs with Z-type ligands; namely, the K^+^ cation and the K-oleate complex were used as passivating ligands. González-Carrero *et al.* prepared K^+^-capped CH_3_NH_3_PbBr_3_ NCs by adding KPF_6_ to the perovskite precursor dimethylformamide solution following the reprecipitation strategy.^330^ The K^+^ counterion is more lipophilic and less coordinating than bromide ions and replaced the excess of methylammonium cation at the NC surface. The NCs effectively self-assembled on a substrate to produce homogeneous solid films. On the contrary, Huang *et al.* added K-oleate to a toluene dispersion of previously prepared CsPbBr_3_ NCs by following a hot-injection protocol; the post-synthetic treatment of the CsPbBr_3_ NCs with K-oleate enhanced their photoluminescence and photostability.^353^

Interestingly, the high dynamic bonding between the NC surface and some capping ligands can be used advantageously to assemble perovskite NCs into two-dimensional superstructures. Zhang *et al.*(354) have reported on the linear assembly of CsPbBr_3_ NCs within PbSO_4_-oleate polymers, resembling the morphology of a peapod. The capping pod mostly preserved the NC optical properties. In addition, González-Carrero *et al.*(355) reported on the linear assembly of CH_3_NH_3_PbBr_3_ NCs in lead(II) polymers by simply mixing the precursors of both the NC and the polymer. Correlative single-particle fluorescence and AFM evidenced the formation of ordered and nonconnected CH_3_NH_3_PbBr_3_ NC–polymers, which were emissive and showed PL intermittency.

**Figure 33:**
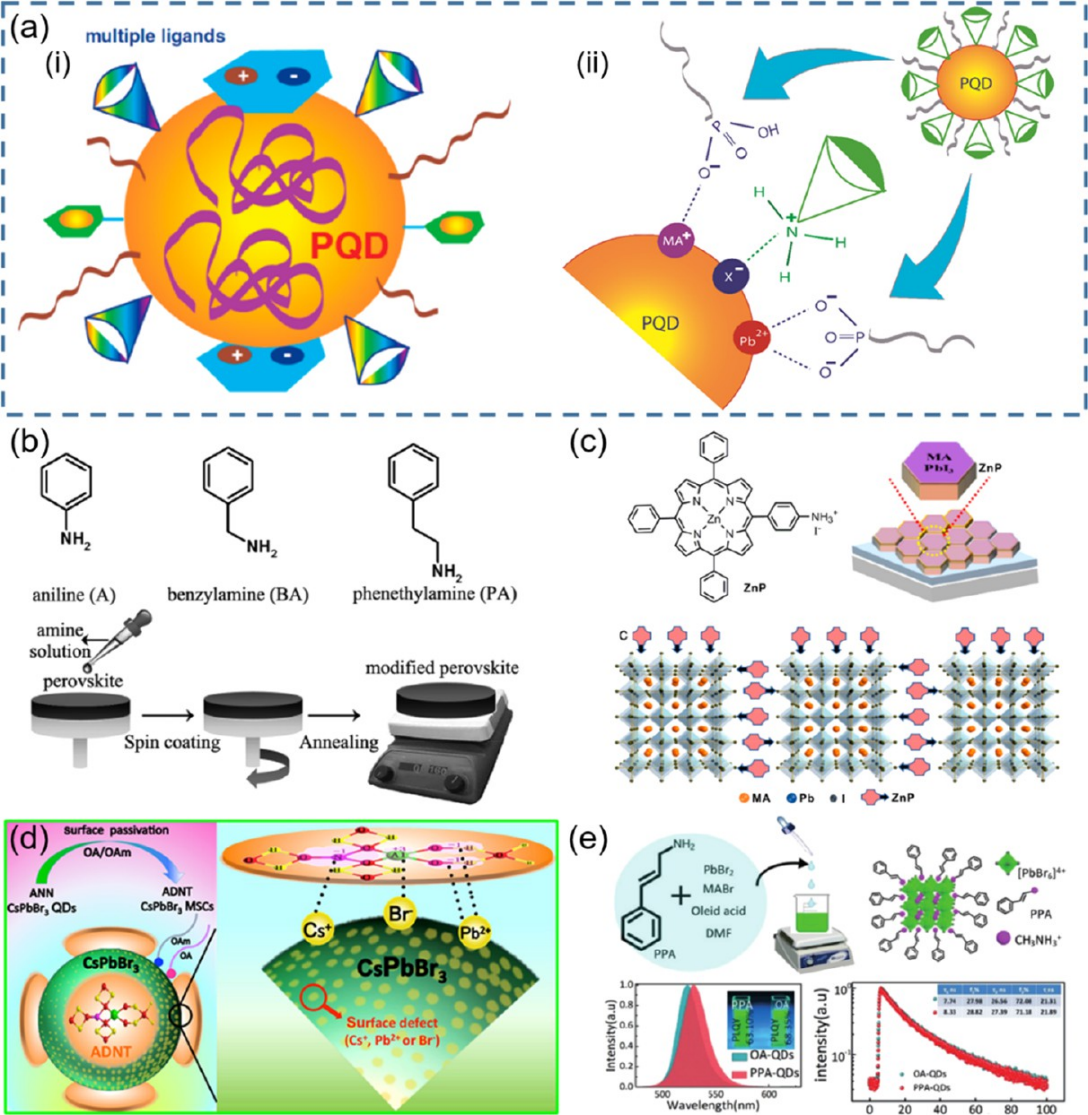
(a-i) Structural model for surface passivation of LHP NCs with multiple defects using a combination of ligands in a “cocktail” approach. Reproduced from ref (356). Copyright 2019 American Chemical Society. (a-ii) Schematic illustration of the major surface passivation mechanism of CH_3_NH_3_PbBr_3_ perovskite NC surface defects. Reproduced with permission from ref (360). Copyright 2019 John Wiley & Sons, Inc. (b) Chemical structures of aniline, benzylamine, and phenethylamine, and schematic illustration of amine treatment of perovskite films through a spin-coating method, followed by an annealing process. Reproduced with permission from ref (359). Copyright 2016 John Wiley & Sons, Inc. (c) Structure of Zn-porphyrin (ZnP), scheme illustration of MAPbI_3_ film with ZnP “doping”, and structure of perovskite encapsulated by ZnP. Reproduced from ref (367). Copyright 2019 American Chemical Society. (d) Schematic illustration of the major surface passivation mechanism of CsPbBr_3_ magic-sized clusters or perovskite NCs surface defects. Reproduced from ref (368). Copyright 2019 American Chemical Society. (e) Illustration of the MAPbBr_3_ NCs processing progress, the structural representation of PAA-perovskite NCs (PAA: 3-phenyl-2-propen-1-amine) in which PAA instead of OA (oleic acid) acts as capping ligands, steady-state PL spectra and representative photograph and PLQY values of PPA-perovskite NCs and OA-perovskite NC colloidal solution under 365 nm UV light, TRPL (time-resolved photoluminescence) spectra of PPA-perovskite NCs and OA-perovskite NC colloidal solution and color-tunable MAPbX_3_ perovskite NCs with PPA as capping ligand. Reproduced with permission from ref (369). Copyright 2018 John Wiley & Sons, Inc.

Simultaneous passivation of both cationic and anionic defects with anionic and cationic ligands is usually required for efficient stabilization of LHP NCs, and this essentially demands a “cocktail” approach, as illustrated in Figure 33a-i.^356^ The degree of acidity and basicity of the ligands is also important for effective passivation, as the defects can have a varying degree of acidity or basicity. Over the years, a wide range of organic acids and amines (*e.g*., oleylamine/oleic acid, octylamine, phosphonic acids (PAs), APTES, l-cysteine, aniline/benzylamine, phenethylamine, and *n*-trioctylphosphine (TOP) have been tested as ligands for LHP NCs.^14,138,332,352,357−365^ For instance, in the case of CsPbI_3_, Cs^+^ is considered as a weak acid, Pb^2+^ a weak acid, as well, and I^–^ a weak base,^366^ while in the case of MAPbBr_3_, MA^+^ is a weak acid and Br^–^ is a weak base (though stronger than I^–^).^360^ Based on the Pearson acid/base case concept, weak acid defects require weak base ligands, while weak base defects require weak acid ligand for optimal passivation.^360^ For example, short-chain organic PAs have stronger acidity, and thus their conjugate base has basicity stronger than that of their longer-chain counterparts.^360^ Four different linear alkyl PAs [PAs with the straight chain from short to long: MPA, *n*-hexylphosphonic acid, 1-tetradecylphosphonic acid (TDPA), and *n*-octadecylphosphonic acid (ODPA)] have been used in conjunction with APTES as capping ligands to synthesize MAPbBr_3_ perovskite NCs.^360^ As illustrated in Figure 33a-ii, the protonated APTES and deprotonated PAs produce weak acidic R–NH_3_^+^, weak basic R–PO_2_(OH)^−^, and even weaker basic R–PO_3_^2^. These ions likely passivate the surface weak basic Br^–^, weak acidic MA^+^, and even weaker acidic Pb^2+^ cations, respectively. In addition, MPA-APTES has larger acid–base equilibrium constant (*K*_eq_) compared with that of HLA-APTES, TDPA-APTES, and ODPA-APTES, thereby producing of a higher concentration of R–NH_3_^+^, R–PO_2_(OH)^−^, and R–PO_3_^2–^. Therefore, better passivation is achieved with the most acidic and shortest chain MPA.^360^ Similarly, a change in basicity of amines can also affect the passivation. As shown in Figure 33b, FAPbI_3_ films have been prepared using amines with different basicity: aniline (p*K*_a_ 4.87), benzylamine (p*K*_a_ 9.34), and phenethylamine (p*K*_a_ 9.83). The basicity of these amines follows the order of phenethylamine > benzylamine > aniline; therefore, protonated phenylalkylamine should be the weakest acid, which should provide the most effective passivation by interacting with the weak base I^–^.^359^

If the acidity and basicity of both the acidic and basic ligands with the same anchoring groups are changed in the precursor solution during synthesis, the passivation outcome can also change. For instance, Pan *et al.*(177) systematically varied the hydrocarbon chain length of carboxylic acids and amines, from 18 carbons (18C) down to 2 carbons (2C), including carboxylic acids including C18A (OA), C12A (dodecanoic acid), C8A (octanoic acid), and C6A (hexanoic acid) and amines including C18B (OLA), C12B (dodecylamine), and C6B (hexylamine), to understand their effect on the surface properties of CsPbBr_3_ PNCs. These organic surfactant molecules affect the nucleation and crystallization processes, with the C18A–C18B sample showing the highest PLQY indicative of the best passivation. This is attributed to the longer chain length C18A–C18B with larger *K*_eq_, which produces higher concentrations of −COO^–^ and −NH_3_^+^ ligands that are stronger bases and acids than that of the short-chain molecules to passivate Cs^+^, Pb^2+^, and Br^–^ defects.

The size and shape of molecular ligands can also strongly influence the effectiveness of passivation of MHPs, partly due to different steric hindrance, which in turn affects the morphology, crystalline phase, and optical and electronic properties of MHPs.^356^ On the other hand, MHPs of different sizes and shapes can create different combinations and types of defects and therefore demand molecular ligands with different sizes and shapes for optimal passivation. In addition to passivating the surface defects through the anchoring groups, the size and shape of the ligands are particularly important in stabilizing MHPs by preventing reaction with external environmental species such as O_2_ and moisture.^346^ In particular, large ligands can afford multiple functional groups in one molecule. For example, butylphosphonic acid 4-ammonium chloride with a combination of phosphate and amino functional groups can simultaneously passivate MA^+^, Pb^2+^, and I^–^ defects.^370^ Additionally, suaraine, polyaniline, and quaternary ammonium salts have been shown to be good capping ligands for MAPbI_3_ bulk, MAPbI_3_ film, and MAPbBr_3_ bulk, respectively.^358,371^ Peptides containing both −NH_3_^+^– and −COO^–^– in one molecule have been used to passivate MA^+^, Pb^2+^, and Br^–^ of MAPbBr_3_ perovskite NCs.^363^ Similarly, trifunctional l-cysteine has been used to passivate MAPbBr_3_ perovskite NCs and induced self-assembly of perovskite NCs, based on synergistic effects among −NH_3_^+^–, −COO^–^–, and −SH– groups.^357^ Therefore, the key choice of the size of molecular ligands not only depends on the size and surface defects distribution of perovskites but also relates to the synergistic effects of the functional groups of the used ligands.

Regarding the ligand shape, this can be linear, branched, umbrella-shaped, planar, or spherical. Most studies to date have used linear-shaped molecules, such as OA and OLA as capping ligands.^84,372^ In addition, a few attempts were made with branched ligands. For instance, Zhu *et al.*(373) used protonated (3-aminopropyl)trimethoxysilane (APTMS, umbrella-shaped) ligands in the synthesis of CsPbBr_3_ perovskite NCs. The authors found that the resulting NCs exhibit improved PLQY and stability in polar solvents. Similarly, umbrella-shaped APTES and POSS PSS-(3-(2-aminoethyl)amino)propyl heptaisobutyl-substituted (NH_2_–POSS) have been used along with OA to passivate MAPbBr_3_ perovskite NCs for enhanced stability.^344^ This is attributed to the strong steric hindrance and propensity for hydrolysis of APTMS, APTES, and NH_2_–POSS, which prevent molecules such as H_2_O and O_2_ from reaching and reacting with the core of perovskites.

The combination of the umbrella-shaped APTES and linear OA does not appear to improve the stability of bulk MAPbI_3_ films, an effect that can be attributed to the higher steric hindrance among APTES molecules.^374^ However, interestingly, linear OA alone is highly effective in passivating bulk films but not perovskite NCs. This is likely because the linear OAs can form a self-assembled monolayer on the bulk film surface, which is less likely for perovskite NCs due to their large curvature.^374^ For bulk MHP films, some planar and spherical molecular ligands also show good passivating ability. As shown in Figure 33c, when the planar molecular ligand of monoammonium ZnP is used as a molecular ligand for MAPbI_3_ film, the interaction between NH_3_^+^ and I^–^ leads to effective passivation.^367^ Another interesting planar molecular ligand is aluminium dihydroxide nitrate tetrahydrate (ADNT), along with OA and OLA, which was found to passivate the CsPbBr_3_ surface very efficiently to the point that PMSCs were generated in addition to perovskite NCs (see the section below).^368^ This was attributed to the ADNT being planar on the surface of the PMSCs or perovskite NCs with its NO_3_^–^ and OH^–^ groups binding to the Cs^+^ and Pb^2+^ defect sites and Al^3+^ binding to the Br^–^ defect sites of the PMSCs or perovskite NCs (Figure 33d). In addition, the spherical-shaped molecular ligand of mesostructured [6,6]-phenyl-C61-butyric acid methyl ester (ms-PCBM) has been used to passivate MAPbI_3_ films owing to the hydrophobic and high-performance mesostructure of ms-PCBM.^376^ It would be interesting to test such ligands for perovskite NCs, as well.

Although long alkyl chain and alkoxysilanes molecular ligands are effective in passivating MHP NCs to improve their optical properties and stability, their insulating nature limits electronic coupling among MHP NCs and thereby impedes charge transfer and transport important for device application.^335,369^ One way to improve inter-NC coupling and charge transport is to use conjugated or conductive molecular ligands, such as aromatic, alkene, and alkyne compounds with an unhindered positive or negative terminal ion that will interact strongly with the surface defects.^335,369^ For instance, as shown in Figure 33e, the conjugated amine containing a C=C group of an aromatic molecule ligand 3-phenyl-2-propen-1-amine (PPA) has been used to prepare MAPbBr_3_ NCs.^369^ Compared with OA, the carrier mobility of bulk PPA-MAPbBr_3_ film increases almost 22 times over that of PA-MAPbBr_3_ films without compromising stability and optical properties. The conductivity of PPA-MAPbBr_3_ perovskite NC films was improved due to enhanced coupling between perovskite NCs.^369^ Similarly, conjugated PPA with both “quasi-coplanar” rigid geometrical configuration and distinct electron delocalization characteristics has also been used to modify MAPbI_3_ films. The conjugated cation coordinating to the surface of the perovskite grains/units provides a network for charge exchange.^377^ In addition, short conductive aromatic capping ligands such as benzylamine (BZA) and benzoic acid (BA) have also been used to synthesize MAPbBr_3_ perovskite NCs with high PLQY (86%), indicative of a well-passivated surface. The perovskite NCs synthesized using BZA/BA capping ligands exhibit higher conductivity and longer charge carrier lifetime compared to those of MAPbBr_3_ perovskite NCs with insulating OA and APTES capping ligands. This was attributed to the delocalization of the excitonic wave function of the perovskite NCs by the aromatic ligands.^335^

The valency or oxidation state of the ligand and the charge density and distribution in the ligand can critically affect how effective it can passivate the MHPs. For monovalent and divalent cationic surface defects, it would be ideal to use corresponding oppositely charged monovalent and divalent ligands for their passivation. With some weak acid ligands such as PAs, multiple conjugate bases with different valency or charges can be produced upon deprotonation, which can passivate differently charged cations, such as MA^+^, Cs^+^, or Pb^2+^ defects.^356^ Specifically, for the PA-APTES MAPbBr_3_ perovskite NCs discussed earlier, APTES is protonated and can produce a charged functional groups of R–NH_3_^+^ to passivate Br^–^. On the other hand, with the two proton transfers of R–PO_2_(OH)^−^, PA can produce two charged functional groups of R–PO_2_(OH)^−^ and R–PO_3_^2–^ that could passivate MA^+^ and Pb^2+^, respectively.^360^ The above example is in contrast to OA-APTES MAPbBr_3_ perovskite NCs that have two charged functional groups of R–NH_3_^+^ and R–COO^–^, with the latter passivating both MA^+^ and Pb^2+^.^360^ Therefore, the valence state of the molecular ligands should ideally be consistent with the valence state of the surface defects for optimal passivation.

### Passivation of Perovskite Magic-Sized Clusters

Perovskite magic-sized clusters (PMSCs) are ultrasmall (usually <2 nm) nanoparticles with a narrow size distribution and strong quantum confinement. Recently, it has been found that the ligands play a key role on the preparation and passivation of PMSCs, that is, clusters that have a single size or in any case an extremely narrow size distribution.^368,375^ Compared to perovskite NCs, PMSC are smaller and less stable and thereby they require better protection or passivation. As a result, strong ligands and high concentrations of ligands favor PMSCs over perovskite NCs.^368,375^ Because of their highly uniform size distribution and narrow optical bandwidth, PMSCs are attractive for studying fundamental issues and as potential building blocks for creating larger PNCs.^378−380^ It was found that one of the key factors in producing PMSCs is the amount of Lewis acid ligands used, with more acids leading to more PMSCs.^368^ However, not all the type of Lewis acids can produce pure PMSCs, and the PMSCs only exist in organic solvent owing to their small size (< 2 nm).^368^

To date, there have been a few reports on PMSCs and their ensembles. Single sized (∼2–4 nm) APbX_3_ (where A = CH_3_NH_3_^+^ or Cs^+^) nanocrystalline phosphors have been synthesized using OA and OLA as capping ligands, showing a high PLQY (∼80%).^381^ CsPbBr_3_ nanoclusters with ∼2 nm size and a sharp absorption peak at ∼398 nm have been synthesized using OA and OLA as capping ligands and converted into highly deep blue-emitting nanoribbons.^382^ In addition, smaller size clusters (∼0.6 nm) of CsPbBr_3_ (nearly equal to the CsPbBr_3_ unit cell length of 0.59 nm) have been synthesized using OA and OLA ligands.^316^ Zhang *et al*. found that the single size of MAPbBr_3_ and CsPbBr_3_ PMSCs are strongly dependent on the ligands used.^368,375^ As shown in Figure 33d, a distinctive inorganic capping ligand based on a trivalent metal hydrated nitrate coordination complex, Al(NO_3_)_3_·9H_2_O), together with OA and OLA, has been used to control the synthesis of CsPbBr_3_ PMSCs and CsPbBr_3_ perovskite NCs. By changing the amount of metal complex ligand used, the final product can be tuned from perovskite NCs to PMSCs or to a mixture of both NCs and PMSCs, with excess ligands favoring PMSCs.^368^ The conversion from CsPbBr_3_ perovskite NCs to PMSCs is mainly related to the concentration of trivalent metal hydrated nitrate coordination complexes (TMHNCCs). The concentration of (TMHNCCs) affects the excitonic absorption of the CsPbBr_3_ PMSCs (λ = 430–441 nm) and CsPbBr_3_ perovskite NCs (λ = 447–518 nm), with more TMHNCC favoring CsPbBr_3_ PMSCs over perovskite NCs.^368^ Due to the ultrasmall size and extremely large surface to volume ratio of PMSCs, a higher concentration of molecular ligands are necessary compared to perovskite NCs.

### Strategies to Gain Insights into the Ligand–Surface Interactions

Pan *et al.*(177) have studied how to gain information on the ligand–surface interaction in CsPbBr_3_ NCs from their purification step. The as-synthesized NCs were purified using hexane and a hexane/acetone mixture. NMR and FTIR measurements demonstrated that ammonium ligands can be preferentially removed from the NC surface compared to carboxylate; this is consistent with the weaker strength of the H-bonding interaction of alkylammonium with the surface bromide atoms [Br···H–N^+^] compared to the lead–carboxylate coordination. The treatment of the NCs with a polar solvent destabilizes the hydrogen bond interaction producing a detachment of the ammonium from the NC surface, as was evidenced by the decrease and disappearance of the N–H bending vibration band in the FTIR spectrum (1575 cm^–1^), while alkene protons (5.50 ppm) from oleate remained unchanged. Solvent-dependent ligand–surface interactions were clearly demonstrated, and this finding should be considered when ligands and washing solvents are used in the synthesis and purification steps.

A washing treatment with an antisolvent reduces the colloidal stability of the NCs due to a decrease of the ligand density on the NC surface. The addition of didodecyldimethylammonium bromide (DDABr), a branched ligand, can promote the exchange of the pristine ligands (oleylamine and oleic acid) on the NC surface with DDABr, thereby enhancing their photostability. However, this strategy has not proved successful enough in protecting the NC surface, as the obtained NCs deteriorated unavoidably after the washing step. The unwashed NCs were then sealed into a resin to fabricate a blue LED, which exhibited a higher photostability than that prepared with pure NCs.^383^ We recommend to read subsection Isolation and Purification of Colloidal MHP Nanocubes, for specific examples on metal-halide perovskite nanocubes.

In addition, post-synthesis ligand exchange allows one to estimate the binding constant of the added ligands to get thermodynamically stable coordination of organic ligands to the NC surface. Thus, two different surface CsPbBr_3_ NCs were prepared using the hot-injection method: (i) NC terminated with oleylammonium bromide (PLQY of 92%) and (ii) NC terminated with Cs-oleate species (PLQY of 69%).^384^ Interestingly, the reduction of scattering was associated with the saturation of the NC binding energy. It has been demonstrated that primary alkyl ammonium and benzylammonium bromides bind to the NC surface with a binding constant >10^5^ M^–1^, but the constant is reduced to 10^4^ M^–1^ with short length ligands, sterically hindered ligands (*e.g*., triethylammonium and oleylammonium), and weak acid ligands (such as phenylammonium). The higher the binding constant of the ligands to the NC surface, the better the long-term stability and emissive properties due to a complete surface passivation. However, the excess of ammonium ligands could transform the core of the NCs by substitution of cesium and reconstruction of the NCs inducing a blue shift in the emission.

Post-synthetic treatment of CsPbI_3_ NCs with a dicarboxylic acid, namely, 2,2′-iminodibenzoic acid, enhanced their PLQY from 80 to 95%. NMR, XPS, and FTIR measurements confirmed the bidentate binding of the ligand by the carboxylic groups. DFT calculations are consistent with the anchoring of the bifunctional ligand to two lead atoms at the NC surface with a binding energy of 1.4 eV, compared to a binding energy of 1.14 eV for oleic acid. The dicarboxylic ligand stabilizes the NC surface, with low structural distortion and phase transformation, leading to high PLQY.^172^

### Post-synthetic Passivation of CsPbBr_3_ NCs

**Figure 34:**
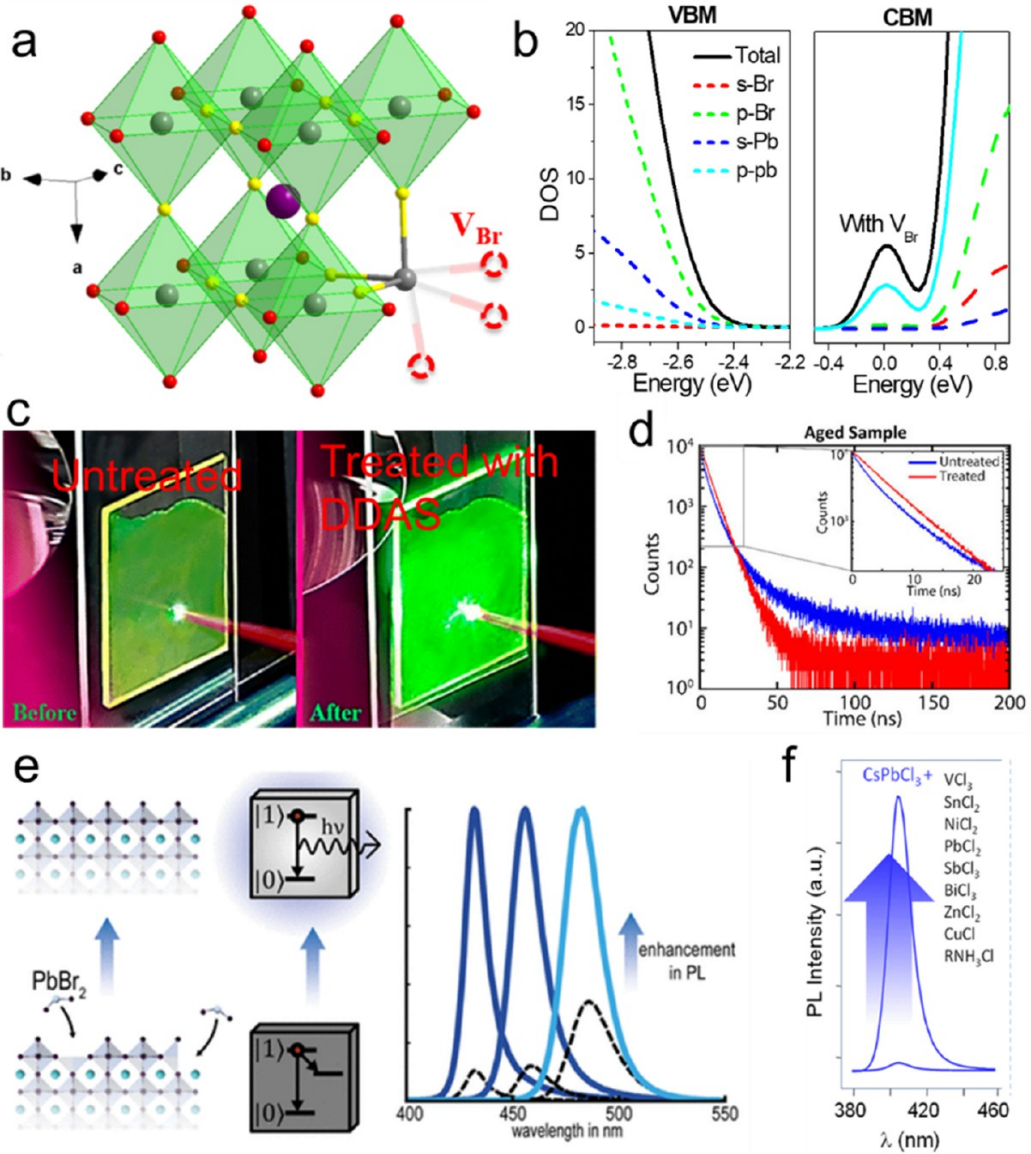
(a) Crystal structure of CsPbBr_3_ NC, with the presence of a surface V_Br_. Reproduced with permission from ref (392). Copyright 2019 John Wiley & Sons, Inc. (b) Electronic density of states (DOS) curves of valence band maximum and conduction band minimum of CsPbBr_3_ with V_Br_. Reproduced with permission from ref (352). Copyright 2019 John Wiley & Sons, Inc. (c) CsPbBr_3_ NC films before and after being treated with DDAS. Reproduced from ref (389). Copyright 2015 American Chemical Society (d) PL decay of treated and untreated CsPbBr_3_ NC with NH_4_SCN. Reproduced from ref (88). Copyright 2017 American Chemical Society. (e) CsPbBr_3_ NPls with post-treatment of PbBr_2_. Reprinted with permission under a Common Creative Attribution 4.0 International license from ref (60). Copyright 2018 American Chemical Society. (f) Greatly improved PL of CsPbCl_3_ NCs after being treated with metal chlorides. Reproduced from ref (87). Copyright 2018 American Chemical Society.

The performance of LHP@capping NCs by following a post-treatment of the NCs has focused on all-inorganic CsPbX_3_. Defect energy levels result from the crystal discontinuity on the surface, and the role of passivation is mainly to reduce (ideally eliminate) the resulting surface defects. It is widely acknowledged that surface cesium atoms of CsPbX_3_ NCs are replaced with protonated amine ligands, which interact with halide atoms through hydrogen bonding (Figure 34a).^17,350,385^ Since only the orbitals of Pb and X atoms contribute to the band-edge, exciton recombination seems to take place primarily within the Pb–X octahedrons. The symmetric crystal structure makes lead vacancy (V_Pb_) hardly affect the exciton recombination while V_X_ considerably influences the recombination process (Figure 34b).^357,386,387^ Therefore, the main purpose of both the post- and *in situ* passivation strategies is to fill the V_X_ on the surface. Furthermore, if the ligands possess physicochemical properties similar to those of halide ions, they can passivate the V_X_ directly. Pan *et al.* initiated the post-treatment of perovskite NCs in late 2015.^388^ CsPbBr_3_ NCs were first treated with oleic acid and then with didodecyl dimethylammonium bromide (DDAB) or DDAS (here S means S^2–^; Figure 34c).^177,389^ The treatment significantly improved the PLQY and the stability of the CsPbBr_3_ NCs and enabled stable stimulated emission from the NCs after 1.2 × 10^8^ laser shots. The pretreatment of the NCs with oleic acid before the adsorption of DDAB is an indication of the complexity of the ligand–NC interaction. After that, Alivisatos’ group treated CsPbBr_3_ NCs with thiocyanate salts (NH_4_SCN, NaSCN) and NH_4_Br^88^ by adding the salt powder into the NC dispersion directly and stirring the mixture at room temperature. They reported a PLQY value close to unity, with an obvious monoexponential PL decay (Figure 34d). The key point of this method is repairing a lead-rich surface (surface with V_X_) with pseudohalogen ions by post-treating CsPbBr_3_ NCs with bromides or related chemicals. For example, tetrafluoroborate salts, ZnX_2_, and PbBr_2_ were used as the post-treating agents to improve the PLQY of green CsPbBr_3_ NCs^195,357,390^ to close to 100%. In addition to these inorganic salts, organic salts with bromides were also applied to repair the surface V_Br_ to provide NCs with a PLQY of 100%.^161^ Such ligands endow CsPbBr_3_ NCs with strong endurance against polar solvent washing and ambient storage, indicating their better potentials in future optoelectronic devices. The post-treatment of blue-emitting perovskite NCs is generally difficult. There are mainly two types of three-dimensional, blue LHPs: mixed-halide perovskites and CsPbBr_3_ nanoplatelets. It is difficult to accurately passivate surface V_X_ of mixed halides since ion exchange occurs easily,^55^ and it is challenging to ensure the stability of the emission wavelength during the surface treatment. For CsPbBr_3_ NPls, poor stability is the main obstacle during post-passivation.^235^ In spite of these difficulties, some interesting studies have been reported. For instance, the treatment of CsPbBr_3_ NPls of different thicknesses with a PbBr_2_–ligand solution led to an overall enhancement of their low PLQY (Figure 34e).^60^ Considering NCs with a shorter emission wavelength, such as CsPbCl_3_ NCs, Pradhan’s and others’ groups conducted comprehensive experiments and demonstrated giant PL enhancement when CsPbCl_3_ NCs were treated with various types of metal chlorides (Figure 34f).^87,148,391^ It should be noted that no doping was detected. Considering the similarity of these inorganic salts, there is no doubt that the passivation of Cl vacancies on NC surface contributes greatly to the enhanced PLQY.^86,101^

### Post-synthesis Passivation *versus In Situ* Passivation of LHP NCs

**Figure 35:**
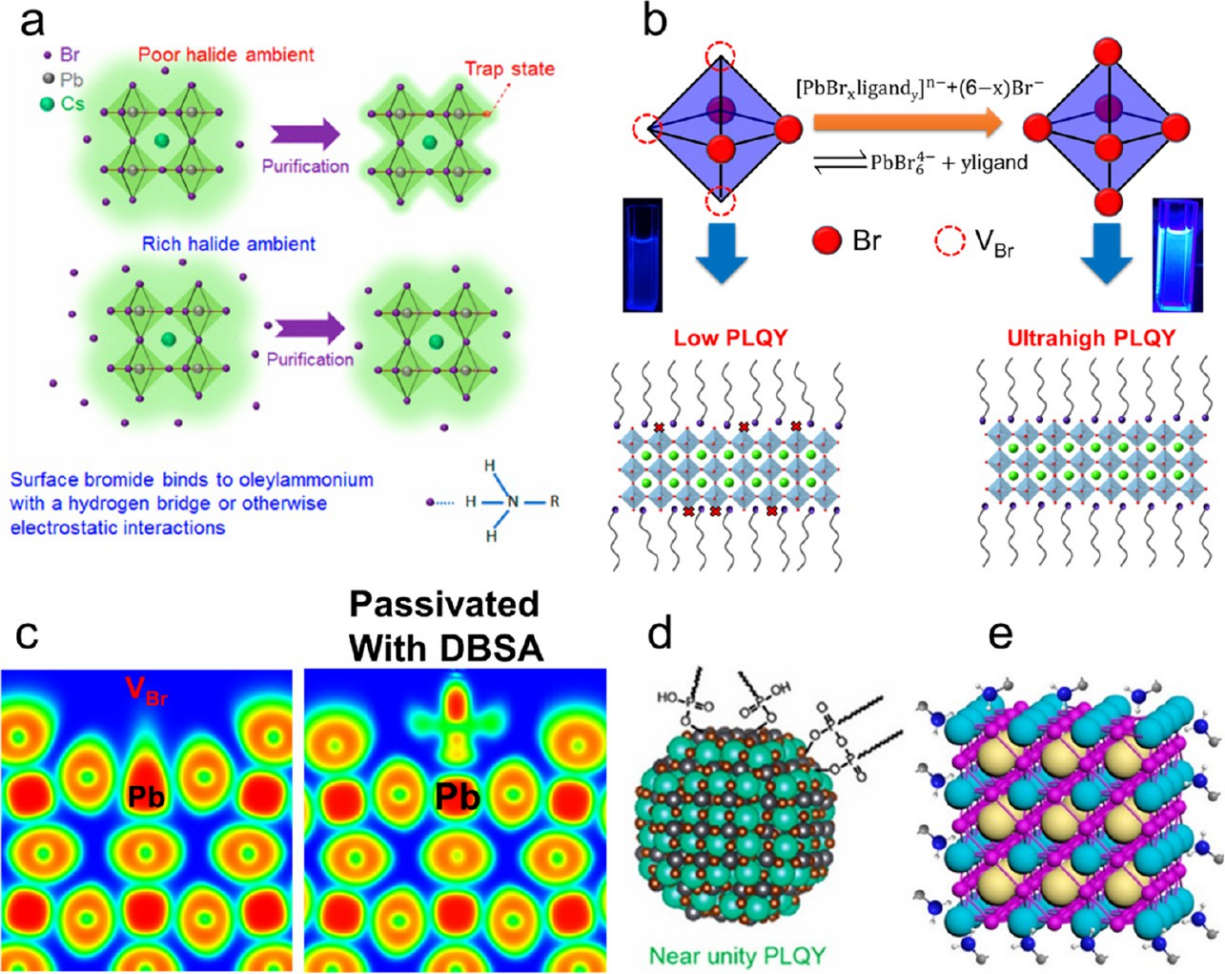
(a) Schematics for conventional and *in situ* passivation under halide-rich conditions with inorganic ammonium bromides. Reproduced from ref (321). Copyright 2017 American Chemical Society. (b) Schematic for ionic-equilibrium-based *in situ* passivation strategy for highly efficient CsPbBr_3_ NPls. Reproduced from ref (398). Copyright 2018 American Chemical Society. Direct *in situ* passivation with (c) X-type DBSA. Reprinted with permissions from ref (352). Copyright 2019 John Wiley & Sons, Inc. (d) X-type alkyl phosphonic acids and (e) L-type oleylamine. Panels d is reprinted from ref (351). Copyright 2019 American Chemical Society. Panels e is reprinted from ref (77). Copyright 2019 American Chemical Society.

The post-passivation strategy is a widely accepted strategy in the field of common semiconductor and perovskite NCs. However, additional impurities are unavoidable in such strategy and this might be detrimental for their optoelectronic properties. Further purification is often necessary to remove the unreacted chemicals, which is challenging for perovskite NCs. Eliminating the surface defects during synthesis, *i.e*., *in situ* passivation, *via* surface stoichiometric control, ligand design, and precursor engineering may be more favorable as no further treatment and purification steps are needed.^327,393−395^ As mentioned above, the main purpose of perovskite NC passivation is to compensate the halide vacancies (V_X_) on the surface. According to this principle, Liu *et al.* added ammonium halide in the precursors to construct halide-rich NCs (Figure 35a).^321^ During and after growth, the excess halide ions in the solution can fill the surface vacancy efficiently, contributing to reduce the nonradiative process and consequently enhancing the NC PLQY. This strategy was further modified by several other groups using metal bromides (ZnBr_2_, MnBr_2_, PbBr_2_, among others)^332,396,397^ to passivate the surface defects and consequently a PLQY close to unity was achieved. Although these metal bromides were added together with the precursors, no NC doping was observed.

LEDs fabricated with the these NCs exhibited a record EQE value of 16.8%, indicating the superiority of the *in situ* passivation strategy; further studies are needed to gain insight into how these metal bromides work. By contrast, the addition of NiCl_2_ during the preparation of CsPbCl_3_ NCs resulted in NC Ni doping as well as a decrease in the surface chloride vacancy density,^399^ thus leading to NCs with a PLQY close to 100%. Therefore, more investigations are needed to identify the significance of the halide salts during synthesis.

Very recently, Yang *et al.* prepared highly efficient and stable CsPbBr_*x*_I_3–*x*_ NCs with emission wavelength at the pure red region (637 nm) through the addition of potassium-oleate.^400^ Potassium bromide was detected on the surface, which passivated the V_X_ and inhibited the halide segregation simultaneously. The final LED exhibited high EQE and especially stable emission peak. Usually, the addition of inorganic halides also introduces impurities to some extent. Then, Wu *et al.* developed an *in situ* passivation strategy with organic halides (oleylammonium bromide) obtaining a record PLQY of 96% for CsPbBr_3_ nanoplatelets emitting in the blue (Figure 35b).^398^ According to their approach, PbBr_6_^4–^ complexes could be formed before nucleation of the NCs by controlling the amount of HBr. The formation of single-layered hybrid perovskites capped with oleylammonium bromide after injection of PbBr_2_ precursor was followed by the disconnection between PbBr_2_ and ligands after the addition of HBr, thus shifting the ionic equilibrium toward the formation of isolated PbBr_6_^4–^ octahedral complexes, due to the increased Br^–^ concentration. The process was monitored by absorption spectroscopy. LEDs based on these NPls exhibited an ultranarrow electroluminescence emission with a full width at high maximum of 12 nm.

Direct *in situ* passivation was carried out with organic ligands of different natures and presenting strong affinity to Pb^2+^ ions: (i) X-type ligands, such as dodecylbenzenesulfonic acid^352^ and alkylphosphonic,^351^ and (ii) L-type ligand, such as oleylamine.^77^ The groups of Zhang and Pradhan prepared CsPbX_3_ NCs with ultrahigh PLQY by adding organic halides with long chains.^78,401,402^ Moreover, organic halides with multi-alkyl chains can participate in the *in situ* passivation of the NCs, but they are not detected on the surface due to their large steric hindrance.^392^ These organic halides play a role during the growth stage by enabling the formation of complete Pb–Br octahedrons and therefore a low surface V_X_ density. The surface is eventually capped by other long-chain ligands, such as oleic acid or oleylamine. This method provides more possibilities for tuning optical and structural features. The above-discussed *in situ* passivation methods were all based on the consideration of filling surface V_X_. In a sense, if the ligands can passivate the exposed lead atoms directly, we would achieve efficient perovskite NCs using simply one kind of ligand.

On the whole, since most of the results confirmed that surface V_X_ is at the origin of carrier trapping and nonradiative recombination,^194,392,399^ the passivation strategy design for trap-free perovskite NCs should focus on the eliminiation of surface V_X_. In fact, researchers have been succeeding in doing this and PLQY close to 100% have been achieved for almost all the visible emission wavelengths.

### Summary and Future Prospects for Surface Chemistry and Passivation of MHP NCs

Perovskite QDs relevant for optoelectronic devices require not only capping ligands that stabilize NCs and enhance their luminescence, but also that promote charge injection and transport at the interface. Long-chain saturated amines and carboxylic acids, such as oleylamine and oleic acid, have been commonly used as passivating ligands of perovskite NCs surface to enhance their stability and optical properties. However, their insulating nature creates an electronic energy barrier and impedes interparticle electronic coupling, thereby limiting the application of the NCs in optoelectronic devices. Thereby, different strategies have been tested to overcome this issue. Control of the surface ligand density on the NC surface has been devised as a way to improve the stability and PLQY, as well as the uniformity and carrier-injection efficiency of perovskite thin films, and it has been attained *via* treatment with a mixture of polar/nonpolar solvents.^184^

Shorter-chain saturated amines and acids have been used to enhance the performance of light-emitting diodes, such as those based on colloidal FAPbBr_3_ NCs capped with *n*-butylamine^403^ and CsPbBr_3_/CH_3_NH_3_Br quasi-core/shell structures^404^ to provide green LEDs with EQE of up to 2.05 and 20.3%, respectively. Moreover, CsPbI_3_ NC LEDs with EQE of 12.6% have been fabricated using octylphosphonic acid.^405^ In addition, relatively short-chain quaternary ammonium bromide salts, such as didodecyldimethylammonium bromide and didecyldimethylammonium bromide, has enabled the preparation of LEDs based on CsPbBr_3_ NCs with an EQE of 9.71%.^406^ Interestingly, long-chain ligands, such as 3-(*N,N*-dimethyloctadecylammonio)propanesulfonate, capable of coordinating simultaneously to the cation and anion of CsPbBr_3_ NC surface, have led to densely packed NC films in which the charge transport is not severely impeded.^171^

Ligand shortening combined with conductive capabilities has proved to be a promising strategy to facilitate charge transport between perovskite NCs by lowering the energy barrier.^335^ The passivation of MAPbBr_3_ QDs with benzylamine and benzoic acid enhances the conductivity and carrier lifetime as well as the charge extraction efficiency, while preserving the high chemical stability and PLQY of the perovskite. In this regard, Yan *et al.* have recently proposed the use 3,4-ethylenedioxythiophene to passivate CsPbBr_3_ NCs to provide photodetectors with enhanced performance by exploiting the ligand capacity to be polymerized on the NC surface under the photocurrent of the photodetector, thus enhancing the device performance in up to 178% while exhibiting high stability in air.^407^ This molecular engineering strategy can be of great interest for the development of high-performance and stable optoelectronic devices based on perovskite NCs. Somewhat related, Hassan *et al.*(408) have shown the beneficial effect of multidentate ligands to passivate effectively perovskite NCs, thus preventing halide segregation in I/Br mixed-halide perovskite LEDs under electroluminescent operation. Moreover, Han *et al.*(409) have recently applied the Lewis base cyclam (1,4,8,11-tetraazacyclotetradecane) as an effective, self-sufficient passivation, multichelating ligand of perovskite NCs, thus boosting the performance of light-emitting diodes (external quantum efficiency (EQE) of 16.24%). These results are encouraging and give clues on the nature of the ligands needed to enhance the charge injection and transport at the interface of the passivated perovskite surfaces. Identifying ideal ligands which enable even more efficient optoelectronic devices, which combine enhanced chemical stability and high efficiency in charge injection and transport at the interface, requires further experimental investigations, as well as state-of-art theoretical calculations on surface chemistry.

Future development of passivation strategies should take into consideration electrical and optical properties, colloidal stability, and operation stability, simultaneously.^170^ However, achieving these advantages together cannot be more challenging, and mixed passivation strategies with both organic and inorganic chemicals may be a better solution. Moreover, additional *in situ* passivation ligand systems are urgently needed to further promote the optoelectronic properties and stabilities.

## 0D Non-perovskite (Perovskite Derivative) NCs

The 2016–2017 reports^410−413^ on so-called “zero-dimensional” (0D) Cs_4_PbX_6_ (X = Cl, Br, or I) materials and NCs inspired many research works on the synthesis and device applications of Cs_4_PbX_6_ colloidal nanocrystals.^414^ Compared to their CsPbX_3_ counterparts (also referred to as 3D perovskites), Cs_4_PbX_6_ NCs were shown to have improved thermal and optical stability, especially with respect to their high PLQY of green emission in the solid state. From a crystal structure point of view, 0D Cs_4_PbX_6_ exhibit isolated [PbX_6_]^4–^ octahedral units—in contrast to the corner-sharing [PbX_6_]^4–^ octahedra of 3D CsPbX_3_—surrounded by Cs^+^ cations that are completely decoupled in all directions. The reduction of dimensionally from 3D to a strongly quantum-confined 0D gives rise to the molecular-like electronic properties of Cs_4_PbX_6_, such as a widened band gap and an increased exciton binding energy, a reduced charge carrier mobility, and a lower conductivity. Meanwhile, it brings several interesting photophysical features into play, like small polaron absoprtion and broad-band ultraviolet (UV) emissions. In the following, we will review the recent work on the 0D perovskite NCs, particularly Cs_4_PbBr_6_, by covering their syntheses and phase transformations, optical properties and molecular features, the origin of green emission, and optoelectronic applications.

### Synthesis and Phase Transformation of Cs_4_PbBr_6_ NCs

**Figure 36:**
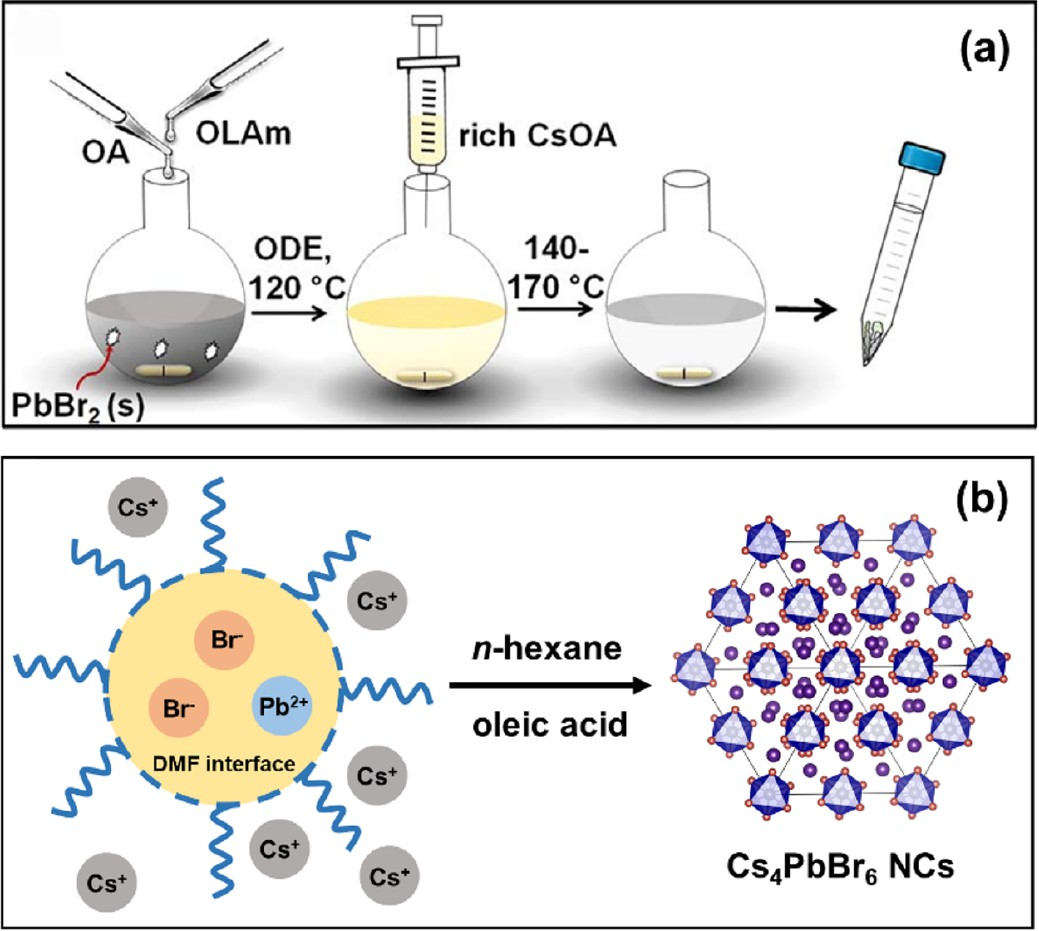
(a) Schematic illustration of the hot-injection method of synthesizing Cs_4_PbBr_6_ NCs in a rich Cs-oleate environment. Reproduced with permission from ref (415). Copyright 2019 Elsevier. (b) Schematic illustration of the low-temperature reverse microemulsion method of synthesizing Cs_4_PbBr_6_ NCs formed at the interface between an “oil” phase (*n*-hexane) and an “aqueous” phase (DMF). Redrawn from ref (413). Copyright 2017 American Chemical Society.

Hot-injection and low-temperature reverse microemulsion methods are two popular methods to obtain highly monodisperse Cs_4_PbX_6_ NCs. The former method is also best known for synthesizing highly luminescent CsPbX_3_ NCs, as shown by Protesescu *et al.*(14) In their developed method, the precursor PbBr_2_ was first dissolved in a nonpolar solvent with a combination of oleic acid and oleylamine and then Cs-oleate complex was injected (Figure 36a). Based on this, Akkerman *et al.* utilized a similar hot-injection strategy, but under Cs-rich conditions, to obtain nearly monodisperse Cs_4_PbX_6_ NCs with the size distribution of 10–36 nm.^411^ After that, Udayabhaskararao *et al.* developed another hot-injection method by mediating the excess ligands, and they found that the size of Cs_4_PbX_6_ NCs can be tuned by controlling the ratio of OA/OLA and also by the temperature.^416^ Meanwhile, Zhang *et al.* reported the synthesis of Cs_4_PbBr_6_ NCs (size distribution: 26 ± 4 nm) using a low-temperature reverse microemulsion method.^413^ As illustrated in Figure 36b, the precursors PbBr_2_ and Cs-oleate were first dissolved in DMF and hexane, respectively. These two solvents are immiscible, and thus, the NC nucleation rate was controlled by the slow release of Cs^+^ ions from the Cs-oleate complex when the solvents were mixed. The microemulsion method has been used to obtain other inorganic perovskite NCs with different dimensionalities (CsPbBr_3_ and CsPb_2_Br_5_),^1350^ as well as the ligand-free highly emissive Cs_4_PbBr_6_ NCs.^418^ Recently, Hui *et al.* reported a one-step method for the synthesis of Cs_4_PbBr_6_ NCs by mixing three independent precursors of Cs, Pb, and Br in a cuvette.^419^ They proposed a two-step pathway for forming Cs_4_PbBr_6_ NCs. First, Pb and Br precursors immediately react to form intermediates (*i.e.*, [PbBr_4_]^2–^, [PbBr_3_]^−^, and [PbBr_6_]^4–^), and then the Cs precursor (CsOA) induces the assembly of the intermediates into Cs_4_PbBr_6_ NCs.

**Figure 37:**
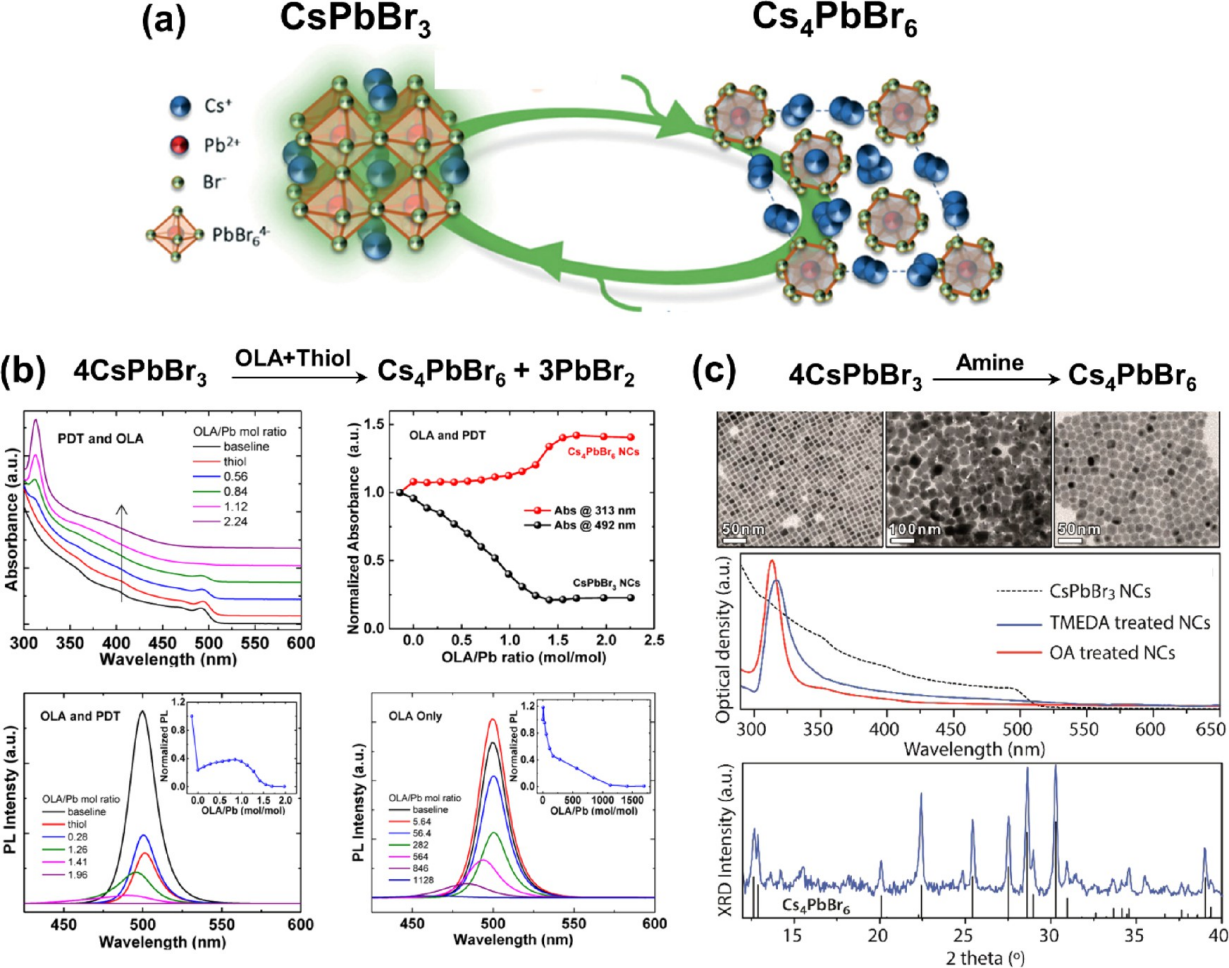
(a) Schematic illustration of the structural transformation between CsPbBr_3_ and Cs_4_PbBr_6_ NCs. Reproduced with permission from ref (420). Copyright 2018 Royal Society of Chemistry. (b) Absorbance spectra, normalized absorbance at two spectral features, and PL spectra of CsPbBr_3_ NCs solutions before and after adding different amount of oleylamine with and without PDT. Reproduced from ref (421). Copyright 2017 American Chemical Society. (c) TEM micrographs, absorption spectra, and XRD patterns of CsPbBr_3_ NCs before and after the treatment with either tetramethylethylenediamine (TMEDA) or OA. Reproduced from ref (422). Copyright 2017 American Chemical Society.

In addition to the direct synthesis methods mentioned above, Cs_4_PbBr_6_ NCs can be obtained *via* the phase transformation from CsPbBr_3_ to Cs_4_PbBr_6_ NCs by adding different amines (Figure 37a). For example, Liu *et al.* showed that after adding OLA into the solution of CsPbBr_3_ NCs, the absorption around 492 nm from CsPbBr_3_ NCs decreased while the absorption around 313 nm from Cs_4_PbBr_6_ NCs increased (Figure 37b).^421^ The evolution of the normalized absorbance at these two spectral positions had the inverse dependence on the OLA concentration. They found that including large amounts of 1,3-propanedithiol (PDT) had almost no effect on the absorption spectrum without adding OLA, indicating that the PDT cannot trigger the transformation. Therefore, such transformation was triggered by adding oleylamine and the size uniformity and chemical stability of the Cs_4_PbBr_6_ NCs can be improved by adding PDT. Palazon and co-workers provided another method to realize this transformation through adding the different amines at room temperature.^422^ They found the optical properties measured after TMEDA treatment were different from those of the starting solution of CsPbBr_3_ NCs. The spectral features (a sharp absorption peak at 317 nm, no absorption in the visible range and no significant green emission) together with XRD patterns indicate the transformation from CsPbBr_3_ to Cs_4_PbBr_6_ NCs (see Figure 37c).

**Figure 38:**
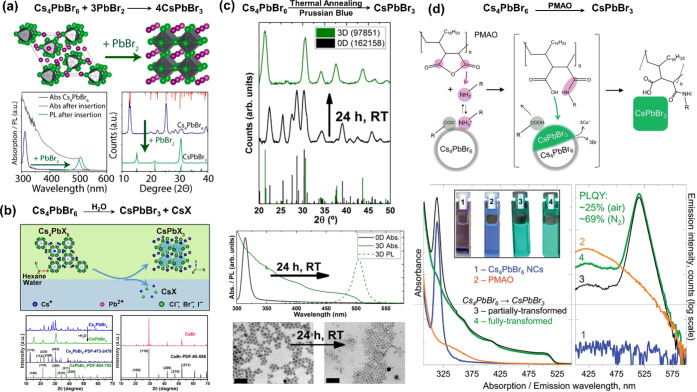
(a) Schematic illustration of phase transformation of the Cs_4_PbBr_6_ to CsPbBr_3_ after insertion of additional PbBr_2_ with the optical absorption, PL spectra, and XRD patterns of Cs_4_PbBr_6_ NCs before and after the insertion reaction. Reproduced from ref (411). Copyright 2017 American Chemical Society. (b) Schematic illustration of crystal structure change and transformation process from Cs_4_PbX_6_ to CsPbX_3_ after water treatment, together with the XRD patterns of Cs_4_PbBr_6_ NCs before and after adding water. Reproduced from ref (417). Copyright 2017 American Chemical Society. (c) XRD patterns, absorption spectra, and TEM images of Cs_4_PbBr_6_ NCs transformed to CsPbBr_3_ by adding Prussian blue. Reproduced from ref (423). Copyright 2017 American Chemical Society. (d) Schematic illustration of the transformation of Cs_4_PbBr_6_ into CsPbBr_3_ NCs induced by PMAO, together with optical absorption and emission spectra of initial Cs_4_PbBr_6_ NCs, PMAO, and partially and fully transformed NCs in toluene solutions. Reproduced with permission under Creative Common CC-BY 3.0 license from ref (424). Copyright 2020 Royal Society of Chemistry.

Reversibly, Cs_4_PbBr_6_ NCs could be transformed back to CsPbBr_3_ NCs through an insertion reaction with PbBr_2_.^411^ As shown in Figure 38, this transformation would lead to the shape change from the hexagonal to cubic structure, as well as the changes of spectral features, including absorption, PL spectra and XRD patterns. The transformation from Cs_4_PbBr_6_ to CsPbBr_3_ enabled the preservation of CsPbBr_3_ NCs size and crystallinity. Wu *et al.* reported the water-triggered transformation from Cs_4_PbX_6_ to CsPbX_3_ NCs with tunable optical properties and improved stability in air.^417^ Such transformation occurred at the interface of water and a nonpolar solvent, leaving the product of CsPbX_3_ NCs in the organic solvent and the byproduct in the water. They highlighted that the high solubility of CsX in water and the interface between nonpolar solvent and water played important roles in the transformation process. In addition, the transformed CsPbBr_3_ NCs showed better stability against moisture than those obtained through the hot-injection method. In addition to the phase transformation triggered by PbBr_2_ or water, Palazon *et al.* showed that Cs_4_PbBr_6_ NCs can be transformed into CsPbBr_3_ NCs either by thermal annealing or by reaction with Prussian blue.^423^ They also proposed that the use of Prussian blue as an additive in 3D CsPbBr_3_ films can stabilize the 3D phase by preventing its transformation to other phases. In a recent work, Baranov *et al*. were able to transform Cs_4_PbBr_6_ NCs to CsPbBr_3_ NCs in a controlled way by reaction with poly(maleic anhydride-*alt*-1-octadecene) (PMAO).^424^ This polymer contains succinic anhydride units that were able to react with the oleylamine ligands bound to the surface of the Cs_4_PbBr_6_ nanocrystals, forming polysuccinamic acid, which was ultimately responsible for the transformation of Cs_4_PbBr_6_ to CsPbBr_3_. This reaction scheme is peculiar as the reaction was slow and intermediate Cs_4_PbBr_6_–CsPbBr_3_ heterostructures could be isolated. When analyzed under high-resolution transmission electron microscopy (HRTEM), clear epitaxial interfaces were identified between the two domains in individual NCs.

### Optical Features of Molecular-like Cs_4_PbBr_6_ NCs

**Figure 39:**
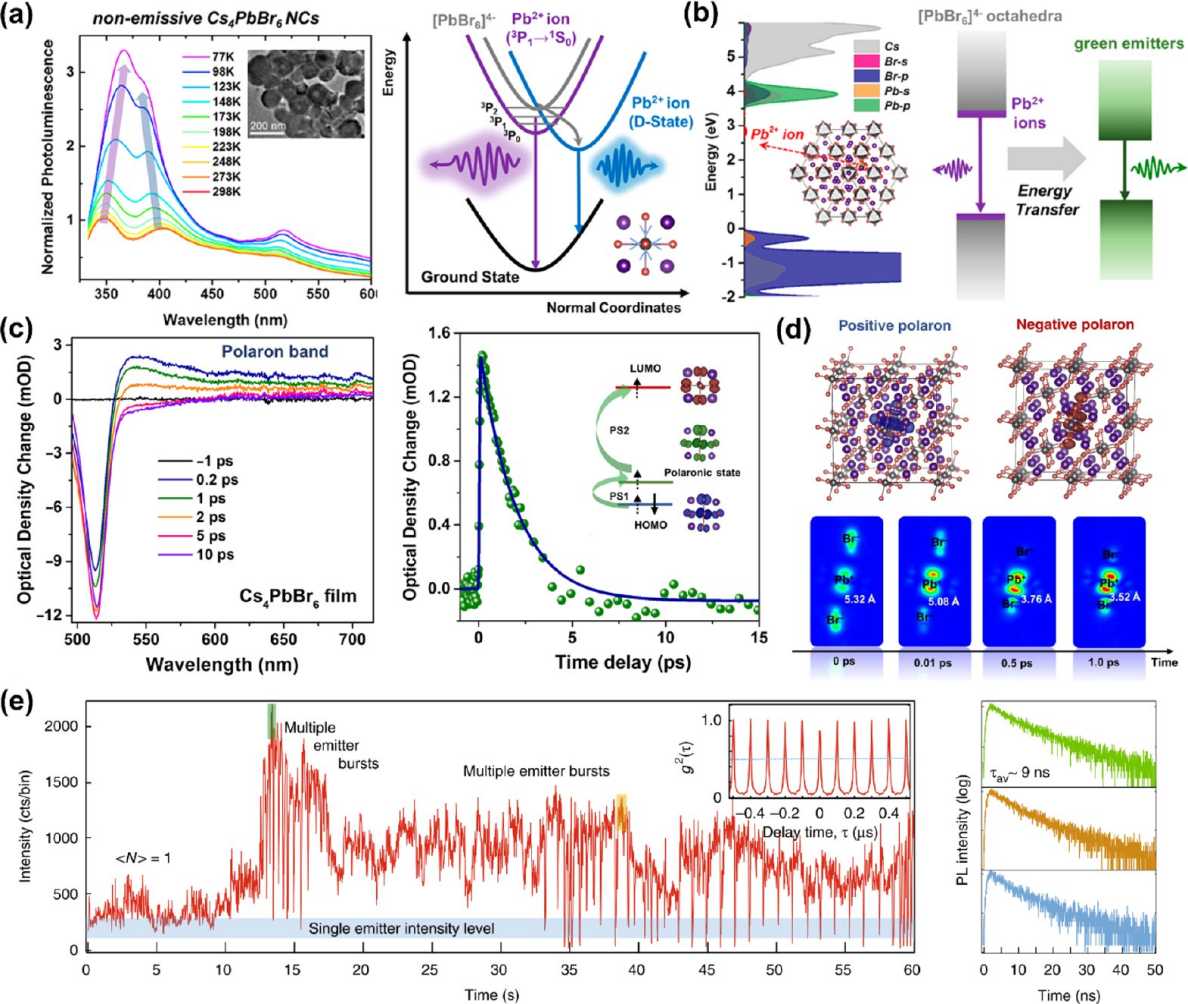
(a) Temperature-dependent PL spectra of non-emissive (non-green-emissive) Cs_4_PbBr_6_ NCs and diagram of ^3^P_1_ to ^1^S_0_ and D-state emissions from Pb^2+^ ions. Reproduced from ref (425). Copyright 2017 American Chemical Society. (b) Projected density of states of Cs_4_PbBr_6_ supercell after the replacement of a Cs^+^ with a Pb^2+^ ion and diagram of UV and visible emissions of Cs_4_PbBr_6_ NCs. Reproduced from ref (425). Copyright 2017 American Chemical Society. (c) Transient absorption spectra and photoexcitation kinetics probed at 600 nm of Cs_4_PbBr_6_ thin films. Reproduced with permission under Creative Common CC BY-NC 4.0 license from ref (426). Copyright 2017 American Association for the Advancement of Science. (d) Charge density distributions for the Cs_4_PbBr_6_ supercell with a positive/negative polaron located in the central octahedron and charge density mapping of conduction band maximum for the central octahedron at selected times. Reproduced with permission under Creative Common CC BY-NC 4.0 license from ref (426). Copyright 2017 American Association for the Advancement of Science. (e) Blinking in individual Cs_4_PbBr_6_ NCs with the emergence of multiple emitters. Reproduced with permission under Creative Common CC BY fromre (427). Copyright 2019 The Authors.

The peculiar crystal structure of 0D inorganic perovskites with isolated lead-halide octahedra enables the study of the intrinsic properties of an individual octahedron, such as intrinsic Pb^2+^ ion emission,^425^ large exciton binding energy, and polaron formation energy,^413,426^ as well as the molecular-like blinking behavior.^427^ From temperature-dependent PL spectra, as given in Figure 39a, non-green-emissive Cs_4_PbBr_6_ NCs showed spectral splitting feature in the UV range that were originated from Pb^2+^ emissions.^425^ The high-energy UV emission (around 350 nm) in the non-green-emissive NCs was attributed to the allowed optical transition of Pb^2+^ ions (*i.e.*, ^3^P_1_ to ^1^S_0_), and the low-energy UV emission (around 400 nm) was assigned to the charge-transfer state involved in the host lattice once a Cs^+^ ion was replaced by a Pb^2+^ ion (Figure 39b). In addition, the energy transfer from Pb^2+^ ions to green luminescent centers occurred in the emissive Cs_4_PbBr_6_ NCs, in addition to the broad-band UV emission.

Meanwhile Yin and co-workers underlined that Cs_4_PbBr_6_ behaves like a molecule by demonstrating its low electrical conductivity and mobility, as well as large polaron binding energy.^426^ As shown in Figure 39c, they observed an additional positive broad-band signal above 530 nm (*i.e.*, polaron absorption) in the transient absorption spectra of the Cs_4_PbBr_6_ thin film and the corresponding kinetics probed at 600 nm showed a lifetime of ∼2 ps. This confirmed the generation of small polarons with large binding energies and tight localization at individual [PbBr_6_]^4–^ octahedra. The short lifetime of the polaron state can be understood by *ab initio* molecular dynamics calculations, showing the central octahedron recovered to the neutral state after 1.2 ps stating from the initial polaronic state (Figure 39d). Thus, after photoexcitation, the structure deformation of single octahedra leads to the formation of localized polarons with short lifetime and limited transport in the Cs_4_PbBr_6_.

The molecular behavior of Cs_4_PbBr_6_ was further proved by the photon emission from individual NCs.^427^ Cs_4_PbBr_6_ NCs showed a burst-like emission behavior with a uniform distribution of PL lifetimes induced by increasing the excitation, and meanwhile exhibited a photobrightening effect because of several emissive centers within the same NC (Figure 39e). Actually, at lower excitation levels, both 3D and 0D perovskite NCs exhibited similar single-photon emission behavior, independent of their structural dimensionalities and NC size. Therefore, the emission statistics of Cs_4_PbBr_6_ and CsPbBr_3_ NCs were similar to those of individual molecular fluorophores, which are different from the traditional semiconductor quantum dots.

**Figure 40:**
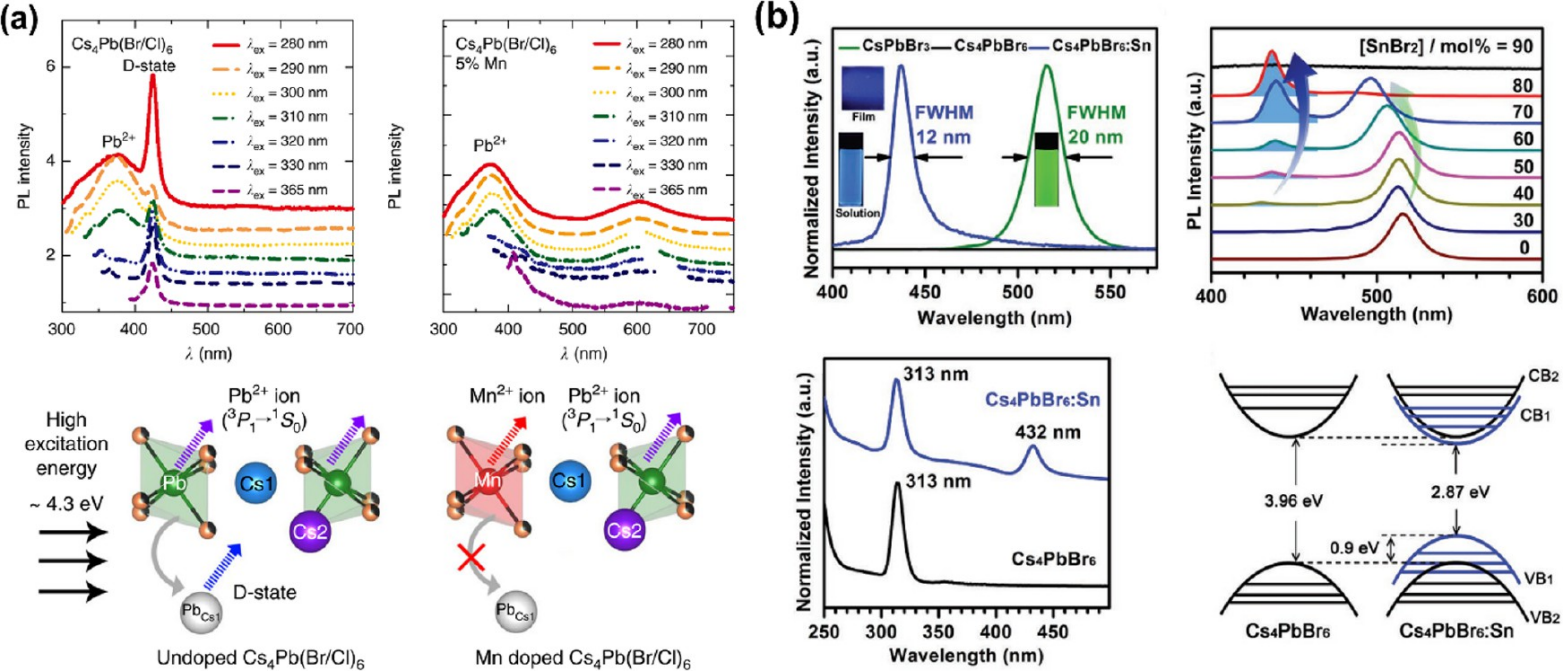
(a) PL spectra of undoped Cs_4_Pb(Br/Cl)_6_ and Mn^2+^-doped Cs_4_Pb(Br/Cl)_6_ measured at different excitation wavelengths and origin of Pb^2+^ emissions and D-state in Cs_4_Pb(Br/Cl)_6_ without and with Mn^2+^ doping. Reproduced with permission under a Creative Commons CC BY 4.0 license from ref (428). Copyright 2018 The Authors. (b) PL spectra of Cs_4_PbBr_6_ NCs, Sn-doped Cs_4_PbBr_6_ NCs, and CsPbBr_3_ NCs, and UV–vis absorption spectra of pure and Sn-doped Cs_4_PbBr_6_ perovskite NCs, together with the schematic illustration of the possible electronic dual-band-gap structure for the Cs_4_PbBr_6_ NCs before and after Sn^2+^ doping. Reproduced with permission from ref (429). Copyright 2019 John Wiley & Sons, Inc.

The intrinsic Pb^2+^ ion emissions of molecular-like 0D perovskites motivated several studies of tuning the optical emissions of Cs_4_PbBr_6_ NCs. Arunkumar and co-workers studied the optical behavior of Cs_4_PbX_6_ NCs through manganese (Mn^2+^) doping at Pb sites.^428^ They demonstrated that the incorporation of Mn^2+^ dopants can stabilize the Cs_4_PbX_6_ structure and suppress the formation of CsPbX_3_ impurities by the enhanced octahedral distortion. They also confirmed the incorporation of Mn^2+^ in the 0D Cs_4_PbX_6_ lattice by the structural characterizations, PL spectra, and PL lifetime (Figure 40a). Moreover, they achieved a high PLQY of Mn^2+^ emission in both colloidal (29%) and solid (21%, powder) forms and attributed the enhanced PLQY to the synergistic effect of structure-induced spatial confinement of Cs_4_PbX_6_ and electronically decoupled PbX_6_ octahedra. Zou *et al.* proposed another method to tailor the band gap of Cs_4_PbBr_6_ NCs to the blue spectral region by changing the local coordination environment of isolated [PbBr_6_]^4–^ octahedra in the Cs_4_PbBr_6_ through Sn^2+^ doping.^429^ Due to the distinctive Pb^2+^-poor and Br^–^-rich reaction environment, the Sn^2+^ ions can be successfully incorporated into the Cs_4_PbBr_6_ NCs, giving rise to the coexisting point defects of substitutional (Sn_Pb_) and interstitial (Br_i_) for an ultranarrow blue emission at ∼437 nm (Figure 40b). They proposed an unusual electronic dual-band-gap structure, composed of the additional band gap (2.87 eV) and original 0D band gap (3.96 eV), to be at the origin of the ultranarrow blue emission.

### Origin of Green Emission in Cs_4_PbBr_6_ NCs

**Figure 41:**
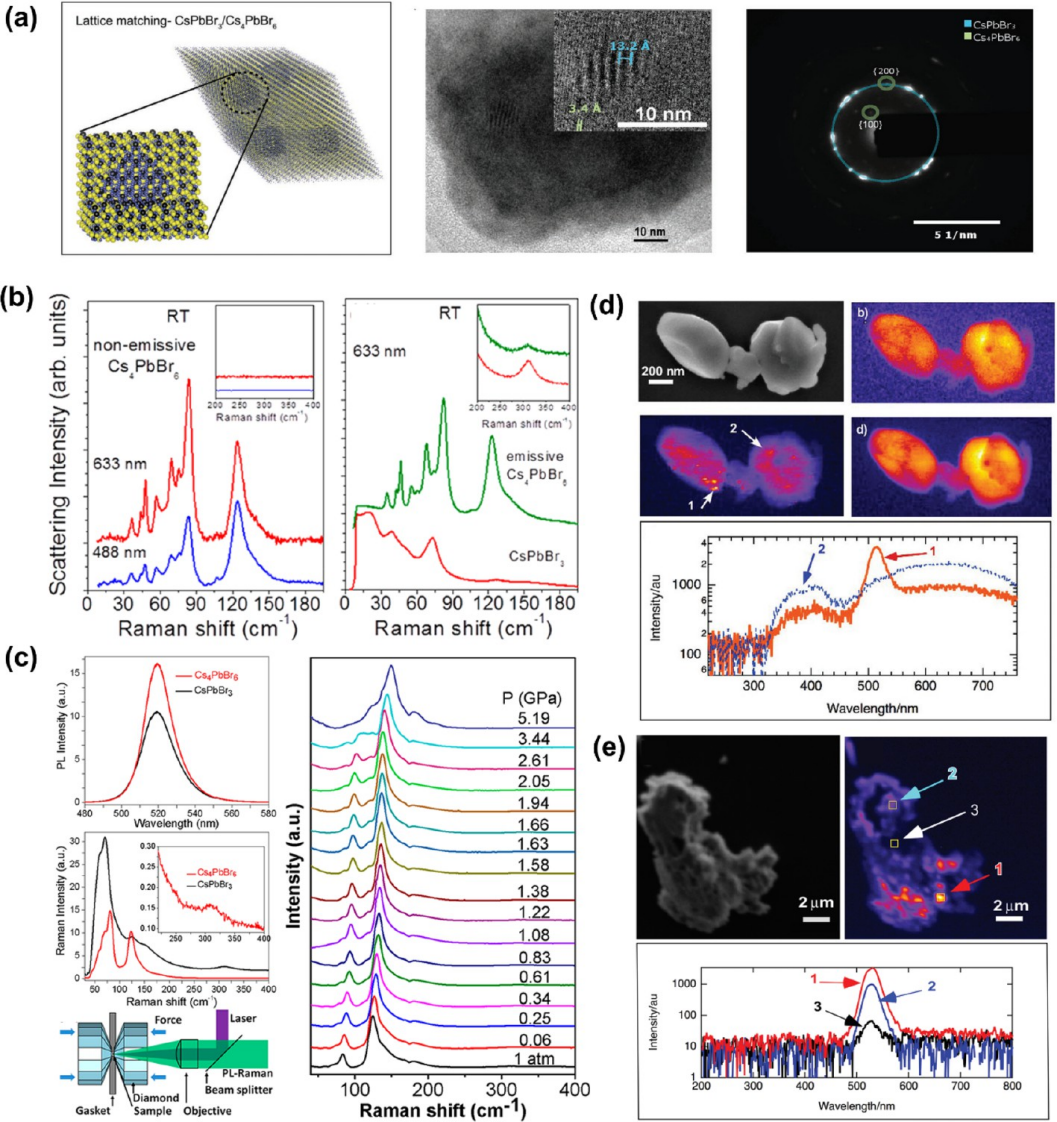
(a) Theoretical model of cubic CsPbBr_3_ perovskite NCs embedded in a matrix made of Cs_4_PbBr_6_ together with a HRTEM image of a CsPbBr_3_-in-Cs_4_PbBr_6_ crystal. Reproduced with permission from ref (430). Copyright 2017 John Wiley & Sons, Inc. (b) Raman spectra of CsPbBr_3_ microcrystals, non-emissive and emissive Cs_4_PbBr_6_ at room temperature. Reproduced from ref (431). Copyright 2019 American Chemical Society. (c) PL spectra, Raman spectra, and diamond anvil cell for confocal pressure Raman–PL and pressure evolution of Raman of emissive Cs_4_PbBr_6_. Reproduced from ref (431). Copyright 2019 American Chemical Society. (d) SEM micrograph of a particle aggregate of Cs_4_PbBr_6_ with CL band-pass images. Reproduced with permission from ref (432). Copyright 2018 Royal Society of Chemistry. (e) SEM micrograph of Cs_4_PbBr_6_ aggregate with CL image and CL spectra for three regions. Reproduced with permission from ref (432). Copyright 2018 Royal Society of Chemistry.

Although the molecular-like quantum optoelectronic behavior of Cs_4_PbBr_6_ NCs is well-studied, the origin of their green emission is still not clear. Several emission mechanisms have been proposed in the literature, including the embedded 3D CsPbBr_3_ impurities, intrinsic point defects, and 2D Cs_2_PbBr_4_ inclusion. For instance, Quan *et al.* confirmed the efficient green-emitting CsPbBr_3_ NCs were embedded in air-stable Cs_4_PbBr_6_ microcrystals, *i.e.*, the coexistence of NCs and the matrix, by powder XRD, high-resolution transmission electron microscopy (HRTEM) and scanning electron microscope (SEM) imaging (Figure 41a).^430^ They suggested the lattice matching between the CsPbBr_3_ NCs and the Cs_4_PbBr_6_ matrix contributed to improved passivation and such spatial confinement can enhance the radiative rate of the NCs. Recently, Qin *et al.* also suggested the presence of CsPbBr_3_ impurities in Cs_4_PbBr_6_ by identifying the Raman difference between emissive and non-emissive Cs_4_PbBr_6_. They found that the Raman spectrum of emissive Cs_4_PbBr_6_ was identical to that of the non-emissive case, but it contains an additional Raman band at ∼29 cm^–1^ that replicated the doublet at 28-30 cm^–1^ of CsPbBr_3_ (Figure 41b).^431^ The concentration of CsPbBr_3_ was estimated to 0.2% in volume and this was below the typical XRD sensitivity. They observed a fast red-shifting, diminishing, and eventual disappearance feature of green emission by employing a diamond anvil cell to probe the response of luminescence centers to hydrostatic pressure (Figure 41c). This can help exclude the Br vacancies as the luminescent centers. Riesen *et al.* concluded that the green emission from Cs_4_PbBr_6_ is due to nanocrystalline CsPbBr_3_ impurities using cathodoluminescence imaging and energy-dispersive X-ray (EDX) measurements.^432^ The CL imaging and spectroscopy showed the presence of small crystals embedded in between larger crystallites of Cs_4_PbBr_6_ which emitted around 520 nm (Figure 41d). EDX showed that the smaller crystal inclusions have a Pb/Br ratio that was approximately two times higher, confirming the CsPbBr_3_ phase (Figure 41e).

**Figure 42:**
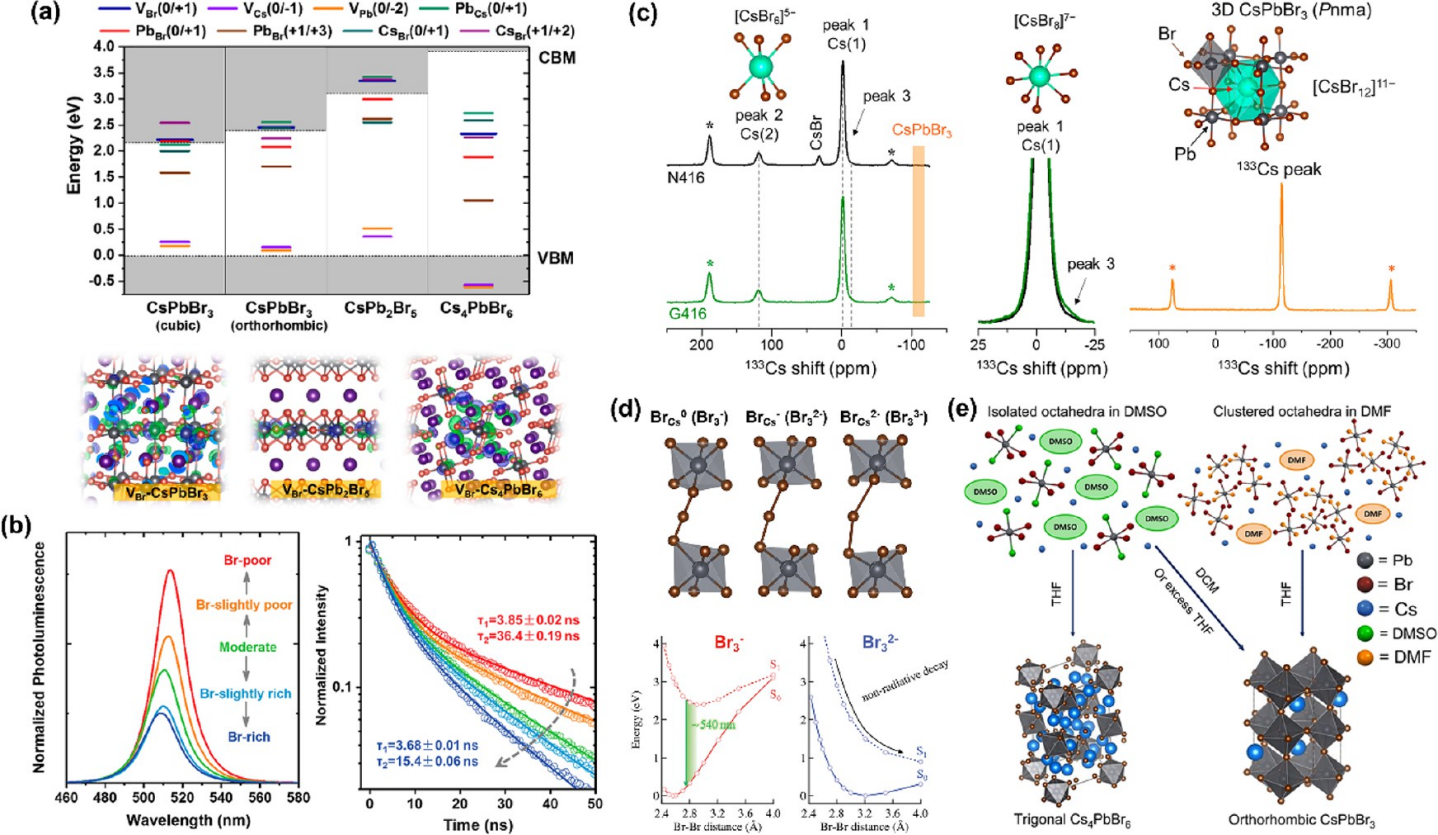
(a) Calculated defect charge transition levels and charge density distributions of V_Br_ defect states for CsPbBr_3_, CsPb_2_Br_5_, and Cs_4_PbBr_6_. (b) Normalized PL spectra and time-resolved PL spectra of Cs_4_PbBr_6_ NCs under different growth conditions. Reproduced from ref (433). Copyright 2018 American Chemical Society. (c) ^133^Cs MAS NMR spectra and corresponding magnified spectra (25 to −25 ppm) of non-emissive and green-emissive Cs_4_PbBr_6_ obtained at 9.4 T and a spinning rate of 10 kHz at 300 K, together with ^133^Cs MAS NMR spectra of CsPbBr_3_ perovskite crystal. Reproduced from ref (434). Copyright 2020 American Chemical Society. (d) Local structure associated with point defect species in Cs_4_PbBr_6_ and relative potential energy surfaces of ground state S_0_ and first excited state S_1_ as a function of the Br–Br distance for Br_3_^–^ and Br_3_^2–^. Reproduced with permission from ref (435). Copyright 2019 Royal Society of Chemistry. (e) Schematic diagram of the effect of the solvodynamic size and solvent–antisolvent pair on the formed CsPbBr_3_ and Cs_4_PbBr_6_ phases. Reproduced under a Creative Commons CC-BY-NC-ND license from ref (436). Copyright 2019 American Chemical Society.

Many other groups have argued that the green emission of Cs_4_PbBr_6_ is not from CsPbBr_3_ impurities but an intrinsic property of Cs_4_PbBr_6_ because of (*i*) the absence of diffraction peak and pattern of CsPbBr_3_, (*ii*) the failure of halogen exchange, and (*iii*) no match of the emission peak for the small-size CsPbBr_3_ NCs. Yin *et al.* have demonstrated that the bromide vacancy (V_Br_) of Cs_4_PbBr_6_ has a low formation energy and is a relevant defect level that contributes to the green emission.^433^ As shown in Figure 42a, in the Pb-rich/Br-poor condition, V_Br_ was the dominant defect and had a transition level energy of ∼2.3 eV located above the valence band maximum (VBM); Pb- and Cs-related vacancies showed a deep transition level (−0.5 eV below the VBM), and the other antisites all had deep transition levels within the band gap. To confirm the green emission from V_Br_ point defects, they synthesized Cs_4_PbBr_6_ NCs under different conditions by controlling the HBr amount, and found the PL intensity increased when increasing the concentration of Br defects and the highest PLQY was achieved in Br-deficient Cs_4_PbBr_6_ NCs (Figure 42b). Moreover, their state-of-the-art characterizations including HRTEM further confirmed the purity of the 0D phase of Br-deficient green-emissive Cs_4_PbBr_6_ NCs and also excluded the presence of CsPbBr_3_ NCs impurities. The theory concerning the inclusion of Br defects was recently supported by Cha and co-workers based on the characteristic magnetic behavior of non-emissive and green-emissive Cs_4_PbBr_6_ perovskite crystals.^434^ They demonstrated the presence of defects in green-emissive Cs_4_PbBr_6_ and the extremely low concentration of a CsPbBr_3_ phase in both non-emissive and green-emissive crystals based on the analysis of ^133^Cs magic-angle-spinning (MAS) NMR spectra (Figure 42c).

Jung and co-workers have a different theoretical view about defect properties of Cs_4_PbBr_6_.^435^ They showed that the Br_Cs_ defects led to the formation of molecular Br_3_-type species that exhibited a range of optical transitions in the visible range, and the green luminescence can be from the emission of optically excited Br_3_ to its ground state. Based on the analysis of the lowest-lying electronic excitation energy as a function of the Br–Br distance (Figure 42d), they found Br_3_^–^ and Br_3_^2–^ provide S_1_–S_0_ energy differences in the range of green emission (∼2.3 eV). They suggested the presence of a radiative mechanism with visible-light emission in Br_3_^–^ molecular species that could contribute to the green emission in Cs_4_PbBr_6_ upon tribromide defect formation.

Ray and co-workers proposed that a 2D Cs_2_PbBr_4_ inclusion may be responsible for the green emission of Cs_4_PbBr_6_ NCs although they found no conclusive experimental evidence supporting this claim.^436^ They found the solvodynamic size of the lead bromide species played a critical role in determining the Cs–Pb–Br composition of the precipitated powders, *i.e.*, the smaller species favored the precipitation of Cs_4_PbBr_6_ and larger species favored the formation of CsPbBr_3_ under the same experimental conditions (Figure 42e). Therefore, Cs_4_PbBr_6_ has a higher tendency to be precipitated out from solutions with stronger coordinating solvents to Pb^2+^, lower absolute concentration of the precursors, and higher CsBr/PbBr_2_ ratios, as compared to 3D CsPbBr_3_ counterpart. They concluded that 3D impurities might not be the only source of the emission and high PLQY and proposed an impurity of 2D Cs_2_PbBr_4_ may also contribute to the green emission.

### Optoelectronic Applications of Cs_4_PbBr_6_ Microcrystals and Nanocrystals

**Figure 43:**
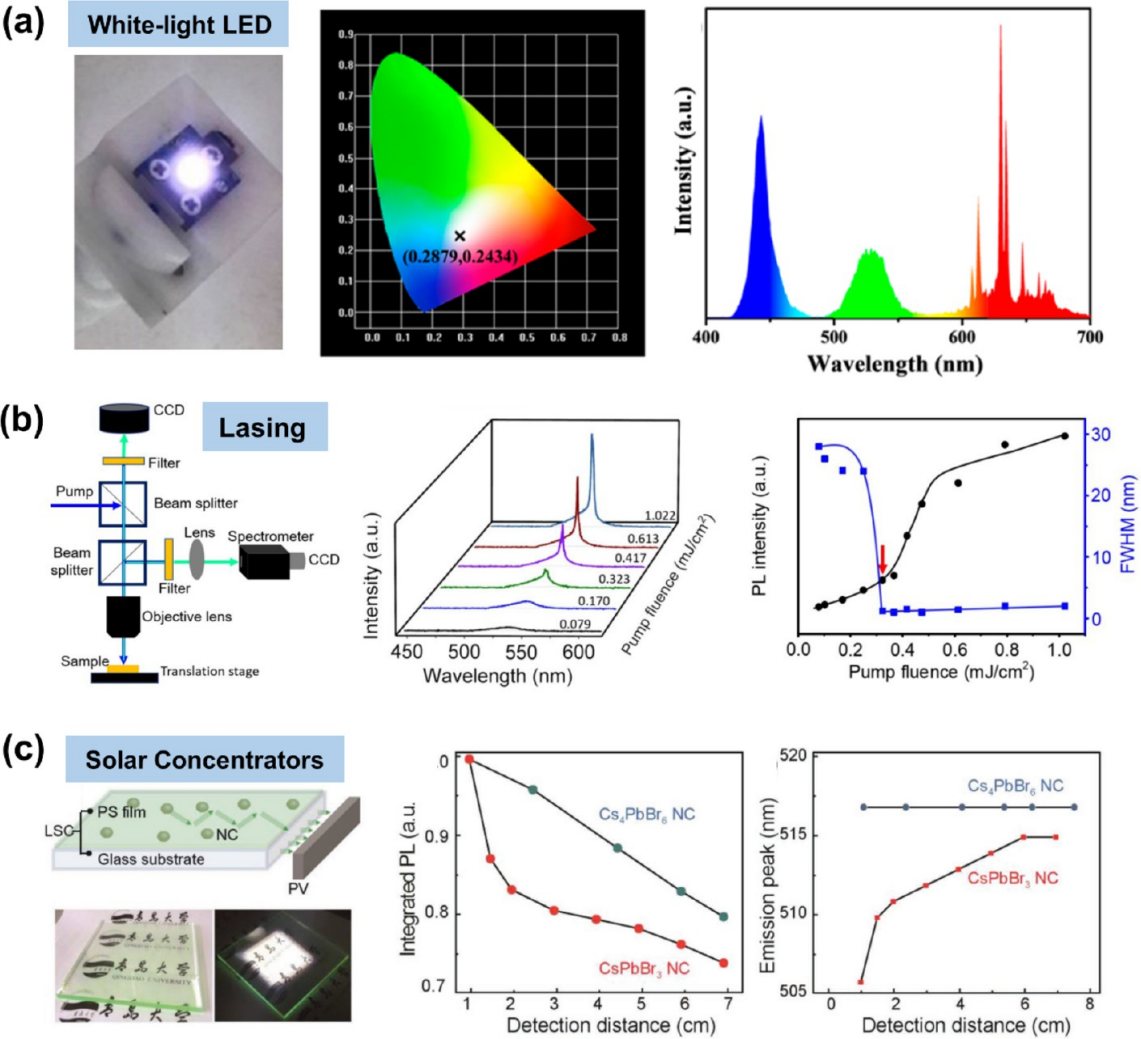
(a) Photographs of LED devices fabricated with Cs_4_PbBr_6_ MCs and K_2_SiF_6_:Mn^4+^ phosphor, color coordinates of the white LEDs, and electroluminescent spectra of the white LED. Reproduced from ref (437). Copyright 2018 American Chemical Society. (b) Schematic of the micro-PL setup for imaging and detection of the PL signal of an individual Cs_4_PbBr_6_ microcrystal and evolution of the PL spectra with the pump fluence and dependence of the PL intensity and fwhm on the pump fluence. Reproduced from ref (438). Copyright 2018 American Chemical Society. (c) Scheme of an LSC and photographs of the LSC comprising perovskite NCs under ambient and one sun illumination and integrated PL intensity and emission peak positions as a function of detection distance. Reproduced with permission from ref (439). Copyright 2019 John Wiley & Sons, Inc.

Despite the unclear origin of green emission in Cs_4_PbBr_6_ NCs, the high PL intensity and PLQY of Cs_4_PbBr_6_ NCs make them interesting for the applications in the optoelectronic devices.^440^ Bao *et al.* reported a synthesis method to obtain highly stable Cs_4_PbBr_6_ microcrystals (MCs) using a microfluidic system.^437^ They incorporated Cs_4_PbBr_6_ MCs with K_2_SiF_6_:Mn^4+^ phosphor onto InGaN blue chips to fabricate the white light-emitting diodes (Figure 43a). The white LED device exhibited a wide color gamut of 119% of National Television Standards Committee (NTSC) standard and a luminous efficiency of 13.91 lm/W. Sun *et al.* developed an antisolvent approach to obtain the phase-pure Cs_4_PbBr_6_ MCs exhibiting intense PL centered at 518 nm with a PLQY of ∼30% and a large binding energy of 267 meV.^438^ They revealed the agreement between the PL excitation spectrum and localized optical absorption of Pb^2+^ in isolated [PbBr_6_]^4–^ octahedra and confirmed that the green emission was an intrinsic feature of Cs_4_PbBr_6_. Moreover, they demonstrated the single- and multimode lasing resonances in individual Cs_4_PbBr_6_ MCs by optical pumping, showing a high photostability even upon rather intense optical pumping (Figure 43b). Cs_4_PbBr_6_ NCs can be used in luminescent solar concentrators (LSCs) (Figure 43c) absorber as they meet the requirements of small absorption/emission spectral overlap, high PLQY, robust stability and ease of synthesis. Zhao and co-workers fabricated semitransparent large-area LSCs using Cs_4_PbBr_6_ NCs and the optimized LSCs exhibited an external optical efficiency of 2.4% and a power conversion efficiency of 1.8% (100 cm^2^).^439^ These results suggest that 0D perovskite MCs and NCs are promising candidates for high-efficiency optoelectronic devices covering a similar application sphere as 3D perovskite NCs.

## Surface Coating Strategies for Stability Improvement

**Figure 44:**
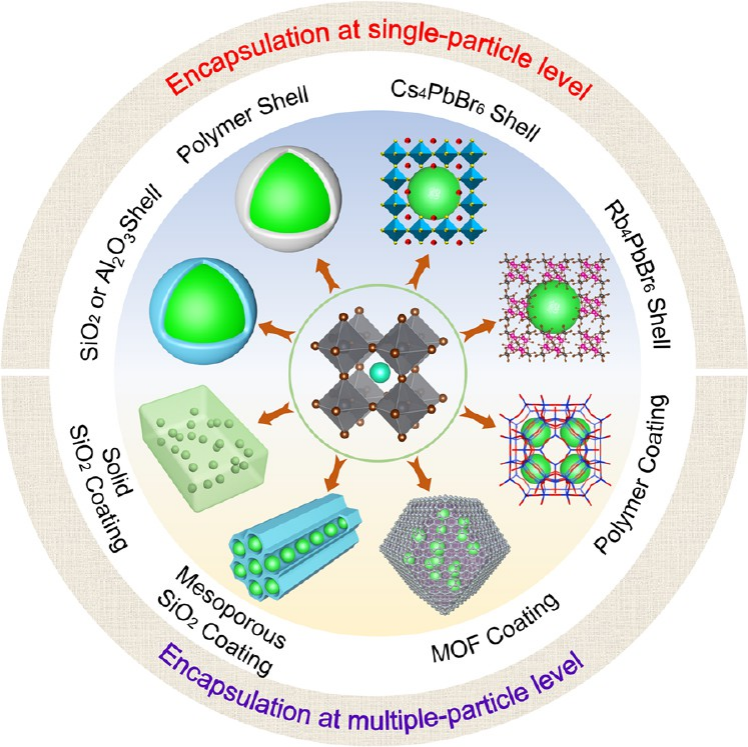
Schematic overview of different types of shell materials employed for coating on perovskite NCs to improve their stability toward heat, moisture, water, and other environmental stresses.

Considering the intrinsic ionic nature,^14,417,441^ the durability of MHP NCs against moisture, oxygen, light and high temperatures is still a significant challenge that has limited their further development and practical applications. Over the years, significant studies have been devoted to the encapsulation of perovskite NCs in various materials either in the form of core–shell NCs or NCs in a matrix as illustrated in Figure 44. The encapsulation process can be carried out by either *in situ* synthesis or post-synthesis surface coating. Encapsulation by inert materials has proven to be a feasible and effective approach to prevent the decomposition and enhance stability, enabling them to survive under water/photo/thermal treatment.^278,329,442−446^ It has been reported that CsPbX_3_ NCs have a high defect-tolerance,^23^ however, the surface traps that probably assist the nonradiative process are still non-negligible.^88^ In addition to surface passivation with molecular ligands, a suitable encapsulation strategy (Figure 44) can also efficiently remove or fix the quenching sites located on the surface, and thus suppress the nonradiative recombination.^301^ Hence, the encapsulation always improves the photophysical properties of MHP NCs owing to the significant passivation effect.^447,448^ In addition, encapsulation also protects against reactive oxygen species.^449^ Furthermore, the energy- and charge-transfer process within MHP NCs can also be tuned with semiconductor shells on their surface. In some cases, brighter PL emission can be achieved by the introduction of wider-gap semiconductors to fabricate type-I composite. In this type, the foreign semiconductor shell has a higher conduction band and a lower valence band compared to CsPbX_3_, leading to confinement of photogenerated carriers.^364,427,430,450−452^ On the contrary, PL quenching occurs in type-II heterostructure when the band gap of CsPbX_3_ NCs overlaps with another semiconductor, favoring the charge diffusion, transfer, and finally separation.^365,427^ Due to the distinctly different carrier performance, these heterostructures with type-I and type-II can be applied in LEDs and photocatalysis, respectively. Despite recent progress in the synthesis of perovskite NCs, further advances in stability enhancement, surface passivation, and charge confinement/separation endowed by encapsulation are still necessary to advance the filed perovskite NCs toward commercial optoelectronic applications. Different strategies for encapsulating perovskite NCs to enhance their stability are discussed below. It should be noted that the conditions (*e.g*., concentration, whether the NCs are in colloidal solution or in powder form, temperature, solvent, time, ligand density, shell thickness in the case of core–shell NCs) used for the comparison of the stability of perovskite NCs is different in different studies. Therefore, the discussion is mainly limited to a specific example in each case.

### Encapsulation at Multiple-Particle Level

Despite the on-going intensive efforts and a plethora of conducted studies, metal-halide perovskite NCs are still suffering from rather poor stability against many common factors such as oxygen, humidity, light illumination, and heat. The identification of suitable encapsulation of perovskite NCs is thus an ongoing task, and several types of protective materials have been suggested, such as silica, organic polymers, metal oxides, metal salts, *etc.*

Silica coating of conventional semiconductor quantum dots (*i.e*, CdSe QDs) has become well-established and often used for MHP NCs as well, due to the nontoxic nature, mechanical robustness, high thermal stability, and good optical transmission of this material.^453,454^ However, as the conventional hydrolysis process to form SiO_2_ shell needs some amount of water, this may appear detrimental for the stability and optical properties of perovskites. Overall, the use of silica encapsulation strategy for MHP NCs requires the right balance between the hydrolysis rate and the ability to form compact and dense SiO_2_ protective shells. There have been few attempts to encapsulate MHP NCs in silica matrix using traditional precursors tetraethyl orthosilicate (TEOS)^455,456^ and octadecyltrimethoxysilane,^457^ while other precursors such as tetramethyl orthosilicate^458^ and APTES^445^ with higher hydrolysis rate were employed to enable the faster formation of a SiO_2_ protective layer under the assistance of a trace amount of water, in which perovskite NCs are able to withstand.^459^ The latter silicate precursors enable to maintain the original high PLQY and narrow PL emission of both organic–inorganic (methylammonium-based) and all-inorganic (cesium-based) lead-halide perovskite NCs for a longer time: for example, the APTES shelled CsPb(Br/I)_3_ NCs maintained 95% PLQY after 3 months of storage.

**Figure 45:**
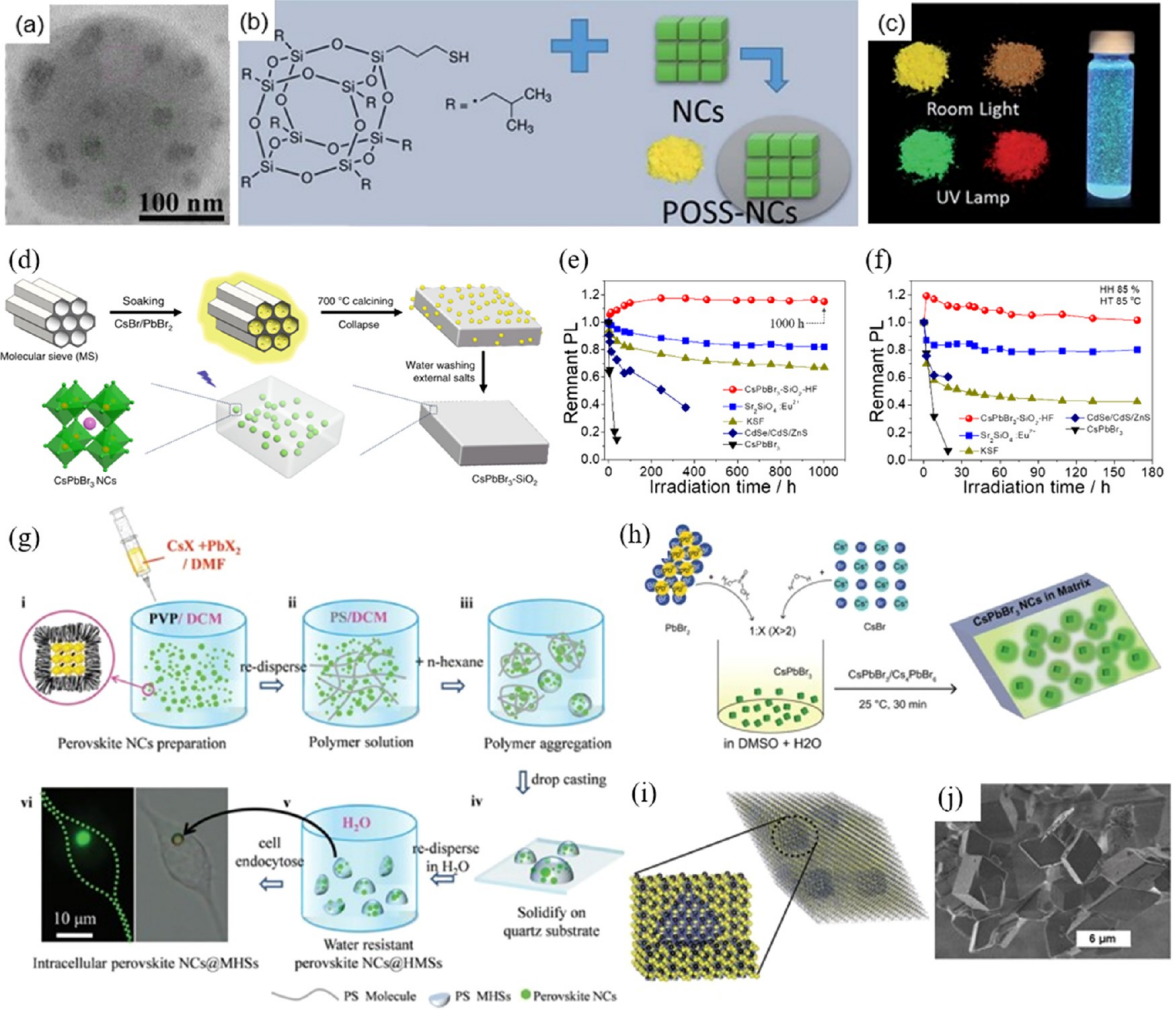
(a) HAADF-STEM image of several CsPbBr_3_ NCs embedded within a mesoporous silica sphere. Reproduced with permission from ref (460). Copyright 2017 Royal Chemical Society. (b) Structure of thiolated polyhedral oligomeric silsesquioxane (POSS) and illustration of the coating process of POSS on presynthesized perovskite NCs. (c) Photographs of POSS-coated green-emititng CsPbBr_3_ and red-emititng CsPb(Br/I)_3_ powders under room light and UV light and a dispersion of green-emitting POSS-CsPbBr_3_ NCs in water. Panels b and c reproduced with permission under a Creative Commons CC BY 3.0 license from ref (462). Copyright 2016 Royal Chemical Society. (d) Schematic diagram of synthesis CsPbBr_3_ NCs into dense SiO_2_ by high-temperature solid-state reaction. (e) Photostabilities of the CsPbBr_3_–SiO_2_, ceramic Sr_2_SiO_4_:Eu^2+^ green phosphor, KSF red phosphor, colloidal CsPbBr_3_ NCs, and CdSe/CdS/ZnS NCs under illumination, sealed with Norland-61 on the LED chips (20 mA, 2.7 V), (f) aged at 85 °C and 85% humidity conditions on the LED chips (20 mA, 2.7 V). Panels d–f are reproduced with permission from ref (283). Reprinted with permission under a Creative Commons CC BY license. Copyright 2020 The Authors. (g) Schematics of encapsulation of CsPbBr_3_ NCs into a PVP matrix resulting in water-resistant composites used for the intracellular imaging. Reproduced with permission from ref (442). Copyright 2017 John Wiley & Sons, Inc. (h) Schematics of one-pot synthesis of CsPbBr_3_-in-Cs_4_PbBr_6_ microcrystals from CsBr and PbBr_2_ precursors. (i) Crystal structure model for composites synthesized in (h), with a Cs_4_PbBr_6_ microcrystal in a rhombic prism shape hosting several CsPbBr_3_ NCs. (j) SEM image of CsPbBr_3_-in-Cs_4_PbBr_6_ prism-shaped microcrystals. Reproduced with permission from ref (430). Copyright 2017 John Wiley & Sons, Inc.

The incorporation of multiple perovskite NCs inside mesoporous silica spheres has been demonstrated to be a good option to improve their thermal stability and photostability, with a final aim to enhance the device performance.^444,460^ High-angle annular dark-field scanning transmission electron microscopy (HAADF-STEM) was used to confirm the presence of several CsPb_2_Br_5_ NCs in an individual mesoporous silica particle, as shown in Figure 45a. These samples were used to fabricate white light-emitting devices (WLED).^460^ Superhydrophobic sponge-like silica aerogels acted as a scaffold to accommodate CH_3_NH_3_PbBr_3_ NCs and could well-preserve both the structure and optical properties of these perovskites due to their amorphous phase and high optical transparency.^461^ This system was then demonstrated to serve as a sensitive fluorescence SO_2_ gas sensor with a reversible quench-and-recovery in the emission response.^461^

Some other silica-related compounds have been explored as well to protect lead-halide perovskite NCs from water and humid environment. Polyhedral oligomeric silsesquioxane with a cage-like structure and functional thiol group able to coordinate with the surface of CsPbBr_3_ NCs (Figure 45b) was used as efficient encapsulating material able to protect these perovskites from water.^462^ The encapsulated powdered samples kept their emission as a dispersion in water for more than 10 weeks, as shown in Figure 45c, and also prevented mixed-anion (Br/I) perovskite powders from ion exchange, thus enabling their use as light-emitting layers in down-conversion WLEDs.^462^

For the conventional hydrolysis process to form a SiO_2_ shell, the involved water can cause irreversible damage to the CsPbX_3_ NCs. On the other hand, the densification extent of SiO_2_ produced by the hydrolysis process is not enough to prevent the penetration of water to the inner CsPbX_3_ NCs. To increase the densification extent of SiO_2_ and improve the stability of CsPbX_3_ NCs, a high-temperature annealing process can promote more densely cross-linked structure of SiO_2_, but the annealing temperature could not exceed 100 °C due to the severe surface oxidation or fusing of CsPbX_3_ NCs. In view of this, Zhang *et al*.^283^ proposed a facile strategy to synthesize ceramic-like stable and highly luminous CsPbBr_3_ NC through template-confined solid-state synthesis and *in situ* encapsulation based on the strategic disintegration of silicon molecular sieve (MS) templates at high temperatures (Figure 45d). The synthesis process is a solid-state reaction at high temperature without organic solvents and organic ligands. Due to the encapsulation of dense SiO_2_ at high temperature (500–800 °C), the as-prepared CsPbBr_3_–SiO_2_ powders exhibited comparable operation stability as the commercial ceramic phosphors (Figure 45e,f).^283^ In addition, a high-temperature solid-state reaction has been used to crystallize CsPbX_3_ NCs in glasses, and the obtained CsPbX_3_ NCs encapsulated with glasses present high PLQY and robust stabilities to moisture, temperature, and UV light irradiation.^463−465^ In a recent work, An *et al.*(466) have been able to grow CsPbBr_3_ NCs inside the pores of mesoporous silica using a molten salt approach at temperatures as low as 350 °C. The specific combination of salts enabled at the same time a high PLQY and a sealing of the pores, such that the NCs were effectively isolated from the external environment. A clear proof of the stability of these composites was given by the preservation of their emission properties even if they were immersed in aqua regia for several weeks.

The encapsulation within an organic (especially, hydrophobic) polymer hosts is yet another popular choice to improve the resistance of perovskite NCs toward harmful environments such as moisture and oxygen. Zhang *et al.* demonstrated successful encapsulation of CsPbBr_3_ NCs using polyvinylpyrrolidone (PVP) and used them as luminescent probes for intracellular imaging in an aqueous environment, as illustrated in Figure 45g.^442^ In addition to PVP, a number of other polymer matrices, including polystyrene (PS),^467^ polycarbonate (PC),^386^ polyurethane,^468^ PMMA,^73^ poly(lauryl methacrylate),^469^ and ethylene vinyl acetate^470^ were employed as protective coatings for perovskite NCs. The protection strategies for perovskite NCs employing those different polymers can be classified into two major categories: *in situ* fabrication from suitable precursors, and post-preparative encapsulation of presynthesized perovskite nanoparticles (the previously mentioned POSS encapsulation technology^462^ belongs to the latter one, as shown in Figure 45b). Within the former strategy, Hintermayr *et al.* used nanocavities formed by amphiphilic block copolymer PS-*b*-P2VP (a combination of hydrophilic P2VP part and hydrophobic PS) which provided a suitable space for the spontaneous nucleation of perovskite precursors.^471^ Core/shell micelles serving as nanoreactors for the *in situ* formation of perovskite NCs were obtained upon the introduction of antisolvents such as toluene and were composed from the P2VP part as a core and the PS as an outer shell. However, the use of the polymer-coated perovskite composites in LEDs may be problematic, as large applied voltages and unavoidable heat generation during the device operation may induce polymer degradation and thus the emission quenching or undesirable shifts.

Capitalizing upon the previous experience with conventional semiconductor QDs,^472−474^ metal oxides such as alumina (Al_2_O_*x*_), TiO_*x*_, and ZnO have been recently applied to shelter perovskite NCs. Atomic layer deposition and wet-chemical template method are two major fabrication routes for metal oxide deposition,^475−477^ while the high temperature used in the annealing process may be an issue resulting in undesirable decomposition of perovskite NCs. It has been reported that the decomposition of CsPbBr_3_ NCs could happen when porous TiO_2_ matrix was annealed at just 85 °C.^365,478^ However, CsPbX_3_ NCs synthesized by the template confined solid synthesis in mesoporous Al_2_O_3_ at 800 °C showed high PLQY and outstanding thermal stability beyond to 300 °C.^479^ Metal-organic frameworks (MOFs) composed of metal ions and bridging organic ligands were also considered as a matrix able to protect and preserve the emission of perovskite NCs in hostile environments.^480−482^ The tunable size and shape of the pores in MOFs and the ability to modify their surface through functional groups enabled their use as smart materials in anti-counterfeiting applications.^483,484^ For instance, Zhang *et al.*(484) demonstrated that the PL of MAPbBr_3_ perovskite NCs in the pores of MOFs can be reversibly switchable (quenched and recovered) by treatment with water and MABr, and thus this process can be used for multiple encryption and decryption of information.

Furthermore, metal-halide salts have been shown to be able to serve as a protective matrix for improving the chemical stability of perovskite NCs,^485,486^ which was inspired from the original work of Eychmüller and co-authors on protecting conventional QDs through the use of such kind of salt matrices.^487−489^ Dirin *et al.* reported a two-step synthesis, in which first nucleation followed by a shelling process to deposit inorganic NaBr shells around multiple CsPbBr_3_ NCs.^490^ Perovskite precursors firstly crystallized on the surface of microsized alkali halides, followed by a coating process driven by surface reaction of amphiphilic Na and Br precursors in nonpolar solvents. A series of other alkali halides including MgX_2_, CaX_2_, SrX_2_, BaX_2_, and ZnX_2_ were tested as well to validate the general applicability of this method.^490^

The combinations of two different semiconductor materials to form core/shell heterostructures have been widely demonstrated for different II–VI, IV–VI, and III–V QDs, where the trap states could be removed and the stability improved.^67,491^ However, the synthetic strategies used for those QDs were not easy to be translated toward lead-halide perovskite NCs, eventually due to their more dynamic surface and lower melting points. CsPbX_3_/ZnS QDs with a heterojunction-like structure were reported, yet only a partial decoration of the surface of CsPbX_3_ NCs with ZnS has been achieved.^492^ Cs_4_PbX_6_ is an insulating material with a wide band gap of 3.9 eV,^493^ and smaller CsPbBr_3_ NCs encapsulated inside a Cs_4_PbBr_6_ matrix were found to preserve high PLQY and thus could be used as optical gain materials in lasers and as emissive layers in LEDs.^364,430^Figure 45h illustrates a one-pot preparation of Cs_4_PbBr_6_-in-CsPbBr_3_ composites from suitable precursors in a liquid environment, while Figure 45i shows lattice alignment of CsPbBr_3_ NCs within the Cs_4_PbBr_6_ matrix; well-faceted Cs_4_PbBr_6_-in-CsPbBr_3_ microprisms are visualized by SEM image in Figure 45j.^430^ More recently, Cao *et al.* demonstrated the use of the CsPbX_3_-in-Cs_4_PbX_6_ composites for X-ray sensing and imaging, with the Cs_4_PbBr_6_ matrix providing a favorable enhancement in the attenuation of X-rays.^494^

### Encapsulation at Single-Particle Level

From the aforementioned encapsulation strategies, a variety of materials including polymers, SiO_2_, and AlO_*x*_ have been employed to stabilize CsPbX_3_ NCs, resulting in impressive stability improvement. The capsule-like structure endowed CsPbX_3_ NCs with enhanced optical properties accompanied by exceptional stability. In these successful encapsulation examples, however, the as-obtained products always had multiple particles in one shell, resulting in large particle size that could reach up to tens of micrometers. In general, the CsPbX_3_ NCs used in optoelectronic devices are assembled in the form of a film, in which undesirable large particles would weaken the film quality and consequently corresponding device performance. In addition to the poor uniformity in film, micrometer-sized particles were unfavorable in many bio-related areas, such as cell uptake. More importantly, the large particle size hampers their solution processability. The straightforward solution for this problem is to shrink the size of the encapsulated CsPbX_3_ product down to the nanoscale. Significant efforts have been devoted to exploring the encapsulation of CsPbX_3_ NC at single-particle level. Up to now, oxides and semiconductors have been employed in the fabrication of CsPbX_3_-based core/shell nanostructures with significantly improved optical properties and stability.

**Figure 46:**
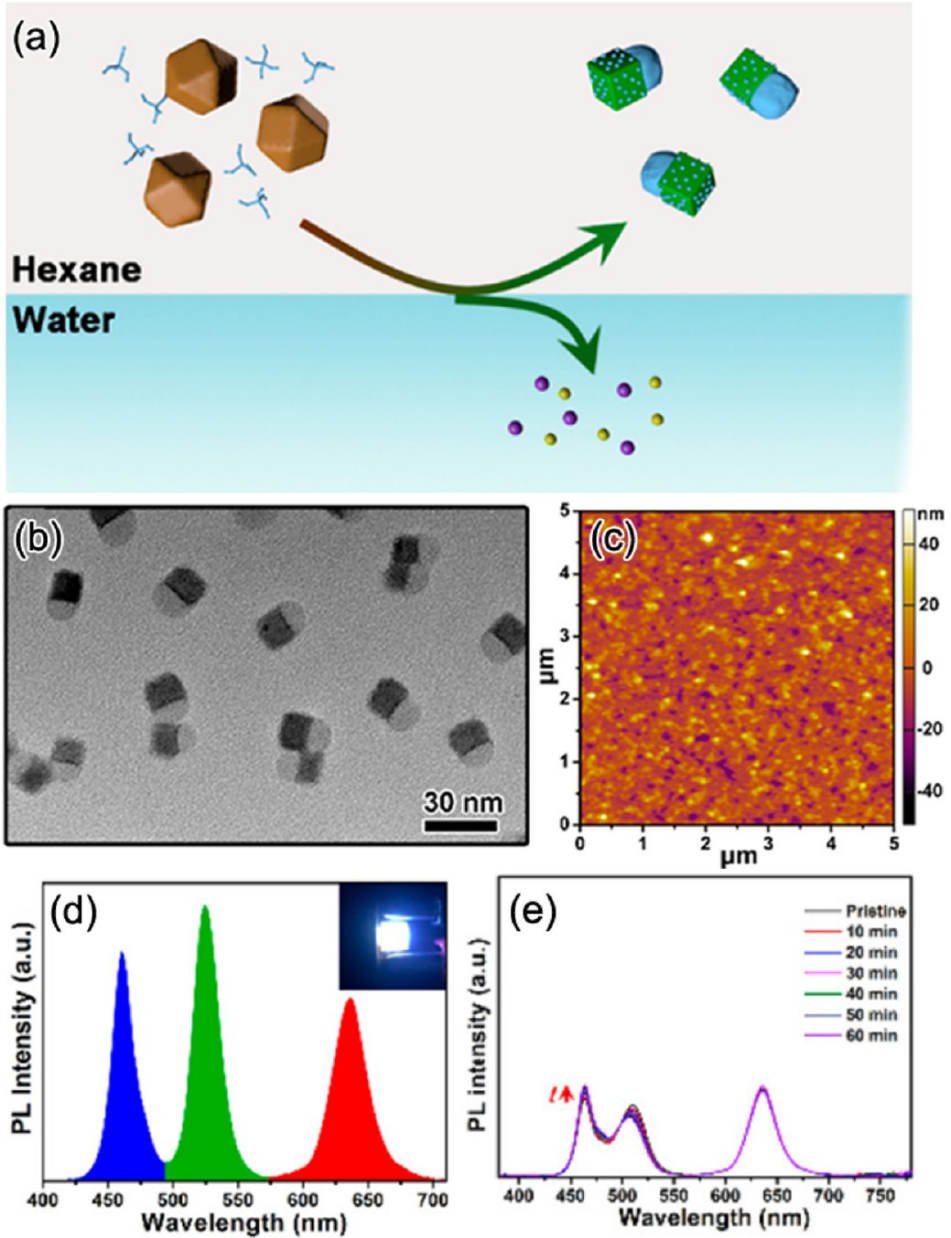
(a) Schematic illustration of the formation of CsPbX_3_/SiO_2_ Janus nanoparticles. (b) TEM image of CsPbBr_3_/SiO_2_ Janus NCs. (c) AFM image of the film fabricated from the CsPbBr_3_/SiO_2_ nanoparticles. (d) PL spectra of CsPbBr_3_/SiO_2_-based WLED device and (e) its corresponding time-dependent PL spectra. Inset in (d) shows an operating device. Reproduced from ref (495). Copyright 2017 American Chemical Society.

In 2017, Hu *et al.*(495) developed a sol-gel method to produce monodispersed CsPbX_3_/SiO_2_ Janus NCs at the oil–water interface. The simultaneous transformation Cs_4_PbX_6_ → CsPbX_3_ and growth of SiO_2_ at one side of CsPbX_3_ NCs led to the formation of a distinctive Janus structure, as shown in Figure 46a,b. As expected, the modification of SiO_2_ ensured CsPbX_3_ NCs fewer surface traps and enhanced photophysical properties. More importantly, CsPbX_3_/SiO_2_ Janus NCs could be fabricated into a high-quality film, which exhibited comparable smoothness and uniformity to pristine CsPbX_3_ NCs, as shown in Figure 46c. In addition, CsPbBr_3_/SiO_2_ Janus nanoparticles could be employed as an emitting layer in a WLED, resulting in a significantly improved photostability under continuous UV irradiation (Figure 46d,e).

**Figure 47:**
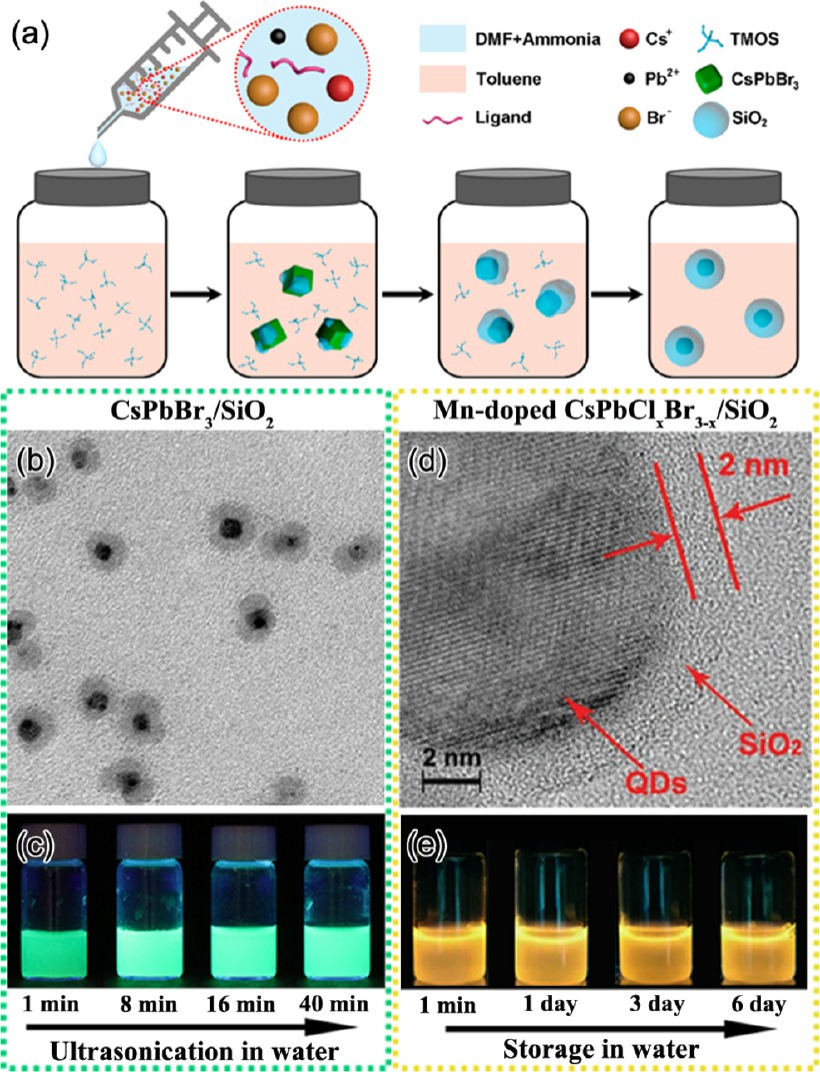
(a) Formation mechanism of CsPbBr_3_/SiO_2_ core/shell NCs. (b) TEM image and (c) photographs of CsPbBr_3_/SiO_2_ NCs showing the durability against water under ultrasonication. Panels a–c reproduced from ref (496). Copyright 2018 American Chemical Society. (d) TEM image and (e) photographs of Mn^2+^-doped CsPbCl_*x*_Br_3–*x*_/SiO_2_ core/shell nanoparticles against water. Panels d and e reproduced with permission from ref (497). Copyright 2019 John Wiley & Sons, Inc.

In comparison with pristine CsPbX_3_ NCs, the aforementioned CsPbX_3_/SiO_2_ Janus structure achieved great strides in their durability against water and irradiation. The long-term stability remains a challenge because of the partial coverage with oxides. The core/shell structure offers a more promising solution due to the complete encapsulation of CsPbX_3_ NCs. SiO_2_^496,497^ and Al_2_O_3_^291^ shells have been successfully coated on the CsPbX_3_ NCs to produce core/shell nanostructures using hydrolysis and atomic layer deposition (ALD), respectively. For example, a modified supersaturated recrystallization approach has been developed to synthesis CsPbBr_3_/SiO_2_ core/shell nanostructures, as shown in Figure 47a.^496^ During the whole reaction, the product quality was sensitive to multiple parameters, such as capping ligand type and density, reaction temperature, silica precursor, and ammonia concentration. Therefore, the well-defined core/shell structure could be realized only by carefully controlling the reaction conditions. As a result, monodisperse core/shell nanoparticles with only one CsPbBr_3_ core encapsulated in one SiO_2_ shell were obtained (Figure 47b), which displayed outstanding stability against water under ultrasonication treatment, as shown in Figure 47c. Later, a reverse microemulsion method was developed to prepare of SiO_2_ shell-coated Mn^2+^-doped CsPbCl_*x*_Br_3–*x*_ NCs by incorporation of the multibranched capping ligand TOPO.^497^ One typical feature of this product was its ultrathin SiO_2_ shell, which ensured not only improved stability but also excellent optical properties. Another method in the preparation of ultrathin inert shell was the colloidal ALD reported by Loiudice *et al.*(291) The resulting CsPbX_3_/AlO_*x*_ core/shell nanoparticles preserved the colloidal stability of CsPbX_3_ NCs and it was possible to control the thickness of the AlO_*x*_ shell from 1 to 6 nm. For inert shell encapsulation, the product always exhibited improved photophysical features and enhanced stability compared to naked CsPbX_3_ NCs. However, the inert shell would weaken the electrical properties, resulting in a poor performance in photoelectric devices such as solar cells and electroluminescent LEDs. It may provide more possibilities in practical applications if one can further reduce the inert shell thickness or employ another semiconductor material to encapsulate CsPbX_3_ NCs.

**Figure 48:**
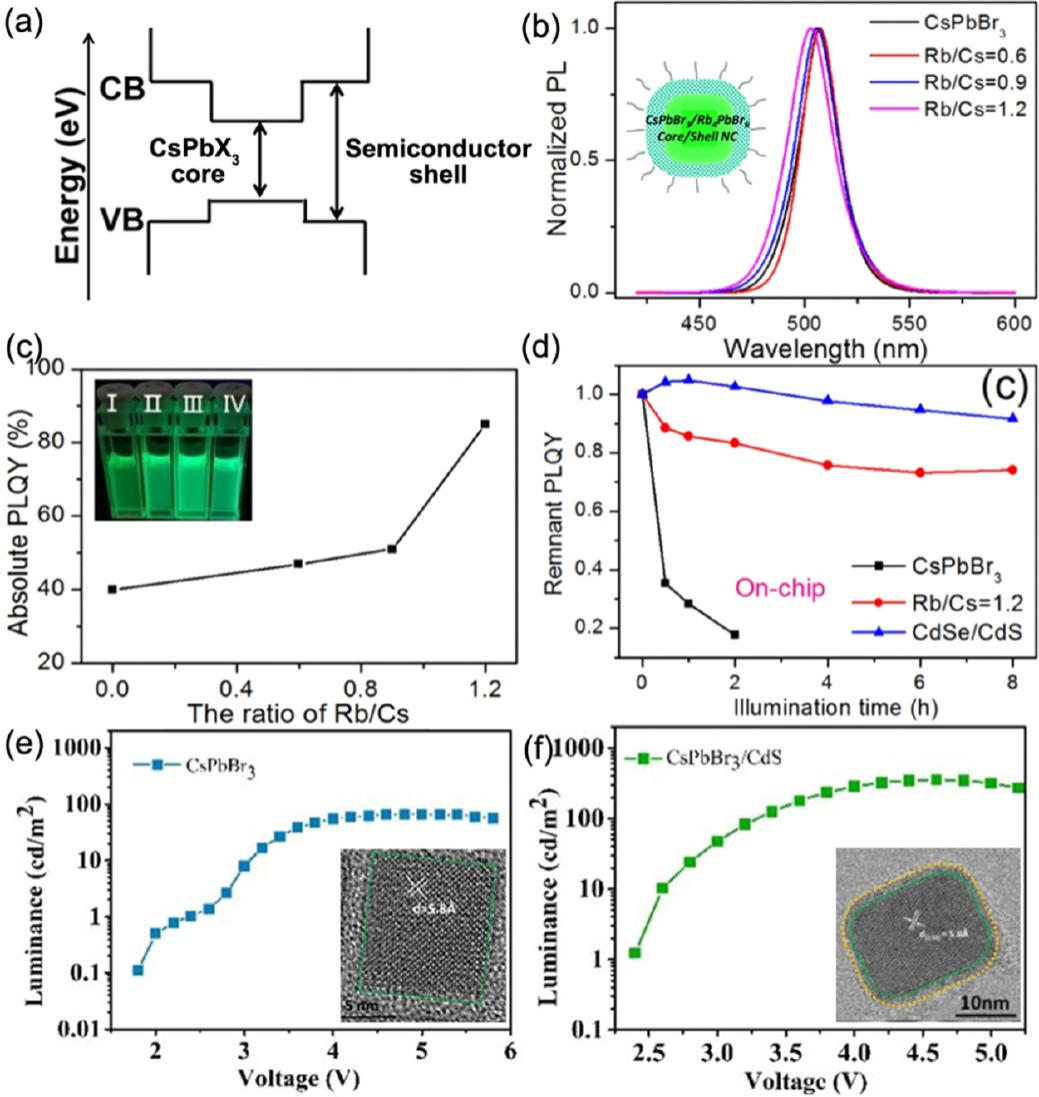
(a) Band alignment of type-I composite with core/shell structure. (b) PL spectra, (c) PLQY, and (d) photostability of naked CsPbBr_3_ and CsPbBr_3_/Rb_4_PbBr_6_ core/shell NCs. Insets in (c) show the images of CsPbBr_3_ NCs with/without Rb treatment under 365 nm lamp: I, CsPbBr_3_; II, Rb/Cs = 0.6; III, Rb/Cs = 0.9; IV, Rb/Cs = 1.2. Panels a–d are reproduced from ref (301). Copyright 2018 American Chemical Society. *L*–*V* curves of (e) CsPbBr_3_ and (f) CsPbBr_3_/CdS core/shell NCs. Insets in (e) and (f) show TEM images of CsPbBr_3_ and CsPbBr_3_/CdS NC. Panels e and f are reproduced with permission under a Creative Common CC-BY license from ref (501). Copyright 2019 Frontiers.

Inspired by the core/shell structure in traditional II–VI (*e.g*., CdSe/ZnS) quantum dots,^122,498^ a variety of semiconductors have been exploited for the synthesis of CsPbX_3_-based heterojunction. In most cases, Cs_4_PbX_6_ is usually employed as the shell composition to enhance CsPbX_3_ performance in photophysical characteristics and durability.^364,417,430,450,452^ It may be ascribed to the similar ternary crystal structure and identical [PbX_6_]^4–^ units in both CsPbX_3_ and Cs4PbX_6_, which makes the corresponding dual-phase composite stable. In the bulk phase and multi-nanoparticle coating, CsPbX_3_ embedded in Cs_4_PbX_6_ host achieves an enhancement in both PLQY and stability compared to the pristine CsPbX_3_. Only limited investigations, however, have been reported in the single-particle encapsulation. A seed-mediated approach has been established in the synthesis of CsPbBr_3_/Cs_4_PbBr_6_ core/shell NCs.^499^ In a typical process, additional cesium and halide precursors were introduced into as-prepared CsPbBr_3_ NC solution under certain conditions, resulting in hexagonal Cs_4_PbBr_6_ shell formation on the CsPbBr_3_ surface. The distinctive core/shell heterostructure consisted of a core with narrow band gap and a shell with a large band gap, resulting in a type-I band alignment, in which CB and VB of the core were located within those of the shell (Figure 48a). Consequently, the excited carriers could be well-confined within the CsPbX_3_ core and gave rise to an enhanced recombination rate and PLQY. In parallel to Cs_4_PbX_6_, also CsPb_2_X_5_, with its large band gap, has been exploited in the fabrication of type-I heterostructures, this time containing a CsPbX_3_ core and a CsPb_2_X_5_ shell, again with the purpose to optimize the optical features.^443,500^

In addition to Cs_*x*_Pb_*y*_X_*z*_ shell growth, Rb_4_PbX_6_ and II–VI semiconductors might be suitable shell materials for CsPbX_3_ NCs to fabricate type-I or quasi-type-I structures. For example, a post-synthesis phase transformation CsPbBr_3_ NC → CsPbBr_3_/Rb_4_PbBr_6_ core/shell nanostructure was presented by reacting CsPbBr_3_ NCs with rubidium oleate (also discussed in Composition Control by Ion Exchange and Suppression of Exchange section).^301^ By controlling the Rb/Cs ratio in the precursor, it was possible to regulate the thickness of the Rb_4_PbBr_6_ shell, resulting in an obvious blue shift in PL emission and increasing absolute PLQY, as shown in Figure 48b,c. More importantly, the core/shell hybrid showed significantly enhanced photostability after a long-term operation, which was comparable to the CdSe/CdS core/shell quantum dots (Figure 48d). Very recently, a II–VI semiconductor CdS shell was found to efficiently suppress the nonradiative recombination of CsPbX_3_ NCs due to the type-I alignment.^501^ In addition, the CdS layer effectively passivates the surface traps, leading to a higher radiative recombination rate. Notably, an inverted LED (ITO/ZnO:Mg/QDs/CBP(4,4′-bis(*N*-carbazolyl)-1,1′-biphenyl)/MoO_3_/Al) was fabricated based on the CsPbBr_3_/CdS core/shell heterostructure. A maximum luminance of 354 cd/m^2^ was measured for CsPbBr_3_/CdS NCs, whereas a value of only 65 cd/m^2^ was measured for pure CsPbBr_3_ nanoparticles. The average EQE was 0.4 and 0.07% for the core/shell and naked samples, respectively. Though the overall performance of core/shell heterostructure is moderate, it sheds some light on future directions in the modification of CsPbX_3_ NCs.

**Figure 49:**
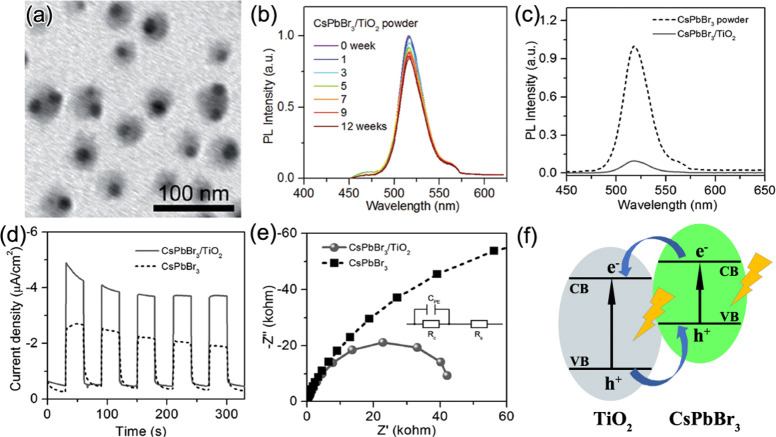
(a) TEM image of CsPbBr_3_/TiO_2_ core/shell NCs. (b) Relative PL intensity of CsPbBr_3_/TiO_2_ NCs in water. (c) PL spectra, (d) transient photocurrent responses, and (e) Nyquist plot of CsPbBr_3_ and CsPbBr_3_/TiO_2_. Panels a–e reproduced with permission from ref (365). Copyright 2017 John Wiley & Sons, Inc. (f) Band alignment within this type-II heterostructure.

By tuning the composite components, one can easily adjust the energy or charger-transfer process.^427^ For example, type-II composites could be fabricated using TiO_2_ shell to encapsulate CsPbBr_3_.^365^ Similar to inert shell coating, the resulting product exhibited a well-defined core/shell nanostructure and excellent stability in water, as shown in Figure 49a,b. However, its PL emission demonstrated an obviously quenching compared to naked CsPbBr_3_ NCs (Figure 49c). Moreover, photoelectrochemical studies including transient photocurrent responses and Nyquist plots suggested an increased charge separation efficiency of CsPbBr_3_ NCs upon TiO_2_ shell encapsulation (Figure 49d,e). In strong contrast to the aforementioned type-I composite, photoinduced charges were effectively separated and accumulated in the TiO_2_ and CsPbBr_3_ components, respectively, and this might facilitate photocatalytic reactions.

## Nanocrystals of Lead-Free Perovskite-Inspired Materials

Despite rapid advancements in the synthesis, understanding and performance of Pb-based perovskite NCs, the toxicity of Pb and the soluble nature of the Pb-based compounds in polar solvents remain an issue for their widespread application. This is related to the fact that the lead content in electronic products is restricted to 0.1 wt % by the Regulation of Hazardous Substances. This lead content restriction is as per the European Union guidelines and may vary in other jurisdictions.^502^ This practical consideration demands for environmentally benign metal-halide perovskites. Furthermore, there is the fundamental question on whether we could identify alternative classes of materials that could replicate the exceptional optoelectronic properties of the Pb-halide perovskites. Both factors have motivated researchers across the globe to undertake extensive theoretical and experimental work for designing Pb-free metal-halide perovskites. In this subsection, we will highlight the major progress of the field, with emphasis on colloidal NC systems. Readers may also refer to prior review articles on Pb-free perovskite NCs.^102,109,114,503−507^ In the present article, we will capture the recent developments and insights into Pb-free metal-halide perovskite NCs. In addition to materials with perovskite crystal structures, we will also discuss perovskite derivatives that are chemically or electronically analogous to MHPs, but do not have a perovskite structure.

### Lead-Free Perovskites and Their Derivatives

#### Colloidal Synthesis and Optical Properties

**Figure 50:**
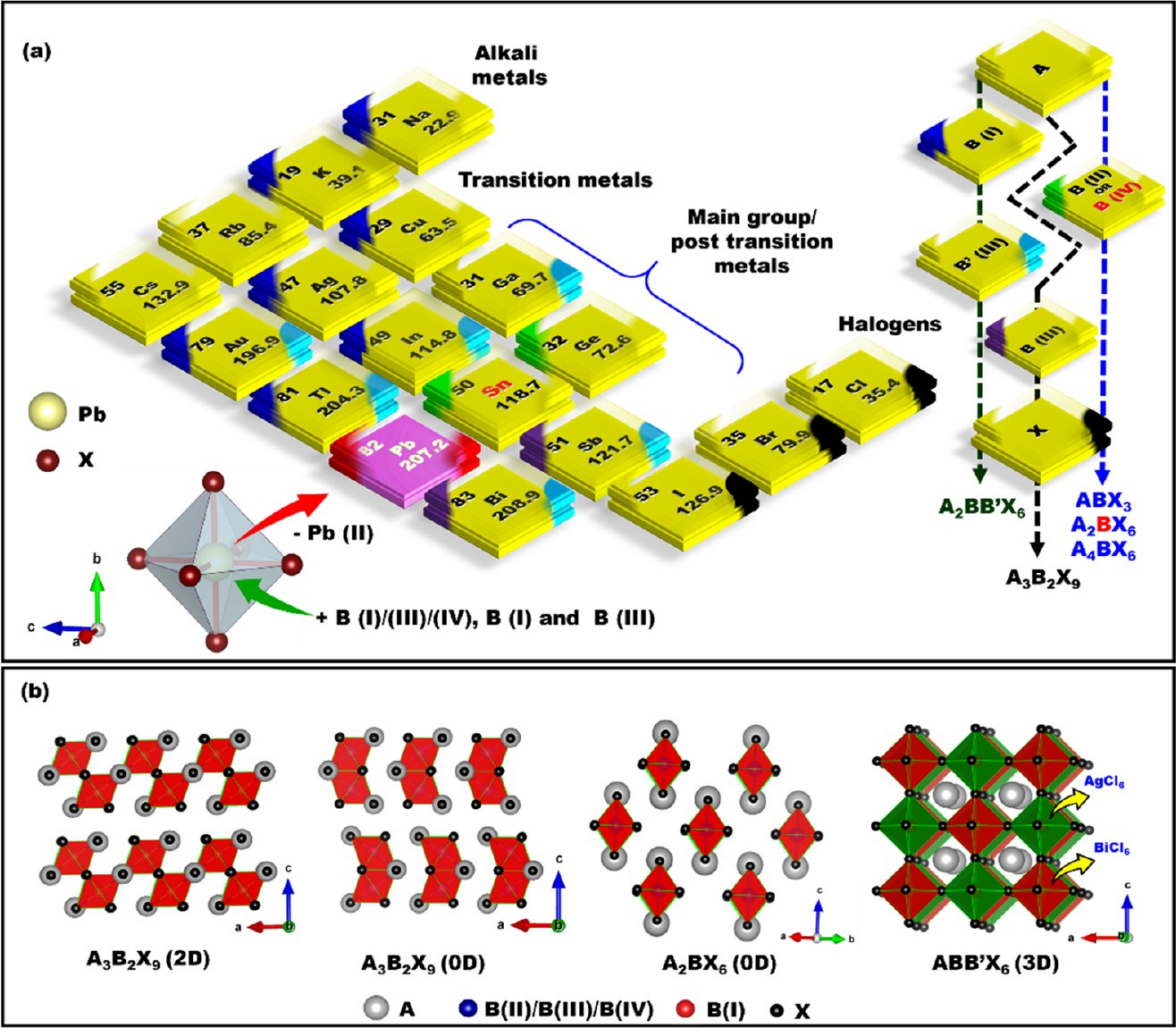
(a) Combinations of elements from a part of periodic table forming possible Pb-free metal-halide perovskite and their derivative structures. The triangular color code specifies the site occupancy as per the compositional formula presented in the extreme right. (b) Structural presentation of Pb-free metal-halide perovskites and perovskite derivatives showing network of octahedra in 3D, 2D, and 0D directions.

Figure 50a shows a selection of elements from the periodic table that are presently being considered as substitutes for Pb(II). The color code specifies the B-site occupancy in composition presented at the extreme right in Figure 50a. The crystal structures of representative generic compositions for which colloidal NCs have already been prepared are given in Figure 50b. Substituting Pb(II) with other group 14 elements (*e.g.*, Sn(II) and Ge(II)) maintains the perovskite crystal structure (*i.e.*, ABX_3_). On the other hand, substituting Pb(II) with an element from group 15 will result in materials with the A_3_B_2_X_9_ stoichiometry, and these materials could either take on a 2D or 0D crystal structure (Figure 50b). To maintain the cubic perovskite crystal structure, the B-site in ABX_3_ compounds could be alternately substituted for group 13 (*e.g.*, Ag(I)) and group 15 (*e.g.*, Bi(III)) elements. This results in double perovskite materials with the generic formula A_2_B(I)B′(III)X_6_. One can go further and replace the B′-site with a tetravalent cation (*e.g.*, Sn^4+^ or Ti^4+^). In order to maintain charge neutrality in a perovskite crystal structure, the B-site would need to be vacant. This, therefore, results in the vacancy-ordered double perovskites, with the generic formula A_2_BX_6_.

##### Sn- and Ge-Based Perovskite and Perovskite Derivative NCs

The direct substitution of Pb(II) with an isovalent element to maintain the ABX_3_ crystal structure has been one of the earliest manifestations of lead-free perovskite derivatives. Sn- and Ge-based perovskites have been successfully demonstrated in bulk thin films, both in hybrid and all-inorganic structures.^508−514^ Colloidal synthesis of CsSnX_3_ NCs with different sizes and shapes has also been reported, along with the tuning of the optical properties.^500,515−517^ The band gap of CsSnX_3_ perovskite is lower compared to the analogues Pb^2+^-based MHP mostly due to higher electronegativity of Sn ions compared to Pb.^515,518^ Huang *et al.* showed that the relatively small band gap changes from CsSnCl_3_ to CsSnI_3_ are due to interatomic Sn s and Sn p character of the VBM and the conduction band minima (CBM), respectively.^519^ This leads to high oscillator strength in these direct band gap perovskites where the photoluminescence peak was assigned to acceptor bound excitons.^519^

**Figure 51:**
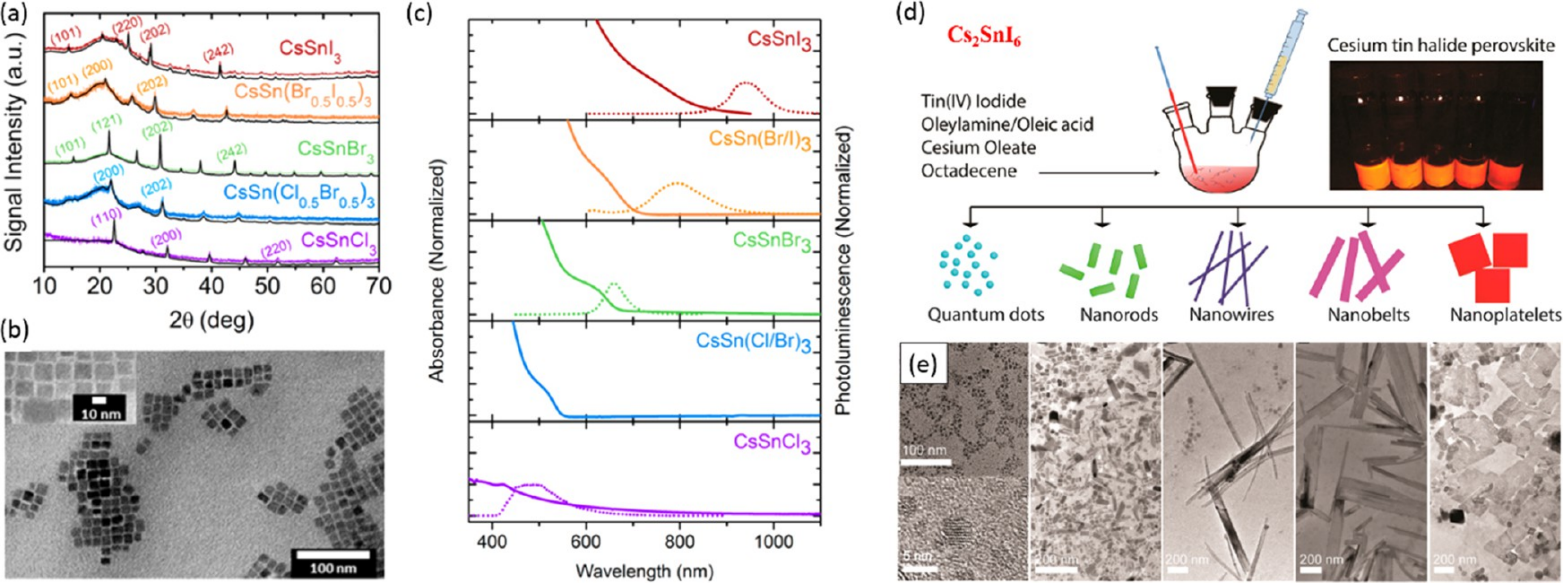
(a) XRD pattern of CsSnX_3_ (X = Cl, Cl_0.5_Br_0.5_, Br, Br_0.5_I_0.5_, I) perovskite NCs synthesized by the hot-injection method. (b) TEM images and (c) UV–vis–NIR absorbance and PL spectra of CsSnX_3_ NCs of different halide compositions. (d) Schematic illustration of the synthesis of Cs_2_SnI_6_ perovskite NCs by the hot-injection approach (left panel) and photograph of the prepared colloidal solutions of Cs_2_SnI_6_ NCs under UV light (right panel). (e) Corresponding TEM images of the Cs_2_SnI_6_ NCs of different morphologies. Panels a–c are reprinted under a Creative Commons CC-BY License from ref (515). Copyright 2016 American Chemical Society. Further permissions related to the material excerpted should be directed to the ACS. Panels d and e are adapted from ref (522). Copyright 2016 American Chemical Society.

The methods for synthesizing lead-free perovskite NCs are essentially the same as those used for the synthesis of lead-halide NCs discussed in earlier sections. The synthesis of CsSnX_3_ NCs were initially reported by Jellicoe *et al.*, who prepared the colloidal NC solution by hot injection (Figure 51a–c).^515^ The CsSnCl_3_ NCs have a perovskite crystal structure with a cubic space group (*Pm*3̅*m*), while the CsSnBr_3_ and CsSnI_3_ NCs have a lower symmetry orthorhombic (*Pnma*) phase (Figure 51a).^515^ The synthesized CsSnX_3_ nanocubes were nearly monodisperse, with a size of 10 nm, and their band gap could be easily tuned over the visible and near-infrared (NIR) range by changing the halide (X = Cl, Br, and I) composition (Figure 51a,b). The CsSnX_3_ NCs exhibit red-shifted emission (lower band gap) compared to corresponding CsPbX_3_ NCs with the same size and halide. Beyond composition, the band gap of CsSnX_3_ NCs could also be tuned through their size and dimensionality.^515,516^ For instance, Wong *et al.*(516) demonstrated the synthesis of strongly quantum-confined CsSnI_3_ NPls with blue-shifted PL (1.59 eV) compared to the PL in bulk (1.3 eV). Computations also predicted that CsSnI_3_ NPls synthesized under Sn-rich conditions would have lower defect densities and higher stability.^516^ In general, 2D-layered perovskites have been reported to exhibit higher stability over their bulk counterparts.^520^ The high density of surface ligands protects 2D-layered perovskites or NPls from air and moisture. It has been shown that Sn-based colloidal 2D perovskite NPls can be easily prepared at room temperature by ligand-assisted nonsolvent crystallization method.^209^

Despite successful shape-controlled synthesis of CsSnX_3_ NCs, the stability of these NCs is still a major concern. This is a consequence of the fact that, when these NCs are exposed to ambient conditions, Sn^2+^ quickly oxidizes to Sn^4+^, forming a different composition, Cs_2_SnX_6_,^515^ which has a 0D crystal structure, as shown in Figure 50.^521−525^ Several reports have indicated trioctyl phosphine ligands to be promising for stabilizing CsSnX_3_ NCs. However, the transformation of Sn(II) to Sn(IV) over time is inevitable.^515,516^ The morphology of perovskite NCs can have an influence on their stability. For instance, Wang *et al.*(526) demonstrated that CsSnBr_3_ cubic nanocages exhibit improved stability under ambient conditions. These nanocages were synthesized by hot injection using stannous 2-ethylhexanoate (instead of TOP-SnBr_2_) as the Sn precursor and MgBr_2_ as the bromide precursor. Importantly, the surface treatment of CsSnBr_3_ nanocages with perfluorooctanoic acid (PFOA) can significantly improve their stability against moisture, light and oxygen. This was attributed to the strongly electronegative F-groups in PFOA suppressing the oxidation of Sn^2+^ to Sn^4+^, whereas the bulky carbon chain prevented O_2_ and H_2_O access to the perovskite through steric hindrance.^526^ The stability of Sn-perovskites could also be improved through the formation of multication alloying at the A- or B-site.^527,528^ Several attempts have also been made to improve the structural and environmental stability by only partially replacing Pb with Sn.^529,530^ Such CsPb_*x*_Sn_1–*x*_X_3_ NCs can be obtained either through ion exchange or *via* direct synthesis.^528,530^ These CsPb_*x*_Sn_1–*x*_X_3_ NCs were found to be stable for months in ambient conditions and have been successfully used in the fabrication of perovskite NC-based solar cells^530^ and LEDs.^531^ However, NCs showed poor performance as compared to the polycrystalline films due to the large number of grain boundaries and surface ligands which retard charge transport.^532^ The environmental and thermal stability were also improved by mixing Cs with MA in the A-site (*i.e*. MA_0.5_Cs_0.5_Pb_1–*x*_SnxBr_3_) *via* the LARP approach.^533^

Although significant progress has been made toward the improvement of the phase stability of Sn-based perovskite NCs, it is still far from reaching the stability and optical quality of Pb-based perovskite NCs. On the other hand, groups have taken advantage of the improved stability of Sn(IV) over Sn(II) to synthesize stable and optically emissive Cs_2_SnI_6_ NCs (Figure 51d).^522,534,535^ These NCs can be prepared by conventional hot injection using oleylamine and oleic acid as ligands.^522^ The shape of the Cs_2_SnI_6_ NCs is easily controllable from spherical dots to nanorods and nanowires, and nanobelts to nanoplatelets (Figure 51e). In addition, these NCs can also be synthesized *via* hot injection without the use of capping ligands, as demonstrated by Weiss and co-workers. In this ligand-free approach, the size of the Cs_2_SnI_6_ NCs (from 12 ± 3 to 38 ± 4 nm) and thus their band gap is tunable by controlling the reaction temperature. Since these NCs are ligand-free, it is easy to process them into high-quality thick NC films by simple drop-casting, and these films could be promising for optoelectronic applications.^534^

On the other hand, unlike Sn-perovskite NCs, only a few attempts have been made to synthesize Ge-perovskite NCs.^536−538^ In general, the synthesis of Ge-perovskite NCs needs to be carried out under an inert atmosphere due to the ready oxidation of Ge(II) to Ge(IV). The instability of Ge(II) is a critical limitation with this class of materials. It has been demonstrated that monodisperse CsGeI_3_ NCs can be prepared by hot injection under an inert atmosphere.^536^ However, the NCs are highly sensitive to electron beam irradiation and they initially transform into CsI single crystals and eventually fragment into randomly oriented small debris. Moreover, CsGeX_3_ (X = Cl, Br, and I) nanorods with tunable optical properties were prepared by solvothermal synthesis, and the solar cells made of CsGeI_3_ NCs exhibit PCE of 4.92%.^538^ Despite these few studies, the shape-controlled synthesis and application of CsGeX_3_ NCs are largely unexplored. Further efforts are needed in this direction, because these NCs might be promising for solar cells due to their low band gap as compared to Sn- and Pb-based perovskites. Ge-based perovskites have also been synthesized as quantum rods (QRs).^538^ The optical band-edge of CsGeX_3_ quantum rods contains sharp absorption peak where the corresponding absorption onset shows a 90 nm red shift from 565 to 655 nm while going from CsGeCl_3_ to CsGeI_3_. The PL peak of these QRs tuned from 607 to 696 nm with a fwhm of about 25 nm.^538^

Beyond Ge-based perovskites, Eu^2+^ and Yb^2+^ have also been used in the B-site.^523,539^ CsEuCl_3_ NCs exhibit strong excitonic absorption at ∼350 nm, with a Stokes-shifted PL at 435 nm. The PL peak has a narrow fwhm of 19 nm. Interestingly, in order to overcome the Eu^2+^ → Eu^3+^ oxidation, EuCl_3_ was used and reduced to Eu^2+^ by oleylamine prior to the injection of Cs-oleate.^523^ Moon *et al.*(539) demonstrated the synthesis of monodisperse CsPbI_3_ NCs by hot injection, with a size of 10 ± 1 nm. These NCs have a low exciton binding energy of 33 meV, suggesting that excitons are readily dissociated at room temperature. The PL peak is Stokes shifted by only 7 nm to the absorption edge, and the PLQY is a high value of 58%. These materials achieved a high photoresponsivity reaching 2.4 × 10^3^ A W^–1^ in photodetectors.^539^

##### Bi-, Sb-, and Tl-Based Perovskite Derivative NCs

**Figure 52:**
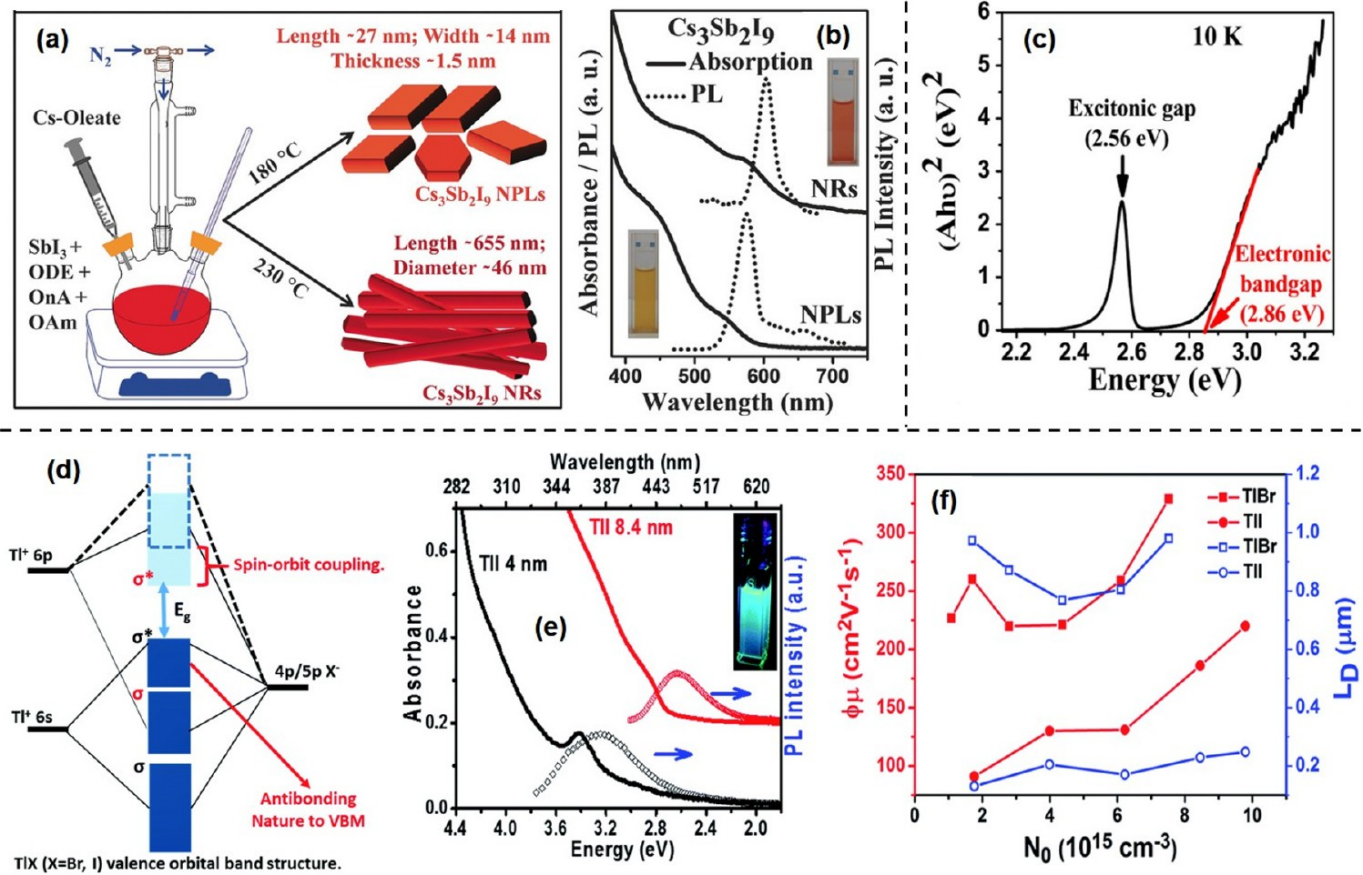
(a) Schematic showing colloidal synthesis of Cs_3_Sb_2_I_9_ nanoplatelets and nanorods. ODE, OnA, and OLA are abbreviated forms of 1-octadecene, octanoic acid, and oleylamine, respectively. (b) Optical absorption and emission spectra of colloidal Cs_3_Sb_2_I_9_ NPls and NRs. Photographs shown in the inset are of colloidal Cs_3_Sb_2_I_9_ NPls (yellow) and NRs (red) under visible light. (c) Tauc plot of Cs_3_Bi_2_I_9_ NCs obtained from optical absorption data measured at 10 K. (d) Schematic illustration of the valence orbital band structure of TlX (X = Br and I). Dark blue color corresponds to bonding and antibonding orbitals formed by hybridization of p and s atomic orbitals of Tl^+^ and X^–^. (e) Optical absorption and emission spectra of TlI NCs. Photograph in the inset shows colloidal dispersion of 8.4 nm TlI NCs under 365 nm UV light. (f) Comparison of effective carrier mobility (ϕμ, red) and carrier diffusion length (*L*_D_, blue) of films of TlBr (28.7 nm) and TlI (8.4 nm) NCs, obtained using terahertz spectroscopy. *N*_0_ is carrier density obtained at a given excitation fluence. Panels a and b are adapted with permission from ref (544). Copyright 2017 John Wiley and Sons. Panel c is adapted from ref (545). Copyright 2018 American Chemical Society. Panels d–f are adapted with permission under a Creative Commons CC BY 3.0 license from ref (554). Copyright 2017 Royal Society of Chemistry.

Next we discuss materials with trivalent B-site cations, namely, Bi(III) and Sb(III). The fact that both Bi(III) and Sb(III) have valence s^2^ electrons, similar to Pb(II), encouraged researchers to explore Bi- and Sb-halide perovskite-derivative NCs.^503,540−547^ However, bismuth and antimony are stable in the +3 oxidation state, whereas lead forms a stable +2 oxidation state. So, two B(III) (B = Sb or Bi) cations replace three Pb(II) ions in A_3_Pb_3_X_9_ (or APbX_3_), forming compounds with the general formula A_3_B_2_X_9_ (Figure 50). Consequently, the 3D perovskite structure of APbX_3_ is lost, resulting in compounds with a 2D (*e.g.*, Rb_3_Sb_2_I_9_) or 0D structure (*e.g.*, Cs_3_Bi_2_I_9_).^97,548^ The incorporation of smaller cations such as Rb as the A-site cation in place of the Cs, the layered phase is favorable over the dimer phase, and thus this favors the growth of 2D Rb_3_Sb_2_I_9_ structures. For instance, Sargent and co-workers reported the synthesis of Rb_3_Sb_2_I_9_ nanoplatelets and single crystals. Interestingly, they found that the nanoplatelets exhibit narrow emission (fwhm = 21 nm) with PL peak centred 512 nm, while the single crystals exhibit broad emission (fwhm = 75 nm) at 635 nm.^548^ There are multiple reports of the synthesis of colloidal Cs_2_Sb_3_I_3_ and Cs_2_Sb_3_Br_3_ NCs.^544,549,550^ Schematics in Figure 52a shows a typical hot-injection synthesis method for forming nanoplatelets and nanorods of Cs_3_Sb_2_I_9_ under different reaction conditions.^544^Figure 52b shows the corresponding UV–visible absorption and photoluminescence spectra. Colloidal dispersion, band-edge emission, and quantum-confinement effects are observed in Cs_3_Sb_2_I_9_ NCs. Figure 52c reports the UV–visible absorption spectra (Tauc plot) of Cs_3_Sb_2_I_9_ NCs at 10 K.^545^ Owing to its 0D structure, Cs_3_Bi_2_I_9_ shows a high exciton binding energy of ∼300 meV, clearly separating the excitonic absorption peak from the band-edge. Reduction of structural dimensionality from 3D to 2D to 0D typically decreases carrier mobility and increases band gap. Therefore, Sb- and Bi-halide perovskite derivatives show inferior photovoltaic properties compared to Pb-halide perovskites. Instead, one can think of other applications such as light-emitting diodes and surface-enhanced Raman spectroscopy using Cs_3_Sb_2_X_9_ and Cs_3_Bi_2_X_9_ NCs.^550−552^ However, further increases in PLQY by optimizing the defect chemistry is required. Interestingly, Leng *et al.*(536) reported that Cl-passivation boost the blue photoluminescence of MA_3_Bi_2_Br_9_ NCs up to a PLQY of 54.1%, which is high compared to other lead-free perovskite or perovskite-derivative NCs. Similarly, high PLQYs of 62% and 22% were reported for Cs_3_Bi_2_Cl_9_ and Cs_3_Bi_2_Br_9_, respectively, using a mixture of octylammonium bromide and oleic acid as ligands.^553^ These high PLQYs were attributed to the effective passivation of surface traps through strong ligand binding. These perovskite derivative NCs are therefore promising for further exploration.

We would like to mention here about another interesting class of materials, namely, TlX (X = Br, I). TlX does not have a perovskite crystal structure. However, (i) Tl(I) is isoelectronic with Pb(II) with 6s^2^ valence electrons, and (ii) the electronic structure of TlX is similar to that of CsPbX_3_. The electronic structure of TlX is similar to the Pb-halide perovskites, in which the valence band is composed of cation 6s halide 5 p orbitals and the conduction band is composed of cation 6p and halide 5p orbitals (Figure 52d). This motivated Mir *et al.* to synthesize colloidal TlX NCs.^554^Figure 52e compares optical absorption and emission of TlI NCs with two different sizes. TlBr and TlI NCs emit UV-blue light with ∼10% PLQY, which is reasonable compared to chloride-based perovskites emitting in the UV-blue range. Notable carrier mobilities and carrier diffusion lengths (*L*_D_) of TlBr (28.7 nm) and TlI (8.4 nm) NCs estimated by terahertz spectroscopy are shown in Figure 52f. Such high values of intrinsic carrier mobility, diffusion length, and PLQY suggest that TlX NCs can be a good optoelectronic material in the UV-blue region. On the other hand, it has to be noted that the Tl-based compounds are highly toxic.^555^

##### Cu-Based NCs

Cu belongs to the group 11, and it is mostly existing in +2 or +1 oxidation sates, and this can be a potential alternative for Pb. In general, Cu-based metal halides mostly crystallize in A_2_CuX_4_ or A_3_Cu_2_X_5_ structures.^45,556−559^ As a remark, there are no Cu-X_6_ octahedra in these structures. The interesting feature of these Cu-based NCs is that they exhibit relatively high PLQYs. For instance, Cs_2_CuX_4_ NCs can be easily prepared at room temperature by LARP, and the Cs_2_CuCl_4_ NCs that are obtained emit at 388 nm with 51.8% PLQY.^556^ In an another study, Booker *et al.*(557) reported broad-band green emission from Cs_2_CuCl_4_ NCs prepared by hot injection, and this is attributed to Cu-defect emission. The morphology of these NCs is tunable from dots to platelets and rods by varying the ratio of coordinating solvents. Moreover, the 0D Cs_3_Cu_2_I_5_ NCs synthesized by hot injection exhibit intense emission at 445 nm with an absolute PLQY of ∼87%, and this makes them promising for deep-blue LEDs.^45^ These colloidal Cs_3_Cu_2_X_5_ (X = I, Br/I, Br, Br/Cl, Cl) NCs can also be synthesized at room temperature through antisolvent precipitation and the prepared Cs_3_Cu_2_Cl_5_ NCs emit green PL with near-unity PLQY.^558^ In this case, the origin of green PL is attributed to self-trapped exciton emission. However, further studies are needed to understand the origin of green emission and high PLQY. Nevertheless, the higher thermal stability due to their inorganic nature, *eco-friendliness*, and high PLQY of these Cu-based NCs makes them promising for lighting and display applications.

##### Colloidal Double Perovskite NCs

**Figure 53:**
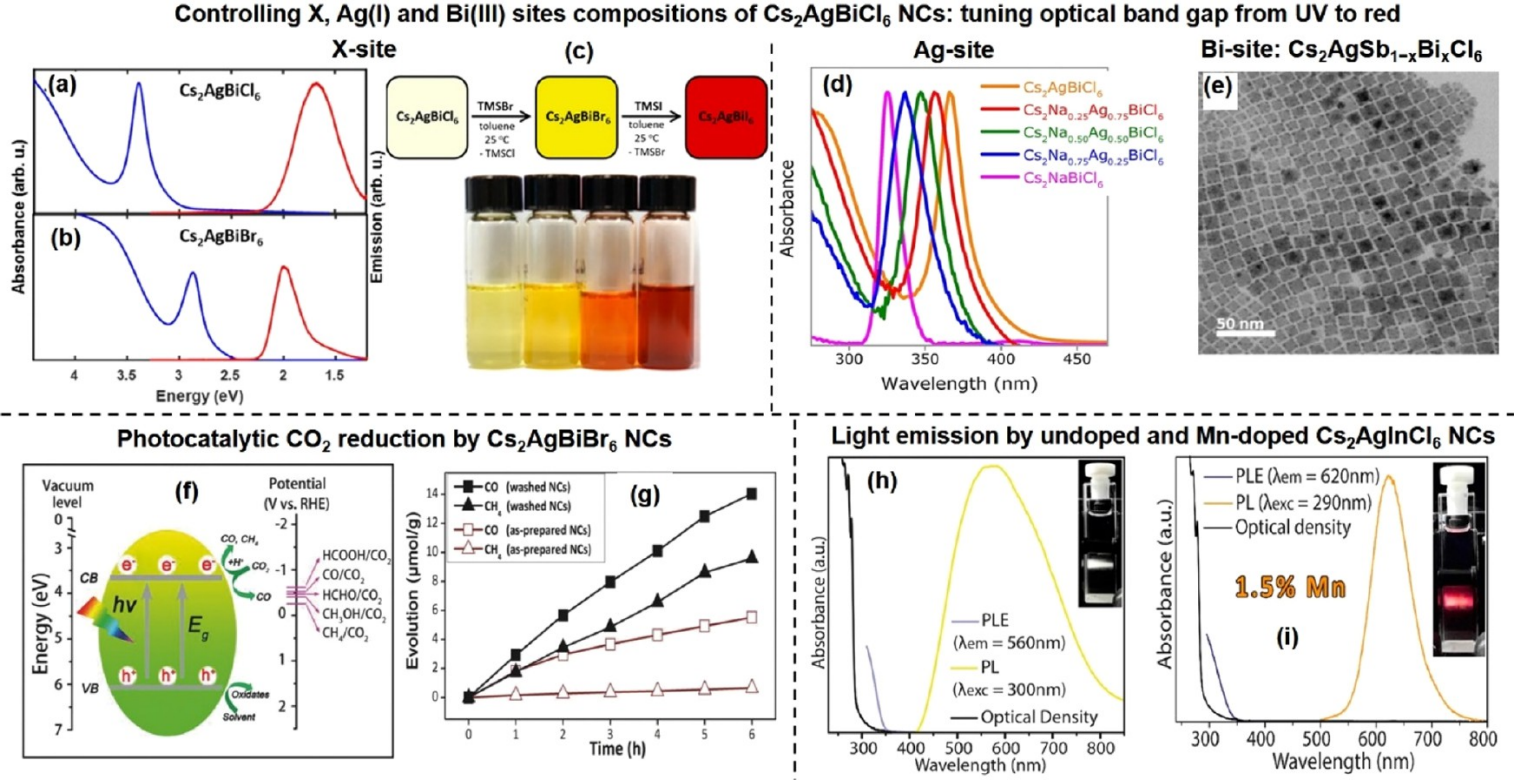
UV–vis absorption spectra (blue) measured at room temperature and PL spectra (red) measured at 20 K for (a) Cs_2_AgBiCl_6_ NCs (NCs) and (b) Cs_2_AgBiBr_6_ NCs. (c) Scheme showing halide exchange reactions using trimethylsilyl halide (TMSBr or TMSI) and photographs (left to right) show colloidal dispersions of Cs_2_AgBiBr_6_, Cs_2_AgBiBr_5.2_I_0.8_, Cs_2_AgBiBr_1.6_I_4.4_, and Cs_2_AgBiI_6_ NCs under visible white light. (d) UV–vis absorption spectra of Na alloyed Cs_2_AgBiCl_6_ NCs showing shift toward higher energy with increasing Na. (e) Transmission electron microscopy image of Cs_2_AgSb_0.30_Bi_0.70_Cl_6_ NCs. (f) Schematic showing mechanism of photocatalytic CO_2_ reduction on the surface of Cs_2_AgBiBr_6_ NCs. (g) Plot of CO and CH_4_ evolution with respect to time upon photocatalytic CO_2_ reduction using as-prepared (red) and washed (black) Cs_2_AgBiBr_6_ NCs. UV–vis absorption, PL, and PL excitation (PLE) spectra of (h) undoped and (i) Mn^2+^-doped Cs_2_AgInCl_6_ NCs. Photographs shown in the inset of panels h and i correspond to respective PL with 300 nm and Xe lamp excitation. Panels a–c are adapted from ref (183). Copyright 2018 American Chemical Society. Panel d is adapted from ref (564). Copyright 2019 American Chemical Society. Panel e is adapted with permission from ref (563). Copyright 2019 AIP Publishing. Panels f and g are adapted with permission from ref (556). Copyright 2018 John Wiley and Sons. Panels h and i are adapted from ref (566). Copyright 2018 American Chemical Society. Further permissions related to the material excerpted should be directed to the ACS.

Another promising lead-free perovskite system is the halide double perovskites (or elpasolites). These materials have the general formula A_2_B(I)B′(III)X_6_ (see Figure 50). Charge neutrality is maintained by replacing two Pb(II) ions from A_2_Pb_2_X_6_ (ABX_3_) with one B(I) and one B′(III) ions, forming compounds like Cs_2_AgBiCl_6_ and Cs_2_AgInCl_6_. Colloidal syntheses of different double perovskite NCs have been reported.^183,516,556,560−569^Figure 53a shows the UV–visible absorption and PL spectra of colloidal Cs_2_AgBiCl_6_ and Cs_2_AgBiBr_6_ NCs. The PL is significantly red-shifted from the band-edge absorptions and is believed to originate from defect and/or self-trapped excitons.^570,571^ Composition driven tuning of the band gap of double perovskite NCs has been attempted by many groups. For example, forming lower band gap materials like Cs_2_AgBiI_6_ is highly desirable for photovoltaics. Unfortunately, Cs_2_AgBiI_6_ in the bulk form could not be prepared owing to their positive heat of formation.^572^ Interestingly, NCs of Cs_2_AgBiI_6_ can be prepared.^183,573^ Therefore, NC synthesis provides an addition handle to prepare compositions and phases of double perovskites, for which the corresponding bulk counterparts do not exist. Creutz *et al.* employed an anion exchange reaction converting Cs_2_AgBiBr_6_ NCs to Cs_2_AgBiI_6_ NCs (Figure 53b).^183^ In general, the anion exchange reaction allowed them to control the X-site composition, and thereby tune the band gap and color of Cs_2_AgBiX_6_ NCs over a wide range, from 1.75 eV (Cs_2_AgBiI_6_) to 3.39 eV (Cs_2_AgBiCl_6_) (Figure 53c).^183^ However, long-term stability of red-colored Cs_2_AgBiI_6_ NCs needs to be improved. In another report, Lamba *et al*. controlled the composition of B(I)-site of Cs_2_(Na_*x*_Ag_1–*x*_)BiCl_6_ NCs to tune the optical band gap in the UV region (Figure 53d).^564^ Likewise, the composition at the B′(III)-site also can be controlled by forming Cs_2_AgSb_1–*x*_Bi_*x*_Cl_6_ NCs (Figure 53e).^563^

Typical double perovskite NCs have a cube shape (Figure 53e), which is similar to that of typical CsPbX_3_ NCs. Most likely, appropriate surface chemistry will be required to prepare double perovskite NCs of different shapes. Fine tuning of reaction conditions is often required to avoid the formation of impurity phases like CsX, AgX, and Cs_3_Bi_2_X_9_.^196^ Furthermore, NCs of double perovskites containing Ag(I), *e.g.*, Cs_2_AgSbCl_6_ and Cs_2_AgInCl_6_, have a tendency to form small Ag NCs.^560^

Double perovskite NCs of Cs_2_AgBiX_6_ (X = Cl, Br) and Cs_2_AgInCl_6_ are reasonably stable for potential applications. Unfortunately, these NCs have wide band gaps, hence they absorb only high-energy (>2.5 eV) photons, and are therefore not suitable for single junction solar cells. Zhou *et al.* used Cs_2_AgBiBr_6_ NCs for the photocatalytic CO_2_ reduction (see Figure 53f,g),^556^ demonstrating photochemical conversion of CO_2_ to solar fuels CO and CH_4_. In perspective, different double perovskite NCs should be tested for such photocatalytic applications. Another potential application of double perovskite NCs could be solid-state lighting. Luo *et al*. reported warm white-light emission with ∼86% PLQY from a bulk sample of Bi-doped Cs_2_(Ag_0.6_Na_0.40_)InCl_6_,^499^ a result that was recently confirmed by Luo *et al.*, who reported warm white-light emission from Bi-doped Cs_2_(Ag_0.6_Na_0.40_)InCl_6_ powders featuring a PLQY of 87.2%.^499^ The devices had a high stability and high-color rendering index.

Different reports on colloidal Cs_2_AgInCl_6_ and Bi-doped Cs_2_AgInCl_6_ NCs show similar broad emission with white or yellow color.^560,566,574^ For example, Figure 53h shows white-light emission with a broad emission spectrum significantly red-shifted from the absorption and PLE data.^566^ Therefore, the PL from such double perovskite NCs will not suffer from the vexing problems of self-absorption and Förster resonance energy transfer.^575^ The broad emission has been assigned to self-trapped exciton,^499,576^ but how it depends on different compositions is not yet well-understood. Yang *et al.*(577) showed that the indirect band gap can be tuned to a direct band gap in Cs_2_AgIn_*x*_Bi_1–*x*_Cl_6_ double perovskite NCs by increasing the In content. The direct band gap double perovskite NCs exhibit higher absorption cross section and the PLQY as compared to indirect band gap (Cs_2_AgBiCl_6_) NCs.

Another approach to impart visible and near-infrared light emission is to dope luminescent metal ions like Mn^2+^ and lanthanides like Yb^3+^ and Er^3+^.^578,579^Figure 53i shows the red-colored light emission from Mn^2+^-doped Cs_2_AgInCl_6_ NCs. Larger lanthanide ions require coordination number ≥6 to incorporate into the lattice. Typical semiconductors like Si, GaAs, and CdSe have coordination number = 4 for the metal ion, and are therefore not suitable for doping lanthanides. Interestingly, both ABX_3_ perovskites and AB(I)B′(III)X_6_ double perovskites with B-site coordination number = 6, can incorporate lanthanide ions.^580^ Yb^3+^- and Er^3+^-doped Cs_2_AgInCl_6_ NCs with near-infrared emission at ∼990 nm due to quantum cutting and 1540 nm (low-loss optical communication range) have been reported.^565,568^ Yb has also been reported to directly substitute Pb to form CsYbI_3_ NCs with an emission wavelength 671 nm and could be synthesized by hot injection.^539^

#### Light-Emission Applications of Lead-Free Perovskite Nanocrystals

**Figure 54:**
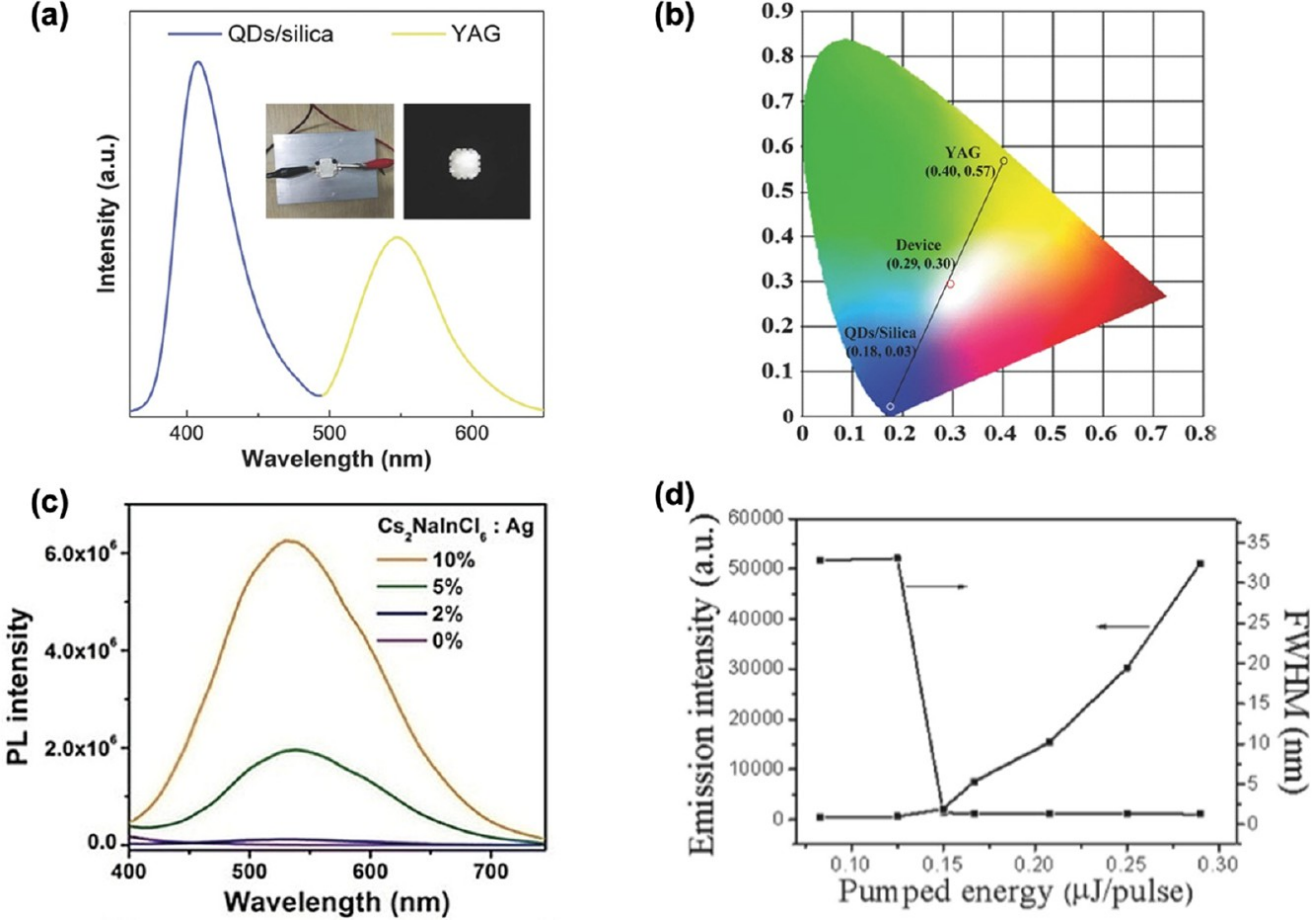
Applications of NCs of lead-free perovskite-inspired materials in light-emission applications. (a) Electrolumienscence spectra and (b) CIE coordinates of the Cs_3_Bi_2_Br_9_ blue phosphor and yellow YAG phosphor excited with a UV-emitting GaN LED, as well as the CIE coordinates of the overall white-light LED. (c) PL spectra of Cs_2_NaInCl_6_ alloyed with Ag. (d) Emission intensity and full-width at half maximum of CsSnI_3_ quantum dots doped in a cholesteric liquid crystal as a function of the pump energy. Panels a and b are reprinted with permission from ref (551). Copyright 2017 John Wiley and Sons. Panel c is reprinted with permission from ref (576). Copyright 2019 John Wiley and Sons. Panel d is reprinted from ref (582). Copyright 2018 American Chemical Society.

For light-emission, lead-free perovskite-inspired materials have mostly been used in applications involving optical excitation rather than charge injection. Namely, these applications are phosphors for white-light emitters and gain media for optically pumped lasers. For phosphors, one approach has been to use a UV GaN LED to excite blue-emitting quantum dots and a yellow-emitting phosphor to achieve white-light LEDs. Leng *et al*.^551^ used Cs_3_Bi_2_Br_9_ colloidal quantum dots as the blue-emitter (410 nm PL wavelength), and Y_3_Al_5_O_2_ (YAG) as the yellow-emitter (broad PL centered at 551 nm wavelength). The white-light LED had CIE coordinates of (0.29,0.30) and a color temperature of 8477 K (Figure 54a,b).^551^ Cs_3_Bi_2_Br_9_ was advantageous because it forms a passivating BiOBr layer in the presence of moisture. This increases the PLQY, but also improves the stability of the quantum dots in the presence of moisture and acid. As a result, the Cs_3_Bi_2_Br_9_ quantum dots could be mixed with TEOS, which was hydrolyzed with water and HBr to form silica. The resulting composite of quantum dots embedded in silica had improved stability, with the 72% of the PL being retained after 16 of exposure to a UV lamp, and 75% of the PL being retained after 16 h heat stressing at 60 °C.^551^ Tan *et al.*(581) also demonstrated white-light LEDs using Cs_2_SnCl_6_ perovskites as the blue-emitter and Ba_2_Sr_2_SiO_4_:Eu^2+^ and GaAlSiN_3_:Eu^2+^ as the yellow phosphors. Under excitation from a UV GaN LED, the white-light LED had CIE coordinates of (0.36,0.37) and color temperature of 4486 K. The Cs_2_SnCl_6_:Bi exhibited blue emission at 455 nm, with a PLQY of 78.9%, which was higher than Cs_3_Bi_2_Br_9_ (10-19% PLQY).^551,581^ The vacancy-ordered perovskite was also stable against moisture, due to the formation of a protective BiOCl layer and due to the tin cation already being in the more stable +4 oxidation state.^581^ Yang *et al.* obtained lead-free blue-emitters with similarly high PLQYs of 32.8% using Eu^2+^-doped CsBr NCs. By combining these NCs with YAG:Ce^3+^ with a UV-emitting GaN LED, white emission with CIE coordinates of (0.32, 0.34) and color temperature of ∼6300 K was obtained.^570^

Recently, it was demonstrated that white-light emission can be achieved using a phosphor comprising solely a double perovskite. Luo *et al.* demonstrated that powders of Cs_2_(Ag_0.6_Na_0.4_)InCl_6_ doped with 0.04% Bi luminesces broadly across 400–800 nm wavelength (centered at 570 nm) with 86 ± 5% PLQY, and 1000 h stability.^499^ The broad emission arises due to the formation of a self-trapped exciton as a result of strong electron–phonon coupling and Jahn-Teller distortion in the AgCl_6_ octahedron. By pressing Cs_2_(Ag_0.6_Na_0.4_)InCl_6_ powder onto a GaN LED and encapsulating with silica, white-light emission was obtained through the blue emission from the LED mixing with the broad emission from the double perovskite phosphor. The white-light LED had CIE coordinates of (0.396, 0.448) and a color temperature of 4054 K, and stability over 1000 h in air.^499^ Han and co-workers also produced a series of works showing broad-band emission from Cs_2_AgIn_*x*_Bi_1–*x*_Cl_6_, Ag-doped Cs_2_NaInCl_6_ and Mn^2+^-doped Cs_2_NaIn_0.75_Bi_0.25_Cl_6_ NCs.^561,576,577^ Cs_2_AgBiCl_6_ has an indirect band gap, with low PLQY. Alloying In into this system resulted in a direct but parity-forbidden band gap. With increasing In content, the PLQY was found to increase up to 36.6% (with 90% In), along with an increase in broad emission centered at 570 nm wavelength. This was attributed to the emission from the parity-forbidden band gap, which prevents absorption but allows radiative recombination.^583^ Cs_2_NaInCl_6_ has a wide band gap of 4.55 eV but almost no PL. Alloying with Ag resulted in an increased PLQY from a broad-band sub-band-gap emission, reaching up to 31.1% with 10% Ag. These results were attributed to a dark self-trapped exciton being present in Cs_2_NaInCl_6_ that became bright with Ag alloying by breaking the parity-forbidden transition in Cs_2_NaInCl_6_ (Figure 54c).^576^ The self-trapped exciton in Cs_2_NaIn_*x*_Bi_1–*x*_Cl_6_ is also believed to be dark, with only blue PL due to free excitons. Broad-band yellow emission was obtained by doping with Mn^2+^, which resulted in a PLQY of 44.6% being obtained. This broad-band transition was attributed to the dark self-trapped exciton transferring to the ^4^T_1_ excited state of Mn^2+^ and relaxing to give PL.^546^ Recently, Lee *et al*. have reported characteristic absorption features in the Na/Bi^3+^ system. Cs_2_NaBiCl_6_ NCs and Cs_2_NaBiBr_6_ NCs showed sharp and discrete single peaks assigned to the s–p transition (6s^2^ → 6s^1^p^1^ ) from the [BiX_6_]^3–^ units within the crystal lattice of elpasolite structures. Such discrete optical transition characteristics have not been observed for Ag/M^3+^ DP or for Cs_3_Bi_2_X_9_ materials.^569,584^ A series of studies on Bi-doped Cs_2_Na_1–*x*_Ag_*x*_InCl_6_ and Cs_2_Na_1–*x*_Ag_*x*_BiCl_6_ NCs were recently reported in which the extent of Ag/Na alloying was found to regulate the PLQY of the NCs.^506,567^ Light emission in these materials was identified to arise from recombination from carriers trapped in localized states. A combined experimental and computation study showed that the extent of localization of the holes (which were found to be localized at AgCl_6_ octahedra), was strongly dependent on the amount of Na^+^ ions, that is, on the average number of NaCl_6_ octahedra surrounding each individual AgCl_6_ octahedron. In essence, the higher this number, the more likely is for the holes to stay localized, and the higher is the PLQY. Also, the same authors found that, regardless of the type of ligands used in the synthesis and of any post-synthesis ligand exchange that was attempted, the PLQY for thee materials could not be increased beyond 37%, against the 86% reported for the bulk.^508^ Their conclusion, based also on a series of experiments and calculations, was that unpassivated surface traps are most likely responsible for the lower PLQY, and therefore, these materials are much less surface tolerant than the corresponding Pb-based halide perovskites.

Beyond the use of lead-free perovskite-inspired materials for phosphors, tin- and germanium-based perovskites have been demonstrated as potential gain materials for optically pumped lasing. Xing *et al.* demonstrated amplified spontaneous emission (ASE) across the visible to near-infrared (700–950 nm wavelength) from CsSnBr_*x*_I_3–*x*_ thin films.^525^ By reducing the trap density in the thin films through the addition of SnF_2_ during synthesis, the lasing threshold in CsSnI_3_ was reduced to a low value of 6 μJ cm^–2^ (whereas lasing was not obtained in the films without SnF_2_) and a quality factor exceeding 500. Lee *et al.*(582) synthesized CsSnI_3_ quantum dots 3–5 nm in size, which were doped into a cholesteric liquid crystal (CLC). The CsSnI_3_ quantum dots acted as the gain medium, and the CLC as the optical resonator. The lasing threshold was ∼0.8 mJ cm^–2^ pulse^–1^, but the quality factor was ∼2000. The device was also air-stable, with the lasing emission intensity decreasing only by 13% after 6 months of storage in air compared to the initial intensity.^582^ Hints of amplified spontaneous emission was also found in CH_3_NH_3_Sn_0.5_Ge_0.5_I_3_ by Nagane *et al.*,^585^ in which the PL fwhm decreased from 75 to 40 nm when the excitation density was increased from 10^15^ to 10^16^ cm^–3^. This 50% mixture of Sn and Ge was also found to give the lowest Urbach energy (of 47 meV) across the Sn–Ge composition series.^585^ Recently, Moon *et al.*(539) reported the synthesis of high-quality cesium ytterbium triiodide (CsYbI_3_) cubic perovskite NCs with a PLQY of 58%. It was found that the CsYbI_3_ NCs exhibit a high photoresponsivity (2.4 × 10^3^ A W^–1^) with an EQE of 5.8 × 10^5^%.

### Designing Additional Double Perovskite Compositions

The exploration of Pb-free materials is driven by theoretical predictions.^586−588^ Based on the above discussion, it appears that, (i) the optoelectronic properties of Pb-free perovskites are still inferior compared to Pb-halide perovskites, and (ii) only a few double perovskite compositions have been explored so far, while there are hundreds of possible compositions for metal-halide double perovskite that have yet to be explored.^589^ The most important criteria when screening materials based on computations are (i) thermodynamic stability, (ii) band gap (which should be <2.5 eV), and (iii) effective masses of charge carriers (which should be <1 electron mass). Additional screening criteria include trap energy, along with the low toxicity and earth abundance of the elements.

**Figure 55:**
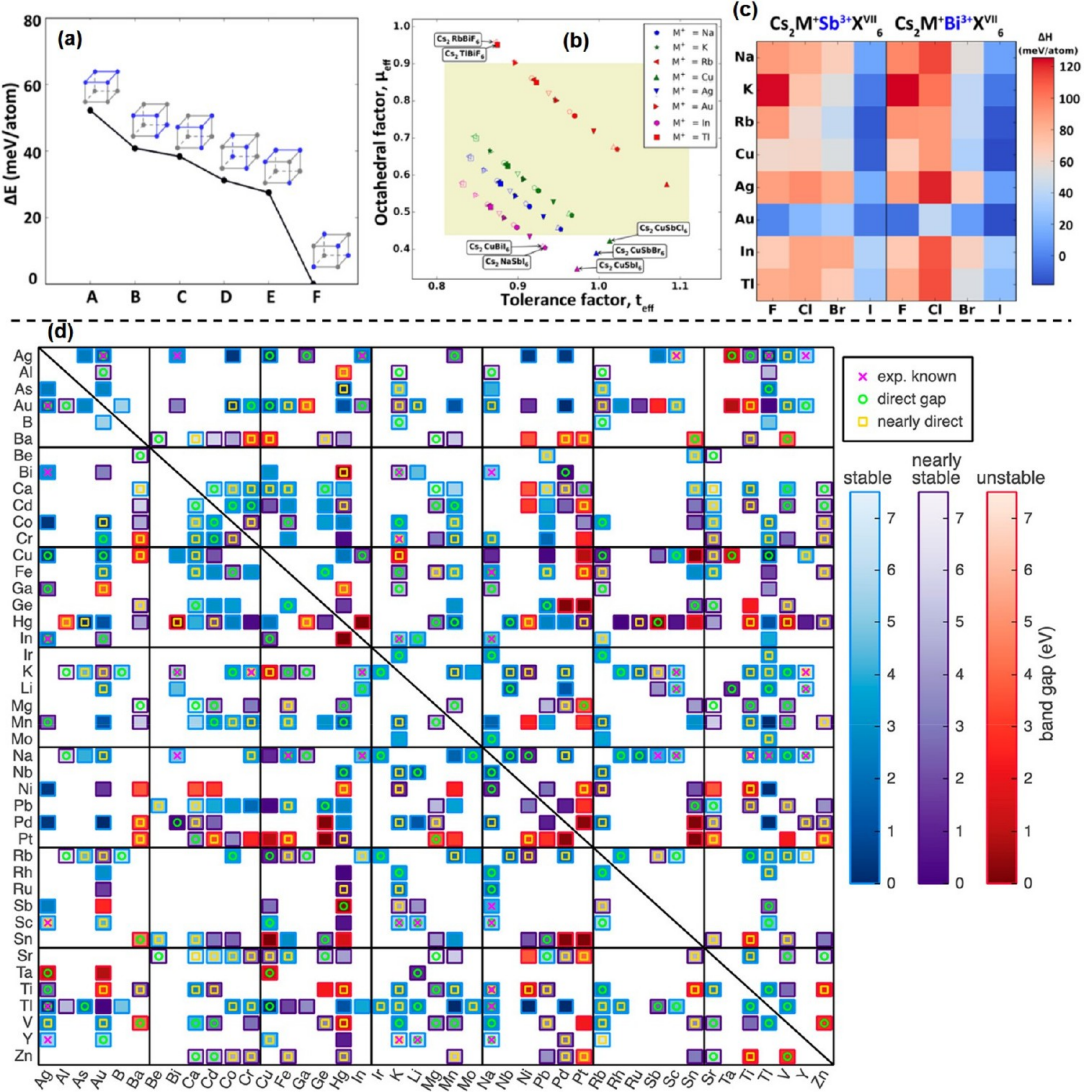
(a) Total energies for different arrangements [AgX_6_]^5–^ and [BiX_6_]^3–^ octahedral motifs calculated in a 2 × 2 × 2 supercell of Cs_2_AgBiCl_6_. The energy for most stable configuration (F) is set at zero. (b) Evaluation of structural stability of different Cs_2_B(I)BiX_6_ and Cs_2_B(I)SbX_6_ with varying compositions for B(I) (indicated by M^+^ in the figure) and X, on the basis effective tolerance factor (*t*_eff_) and octahedral factor (μ_eff_) variables. The compositions present outside the inner square are unstable. Red, green, blue, and maroon colors correspond to F, Cl, Br, and I, respectively. The open and filled symbols specify Sb- and Bi-containing perovskites, respectively. (c) Thermodynamic stability of the double perovskite compositions calculated using decomposition enthalpy (Δ*H*). Higher positive values of Δ*H* indicate more stable compositions. (d) Map showing calculated thermodynamic stability, band gap, and experimental existence for a large number Cs_2_BB′Cl_6_ double perovskite compositions with different combinations of B and B′, shown along the axes. The map is mirrored across the diagonal line because B and B′ are treated equivalently. Details of calculation and classification such as stable, nearly stable, and unstable are given in ref (589). Panels a–c are adapted from ref (590). Copyright 2017 American Chemical Society. Panel d is adapted from ref (589). Copyright 2020 American Chemical Society.

The stability of double perovskites strongly depends on the structural parameters. A_2_B(I)B′(III)X_6_ has two kinds of octahedral motifs like [B(I)X_6_]^5–^ and [B(III)X_6_]^3–^. For example, in Cs_2_AgBiCl_6_, the motifs are [AgX_6_]^5–^ and [BiX_6_]^3–^ and are represented by blue and gray dots, respectively, in Figure 55a. In a crystal, the octahedral motifs can be arranged in six different ways (A–F), as shown in Figure 55a. For Cs_2_AgBiCl_6_, it is found that the F arrangement, *i.e*., with [AgX_6_]^5–^ and [BiX_6_]^3–^ motifs being arranged alternatively, gives the thermodynamic stable state. This thermodynamic stability of F arrangement is at the basis of double perovskite structure shown in Figure 50.

For ABX_3_ perovskites, the structural stability can be described by the Goldschmidt tolerance factor [)] and octahedral factor (*m* = *r*_B_/*r*_X_). These are defined using the idealized solid-sphere model, where *r*_A_, *r*_B_, and *r*_X_ are the ionic radii of A, B, and X, respectively. It has been found empirically that for the formation of ABX_3_ halide perovskites requires 0.81 < *t* < 1.11 and 0.44 < μ < 0.90.^591^ For A_2_B(I)B′(III)X_6_ double perovskite, there are two B-site cations, and therefore,  and *m*_eff_ = (*r*_B_ + *r*_B′_)/2*r*_X_. The shaded region in Figure 55b empirically suggests the requirement of *t*_eff_ and *m*_eff_ to form stable double perovskite of Cs_2_B(I)BiX_6_ and Cs_2_B(I)SbX_6_ with varying compositions for B(I) (indicated by M^+^ in the figure) and X. Such crystallographic parameters provide the initial assessment regarding the formability of a double perovskite composition. Furthermore, one can calculate the decomposition enthalpy (Δ*H*) for double perovskites using DFT (Figure 55c). In the present calculation,^590^ positive values of Δ*H* indicate thermodynamic stability. Particularly, samples with Δ*H* > 20 meV/atom are expected to be stable. Figure 55c shows that the iodide compositions show poor thermodynamic stability, which also corroborates the fact that many iodide compositions have μ values that are too small and inhibit the formation of octahedral motifs. This instability of iodide double perovskites has also been observed experimentally is most likely the main reason for the absence of experimentally observed narrow (∼2 eV) band gap double perovskites. Fluoride-based double perovskites are also stable, but often not the preferred material for optoelectronics, since the high electronegativity of fluoride is expected to yield wide band gap insulating materials.

High stability and the possibility of reasonably narrow band gap led Bartel *et al.* to screen 311 compositions of Cs_2_B(I)B′(III)Cl_6_.^589^ The mapping of these compounds, showing their thermodynamic stability, nature of the band gap, and whether they exist experimentally is displayed in Figure 55d. They could identify about 47 nontoxic double perovskite compositions with direct or nearly direct (within 100 meV) computed band gaps between 1 and 3 eV. However, many of these compositions need experimental verification.

### Summary and Future Outlook for Pb-Free MHP NCs

Various colloidal Pb-free metal-halide perovskite NCs like Cs_3_B_2_X_9_ (B = Sb and Bi), CsBX_3_ (B = Sn and Ge), and Cs_4_SnX_6_ have prepared in recent years. CsSnX_3_ and CsGeX_3_ NCs are unstable. By contrast Cs_3_Sb_2_X_9_, Cs_3_Bi_2_X_9_ and Cs_4_SnX_6_ NCs have improved stability, but charge transport is restricted due to reduced structural dimensionality (2D or 0D) in these materials. Nevertheless, these materials may find applications as stable blue phosphors, which can be used in combination with yellow phosphors for white-light emission. Interestingly, non-perovskite TlX possesses similar electronic structure to CsPbX_3_ and have demonstrated promising optoelectronic properties in the UV-blue region. However, Tl compounds are highly toxic.

Despite reasonable progress in the synthesis of double perovskite NCs, a better understanding of the origin of PL is required to tune the intensity, peak energy and shape of the broad PL, by fine tuning the composition. Compositional fine tuning is also expected to suppress the effect of reduction of Ag(I) to Ag(0) on the PL. Furthermore, doping with lanthanides (Yb^3+^, Er^3+^, *etc.*) can provide intense near-infrared emission, required for optical communication, infrared LEDs and remote sensing. Exploring light emission properties of metal-halide double perovskites and their derivatives for real life application is an important future direction. However, an important limitation of the double perovskites is their wide and/or indirect band gap. Therefore, different classes of double perovskite compositions need to be synthesized both in the bulk and nanocrystalline form. Recent work also suggests that the band gap could be reduced in alloys between compounds that form a type II alignment.^237^ We hope that, in near feature, researchers will develop stable metal-halide double perovskite compositions with <2 eV band gap, along with good charge transport properties. In the search for different Pb-free perovskite semiconductors, compositions of chalcogenide perovskites,^592^ mixed-halide chalcogenide perovskites^593^ and oxide perovskites^594^ provide additional options.^595^

Finally, very little is reported on the use of perovskite derivative NCs in electrically driven applications, such as LEDs, although recent work on thin films motivates this effort. Of the handful of examples of lead-free NC LEDs, recent reports of Cs_3_Cu_2_I_5_ are some of the more promising. 1.12% EQE was achieved, with deep blue emission.^596^ The devices exhibited reasonable stability with a half-life of more than 100 h.^596^ In addition, Ma *et al.*(547) demonstrated LEDs from Cs_3_Sb_2_Br_9_ quantum dots, with electroluminescence at 408 nm (violet color) and an EQE of 0.2%. Cs_3_Sb_2_Br_9_ is a particularly suitable material for demonstration in LEDs given that they have a PLQY of 51.2%, which is larger than other A_3_B_2_X_9_ quantum dots. Cs_3_Sb_2_Br_9_ is also stable against heat, UV illumination, air and the presence of moisture, and the LEDs retained 90% of the initial electroluminescence intensity after 6 h of operation at 7 V (∼70 mA cm^–2^ current density).^547^ This is an improvement over many Pb-based perovskite quantum dots. In thin films, Rand *et al.* found that near-infrared LEDs with Pb–Sn perovskites had a 2 orders of magnitude improvement in radiance when the films were grown with the addition of a bulky organoammonium halide ligand to passivate the surface and reduce the grain size to better confine carriers.^597^ NCs with carefully chosen ligands could therefore be worth investigation. Furthermore, Zhang *et al.*(598) recently demonstrated electroluminescence from self-trapped excitons in thin films of a Ruddlesden-Popper (C_18_H_35_NH_3_)_2_SnBr_4_ perovskite. This perovskite was synthesized by hot injection to form microplates, and self-trapping occurred in the [SnBr_6_]^4–^, which are electronically isolated from neighboring Sn–Br layers by the long oleylamine cations. Electroluminescence from the self-trapped exciton (centered at 625 nm wavelength) was obtained, with 350 cd m^–2^ and 0.1% EQE achieved. The turn-on voltage was low, at 2.2 V, and it was believed that electrons and holes were directly injected into the self-trapped states.^597,598^ This motivates future efforts to (i) understand the nature and behavior of the self-trapped exciton in the quantum-confined regime (*i.e*., in very small size NCs); (ii) explore how to narrow the PL line width (*i.e*., by varying the composition); (iii) improve charge injection into the NCs; and (iv) to further investigate other optical properties, such as anti-Stokes shifted PL.^599,600^ Finally, the ambitious goal would be to achieve white-light electroluminescence from self-trapped excitons in double perovskites.

## Doping (A and B-Sites) of MHP NCs

### B-Site Doping

Doping in metal-halide perovskite NCs has been extensively studied to improve their optical and electronic properties and structural stability by modifying the electronic structure or introducing additional channels of energy and charge transfer. Both A- and B-site doping with various mono-, di- and trivalent metal ions have been explored for this purpose. In general, A- and B-site doping can be achieved either by cation exchange or through *in situ* synthesis.^108,573,601,602^ Doping through cation exchange is briefly introduced in the section named Composition Control by Ion Exchange and Suppression of Exchange. In this section, we provide an extensive discussion on the recent progress made on A- and B-site-doped MHP NCs for improved stability and enhanced PLQY and the characterization of the additional properties resulting from doping in metal-halide perovskite NCs. In particular, special attention is paid to the Mn^2+^- and lanthanide-doped perovskite NCs, which have been widely studied over the years due to their interesting properties and potential applications.

### Mn^2+^ Doping in Perovskite Nanocrystals

Mn^2+^-doped colloidal semiconductor NCs have been a topic of intensive research for many decades, because the doping can introduce various additional optical, electronic, and magnetic properties through the interaction of the exciton with dopants.^602,603^ In Mn^2+^-doped semiconductors, the exciton energy transfer from a semiconductor host to Mn^2+^ dopants leads to orange emission by a spin-forbidden ^4^T_1_–^6^A_1_ Mn d–d transition. With the emergence of halide perovskites as a novel class of semiconductors, the Mn^2+^ doping concept has been extended to this class of materials and significant progress has been made over the last few years. Currently, doping of Mn^2+^ in halide perovskite NCs has been demonstrated mostly in cesium lead-halide NCs (CsPbX_3_, X = Cl, Br, I).

#### Mn^2+^ Doping in CsPbCl_3_ NCs

**Figure 56:**
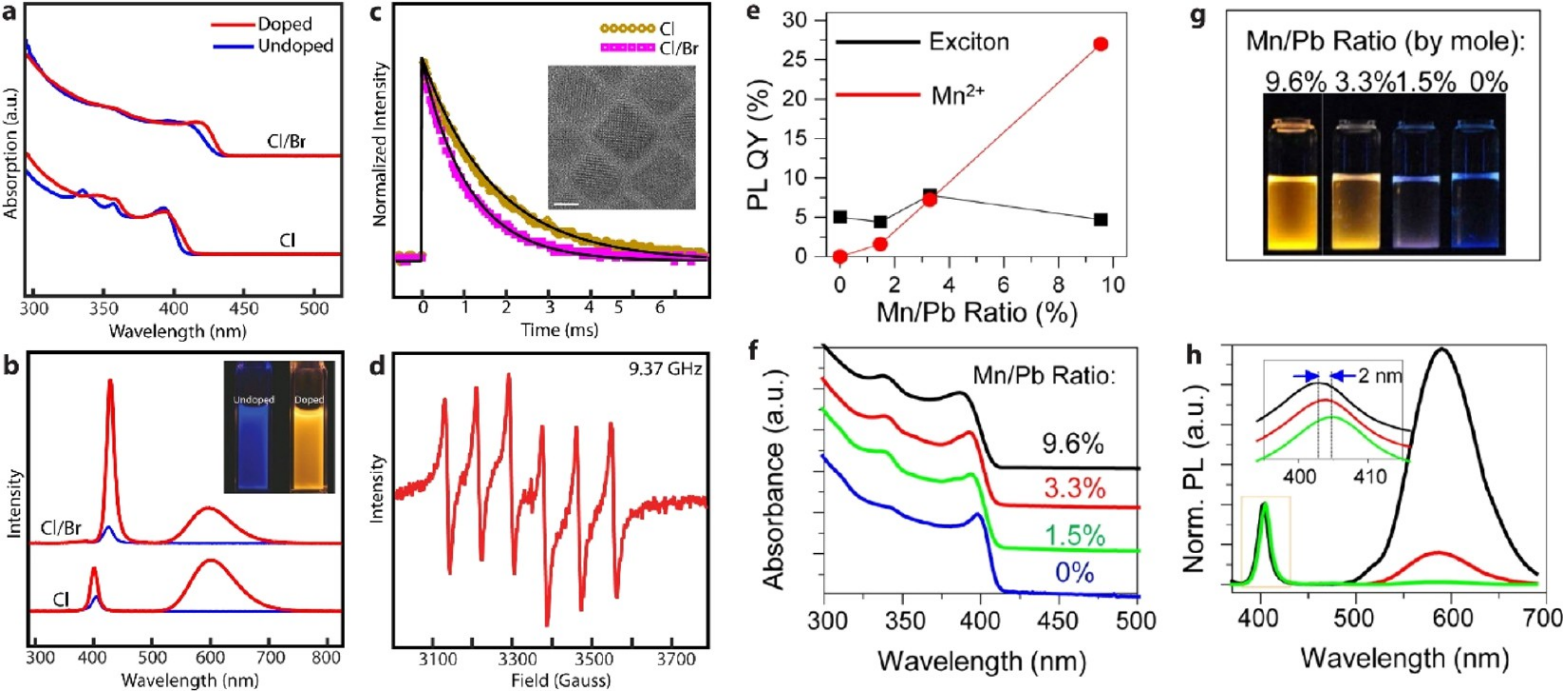
Synthesis and properties of Mn^2+^-doped CsPbCl_3_ NCs. (a) Absorption and (b) PL spectra of Mn^2+^-doped CsPbCl_3_ and CsPbCl_*x*_Br_3–*x*_ NCs with those of undoped control samples. (c) Time-dependent PL decay of Mn phosphorescence. (d) EPR signal from Mn^2+^-doped CsPbCl_3_ NCs. Images in a–d were taken from ref (604). Copyright 2016 American Chemical Society. (e) Dependence of exciton and Mn^2+^ PLQY on dopant concentration. (f,h) Normalized absorption and (f) PL spectra (h) of Mn^2+^-doped CsPbCl_3_ NCs of varying dopant concentration. Increased Mn^2+^ content is associated with a gradual blue shift of both the band-edge peak in absorption and the intrinsic NC PL peak (expanded in the inset of panel d); this can be attributed to the effects of alloying on the NC band structure. The intensity of Mn^2+^ emission increases with increasing the Mn^2+^ content with out effecting the peak position. (g) Photograph of hexane solutions of Mn^2+^-doped CsPbCl_3_ NCs of varying Mn^2+^ content illuminated by a UV lamp (365 nm). Solutions were diluted to exhibit the same optical density at 365 nm. Images in e–h were taken from ref (605). Copyright 2016 American Chemical Society.

The initial successful Mn^2+^ doping of metal-halide perovskite NCs was performed in CsPbCl_3_ NCs with nanocube morphology, which was reported by two different groups in 2016 (Son group and Klimov group).^604,605^ Mn^2+^ doping in CsPbCl_3_ NCs was achieved by adding MnCl_2_, an additional reactant as the source of Mn^2+^, under the typical hot-injection synthesis conditions of CsPbCl_3_ NCs. This resulted in doping of Mn^2+^ at the level of <1 to 10%, which showed distinct Mn^2+^ luminescence centered around 600 nm resulting from the sensitization of the Mn^2+^ ligand field transition. In this synthesis, MnCl_2_ was the most effective precursor of Mn^2+^ ions, though many other precursors such as Mn(ac)_2_, Mn(acac)_2_, and Mn(oleate)_2_ were also used as dopant precursors. However, in contrast to MnCl_2_ and CsPbCl_3_ pair for Mn^2+^ doping, extending the same approach to doping of Mn^2+^ in CsPbBr_3_ NCs using MnBr_2_ was not successful. On the other hand, when MnCl_2_ was used as precursor for Mn^2+^ doping in CsPbBr_3_,^606^ Mn^2+^-doped Cl/Br mixed-halide NCs were obtained. This suggests that the formation of the Mn–Cl bond is preferred over that of the Mn–Br bond when attempting doping using Mn^2+^ halide as the precursor. Figure 56a,b shows the absorption and photoluminescence spectra of the undoped and Mn-doped CsPbCl_3_ and CsPb(Cl/Br)_3_ NCs synthesized using MnCl_2_ as the Mn^2+^ precursor at a doping concentration of <1%. The characteristic Mn^2+^ photoluminescence appearing at ∼600 nm indicates the doping of Mn^2+^ ions into perovskite NC hosts. The resultant Mn^2+^ photoluminescence is caused by the energy transfer from the host to d-d transition of Mn^2+^ ions. At low doping concentrations, the Mn^2+^ luminescence exhibits nearly single exponential decay, as expected from the relatively homogenous ligand field environment and weak interdopant coupling. EPR data of Mn-doped CsPbCl_3_ with <1% doping also showed the characteristic fine structure of Mn^2+^ expected from cubic lattice symmetry, confirming the successful doping of Mn^2+^ in the NC host.

**Figure 57:**
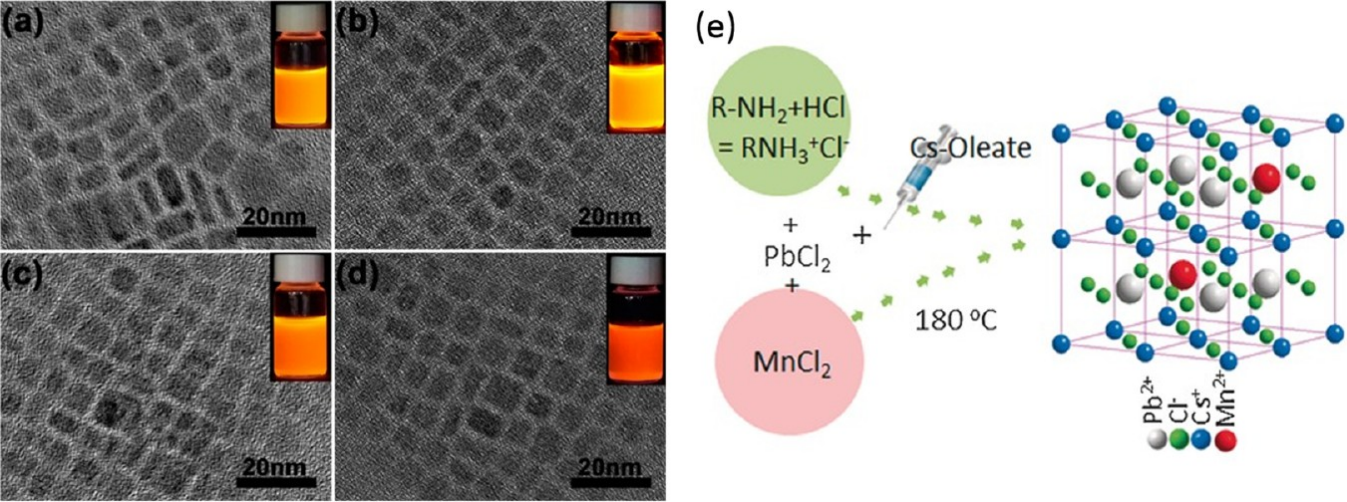
Heavy Mn^2+^ doping in CsPbCl_3_ NCs. (a–d) TEM images of the CsPb_*x*_Mn_1–*x*_Cl_3_ NCs that are prepared with Pb to Mn molar feed ratios of 1:1.25 (a), 1:2.5 (b), 1:5 (c), and 1:10 (d) at 170 °C; *x* = 0.02, 0.04, 0.10, and 0.27, respectively. Insets: Corresponding PL images excited by 365 nm UV light. Panels a–d are reprinted from ref (606). Copyright 2017 American Chemical Society (e,f) Two additional methods of increasing Mn^2+^ doping level in CsPbCl_3_ NCs. Panel e is reprinted with permission from ref (608). Copyright 2017 John Wiley & Sons, Inc.

**Figure 58:**
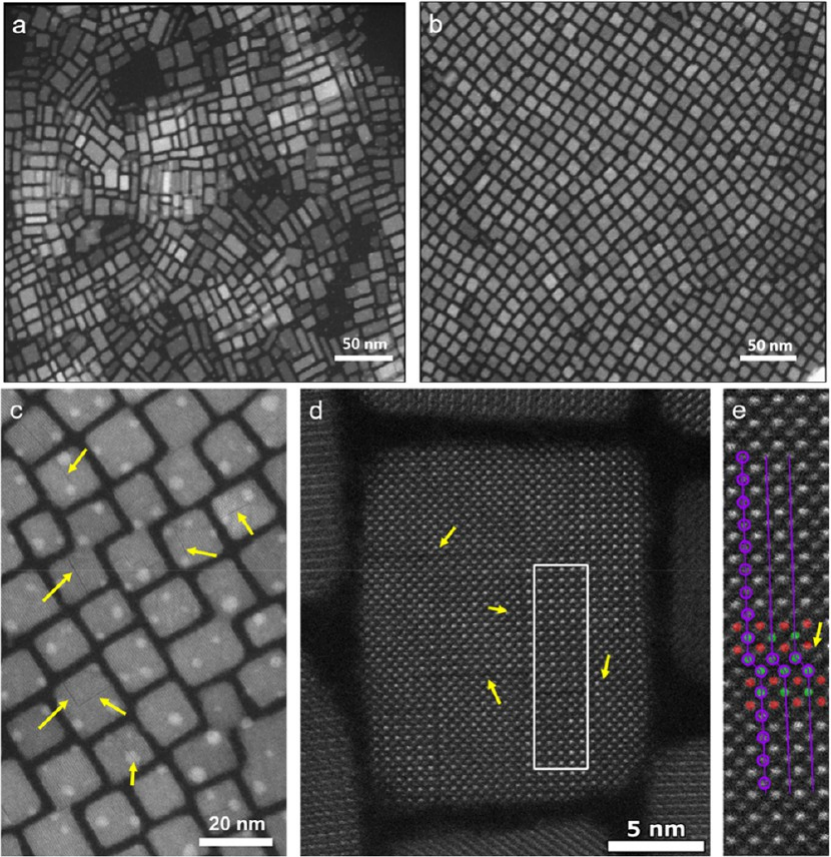
Overview TEM images of (a) pure and (b,c) Mn^2+^-doped CsPbCl_3_ NCs (Mn to Pb feed ratio 3:1), as shown in (c), with a large number of NCs have one or more line defects. (f,g) Corresponding atomically resolved HAADF-STEM image showing R–P defect planes (Pb/Mn–Cl = red, Cs = green). The lattices are shifted half of the unit cell at the grain boundaries. Panel a–e are reprinted with permission under a Creative Commons CC-BY 4.0 license from ref (602). Copyright 2020 John Wiley & Sons, Inc.

In later studies on Mn^2+^ doping in CsPbCl_3_ NCs, additional efforts were made to increase the doping concentration. In principle, heavily doped NCs should be called alloys rather than doped NCs, because doping in NCs generally refers only to a few dopants per NCs.^607^ However, most often heavily doped NCs are still called doped NCs.^606^ Herein, we do not make any difference between alloys and doped NCs for readers to avoid confusion with the current literature. Exploration of these heavily doped or alloy NCs were partially motivated by the desire to replace Pb with less toxic elements, and this is important for practical applications of perovskite NCs. For example, Liu *et al.* reported the Mn^2+^ substitution ratio is up to 46% and a luminescence quantum yield of 56% in CsPbCl_3_, which was achieved using the higher Mn/Pb ratio in the reactant mixture (Figure 57a–d).^606^ Das Adhikari *et al.* reported another method of increasing the Mn^2+^ doping concentration by using oleylammonium chloride as an additional reactant (Figure 57e).^608^ In addition to the hot-injection doping, room-temperature Mn^2+^ doping was also demonstrated. Xu *et al.* reported Mn^2+^ doping at room temperature using non-halide Mn^2+^ precursors.^609^ In their report, they used metal acetate salts as the precursor, which were converted to metal-oleate complexes in the presence of ligands, and then added HCl to protonate the carboxylate group, increasing the amount of monomer initiating the formation of nanocubes. The presence of HCl also promoted a Cl-rich surface, supplying ample binding sites for Mn^2+^ ions and facilitating Mn^2+^ doping. Further coating of Mn^2+^-doped CsPbCl_3_ with an additional CsPbCl_3_ shell improved the Mn^2+^ luminescence quantum yield. Recently, Paul *et al*.^602^ reported that the size distribution of CsPbCl_3_ NCs significantly improves with slight doping of Mn^2+^ ions during their synthesis by an ultrasonication approach (Figure 58a,b). This results in a prominent excitonic resonance for Mn^2+^-doped CsPbCl_3_ NCs as compared to pure CsPbCl_3_ NCs. (the reader is directed to the OPTICAL PROPERTIES section for more details). Interestingly, it was observed that Mn^2+^ doping leads to the formation of R–P defects within the host NCs, in which (Pb/Mn)–Cl atomic columns were shifted by half a unit cell at the border of the defect planes (Figure 58c–e), thus inducing quantum confinement within the host NCs. This results in a gradual blue shift of excitonic absorption and PL peaks. The authors hypothesized that the formation of such R–P defects may be triggered by the size difference between Mn^2+^ (1.6 Å) and Pb^2+^ (2.38 Å) ions.

**Figure 59:**
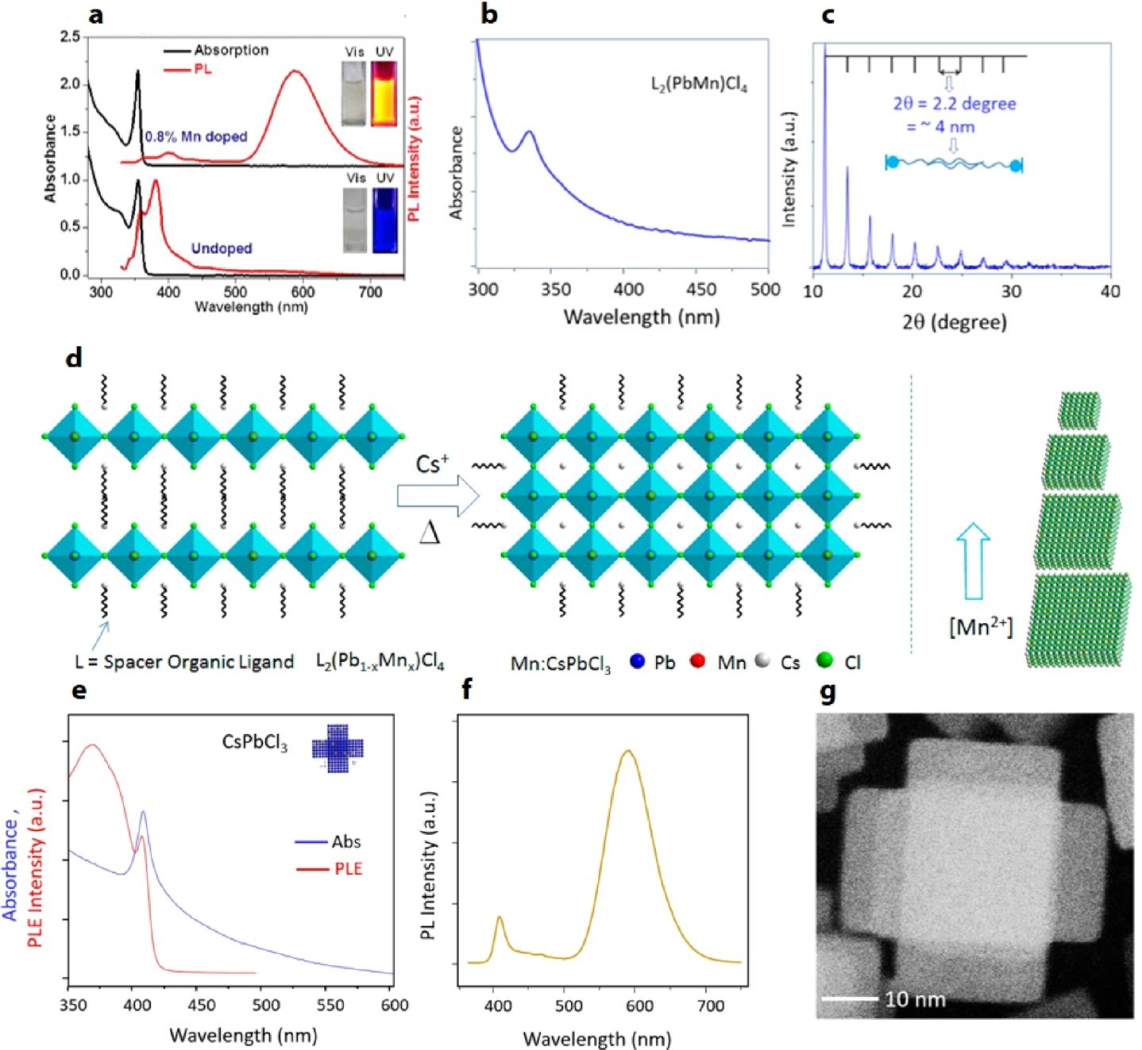
Mn^2+^ doping of anisotropic CsPbCl_3_ NCs. (a) Absorption and PL spectra of undoped and 0.8% Mn^2+^-doped CsPbCl_3_ nanoplatelets. Panel a is reprinted from ref (238). Copyright 2017 American Chemical Society. (b) Absorption spectra of the layered perovskites. The peak at 334 nm is characteristic of the monolayered structures. (c) Powder X-ray diffraction pattern of the layered perovskites. The interpeak spacing was 2.2° (2θ), which corresponds to ∼4 nm. (d) Schematic presentation of formation of doped perovskites from layered perovskites L_2_(Pb_1–*x*_Mn_*x*_)Cl_4_. L stands for *n*-butylammonium and oleylammonium ions. The schematic shows Mn^2+^ concentration in the reaction mixture to control the size of Mn^2+^-doped platelets. With the increase of the amount of Mn^2+^ in layered perovskites, the surface area of the platelets decreases. Panels b–d are reprinted with permission from ref (610). Copyright 2018 American Chemical Society. (e) Absorption and PLE spectra of Mn^2+^-doped CsPbCl_3_ hexapod nanostructures. PLE was measured at Mn^2+^ PL maxima. (f) PL spectra of Mn^2+^-doped CsPbCl_3_ armed structures. Excitation wavelength was 350 nm. (g) HRSTEM of Mn^2+^-doped CsPbCl_3_ hexapod. Panels e–g are reprinted from ref (611). Copyright 2019 American Chemical Society.

Although earlier studies focused on Mn^2+^ doping in cube-shaped CsPbCl_3_ NCs with very weak quantum confinement, more recent studies reported the synthesis of CsPbCl_3_ NCs of different morphologies, such as NPls with strong confinement and branched structures. Mir *et al.* synthesized Mn^2+^-doped CsPbCl_3_ NPls of thickness 2.2 nm, which exhibit strong quantum confinement along the thickness direction (Figure 59a).^238^ Das Adhikari *et al.* reported another method of doping Mn^2+^ in CsPbCl_3_ NPls, which involves the initial synthesis of a Mn^2+^-doped monolayer structure and subsequent formation of NPls by the addition of cesium-oleate.^610^ They synthesized 5-nm-thick NPls with different lateral sizes (20–580 nm) that varied with the amount of Cs^+^ and Mn^2+^ employed in the reaction (Figure 59b–d). Quantum confinement of the exciton in Mn^2+^-doped semiconductor NCs can enhance the exciton–dopant interaction, which determines various magneto-optical properties. Continued progress in the synthesis of strongly confined Mn^2+^-doped perovskite NCs is important for expanding their applicability. In another study, Mn^2+^-doped CsPbCl_3_ branched hexapods were synthesized using a seeded growth approach.^611^ Cores were first formed under halide-deficient conditions. In the second step, the reaction was enriched with halides to facilitate the arm growths. In the presence of Mn^2+^ precursor in the second step, the final product consisted of Mn^2+^-doped branched CsPbCl_3_ NCs (Figure 59e–g).^611^

#### Mn^2+^ Doping in CsPbBr_3_ NCs

Most of the work on Mn^2+^ doping of CsPbX_3_ NCs has focused on CsPbCl_3_ despite its less desirable optical properties than other halide systems (higher band gap and lower luminescence quantum yield). This is because doping of Mn^2+^ is most favorable in CsPbCl_3_ host and becomes increasingly more difficult for bromide and iodide perovskite NCs. Simply extending the doping method used for producing Mn^2+^-doped CsPbCl_3_ NCs described above did not produce Mn^2+^-doped CsPbBr_3_ NCs. It was hypothesized in the work by Liu *et al.* that direct hot-injection synthesis of Mn^2+^-doped CsPbBr_3_ using MnBr_2_ was energetically unfavorable owing to the large difference in bond energy between Pb–Br (249 kJ/mol) and Mn–Br (314 kJ/mol) compared to that between Pb–Cl (301 kJ/mol) and Mn–Cl (338 kJ/mol).^605^ The authors argued that the higher stability of the Mn–Br bond compared to the Pb–Br bond prevented the incorporation of Mn^2+^ into the CsPbBr_3_ lattice. Because of the difficulty of direct Mn^2+^ doping in CsPbBr_3_ NCs, various post-synthesis doping methods were developed.

**Figure 60:**
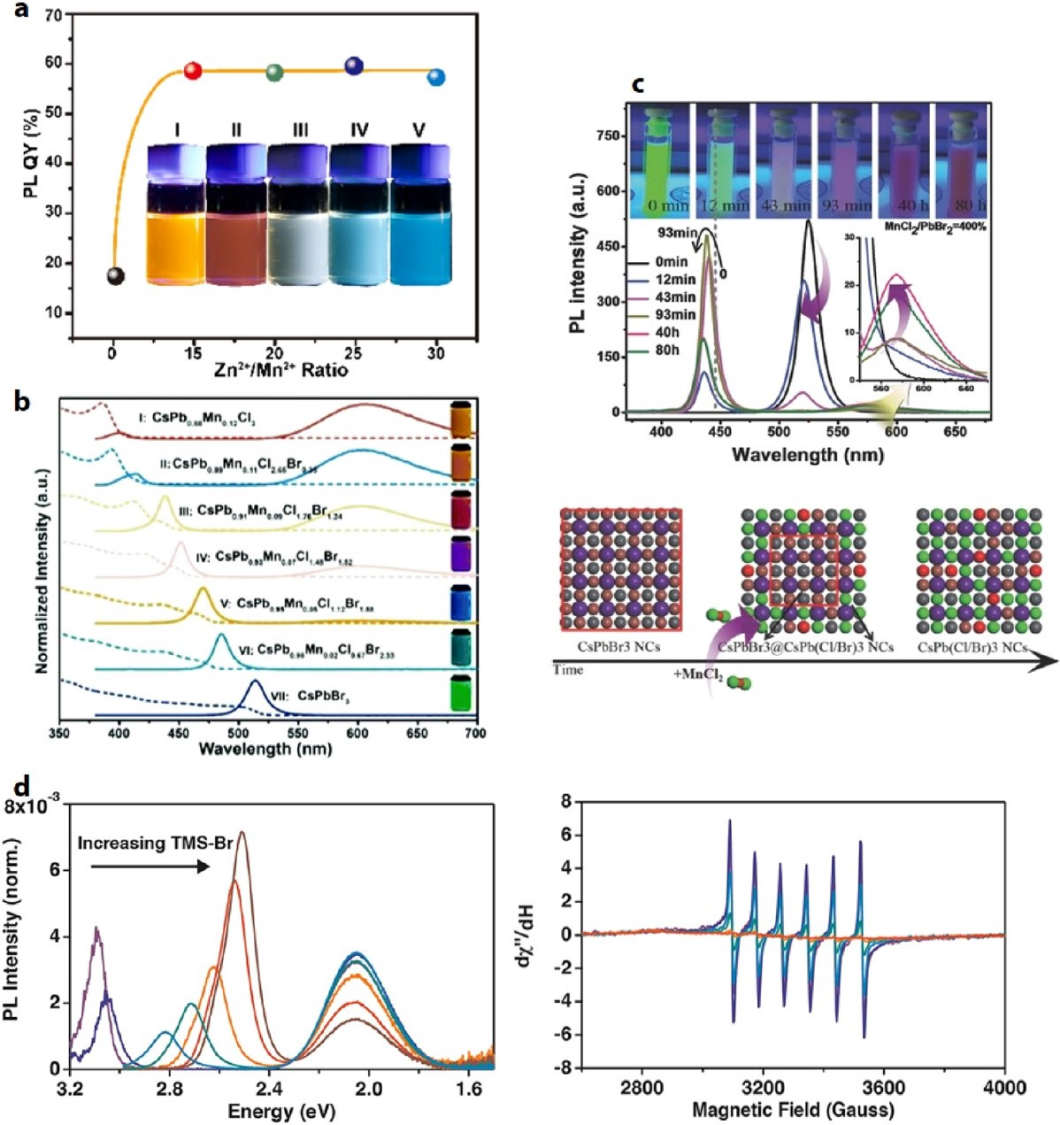
Post-synthesis anion exchange of Mn^2+^-doped CsPbCl_3_. (a) PLQY *vs* Mn^2+^ content of the initial and ion exchanged CsPb_0.75_Cl_3_:0.25Mn^2+^ NCs. The initial NCs have a low QY of 17.8%. After ion exchange, the QY of the samples sharply increases to 59.3% and maintains that level with the increasing ion exchange reaction time. The photographs of the pristine CsPb_0.75_Cl_3_:0.25Mn^2+^ NCs and ion-exchanged NCs with different reaction times under 365 nm UV lamp illumination. The color changes come from the decrease of the Mn^2+^ content. Reprinted from ref (311). Copyright 2018 American Chemical Society. (b) UV–vis absorption (dashed line) and photoluminescence spectra (solid line) of Cs(Pb_*x*_Mn_1–*x*_)(Cl_*y*_Br_1–*y*_)_3_ NCs. Reprinted with permission from ref (612). Copyright 2017 Royal Society of Chemistry. (c) Temporal evolution of PL spectra of CsPbBr_3_ NCs after adding the MnCl_2_ precursor. The inset is the corresponding digital photograph at different times under the irradiation of a 365 nm UV lamp. Sketch of the ion exchange process from pure CsPbBr_3_ NCs *via* adding MnCl_2_ precursor. Images were taken with permission from ref (613). Copyright 2017 John Wiley and Sons. (d) PL spectra (left) and EPR spectra (right) of 1.1% Mn^2+^-doped CsPbCl_3_ NCs in the EPR tube during the course of an anion exchange reaction; note that Mn^2+^ PL is seen centered at ∼610 nm at every stage of the anion exchange reaction. The PL spectra are each normalized to their total integrated PL intensity. A 365 nm diode was used for excitation. Each spectrum was taken at the same NC concentration, and the NCs were never removed from the EPR tube over the entire experiment. Panels d and e are reprinted from ref (614). Copyright 2019 American Chemical Society.

In an early attempt by Li *et al.*(311) post-synthesis halide exchange reaction on Mn^2+^-doped CsPbCl_3_ was attempted, but this was only partially successful. The halide exchange of Mn^2+^-doped CsPbCl_3_ with Br- using ZnBr_2_ salt dissolved in the mixture of hexane and oleylamine as the precursor resulted in not only the exchange of halide but also removal of doped Mn^2+^ ions in the host NCs (Figure 60a).^311^ Cation exchange from Pb^2+^ to Mn^2+^ in CsPbBr_3_ NCs using MnCl_2_ was also attempted by Li *et al*. (Figure 60b).^612^ However, this approach also suffered from the halide exchange from Br^–^ to Cl^–^, forming Mn^2+^-doped CsPb(Cl/Br)_3_ NCs with mostly Cl^–^ occupying the anion sublattice. Huang *et al.* reported another post-synthesis Mn^2+^ doping method based on halide exchange-driven cation exchange (Figure 60c).^613^ In this method, the addition of MnCl_2_ solution dissolved in DMF to the colloidal solution of CsPbBr_3_ NCs in toluene resulted in the production of Mn^2+^-doped CsPb(Cl/Br)_3_ NCs. Doping of Mn^2+^ was facilitated by the halide exchange, which was conjectured to be the result of simultaneous opening up of the rigid halide octahedron structure around Pb^2+^ as well as the Pb^2+^ to Mn^2+^ cation exchange. However, the approach has the same limitation of obtaining mixed-halide phase after doping, since using MnBr_2_ solution did not result in Mn^2+^ doping. Qiao *et al.* and Parobek *et al.* extended the halide exchange-driven cation exchange approach as a method for Mn^2+^ doping by combining photoinduced halide exchange.^312,615^ In this method, halide was provided *in situ via* photoinduced reductive dissociation of the solvent (CH_2_Br_2_) near the surface of the NCs, and a non-halide Mn^2+^ salt was used as the Mn^2+^ source. This approach was able to dope Mn^2+^ in small CsPbBr_3_ NCs, however, the intensity of the Mn^2+^ luminescence was relatively low, indicating a lower doping concentration.

In Mn-doped CsPb(Cl/Br)_3_ NCs with mixed-halide composition, the characteristic Mn^2+^ luminescence is still observed since the band gap of the host NCs is still sufficiently high to enable the sensitization of Mn^2+^ transition. However, the Mn^2+^ emission intensity decreases as the Br^–^ content increases in the mixed-halide NCs. Initially, this was explained by a work from Meijerink and co-workers attributing the decrease in Mn^2+^ emission to thermally assisted back energy transfer from Mn^2+^ to the host NCs, similar to Mn^2+^-doped CdSe.^616^ This point was argued by Gamelin and co-workers and they showed the presence of the exciton emission at 4 K, whereas in Mn^2+^-doped CdSe the Mn^2+^ emission is only present due to the lack of any thermal-related back energy transfer. The mixed-halide perovskite exhibited a temperature-dependent behavior similar to Mn^2+^-doped CsPbCl_3_, which has also a higher energy gap for thermally assisted back energy transfer to occur.^617^ Instead, Gamelin and co-workers attributed the change in PL properties to the clustering of Mn^2+^ in the lattice as the anion is exchanged from Cl to Br. They supported this by performing anion exchange from Mn^2+^-doped CsPbCl_3_ to Mn^2+^-doped CsPb(Cl_1–*x*_Br_*x*_)_3_ with TMS-Br while showing the retention of Mn^2+^ emission but the disappearance of the EPR signal (Figure 60d).^614^

Another avenue toward post-synthesis doping of Mn^2+^ was reported by Mir *et al.*, who used slightly different solvent conditions and were able to dope Mn^2+^ in CsPbBr_3_ NCs without concomitant halide exchange.^618^ In this modified approach, Mn^2+^ doping was achieved using MnBr_2_ dissolved in the mixture of toluene and acetone, where MnBr_2_ and CsPbBr_3_ can coexist due to moderately polar environment of the mixed solvent. It was conjectured that Mn^2+^ doping under these conditions takes advantage of the dynamic nature of binding of a ligand to adsorb dopants on the surface of NCs and fast halide migration to incorporate dopants into the CsPbBr_3_ NCs, although the detailed mechanism was not fully understood. Employing the same approach, they were able to synthesize Mn^2+^-doped CsPbBr_3_ NCs with different morphologies, including nanocubes and NPls. In the case of NPls, sensitized Mn^2+^ luminescence was observed due to the increased band gap from the quantum confinement. Pradhan and co-workers also employed a post-synthetic method to dope CsPbBr_3_ NPls by mixing them with MnBr_2_ in a toluene solution. They have co-related the local Mn^2+^-halide concentration with the change in emission intensity with dilution and preconcentration by evaporation of the dispersed solvent.^619^

**Figure 61:**
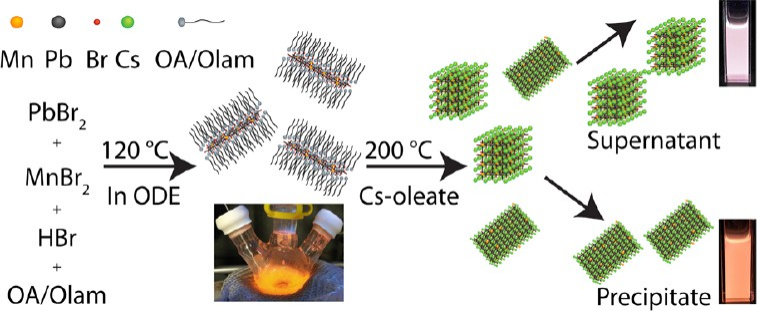
Mn^2+^ doping in CsPbBr_3_ NCs: Schematic representation of synthesis of Mn^2+^-doped CsPbBr_3_ NCs. Image was taken from ref (620). Copyright 2018 American Chemical Society.

Although the earlier attempts to dope Mn^2+^ in CsPbBr_3_ NCs *via* one-pot hot-injection synthesis only resulted in a NC with enhanced stability but no visible Mn^2+^ luminescence,^621^ Parobek *et al.* developed a direct hot-injection method that produces Mn^2+^-doped CsPbBr_3_ NCs *via* a two-step synthesis that exhibit Mn^2+^ luminescence (Figure 61).^620^ In the first step of the synthesis, a Mn^2+^-doped monolayer 2D structure is synthesized (L_2_[Pb_*x*_Mn_1–*x*_Br_4_], where L is a ligand) as an intermediate species. The presence of the intermediate 2D structure doped with Mn^2+^ was confirmed by small-angle X-ray diffraction, which revealed the presence of stacked 2D layers with 4.1 nm interlayer spacing. Further confirmation of Mn^2+^ doping within the 2D structure came from the absorption spectrum and photoluminescence excitation spectrum at 620 nm where the Mn^2+^ luminescence is observed. In the second step, the intermediate structure was converted to Mn^2+^-doped CsPbBr_3_ NCs by adding Cs-oleate at 200°C. Interestingly, the resulting product was a mixture of Mn^2+^-doped CsPbBr_3_ NCs with two different morphologies, *i.e*., nanocubes (6.5–8.5 nm) and NPls (∼2 nm in thickness), which were separated from each other *via* centrifugation. Since both Mn^2+^-doped CsPbBr_3_ NCs have sufficiently high band gap, due to quantum confinement, sensitized Mn^2+^ luminescence was observed in this work.

#### Mn^2+^ Doping in CsPbI_3_ NCs

Doping of Mn^2+^ in CsPbI_3_ NCs has also been reported by several groups. Akkerman *et al.* reported the synthesis of Mn^2+^-doped CsPbI_3_ using MnI_2_ as an additional reactant added in the hot-injection synthesis of CsPbI_3_ NCs.^607^ Mn-doped CsPbI_3_ NCs with ∼12 nm size were obtained from this synthesis. Unlike in Mn^2+^-doped CsPbCl_3_ and CsPbBr_3_ NCs, the band gap of CsPbCl_3_ NCs is smaller than the d–d ligand field transition energy of Mn^2+^, which prevents sensitization of the Mn^2+^ luminescence. This also makes it more challenging to confirm doping by spectroscopic techniques. On the other hand, the purpose of that work was to stabilize the perovskite phase and prevent its transition to the δ-CsPbI_3_ non-perovskite phase, as will be discussed later in more detail. In another work, Mir *et al.* reported post-synthesis Mn^2+^ doping using MnI_2_ dissolved in methyl acetate as the precursor of Mn^2+^. In their reaction, doping was achieved at room temperature by mixing the solutions of CsPbI_3_ NCs and MnI_2_.^601^

#### Sensitized Mn^2+^ Luminescence and Energy Transfer Dynamics

So far, the most studied optical properties of Mn^2+^-doped CsPbX_3_ NCs are related to the sensitized Mn luminescence along with the competitive dynamics between the radiative recombination of exciton and energy transfer to Mn^2+^. The relative intensities of exciton and Mn^2+^-dopant emissions depend on various factors, including the doping density, the relative energetics of host NC band gap and the d–d transition of the Mn^2+^ involved in the sensitization, degree of quantum confinement in the host NCs and temperature. While a complete picture of the correlation between these variables and PL intensities has not yet emerged, several recent studies have provided additional insights on the energy-transfer dynamics and microscopic mechanisms based on temperature-dependent transient absorption and photoluminescence, as described below.

**Figure 62:**
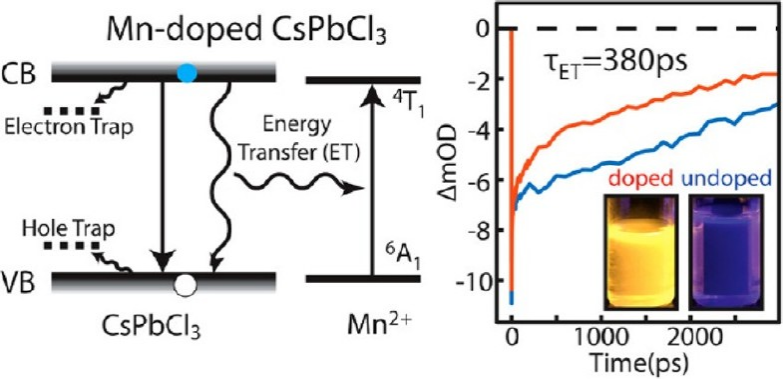
Dynamics of Mn^2+^-doped CsPbCl_3_: Scheme of exciton to dopant energy transfer in Mn^2+^-doped CsPbCl_3_ NCs and transient absorption data for doped and undoped CsPbCl_3_. Images were taken from ref (622). Copyright 2017 American Chemical Society

For the Mn^2+^-doped CsPbX_3_ NCs, Rossi *et al.* performed time-resolved experiments to directly measure the rate of energy transfer instead of estimating it from the luminescence quantum yield and relative intensities of luminescence from the host and Mn^2+^.^622^ In their study, the energy-transfer time (τ_ET_) was obtained by a comparative analysis of the recovery time of the bleach signal at the band-edge in Mn^2+^-doped and undoped NCs using pump–probe transient absorption spectroscopy. The energy-transfer pathway that exists only in Mn^2+^-doped NCs was manifested as an additional dynamic component in the bleach recovery of the exciton, as shown in Figure 62. In Mn^2+^-doped CsPbCl_3_ nanocubes with an edge length of 10 nm and a ∼0.4% doping concentration, τ_ET_ was determined to be ∼380 ps.^622^ Compared to the τ_ET_ value of the previously studied Mn^2+^-doped CdS/ZnS NCs after correcting for the difference in doping concentration, the τ_ET_ in the Mn^2+^-doped CsPbCl_3_ nanocubes is 2–5 times slower. The slower energy transfer in CsPbCl_3_ NCs compared to II–VI QDs was attributed to the intrinsically weaker exchange interaction among excitons and d electrons of the dopant in CsPbCl_3_ NCs and the weaker quantum confinement of the host NCs. De *et al.* also performed transient absorption spectroscopy in Mn^2+^-doped and undoped CsPbCl_3_ NCs and made a similar observation.^623^ They also observed the faster recovery of the bleach at the band-edge in Mn^2+^-doped NCs reflecting the energy transfer. So far, direct time-resolved studies have been limited to CsPbCl_3_ NCs with weak confinement. An indirect study on the rate of energy transfer based on relative intensities of exciton and Mn^2+^ PL was performed in CsPb(Cl/Br)_3_ NCs as a function of the halide composition. In the study by Xu *et al.*, the variation of *I*_Mn_/*I*_exc_ (ratio of Mn^2+^ and exciton photoluminescence intensity) with Br/Cl ratio in the host NCs was systematically studied.^616^ An initial fast increase in the I_Mn_/I_exc_ with increasing Br^–^ content is followed by a decrease for higher Br^–^ contents. The authors explained this observation by a reduced exciton decay rate and faster exciton to Mn^2+^ energy transfer upon Br^–^ substitution. Clearly, further investigation of other Mn^2+^-doped CsPbX_3_ NCs with different halide compositions and varying degrees of quantum confinement is necessary to obtain a better picture of the coupling between the exciton and dopant in this system.

**Figure 63:**
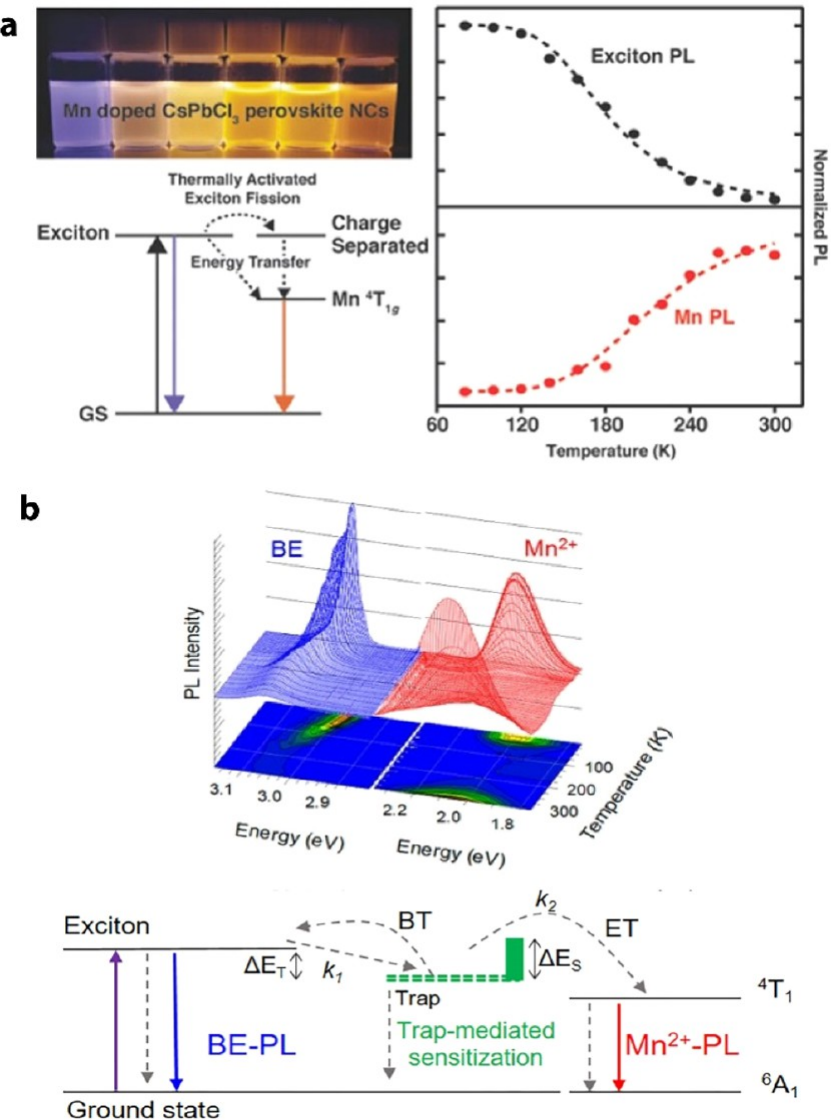
Temperature-dependent PL of Mn^2+^-doped CsPbCl_3_ NCs. Temperature-dependent exciton PL and Mn^2+^ PL intensities in Mn^2+^-doped CsPbCl_3_ NC films deposited on silicon substrates with 2.4% Mn^2+^, normalized at 80 K. Energy level diagram describing the energy tranfer process *via* thermally activated charge-separated state is also shown. Taken from ref (617). Copyright 2017 American Chemical Society. (b) 3D plot of PL spectra of the band-edge exciton (BE) and Mn^2+^ luminescence from Mn^2+^-doped CsPbCl_3_ NCs. Bottom figure is the Jabloski diagram showing the energy-transfer process through intermediate shallow trap state. Reprinted from ref (624). Copyright 2019 American Chemical Society.

A number of temperature-dependent studies on the intensity and lifetime of exciton and Mn^2+^ photoluminescence were performed by several groups, from which the involvement of a charge-separated state of exciton or trapped exciton in the energy-transfer process was inferred. Yuan *et al.* measured the temperature-dependent exciton and Mn^2+^ PL intensities at 80–300 K.^617^ Exciton PL increased with decrease in temperature in this range, whereas Mn^2+^ PL exhibited the opposite behavior (Figure 63). To explain the observed temperature-dependent PL intensities, the authors introduced a thermally activated charge-separated state that is longer-lived than the exciton and that also participates in the energy-transfer process. In this scheme, the temperature-dependent competition between radiative recombination of exciton and formation of charge-separated state ultimately determined the temperature-dependent competitive kinetics of exciton relaxation and energy transfer. More recently, Pinchetti *et al.* extended the range of temperature down to 5 K and studied the temperature-dependent branching between exciton recombination and energy transfer.^624^ The noteworthy observation is the reappearance of Mn^2+^ PL intensity below 70 K that increases with decrease in temperature, which contrasts to the trend at the higher temperatures. To explain the more complex temperature dependence of the PL intensities, the authors proposed a two-step process involving the initial localization of band-edge excitons in a shallow trap that mediates the sensitization of the dopants and repopulates the band-edge by thermally activated back-transfer. While this trap-mediated process was considered dominating above 70 K, the authors suggested that the barrierless energy transfer directly from band-edge exciton to Mn^2+^ occurs below 70 K, which explains the reemergence of Mn^2+^ PL at the lower temperatures.

### B-Site Doping to Stabilize Red-Emitting CsPbI_3_ NCs

The assessment of the Goldschmidt’s tolerance factor (*t*) using ionic radii is very popular among the halide perovskite community, but one must remember that this was initially proposed for oxide and fluoride-based perovskites, for which only ionic interactions can be safely considered. However, in comparison with fluoride, the polarizability of iodide induces a covalent character to the bonding and the traditional calculation of *t* does not clearly account for the stability of the perovskite system. Travis *et al.* correlated different experimental result to obtain the exact radii, and they found that the radii of Pb(II) in chloride, bromide, and iodide are 0.99, 0.98, and 1.03 Å, respectively, that is, significantly shorter than the Shannon ionic radius (1.19 Å).^51^ Hence, they proposed a modified *t* calculation with the experimentally obtained radii values. After considering all these the calculated *t* for CsPbI_3_ is 0.89, which is on the margin of the stable perovskite structure. In that case, the red emissive α-CsPbI_3_ NCs degrade into the yellow non-emitting δ-CsPbI_3_ phase after few days of preparation.

**Figure 64:**
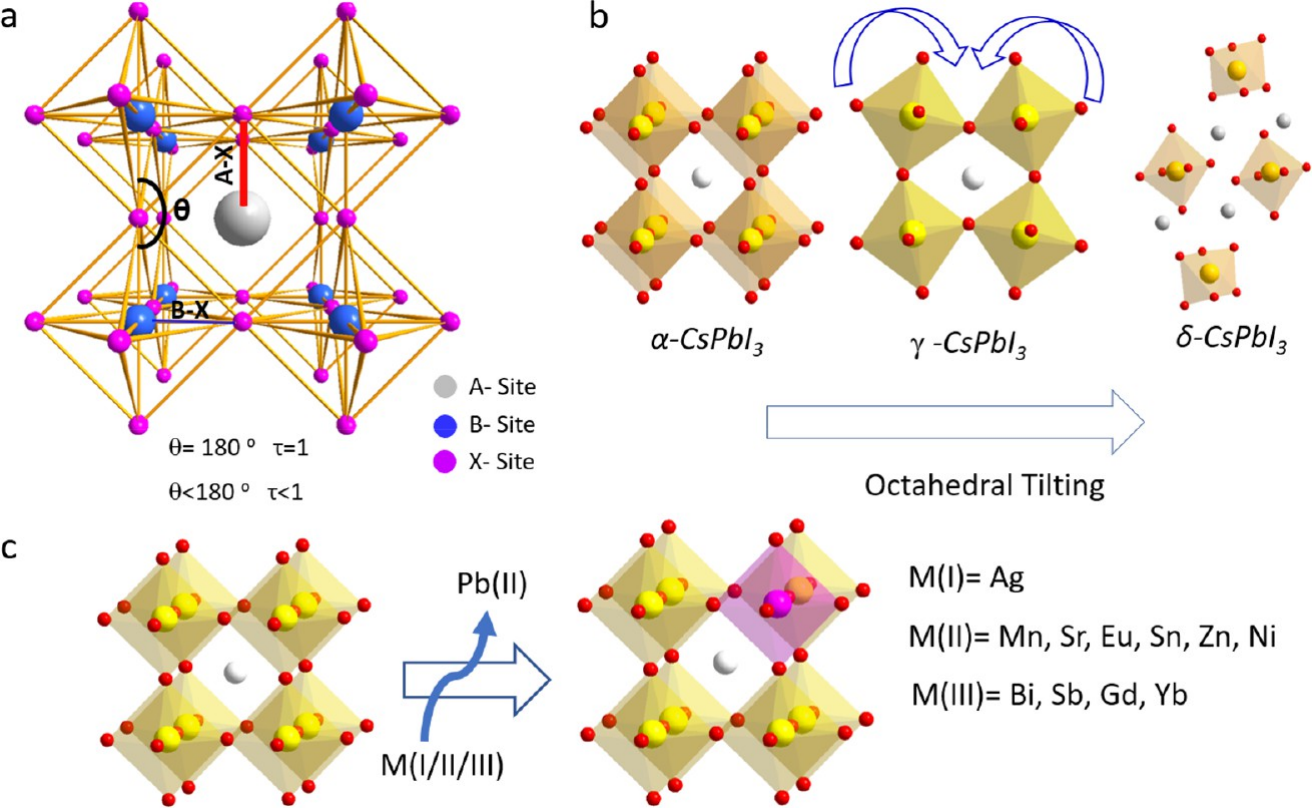
(a) Schematic representation of a typical ABX_3_ unit cell showing bond lengths, tilting angle (θ), and relationship of tilting angle (θ) with Goldschmidt tolerance factor (*t*). Reproduced from ref (625). Copyright 2019 American Chemical Society. (b) Various ABX_3_ unit cells with increasing tolerance factor. (c) Schematic illustration of B-site doping with various reported metal ions.

**Figure 65:**
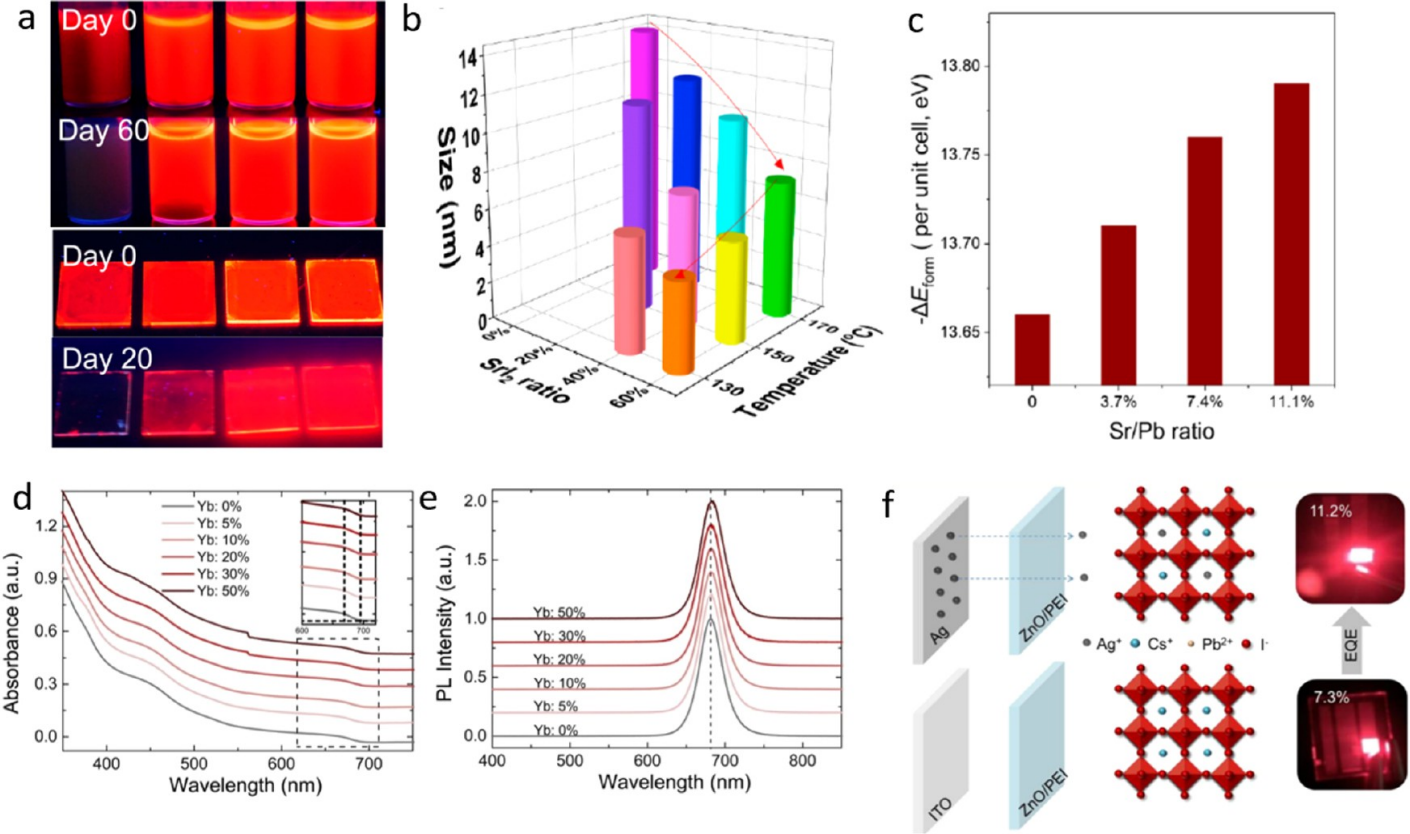
(a) Digital image of films and NC suspension of CsPbI_3_ NCs prepared with 0% SrI_2_ at 170 °C, 40% SrI_2_ at 170 °C, 60% SrI_2_ at 170 °C, and 60% SrI_2_ at 150 °C. (b) Plots showing the change of size of NCs with the amount of SrI_2_ introduction at different temperatures. (c) Histogram showing change in formation energy with change in Sr to Pb ratios. Images a–c were obtained from ref (627). Copyright 2019 American Chemical Society. Absorption (d) and PL spectra (e) of CsPbI_3_ NCs synthesized at various loadings of Yb. Inset of (d) is the enlarged view of the band-edge of all absorption spectra. The spectra shows that the band gap remains unaltered regardless of the amount of Yb doping. Panels d and e were reproduced with permission from ref (628). Copyright 2019 Royal Society of Chemistry. (f) Films of CsPbI_3_ NCs with and without Ag(I) and their respective lighting LEDs. Panel f is reproduced from ref (631). Copyright 2018 American Chemical Society.

It has been widely reported that the stability of the black perovskite phase of CsPbI_3_ NCs can be significantly improved by doping or alloying them with a divalent cation of a smaller ionic radius than that of Pb^2+^, which leads to an increase in *t*. A schematic of the B-site doping and the various dopant ions studied to date are illustrated in Figure 64c. For instance, Akkerman *et al*. have shown that alloying of α-CsPbI_3_ NCs with Mn^2+^ leads to a significant enhancement in their stability while preserving the optical features and crystal structure of pristine CsPbI_3_ NCs.^607^ The authors demonstrated that the CsPb_*x*_Mn_1–*x*_I_3_ NCs were stable over a month in either colloidal solution or thin films. The density functional calculations showed that the conduction and valence bands of CsPbI_3_ are influenced by both s and p orbitals of Pb and I respectively, while the Mn d-states remained far below the conduction band. Hence, Mn^2+^ doping did not alter the band gap or optical features of the pristine NCs. Similarly, alloying CsPbI_3_ NCs with Sn^2+^ also enhances their stability, but in this case it does influence the band gap of the NCs, hence their optical features.^530^ As discussed in earlier sections, CsSnI_3_ is not stable because of the ease of oxidation of Sn^2+^ to Sn^4+^. Interestingly, the alloyed CsSn_1–*x*_ Pb _*x*_ I_3_ NCs remained stable for more than 150 days. CsSnI_3_ and CsPbI_3_ have band gaps of 1.3 and 1.75 eV, respectively, and their alloyed NCs possess intermediate band gap. These works suggest that the selection of proper B-site dopants remains critical for preserving phase stability, but its influence on the optical properties of the NCs cannot be ignored. In another work, Shen *et al.*(626) demonstrated that alloying with Zn^2+^ reduces the nonradiative decay rates by suppressing the defect states in CsPbI_3_ NCs, and increases the radiative decay rates by enhancing the exciton binding energy of the NCs. Recently, Yao *et al.* reported the use of Sr(II) as a dopant to stabilize cubic-CsPbI_3_ NCs.^627^ As the ionic radius of Sr(II) is smaller than that of Pb(II), its inclusion in CsPbI_3_ NCs leads to the contraction of the crystal lattice and thus improves its phase stability. Figure 65a presents a photograph of the CsPbI_3_ NC suspensions and the corresponding films prepared under the addition of different percentages of SrI_2_ and the colloidal solutions and films after 60 and 20 days of preparation, respectively. In addition, the average size of the doped CsPbI_3_ NCs was found to be dependent on the SrI_2_ loading at various temperatures (Figure 65b),^627^ in analogy with other reports in which the concentration of halide ions in the synthesis is a key parameter for controlling the size of perovskite NCs.^79,149^ To support the experimental findings on increased stability, the authors further computed the formation energy of doped cubic CsPbI_3_ NCs, and it increases with increasing the the Sr to Pb ratios, as shown in Figure 65c.

In addition to isovalent doping/alloying, the introduction of heterovalent ions (*e.g*., Yb(III), Gd(III)and Sb(III)) ions was also explored to stabilize the cubic phase and preserve the red emission of the CsPbI_3_ NCs.^628−630^Figure 65d,e presents the absorption and PL spectra of CsPbI_3_ NCs with various amounts of Yb(III) doping. From the band-edge absorption spectra (inset of Figure 65d), the band gap was found to be unchanged regardless of the level of doping. In addition, the authors claimed that the PLQY increased from 75% to 86% with 20% Yb(III) doping, while it decreases at higher amounts of doping. The authors attributed this enhancement to reduction in the density of defects and trap states created by surface and lattice vacancies.^628^ In another work, Lu *et al.* found spontaneous Ag(I) doping in CsPbI_3_ film when an Ag film was used as an electrode in place of ITO in an LED device.^631^ In addition, they claimed that the Ag (I) ions passivate the CsPbI_3_ NC surface, leading to the increase of EQE from 7.3 to 11.2% using Ag electrode in the LED device (Figure 65f ).

**Figure 66:**
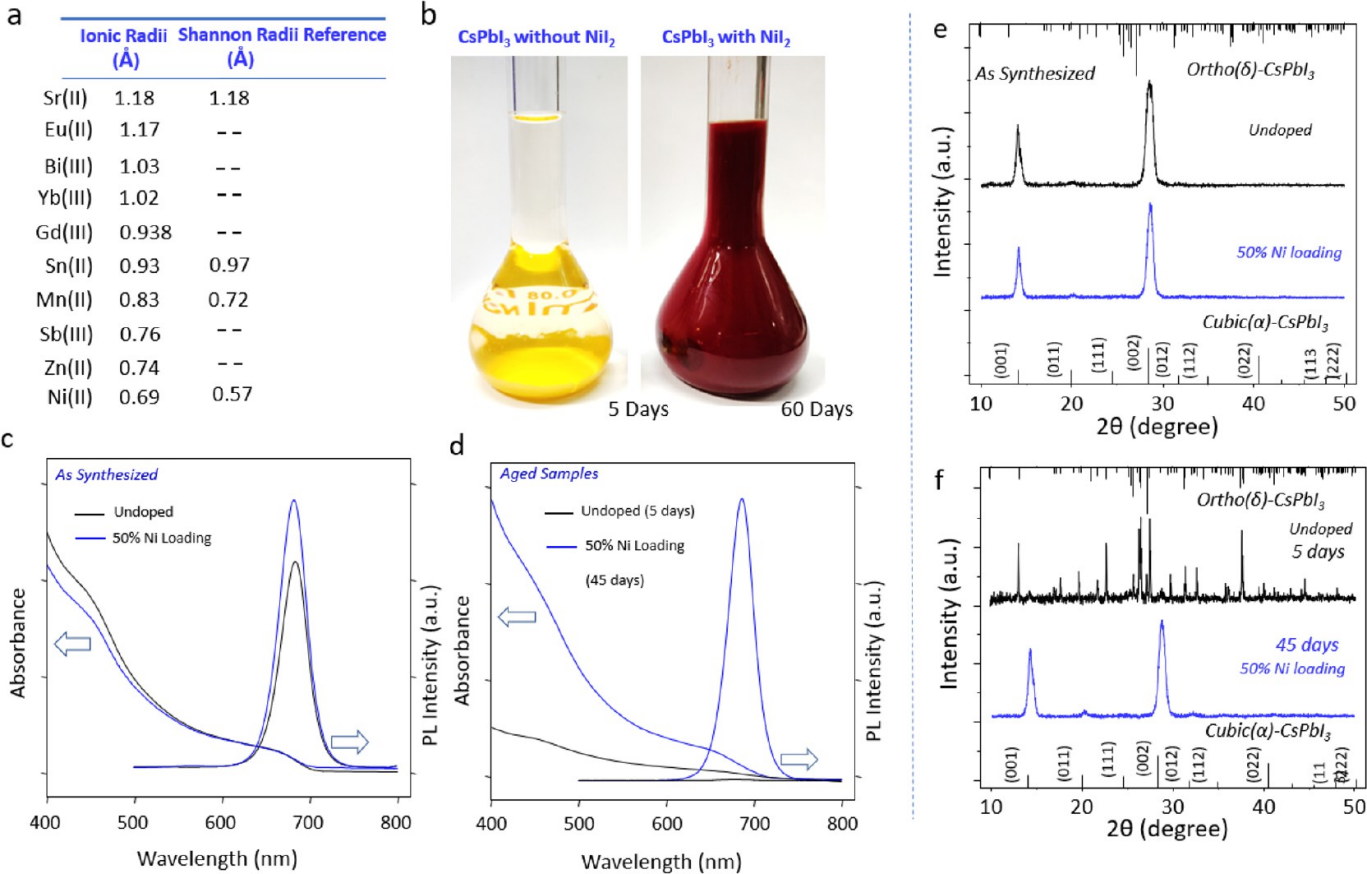
(a) Table showing ionic and Shannon radii of various metal ions used for stabilizing CsPbI_3_ NCs. (b) Photographs of CsPbI_3_ NCs dispersions without and with NiI_2_ addition in the respective syntheses. (c,d) Time-dependent absorption and PL spectra of as-synthesized and aged CsPbI_3_ NCs with and without NiI_2_ addition. (e,f) Powder X-ray diffractions of as-synthesized and aged samples with and without Ni-incorporated CsPbI_3_ NCs. These images were obtained from ref (632). Copyright 2019 American Chemical Society.

While analyzing the various reports on doping metal ions to achieve phase stabilization of red-emitting perovskite CsPbI_3_, Behera *et al.* found a correlation between temporal stability (either in solution or in the film ) and size of the B-site (*i.e*., the Pb^2+^ site) dopant ions of CsPbI_3_. A list of ions used as dopants, along with the available values of the corresponding Shannon radii, is provided in Figure 66a. Among these, Ni(II) has the lowest Shannon radius, and it was found that CsPbI_3_ NCs doped with this ion exhibited relatively longer stability.^632^ Photographs of the colloidal suspensions of Ni(II)-doped and undoped CsPbI_3_ NCs are shown in Figure 66b: the suspension containing undoped NCs turned yellow after 5 days, while the one containing the Ni(II)-doped NCs preserved its red color even after 45 days of aging. Figure 66c,d presents the absorption and PL spectra of suspensions of the undoped and Ni(II)-doped NCs (as-synthesized and aged). Both suspensions are red-emitting soon after their synthesis. However, while the undoped sample becomes non-emissive after 5 days (Figure 66d), the Ni(II)-doped sample remains strongly red-emitting even after 45 days. The phase change of the undoped sample after 5 days can be clearly seen in the powder X-ray measurements (Figure 66e,f).

As discussed above, most reports suggest that the doping or alloying in CsPbI_3_ NCs improves the phase stability, and thus the optical quality and stability. It has also been claimed that the doping removes nonradiative traps. In most cases, it was speculated that the divalent dopants occupy Pb positions of the crystal lattice. In some reports, theoretical supports were also provided for their experimental observations. However, there is no clear microscopic evidence for solid for doping of B-site to date. On the other hand, in most studies, respective iodide precursors were introduced for B-site doping, and this could lead to iodide-rich condition in the synthesis. For instance, Liu *et al.* promoted iodide-rich conditions in a typical CsPbI_3_ NC synthesis using GeI_2_ as an additional Iodide source.^633^ The authors claimed that the excess iodide in the reaction helped to stabilize the CsPbI_3_ NCs, however, unlike other bivalent metals Ge was not incorporated in the NC lattice. A similar observation was reported by Woo *et al.* using ZnI_2_ as an additional iodide precursor in the CsPbI_3_ NC synthesis.^397^ On the other hand, Imran *et al.*(178) and Cai *et al.*(168) separately reported the use of non-halide Pb and Cs precursors in the perovskite NC synthesis, in which the reaction was triggered by benzoyl iodide and trimethylsilyl iodide as iodide precursors, respectively. In both cases, stable CsPbI_3_ NCs were prepared. These results put under discussion the real need of doping in order to improve the stability of black-phase CsPbI_3_ NCs by replacing Pb(II) by mono-, di-, and trivalent dopant ions. Hence, in-depth experimental and theoretical studies are needed for better understanding and concluding the role of dopants in the stabilization of black-phase, red-emitting CsPbI_3_ NCs.

### Apparent A-Site Doping

**Figure 67:**
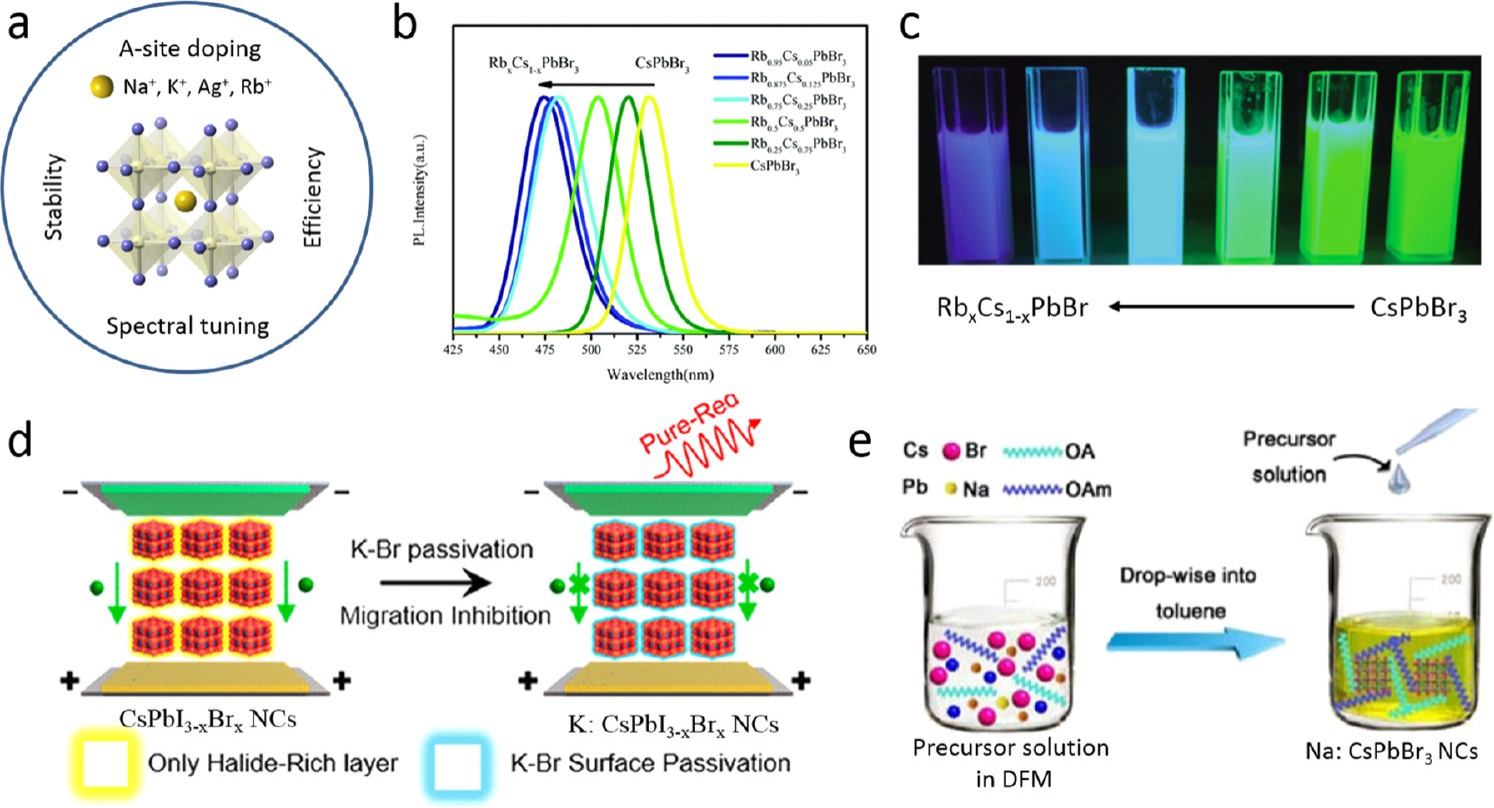
(a) Schematic illustration of the perovskite cubic crystal structure with possible A-site dopants including Na^+^, K^+^, Ag^+^, and Rb^+^. Through doping, enhanced stability, device efficiency, and spectral tuning have been achieved. (b) Photoluminescence spectra of Rb_*x*_Cs_1–*x*_PbBr_3_ colloidal solutions under 365 nm excitation. (c) Photograph of the colloidal solutions of Rb^+^-doped CsPbBr_3_ perovskite NCs under UV light illumination. Panels b and c are reproduced with permission from ref (583). Copyright 2018 Royal Society of Chemistry. (d) Schematic illustration of doping K^+^ into CsPbI_3–*x*_Br_*x*_ NCs by surface passivation to improve red photoluminescence. Reproduced from ref (400). Copyright 2020 American Chemical Society. (e) Schematic illustration of the synthesis of the Na^+^-doped CsPbBr_3_ NCs by ligand-assisted reprecipitation approach. Reproduced from ref (637). Copyright 2019 American Chemical Society.

In addition to the bivalent metal cation dopants discussed above, several monovalent cation dopants such as Rb^+^, Na^+^, K^+^, and Ag^+^ are also being intensively investigated to enhance the stability as well as photoluminescence efficiency of perovskite NCs (Figure 67a).^573,634^ It has been claimed that these dopants occupy A-sites of perovskite NC lattice. The selection of dopants is generally inspired from the previous research on perovskite solar cells, in which perovskite films were doped with various monovalent cations to improve their power conversion efficiency and stability.^108,573,634,635^ The phase stability of perovskites with specific monovalent cations depends on their size and thus tolerance factor as discussed above.^635^ For instance, Cs^+^, MA^+^, and FA^+^ ions fit well into the A-site of the lead iodide perovskite structure (Cs^+^ “less” well than MA^+^ and FA^+^, as discussed in the previous sections), while small metal ions such as Li^+^, Rb^+^, Na^+^, and K^+^ cannot stabilize the perovskite structure due to a low tolerance factor.^635^ Interestingly, doping these small cations into perovskite NCs improves their optical properties and phase stability. Saliba *et al.*(635) showed the incorporation of small and oxidation-stable Rb^+^ into mixed cation perovskite (CsMAFA) films to create photoactive perovskite films with excellent material properties. Interestingly, Rb^+^ incorporation does not alter the valence band position of the host perovskite. They have showed that the Rb^+^ doping into perovskites leads to higher phase stability and more reproducible power conversion efficiencies (PEC). Further studies revealed that Rb^+^ incorporation can also enhance the performance of the corresponding light-emitting diodes.^636^

Recently, the concept of Rb^+^ doping into bulk perovskites has been extended to perovskite NCs as well.^583,638−641^ For instance, Wu *et al.*(583) synthesized Rb^+^-doped CsPbBr_3_ perovskite NCs with different ratios of Rb/Cs by the hot-injection method. It was found that the band gap gradually increases and thus the photoluminescence blue shifts with the increase of the Rb/Cs ratio (Figure 67b,c). It is very interesting that the Rb_*x*_Cs_1–*x*_PbBr_3_ colloidal solution exhibit blue photoluminescence with increasing the Rb^+^ dopant concentrations. The authors attributed the increase in the band gap to changes in the valence and conduction bands caused by the decrease of in-plane Pb–Br–Pb bond angle of the [PbBr_6_] octahedron by the replacement of Cs^+^ with small Rb^+^ ions that does not fit well into perovskite lattice due to low tolerance factor.^583^ A similar blue shift in photoluminescence was observed for Rb_*x*_Cs_1–*x*_PbBrI_2_ NCs with increasing ratio of Rb/Cs.^642^ Furthermore, Rb^+^ ions can also be doped into perovskite nanoplatelets of different thicknesses to achieve tunable emission (green–sky blue–blue) with PLQY over 60%, as shown by Sargent and co-workers.^640^ The fabrication of sky-blue and deep-blue LEDs has been demonstrated using these mixed cation Rb_*x*_Cs_1–*x*_PbBr_3_ nanoplatelets, and they exhibit relatively high thermal stability and operational stability. Despite these few studies, the location (either on the surface or inside the lattice) of Rb^+^ ions in perovskite lattice is still unclear. Very recently, Etgar and co-workers^638^ performed EDS analysis on atomically resolved HAAD-STEM images of Rb_*x*_Cs_1–*x*_PbBr_3_ NCs to understand the position of Rb^+^ ions in the lattice. They claimed that at medium dopant concentrations the Rb^+^ ions stays in the core region, while the Cs atoms are preferentially located in the shell region, forming core–shell like structures. However, at high Rb^+^ dopant concentrations a phase separation of Rb^+^ occurs within the perovskite NCs, because Rb atoms cannot form the perovskite phase. In contrast, Kubicki *et al.*(643) performed ^14^N solid-state magic-angle spinning (MAS) NMR to probe the compositions of mixed cation (Cs, Rb, K, MA, FA) perovskites and they found no signs of Rb or K incorporation into the bulk perovskite lattice. From an X-ray photoelectron spectroscopy study, they found that the surface of perovskites has rubidium-rich phases, which can acts as a passivation layer for the perovskites.

In addition, other alkali metal ions including K^+^ and Na^+^ are also gaining attention as potential dopants for perovskite NCs to enhance their stability and photoluminescence efficiency. For instance, Huang *et al.*(353) reported a post-synthetic surface treatment of CsPbBr_3_ perovskite NCs with K-oleate to improve their PLYQ and photostability. After K^+^ treatment, the NC films retained their original photoluminescence intensity even after 150 h of illumination. However, it is not clear whether the K^+^ ions just passivated the NC surface or it diffused into the perovskite lattice. Similarly, CsPbI_3–*x*_Br_*x*_ NCs were treated with K-oleate to enhance red photoluminescence, as demonstrated by Yang *et al.* (Figure 67d).^400^ The addition of K-oleate led to the formation of KBr on the CsPbI_3–*x*_Br_*x*_ NC surface, which then passivated the NC surface effectively to obtain PLQY over 90% (Figure 67d). More importantly, the K^+^ ions were able to protect the NC surface from halide segregation, and the LED made using these NCs exhibited stable electroluminescence and high brightness. On the other hand, Na^+^ ions were incorporated into colloidal CsPbBr_3_ NCs by ligand-assisted reprecipitation approach, as shown in Figure 67e.^637^ It was found that the Na^+^-doped CsPbBr_3_ NCs exhibit better color purity and higher PLQY. This was attributed to the reduction of nonradiative trap centers in NCs by Na^+^ passivation. In addition, a gradual blue shift in the emission peak was observed with an increasing Na^+^ dopant concentration similar to Rb^+^-doped perovskite NCs. More importantly, the Na^+^-doped CsPbBr_3_ NCs had enhanced stability against ultraviolet light, heat, and moisture compared to pure CsPbBr_3_ NCs, and thus the white LEDs fabricated using these Na:CsPbBr_3_ NCs as phosphors showed superior stability even under continuous runs for over 500 h.^637^ In another report, Chen *et al.*(644) demonstrated the *in situ* incorporation of Na^+^ ions into CsPbBr_3_ NCs prepared directly on a substrate using NaBr additive in the precursor solution. The authors claimed that the added NaBr passivates the NC defects and also improves the conductivity of the films. More importantly, the green LEDs fabricated using Na:CsPbBr_3_ exhibited a maximum EQE of 17.4%, which is higher than the values measured on the LEDs prepared using pure CsPbBr_3_ NCs (EQE ∼ 12%).^644^ Based on the above discussed examples, it is clear that doping perovskite NCs with smaller monovalent cations improves their stability as well as PLQY, and thus the efficiency of LEDs. Despite these early studies, the mechanism of doping is rather unclear and the question regarding the position of dopants in the NCs (surface or inside crystal lattice) is yet to be addressed satisfactorily. Addressing this question requires a detailed analysis of atomically resolved HAAD-STEM images, but this is challenging, as the amount of dopants is rather small and perovskite NCs are quite prone to damage induced by electron beam irradiation. In addition, the relation between the concentration of dopants and the emission efficiency is yet to be investigated in detail. It is likely that, in all studied cases, there is an optimum dopant concentration that maximizes the PLQY, past which any additional doping may start actually degrading the emission efficiency.

### Lanthanide-Doped Perovskite Nanocrystals

Lanthanide ions are widely used as luminescence activators in inorganic materials.^645,646^ For example, the spectral conversion phosphors in fluorescent lighting use lanthanides as activators to emit visible photons following absorption of high-energy photons by the host material (either the host lattice itself or an additional “sensitizer” impurity). Trivalent lanthanides (Ln^3+^) are particularly excellent in this role. The high shielding of their 4f valence orbitals results in sharp-line f–f emission that is relatively insensitive to the crystalline field around the lanthanide. Furthermore, white light of almost arbitrary color temperature can be generated by combining several lanthanides.

The f–f internal transitions of the lanthanides are parity-forbidden and are only weakly coupled to lattice distortions that might relax this forbiddenness. Their radiative lifetimes are therefore often extremely long (*e.g.*, milliseconds). In crystalline or amorphous matrices with only low-energy vibrations, these ions frequently show very large photoluminescence quantum yields. In a minority of cases, the optical spectroscopy of the lanthanides is dominated not by f–f transitions but rather by f–d transitions. These specific cases include Ce^3+^ and divalent lanthanides, most commonly Eu^2+^. These f–d transitions are parity allowed, and because of the much greater interaction of the 5d orbitals with the surrounding environment, they are vibronically broadened and their energy is more sensitive to the specific ligand-field environment.

In fluorescent lighting, the phosphor matrices are generally oxides (*e.g.*, Eu^3+^-doped Y_2_O_3_ red phosphor) that are robust under the very short wavelength excitation of the mercury gas discharge (254 nm), and the materials need to absorb strongly at these short wavelengths. For other applications, greater visible-light absorption is desirable. Lanthanide-doped perovskite NCs have recently begun attracting broad attention as candidates for visible-light sensitized phosphors.^580,618,629,647−659,661−663,1354^ In contrast with the extensively studied Ln^3+^-doped fluoride NCs used as upconversion phosphors (*e.g.*, Ln^3+^:NaYF_4_, Ln^3+^:LaF_3_, *etc.*),^664−666^ Ln^3+^ emission in lead-halide perovskite NCs is generated by direct excitation of the semiconductor band-to-band transitions, which have oscillator strengths ∼10^5^ times greater than those of the Ln^3+^ f–f transitions. Additional materials with distinctive spectral characteristics have been created by combining the energy-tunable light-harvesting capabilities of metal-halide perovskite NCs with the excellent radiative properties of lanthanide dopants. These materials could be promising for applications such as solar spectral conversion and other related technologies.

**Figure 68:**
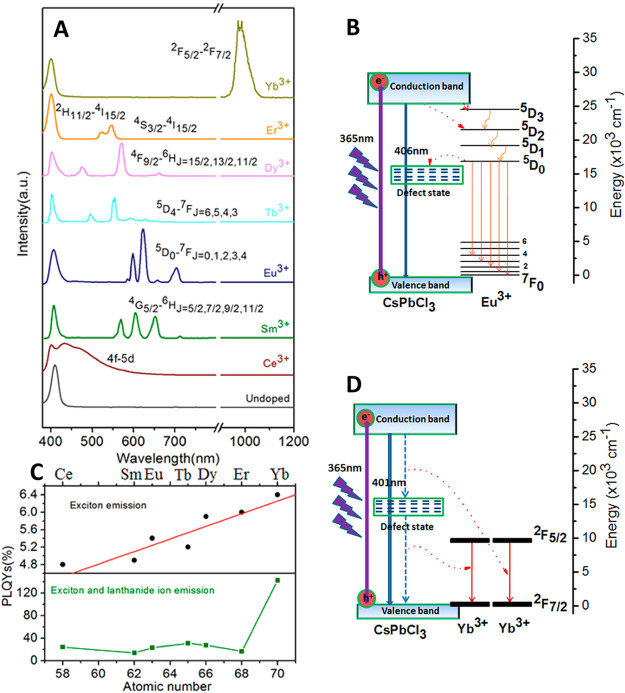
(A) Emission spectra of CsPbCl_3_ NCs doped with different lanthanide ions. (B) Proposed energy level diagram for Eu^3+^-doped CsPbCl_3_ NCs showing a possible photoluminescence mechanism. (C) Photoluminescence quantum yields for excitonic (top) and overall (exciton + Ln^3+^, bottom) emission features. (D) Proposed energy-level diagram for Yb^3+^-doped CsPbCl_3_ NCs showing a possible mechanism for quantum cutting *via* stepwise energy transfer involving a deep (mid-gap) defect state. Adapted from ref (663). Copyright 2017 American Chemical Society.

Lanthanide-doped lead-halide perovskite NCs appeared in 2017, when the Song group surveyed a series of Ln^3+^-doped CsPbCl_3_ and anion-alloyed CsPb(Cl_1–*x*_Br_*x*_)_3_ NCs involving the entire series of trivalent lanthanide ions.^661,663^Figure 68a shows the overview figure from one of these studies, organized from top to bottom according to decreasing 4f electron count of the Ln^3+^ dopant in CsPbCl_3_ NCs and referenced to the undoped CsPbCl_3_ spectrum. A few aspects of these data are notable. First, in each case (except Ce^3+^), the PL spectrum shows both excitonic PL and the characteristic f–f emission features of the lanthanide known from previous studies in analogous chloride host lattices. The Ce^3+^-doped NCs show broad emission near the perovskite band gap, attributed to the well-known f–d emission of this ion. For most cases, the sensitization scheme was considered to involve perovskite photoexcitation followed by nonradiative relaxation within the 4f manifold of excited states until a sizable energy gap was reached, at which point f–f emission is observed (Figure 68b).^663^

**Figure 69:**
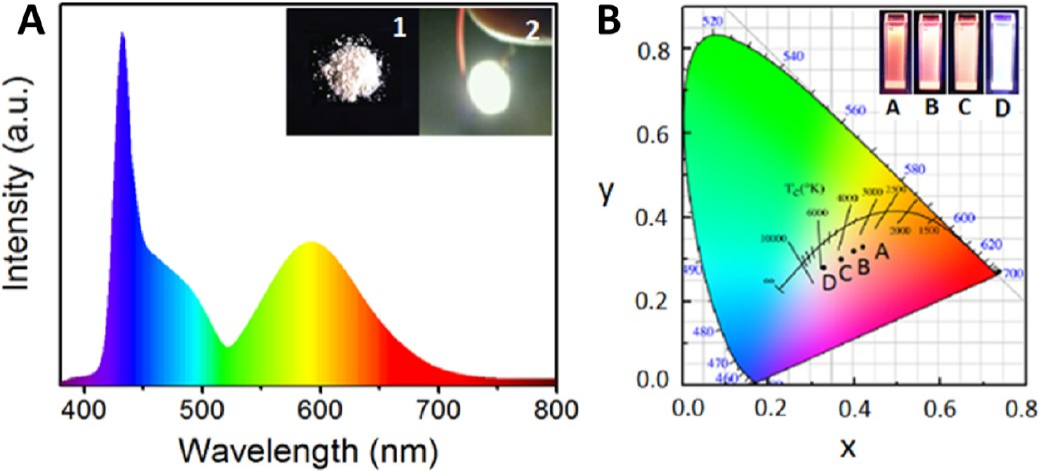
(A) Emission spectrum of a WLED based on 2.7% Ce^3+^/9.1% Mn^2+^-codoped CsPb(Cl_0.6_Br_0.4_)_3_ nanocrystals pumped by an underlying UV diode. Inset 1 shows the powdered phosphor composite made by mixing the NCs with polystyrene, and inset 2 shows a photograph of the operating device prepared by depositing the phosphor composite onto a 365 nm chip. (B) CIE chromaticity coordinate plot for WLEDs using Ce^3+^/Mn^2+^ codoped CsPb(Cl_1–*x*_Br_*x*_)_3_ NC phosphors [A(0.42, 0.33), B(0.39, 0.32), C(0.37, 0.30), and D(0.33, 0.29)]. The inset shows photographs of the PL from colloidal 2.7% Ce^3+^/9.1% Mn^2+^-codoped CsPb(Cl_1–*x*_Br_*x*_)_3_ NCs with different values of *x* under 365 nm excitation. Adapted from ref (652). Copyright 2018 American Chemical Society.

The Ln^3+^ PL sensitized by semiconductor photoexcitation has resulted in the use of such Ln^3+^-doped NCs in numerous phosphor applications, including lighting or display technologies, near-IR optics, and telecommunications. For example, the Song group subsequently demonstrated the use of CsPbCl_3_ and CsPb(Cl_1–*x*_Br_*x*_)_3_ NCs codoped with pairs of impurity ions, *e.g.*, Ce^3+^/Mn^2+^, Ce^3+^/Eu^3+^, Ce^3+^/Sm^3+^, Bi^3+^/Eu^3+^, and Bi^3+^/Sm^3+^, as spectral converters for white-light generation.^652^ Both ions in these pairs can be sensitized by the host NC, and they function roughly independently of one another, such that color rendering can be optimized by controlling the relative and absolute concentrations of each dopant (Figure 69). Particularly efficient white-light emission was achieved with Ce^3+^/Mn^2+^ codoping of CsPb(Cl_0.6_Br_0.4_)_3_ NCs. These NCs showed PLQYs of ∼75% and luminous efficiencies as high as 51 lm/W with good color rendering (∼89) when pumped at 365 nm from a UV LED chip. These performances demonstrate the lanthanide-doped perovskite NCs’ potential as promising alternatives to undoped NCs or other phosphors for lighting applications.

A second notable feature of the data in Figure 68a emerged from PLQY measurements (Figure 68c). For each dopant except Yb^3+^, the PLQY was modest, summing to a combined value of ∼25% split between the exciton and the visible lanthanide transitions. For Yb^3+^, however, the PLQY appeared to exceed 100%, reaching a value of ∼127% for the f–f transition and ∼20% for the exciton in the NCs shown in Figure 68a. PLQYs over 100% in Yb^3+^-doped crystals are rare but not unknown.^667−669^ The phenomenon, referred to as “quantum cutting”, has generally involved participation of pairs of Ln^3+^ ions with matched energy levels, such as one Pr^3+^ + two Yb^3+^ ions. In this case, it was proposed^663^ that the process requires only Yb^3+^ and the semiconductor NC, involving the suggested stepwise energy transfer shown in Figure 68d. Although the precise microscopic mechanism of quantum cutting remains uncertain at this time, quantum cutting in Yb^3+^-doped CsPbX_3_ NCs has now been observed in multiple laboratories, and it represents a major direction in the doping of NCs.

The Song group’s synthesis of Ln^3+^-doped CsPbX_3_ NCs involved the injection of cation precursors into organic solutions of anions at elevated temperatures (≳200 °C), akin to popular procedures for preparing undoped perovskite NCs. An alternative “inverted” approach involving injection of trimethylsilyl halide (TMS-X) precursors into organic solutions of the cation precursors was explored by the Gamelin group: they found that higher Yb^3+^ solubilities could be achieved by this approach, and that the resulting NCs showed correspondingly improved spectroscopic properties, specifically in the form of greater reduction of excitonic photoluminescence and greater Yb^3+^ f–f PLQYs, now approaching 200%.^650^ Other methods for doping Yb^3+^ into perovskite NCs have also been explored. The Nag group demonstrated post-synthetic doping of Yb^3+^ into not just CsPbCl_3_ NCs but also into NPls of both CsPbBr_3_ and CsPbI_3_ composition.^618^ Yb^3+^ doping was achieved by an interesting post-synthetic cation-exchange strategy, in which Yb(NO_3_)_3_ dissolved in a mixture of methyl acetate/toluene was added to NC dispersions under continuous stirring for only 1 min, followed by washing using MeOAc as the antisolvent. The process is thus analogous to that recently explored for post-synthetic Mn^2+^ doping of lead-halide perovskite NCs,^613,670^ but now involving Ln^3+^ ions. This interesting chemistry reflects the extreme fluidity of the perovskite lattice, and the ability to drive cation exchange reactions at room temperature. It is unclear whether these materials made by post-synthetic cation exchange also show the very high PLQYs of those made at high temperature, but future comparative studies could shed some insight into the participating defect structures if temperature is an important contributor to their formation or stability.

The Gamelin group proposed a concerted rather than stepwise mechanism for the microscopic quantum-cutting process.^650^ They observed picosecond exciton depletion associated with Yb^3+^ doping, which appeared too rapid for normal energy transfer to Ln^3+^ ions, and hence the participation of a dopant-induced defect state was hypothesized. In this mechanism, energy is first transferred to this defect state, where it subsequently bifurcates to excite two Yb^3+^ ions simultaneously. The hypothesis of a participating shallow dopant-induced defect state is supported by the observation of similar rapid exciton depletion as well as near-band-edge trap-state emission when Yb^3+^ is replaced by spectroscopically inactive Ln^3+^ ions (*e.g.*, La^3+^).^650^ Time-resolved measurements have confirmed the presence of an intermediate state, showing a *ca.* 7 ns rise time of Yb^3+^ photoluminescence at room temperature.^1351^ Beyond this, the microscopic details remain unclear. Because no mid-gap intermediate state is involved, the excitations of the two Yb^3+^ ions must be correlated and this mechanism therefore predicts correlated emission from these two Yb^3+^ ions, but such correlation remains to be demonstrated. The Gamelin group also noted that the excess charge of Yb^3+^ requires compensation and speculated that this compensation may be accomplished by substituting three Pb^2+^ ions with only two Yb^3+^ ions, thereby creating a Pb^2+^ vacancy (V_Pb_), by analogy to the well-known McPherson pair motif in related metal-halide lattices.^650^ Computational work has suggested that a bent charge-neutral Yb–Cl–V_Pb_–Cl–Yb defect cluster could indeed give rise to such a concerted energy transfer, and has identified accumulation of charge density on neighboring Pb^2+^ ions as important in the microscopic energy-transfer mechanism.^1355^

**Figure 70:**
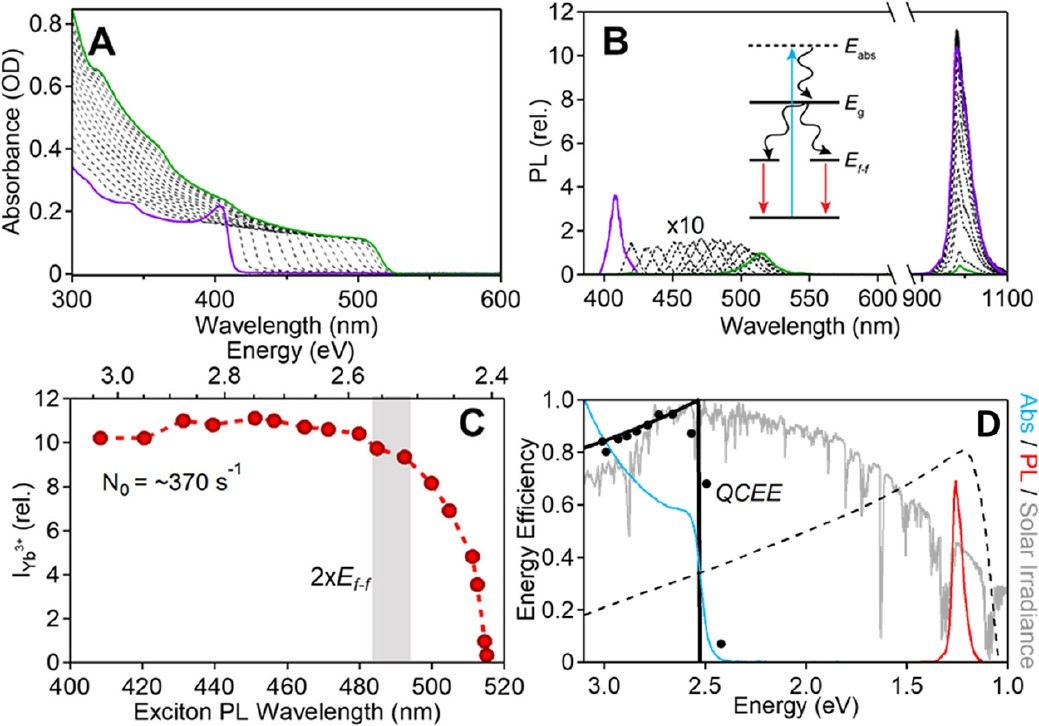
(A) Absorption spectra of 7.7% Yb^3+^:CsPb(Cl_1–*x*_Br_*x*_)_3_ NCs monitored during anion exchange from Yb^3+^:CsPbCl_3_ (purple) to Yb^3+^:CsPbBr_3_ (green). (B) PL spectra collected *in situ* during the reaction of panel A. PL spectra were measured using a constant NC excitation rate. The inset illustrates the quantum-cutting process. (C) Plot of the Yb^3+^^2^F_5/2_ → ^2^F_7/2_ PL intensity *vs* the exciton PL wavelength, from the spectra in panel B. The gray shaded area marks approximately twice the Yb^3+^(^2^F_7/2_ →^2^F_5/2_) absorption onset (2 × *E*_f–f_) estimated from the PL spectra, *i.e.*, the anticipated energy threshold for quantum cutting in these materials below which energy conservation cannot be maintained. (D) Data from a second experiment like panel C, plotted as the quantum-cutting energy efficiency (QCEE) *vsE*_abs_ (black circles). The black curve plots the idealized QCEE for band-gap-optimized Yb^3+^:CsPb(Cl_1–*x*_Br_*x*_)_3_ NCs (*x* ∼ 0.75, solid black curve). These NCs had a measured PLQY approaching 200%. For comparison, the energy-conversion efficiency of a typical c-Si photovoltaic cell (dashed black), the AM1.5 solar spectral irradiance (gray), and the absorption (blue) and PL spectra (red) of the Yb^3+^:CsPb(Cl_1–*x*_Br_*x*_)_3_ NCs are also plotted. Reprinted from ref (659). Copyright 2019 American Chemical Society.

A second important observation came from experiments using post-synthetic anion-exchange chemistries to tune the band gap of Yb^3+^-doped perovskite NCs.^659^Figure 70 summarizes the results of one set of measurements that began with Yb^3+^-doped CsPbCl_3_ NCs. Figure 70a shows that substochiometric titrations of the reactive bromide precursor TMSBr narrowed the perovskite band gap, ultimately reaching ∼515 nm at complete anion exchange to form Yb^3+^-doped CsPbBr_3_. Figure 70b plots the excitonic and Yb^3+^ PL spectra for each CsPb(Cl_1–*x*_Br_*x*_)_3_ composition within this series, and Figure 70c summarizes these results by plotting the Yb^3+^ PL intensity *versus* the exciton PL wavelength. These data show that the Yb^3+^ PL intensity remains essentially constant with added bromide until *E*_g_ reaches approximately 2 times the f–f energy (gray bar in Figure 70c), at which point the PL drops precipitously. Further experiments showed that the PL recovered upon reverse anion exchange, following much the same trajectory.^659^ These results verify the origin of this Yb^3+^ PL as coming from a 2-for-1 quantum-cutting process. Moreover, these results demonstrate an extremely high quantum-cutting *energy efficiency* (QCEE) of the sensitized PL process, quantified as . Figure 70d plots data from another experiment like that in Figure 70c, but now representing the data as the QCEE *versus* absorption threshold energy. This representation shows that experimental QCEEs exceeding 90% can be obtained, *i.e.*, only a very small portion of the energy from the absorbed photon is lost as heat, whereas the vast majority is re-emitted in the near-infrared. This value can be contrasted with the ∼25% energy efficiency of a high-efficiency silicon heterojunction solar cell converting the same blue photon (dashed line in Figure 70d). These results have major significance for potential applications of these materials as spectral conversion layers in photovoltaics; in addition to demonstrating optimization of the band gap for minimal thermalization loss, these results show that the emitted light is well-matched to the absorption onset of red-sensitive Si photovoltaics (Figure 70d).

To further exploit the quantum-cutting properties, the Song group has developed a series of bi- and tridoped lead-halide perovskite NCs incorporating additional amounts of Pr^3+^ and Ce^3+^, by analogy to more traditional quantum-cutting compositions.^647,661^ Codoping is achieved by hot injection with subsequent anion exchange using PbX_2_ to tune the energy gap. Pr^3+^ and Ce^3+^ both possess excited states at energies close to the perovskite energy gap, and time-resolved PL measurements showed participation of these ions, which dramatically slowed the arrival time of the energy in the Yb^3+^ ions, as detected by time-resolved PL.^661^ Maximum PLQYs of 173% were reported for the optimized Yb^3+^/Pr^3+^/Ce^3+^ tridoped CsPb(Cl_0.33_Br_0.66_)_3_ NCs. These codoped materials may help to minimize the importance of uncontrolled traps as intermediate states in the quantum-cutting process by instead routing energy through well-defined and well-controlled Ln^3+^ intermediate states.

**Figure 71:**
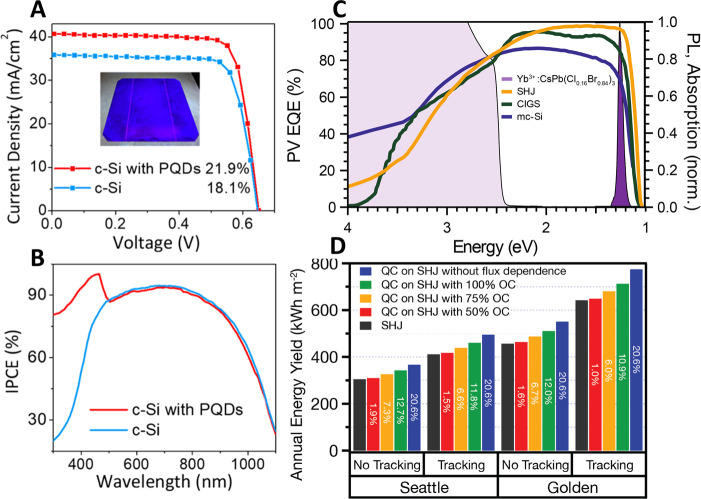
(A) *J*–*V* curves of a single-crystal silicon solar cell with and without a coating of Yb^3+^(6%)–Pr^3+^(4%)–Ce^3+^(3%)-tridoped CsPb(Cl_0.33_Br_0.67_)_3_ NCs, showing an increase in power-conversion efficiency from 18.1 to 21.9% upon addition of the NCs. (B) IPCE (EQE) curves of a single-crystal silicon solar cell with and without a coating of Yb^3+^(6%)–Pr^3+^(4%)–Ce^3+^(3%)-tridoped CsPb(Cl_0.33_Br_0.67_)_3_ NCs, showing enhancement at short wavelengths where the NCs absorb. (C) EQE characteristics of Si heterojunction (SHJ), CIGS, and multicrystalline Si photovoltaics and the absorption and near-infrared (∼1.26 eV) emission of Yb^3+^:CsPb(Cl_0.16_Br_0.84_)_3_ quantum cutters, showing the excellent match of the quantum-cutter absorption and photoluminescence with the solar cell response curves, particularly for red-sensitive SHJ. (D) Areal annual energy production yield of a Yb^3+^:CsPb(Cl_1–*x*_Br_*x*_)_3_/SHJ QC/PV device with and without 2-axis tracking mechanisms and for different efficiencies of optical coupling, including the effects of flux-dependent PLQY. Relative percentage increases are labeled on each bar. Results are presented for two geographic locations in the United States: Seattle, WA, and Golden, CO. Panels A and B are reprinted from ref (661). Copyright 2019 American Chemical Society. Panels C and D are reprinted with permission from ref (657). Copyright 2019 Royal Society of Chemistry.

The Song group has made substantial progress in integrating Yb^3+^-doped CsPb(Cl_1–*x*_Br_*x*_)_3_ NCs with both Si and CIGS photovoltaics.^647,661^ Impressive gains in power conversion efficiencies have been achieved simply by modifying the front surfaces of the PVs with doped perovskite NC spectral conversion layers through a solution coating method. Layer thicknesses of ∼230 nm were found to allow the NCs to absorb most super-band-gap photons and downshift their energy *via* quantum cutting to ∼990 nm Yb^3+^ emission, without introducing too much light scattering at sub-band-gap wavelengths that would interfere with transmission of those wavelengths to the underlying photovoltaic cell (so that lanthanide emission can be absorbed by the cell). Figure 71A shows experimental *J–V* data^661^ collected for a crystalline (c) Si PV before and after coating with quantum-cutting NCs. An absolute increase of 3.1% (>20% rel.) is observed in the power-conversion efficiency of this cell. Confirmation that this increase results from spectral downshifting comes from the action spectrum of Figure 71B,^661^ which shows little change throughout the spectral response until the perovskite band gap is reached, at which point the incident power conversion efficiency increases sharply. These results demonstrate the promise of these materials for making major improvements to photovoltaic efficiencies. The Gamelin group has performed detailed balance calculations to assess the maximum thermodynamic efficiency increases that can be anticipated from various photovoltaics types by taking advantage of this quantum cutting, using the real spectroscopic characteristics of these materials.^657^Figure 71C shows the spectral characteristics of the narrowest gap Yb^3+^:CsPb(Cl_1–*x*_Br_*x*_)_3_ composition for which high-efficiency quantum cutting is feasible, in comparison with the external quantum efficiency curves of multicrystalline Si, CIGS, and silicon heterojunction (SHJ) cells. SHJ technology is very nearly optimal for pairing with these quantum cutters because of its better red sensitivity. The calculations considered various known loss processes, including power saturation^658^ of the quantum-cutting luminescence and incomplete capture of emitted photons by the underlying cell, to project annual energy yields for different implementations. Figure 71D summarizes these calculations, showing that under all circumstances, sizable increases are anticipated. For example, a relative increase of 7.3% is anticipated in the case where the PLQY = 200%, photon capture = 75%, and the real saturation response is included. These experimental and computational results indicate that substantial progress toward exceeding the Shockley–Queisser single-junction efficiency limit can be anticipated from this technology pending engineering advances.

A second approach to harnessing the energy efficiency of these quantum-cutting NCs in solar technologies is to integrate them into LSCs. Doped NC LSCs were initially introduced using Mn^2+^-doped ZnSe as the active material, absorbing short-wavelength solar photons and emitting from the internal Mn^2+^ d–d excited state.^671^ This work demonstrated that doped nanocrystals excel at separating the tasks of photon absorption and photon emission, yielding the lowest reabsorption losses of any spectral downshifter investigated to date.^672^ Mn^2+^ emission occurs higher in energy than desired for this technology, however, and other copper-containing luminescent NCs (*e.g.*, Cu^+^-doped quantum dots or CuInS_2_) have the best overall solar conversion efficiencies.^672−676^

**Figure 72:**
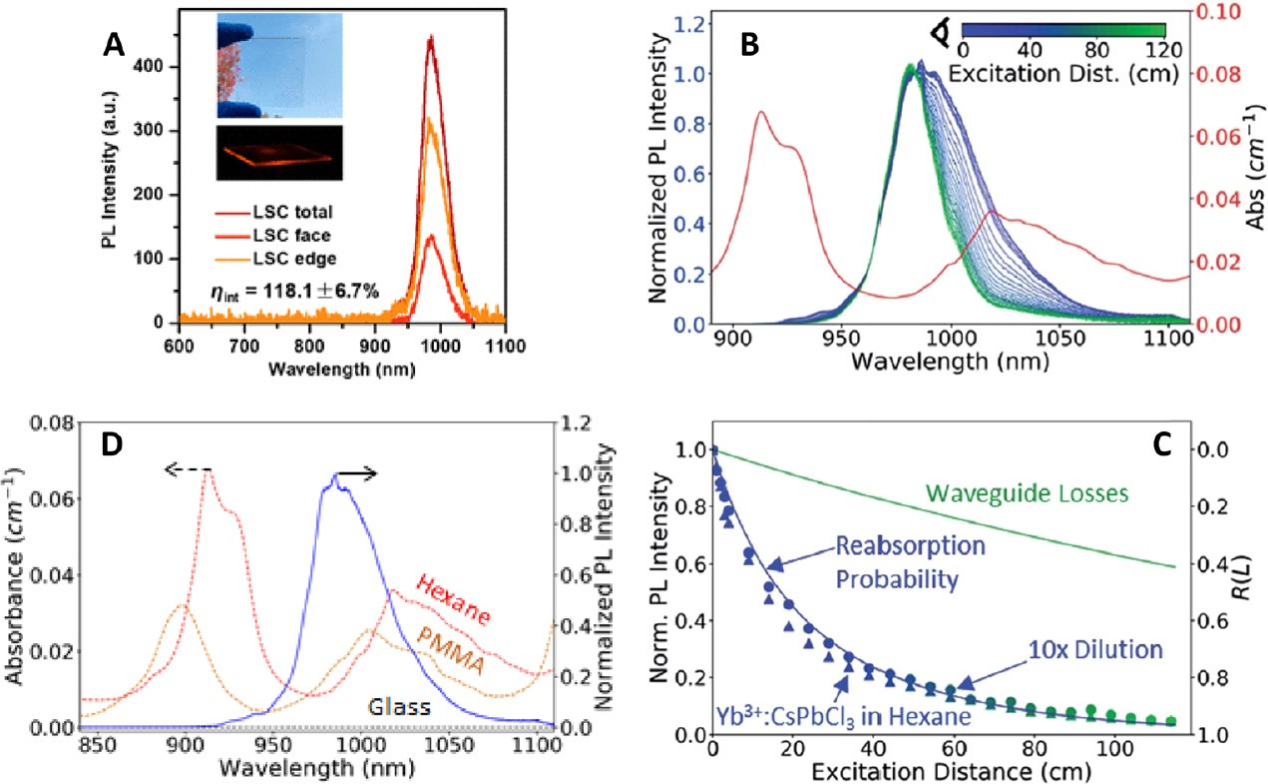
(A) Total (dark red), facial (light red), and edge (orange) emission spectra measured from a 5 cm^–1^ × 5 cm^–1^ LSC made using Yb^3+^-doped CsPbCl_3_ NCs. The internal optical efficiency (edge-emitted photons/absorbed solar photons) of this device was measured to be 118 ± 7%. The top inset shows the high transparency of the LSC to visible light, and the bottom inset shows the LSC’s edge emission under UV illumination, collected with a 570 nm long-pass filter. The PLQY of these NCs was measured to be 164 ± 7% prior to incorporation into the LSC. The optical density of this LSC at the perovskite absorption edge is 0.2. (B) Normalized PL spectra of Yb^3+^:CsPbCl_3_ NCs suspended in hexane with o.d. ∼ 0.75 at 375 nm, obtained from a liquid 1D LSC experiment at various excitation distances relative to the edge-mounted photodetector (inset). The red curve shows the absorption spectrum of the hexane solvent. (C) Integrated normalized Yb^3+^:CsPbCl_3_ NC PL intensity plotted as a function of excitation distance away from the photodetector for NCs in hexane with o.d. ∼ 0.75 (triangles) and o.d. ∼ 0.075 (circles) at 375 nm. The blue trace is the reabsorption probability predicted from a model. The green line is the experimental performance limit of the 1D LSC waveguide itself. All PL data were collected with excitation at 375 nm, and all data were collected at room temperature. (D) Absorption spectrum of hexane (red), a representative PMMA sample (orange), and Schott optical-quality glass (black) overlaid with the normalized PL spectrum of Yb^3+^:CsPbCl_3_ NCs (blue). Panel A is adapted from ref (655). Copyright 2018 American Chemical Society. Panels B–D are adapted with permission from ref (656). Copyright 2019 Royal Society of Chemistry.

Two studies have investigated quantum-cutting Yb^3+^:CsPb(Cl_1–*x*_Br_*x*_)_3_ NCs in LSCs. The Wu group has incorporated Yb^3+^:CsPbCl_3_ NCs into 5 cm × 5 cm acrylic waveguides (Figure 72A) and reported an internal optical efficiency (edge-emitted photons/absorbed solar photons) of 118% for Yb^3+^:CsPbCl_3_ NCs in PMMA, extrapolating to estimate the performance of large-area devices.^655^ The optical density of these devices was rather small (0.2 at the absorption edge), possibly because of solubility limitations within the PMMA matrix. Moreover, the band gap of Yb^3+^:CsPbCl_3_ NCs is large, limiting absorption to only ∼3% of the solar flux at AM1.5. The external power conversion efficiency of the 5 cm × 5 cm device was thus only 3.7%. Nonetheless, the power of quantum cutting and lanthanide emission is evident, resulting in high PLQYs (∼164% for these NCs) and very low reabsorption of the emitted light by the same lanthanide f–f transitions.

A key objective of LSCs is to concentrate photons harvested over large LSC facial areas onto small PV areas. It is therefore critical to evaluate photon losses in large-scale waveguides, for example on the scale of a building’s window, because many important loss mechanisms that do not appear detrimental in short waveguides turn out to be problematic over larger distances. To this end, the Gamelin group measured waveguiding within a 120 cm 1D LSC and found that Yb^3+^:CsPbCl_3_ NC have negligible intrinsic attenuation losses over these very large waveguide lengths, as expected from their strongly downshifted emission and the very small extinction coefficients of the f–f transitions, but they also found severe attenuation of the f–f emission when the waveguide contained C–H bonds (high-frequency vibrations).^656^ This result has very important implications for any LSC work involving Yb^3+^, because it precludes the use of popular acrylics as the waveguide medium. This group demonstrated that the problem could be reduced or removed by eliminating C–H vibrations within the waveguide medium. Implementation of this strategy in a 2D LSC will require additional waveguide innovations.

Beyond conventional 2D LSCs, Gamelin’s group further proposed and modeled a “monolithic-bilayer” LSC architecture that integrates quantum-cutting NCs with conventional LSC chromophores in vertical series within the same waveguide.^656^ This architecture offers similar advantages of tandem LSCs, but in a much simpler configuration. Modeling predicted that a monolithic bilayer configuration could improve the performance of state-of-the-art CuInS_2_ LSCs by at least 19%, for example. Instead of summing voltages from the two layers of a tandem LSC, the bilayer device sums the currents from each layer at the same voltage, allowing use of only a single PV rather than two PVs with separate band gaps. The bilayer approach also avoids the challenge of current matching in tandem LSCs. Experimental demonstration of the device will require C–H-free waveguides, as discussed above.

**Figure 73:**
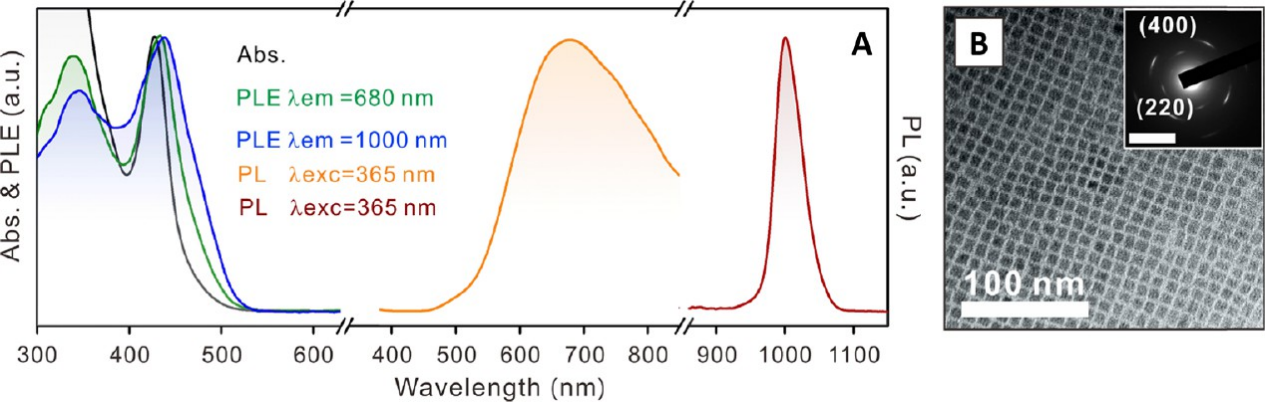
(A) Absorption, PL, and PLE spectra of 2.9% Yb^3+^-doped Cs_2_AgBiBr_6_ NCs. (B) TEM image of the Yb^3+^-doped Cs_2_AgBiBr_6_ NCs. Inset: Selected area electron diffraction pattern for the same NCs (scale bar = 2 nm^–1^). Adapted from ref (562). Copyright 2019 American Chemical Society.

In related materials, lanthanide doping of lead-free metal-halide elpasolite (so-called “double perovskite”) NCs have yielded promising results that may point the way to convert these materials, which generally show strong absorption but poor luminescence, into useful luminescent materials. Three publications exploring Ln^3+^ doping of colloidal Cs_2_AgInCl_6_ NCs appeared within a few months of one another.^562,565,568^ The Kim group synthesized colloidal Cs_2_AgInCl_6_ NCs doped with Yb^3+^, Er^3+^, or both simultaneously, and they demonstrated that f–f emission from these lanthanides can be generated by photoexcitation of the host NCs.^565^ The PLQYs in these NCs were noted to be over an order of magnitude smaller than those of the Yb^3+^:CsPb(Cl_1–*x*_Br_*x*_)_3_ NCs, and the PLE spectra curiously did not reflect the absorption features of the materials. In parallel, the Chen group studied Yb^3+^ doping of Cs_2_AgBiCl_6_ and Cs_2_AgBiBr_6_ NCs, showing that both lattices can be used to host Yb^3+^ ions and sensitize their f–f luminescence.^562^Figure 73 summarizes some key results from this study, showing the observation of both Yb^3+^ and broad trap luminescence with UV photoexcitation of the Cs_2_AgBiBr_6_ NCs when doped with a few % Yb^3+^. The PLE spectra track the absorption spectra, demonstrating conclusively the key result of Yb^3+^ sensitization by the Cs_2_AgInBr_6_ NC host. The Nag group also examined Yb^3+^ doping of colloidal Cs_2_AgInCl_6_ NCs.^568^ Their results highlighted that the sensitized Yb^3+^ PL is much stronger than the weak, broad luminescence of the undoped NCs, and that the latter gets even weaker upon introduction of Yb^3+^. These observations show that Yb^3+^ competes with both nonradiative recombination and trapping for the energy of the absorbed photon. Although the PLQYs of all of these elpasolites were small (<10%), further synthetic advances with elpasolite NCs may help to boost this value by suppressing nonradiative decay in these materials. Notably, however, Yb^3+^ doping into Cs_2_AgInCl_6_ and other elpasolite lattices can be achieved by isovalent substitution, meaning that it occurs without formation of the same kind of closely associated charge-compensating defect hypothesized to play a role in the quantum-cutting mechanism of the Yb^3+^:CsPb(Cl_1–*x*_Br_*x*_)_3_ NCs. It is unclear whether such a defect level is actually necessary or merely incidental in those quantum-cutting compositions, and further development of luminescent Yb^3+^-doped elpasolite NCs could help to address this question. If quantum yields comparable to those found in Yb^3+^:CsPb(Cl_1–*x*_Br_*x*_)_3_ NCs can ultimately be achieved in double perovskites too, their lead-free compositions would be very attractive for large-scale solar applications.

## Self-Assembly

### Self-Assembly of Nanocubes

Over the last few decades, self-assembly of colloidal nano- and microparticles into long-range ordered superlattices (SLs) has been widely investigated on various material systems.^660,677−680^ Similar to atoms in crystals where the lattice defines the physical properties of the bulk compound, the NCs in the SL could eventually determine additional collective properties of the solid. This is a crucial step for the integration of the colloidal nanostructures into devices. Uniform NCs can assemble into 1D, 2D, and 3D architectures through the single component or through binary (or even ternary) self-assembly of larger and smaller particles.^677^ The forces that drive NC self-assembly range from hard- to soft-particle interactions. Taking advantage of previous knowledge gained on the self-assembly of conventional monodisperse colloidal NCs, a large variety of self-assembly techniques have been reported over the years such as evaporation-driven or destabilization-driven approaches, as well as spontaneous and template-assisted self-assembly.^660,677,679^ Recently, these techniques have been extended to self-assembly of halide perovskite NCs into highly ordered SLs for exploration of their collective properties that can be very different from their individual constituents.^21,80,81,111,143,160,322,681−689^ Near monodispersity of NCs and high shape uniformity are important factors to obtain long-range ordered NC SLs.^660,690,691^ Fortunately, these conditions are easily met for all-inorganic CsPbX_3_ perovskite NCs as they are often prepared with near monodispersity regardless of the synthesis method, as discussed in the synthesis section.^14,30,53,134,143^ As a result, these perovskite nanocubes tend to self-assemble into 1D or 2D SLs on a TEM grid upon solvent evaporation from a droplet of high concentrated colloidal solution. Initial examples of CsPbBr_3_ nanocube SLs date back to 2017, when 2D and 3D assemblies were obtained by the solvent evaporation method.^21,111,322^ In reference (322) small superlattice domains on TEM grids exhibit a simple cubic packing of the nanocubes with a lattice constant of ≈12.5 nm and an interparticle separation of ≈2.3 nm. The SLs show a red shift of 15 nm compared to individual NCs. Upon applying high pressure, the NCs in the SLs fuse together and the corresponding SLs transform into single-crystalline nanoplatelets. In refs (21 and 111) much larger 3D aggregates were obtained on silicon substrates which also exhibited a red-shifted PL peak at room temperature.

**Figure 74:**
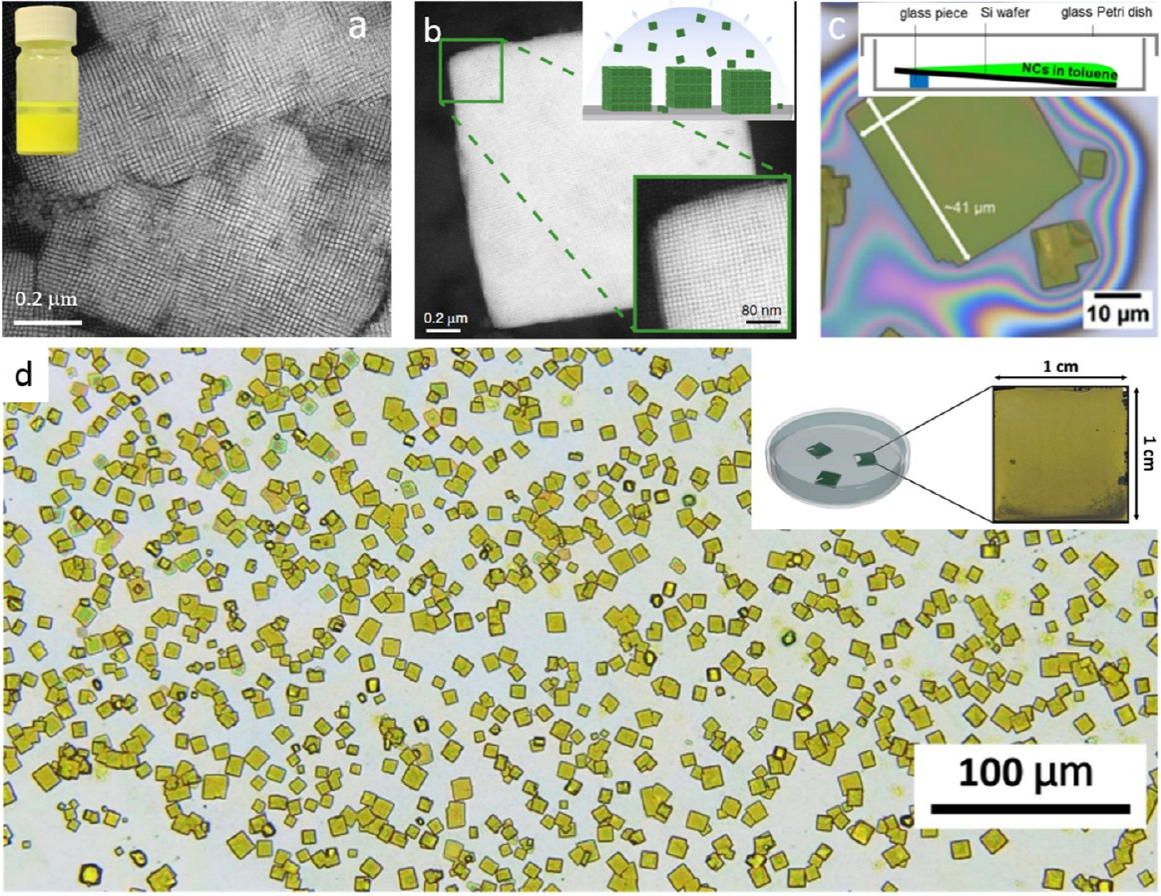
HAADF-STEM image of CsPbBr_3_ nanocube SLs obtained by spontaneous self-assembly in solution (a) and by solvent evaporation (b). Panel a is reprinted with permission from ref (80). Copyright 2018 John Wiley & Sons, Inc. Panel b is reprinted with permission from ref (81). Copyright 2018 Nature Publishing Group. (c) Optical microscopy images of large (50 μm) CsPbBr_3_ nanocube SLs prepared on top of a tilted Si wafer. Reproduced from ref (143). Copyright 2018 American Chemical Society. (d) Large-area, nearly uniform CsPbBr_3_ nanocube SLs prepared on a Si substrate by solvent drying in a closed Petri dish, and the inset illustrates the experimental for self-assembly on a Si substarte in a Petri dish (left panel) and an optical microscopy image of a Si substrate (right panel) covered with densely packed SLs. Reproduced from ref (692). Copyright 2019 American Chemical Society.

One of the interesting features of CsPbBr_3_ nanocubes is that they spontaneously self-assemble into SLs in a sufficiently concentrated colloidal solution, as shown by Tong *et al.* (Figure 74a).^80^ The red-shifted PL from SLs makes them better suited as pure-green emitters (ideal wavelength of ∼530 nm), whereas individual nanocubes emit below 518 nm. In ref (80) the origin of the red shift was attributed to the mini-band formation caused by the electronic coupling of nanocube subunits in SLs. Interestingly, the colloidal SLs partially preserve their supercrystal morphology even after halide exchange reaction, and thus their optical properties are easily tunable across the visible wavelength range. However, spontaneous self-assembly comes with less control over the morphology and size of the produced SLs. On the other hand, solvent drying techniques produce large area well-defined square-shaped 3D CsPbBr_3_ SLs, as initially demonstrated by Kovalenko *et al.* (Figure 74b).^81,111^ Interestingly, these SLs generate short, intense bursts of light so-called superfluorescence - upon light excitation due to coherent and cooperative emission of nanocubes in the SLs.^81^ The peak position of superfluorescence red-shifted with more than 20-fold accelerated radiative decay as compared to uncoupled nanocubes. Recently, a similar phenomenon has been reported in CsPbBr_3_ SLs by Xie and co-workers.^693^ They claimed that the stimulated emission of nanocube assemblies in SLs is not limited by the traditional population-inversion condition. However, the SLs reported in that work are not well-defined regarding their morphology and the yield appears to be low based on the given electron microscopy images.

Several attempts have been made to optimize the solvent drying technique to achieve large area cubic SLs with high yield.^143^ One of the critical factors for obtaining SLs is the size distribution and shape purity of the corresponding perovskite NC building blocks. In this regard, Imran and co-workers^143^ showed the fabrication of large cubic or rectangular 3D SLs (∼50 μm lateral size) in very high yield using the shape-pure and nearly monodisperse CsPbBr_3_ nanocubes prepared using secondary aliphatic amines (Figure 74c). Such large size SLs have been accomplished by evaporation of solvent from a colloidal solution on top of a tilted Si wafer either inside a glovebox or at ambient conditions (inset of Figure 74c). Furthermore, large area, nearly uniform CsPbBr_3_ NC SLs were prepared by slow solvent evaporation on a Si substrate placed in a closed petri dish (Figure 74d), and the structural coherences of these SLs were revealed by SL reflection peaks in wide-angle X-ray diffraction measurements.^692^ These are fingerprint peaks to long-range order and high crystallinity of nanocubes and the angular separation if these peaks are very sensitive to the periodicity of SL. It is very important to consider that the NCs of SLs can coalesce into larger structures, and this can significantly affect their PL properties by energy-transfer process.^83^ Despite significant progress toward the fabrication of high-quality CsPbBr_3_ nanocube 3D SLs, only a few studies have been published on the preparation of 2D and 1D SLs.^681,683,694^ Very recently, Patra *et al.* demonstrated the preparation of ultrasmooth self-assembled monolayers using CsPbBr_3_ nanocubes functionalized with short-chain thiocyanate ligands (SCN^–^).^683^

**Figure 75:**
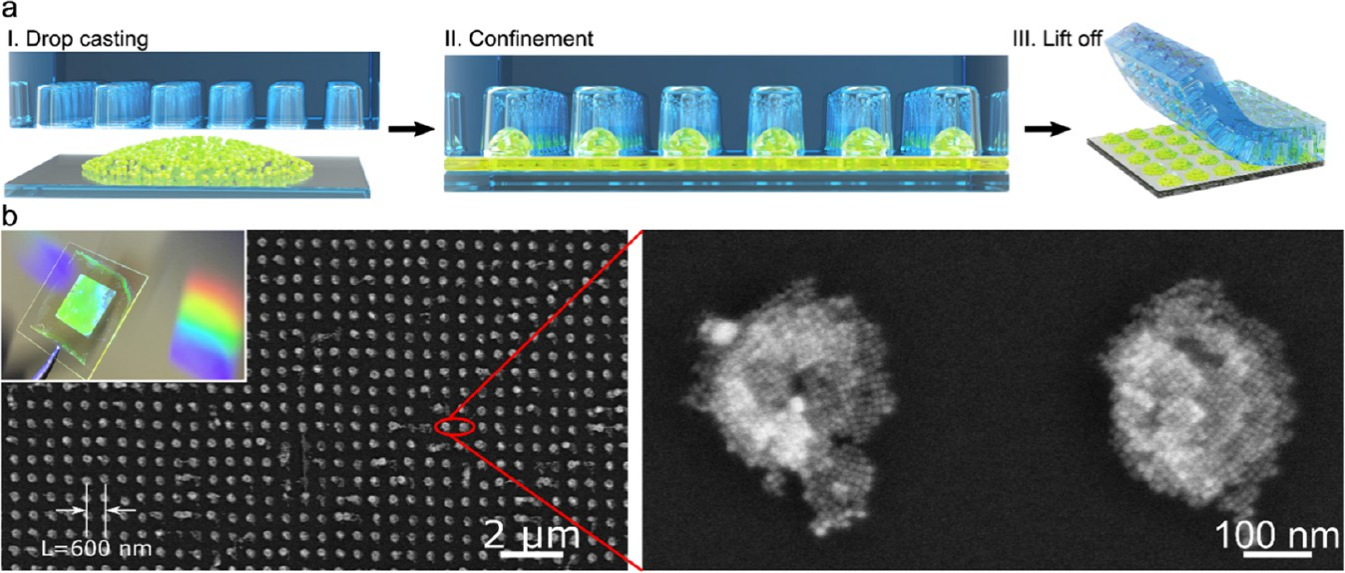
(a) Schematic illustration of PDMS template assisted self-assembly CsPbBr_3_ nanocubes into 2D photonic SLs. (b) Corresponding SEM images of 2D photonic CsPbBr_3_ SLs with lattice parameters of 600 nm (inset: photograph of the CsPbBr_3_ SL arrays on glass substrates under white light illumination). Reproduced with permission under a Creative Commons CC-BY License from ref (688). Copyright 2020 John Wiley & Sons, Inc.

Device applications of SLs will most likely require control over their dimensionality and positioning on a given solid substrate. However, it is extremely difficult to fulfill these conditions using the self-assembly techniques discussed above. Alternatively, template-assisted self-assembly has been gaining significant attention to achieve these conditions.^685,695,696^ However, the packing of perovskite NCs in the assemblies patterned by this approach has yet to be investigated in detail. Very recently, David *et al.*(688) reported the fabrication of 2D perovskite photonic SLs using prepatterned PDMS templates. The height and lateral dimensions of the SLs were controllable by the predesigned PDMS template (Figure 75a,b). These photonic crystals exhibit field enhancement at NIR excitation by a light trapping mechanism. However, such self-assemblies are not perfect as the SLs obtained by the slow solvent evaporation approach (Figure 75b). Therefore, there is still plenty of room for the optimization of perovskite SLs obtained by the template-assisted assembly. Despite rapid developments in the field of perovskite NCs, there is still a lack of knowledge on the various NC assemblies such as free-standing SLs, binary and ternary SLs.

### Self-Assembly of Anisotropic LHP NCs

**Figure 76:**
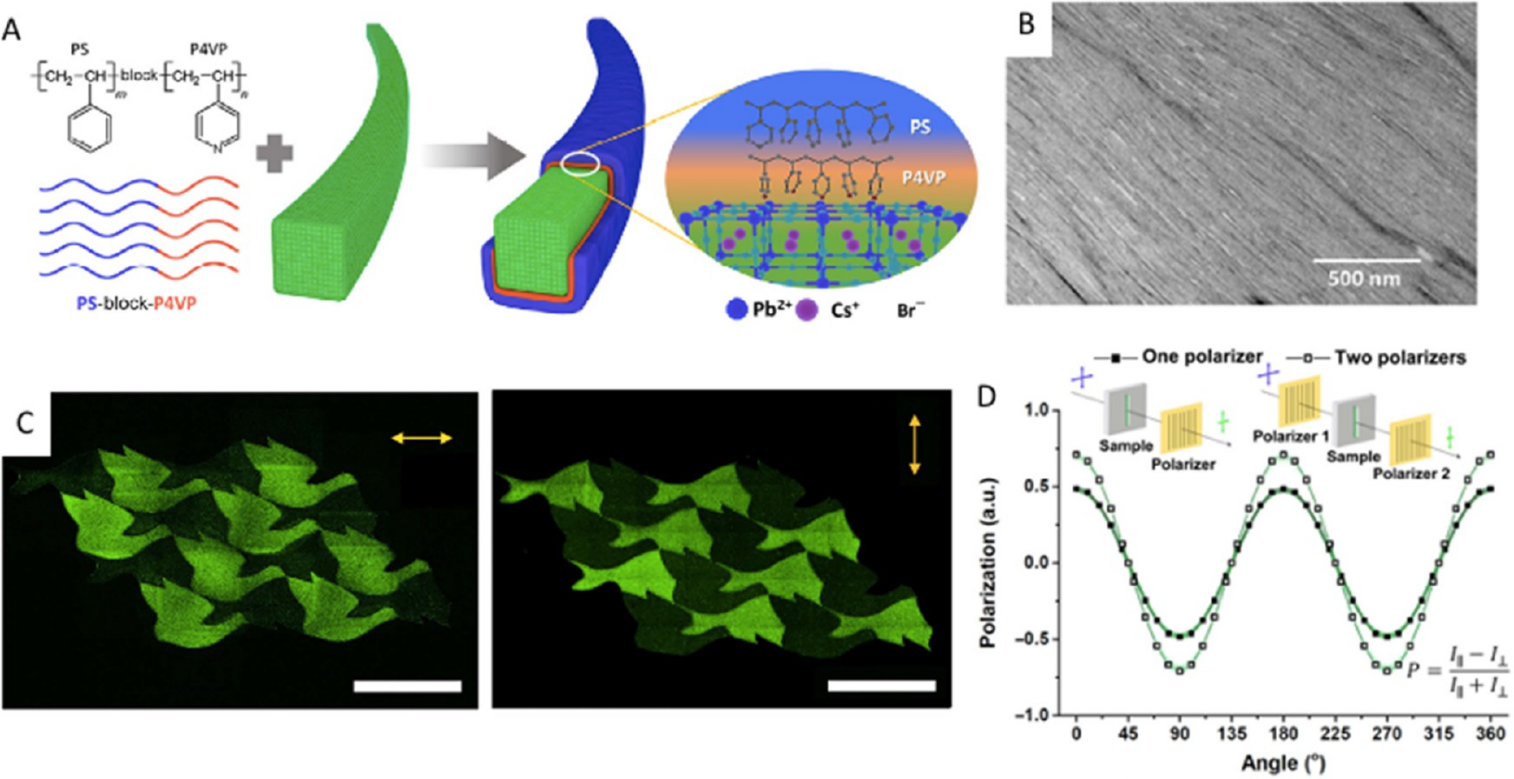
(A) Schematic illustration of synthesis of block-copolymer-coated CsPbBr_3_ NWs with core–shell configurations. (B) Self-assembled NW monolayer using the Langmuir–Blodgett method. Reproduced with permission from ref (697). Copyright 2020 Springer Nature. (C) Polarized emission from printed polymer–CsPbBr_3_ NW composite with horizontal polarization (left) and vertical polarization (right). Scale bars, 1 mm. (D) Polarized emission from printed perovskite NW composites as a function of different angles. Reproduced with permission under a Creative Commons CC BY-NC license from ref (699). Copyright 2019 The Authors.

Self-assembly of other shapes including nanorods,^686^ nanowires,^22,697^ and nanoplatelets^682^ has also been reported. For instance, Yang and co-workers^682^ reported the self-assembly of 2D perovskite nanosheets by a layer-by-layer approach. Interestingly, the 2D perovskite nanosheets SLs resemble Ruddlesden–Popper layered perovskite phase. This self-assembly process is reversible as the SLs transform into individual building blocks upon sonication. One-dimensional (1D) NWs show potential anisotropic optoelectronic properties when they are highly oriented. It has been shown that oriented self-assemblies of perovskite NWs were obtained at the air-liquid interface by Langmuir-Blodgett technique.^22,697^ However, the ionic nature of halide perovskites limits their stability at air-water assembly interface. To realize the assembly of perovskite NWs with better stability against water, a core–shell-type configuration has been introduced using the amphiphilic block copolymer such as polystyrene-*block*-poly(4-vinylpyridine) (PS-P4VP) (Figure 76A,B).^697^ The shelling polymer materials can not only prevent the NW bundling, ensuring a better solution dispersion, but also improve the stability of NWs against water, due to the blocking effect of hydrophobic polystyrene. For perovskite NWs, the PLQY typically shows significant reduction due to their large surface to volume ratio comparing with NCs, the polymer coating represents an effective strategy for the enhancement of their absolute quantum efficiency due to the passivation effect. With a modified Langmuir–Blodgett technique, the polymer-coated perovskite NWs were able to assemble into a uniform monolayer with the uniaxial alignment at the air-liquid interface. The anisotropic polarized PL emission was detected at different angles from the oriented nanowire monolayers.

In addition to the conventional patterning method, a direct ink writing technique has been developed using the aligned cellulose fibrils embedded into a hydrogel matrix.^698^ This method can control the anisotropic alignment of nanocomposite with 3D architectures. The polymer-coated perovskite NW bundles were used as a printing nanocomposite ink.^699^ It is possible to control the orientation of polymer-perovskite NW nanocomposites through the 3D printing technique, which influences their polarized PL emission (Figure 76C,D). The polarization anisotropy in 3D-printed perovskite NW composite could be promising for optical device applications.

## Morphological and Structural Characterization

As discussed in previous sections, the morphology and crystal structure of perovskite NCs play an important role in their optical properties. This section is focused on the morphological and structural characterization of perovskite NCs using electron microscopy and X-ray scattering techniques, respectively. LHPs are very sensitive to electron beam illumination and they often tend to degrade into metallic Pb. In particular, it is extremely difficult to obtain high-resolution electron microscopy images. Therefore, electron microscopy images of perovskite NCs have to be acquired with extreme care. We discuss the current challenges and recent advances in electron microscopy studies on various kinds of perovskite NCs. On the other hand, various X-ray scattering techniques have been used for the structural characterization of perovskite NCs and their assemblies. We discuss the application of various X-ray scattering techniques on PeNCs, ranging from common XRD measurements to advanced synchrotron-based *in situ* measurements with 2D detectors. In particular, studies about phase-stability and degradation are discussed. In addition, we discuss X-ray scattering studies used to investigate structure–function correlations.

### Electron Microscopy

Aberration-corrected (scanning) transmission electron microscopy ((S)TEM) has a become a standard technique to investigate nanomaterials at the atomic level. With the development of *C*_*s*_ (spherical aberration) and *C*_*c*_ (chromatic aberration) corrected microscopes, it has become feasible to obtain structural information at the atomic scale, even using low acceleration voltages. Such investigations allow us to correlate the (atomic) structure of nanomaterials with their chemical and physical properties. The acquisition of atomically resolved (S)TEM images of halide perovskites using conventional electron dose rates is however hindered by their sensitivity to the energetic electron beam. Upon illumination, structural damage and/or phase transitions could occur, which hampers a visualization/characterization of the initial (crystal) structure of the halide perovskite NCs. Therefore, electron microscopy studies of halide perovskite NCs have to be performed with extreme care.

#### Degradation of LHP NCs under the Electron Beam

**Figure 77:**
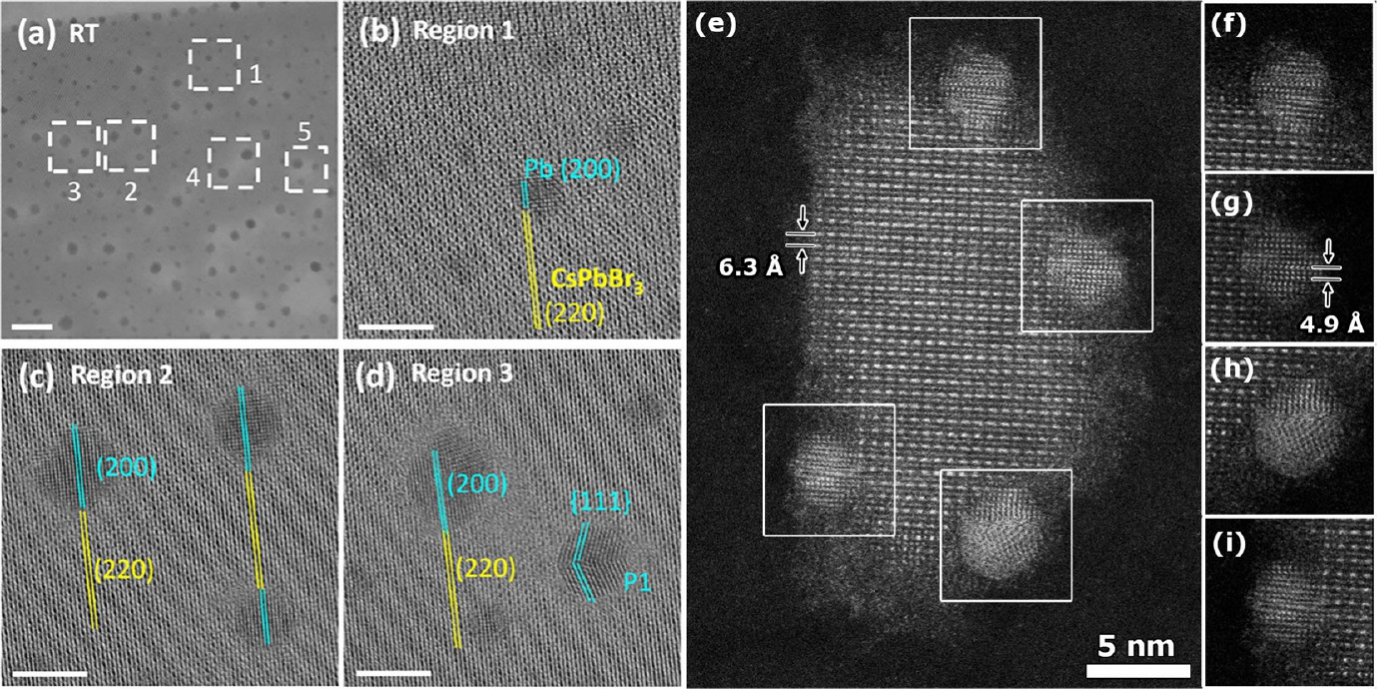
(a–d) High-resolution TEM analysis of the formation of metallic Pb particles on a 3 nm thick CsPbBr_3_ nanosheet at room temperature: (a) overview image (scale bar: 20 nm) and (b–d) high-resolution TEM images of three regions of interest in (a), which show the presence of Pb particles (scale bars: 5 nm). Reproduced from ref (139). Copyright 2017 American Chemical Society. (e–i) High-resolution HAADF-STEM image of a CsPbI_3_ NC showing the presence of bright spherical particles mainly at the edge of the nanoparticle (f–i). These particles were identified as metallic Pb. Reproduced with permission from ref (30). Copyright 2016 John Wiley & Sons, Inc.

Illuminating halide perovskite NCs with an energetic electron beam results in the rapid formation of high contrast particles, hampering the acquisition of an image at both nano and atomic scale of halide perovskite NCs. Such behavior has been reported in multiple studies using either a parallel beam in TEM mode (Figure 77a–d) or a focused electron probe in STEM (Figure 77e–i).^16,30,48,74,139,700,701^ Yu *et al.* performed comparative studies on lead-halide perovskite nanostructures at both low and high accelerating voltages in both TEM and STEM mode, which showed clear, rapid formation of high contrast particles in all cases.^702^ Different claims have been made about the nature of these nanometer-sized nanoparticles and the resulting structural deformations in the perovskite NC.^16,30,48,702^ Recently, Dang *et al.* demonstrated that these particles consist of metallic lead and that their nucleation mainly results from a radiolysis process.^139^ It was shown that at both low and high irradiation voltages desorption of halogen atoms from the surface of the perovskites and reduction of Pb^2+^ ions to Pb^0^ were induced by the interaction with the electron beam. Subsequently, neighboring Pb^0^ atoms diffused and aggregated into nanometer-sized, spherical Pb particles. The formation of such metallic lead nanoparticles preferentially occurs at the edges and corners of the perovskite NCs. Halide perovskite NCs with a high surface area to volume ratio, such as thin nanowires and nanoplatelets, are therefore more susceptible to such electron beam induced damage.^139^ Next to the formation of metallic lead particles, degradation and loss of crystallinity at the edges and/or corners of halide perovskite NCs are additional challenges when investigating (thin) halide perovskite NCs. Both phenomena can be observed in movie S2 (degradation of a CsPbBr_3_ nanocube upon continuous scanning of the electron beam) and Figure S1 (a few selected high-resolution HAADF-STEM frames of movie S2), where the degradation of a single CsPbBr_3_ nanocube is observed. This complicates the investigation of the surface termination of halide perovskite NCs since such degradation is (extremely) rapid depending on the thickness of the nanomaterial.

#### Acquisition of Atomically Resolved Images

##### All-Inorganic Halide Perovskite NCs

**Figure 78:**
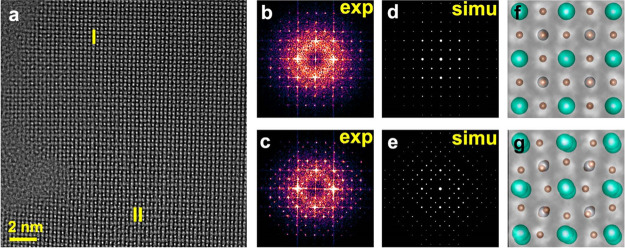
Aberration-corrected high-resolution TEM performed on CsPbBr_3_ showing the coexistence of the cubic and the orthorhombic phases. (b,c) Fourier transforms from regions I (b) and II (c), which are highlighted in image (a). (d,e) Simulated electron diffraction patterns of the cubic (d) and the orthorhombic (e) CsPbBr_3_ phase. (f,g) Enlarged images from regions I (f) and II (g). The cubic and orthorhombic structure models are overlaid on (f) and (g), respectively. Reproduced from ref (702). Copyright 2016 American Chemical Society.

To overcome electron beam-induced sample degradation, aberration-corrected high resolution TEM,^703−705^ low-dose in-line holography,^702^ and dose-controlled aberration-corrected STEM imaging^22,30,73,702,706,707^ have been successfully applied to study all-inorganic lead-halide perovskite nanomaterials at the atomic level. Yu *et al.* initially visualized the pristine structure of ultrathin two-dimensional CsPbBr_3_ perovskites by applying low-dose in-line holography.^702^ Using this low-dose technique, a series of aberration-corrected high-resolution TEM images were acquired and the phase information was extracted by reconstructing the image series. The atomic structure of these two-dimensional CsPbBr_3_ perovskites was successfully studied before any electron beam-induced sample alterations had occurred. This study revealed the coexistence of the high-temperature cubic and the low-temperature orthorhombic phases in such CsPbBr_3_ nanosheets. It must be pointed out that the two phases have a close structural similarity, where only a small tilting of the PbBr_6_ octahedra is necessary to transform from the cubic phase into the orthorhombic phase. To distinguish between these two phases, high-quality data with an optimal resolution are required. In addition, they also successfully acquired single dose-controlled aberration-corrected high resolution TEM images using a negative *C*_*s*_ which revealed this two-phase coexistence (Figure 78a). The spatial resolution in these images is sufficient to directly observe the octahedral tilting in the experimental images in Figure 78f,g; however, the difference is more clearly observable in the Fourier transforms in Figure 78b,c.

**Figure 79:**
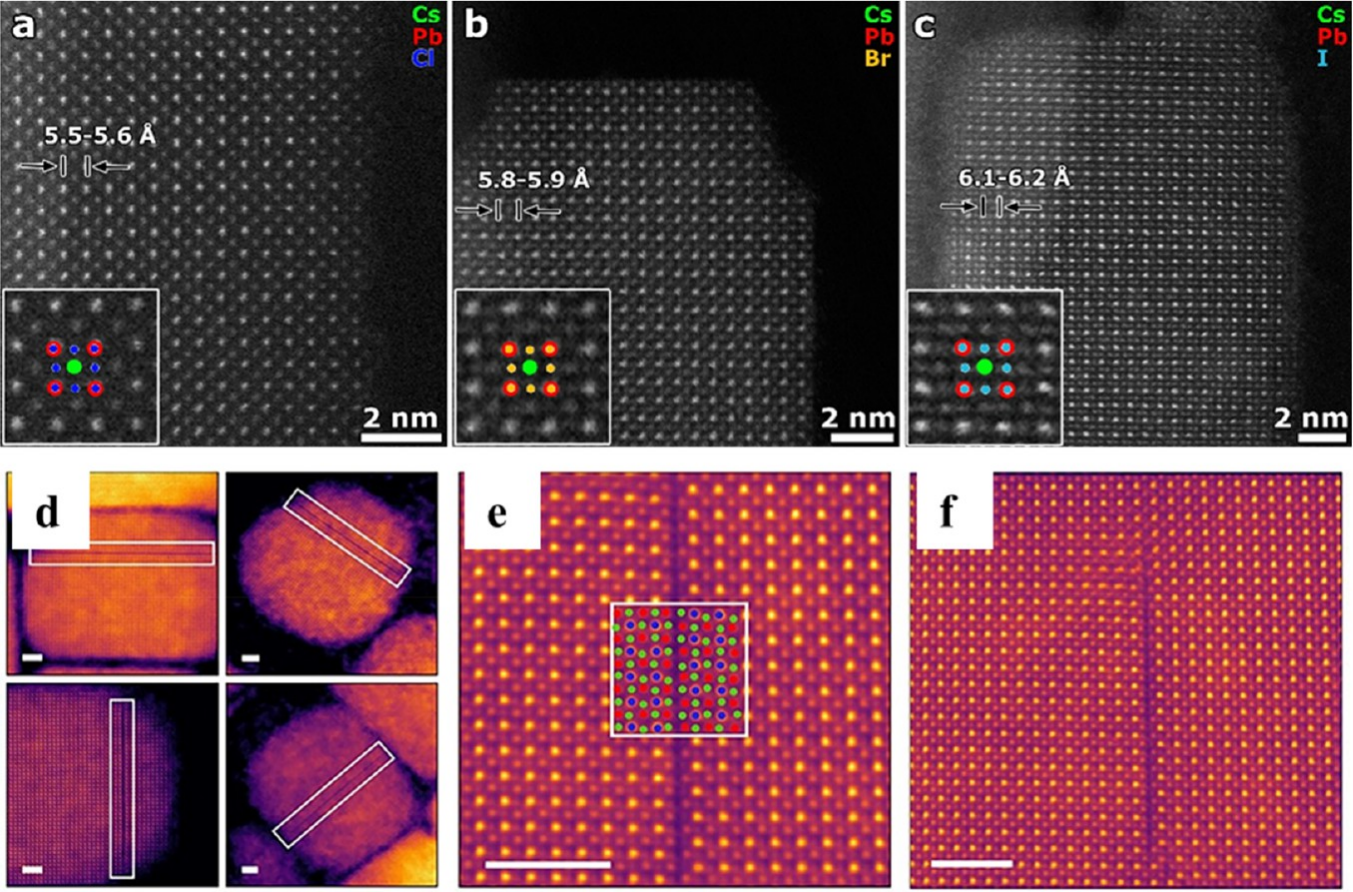
High-resolution HAADF-STEM images of a (a) CsPbCl_3_, (b) CsPbBr_3_, and (c) CsPbI_3_ perovskite nanowire. The different atomic columns are identified using the intensity–atomic number relation in HAADF-STEM imaging. Reproduced with permission from ref (22). Copyright 2016 John Wiley & Sons, Inc. (d) Overview HAADF-STEM images showing the presence of Ruddlesden–Popper planar defects (highlighted as rectangular boxes) in several CsPbBr_3_ NCs. (e) Atomic-resolution HAADF-STEM image of a Ruddlesden–Popper planar defect with an overlaid atomic model (blue, Pb; red, Cs; green, Br). (f) Atomic-resolution HAADF-STEM image of a Ruddlesden–Popper planar defect extending only a few unit cells. The scale bars correspond to 3 nm. Reproduced from ref (707). Copyright 2018 American Chemical Society.

Multiple aberration-corrected high resolution HAADF-STEM studies have been carried out to investigate the crystal structure of all-inorganic lead-halide perovskite NCs.^22,30,73,702,706,707^ The advantage of STEM imaging in comparison to TEM imaging is that the intensity in such an image scales with the projected thickness of the NC and the average atomic number of the elements present along the projection direction. This intensity–atomic number relation can be exploited to identify atomic columns based on their composition if a significant atomic number difference is present for the different elements. Thereby, the use of high resolution HAADF-STEM imaging will enable a direct identification of the different atomic columns in the perovskite NC under investigation (at a location of similar thickness), which is an advantage of using STEM in comparison to TEM. For example, for CsPbrBr_3_ perovskites with *Z*_Pb_ = 82, *Z*_Cs_ = 55, and *Z*_Br_ = 35 (Figure 79b), this relation can be exploited to distinguish the different atomic columns in the cubic [100] or orthorhombic [110] zone in a straightforward manner. In this orientation, the bright atomic columns in the cubic [100] or orthorhombic [110] zone are mixed Pb–I columns with an average atomic number of 58.5 due to the alternating nature of the presence of Pb and I atoms in the column, which have higher atomic numbers than Cs and Br. Subsequently, the Cs atomic columns will appear brighter than the Br columns since Cs is heavier than Br, which have the lowest intensity value. This intensity–atomic number relation (in combination with the knowledge on the crystal structure) will also enable the elemental identification of CsPbrCl_3_ and CsPbI_3_ perovskites (Figure 79a,c, respectively). This powerful technique has been used to study various all-inorganic halide perovskites. For example, Tong *et al.* revealed that CsPbBr_3_ nanowires were formed through oriented-attachment mechanism of initially formed CsPbBr_3_ nanocubes by imaging an intermediate nanowires.^22^ Morrell *et al.* visualized the presence of Ruddlesden–Popper planar defects in CsPbBr_3_ NCs at the atomic level (Figure 79d–f).^707^

##### Organic–Inorganic Hybrid Halide Perovskite NCs

**Figure 80:**
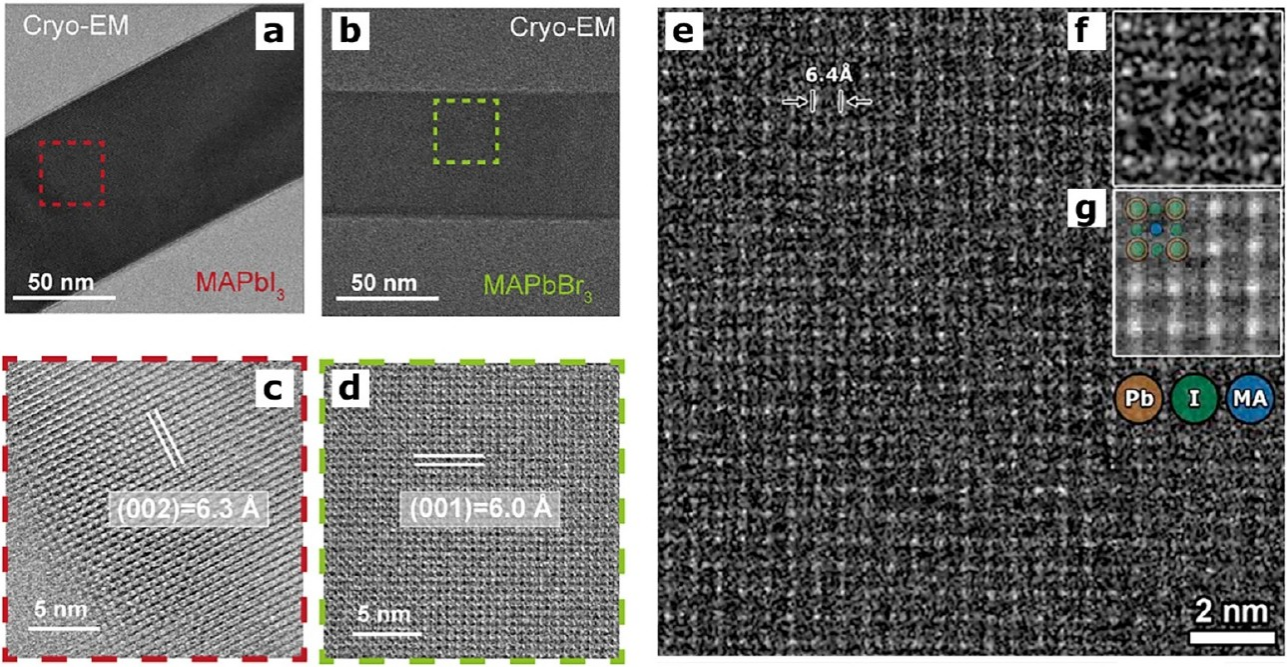
(a–d) Cryo-EM investigation of CH_3_NH_3_PbI_3_ and CH_3_NH_3_PbBr_3_ nanowires. Overview cryo-EM images of both rods are visualized in (a) and (b). Atomically resolved TEM images capturing both the PbI_6_ octahedra and the methylammonium molecules in CH_3_NH_3_PbI_3_ (c) and CH_3_NH_3_PbBr_3_ (d). Panels a–d are reproduced with permission from ref (710). Copyright 2019 Elsevier. (e–g) Low-dose aberration-corrected HAADF-STEM imaging in combination with a template-matching procedure on a CH_3_NH_3_PbI_3_ NC. The atomic arrangement of the CH_3_NH_3_PbI_3_ NC is clearly resolved in the averaged template (g) of the low-dose HAADF-STEM image in (e), performed on the template image in (f). Reproduced with permission under a Creative Commons CC-BY-NC from ref (273). Copyright 2017 The Authors.

The characterization of organic–inorganic hybrid halide perovskite NCs is even more challenging since these perovskites tend to degrade instantaneous upon electron beam illumination.^16,700^ Recently, a few successful studies on methylammonium-based hybrid perovskites have been performed using low-dose high resolution TEM,^30^ cryogenic electron microscopy (cryo-EM),^367^ low-dose aberration-corrected HAADF-STEM^273^ and integrated differential phase contrast STEM (iDPC-STEM).^708^ The initial atomically resolved HRTEM image of a CH_3_NH_3_PbBr_3_ perovskite was collected using a Gatan K2 direct-detection electron-counting camera by Zhang *et al.*(709) The high detective quantum efficiency of a direct-detection camera enables the investigation of highly beam sensitive materials as extremely low-dose conditions can be applied. In this work, they revealed that the CH_3_NH_3_PbBr_3_ crystals consist of ordered nanometer-sized domains with off-centered CH_3_NH_3_ cations with an in-plane and out-plane orientation, which provides direct evidence of the ferroelectric order in CH_3_NH_3_PbBr_3_. Cryo-EM is a technique which is often used to study the native state of a material/specimen by rapidly freezing the material. This technique is mostly used in life sciences. Recently, Li *et al.* preserved the native state of methylammonium-based hybrid perovskites by plunge-freezing the sample in liquid nitrogen which enabled them to observe the atomic structure of the native state of CH_3_NH_3_PbI_3_ and CH_3_NH_3_PbBr_3_ nanowires (Figure 80a–d).^710^ The high resolution cryo-TEM images were acquired at a temperature of −175 °C using a direct detection camera in electron counting mode. The use of such cameras will be of key importance to further progress in the study of these beam sensitive hybrid halide perovskites. In addition to these low-dose HRTEM studies, the use of HAADF-STEM has also been proven successful for the study of hybrid halide perovskites although it is often considered to be more destructive when imaging halide perovskites. Debroye *et al.* were able to retrieve the native atomic structure of CH_3_NH_3_PbI_3_ NCs using low-dose aberration-corrected HAADF-STEM imaging in combination with a template-matching procedure (Figure 80e–g).^273^ The low-dose condition resulted in the acquisition of a single HAADF-STEM image (Figure 80e) with a very low signal to noise ratio hampering the interpretability of the image. The template matching algorithm statistically averaged a small part of the HAADF-STEM image resulting in an image with an improved signal to noise ratio. Such an algorithm searches throughout the image for specific regions which match the template (Figure 80f). In this work, the perovskite lattice of a hybrid lead iodide perovskite was successfully observed in the final averaged template in Figure 80g. This technique can only be used for an averaged observation of the crystal structure, local defects, and/or the surface termination of the NC cannot be investigated using this averaging technique. The development of pixelated electron detectors has enabled another approach for low-dose high resolution STEM imaging using iDPC-STEM. A early iDPC-STEM attempt for the investigation of CH_3_NH_3_PbBr_3_ perovskites was performed by Song *et al*.^708^

#### Going beyond Qualitative Images

**Figure 81:**
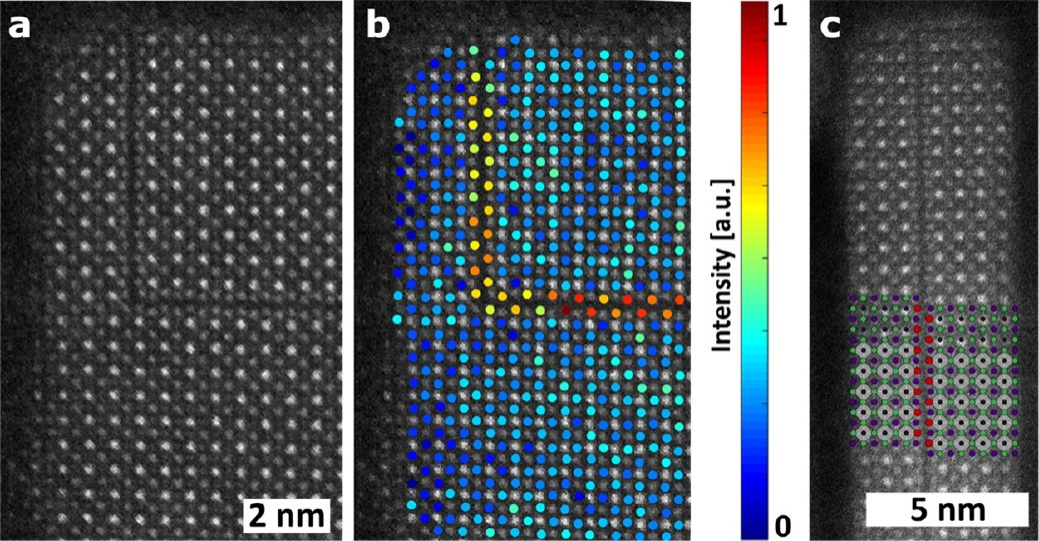
(a) High-resolution HAADF-STEM image of a CsPb(Cl:Br:I)_3_ NC showing the presence of plane shifts. (b) Calculated volume of the fitted Gaussian peaks of the halide columns of the NC indicates increased intensity values of the halide columns around the Ruddlesden–Popper planes, confirming an increased concentration of iodide ions at these positions. (c) Ruddlesden–Popper plane shift model (Cs^+^ = purple, Pb_2_^+^ = black, Cl^–^/Br^–^ = blue, I^–^ = red, and PbX_6_ octahedra = gray) overlapping an HAADF-STEM image of a CsPb(Cl:Br:I)_3_ NC. Reproduced under a Creative Commons CC-BY-NC-ND license from ref (713). Copyright 2019 American Chemical Society.

Quantitative methods are emerging to retrieve additional in-depth information on halide perovskite NCs such as the measurement of lattice parameters unit cell by unit cell. Such a measurement will enable the unambiguous identification of the cubic and orthorhombic structure of lead-halide perovskite NCs, which differ approximately 0.05 Å in lattice parameter. This requires the identification of the different atom types and a precise measurement of their atomic column positions. In addition, the precise localization of the atom positions enables the investigation of possible PbX_6_ (X = Cl, Br, I) octahedral tilt, which is expected in the orthorhombic phase. In principle, such an analysis can be performed both using aberration-corrected TEM and STEM imaging. However, the identification of the atom types in each atom column in atomically resolved TEM images is not straightforward, since the intensity in such images is not sensitive to chemical information. In order to distinct between different atom types, a quantitative statistical phase analysis needs to be carried out. In this manner, tilting of the PbX_6_ octahedron was observed in CsPbBr_3_ nanosheets using in-line holography (Figure 80f,g).^702^ In addition, a unit cell by unit cell characterization of the lattice parameters showed that both the cubic and orthorhombic phases exhibit a lattice expansion compared to their bulk counterpart, while still being able to identify orthorhombic regions from cubic regions as they exhibit smaller lattice distances.^702^ Quantification of the atom positions in an atomically resolved STEM image of a CsPbX_3_ (X = Cl, Br, I) NC can be performed in a more straightforward manner, since the average atomic numbers of the different atom columns are sufficiently large and the intensity in such images scales with the atomic numbers of the present elements. Van der Stam *et al.* confirmed a lattice contraction after a cation exchange in colloidal CsPbBr_3_ NCs resulting in doped CsPb_1–*x*_M_*x*_Br_3_ NCs (M = Sn^2+^, Cd^2+^, and Zn^2+^; 0 < *x* ≤ 0.1).^304^ Here, the lattice parameters are quantified using statistical parameter estimation theory^711,712^ to retrieve the atom positions of each atom column. In addition, the intensity–atomic number relation in HAADF-STEM imaging can be used to identify different atom types in mixed-halide perovskites. Akkerman *et al.* investigated all-inorganic Ruddlesden–Popper double Cl–I and triple Cl–Br–I lead-halide perovskite NCs and the position of the different halides in the perovskite structure using quantitative high resolution HAADF-STEM imaging (Figure 81).^713^ The intensities of the halide atom columns were calculated by fitting a Gaussian function to each atom column (Figure 81b). This work revealed that the small amount of iodide clusters at the Ruddlesden–Popper planes. Until now, quantitative (S)TEM techniques have only been applied successfully to all-inorganic halide perovskite NCs.

#### Summary and Outlook for Electron Microscopy Studies on MHP NCs

The previous sections have shown that halide perovskites have been studied successfully at the atomic level using a range of techniques. Although these perovskites are very sensitive to the electron beam, the use of a parallel beam as a focused probe has been exploited. Most of these studies dealt with beam damage and therefore often low-dose conditions are required to study the native state of these halide perovskites. Recently, a few successful studies have been performed on organic–inorganic hybrid halide perovskite NCs. The use of detectors with a high detective quantum efficiency has played a big role in lowering the necessary dose needed to study the native state of hybrid halide perovskites. Despite recent advances, there are still many challenges in electron microscopy of perovskite NCs. For example, quantitative determination and location of dopants in perovskite NCs is one of the main challenges to be addressed for a better understanding of doped-perovskite NCs. It is well-known that LHPs undergo phase changes at certain temperatures, and this has often been studied by optical and X-ray characterization. It would be very interesting to probe such phase changes at the atomic level with *in situ* electron microscopy characterization at the single-particle level to obtain additional insights. Another important challenge is to apply 3D atomic imaging techniques to perovskite NCs to study their crystal structures.

### X-ray Scattering Techniques and Their Impact on the Stability and Degradation Analysis of Perovskite NCs

X-ray scattering is a powerful technique to investigate structures not only on atomic lengths scales (angstroms, Å) but also on the mesoscale (nanometer). High time resolution is feasible, especially with synchrotron radiation, and *in situ* investigations on many different NC systems are conceivable. This approach gives insights into the kinetics as well as structure–function correlations. In particular, when coupled to other *in situ* techniques, *e.g*., UV/vis or photoluminescence measurements, X-ray scattering is a versatile and fruitful technique for providing a quantitative understanding.

So far, X-ray scattering techniques have shown a high impact by analyzing the crystal structure of perovskite NCs: in addition to probing the inherent crystal structure (crystal lattice topography), the ordering and alignment of perovskite NCs (superstructure) can be analyzed. Thus, X-ray scattering techniques are a precise analysis tool for crystal and super-structure, crystal orientation, phase identification and phase change tracking in perovskite NCs, which are used in different areas ranging from photovoltaic and photodetectors to LEDs.^223,334,714−716^

The focus of this section lies in the application of various X-ray scattering techniques on perovskite NCs, ranging from common XRD measurements to advanced synchrotron-based *in situ* measurements with 2D detectors. In particular, studies about stability and degradation will be mentioned, as well as studies about structure–function correlations. We aim to give also insights into more advanced scattering techniques such as grazing-incidence small- and wide-angle X-ray scattering (GISAXS and GIWAXS) and experimental setups that will help to improve perovskite NC research and facilitate the road toward broader use of mentioned methods.

Fundamental understanding of the stability of perovskites is still one of the big challenges in the field.^85,110,717^ Thus, mechanisms of degradation have to be investigated in detail and, whenever possible, with high time resolution. Investigations that capture processes in real time are commonly referred to as *in situ* (in place) in contrast to *ex situ* (out of place) experiments, that only capture the status after the time-dependent process. *In situ* experiments usually pose additional experimental challenges, *e.g*., the necessity of high flux X-ray radiation (*e.g*., *via* synchrotron access) and transportable experimental setups, detailed knowledge of reaction kinetics, as well as considering damage induced by the high-intensity X-ray beam. *In situ* and *operando* studies have already been used heavily on bulk and thin-film materials and offer many possibilities in perovskite NC thin-film analysis including the elucidation of superstructural features.^718−722^

#### Introduction to X-ray Scattering Methods Used in the Characterization of MHP NCs

Elastic X-ray scattering is a nondestructive reciprocal space technique, *i.e*., it yields the Fourier transform of the electron density of the probed material. This results in a diffraction pattern that contains information about typical reciprocal distances in the sample, denoted . These distances can be probed by X-ray scattering. Photons of wavelength λ impinge on the sample and are scattered if they fulfill the Laue condition  with incoming wavevector , final wave vector  and reciprocal lattice vector . Thus, the momentum change of the photon depends on the structural lattice ordering of the sample. The momentum change *q* can be converted to a real space distance *d* using the equation *q* = 2π/*d*. The scattering event results in a change in the photon’s trajectory which can be given as an angle 2θ using the Bragg equation *n*λ = 2*d* sin θ. Diffraction peaks (reflexes) are indexed according to the diffractive planes that give rise to the interference pattern. For indexing, Miller indices (*hkl*) are used. Further details about diffraction techniques on functional material, *e.g*., perovskite LED and PV application, can be found in literature.^677,723,724^

In laboratories, XRD in Bragg–Brentano reflection geometry is well-suited for thin-film studies including perovskite NCs. In addition to classical XRD measurements, with the use of 2D detectors, additional scattering methods have been established. Depending on the detector placement, small- or wide-angle X-ray scattering (SAXS or WAXS) can be observed, which corresponds to large and small distances probed, respectively. Whereas SAXS and WAXS are very powerful for the analysis of volume samples, to study supported thin films can be challenging. A substantial contribution from the support material can challenge the analysis of the thin film. In such cases, grazing-incidence small- and wide-angle X-ray scattering offer possibilities for structure analysis. GISAXS and GIWAXS are performed in reflection geometry with a fixed grazing-incidence angle (α_i_ ≪ 1°). This offers the possibility to minimize substrate contributions to the scattering signal by selecting an incidence angle below the critical angle of the substrate, thus preventing the penetration of the incident beam into the substrate and/or subsequent layers. X-ray scattering is not a local method like high-resolution real-space imaging and can probe an ensemble of small crystallites, with the probe volume depending on the beam size. In particular, when considering the grazing-incidence geometry, the illuminated surface area can be rather large (order of mm^2^). The probed volume depends on the penetration depth, which is dependent on the X-ray wavelength and the sample material.

**Figure 82:**
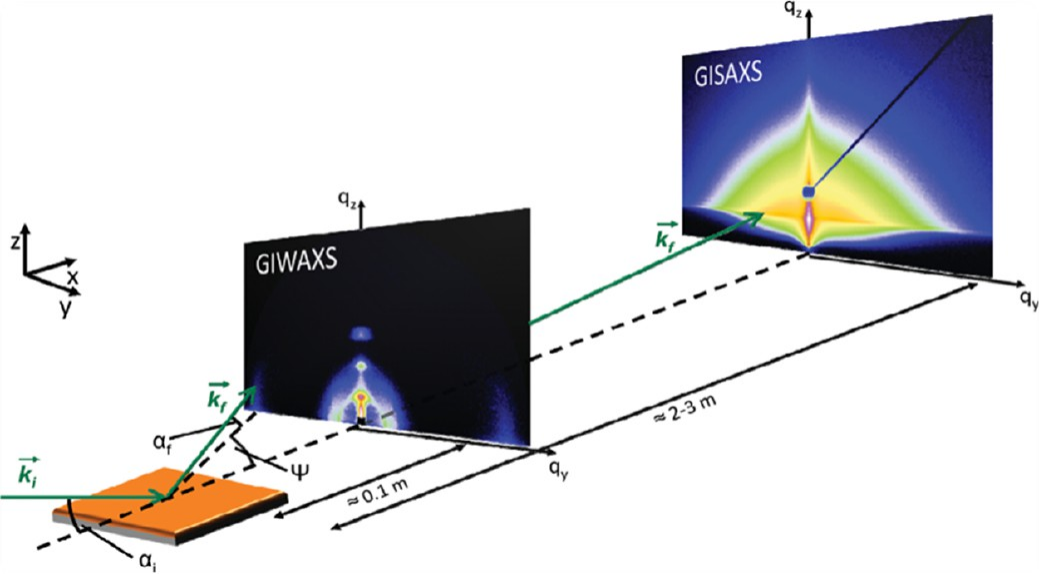
Schematic illustration of a typical GIXS setup with reference geometry. Photons with wavevector  impinge on the sample under an incidence angle α_*i*_. Scattered photons with wavevector  leave the sample under an in-plane exit angle α_f_ and an out-of-plane exit angle Ψ. GIWAXS and GISAXS require different sample–detector distances, typically in the range of around 100 mm and 2–3 m, respectively. Reproduced with permission from ref (725). Copyright 2019 John Wiley & Sons, Inc.

A typical experimental GIXS setup is shown in Figure 82.^725^ The reference coordinate system is commonly placed onto the sample surface, with *z* being normal to the surface, *x* along the beam direction and *y* perpendicular to the *xz* plane. When placing the 2D detector rather close to the sample (around 100 mm), GIWAXS patterns can be observed. GIWAXS probes the crystalline part of the sample and results in a 2D diffraction pattern on the detector. Questions that aim at texture or morphology analysis can only be partially answered by XRD, since only a small region around  is probed. In GIWAXS, however, a full 2D plane in *q*_*r*_ and *q*_*z*_ is recorded. The image on the detector is a result of the orientation sphere of the reciprocal lattice points cutting the Ewald’s sphere. Unfortunately, the projection onto a 2D grid results in a range of missing *q* values, because *q*_*x*_ ≠ 0. The usual 2D representation in reciprocal space plots the momentum change *q*_*r*_*versus* the momentum change in *z*-direction *q*_*z*_. Thus, in addition to the classical crystal structure, it can give information about the preferential orientation or texture of crystallites on the sample. Diffraction peaks and rings are labeled in analogy to XRD patterns. From the width of the Bragg diffraction peaks and rings the upper limit for crystallite size can be extracted using the Debye–Scherrer equation.^726^ Bragg spots can arise for highly ordered systems with long-range order, *e.g*., single crystals or ordered superlattice diffraction of NCs, due to distinct points in the reciprocal lattice space of those systems. In contrast, isotropic orientation of the crystallites results in a powder scattering pattern, which is identified by the ring-shaped and uniform intensity distribution on the 2D detector.

When moving the 2D detector to larger distances on the order of 1–4 m, a GISAXS signal can be recorded. GISAXS probes distances on the mesoscale (nanometer regime) and is commonly used to investigate the morphology, *i.e.*, domain sizes and interdomain distances of thin films or superstructures of NCs. Not only the crystalline parts of the sample contribute to the scattering, since GISAXS probes the dispersion of the sample, which in turn is related to the scattering length density (SLD). SLD is a material-specific property. Refraction inside the film leads to enhanced out-coupling under the critical angle of the thin film (so-called Yoneda peak). By analyzing this material-sensitive Yoneda region by horizontal line cuts (in *q*_*y*_ direction), material-specific structure information is accessible. For the analysis commonly the so-called distorted wave Born approximation (DWBA) is combined with several approximations such as the effective interface approximation (EIA) and the local monodisperse approximation (LMA). For more information the reader is referred to the literature.^727,728^ In addition, GISAXS patterns of highly ordered systems show Bragg peaks similar to GIWAXS, which, however, originate from a larger-scale structure as compared to GIWAXS.^729−733^

#### 1D X-ray Diffraction Measurements

**Figure 83:**
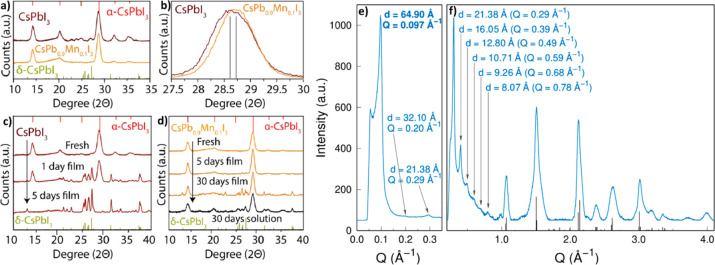
(a) XRD patterns of CsPbI_3_ and Mn^2+^-substituted phase. The calculated α- and δ-phase patterns are shown in red and green, respectively. (b) Zoom in of (a) to visualize the shift in Bragg peak position due to Mn^2+^-induced lattice parameter changes. (c,d) Time-dependent XRD patterns of CsPbI_3_ and CsPb_0.9_Mn_0.1_I_3_ over several days, showing the difference in degradation kinetics. Reproduced from ref (607). Copyright 2017 American Chemical Society. (e,f) Low- and high-angle XRD pattern of CsPbBr_3_ nanoplatelets. The first peak corresponds to a distance of the NPs of 6.5 nm. Higher harmonics are also visible, which confirms the high degree of long-range order. The NPs have a thickness of around 3.5 nm, as confirmed by TEM imaging. From the high-angle region, an orthorhombic structure could be determined. Reproduced from ref (210). Copyright 2019 American Chemical Society.

Common XRD measurements with 1D detectors are probably the most frequently used X-ray scattering technique and available in many laboratories at moderate cost. The collection of XRD patterns can be a powerful and comparatively easy tool to identify and distinguish phases in a sample. A complete measurement can often be conducted in less than 1 h including sample preparation and measurement setup. Measurements of thin films, *e.g*., when dealing with PeNCs deposited on a substrate, are possible using the Bragg–Brentano geometry.^80,315,450,734^ Straightforward studies employ *ex situ* XRD measurements. By comparing XRD patterns to libraries, previous measurements, or literature, crystalline phases can be identified with a high degree of certainty.^223,274,450,606,681,735,736^ For example, Bertolotti *et al*. used X-ray scattering techniques to analyze the long sought after crystal structure of thin films of CsPbBr_3_ NPls.^210^ The high asymmetry of NPls favors narrow-band emission acting as nanowells with well-defined dimensions and low variation, thus resulting in discrete band gaps, which are very beneficial for LED application. The crystal structure of NPls is not easily accessible because of their quasi-2D shape. In their study, a combination of low- and high-angle XRD and wide-angle X-ray total scattering (WAXSTS) was used. The high-angle XRD region interestingly suggested an orthorhombic crystal structure as shown in Figure 83f. The result was confirmed *via* Debye scattering equation modeling.^726,737,738^ Discriminating between different phases—even for small crystallites like NPls—is an important feature of X-ray scattering by accessing a statistically relevant ensemble of crystallites. The low-angle region in XRD on the other hand, gives information about large distances present in the structure. In this case, it revealed interplatelet distances as shown in Figure 83e. The first peak appeared at *q* = 4π sin θ/λ = 0.097 Å, which corresponds to a distance of *d*_1_ = 64.90 Å. The high degree of long-range ordering was confirmed by the high number of harmonics toward higher 2θ values marked with *d*_n_ in Figure 83f.

#### Time-Dependent XRD Studies

To investigate kinetic changes, time-dependent XRD measurement protocols are well-suited. This is especially useful for degradation studies that occur over many hours up to months, and the same measurement is repeated at certain intervals. Numerous stability related studies were done on perovskite NC systems and examined by time-dependent XRD studies.^315,607,736,739^ For example, MAPbBr_3_ perovskite NCs can be effectively stabilized by essential amino acids as identified by an unchanged XRD pattern over 6 months.^735^ CsPbBr_3_ NCs for white LEDs showed higher resistance against heat and moisture-induced degradation by coating with alkyl phosphate^274^ and CsPbX_3_ NCs were effectively stabilized by a PMMA matrix as shown by time-dependent XRD studies over several days in 80% relative humidity.^739^

A different approach toward stabilizing CsPbI_3_ was reported by Akkerman *et al.* and confirmed by time-dependent XRD measurements.^607^ It is well-known that the stability of the cubic perovskite α-phase is connected to the Goldschmidt tolerance factor and thus the stability can be tuned by site occupation substitution.^740^ Pristine CsPbI_3_ suffers from poor stability and is unstable in air. The cubic α-phase decomposes rapidly (within days) into the yellow δ-phase, and corresponding time-dependent XRD data are shown in Figure 83c,d. To obtain stable cubic α-CsPbI_3_, Pb^+^ was partially replaced by Mn^2+^ without significant changes to the crystal structure and, more importantly, without inducing significant changes in PL, trPL, and absorption properties of the material, as was shown by Liu *et al.*(606) Klimov *et al*. showed that Mn^2+^ doping can even be beneficial for its emission properties.^605^ By adding MnI_2_ to the precursor solution, alloying could be achieved by Akkerman *et al*. resulting in a cubic drop-cast CsPb_*x*_Mn_1–*x*_I_3_ phase. Meanwhile, the octahedral (Pb/Mn)O_6_ geometry was preserved. The partial substitution of Pb^2+^ with Mn^2+^ led to a small reduction in unit cell size and more favorable Goldschmidt tolerance factor for a cubic system (see also previous sections on doping/alloying of NCs). Thus, the structure factor of the crystallographic unit cell changes, which resulted in a changed X-ray diffraction pattern. The decrease in unit cell was verified by XRD measurements reported in Figure 83b. CsPb_*x*_Mn_1–*x*_I_3_ showed increased stability as proven by time-dependent XRD measurement over the course of 4 weeks. In Figure 83d the partial transition toward the orthorhombic δ-phase can be seen starting on day 5 at ∼25-28° 2θ. As predicted by DFT calculations a lattice contraction of around 1% was observed in XRD (cf. Figure 83b) for the chemical composition CsPb_0.91_Mn_0.09_I_3_ resulting in decreased metal–iodine bonds.

Challenging for PeNCs, especially for CsPbI_3_ NCs, is the poor stability against illumination. Boote *et al.* followed the degradation of drop-cast CsPbX_3_ NCs thin films by time-dependent XRD for up to 16 h under 1 sun irradiation and ambient conditions.^736^ They found that CsPbBr_3_ was phase-stable (orthorhombic γ-phase) under 1 sun illumination for up to 16 h and when heating up to 250 °C. CsPbI_3_, however, was most unstable in the CsPbX_3_ series as the nonluminescent yellow phase appeared, as can be identified by decreasing Bragg diffraction intensity, which indicated decomposition into a noncrystalline or amorphous phase.^736^ However, after some hours of illumination and before the loss of crystallinity occurred, CsPbCl_3_ and CsPbBr_3_ showed an increased intensity and decreased fwhm of the (100) and (200) reflexes. This led to the conclusion of crystal growth and possibly oriented crystal growth with a changed preferential orientation of the NCs. However, XRD by itself is only partly able to elucidate the texture of an ensemble of crystallites. Preferential orientation is better probed by (GI)WAXS, which is described below. CsPbI_3_ thin films washed with methyl acetate solution, for example, showed no change in Bragg peak intensity and were stable under continuous illumination.^732^ XRD confirmed the same phase and no observable crystallographic changes under illumination. This highlights the importance of surface quality in perovskite NCs and their influence on the PeNCs stability.

XRD studies can also help to elucidate degradation mechanisms. It is known that CsPbBr_3_ degrades to a yellow phase under illumination, which is accompanied by a strong PL-quenching, thereby decreasing the EQE of an LED-device drastically. Huang *et al*. carried out studies with different stress factors on the device, *e.g*., illumination, oxygen, humidity and temperature.^223^ Illumination of 175 mW/cm^2^ for 8 h led to a color change of the thin film from green to yellow. The degradation was tracked using time-dependent XRD measurements. The cubic (100) and (200) Bragg reflexes of the perovskite NCs first broadened and then increased in intensity and sharpness. This indicated a crystal growth and thus was correlated with an observed PL red shift. Under higher illumination strength of 350 mW/cm^2^ the degradation species PbO was identified *via* XRD after 8 h. The driving force of degradation was determined to be oxidation (by oxygen) in combination with illumination strength and moisture, which seemed to support ion migration in crystal growth. Supported by XRD analysis, it was shown that under oxygen stress but no illumination no yellow phase and no PL loss occurred. Li *et al*. showed by XRD analysis that cubic CsPbBr_3_ NC-495 thin films also degraded into PbCO_3_ and PbO and Cs_4_PbBr_6_ under illumination in ambient conditions.^741^ Larger NC-520 thin films did not decompose within 20 h of illumination.

#### 2D GIXS Imaging

##### Applying Advanced X-ray Scattering Techniques

When texture and morphology information about the sample are of critical interest, XRD can only supply insufficient information, since it only provides information along *q*_*r*_ ≈ 0. For texture and/or morphology investigations, a larger *q*-space needs to be probed. As described above, small- and wide-angle X-ray scattering onto a 2D detector can be the solution to this problem. Details about those measurement techniques are described above.

**Figure 84:**
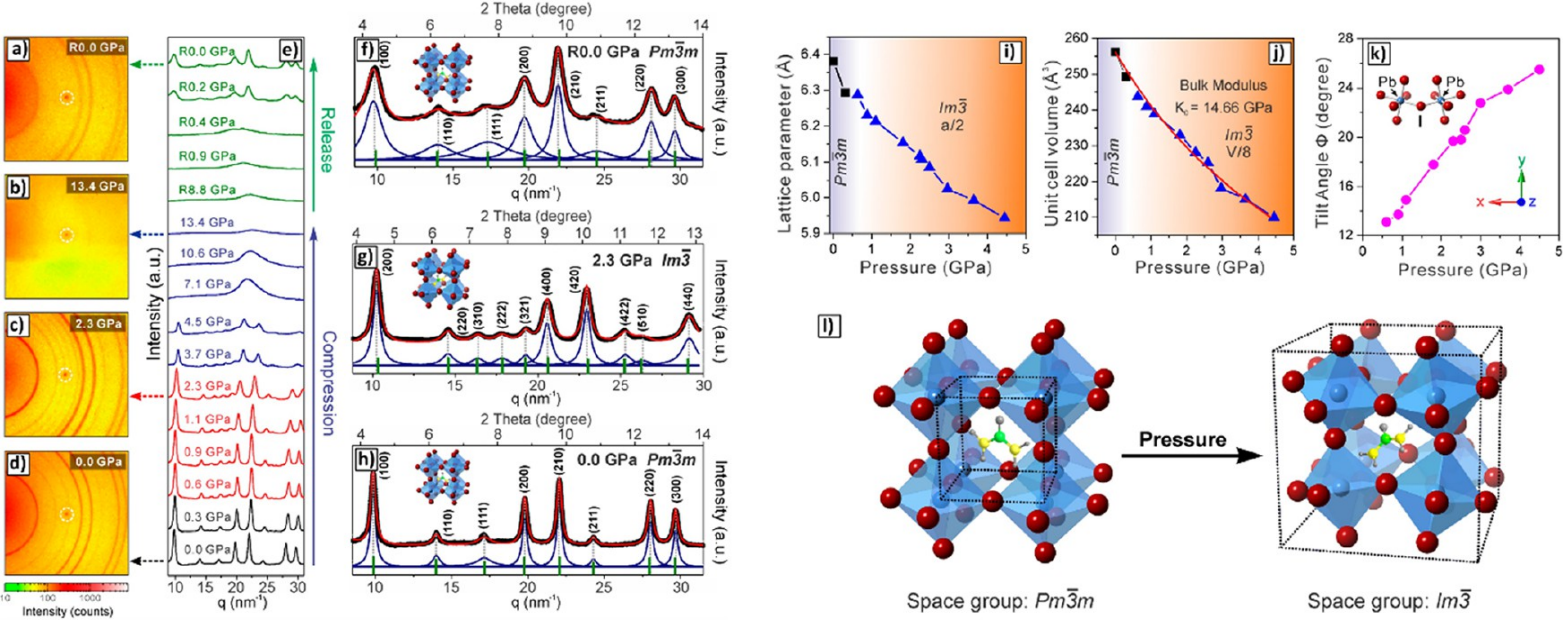
(a–d) *In situ* GIWAXS patterns of FAPbI_3_ NCs during compression and subsequent decompression with (e) corresponding pseudo-XRD patterns (radially integrated GIWAXS images). White circles represent noise. (f–h) Fitted and indexed pseudo-XRD patterns with structure representation and corresponding calculated reflex positions. (i–k) Derived lattice parameters, unit cell volume, and octahedral tilt angle evolutions depending on pressure. (l) Schematic representation of the structural changes occurring during pressure increase. The [PbI_6_]^4–^ octahedron tilts along the cubic [111] direction. Reproduced from ref (742). Copyright 2018 American Chemical Society.

For example, Zhu *et al.* investigated the phase transitions of FAPbX_3_ NCs, X = Cl, Br, I, by *in situ* WAXS and UV/vis measurements during the application of pressure in the range from 0 to 13.4 GPa.^742^ Pressure was applied using a customized diamond anvil cell that enabled WAXS measurements at a synchrotron at the same time. Radial integration of (GI)WAXS images lead to a pseudo-XRD plot (signal intensity *vsq*) that can be indexed in analogy to XRD patterns. Indexing the cut at ambient conditions showed a cubic space group (*Pm*3̅*m*) and a lattice constant of *a* = 6.35 Å. While increasing the pressure the WAXS pattern changed. First, additional Bragg rings appeared as seen in Figure 84a–d, which was attributed to a different cubic phase (*Im*3̅). Corresponding pseudo-XRD patterns are shown in Figure 84e. Further increase in pressure led to increased tilting of the [PbI_6_]^4–^ octahedron (cf. Figure 84f–h) and increasing fwhm of the Bragg rings. The latter usually indicates smaller crystallites or a loss in crystallinity. As expected, a decrease in lattice parameters was observed (red-shifted *q* values). Before the sample finally transformed into the amorphous state, degradation into the orthorhombic phase (*Pnma*) was observed. The amorphous state was reversible when decreasing the pressure below 0.4 GPa. At this point, a fast reordering into the original cubic *Pm*3̅*m* phase occurred. However, the Bragg rings showed a broadening compared to the original sample at ambient conditions, indicating a slight loss in crystallinity of the FAPbI_3_ NC film. Often scanning electron microscopy or transmission electron microscopy measurements are chosen to verify and improve the structure model developed through X-ray scattering methods. With TEM measurements, it was confirmed that no significant change in particle size and shape was induced by the pressure cycle. The complete lattice parameter and unit cell volume evolution as deduced from WAXS analysis is plotted in Figure 84i–k. The corresponding tilt of the [PbI_6_]^4–^ octahedron is shown in Figure 84l. *In situ* PL and UV/vis measurements showed a pressure tunable band gap between 1.44 and 2.17 eV. This WAXS study successfully correlated structural changes to optoelectronic properties that might be vital for further research and the development of industrial production techniques. The results may influence the fine-tuning of the band gap for applications in optoelectronic devices like PV or LEDs.

#### Investigation of Superstructures by Advanced X-ray Scattering Techniques

Perovskite materials can be driven to self-assembly into 1D, 2D or 3D superlattices which has given rise to focused research on targeted functionalization of low dimensional perovskites and perovskite NC superlattice structures.^160,319,677,684,716,743−747^ Improved strategies to control shape and size have been found in recent years and targeted tuning is within reach.^319,748^ As more methods for self-assembly and directed superlattice growth of NCs become available also the need for more detailed structural, superstructural and morphological characterization techniques arises. Long-range ordering of the NCs leads to a scattering signal. However, depending on the magnitude of the superlattice parameters, too long distances cannot be probed by conventional XRD. Long distances, corresponding to exceedingly small diffraction angles of less than 2° 2θ are better accessible by increasing the sample detector distance to several meters. (GI)SAXS is a suitable tool to investigate superlattice ensembles revealing information in *q*_*y*_ and *q*_*z*_ direction.^677,724,744,749^

Horizontal line cuts can be performed on the 2D GISAXS data in the Yoneda region, which gives information about the typical stacking distances present in the sample. From the *q* ratio of those peaks a first structure model can be derived, *e.g*., from the *q* ratios *q*:√*q*:2*q* for a simple cubic superlattice structure.^191,196,684^ Further information about GISAXS interpretation and morphological modeling can be found above. In particular, in combination with TEM/HRTEM and fast Fourier transform (FFT) analysis of real-space imaging, (GI)SAXS can give precise information about superlattice stacking, as explained above.^160^ The interplay between superstructure and crystal structure changes, and optoelectronic properties is of key interest for optoelectronic device research. The combination of *in situ* (GI)WAXS and (GI)SAXS can be immensely powerful to track phase transition and superlattice changes simultaneously. Real-space methods like SEM/TEM/STM can be used complementary to reciprocal space imaging techniques and probe local areas and ensemble information, respectively.^750^

**Figure 85:**
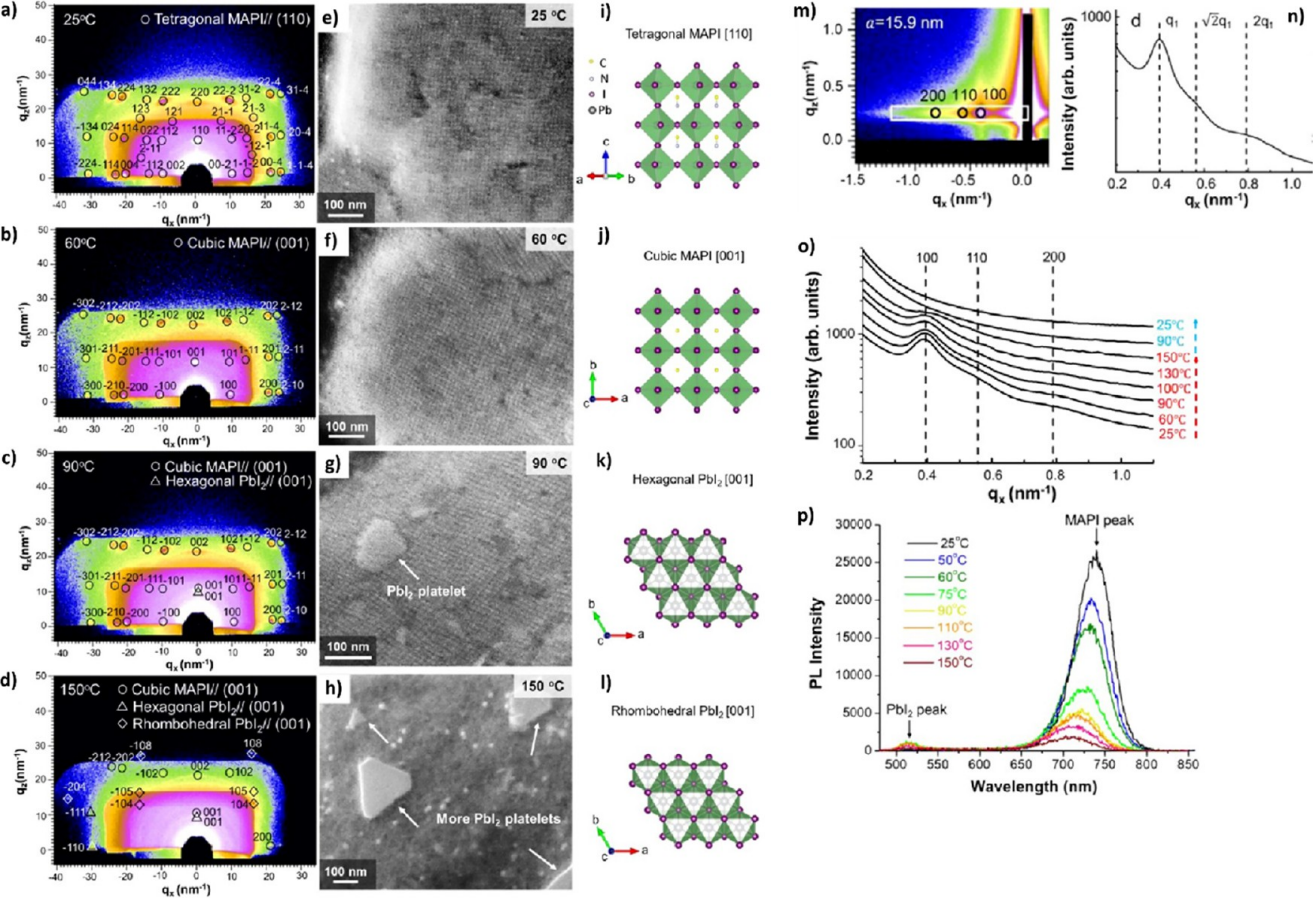
(a–d) *In situ* GIWAXS images of MAPbI_3_ NCs heated to 150 °C. The attenuation on the side is due to experimental restrictions. The pattern suggests high ordering with the tetragonal (110) plane oriented parallel to the substrate. A phase transition from tetragonal to cubic is observed around 60 °C. At higher temperatures, hexagonal and rhombohedral PbI_2_ can be identified as a degradation product. (e–h) Corresponding SEM images and (i–l) corresponding derived structure representations. (m,n) GISAXS image and azimuthal cut to determine the cubic superstructure with a superlattice constant of 15.9 nm. The three largest distances are marked. (o) Horizontal GISAXS cuts (along *q*_*x*_), showing a clear loss in ordering around 150 °C. The diffraction peaks stem from a cubic superlattice. (p) PL emission spectra taken with an excitation wavelength of 442 nm. Reproduced from ref (160). Copyright 2019 American Chemical Society.

**Figure 86:**
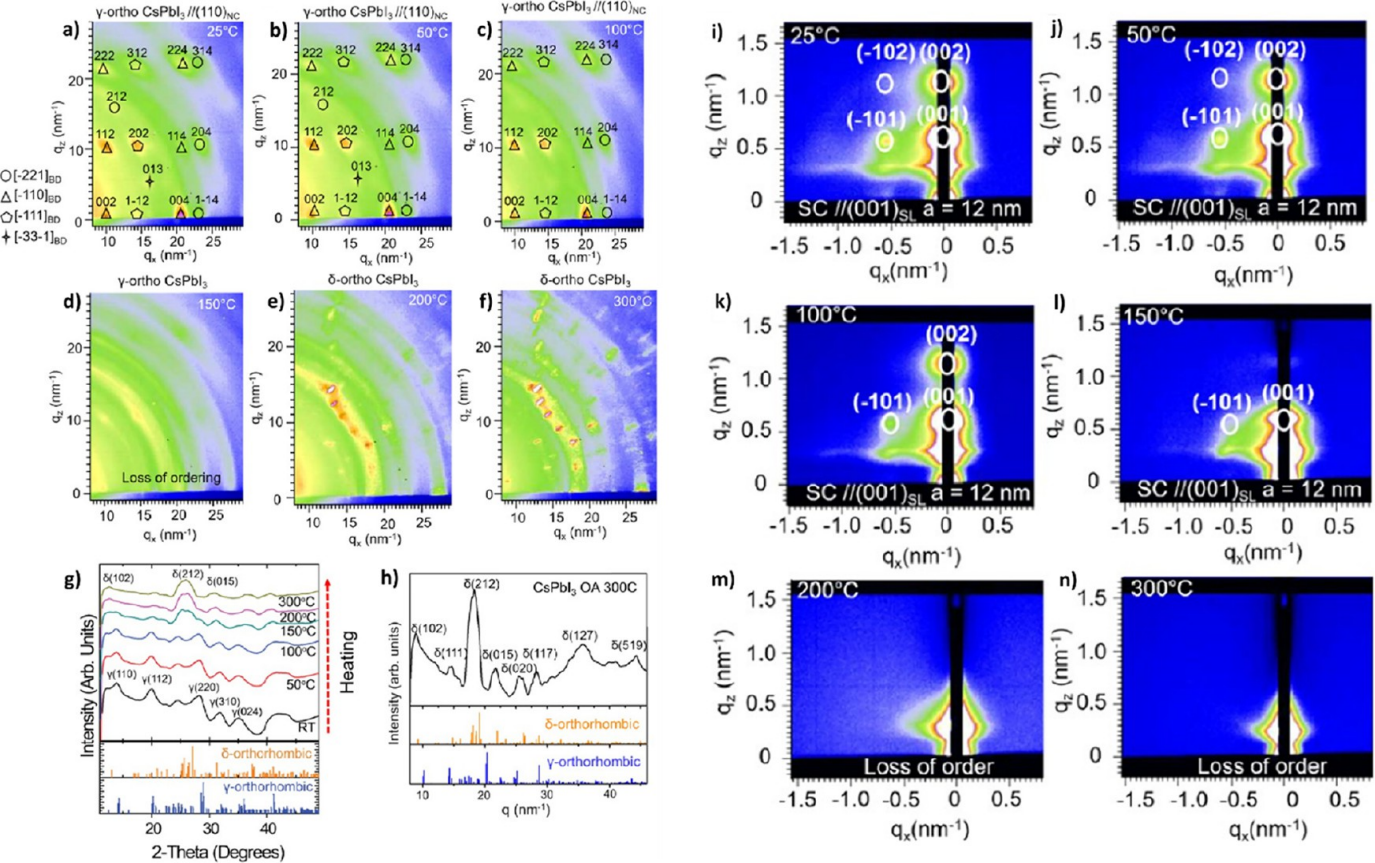
(a–f) *In situ* GIWAXS images of CsPbI_3_ NCs taken during heating from RT to 300 °C. Distinct Bragg spots indicate high ordering with the orthorhombic (110) plane parallel to the substrate. (g,h) Pseudo-XRD patterns generated by radial cuts to compare with calculated XRD patterns of the γ- and δ-phase. Bragg peaks of interest are indexed to the corresponding phase. (i–n) *In situ* GISAXS images taken during the heating process. Clear long-range ordering of the NCs into a superstructure is visible. The loss of ordering starts around 150 °C. Reproduced from ref (191). Copyright 2019 American Chemical Society.

For example, Zhang *et al.* investigated the thermally induced crystal and superstructural changes of luminescent cuboidal MAPbI_3_ NCs by *in situ* GISAXS and GIWAXS imaging.^160^ They found that the chosen evaporation method formed MAPbI_3_ NC films with an ordered superlattice. In Figure 85m, a GISAXS image of MAPbI_3_ NCs is shown which exhibits distinct in-plane features that can be indexed to a cubic superlattice. A horizontal line cut (cf. Figure 85n) shows a distinct peak at ∼0.4 nm^–1^, which corresponds to a superlattice constant of around 15.9 nm. GISAXS images were taken during the heating and cooling process and an evolution of cuts is shown in Figure 85o. Thereby the authors could show that the ordering of the lattice persists under elevated temperatures until approximately 150 °C. This agreed with steadily decreasing PL intensity, as shown in Figure 85p. GIWAXS patterns (cf. Figure 85a) were indexed to a tetragonal space group with an orientation of mainly the (110) plane parallel to the substrate. Distinct Bragg spots were visible in the GIWAXS pattern, which agreed with the high ordering of a superlattice. Upon heating to 60 °C, a phase transition from tetragonal to cubic was observed. *In situ* GIWAXS patterns, corresponding SEM images, and schematic representations are found in Figure 85a–l. When reaching 90 °C, MAPbI_3_ NCs started to decompose and highly oriented hexagonal (001)-PbI_2_ was found. At 150 °C rhombohedral PbI_2_ (*R*3̅*m*) was visible in the GIWAXS pattern as a degradation product, and all superlattice ordering was lost (cf. Figure 85d,h,o). In this study, the scattering methods of *in situ* GIWAXS and GISAXS were used in combination with real space SEM/TEM imaging to elucidate the exact phase at varying temperatures, phase transition points, phase changes and preferential orientations of MAPI NCs during thermally induced degradation. Thomas *et al.* applied *in situ* GISAXS and GIWAXS to investigate the heating response of all-inorganic cube-shaped CsPbI_3_ NCs under humid conditions in air.^191^ The perovskite NCs were ligand-stabilized to improve their resistance to moisture degradation by providing a hydrophobic shell. GIXS was used to investigate the degradation and phase transitions as well as loss of superstructural ordering. Indexing of GIWAXS patterns taken at RT showed the γ-orthorhombic phase (*Pbnm*), as shown in Figure 86a,g. The spot-like pattern indicated a high degree of ordering into a superlattice with γ-(110) and γ-(002) being oriented parallel to the substrate. Often, indexing is tested for different space groups to sufficiently explain the full diffraction pattern. In this case, indexing with a cubic phase left some Bragg spots unexplained and therefore the γ-phase was favored. The black γ- or α-phase of CsPbI_3_ is the optoelectronically interesting phase as opposed by the yellow δ-phase. *In situ* GIWAXS imaging while heating γ-CsPbI_3_ from RT to 300°C under 40% relative humidity, revealed the γ- to δ-phase transition occurring at ∼150 °C (cf. Figure 86a–h). GISAXS suggested a simple cubic superstructure with a lattice spacing of 12 nm and (001)_SL_ orientation (cf. Figure 86i–n). A complete loss of the cubic superlattice ordering was observed at 200°C. Whether the phase transition leads to a loss in superlattice ordering or whether a loss in ordering makes a phase change more favorable is difficult to tell. Thomas *et al.* believe that the main driving force was the thermally induced loss in surface-capping ligands.

GIXS can also be coupled to other *in situ* techniques like TEM, PL or UV/vis, which can be a powerful approach to investigate degradation and structure–function relations. For example, Zhang *et al.* applied a combination of HRTEM/FFT and GISAXS/GIWAXS imaging to lead-free cubic Cs_2_AgBiBr_6_ perovskite NCs.^193^ Disintegration of the superlattice was observed around 200 °C and total loss of ordering of the cubic superlattice was reached at 250 °C. Jurow *et al.* used GISAXS to find correlation distances of 3.8 nm in *q*_*z*_ direction of CsPbBr_3_ NCs when tuning the transition dipole moment for improved optical characteristics.^751^ An interesting alternative to (GI)SAXS superlattice analysis is wide-angle parallel beam X-ray scattering as done by Toso *et al.*(692) They used the fact that highly ordered CsPbBr_3_ NCs form superlattice scattering planes for previously diffracted X-rays that stem from scattering on crystal lattice planes. This interference gives rise to equally spaced satellite peaks and its position is given by *q*_*n*_ = 2π*n*/Λ, with Λ being the average superlattice spacing. With this method, an average spacing of Λ ≈ 12.2 nm was found.

#### X-ray Scattering on Colloidal Dispersions

X-ray scattering is not limited to solid bulks or thin films. Colloidal perovskite NCs in solution can also be investigated by scattering techniques to give insight into the crystal structure and morphology. Precursor engineering has been an important, though not very precise nor predictable method to optimize perovskite materials.^752^ For example, Pratap *et al*. investigated colloidal perovskite precursor dispersions by GIWAXS and UV/vis and found four stages of thin-film formation: nanoparticles in solution, nanoparticle growth, formation of aggregates and complex clusters, and fragmentation of large aggregates.^753^ Thus, the key steps in thin-film formation for device fabrication could be looked at in detail using a combination of scattering techniques and optical measurements. Van der Burgt *et al*. used transmission SAXS to monitor the formation of perovskite supraparticles in solution (inside a quartz capillary) and were able to prove that the addition of methyl acetate triggered the formation of supraparticles over the course of several days.^684^

#### Summary and Outlook for X-ray Scattering Characterization of MHP NCs

X-ray scattering techniques are remarkably useful for analyzing crystal structure, degradation induced phase changes, preferential orientation, crystallinity, morphology, and superstructure of perovskite NCs. As advanced scattering techniques become better understood and availability increases, more and more focus is put on *in situ* GIXS measurements. Grazing-incidence geometry allows for analyzing statistically relevant sample volumes. With small- and wide-angle X-ray scattering (SAXS and WAXS), methods are available to probe length scales from the crystal to the mesoscale. X-ray scattering can be coupled to additional *in situ* compatible measurements, *e.g*., PL, trPL, or UV/vis. Thereby, a wide-ranging toolbox of techniques is available that allows for flexible and focused investigations of structure–function correlation, especially in the field of PV and LED, where optoelectronic properties are of key interest and often heavily influenced by structure.^334,754,755^ X-ray scattering techniques are being constantly improved and especially image processing and simulation of 2D scattering images from advanced scattering techniques will become increasingly available.^756−759^*In situ* investigations on deposition techniques well-fitted for industrial purposes, *e.g*., roll to roll processing, and coupling to advanced experiments for degradation and formation investigations might well be in the focus of future research. However, also more easily accessible X-ray diffraction routinely available at many groups can be greatly beneficial for perovskite NC studies. XRD measurements can be used to provide phase information, phase purity, lattice parameters and can give hints for superstructural arrangements and crystallinity. Thereby, X-ray scattering techniques will help perovskite NC systems to gain even more attention from the scientific community and become increasingly promising for exploring fundamental properties as well industrial applications.^747,760,761^

## Optical Properties

### Linear Absorption and Photoluminescence

MHPs have been known for their intriguing optical and electronic properties that are appealing for low-cost, high-performance optoelectronic devices. These include tunable photoluminescence across the entire visible spectrum, high-color purity, multicolor chromism, high absorption coefficients, high PLQY, and long charge carrier diffusion lengths.^13,762−764^ The band gap of MHPs is easily tunable over UV–vis–near-IR wavelengths by varying the halide compositions (X = I^–^, Br^–^, Cl^–^).^14,31,538,765−769^ They have been intensely explored in solar energy and light harvesting applications. MHP-based colloidal NCs exhibit high PLQY compared to classical, core-only quantum dots, suggesting the significant reduction of nonradiative loss channels prevalent in the corresponding bulk MHPs films. In the previous sections, we reviewed the shape and composition-controlled synthesis of perovskite NCs. In this section, we focus on their optical properties. We start by briefly reviewing the optical properties of bulk MHPs and then review how they change when the size of the crystals decreases to the nanoscale. We further discuss the phenomena which manifest only in NCs, such as quantum confinement. As discussed in other sections, the advances in the synthesis enable the preparation of MHP NCs with highly controlled size, shape and surface properties. These NCs provide a very convenient platform to study the optical properties of MHPs which are not specific only to nanoscale. In this context, we review the optical, spin and electronic properties of colloidal MHP NCs and how they can be used to reveal insights into the properties of their bulk counterparts.

#### Electronic Band Structure

**Figure 87:**
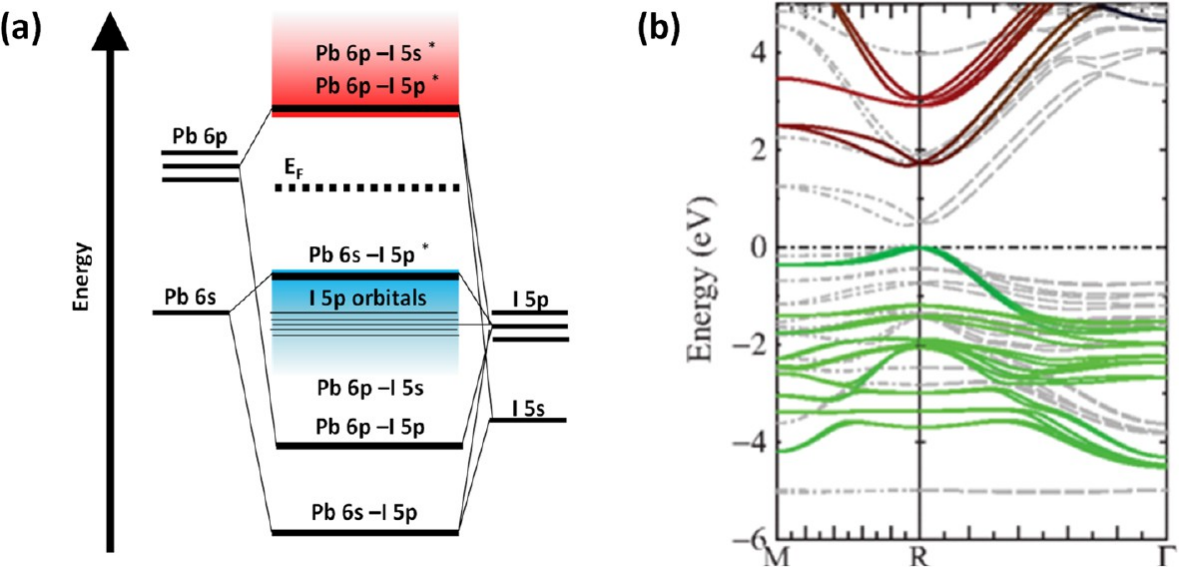
(a) Electronic bands formation in the case of MAPbI_3_ perovskites due to hybridization of Pb and I orbitals. Schematic is drawn according to ref (770). (b) Calculated energy band diagram of the 3D CH_3_NH_3_PbI_3_ perovskites under quasiparticle self-consistent GW approximation. Adapted with permission from ref (773). Copyright 2014 American Physical Society.

**Figure 88:**
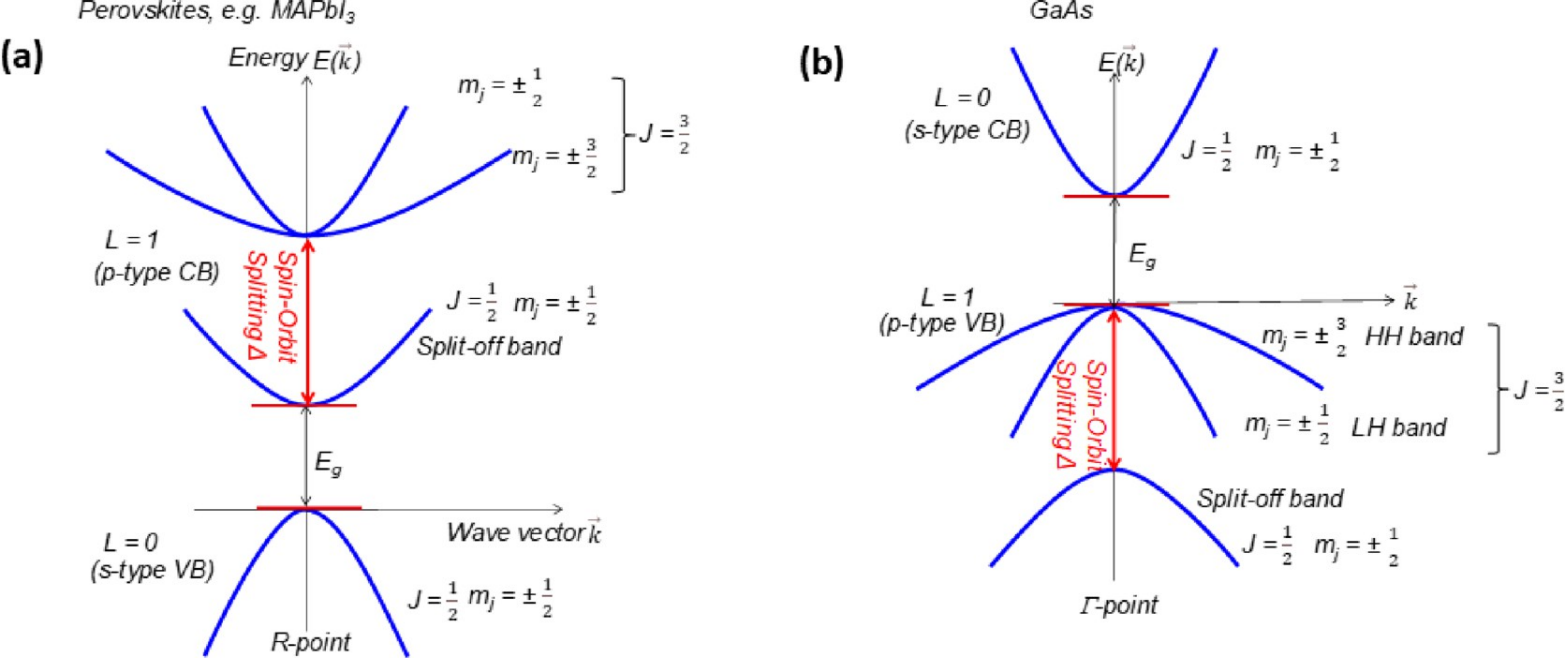
Schematic representation of the electronic band structure of (a) lead-based MHP and (b) GaAs. The VB of GaAs consists of heavy hole (HH) and light hole (LH) bands, along with a split-off band. On the contrary, MHP has an inverted band structure with the split-off band being the CB.

In lead-based MHP, the conduction band consists of σ-antibonding Pb 6p orbitals and halide np orbitals, hence possesses a p-type character (Figure 87a). The electronic configuration of Pb(II) is 6s^2^6p^0^, and it is np^6^ for halides (where *n* = 3–5 from Cl to I).^770,771^ The valence band in MHP is made of σ-antibonding Pb 6s and halide np orbitals, conferring the band a partial s-type character. In effect, the transition from the valence to the conduction band is dipole allowed.^772^Figure 87b shows the calculated electronic band structure of the 3D MAPbI_3_ perovskites under quasiparticle self-consistent GW approximation (QSGW).^773^ The color of the bands corresponds to their orbital characters where green, red and blue depicts I 5p, Pb 6p, and Pb 6s orbitals, respectively. M and R points are the zone-boundary points close to (1/2,1/2,0) and (1/2,1/2,1/2), respectively. MAPbI_3_ has a direct band gap with the CBM and VBM lying at the R point of the Brillouin zone. The VBM and CBM are shifted slightly from R as a consequence of spin–orbit coupling (SOC). As lead and iodine are heavy elements, SOC is large in MHPs and has a significant effect in their optical and electronic properties. Even *et al.* reported that the exclusion of SOC severely underestimates the band gap calculation in MHPs.^774^ Importantly, the SOC strongly influences the width of the band gap. Specifically, the optical band gaps in MAPbI_3_ and MAPbBr_3_ shrink by 0.5 and 0.8 eV, respectively, when SOC is taken into account as compared to the band gap calculation without considering SOC.^774^ Due to its p-type character, the conduction band is affected strongly due to SOC while the valence band remains nearly unaffected. This leads to two-fold degenerate split-off (SO) states representing the CBM in the lead-based MHPs (Figure 88a).^774^

The electronic band structure of MHP is inverted compared to the classical semiconductor such as GaAs (Figure 88b). In GaAs the CBM and VBM lie at the Γ point of the Brillouin zone where the CB is s-type with orbital angular momentum *L* = 0 and the VB is p-type with *L* = 1. The VB in GaAs consists of heavy hole (HH) band and light hole (LH) band with total spin angular momentum *J* = 3/2 and magnetic quantum number *m*_*j*_ = ±3/2 for HH and ±1/2 for LH. The split off band with *J* = 1/2 lies below the LH band separated by the spin–orbit coupling induced splitting (Δ). In the case of MHP, the VB is s-type, whereas the CB is p-type where the split off band (*J* = 1/2) represents the CB. Importantly, the valence and the conduction bands in MAPbI_3_ have high-energy dispersion in k-space which gives rise to small hole and electron effective masses. The small carrier effective masses are consistent with high mobilities and long carrier diffusion lengths in this material.^773^

#### Optical Band Gap

**Figure 89:**
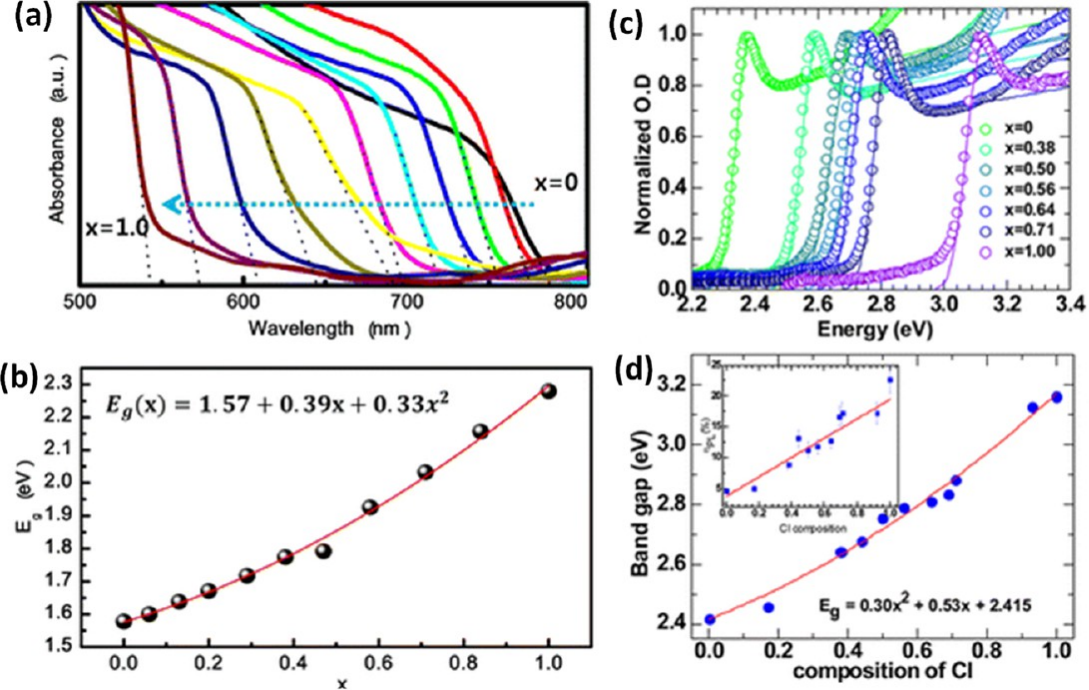
(a) Absorption spectra of FTO/bl-TiO_2_/mp-TiO_2_/ MAPb(I_1–*x*_Br_*x*_)_3_/Au cells, where the band gap shifts toward lower wavelengths with increasing Br substitution. (b) Composition-dependent band gap follows a quadratic relationship with respect to Br concentration (*x*). Adapted from ref (765). Copyright 2013 American Chemical Society. (c) Absorption spectra of MAPb(Br_1–*x*_Cl_*x*_)_3_ bulk thin films, where *x* varies from 0 (MAPbBr_3_) to 1 (in the case of MAPbCl_3_). The circles correspond to the experimental data, whereas the solid lines are simulated absorption spectra using the Sommerfeld model, considering the enhancement in the absorption coefficient by taking into account for the Coulomb field of the exciton. (d) Quadratic behavior of the band gap with Cl composition in the case of MAPb(Br_1–*x*_Cl_*x*_)_3_ thin films. Adapted from ref (768). Copyright 2015 American Chemical Society.

Since the top of the valence band in MHPs are dominated by the halide p orbitals (Figure 87b) with only minor contributions from antibonding Pb 6s^2^ orbitals, the valence band position becomes sensitive to the choice of halide ions. The band gap increases from I- to Br- to Cl-based MHPs. The increase in band gap is predominately driven by the downshift of valence band while the conduction band upshift is less pronounced.^771^ Noh *et al.* showed optical band gap tuning in mixed-halide MAPb(I_1–*x*_Br_*x*_)_3_ perovskites by changing the compositions of I and Br ions.^765^Figure 89a shows the corresponding experimental absorption spectra of mp-TiO_2_/MAPb(I_1–*x*_Br_*x*_)_3_ (0 ≤ *x* ≤ 1). The absorption onsets of mp-TiO_2/_MAPb(I_1–*x*_Br_*x*_)_3_ vary from 786 nm (1.58 eV) to 544 nm (2.28 eV), resulting in wide color tunability. The estimated band gaps from the absorption onsets were observed to follow a quadratic relationship with halide compositions (Figure 89b). The absorption spectra increases sharply at the optical band-edge consistent with a direct band gap with allowed transitions. While in the iodide, the excitonic contribution is not much pronounced, and it becomes prominent at the optical band-edge when moving from I- to Br- to Cl-based MHPs (Figure 89c). Kumawat *et al.* calculated the band gap in MAPb(Br_1–*x*_Cl_*x*_)_3_ by considering the effect of excitonic contribution at the band-edge using the Sommerfeld model. The band gap increases from 2.4 eV for MAPbBr_3_ to 3.1 eV for MAPbCl_3_.^768^ Similar to the case of I–Br-based mixed-halide MHPs, the band gap tuning in Br–Cl-based MHPs varies in a quadratic fashion with the Cl composition (Figure 89d). Similar to MA-based MHPs, FA- and Cs-based LHPs also exhibit similar trends of band gap tuning with the change in halide compositions.

It is important to note that A-site cations such as MA, FA, or Cs, do not contribute to the electronic band gap directly but can still influence the crystal structure *via* rotation of Pb-X-Pb bond angles and thus, indirectly modify the band gap.^775−777^ Beyond lead-based systems, there has been extensive work on MHPs based on Sn and Ge, as well as halide double perovskites and other perovskite-inspired materials (refer to NANOCRYSTALS OF LEAD-FREE PEROVSKITE-INSPIRED MATERIALS). The band gap of CsSnX_3_ perovskite is lower compared to the Pb^2+^ analogues due to higher electronegativity of Sn ions compared to Pb.^515,518^ Huang *et al.* showed that there is relatively small amount of change in the band gap from CsSnCl_3_ to CsSnI_3_ compared to Pb-based MHP, due to interatomic Sn s and Sn p character of the VBM and CBM.^519^ Unlike Pb^2+^-based MHP, lead-free double perovskites (DP) with stoichiometric formula A_2_B^I^B^III^X_6_;^183,499,778,779^ show weak photoluminescence due to indirect band gap or parity-forbidden direct transitions. Using DFT calculations, Meng *et al.* predicted that, out of nine possible DP, six of them show parity-forbidden direct band gap transitions.^778^

#### Band Gap Excitation

**Figure 90:**
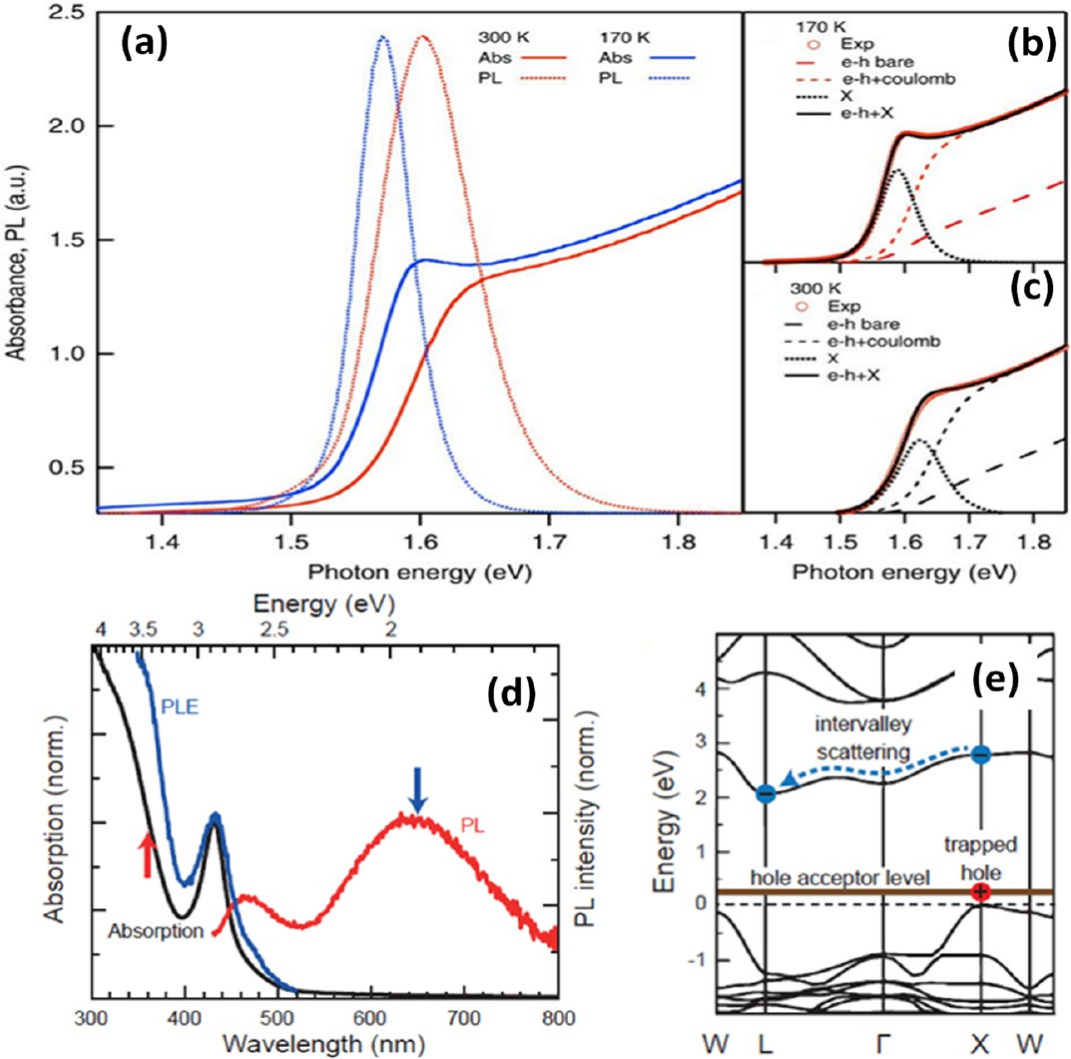
(a) Absorption (continuous line) and PL (dashed line) spectra in MAPbI_3_ films, recorded at 300 and 170 K. Elliot fits to the experimental absorption spectra at (b) 170 and (c) 300 K. Excitonic and band to band oscillator strength contributions calculated by taking into account Coulomb interaction according to Elliott’s theory of Wannier excitons are shown in (b) and (c). The excitonic contribution is enhanced at low temperature. Adapted with permission from ref (780). Copyright 2014 Nature Publishing Group, a division of Macmillan Publishers Limited. All rights reserved. (d) Linear absorption, PL, and PLE (detected at 650 nm) spectra of DP NCs dispersed in toluene. (e) Electronic band structure of Cs_2_AgBiBr_6_ double perovskite. The blue and red circles represent the electron and trapped hole, where the brown solid line indicates the hole acceptor level. Adapted from ref (779). Copyright 2020 American Chemical Society.

As observed in Figure 89c, the excitonic transitions at the band gap in MHPs imply considerable Coulomb interactions between the electrons and holes. Therefore, the absorption coefficient does not simply follow the square root dependence as in the case of free electrons and holes. Instead, there is an additional contribution from Sommerfeld enhancement above the band-edge and excitonic transitions below. Therefore, it is appropriate to deconvolute excitonic *versus* continuum transition probabilities.^768,780^ In the case of bulk MHPs, Saba *et al.* used the Elliot theory of Wannier–Mott excitons to model the measured absorption spectra.^780^Figure 90a shows the linear absorption and emission spectra of MAPbI_3_ thin films at 300 and 170 K, respectively, as measured by Saba *et al*.^780^ The excitonic *versus* continuum contributions have been separated out using Elliot model. It was noticed that at lower temperatures the excitonic contribution increases in MHPs (Figure 90b,c).

Similarly to Pb^2+^-based MHP, the optical band-edge of lead-free double perovskites is also dominated by sharp absorption resonance (Figure 90d).^779^ This optical resonance has been assigned to self-trapped exciton in the case of Cs_2_AgInCl_6_.^499^ In the case of Cs_2_AgBiBr_6_ double perovskites, Dey *et al.* explained the origin of this sharp optical resonance with the help of the electronic band structure (Figure 90e).^779^ It was demonstrated that it is unlikely that the high effective mass electron along with the low effective mass hole at the direct band gap could lead to a bound state with strong binding energy, because the reduced mass of the electron–hole pair would have been small in such case. Considering the effect of hole trapping by Ag vacancies, they concluded that the bound hole along with the high effective mass electron could lead to a defect bound exciton at the direct band gap. Consequently, the high-energy PL emission close to the optical resonance (Figure 90d) was assigned to the radiative recombination of these direct bound excitons due to their giant oscillator strength. This was corroborated by theoretical calculations in which using ground- and excited-state *ab initio* methods, Palummo *et al.* showed that the first absorption peak in Cs_2_AgBiBr_6_ and Cs_2_In_2_X_6_ is consistent with bound excitons.^781^

**Figure 91:**
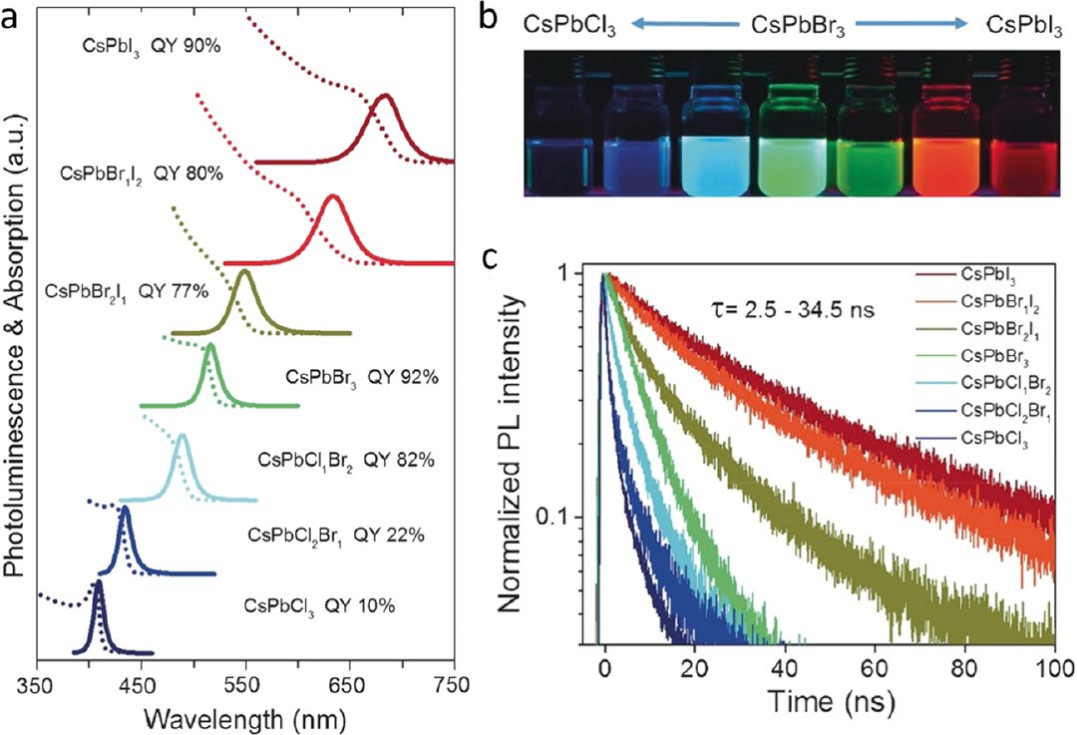
(a) Linear absorption and PL spectra and the corresponding PLQYs of CsPbX_3_ NCs of different halide compositions synthesized by ultrasonication approach. (b) Photograph of the corresponding colloidal dispersions in hexane under UV light. (c) PL decay traces of the corresponding NCs. Adapted with permission from ref (30). Copyright 2016 John Wiley & Sons, Inc.

Bulk MHPs typically exhibit weak PLQY limiting their light-emitting applications. Crucially, this property is radically changed when moving from bulk to nanocrystals underscoring the effect of the crystal size and interface composition on the optical properties of MHP. Specifically, it has been shown that reducing the crystal size to nanoscale leads to a significant improvement in PLQY.^14,25,29,30,169^ Since the early report of highly luminescent (PLQY ∼80%) green emissive MAPbBr_3_ colloidal crystals,^25^ significant research efforts have been devoted to the development of colloidal MHP NCs made of different cation and anion compositions with improved optical properties regarding their stability, PL tunability, PLQY (discussed in previous sections). Similar to their bulk counterparts, the optical band gap and PL emission in colloidal MHP NCs is easily tunable across the visible region of the electromagnetic spectrum by varying the halide composition.^14,30^ For example, colloidal CsPbX_3_ NCs synthesized by ultrasonication approach exhibit extremely high PLQYs and tunable emission between 400 and 680 nm by just varying the halide composition (Figure 91a,b). Br- and I-based MHP NCs exhibit near-unity PLQY under optimized synthesis conditions, while the Cl-based MHPs exhibit lower PLQY.^14,30^ The low PLQY of Cl-based perovskites has been attributed to the halide vacancies acting as nonradiative traps. PL decay gets faster going from iodide *via* bromide to chloride-based CPbX_3_ perovskite NCs (Figure 91c). The faster PL decay time and low PLQY in the case of Cl-based NCs suggest that they exhibit higher nonradiative rates as compared to the iodide and bromine-based NCs. Nevertheless, it has been shown recently that the PLQY in these perovskites can also be dramatically improved to near-unity by doping with metal halides such as CuCl_2_ and MgCl_2_.^782,783^ The origin of the high PLQYs of the colloidal MHPs with respect to the bulk material is still an intensively investigated subject.^147,784^ It is postulated that the increased surface to volume ratio and effective surface passivation with ligand molecules, and thereby a removal of surface traps, causes the increased PLQY of colloidal MHP NCs as compared to their bulk counterparts. A recent study suggested that increased oscillator strength in MHP NCs results in enhanced PLQY when their morphology is tuned from bulk to nanoscale.^784^

#### Quantum-Confinement Effect on Optical Band Gap

Another important consequence of the reduction in size of the MHP crystals is the manifestation of quantum-confinement effects. Three size ranges may be delimited: (i) when the size is much larger than the exciton Bohr radius (*d* ≫ *a*_B_), so that the confinement effects are negligible, (ii) weak confinement regime when the size is comparable with the exciton Bohr radius, and (iii) strong confinement regime when the exciton Bohr radius is larger than the NC (*a*_B_ ≫ *d*). Interestingly, the average size distribution of typical colloidal MHP NCs is ∼10 ± 1 nm which falls under the weak confinement regime where the effect on the band gap is small. Nonetheless, in the strong confinement regime provides a means to effectively tune the band gap in MHPs.^16,60,138,150,785,786^ In the strong quantum-confinement regime, the electron and the hole should be viewed as independent particles and their confinement energies needs to be calculated first before taking into account their Coulomb interaction.^787^

**Figure 92:**
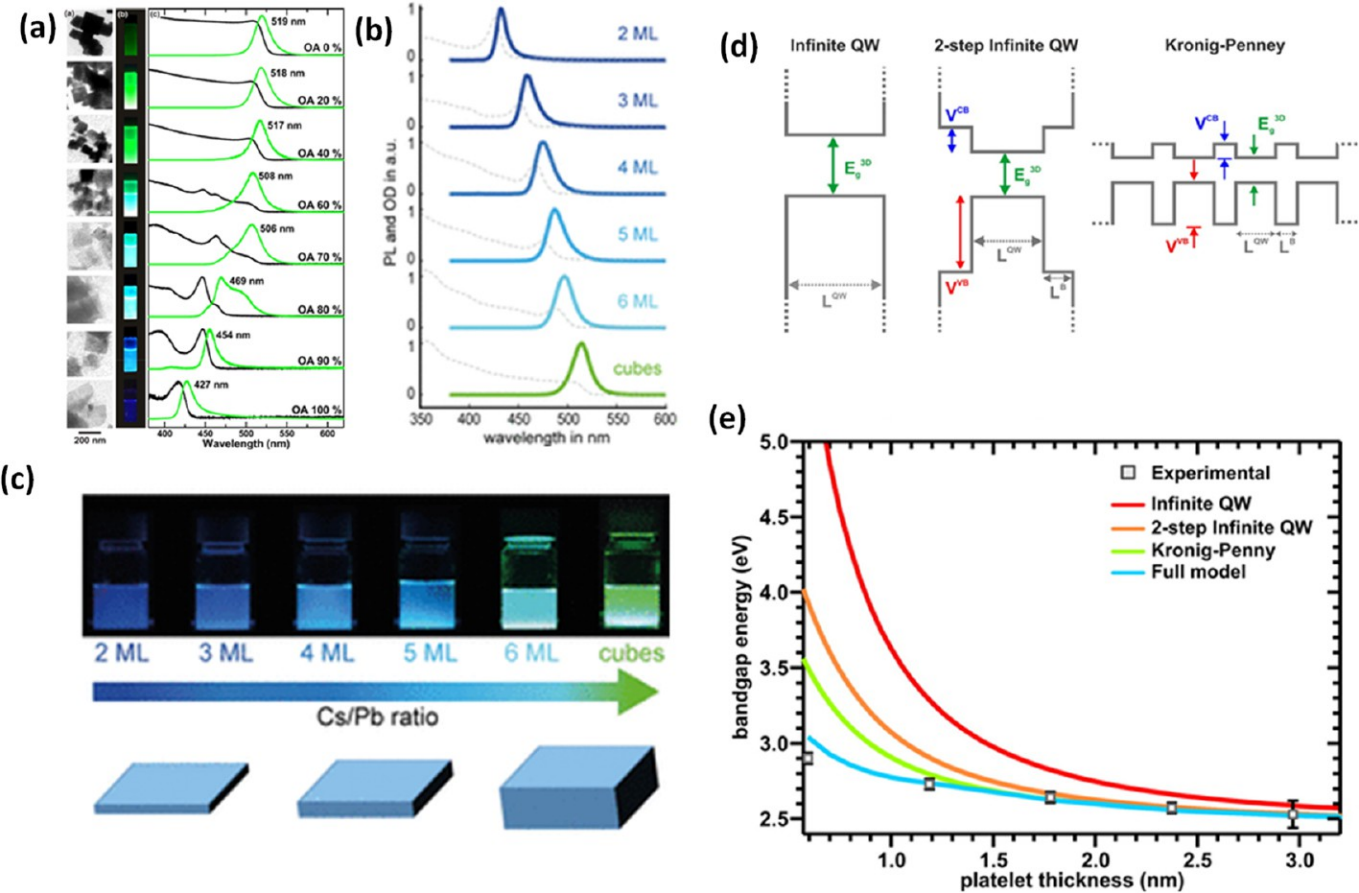
(a) TEM images, pictures of NPl solutions, linear absorption, and PL spectra of MAPbBr_3_ NPls. Adapted from ref (16). Copyright 2015 American Chemical Society. (b) Linear absorption and PL spectra of CsPbBr_3_ NPls with varying thicknesses. (c) Pictures of CsPbBr_3_ NPls and cubes dispersed in hexane under UV-light exposure. The emission wavelength red shifts with increase in monolayer thickness. Panels b and c are adapted from ref (60). Copyright 2018 American Chemical Society. (d) Quantum well models used to reproduce the experimental spectra, as shown in c. (e) Calculation of the energy of perovskite nanoplatelets as a function of platelet thickness (solid lines) and the experimentally determined values (gray squares). Panels d and e were adapted from ref (16). Copyright 2015 American Chemical Society.

Colloidal 2D perovskite nanoplatelets have been greatly explored to understand the quantum-confinement effects in MHPs (refer to Nanoplatelets section for detailed discussion). Sichert *et al.* demonstrated and modeled the two-dimensional quantization behavior and excitonic effects in MHP NPls based on MAPbBr_3_ perovskites (Figure 92a).^16^ Later, Bohn *et al.* showed precise control over the thickness of CsPbBr_3_ perovskite NPls by varying it, from 2 to 6 monolayers (Figure 92b).^60^ The colloidal NPls exhibit sharp optical transitions at the band-edge due to strong quantum-confinement effect. Hence, in 2D perovskite NPls the exciton binding energy enhanced compared to 3D nanocubes.^16,60^ The exciton binding energy in CsPbBr_3_ increases from 30 to 280 meV when their dimension changes from 3D nanocubes to 2D NPls with 2 monolayer thickness (Figure 92c).^60^

Sichert *et al.* demonstrated that the simple consideration of an infinite quantum well model (Figure 92d) overestimates the quantization energy compared to the experimentally determined values under the assumption of infinite confinement energy.^16^ They successfully modeled the band gap energies for NPls with monolayer numbers (*n*) = 3, 4, 5, under the approximation of a one-band effective-mass Kronig–Penny model. They observed for thinner NPls (Figure 92e), such as *n* = 2 and *n* = 1 where the exciton binding is very high, the discrepancies between theory and experiment are quite large.^16^ In the case of bulk perovskites with low dielectric constant, Coulomb screening dominates, leading to a reduction of the exciton binding energy. In the case of extremely thin NPls, most of the electric field lines between electron and hole are outside of the platelets where the dielectric constant is low compared to that of the semiconductor platelets, and this minimizes the Coulomb screening and thus enhances the exciton binding energy, accounting for the results obtained for extremely thin platelets.^16^

##### Effect of Dielectric Confinement on Low-Dimensional MHPs

**Figure 93:**
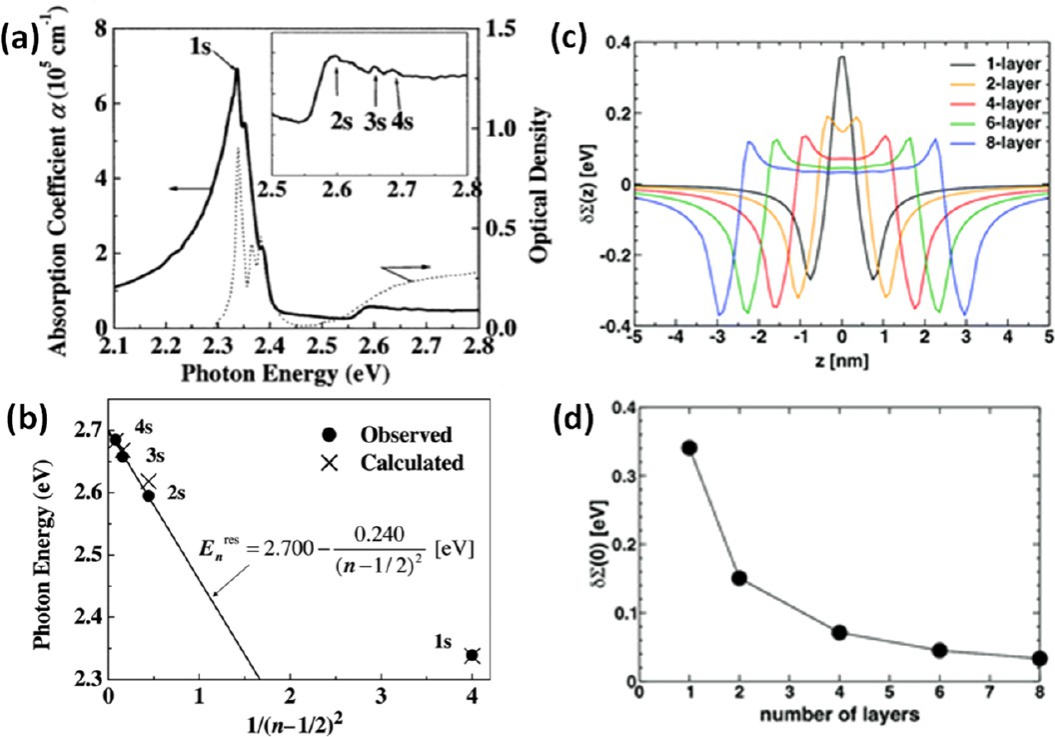
(a) The solid line shows the optical absorption spectrum of a (C_6_H_13_NH_3_)_2_PbI_4_ single crystal at 5 K obtained from the Kramers–Kronig transformation of its reflection spectrum. The inset shows the expansion around 2.6 eV. Dotted line shows the optical absorption spectrum of (C_6_H_13_NH_3_)_2_PbI_4_ polycrystalline film measured at 5 K. (b) Resonance energies of Wannier series excitons in (C_6_H_13_NH_3_)_2_PbI_4_ as a function of 1/(*N* – 1/2)^2^. Closed circles and crosses represent the observed and calculated energies of the excitons, respectively. The solid line shows the fitting based on a simple two-dimensional Wannier exciton model. Panels a and b are adapted with permission from ref (788). Copyright 2005 American Physical Society. (c) Self-energy profile δ∑(*z*) for slabs of CH_3_NH_3_PbI_3_. (d) Self-energy taken at the slab center δ∑(0). Panels c and d are adapted with permission from ref (789). Copyright 2016 Royal Society of Chemistry.

When charge carriers are confined in low dimensional multilayer halide perovskites, their self-energy could be further enhanced by the surrounding polarizability of the perovskite lattice arising due to dielectric inhomogeneity.^789−791^ This effect influences the electron–hole interaction energy, thus giving rise to a strong exciton resonance (Figure 93a). In the case of (C_6_H_13_NH_3_)_2_PbI_4_ crystals^788^ and for bromide compounds (C_4_H_9_NH_3_)_2_PbBr_4_ and (C_6_H_5_C_2_H_4_NH_3_)_2_PbBr_4_),^792^ it was shown that the exciton resonance spectra deviated from the well-known 2D-hydrogen-like series due to the dielectric confinement effect (Figure 93b). Sapori *et al*. calculated the self-energy profile for MAPbI_3_ nanoplatelets (Figure 93c).^789^ The self-energy is equivalent to one-particle electrostatic potential profile acting on a charge carrier in layered heterostructures and calculated by solving the inhomogeneous Poisson equation. The self-energy value is higher at the center of the slab for lower thicknesses due to dielectric confinement effects (Figure 93d).^789^

##### Effect of Temperature on Optical Transitions

**Figure 94:**
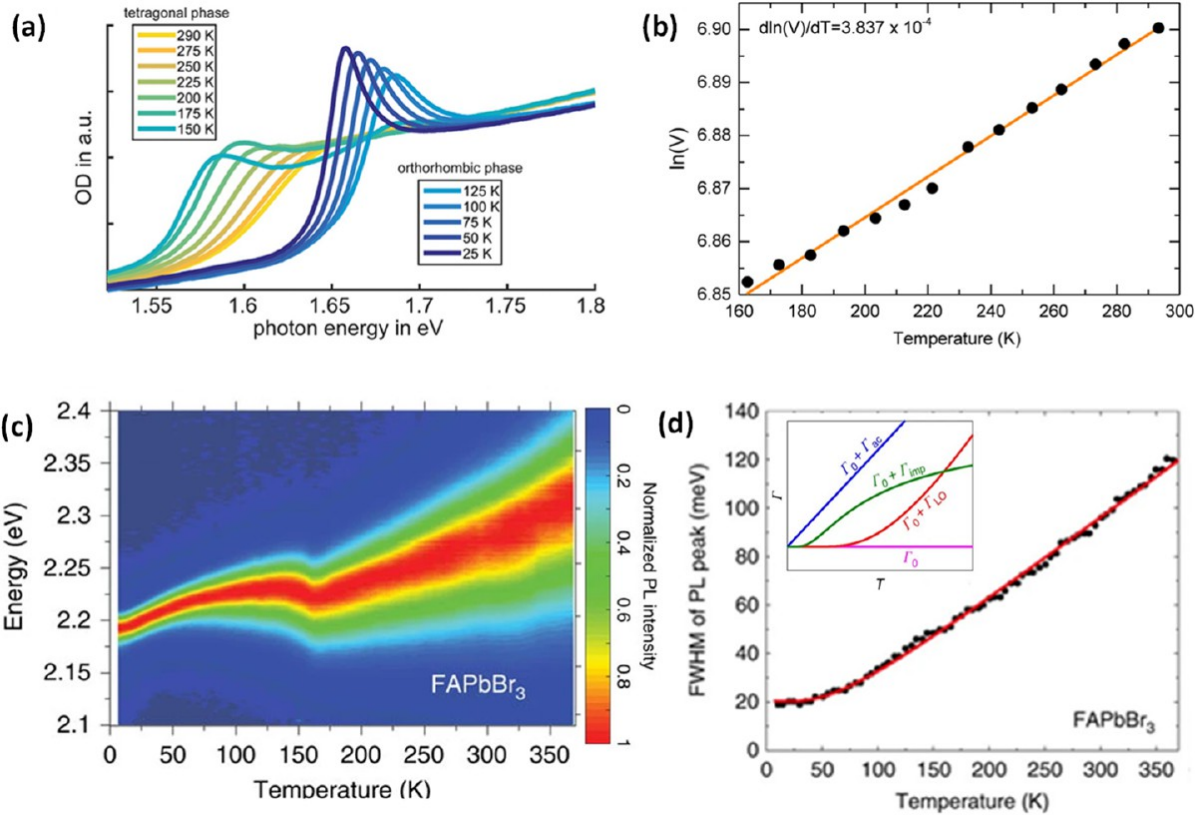
(a) Temperature-dependent absorption spectra on CH_3_NH_3_PbI_3_ nanoplatelets for temperatures of 25 to 290 K. The 1s exciton transition is prominent at low temperature. Adapted from ref (793). Copyright 2017 American Chemical Society. (b) Ln of the volume of CH_3_NH_3_PbI_3_ in tetragonal phase as a function of temperature. Solid line is a linear fit representing positive coefficient of thermal expansion. Adapted from ref (794). Copyright 2016 American Chemical Society. (c) Color plot for the normalized steady-state PL spectra of FAPbBr_3_ thin film between a temperature range of 10 to 370 K. (d) Corresponding fwhm of the steady-state PL spectra. Panels c and d are adapted with permission under a Creative Commons CC BY license from ref (795). Copyright 2016 The Authors.

Unlike most conventional semiconductors (*e.g.*, GaAs, GaN, or Si), MHPs show a blue shift in the band gap with increasing temperature (Figure 94a).^780,794−796^ MAPbI_3_ undergoes a phase transition from the tetragonal to the orthorhombic phase at temperatures below 163 K.^793,794,796^ In both phases, the band gap increases with increasing temperature due to a large coefficient of thermal expansion, which results in a positive temperature coefficient of the band gap (Figure 94b).^794^ Singh *et al.* showed that in the case of MAPbI_3_, lattice dilation plays a more significant role compared to electron–phonon coupling.^794^ They determined the volume expansion coefficient of CH_3_NH_3_PbI_3_ to be (1.35 ± 0.014) × 10^–4^ K^–1^ which is 50 times higher than crystalline Si. In contrast to MAPbI_3_ and MAPbBr_3_, MAPbCl_3_ shows a decrease in the band gap with increasing temperature because electron–phonon coupling dominates over the effects of lattice dilation.^797^ In MHPs, electron–phonon coupling is found to be very strong, in which Fröhlich interactions between carriers and optical phonons are the dominant source of electron–phonon coupling.^795,798^ In the case of vacancy ordered halide perovskites single crystals, such as Cs_3_Bi_2_I_9_, Cs_3_Sb_2_I_9_, and Rb_3_Bi_2_I_9_, the emission process has been explained with self-trapped excitons which arise due to strong electron–phonon coupling inducing the formation of small polarons.^541^

**Figure 95:**
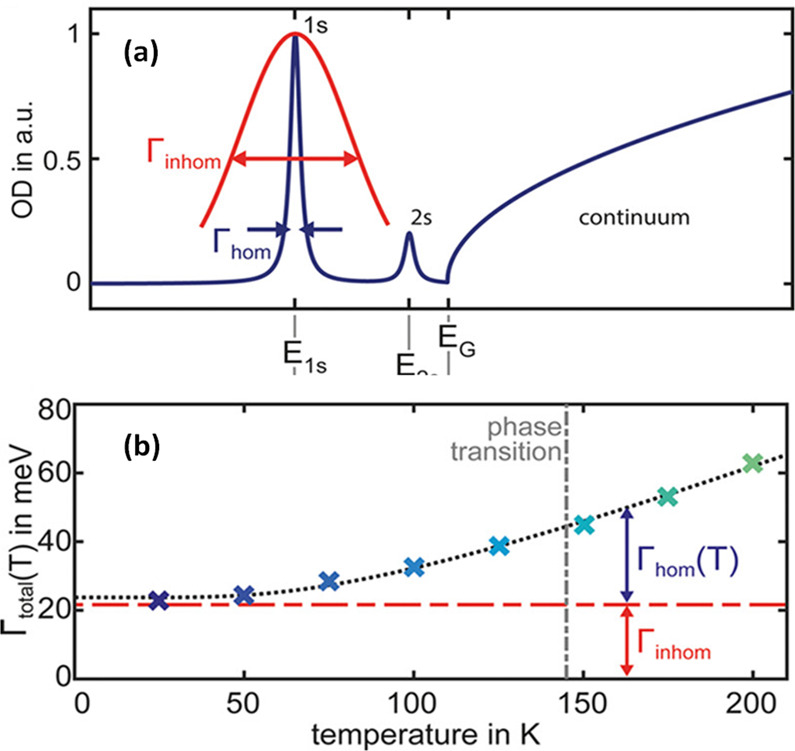
(a) Schematic of the absorption spectrum of one single bulk-like NPl where the homogeneous broadening of the Lorentzian-shaped excitonic peaks is given by Γ_homo_ (dark blue). The excitonic levels (1s, 2s) and the continuum onset are well-separated and easily distinguishable. (b) Total exciton line broadening of the 1s exciton state Γ_total_ (=Γ_homo_ + Γ_inhomo_) as a function of temperature *T* (<200 K), where the dotted line represents the fitting of the theoretical model considering LO phonons. The Γ_homo_ has been calculated using the exciton dephasing time (*T*_2_) as Γ_homo_ = 2*h*/*T*_2_. Adapted from ref (793). Copyright 2018 American Chemical Society.

In MHP, charge carrier scattering with longitudinal optical (LO) phonons has been shown to cause the broadening of the excitonic absorption^793^ and photoluminescence peaks (Figure 94d).^795^ In general, impurities or (in the case of NCs) polydispersity can cause the exciton line width to further broaden inhomogeneously (Γ_inhomo_), as shown in Figure 95a.^793^ Γ_inhomo_ is a temperature independent quantity whereas Γ_homo_ depends on temperature (Figure 95b). Bohn *et al.* determined the homogeneous line broadening in the case of MAPbI_3_ nanoplatelets using temperature-dependent transient four wave mixing (FWM).^793^ They determined an exciton dephasing time (*T*_2_) ∼ 800 ± 20 fs for the 1s exciton in MAPbI_3_ nanoplatelets at 25 K, giving rise to Γ_homo_= 2ℏ/*T*_2_ = 1.7 ± 0.1 meV and Γ_inhomo_ = 22 ± 1 meV.^793^

##### Stokes Shift

**Figure 96:**
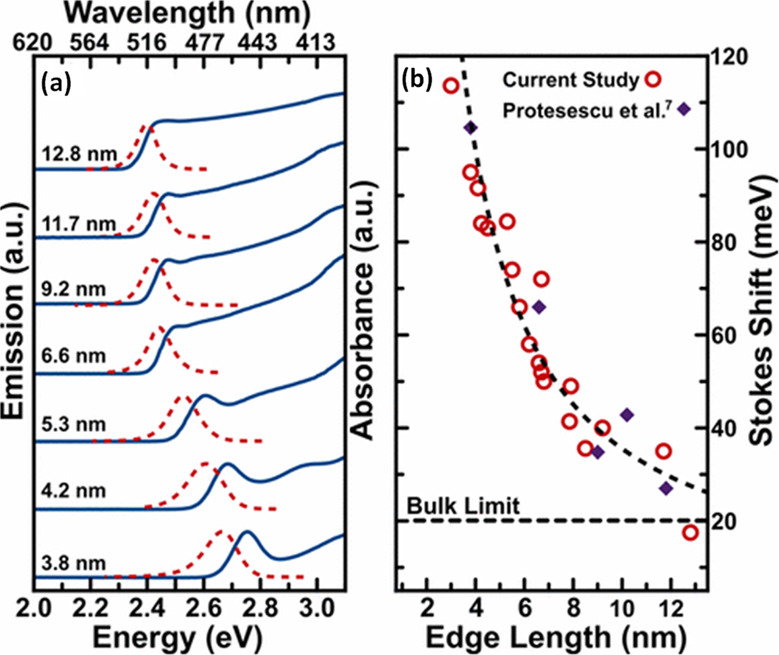
(a) CsPbBr_3_ NCs ensemble absorption (solid blue lines) and emission (dashed red lines, *E*_exc_ = 3.543 eV, λ_exc_ = 350 nm) spectra for a series of varying sizes. All absorption/emission spectral pairs are offset for clarity. (b) Corresponding size-dependent Stokes shifts and those extracted from existing literature. Adapted from ref (785). Copyright 2017 American Chemical Society.

Quantum-confined (QC) MHP NCs exhibit blue-shifted emission compared to their 3D bulk counterparts. For instance, strongly quantum-confined CsPbBr_3_ NCs emit blue photoluminescence, while their 3D counterparts emit green photoluminescence. Brennan *et al.* synthesized CsPbBr_3_ nanocubes with size distribution ranging from 13 to 4 nm.^785^ They found that the Stokes shift for CsPbBr_3_ nanocubes increased with increasing quantum confinement (or decreasing size).^785^ The Stokes shift was found to decrease from 82 to 20 meV for the CsPbBr_3_ nanocubes as the size increased from 4 to 13 nm (Figure 96a,b). The size-dependent Stokes shift has been explained by the confinement of the hole state.^785^ For double perovskites, vacancy-ordered halide perovskites, and inorganic zero dimensional tin-halide perovskites Cs_4–*x*_A_*x*_Sn(Br_1–*y*_I_*y*_)_6_ (A = Rb, K; *x* ≤ 1, *y* ≤ 1), the long-lived emission was strongly Stokes-shifted and have been assigned to the formation of self-trapped excitons.^541,799,800^

Similarly to the band gap width, the Stokes shift for MAPbBr_3_ and CsPbBr_3_ single crystals has been found to be highly temperature-dependent, as shown by Guo *et al.*(801) They have observed that the luminescence Stokes shifts for MAPbBr_3_ and CsPbBr_3_ single crystals increased with increasing temperature between 60 and 300 K range. However, below 50 K, the luminescence Stokes shift weakly depended on temperature and decreased as the temperature increased.^801^ This temperature-dependent Stokes shift was explained in terms of a classical Debye-like relaxation process of the dielectric response function originating from the anharmonicity of the LO (longitudinal-optical) phonons at about 160 cm^–1^ in the lead bromide sublattice.

##### Exciton Fine Structure

Excitons are the central emitting species in the semiconductor nanostructures that appear as additional (sometimes sharp) optical transitions at the optical band-edge.^802−804^ The degeneracies of the lowest exciton states are broken by strong exchange interactions, spin–orbit coupling, intrinsic crystal field; nanostructures shape anisotropy giving rise to the multiple splitting of the lowest exciton states known as exciton fine structure.^803,805−808^ In almost all bulk III–V semiconductors heterostructures, II–VI core–shell colloidal nanostructures, the lowest available exciton states are found to be optically inactive, also known as dark exciton.^809−811^ In the case of bulk semiconductor the splitting between the bright (optically active) and the dark (optically inactive) exciton is very small, generally less than the thermal energy even at cryogenic temperature. Hence, the photoluminescence decay is not strongly affected by temperature.^802,803^ Nevertheless, the energy separation between them is increased up to tens of meV in the case of semiconductor nanostructures where the photoluminescence decay is prolonged at low temperature due to acoustic phonon-mediated relaxation of the bright exciton to the dark exciton states.^809,811−813^

**Figure 97:**
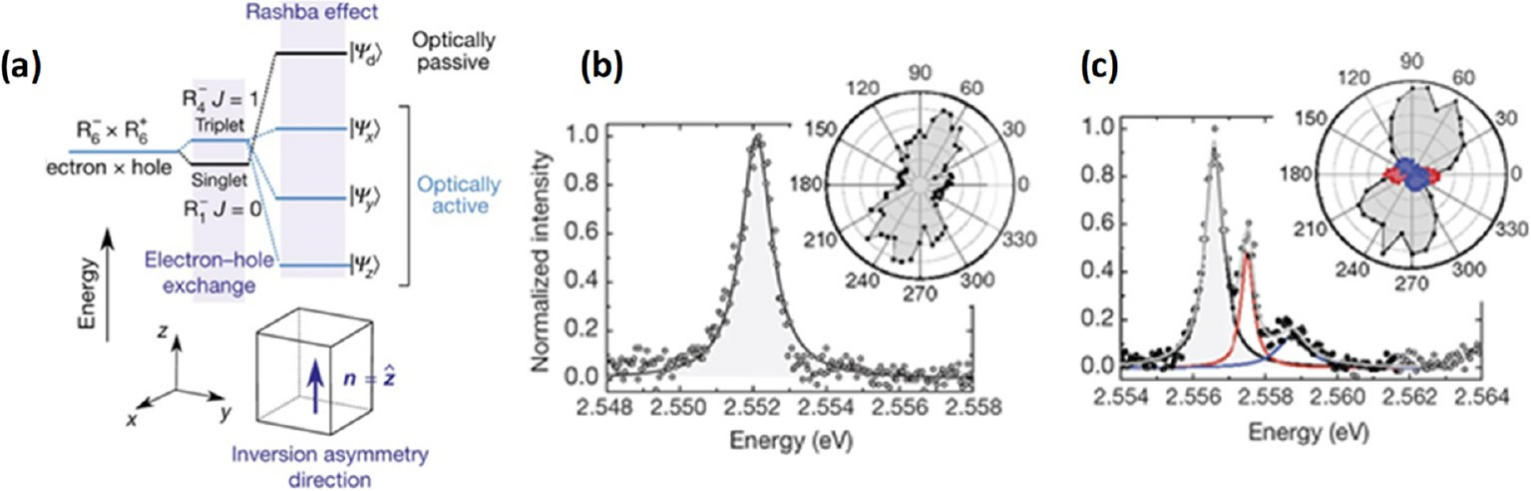
(a) Fine structure of the band-edge exciton considering short-range electron–hole exchange (middle) and then including the Rashba effect (right) under orthorhombic symmetry. The latter splits the exciton into three bright states with transition dipoles oriented along the orthorhombic symmetry axes (labeled *x*, *y*, and *z*) and a higher-energy dark state (labeled “d”). The energetic order of the three lowest sublevels is determined by the orthorhombic distortion. Photoluminescence spectra from individual NCs showing (b) one and (c) three photoluminescence peaks. Adapted with permission from ref (147). Copyright 2018 Macmillan Publishers Limited, part of Springer Nature. All rights reserved.

In almost all MHPs, it is observed that the photoluminescence decay becomes faster at low temperature where the photoluminescence quantum yield still remains high.^147,814^ The explanation of the high radiative recombination in Pb^2+^-halide perovskites at low temperature was proposed by Becker *et al*.^147^ They claimed the lowest exciton state as the bright triplet state for CsPbX_3_ (X = Br, Cl, I) crystals structure arising due to combination of strong spin–orbit coupling with Rashba effect.^806^ According to them,^147^ if only short-range electron–hole exchange interaction is taken into account then the singlet state lies below the triplet state, making the lowest available exciton state dark (Figure 97a). They showed that inclusion of Rashba effect leads to the alteration of the bright and dark exciton levels in CsPbX_3_ NCs. If the effective Rashba field is parallel to one of the orthorhombic symmetry axes of the CsPbX_3_ NCs the bright triplet exciton states split into three linearly polarized sublevels (Figure 97b,c).^147^ In a detailed study by Sercel *et al.* they demonstrated that the ground state of the perovskite nanostructure is indeed optically inactive (dark) like any other classical semiconductor quantum dots, if only exchange interaction has been considered.^806^ However, the experimentally observed bright exciton level order in tetragonal CsPbBr_3_ NCs can be explained including the contribution of the Rashba effect, which supports the theory by Becker *et al*.^147^ Moreover, it was shown that the bright–dark state positions could be reversed in low dimensional nanostructures, which, consequently, possess a dark exciton ground state.^806^ Opposite experimental observations were found in the case of CsPbBr_3_, FAPbBr_3_, and FAPbI_3_ NCs.^760,815,816^

**Figure 98:**
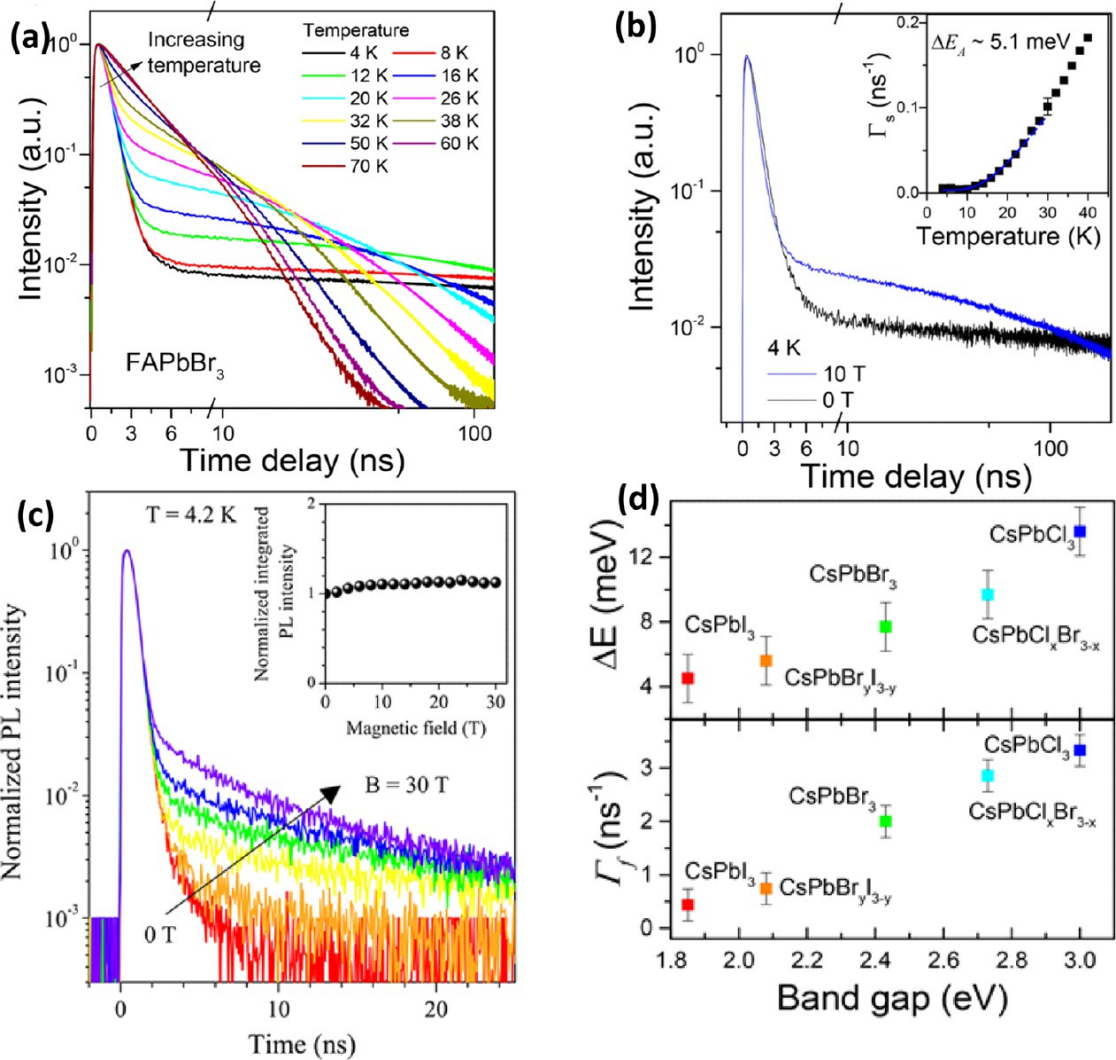
(a) Temperature-dependent PL decay traces in FAPbBr_3_ NCs. (b) PL decay curves recorded from FAPbBr_3_ nanocrystals at 4 K with and without an applied external magnetic field of 10 T. Inset shows the temperature-dependent relaxation rate of the slow decay component analyzed with the three-level model yielding a bright dark energy splitting of 5.1 meV. Adapted from ref (815). Copyright 2018 American Chemical Society. (c) PL decay recorded at cryogenic temperature for CsPbBr_3_ nanocrystals at varying magnetic fields. The inset shows the integrated PL intensity with respect to magnetic field. Adapted from ref (760). Copyright 2017 American Chemical Society. (d) Anion composition dependence of the bright–dark energy splitting (Δ*E*) and the decay rate of the fast component (Γ_f_). The values are plotted *vs* the band gaps of different nanocrystals at room temperature. Adapted from refs (760) and (815). Copyright 2017 and 2018 American Chemical Society.

Figure 98a shows the temperature-dependent PL decay in FAPbBr_3_ NCs. At 4 K, PL decay is biexponential with a dominant fast decay component, followed by a slow component. With rise in temperature the slow components grows gradually and becomes more prominent at higher temperature. Similar observation was also noticed in the case of CsPbX_3_ NCs.^760,815,817^ The first component is assigned to the radiative decay of the bright exciton whereas the slow component is assigned to the dark exciton decay, the rate of which is increases at higher temperature, as shown in the inset of Figure 98b.^815^ The lengthening of the dark exciton relaxation rate at higher temperature is due to thermal activation.^760,815^Figure 98b also shows that, under the application of an external magnetic field of 10 T, the amplitude of the slow decay component enhances which is due to dark exciton states mixing with the bright exciton states resulting into the brightening of the dark excitons.^760,811,815^ Similar observation was also noticed by Canneson *et al.*(760) in CsPbBr_3_ NCs, as shown in Figure 98c, where, with increase in magnetic field, the amplitude of the slow component enhances due to magnetic field-induced brightening of the dark exciton states. Using a three-level model for bright and dark excitons, Chen *et al.*(815) determined the energy splitting (Δ*E*) between the bright and dark exciton states, as depicted in Figure 98d. Δ*E* increases while going from CsPbI_3_ to CsPbCl_3_. It depends not only on anion composition but also on A-site cation composition.^815^ It has been also demonstrated that an external dopant like Mn^2+^ is able to manipulate the dark and bright exciton mixing.^817^ In the case of CsPbCl_3_ NCs, a similar brightening of the dark exciton states has been observed upon Mn^2+^ doping.^817^ The amplitude of the slow decay component at the cryogenic temperature was enhanced 5–10 times in Mn^2+^ CsPbCl_3_ NCs as compared to the undoped CsPbCl_3_ NCs.^817^

A direct observation of dark exciton emission in FAPbBr_3_ NCs has been reported by Tamarat *et al.*(816) Using magneto-optical studies at cryogenic temperature, they observed that a low-energy zero-phonon line appears at 2–2.8 meV below the zero-phonon line of bright triplet state at 7 T, which has been ascribed to dark singlet exciton state resulting due to the mixing of dark states with neighboring bright states. Similar to Chen *et al.*, they also observed magnetic field induced brightening of the dark singlet state at low temperature.^816^

**Figure 99:**
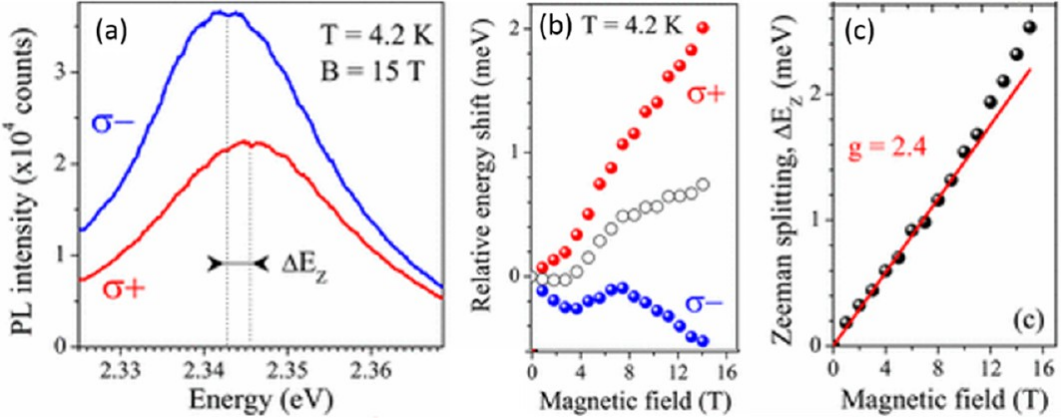
(a) Time-integrated polarization-resolved PL spectra of CsPbBr_3_ NCs at *B* = 15 T and *T* = 4.2 K. The spectra are split by the Zeeman splitting, Δ*E*_Z_. (b) Magnetic field dependence of the relative spectral positions of σ^–^ (open blue circles) and σ^+^ (red circles) components. Open circles show the shift of the center of gravity of the two polarized components, corresponding to the exciton shift without contribution of the Zeeman splitting. (c) Zeeman splitting of emission line. Red line is a linear fit with |*g*| = 2.4. Adapted from ref (760). Copyright 2017 American Chemical Society.

At cryogenic temperature, the bright excitons can further split into narrow spectral lines.^147,818,819^ Yin *et al.* had shown in CsPbI_3_ NCs the bright exciton split into two linear orthogonal polarized emission with energy separation of few hundred μeV.^819^ They also showed that in photocharged CsPbI_3_ nanocrystal the doublet emission of bright exciton switched to a single emission peak due to elimination of electron–hole exchange interaction.^819^ In the case of CsPbBr_3_ NCs Fu *et al.* found two different kind of fine structure splitting of bright exciton for orthorhombic and tetragonal phases.^818^ Under application of external magnetic field of 15 T Canneson *et al.* observed the bright exciton emission from CsPbBr_3_ NCs, to be circularly polarized where the left handed circularly polarized light was more intense compared to right handed circularly polarized light (Figure 99a-b).^760^ With increase in magnetic field the splitting between two opposite circularly polarized light enhances due to increased amount of Zeeman splitting from which they could determine the exciton g factor for CsPbBr_3_ NCs to be 2.4 with electron and hole *g* factors of +2.18 and −0.22, respectively (Figure 99c).^760^

##### Spin-Polarization of Optically Generated Carriers

The exciton spin that determines the singlet/triplet dark/bright character of the states plays a critical role in controlling the optical transitions in semiconductor NCs. For instance, a spin-flip may cause a transition from an optically active (bright) to a passive (dark) state. Therefore, selective excitation of exciton spin is an effective approach to tune the optical properties of NCs.

**Figure 100:**
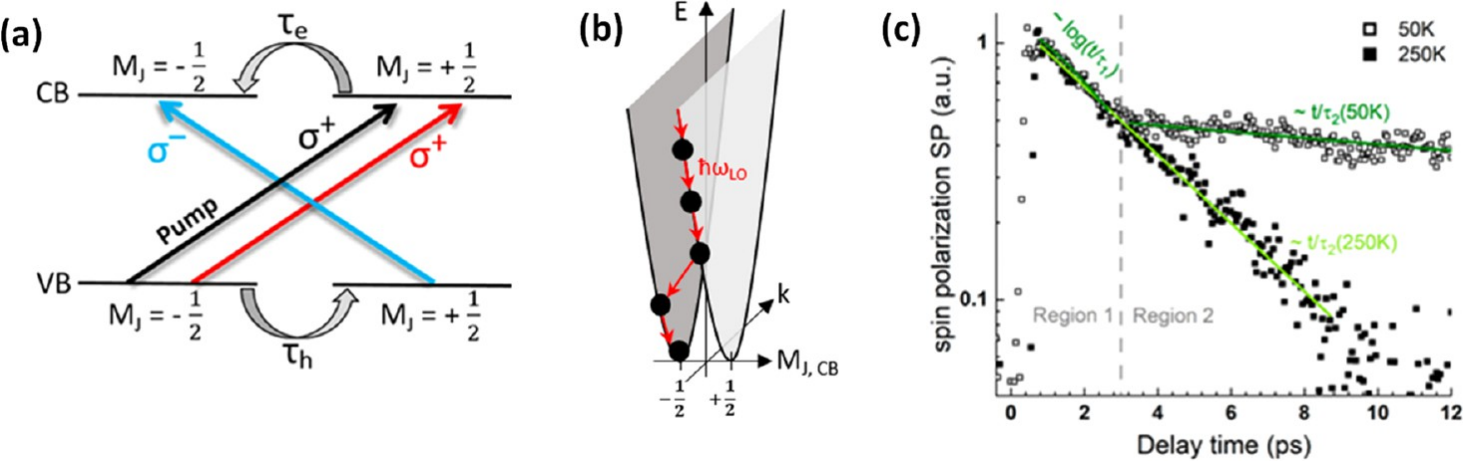
(a) Spin-dependent optical transitions between valence and conduction band states induced by circularly polarized pump and probe beams with similar and opposite helicities. (b) Electron spin-relaxation process in CB due to LO–phonon scattering process. (c) Temperature-dependent SP in CsPbI_3_ NCs. At 50 K, an additional spin-relaxation channel appears due to the exciton spin-flip. Adapted from ref (821). Copyright 2020 American Chemical Society.

Spin dynamics of particular spin states of photoexcited carriers could be studied using helicity-dependent time-resolved differential transmission spectroscopy by employing circularly polarized light for pump and probe.^820^ The helicity of the circular polarization can be controlled by the rotation of the azimuthal angle of the optical axis of a quarter wave plate (λ/4) with respect to the linear polarization axis of the pump/probe beam. The detector view conventions for positive helicity: σ^+^ = left handed circular polarization; negative helicity: σ^–^ = right handed circular polarization. Figure 100a schematically illustrates the possible optical transitions induced by the circularly polarized resonant optical pumping at the band gap of Pb^2+^-based MHP. Under σ^+^ excitation, the electron flips from the *M*_J,VB_ = −1/2 state into the *M*_J,CB_ = +1/2 state because of the conservation of angular momentum in an optical transition. Then, the conduction band electron can undergo an intraband *M*_J_ spin-flip from *M*_J,CB_ = +1/2 to *M*_J,CB_ = −1/2 at a rate of 1/τ_e_. Similarly, the holes in the VB can undergo spin-flip from *M*_J,VB_ = −1/2 to *M*_J,VB_ = +1/2 at a rate 1/τ_h_.

**Figure 101:**
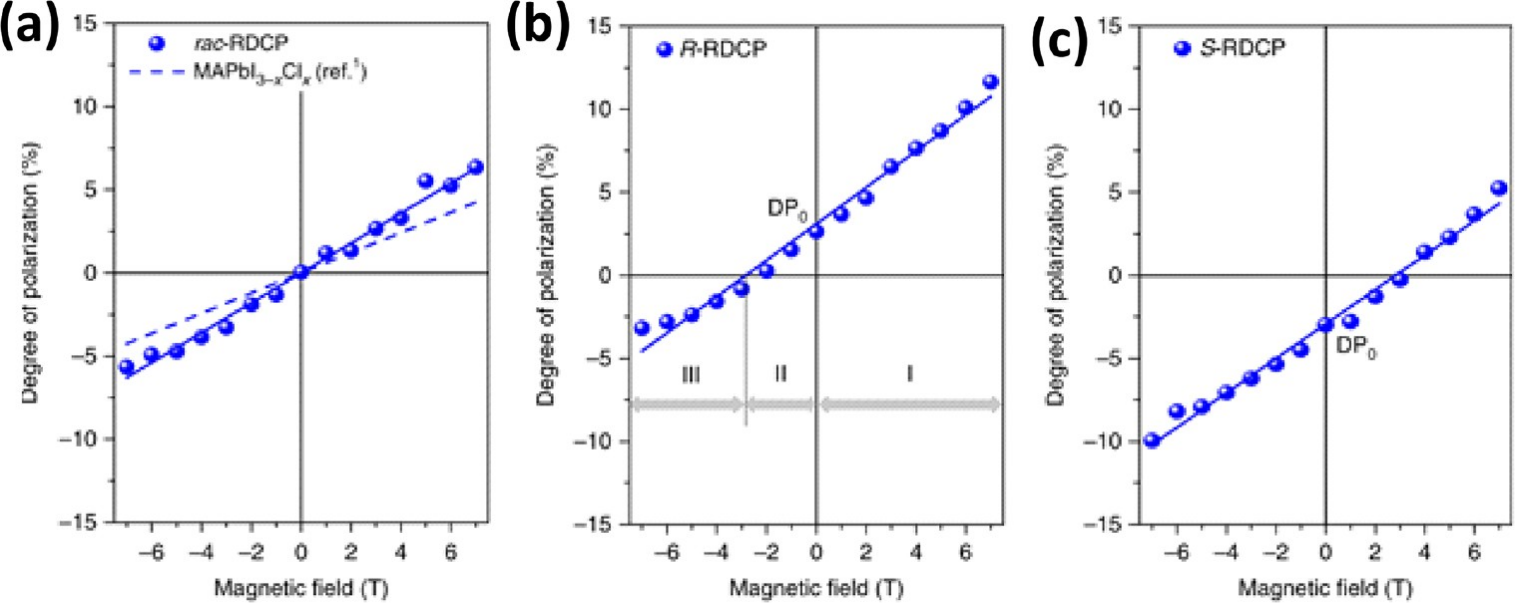
Degree of photoluminescence polarization for *rac*-RDCP (a), *R*-RDCP (b), and *S*-RDCP (c) with magnetic field varied from −7 T to 7 T. The graph of *R*-RDCP is divided into three regions: I, II, and III. At *B* = 0 (no external magnetic field), there is a degree of polarization (DP_0_) for *R*-RDCP. When a positive magnetic field is applied, the degree of polarization increases with the magnetic field (region I). In region II, as a negative magnetic field is applied, the degree of polarization decreases accordingly until it is zero. As a stronger negative magnetic field is applied, the degree of polarization changes sign from positive to negative (region III). Opposite phenomena are observed for *S*-RDCP. Adapted with permission from ref (20). Copyright 2018 The Authors.

Strohmair *et al.* have reported spin-relaxation processes of free charge carriers in CsPbI_3_ nanocubes using circularly polarized differential transmission spectroscopy.^821^ They observed that the spin-polarization (SP_max_) [(Δ*T*/*T*)_σ+σ+_ – (Δ*T*/*T*)_σ+σ–_] decreases dramatically for above band gap excitation and is almost 70% smaller at 2.32 eV compared to SP_max_ detected at 1.92 eV. The spin-polarization of the photoexcited charge carriers diminishes during thermalization and cooling down to the band-edge by emitting longitudinal optical phonons (Figure 100b). In this context, Strohmair *et al.* emphasized the dominant contribution from LO phonons *via* the Elliott-Yafet spin-relaxation mechanism in the case of free carriers in CsPbI_3_ NCs due to strong Fröhlich interaction present in MHPs.^821^ At low temperature the spin-relaxation time increases and an additional fast relaxation channel appears due to excitonic processes becoming prominent. The faster spin-relaxation channel at low temperature occurs *via* Coulomb mediated exchange interaction according to the Bir–Aronov–Pikus (BAP) model (Figure 100c).^821,822^ Spin-dynamics has also been studied in polycrystalline MAPbI_3_ thin films using helicity-dependent time-resolved transient absorption spectroscopy. The spin-relaxation times of the photoexcited free charge carriers have been found to be in the range of a few picoseconds, and the spin-depolarization was attributed to the Elliott-Yafet (EY) mechanism.^823^ In the case of 2D-layered (C_6_H_5_C_2_H_4_NH_3_)_2_PbI_4_, the observed exciton spin-relaxation time was even on the shorter time scales. Due to the fact that the exciton binding energy in 2D-layered perovskites is relatively high (∼180 meV), the spin-relaxation mechanism is usually controlled *via* Coulomb exchange interaction and is described by BAP model.^822^ Using pump–probe Kerr rotation, Belykh *et al*. measured that the charge carrier spin-relaxation in CsPbBr_3_ perovskite crystals is in the nanosecond regime.^46^ They assigned the long-lasting spin-relaxation time to hyperfine interaction between localized charge carriers and the nuclei spins. Li *et al.*(152) have observed decrease in spin lifetime with decrease in size CsPbBr_3_ and CsPbI_3_ QDs. In the case of CsPbI_3_ QDs, the spin-relaxation time decreased from 3.2 to 1.9 ps for the QD size reduction from 8.3 to 4.2 nm while it decreased from 1.9 to 1.2 ps for CsPbBr_3_ QDs with size decreasing from 7.5 to 3.5 nm. Elliot-Yafet spin-relaxation mechanism was postulated to be absent in the case of CsPbBr_3_ QDs where electron–hole exchange interaction, surface scattering, and spin–spin interaction have been held responsible as probable spin-relaxation channel.^152^ Furthermore, spin-polarization has also been induced externally in MHPs using chiral ligands or by doping with transition metal ions.^20,817^ For example, Long *et al.* were able to achieve 3% spin-polarized photoluminescence from the reduced dimensional chiral perovskites at zero applied magnetic field due to the different emission rates of right and left handed circularly polarized light.^20^ To achieve the same magnitude of spin-polarized photoluminescence from achiral perovskite an external magnetic field of 5 T is needed, as shown in Figure 101.

### Chiral Perovskite NCs

Thanks to the flexible chemical composition and surface chemistry of perovskite NCs, additional functions and properties can easily be introduced through surface modifications. Recently, there has been a growing research attention regarding the introduction of chiral function into halide perovskites by their interaction with chiral ligands.^540,824−840^ The concept of chirality or handedness refers to the functional property of chiral materials/molecules to not be superimposable with their mirror images, and these are called enantiomers (*i.e.*, (*R*)-(−) (right) and (*S*)-(+) (left)).^841^ The distinctive property of chiral molecules is their ability to rotate the plane of linearly polarized light differently depending on the respective enantiomer. The reason for this so-called optical rotation lies in the circular birefringence, *i.e*., the refractive index is different for right and left circularly polarized light. With linearly polarized light depicted as a superposition of two circularly polarized waves (clockwise and counterclockwise), the polarization of light is rotated when passing through a chiral medium. Chiral molecules play a crucial role in many biological processes, real life systems and electronic devices.^842^ In general, most small chiral molecules exhibit optical activity in the ultraviolet region of the light spectrum. However, interestingly, it has been shown that such molecules can confer chirality on colloidal metallic or semiconductor NCs that show optical activity in the visible to near-IR region by means of surface functionalization. Over the last few decades, a significant amount of research work has been done regarding the fabrication and application of chiral plasmonic, and semiconductor NCs. Recently, these concepts have been extended to recently emerged perovskites for a variety of applications, including ferroelectrics, chiroptoelectronics, and chiro-spintronics. Readers may also refer to two latest review articles on chiral perovskites.^840,1356^

The initial studies on chiral perovskites were mainly focused on 1D single crystals, 2D-layered systems and bulk thin films.^20,824,826,828,832,836,837,839,840^ In 2003, Billing *et al.*(828) reported the synthesis of organic–inorganic hybrid 1D perovskite single crystals (((*S*)-C_6_H_5_C_2_H_4_NH_3_)[PbBr_3_]) by *in situ* incorporation of a chiral amine (1-phenylethylammonium (PEA), also called methylbenzylammonium (MBA)) as the counterion. However, their chiral properties were not investigated. After being out of limelight for a few years, chiral perovskites have regained attention after the chiroptical study of (*S*-MBA)_2_PbI_4_ and (*R*-MBA)_2_PbI_4_ 2D-layered perovskite films by Moon and co-workers in 2017.^837^ These perovskite enantiomers were achieved through the incorporation of the respective chiral organic molecule (*S*-MBA and *R*-MBA) into the layered lead-iodide framework. They exhibit oppositely signed circular dichroism (CD) signals at their excitonic transitions, while the chiral molecules alone do not show any CD signal at these wavelengths. After these findings, chiral 2D-layered perovskite films and single crystals have been significantly explored regarding their synthesis and applications.^824−826,832,836,838−840^ For instance, Chen *et al.*(839) and Wang *et al.*(832) independently demonstrated the fabrication of flexible photodetectors using chiral 2D perovskites for efficient detection of circularly polarized light. The principle of these CP light photodetectors is the generation of different photocurrents for different circular polarization states of detected photons. Furthermore, chiral 2D perovskites are being studied for exploring circularly polarized photoluminescence (CPP)^824,826^ and ferroelectricity.^840^ Recently, these concepts have been extended to colloidal perovskite NCs. They can be excellent candidates as CP light sources for optoelectronic applications owing to their high PLQY and easily tunable emission color. However, unlike chiral 2D-layered perovskites, only a few studies have been reported on colloidal chiral perovskite NCs.

**Figure 102:**
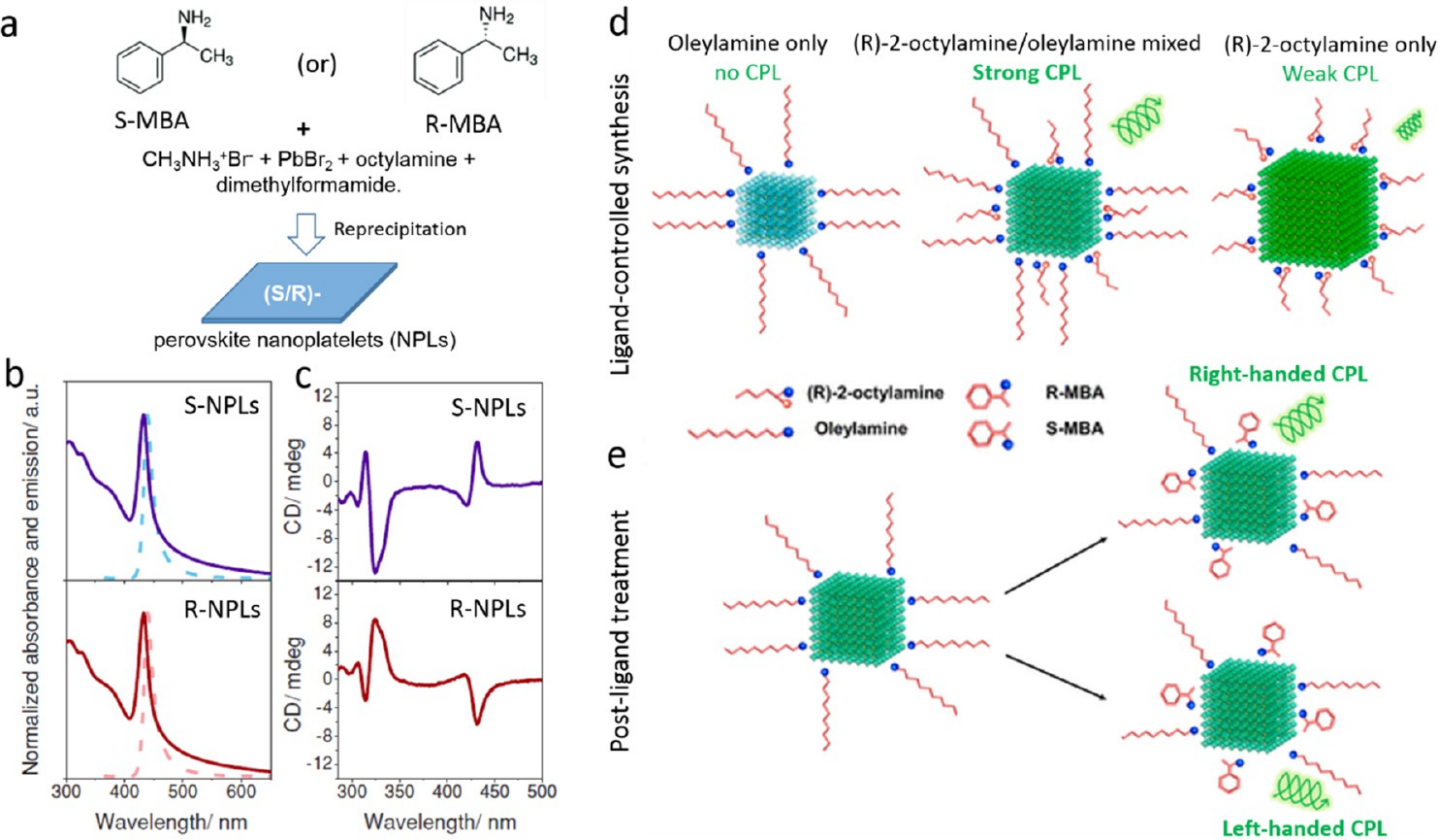
(a) Schematic representation of the synthesis of chiral organic–inorganic hybrid perovskite NPls by reprecipitation in the presence of chiral ligands (*S*-MBA and *R*-MBA). (b,c) UV–vis absorption (solid line) and PL (dotted line) (b), and the corresponding CD spectra (c) of the enantiomeric NPls obtained with *S*-MBA and *R*-MBA ligands. Panels b and c are adapted from ref (829). Copyright 2018 John Wiley & Sons, Inc. (d,e) Schematic illustration of the synthesis of FAPbBr_3_ nanocubes in the presence of the chiral ligand (*R*)-2-octylamine (d) and post-synthetic surface treatment of FAPbBr_3_ nanocubes with chiral ligands (*R*-, *S*-MBA.Br) (e). Panels d and e are adapted from ref (825). Copyright 2020 American Chemical Society.

**Figure 103:**
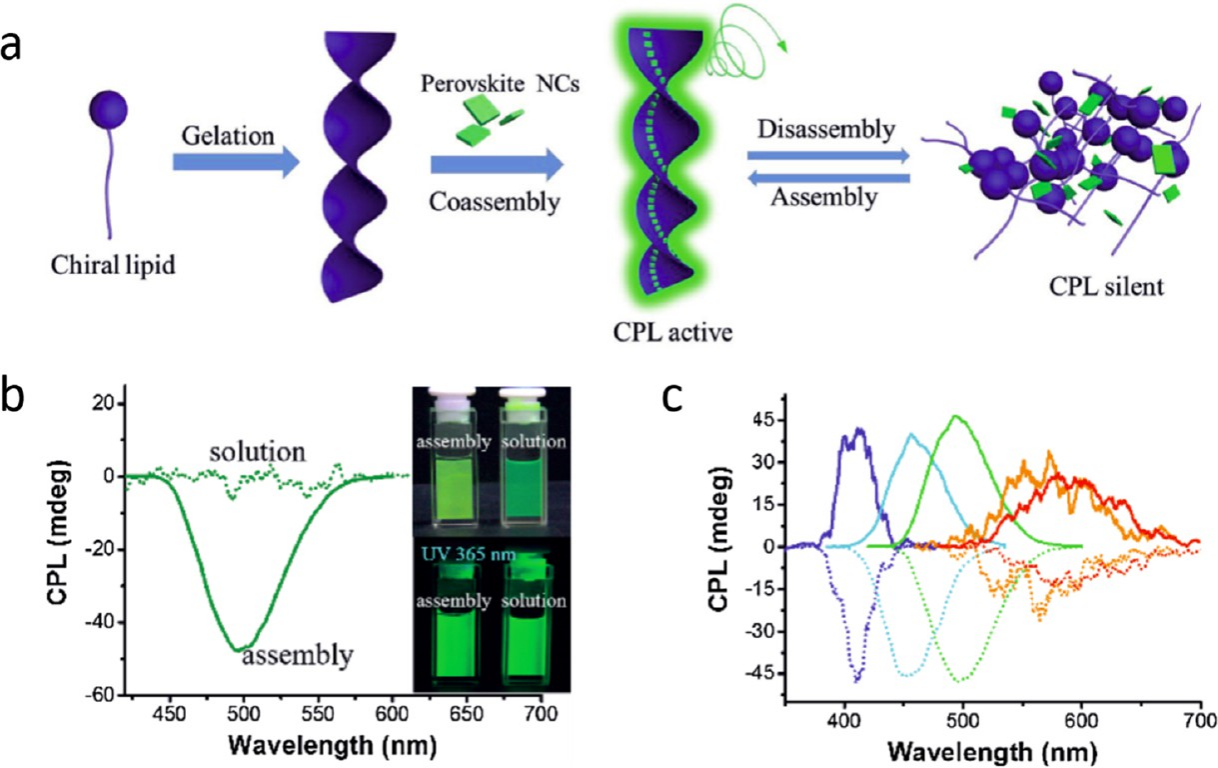
(a) Schematic illustration showing the gelation of chiral lipids into helical structure followed by coassembly of perovskite NCs along the helical gel to obtain chiral assemblies that emit circularly polarized luminescence (CPL). (b) CPL spectra of chiral gel induced CsPbBr_3_ assemblies and the disassembled CsPbBr_3_ colloidal solution, with the latter obtained by dispersing the gel-perovskite assemblies in chlorobenzene solution. The inset shows the photographs of the colloidal solutions containing perovskite NC assembles and individual NCs (disassembled) under room light (top) and UV light (bottom, 365 nm) illumination. (c) CPL spectra of chiral gel induced CsPbX_3_ NC assemblies of different halide composition. Adapted with permission from ref (830). Copyright 2018 John Wiley & Sons, Inc.

Generally, there are three different synthetic approaches to obtain colloidal chiral perovskite NCs: (1) *in situ* incorporation of chiral ligands during the synthesis (Figure 102)^825,829,834^ (similar to the case of chiral 2D-layered perovskites), (2) post-synthetic surface treatment with chiral molecules^825^ or chiral assemblies (Figures 102 and 103), and (3) synthesis of helical perovskite NCs (not yet achieved). Figure 102b summarizes the first two (*in situ* and post-synthetic) strategies one can use to synthesize chiral perovskite NCs. In this regard, Waldeck and co-workers^829^ demonstrated the *in situ* incorporation of chiral molecules (*S*-MBA and *R*-MBA) onto hybrid perovskite NPls synthesized by the reprecipitation method (Figure 102a). In this case, the chiral MBA cation molecules were introduced along with achiral octylamine as ligands into the precursor solution. The injection of precursor solution into toluene leads to the formation of chiral hybrid perovskite NPls, which exhibit sharp excitonic absorption and emission features that are consistent with quantum-confined NPls (Figure 102b). The NPls (*S*-,*R*-NPls) obtained with the two enantiomer ligands (*S*-,*R*-MBA) exhibit a mirror-image like CD spectrum with peaks at their excitonic absorption (Figure 102c), where the ligand molecules alone do not show any CD signal. The authors take this as a clear indication for the ligands imprinting their chirality onto the NPls electronic structure. In addition, the CD peaks observed at higher energy (∼300–350 nm) were assigned to the charge-transfer bands between the chiral ligands and the NPl surface (Figure 102c). Very recently, this *in situ* synthesis of chiral perovskite NCs has been extended to CsPbBr_3_^834^ and FAPbBr_3_^825^ NCs using the short chiral ligand α-octylamine (Figure 102d). The partial replacement of oleylamine with (*R*)-2-octylamine during the synthesis of FAPbBr_3_ NCs results in monodisperse chiral perovskite NCs that emit CPL with a luminescence dissymmetry (*g*) factor of 6.8 × 10^–2^, which is among the highest of reported perovskite materials.

In addition to these direct synthetic strategies, post-synthetic treatments have also been used to induce chirality in perovskite NCs by two different approaches. Firstly, the surface of presynthesized perovskite NCs can be modified with chiral ligands through ligand exchange (Figure 102d).^825^ For instance, Luther and co-workers demonstrated the post-synthetic ligand exchange on FAPbBr_3_ NCs with chiral ligands (*S*-,*R*-MBA), which induces CPL with an average dissymmetry *g*-factor of ±1.18 × 10^–2^. The second post-synthetic approach is the supramolecular self-assembly of NCs into helical structures.^830,843^ Previously, this approach was extensively used to induce chirality in plasmonic NCs using biomolecules such as DNA, DNA origami, and supramolecular fibers.^843−845^ Recently, Shi *et al.*(830) demonstrated the supramolecular self-assembly of CsPbX_3_ (X = Cl, Br, and I) into chiral assemblies that emit CPL by mixing chiral organogels made of lipids (*N*,*N*′-bis(octadecyl)-l-glutamic diamide (LGAm) and its enantiomer (DGAm)) with perovskite NCs in hexane (Figure 103a). The shape of the emission spectra remains unchanged with a slight red shift after self-assembly. However, interestingly, the perovskite NC assemblies exhibit CPL with a dissymmetry *g*-factor up to 10^–3^, while disassembled gels do not show CPL (Figure 103b). The disassembled gels were obtained by dissolving the DGAm–CsPbBr_3_ hybrid assemblies in chlorobenzene. The wavelength of the CPL peak is easily tunable across the visible spectrum of light by varying the halide composition (Figure 103c). Furthermore, it has been shown that these chiral assemblies could be incorporated into polymer film to obtain flexible CPL devices. Despite these interesting reports, the study of chiral perovskite NCs is still in the beginning stage. There are many questions yet to be addressed regarding colloidal chiral perovskite NCs. For instance, the mechanism of chiral induction *via* surface ligands on 3D perovskite NCs is still unclear. In addition, the number of chiral ligands used so far to modify the surface of perovskite NCs are limited because many chiral molecules are not missile in nonpolar solvents. Furthermore, perovskite NCs with helical morphology have yet to be achieved. More importantly, the application of chiral perovskite NCs in optoelectronic and spintronic devices needs to be explored.

### Charge Carrier Dynamics

Understanding the fate of the photoexcited charge carriers in a semiconducting material is of fundamental importance for the development of efficient optoelectronic devices. Photoexcitation produces electron- hole pairs whose energy relaxation channels depend on a variety of conditions.^10,846,847^ Followed by initial carrier thermalization, the hot charge carrier loses its energy by emitting optical phonons and successively relaxes down to the electronic band-edge. The charge carriers then either radiatively decay to produce light or recombine nonradiatively. The following sections discuss various such energy relaxation dynamics in MHP under ultrafast photoexcitation.

**Figure 104:**
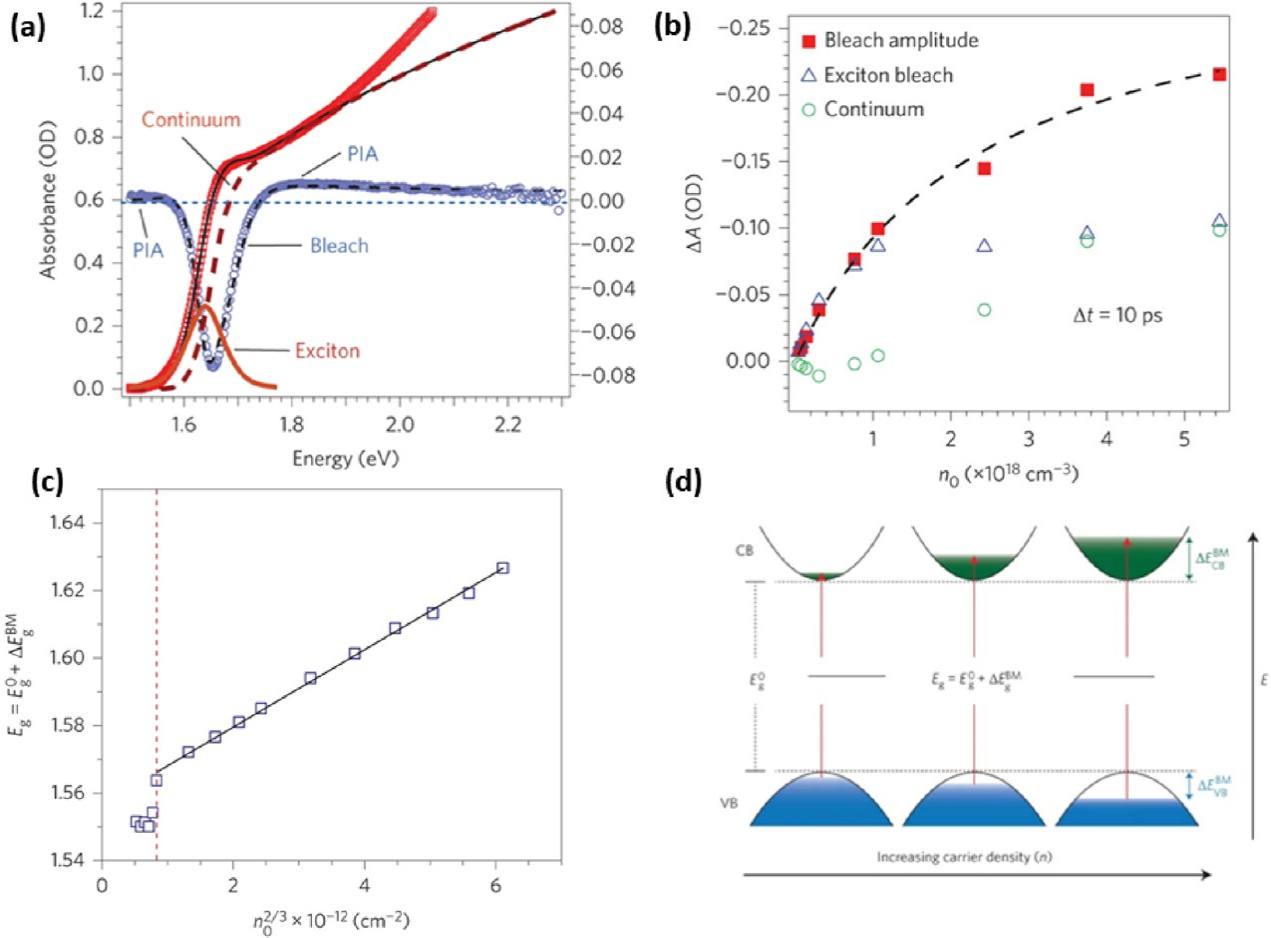
(a) Steady-state (red squares) and transient absorption (TA) (blue circles) spectra of a typical MAPbI_3_ perovskite thin film. Black line: modeled band-edge absorption. Red dashed line: continuum contribution. Red line: exciton contribution. Adapted with permission from ref (848). Copyright 2015 Nature Publishing Group. (b) Δ*A* at ℏω_probe_ = 1.65 eV as a function of the initial charge carrier density *n*_0_. Blue triangles: exciton contribution. Green circles: continuum band contribution. Adapted with permission from ref (848). Copyright 2015 Nature Publishing Group. (c) Modulation of the intrinsic band gap of MAPbI_3_ according to the Burstein–Moss model. The vertical dashed line marks the onset of band gap broadening. The solid line is a linear fit to the data after the onset. The linear trend indicates an agreement with band filling by free charge carriers. Adapted with permission from ref (849). Copyright 2014 Nature Publishing Group. (d) Schematic representation of the Burstein–Moss effect showing the contribution from both, electrons in the conduction band (CB) and holes in the valence band (VB) due to their similar effective masses. Adapted with permission from ref (849). Copyright 2014 Nature Publishing Group.

Figure 104a represents a typical steady state linear absorption spectrum (red squares) and a transient absorption (TA) spectrum (blue circles) of a planar MAPbI_3_ perovskite thin film. The absorption spectrum of MAPbI_3_ shows a steep rise at the absorption onset (at 1.6 eV). According to the Elliot model (Figure 90b,c), both excitonic and band to band continuum transitions contribute to the optical band gap in MHPs. This is shown by the representative TA spectrum (*ℏω*_pump_ = 1.82 eV) at a pump–probe delay of 10 ps. It has two general features: a sharp photobleach (PB) and a broad photoinduced absorption (PIA).^848^ The PB signal peaking at ∼1.65 eV has been attributed to both band filling and free carrier induced bleaching of the exciton transition. The PIA has been related to several factors such as hot carrier (HC) cooling, polaron formation and free carrier absorption.^848^ With increase in the excitation fluence, the amplitude of the PB signal (−Δ*A*) increases in a nonlinear fashion (Figure 104b). The spectral position was found to depend on the initial carrier density *n*_0_. Manser *et al.* reported a carrier density-dependent blue shift and broadening of the PB signal in MAPbI_3_ thin films due to the Burstein–Moss shift (Figure 104c).^849^ When the photogenerated carriers fill the electronic band-edge states (valence and conduction band), the effective band gap shifts toward higher energy due to Pauli blocking (Figure 104d).

#### Hot Carrier Relaxation Dynamics

**Figure 105:**
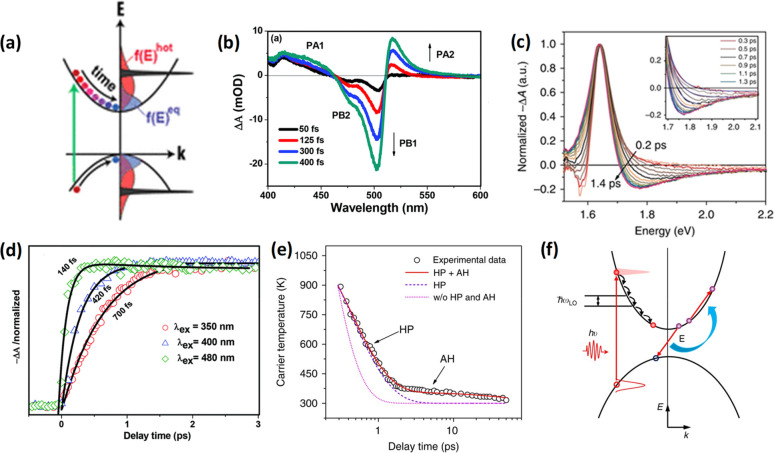
(a) Thermalization and relaxation schemes of the photoexcited electrons and holes. The initial δ-like distribution of the electrons and holes changes to an equilibrium distribution in two stages. Adapted under a Creative Commons CC-BY license from ref (857). Copyright 2018 American Chemical Society. (b) Time-dependent evolution of the TA spectrum (λ_ex_ = 350 nm) of CsPbBr_3_ NCs in the early time window (0.05–0.4 ps). Adapted with permission from ref (851). Copyright 2017 Royal Society of Chemistry. (c) Normalized TA spectra with variable delays from 0.2 to 1.4 ps, with the inset showing the high-energy tails fitted to the M–B distribution for extraction of the HC temperature. Adapted with permission under a Creative Commons Attribution 4.0 International License from ref (50). Copyright 2017 The Authors. (d) Formation kinetics of the PB1 band (representing HC cooling time) of CsPbBr_3_ NCs as a function of the excitation wavelengths. Adapted with permission from ref (851). Copyright 2017 Royal Society of Chemistry. (e) HC cooling dynamics in a MAPbI_3_ thin film following photoexcitation at 2.48 eV with a carrier density *n*_0_ of 10.4 × 10^18^ cm^–3^ at RT. Black circles: HC temperature extracted from TA spectra. The lines show the calculated HC cooling dynamics for τ_h_ = 0.6 ps: with a hot-phonon (HP) effect only (violet dashed line); with both HP and Auger heating (AH) effects (bright red line); and without HP and AH effects (magenta line). Adapted with permission under a Creative Commons Attribution 4.0 International license from ref (50). Copyright 2017 The Authors. (f) Schematic of the hot electron relaxation process. Auger heating, which contributes to further deceleration of hot electron cooling, is also shown. The same processes apply to the hot holes but are omitted for clarity. Reprinted with permission under a Creative Commons CC BY 4.0 license from ref (50). Copyright 2017 The Authors.

When excited by photons with energy higher than the band-gap energy, the charge carriers (electrons and holes) are produced in states much above the band-edge states with a non-equilibrium distribution in energy. These “hot carriers” thermalize through carrier–carrier scattering processes within 1 ps. The subsequent process is called “carrier cooling”, in which the quasi-equilibrated HCs (at temperature higher than the lattice temperature and governed by the Fermi–Dirac distribution) dissipate their excess energy as heat *via* phonon emission and come to the band-edge states (Figure 105a).^50,850^

The overall hot carrier cooling process can be probed using ultrafast transient absorption and photoluminescence measurement techniques. Figure 105b shows the TA spectra of CsPbBr_3_ NCs for short (0.05–0.4 ps) time scales, where the TA spectra comprise positive differential absorption (Δ*A*) bands PA1 and PA2 and a strong negative photobleach signal due to carrier-filling effect of the band-edge states.^851^ The formation kinetics of this bleach signal (PB1) delivers a carrier cooling time (τ_C_).^851,852^ The time dependence of the recovery of the secondary weak bleach signal (PB2) (Figure 105b) matches with the formation kinetics of PB1. The lower energy absorption band (PA2), which is related to the HC cooling, has been recently attributed to polaron formation.^853−856^ Another approach to probing HC cooling is by measuring the carrier temperature by fitting the high-energy tail of the TA spectra to a Maxwell–Boltzmann distribution (Figure 105c).^50,848^ However, the exact estimation of the individual contributions of hot holes and hot electrons to the carrier cooling time is difficult as the excess energy is almost equally distributed between the hot electrons and hot holes. As the energy of HC depends on the energy provided in excess of the band gap energy, τ_C_ directly correlates with the excitation energy. The higher the excitation energy is, the longer is the hot carrier cooling time. In the case of CsPbBr_3_ NCs, Mondal *et al.* reported an increase in τ_C_ from 140 to 700 fs, as the excitation wavelength was changed from 480 to 350 nm (Figure 105d).^851^

The HC cooling dynamics also depends on the excitation fluence and cannot be explained by the hot phonon effect alone.^706,850,858^ For all APbBr_3_ NCs (A = Cs, MA, and FA), HC cooling slows down with an increase in excitation fluence.^859^ At high excitation fluence, the carrier–carrier interactions come into the picture due to high carrier densities and this can cause re-excitation of the hot charge carriers (called “Auger heating”) and slow down the overall HC cooling process. Fu *et al.* reported that, at carrier densities above 10^19^ cm^–3^, the HC cooling dynamics is governed by the combined effect of a hot phonon and Auger heating.^50^Figure 105e shows the HC cooling dynamics in MAPbI_3_ when photoexcited at 2.48 eV at room temperature with an initial carrier density of 1 × 10^19^ cm^–3^. Two gradients are clearly visible, indicating the presence of two distinct HC cooling mechanisms which are the hot phonon effect and the Auger effect. There are two distinct types of Auger recombination processes: the intraband and the interband Auger recombination among which the latter process (also called Auger heating) causes a nonradiative transfer of the electron–hole recombination energy to a third electron (or hole), resulting in the excitation of the third carrier to higher energy level (Figure 105f). As the Auger lifetime (τ_Aug_) is dependent on the volume (*V*) of the NCs (τ_Aug_ ∼ *V*^1/2^), the HC cooling time at high excitation fluence is expected to be dependent on the NCs’ volume.^860^ Indeed, an increase in the HC cooling time from 12 to 27 ps has been observed with increasing the size of the NCs from 4.9 to 11.6 nm.^850^

In CsPbX_3_ (X = Br and I) NCs, τ_C_ (for the same amount of excess energy) decreases while going from iodide-based NCs to bromide-based NCs: CsPbI_3_ (580 fs) > CsPb(Br/I)_3_ (380 fs) > CsPbBr_3_ (310 fs).^852^ As the halide’s orbitals mainly contribute to the valence band of the NCs, this HC cooling time seems to represent the effective hot hole cooling dynamics rather than the hot electron cooling.^852^ The HC cooling time is also influenced by the A-site cation composition in MHP NCs, where it was observed that τ_C_ decreases from Cs- to FA-based NCs: CsPbBr_3_ (390 fs) > MAPbBr_3_ (270 ps) > FAPbBr_3_ (210 fs).^859^ A faster τ_C_ in hybrid perovskites (FAPbBr_3_ and MAPbBr_3_) compared to that in the Cs-based MHP is attributed to a stronger carrier–phonon coupling facilitated by the vibrational modes of the organic cations.^859,861,862^ The role of molecular vibrations in HC relaxation is confirmed by the ability to slow down the cooling process at lower temperatures for FAPbBr_3_, while no/a less strong effect is observed for CsPbBr_3_ NCs.^863^ The dependence of the HC cooling on the B-site cation was studied by a partial replacement (60%) of Pb with Sn and found to slow down the HC cooling time of MAPbI_3_ from 0.3 to 93 ps.^864^ A very slow HC cooling in FASnI_3_ thin films was reported to give rise to hot PL.^865^ However, as an opposite trend (a faster HC cooling dynamics upon partial Sn substitution in CsPbBr_3_ NCs) is also reported recently,^528^ and more studies are needed to understand the exact role of “Sn” on HC cooling dynamics in Sn-doped lead-halide perovskite NCs.

In quantum-confined systems, the HC cooling time depends on the size of the NCs. For example, HC cooling dynamics become faster (from 700 to 500 fs) when the size of MAPbBr_3_ NCs is increased from ∼4.9 to 11.3 nm.^850^ A slower HC cooling in smaller NCs is attributed to the intrinsic phonon bottleneck effect due to the availability of fewer phonon modes.^850,866^ Interestingly, a small change in HC cooling time of CsPbBr_3_ NCs on varying the edge length from 2.6 to 6.2 nm indicates the absence of any hot phonon bottleneck in this class of NCs.^867^ The effect of dimensionality on HC cooling dynamics was also investigated. The HC cooling was reported to be much faster in 2D MAPbI_3_ NPls compared to quasi-3D system^857^ due to the low dielectric screening and high surface to volume ratio of the 2D NPls. An increase in HC cooling time from 260 to 720 fs for A_2_PbI_4_ on changing the organic spacers from C_6_H_5_C_2_H_4_NH_3_^+^ (ε = 3.3) to HOC_2_H_4_NH_3_^+^ (ε = 37) is a reflection of the influence of dielectric screening on HC cooling dynamics, too.^868^ A slowdown of HC cooling due to the formation of large polarons at low excitation fluence has also been reported very recently.^853,869,20^

#### Carrier Trapping and Recombination Dynamics in MHPs

Radiative recombination of the charge carriers is one of the most important channels in direct band gap semiconductors that determines their utility in optoelectronic devices. Radiative recombination is slow compared to exciton dephasing, spin-relaxation, and HC cooling time and is commonly observed on the picosecond–nanosecond time scale. If indeed the perovskites were perfectly defect tolerant,^21,870^ the radiative recombination would have been the only route for the relaxation of the charge carriers. However, multiexponential PL decay dynamics of most perovskites NCs even at low excitation fluence suggests the existence of sub-band-gap energy levels arising from various defects that act as trap centres.^101,871−876^ These trapped carriers can return to the conduction or valence band and recombine radiatively, if the de-trapping process is effective such as in the case of shallow defects.^875,814^ This process is responsible for an additional longer decay component in the PL decay profile.^877,878^ However, when the separation between the trap state and band-edge is large, as in the case of deep traps, the charge carriers relax nonradiatively.^875^ For smaller NCs, which have a high surface to volume ratio, “surface trapping” can also facilitate nonradiative recombination of the charge carriers resulting in lowering of the PL efficiency and acceleration of the PL decay dynamics.

**Figure 106:**
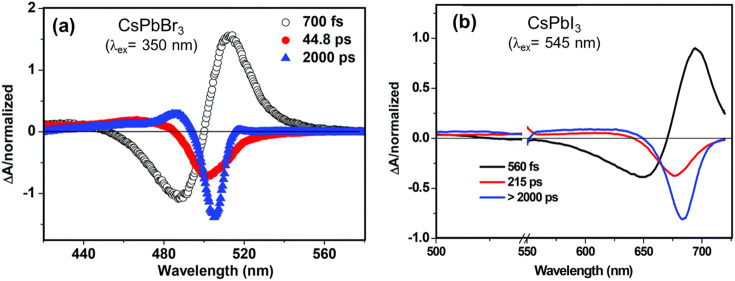
Decay-associated TA spectra of (a) CsPbBr_3_ and (b) CsPbI_3_ NCs. Adapted with permission from ref (851). Copyright 2017 Royal Society of Chemistry.

While the time constants for the radiative processes are most commonly estimated from the PL decay profiles measured using the time correlated single-photon counting (TCSPC) technique, the nonradiative recombination processes are much faster and require ultrafast TA and PL measurements. Most often, the temporal profile of the photobleach recovery signal (in TA measurements) contains a fast component due to carrier trapping in addition to the long component due to radiative recombination. The bleach recovery kinetics of CsPbBr_3_ NCs consists of two components (∼45 ps and ∼2 ns) (Figure 106) in which the former has been assigned to electron trapping.^851^

**Figure 107:**
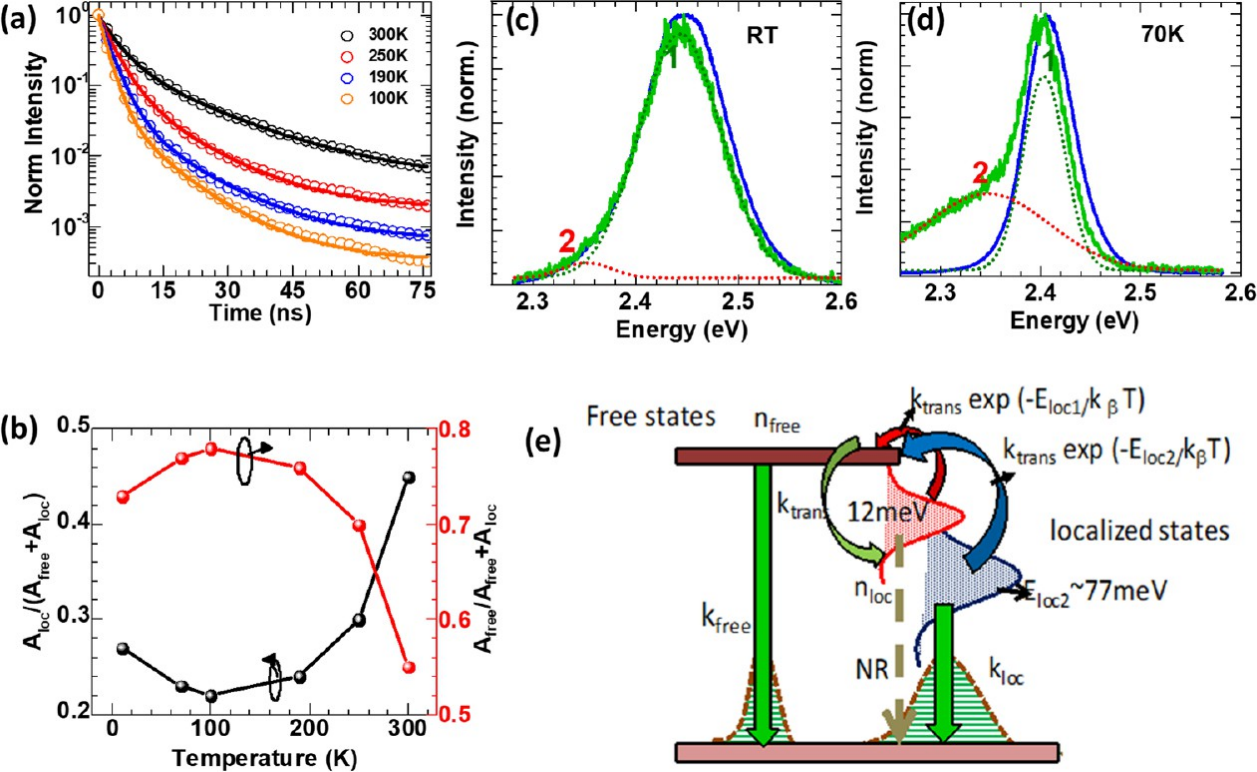
(a) Temperature-dependent PL decay in CsPbBr_3_ NCs. (b) Relative weight contributions of the free *vs* localized states in controlling the PL dynamics at different temperatures. Time-resolved PL spectra (PL1 at *t* = 0 and PL2 at *t* = 32 ns) at room temperature (c) and at low temperature (d) for CsPbBr_3_ NCs. (e) Schematic of the model depicting interactions between free and localized states. Adapted with permission from ref (814). Copyright 2018 John Wiley & Sons, Inc.

In the case of CsPbI_3_, carrier trapping time is estimated as ∼215–400 ps.^175,851,879^ A recent theoretical study shows that halide vacancies in the NCs are the major contributor to the defect energy levels, which are shallow in nature for CsPbBr_3_ and CsPbI_3_, but deep in the case of CsPbCl_3_.^86,194^ The high trap density in large band gap CsPbCl_3_ NCs accounts for its weak luminescence (PLQY <10%) and TA studies show multiple carrier trapping channels with time constants ranging from 3 to 64 ps.^148,783,880,881,782^ Dey *et al.* have studied the temperature-dependent time-resolved PL dynamics in the case of CsPbBr_3_ NCs, where they observed the PL decay getting faster when lowering the temperature.^814^ Additionally, a low-energy PL peak appeared at low temperature for the long time delays (Figure 107a–d). Both effects can be attributed to the presence of defect states. While at room temperature the efficient detrapping process slows down the PL decay, the emission from these localized states becomes significant at low temperatures as evident in the formation of the additional low-energy PL peak (Figure 107e).^814^ Very recently, trapping of the hot charge carriers in states within the band itself has been reported for APbBr_3_ NCs.^189,882,883^

**Figure 108:**
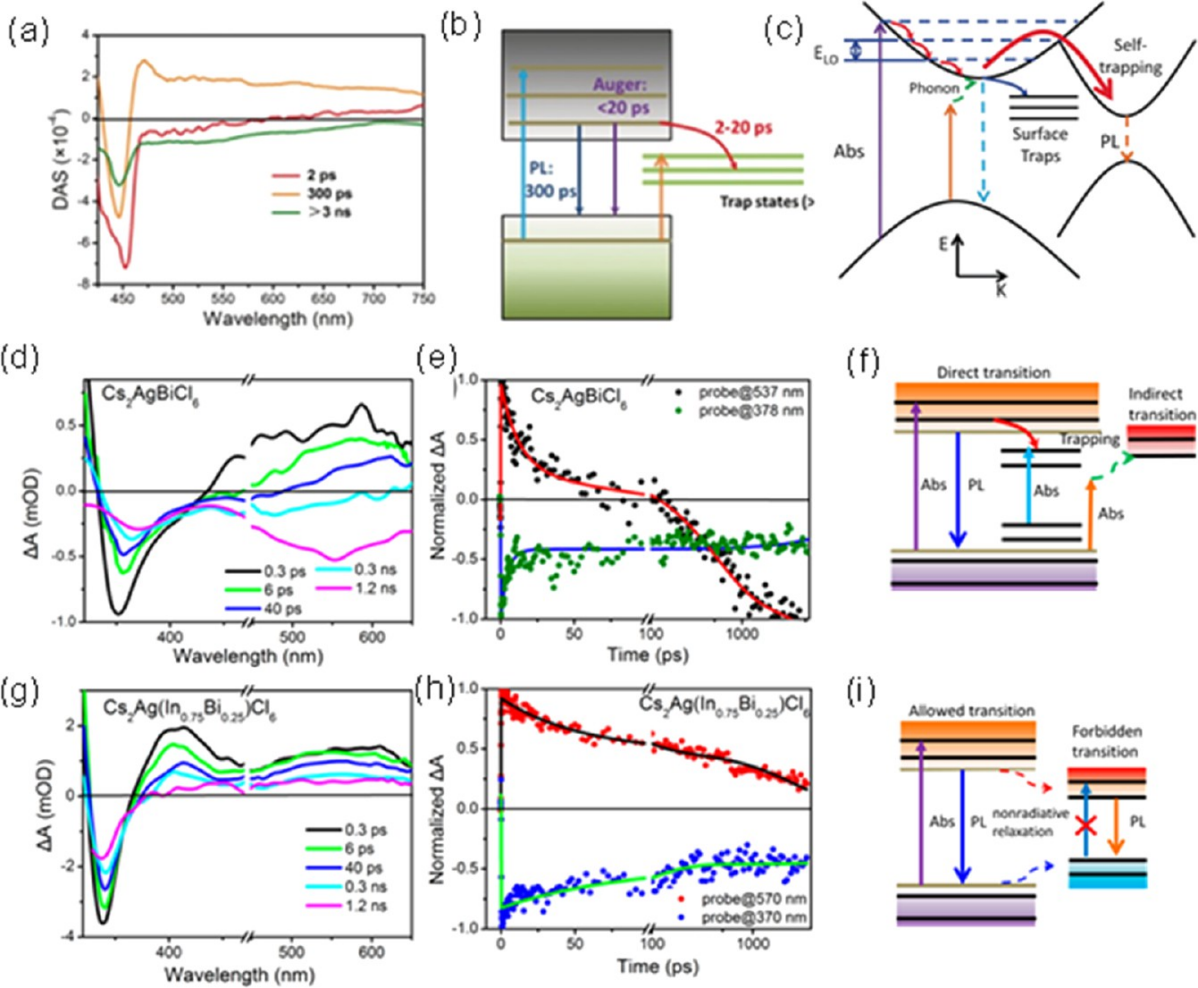
(a) Decay-associated spectra of Cs_3_Bi_2_X_9_ NCs. (b) Model illustrating several photoinduced processes in Cs_3_Bi_2_X_9_ NCs. Adapted with permission from ref (543). Copyright 2017 John Wiley & Sons. (c) Schematic illustration of the carrier dynamics of the Cs_2_AgSb_0.25_Bi_0.75_Br_6_ double perovskites. Adapted with permission from ref (571). Copyright 2019 John Wiley & Sons. Respective transient absorption spectra, kinetics, and schematic model explain the carrier relaxation channels of (d–f) Cs_2_AgBiCl_6_ and (g–i) Cs_2_AgIn_0.75_Bi_0.25_Cl_6_ NCs. Adapted from ref (884). Copyright 2018 American Chemical Society.

Lead-free perovskite NCs, which are recently receiving increasing attention due to their nontoxic nature,^543,544,571,884^ possess a very low PLQY and are so far less explored. For Cs_3_Bi_2_X_9_ (X = Cl, Br, I) NCs, the estimated time constant for carrier trapping, band-edge radiative recombination and trapped charge carrier relaxation are 2-20 ps, ∼300 ps and >3 ns, respectively (Figure 108a,b).^543^ Bleach recovery kinetics of Cs_2_AgSb_0.25_Bi_0.75_Br_6_ NCs reveal three components which have been attributed to self-trapping of the charge carriers (1–2 ps), surface trapping (50–100 ps), and geminate recombination (>5 ns).^571^ TA spectra of Cs_2_AgBiCl_6_ and Cs_2_AgIn_0.75_Bi_0.25_Cl_6_ show two-component ground-state bleach recovery with time constants of ∼100 ps due to carrier trapping and >2 ns due to radiative recombination.^884^ In the former case, the trapping contribution is, however, larger and an additional strong bleach signal due to sub-band-gap trap state absorption or indirect band gap transition is observed (Figure 108d). Recently, in the case of Cs_2_AgBiBr_6_ NCs, Dey *et al.* showed that the PL originates from defect-related bound excitons at the Γ-point corresponding to the direct band transition, *via* trapping of holes occurring on a time scale of hundreds femtoseconds.^779^ The PL measurements on the picosecond time scale using a streak camera revealed that the PL maximum, which is originally close to the excitonic resonance, shifts by more than 1 eV toward longer wavelength/lower energy within tens of picoseconds. This has been attributed to intervalley scattering. Whereas the emission from the direct bound excitons decays fast, the indirect emission showed a slow recombination.^779^ More experimental studies in combination with theoretical calculations are needed for a clear understanding of the underlying photophysical processes in these systems. Readers interested in carrier dynamics of lead-free perovskites may go through the accounts of Yang and Han.^885^

#### Exciton Recombination

In bulk and NC LHPs, both excitons and free carriers contribute to the radiative recombination.^849^ The populations of excitons and free carriers are determined by the initial exciton concentration and the exciton binding energy (*E*_B_). Excitons in bulk LHPs possess a very small *E*_B_, but the η_PL_ is typically lower than 10%.^886^ On the other hand, the quantum and dielectric confinement effects in LHP NCs increase *E*_B_ and the η_PL_ can approach unity at relatively low excitation density. At higher exciton concentrations, the Auger recombination pathway, including biexcitons and trions,^887−889^ and trap-assisted nonradiative recombination^885^ come into play. Therefore, it is obvious that the suppression of nonradiative recombination losses is essential to realize the optoelectronic applications of LHPs.

**Figure 109:**
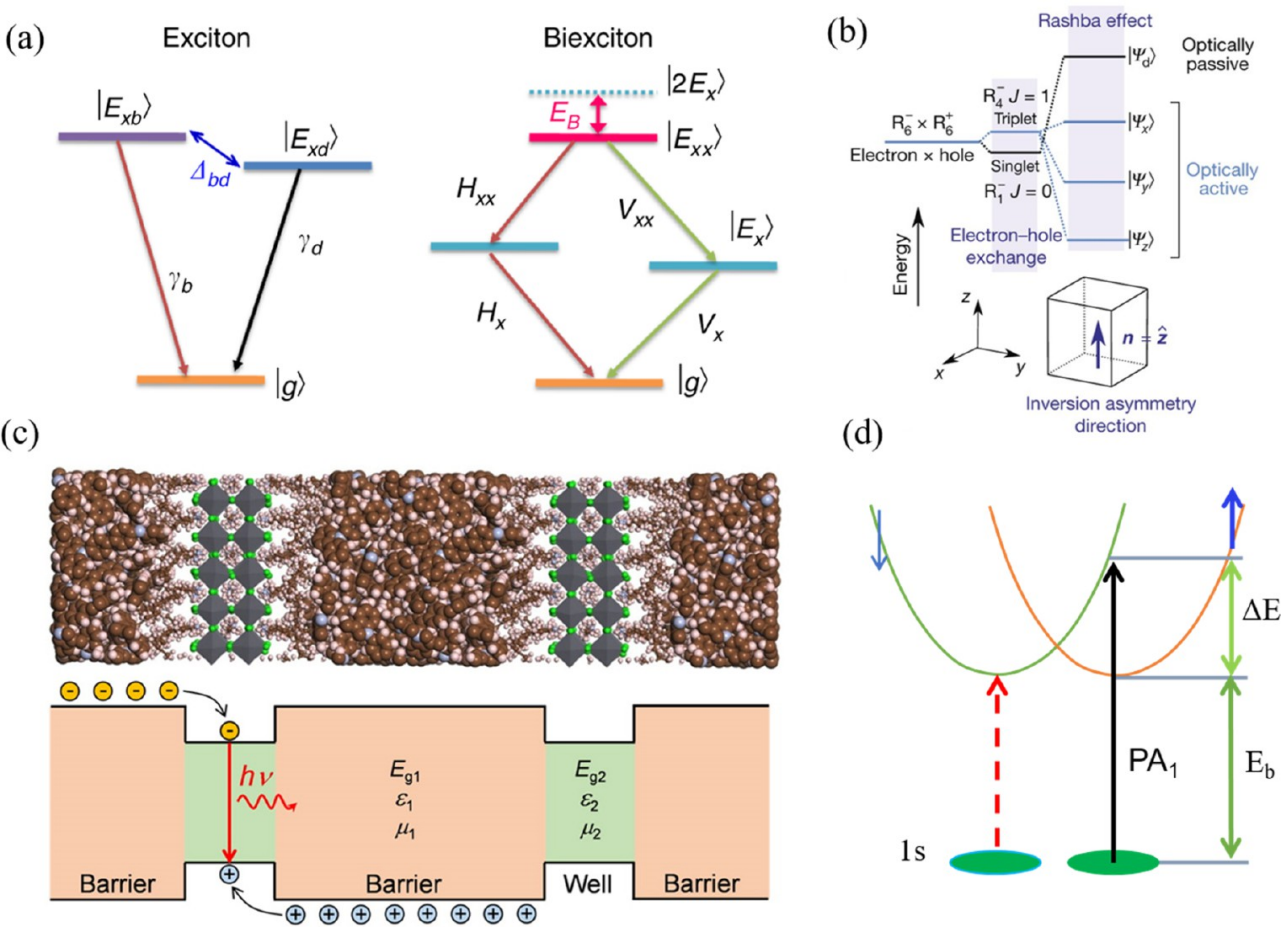
Excitonic characteristics in the lead-halide perovskites. (a) Schematic illustration of charge carrier recombination, including exciton and biexciton transitions, in the 2D CsPbBr_3_ perovskites. Adapted with permission under a Creative Commons CC BY license from ref (891). Copyright 2019 The Authors. (b) Schematic band structure demonstrating short-range electron–hole exchange and Rashba effect in 3D orthorhombic CsPbBr_3_ NCs. Adapted with permission from ref (147). Copyright 2018 Macmillan Publishers Limited, part of Springer Nature. All rights reserved. (c) Schematic (bottom) and molecular model (top) of the dielectric quantum wells formed between low dielectric constant, *k*, (barriers) and 2D MAPbBr_3_ perovskites (wells), illustrating excitonic recombination to enhance *E*_B_ in the wells. Adapted from ref (211). Copyright 2016 American Chemical Society. (d) Schematic band diagram depicting Rashba splitting in that occur due to SOC in the 2D (C_6_H_5_C_2_H_4_NH_3_)_2_PbI_4_.

It has been suggested that 2D LHPs possess a very high *E*_B_ that results in a low nonradiative recombination rate. This is due to (i) a relatively low density of intrinsic defects (owing to high defect formation energy), (ii) the presence of distinct polaronic effects, and (iii) the Rashba splitting induced bright triplet excitons (Figure 109a,b).^147,890,891^ More specifically, a high carrier recombination rate can be achieved by increasing the overlap between hole and electron wave functions by quantum confinement, enhancing the exciton localization (the Frenkel-like excitons).^892,893^ In fact, higher exciton binding energy should lead to high biexciton binding energy and thus recombination probability. Biexciton states are manifested by many-body excitonic interactions where two bright exciton states of opposite spins (±1) comprise one biexciton state. They have very low optical transition probability and are not stable at room temperature. Thus, they could exist either at cryogenic temperatures or under femtosecond pulse laser excitation. Chen *et al.* showed that the coupling of CsPbBr_3_ NPls (where exciton binding energy is very high) with plasmonic nanogap leads to an enhancement in the biexciton recombination under continuous wave excitation at room temperature.^891^ As shown in Figure 109a, a biexciton state decays through a cascade process of emitting either two horizontally or vertically polarized photons. The biexciton emission energy (*ℏ*ω_*xx*_) is determined by the energy gap between the biexciton energy (*E*_*xx*_) and single exciton emission (*E*_*x*_) *via* ℏω_*xx*_ = *E*_*xx*_ – *E*_*x*_. Thus, when two bright excitons bind together to form one biexciton, the energy of the whole system is decreased by Δ_*xx*_ (=2ℏω_*x*_ – ℏω_*xx*_), which is the biexciton binding energy. Indeed, the enhancement of *E*_B_ plays a crucial role in high-performance light-emitting devices. *E*_B_ can be increased by more than 1 order of magnitude from ∼10 meV in 3D bulk LHPs^894−896^ to >150 meV in 2D LHPs^200,892,895,897,898^ due to dielectric confinement effect (Figure 109c).^895,899,900^ In the quasi-2D perovskites, (BA)_2_(MA)_*n*−1_Pb_*n*_I_3*n*+1_, one can increase *E*_B_ up to 470 meV.^901^

Strong spin–orbit coupling and inversion asymmetry have been observed in inorganic CsPbX_3_ LHP NCs.^147^ It has been proposed that these systems also exhibit a high degree of Rashba splitting in the excited-state energy levels, which alters the degeneracy of triplet excited states and the order of energy sublevels, thereby yielding a bright triplet state as the lowest energy state.^147^ This is distinct from most quantum emitters, including organic fluorophores and inorganic quantum dots, in which the lowest excited states correspond to the dark triplets.^147^ It is noteworthy that recent experimental observations have suggested that the Rashba splitting effect becomes more pronounced in 2D quantum well and quasi-2D LHPs (Figure 109d).^902^

#### Multiexciton Dynamics

**Figure 110:**
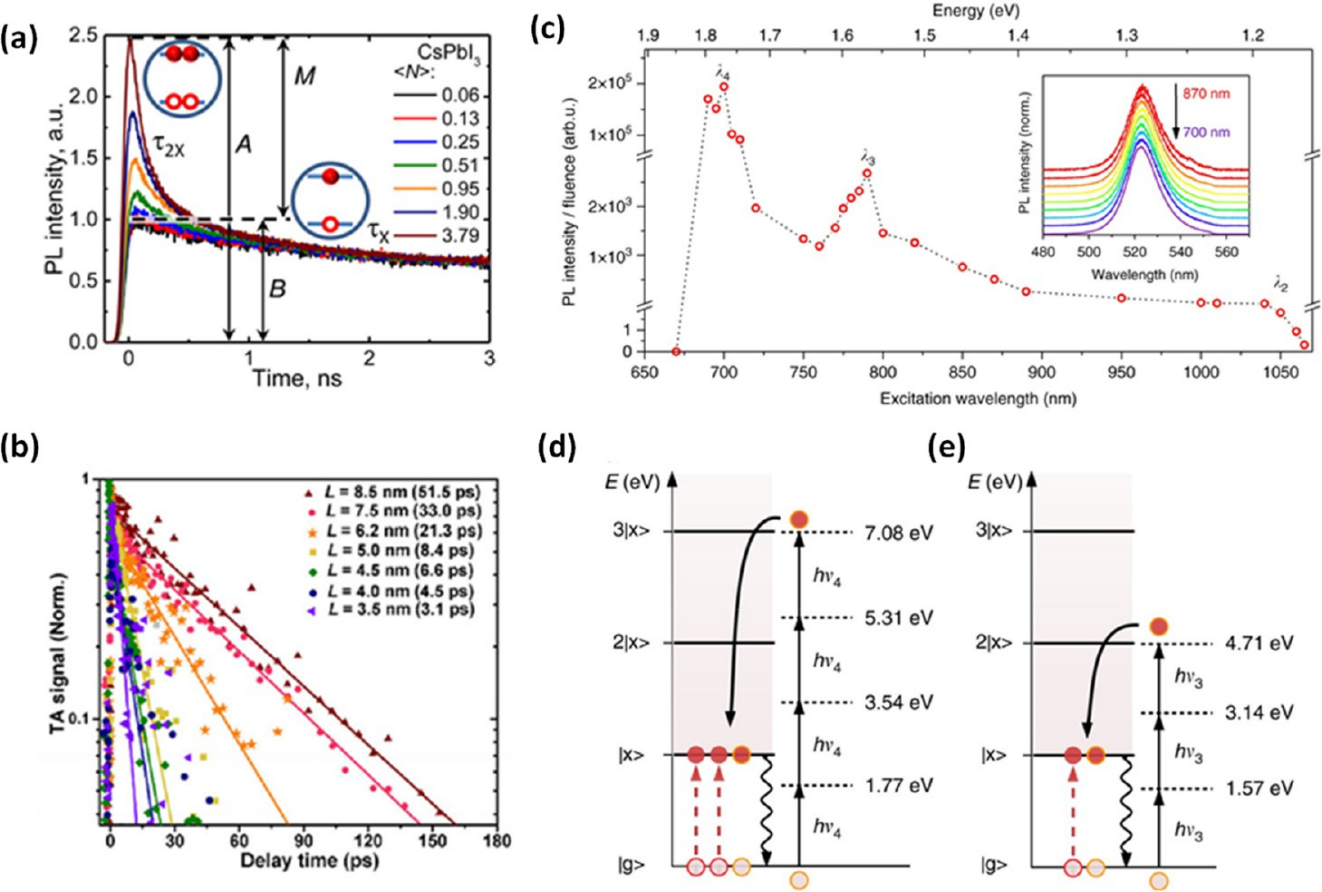
(a) Pump-fluence-dependent PL dynamics of CsPbI_3_. At early time, a short-lived PL component due to biexcitons (denoted as τ_2X_) emerges at higher pump intensities. A and B denote the amplitudes of the total PL signal and its single-exciton component, respectively, while M = A–B denotes the amplitude of the multiexciton signal. Adapted from ref (854). Copyright 2016 American Chemical Society. (b) Variation in biexciton lifetime with varying sizes of CsPbBr_3_ NCs. Adapted with permission from ref (907). Copyright 2018 Tsinghua University Press and Springer-Verlag GmbH Germany, part of Springer Nature. (c) Nonlinear absorption-induced PL in CsPbBr_3_ NCs as a function of the below-band-gap excitation wavelength. The dashed line is a guide for the eye. The inset shows the normalized PL spectra for excitation wavelengths varying from 870 to 700 nm. Energy diagram of the resonances between multiphoton excitation and multiexciton generation in CsPbBr_3_ NCs. Photoexcitation at 3*E*_*x*_ (d) and 2*E*_*x*_ (e) and subsequent generation of three (d) and two (e) excitons *via* multiple photon excitation processes with photons of energies *h*ν_4_ and *h*ν_3_, respectively. Panels c–e are adapted with permission under a Creative Commons CC by license from ref (908). Copyright 2018 The Authors.

When the excess energy available to a HC is high enough, it can generate a second exciton by transferring this energy. Generation of multiple excitons by absorption of a single photon can enhance the PCE of single-junction photovoltaics. In bulk semiconductors, the carrier multiplication efficiency is usually low due to rapid intraband relaxation processes. However, in the nanoscale regime, multiexciton generation is more efficient with a minimal energy loss. For example, generation of seven excitons is documented for PbSe QDs upon excitation with a photon energy of 7.8*E*_g_, indicating an energy loss of only ∼10%.^903^ Multiexcitons can also be generated using high fluence of the excitation laser pulse. Even though the solar flux density is not high enough to produce multiple excitons, studies on multiexciton dynamics are commonly performed using high photon flux.^854,872,904−906^ However, as the multiexciton dynamics is independent of the method of generation, the results of these studies can be applied to improve solar cell applications. Makarov *et al.* studied multiexciton dynamics in CsPbI_3_ NCs by monitoring the PL kinetics as a function of the pump fluence.^854^ The appearance of an additional fast decay component at higher laser fluences (Figure 110a) indicates the formation of multiexcitons. The generation of a large number of charge carriers in spatially confined NCs enhances the carrier–carrier interaction, which leads to Auger recombination, an additional nonradiative channel for the relaxation of the charge carriers. As both VB and the CB edge states of the perovskite NCs can accommodate a maximum of two charge carriers (two-fold degeneracy), multiexciton generation in these systems is limited to biexcitons.^854^ As the carrier–carrier interaction is enhanced in a confined condition, the volume (*V*) of the NCs influences the biexciton lifetime of a system.

Systematic studies of the volume dependence of the Auger lifetime of a series of NCs (FAPbBr_3_ and CsPbBr_3_) with varying sizes from a strongly regime to a weakly confined regime, confirm the decrease in the biexciton lifetime with decrease in NC volume (Figure 110b).^863,907^ The volume scaling of the biexciton lifetime (τ_*xx*_) is usually represented as τ_*xx*_ = γ*V*, where γ is the scaling factor, whose value is found to be an order of magnitude lower for FAPbBr_3_ (0.068 ± 0.005 ps/nm^3^) and CsPbBr_3_ (0.085 ± 0.001 ps/nm^3^) compared to CdSe or PbSe QDs (for which γ ≈ 1 ps/nm^3^).^863^ However, the reason for this large variation is not yet clear. A high multiexciton efficiency and low multiexciton generation threshold are advantageous from the practical point of view, and in this context, intermediate-confined FAPbI_3_ NCs appear to be the best choice. Multiexciton generation with a threshold of 2.25*E*_g_ and an efficiency of 75% has been demonstrated for this system.^909^ Even for CsPbI_3_, a carrier multiplication efficiency of 98% is reported for *E*_exc_ ≥ 2*E*_g_.^910^ The biexciton lifetime of the pure CsPbX_3_ NCs varies with the halide composition as CsPbI_3_ (90–115 ps) > CsPbBr_3_ (40–74 ps) > CsPbCl_3_ (∼20 ps).^854,855,860,888,904,911,912^ Systems with higher biexciton lifetime are of great interest as they provide a longer period for the extraction of biexcitons prior to nonradiative Auger recombination. Mondal *et al.* have shown that the biexciton lifetime of CsPbI_3_ can be almost doubled by doping a small amount of chloride or formamidinium ion into the system.^912^ Eperon *et al.* found a longer biexciton lifetime (198–227 ps) in hybrid perovskite NCs, FAPbBr_3_ and MAPbI_3_, compared to all-inorganic, CsPbBr_3_ NCs (74 ps).^888^ The effect of dimensionality of the perovskite NCs on the biexciton lifetime has also been studied using CsPbBr_3_ NPls and NRs of different lateral areas and rod lengths, respectively.^913^ A linear correlation is found between the biexciton Auger lifetime and the NPl lateral area and the NR length, which is related to exciton collision frequency. Reduced Auger probability per collision in 2D materials (NPls) explains the longer biexciton lifetime of it compared to that in 1D NRs.

Another possible nonradiative loss channel is the formation of a trion, which is a localized center containing three charged particles. A positively charged trion consists of two holes and one electron and a negatively charged one comprises two electrons and a hole. These species are formed on photoexcitation of a NC, which already contains a trapped electron or hole. Since the formation of trions requires re-excitation of the same NC, it can be avoided by performing the measurements under vigorously stirring, such that each photon is absorbed by a fresh NC sample. As the trions influence the PL behavior of the NCs (*e.g*., contributed to PL intermittency), it is important to understand the trion dynamics and several studies have been dedicated to this.^26,914,915^ A trion lifetime of 235 ps has been estimated by comparing the normalized bleach/PL kinetics of static and stirred CsPbI_3_ colloidal NCs.^854^ Yarita *et al.* estimated the lifetime of a biexciton and a trion in CsPbBr_3_ NCs to be 39 and 190 ps, respectively, by performing pump-fluence-dependent TA measurements.^916^ Wang *et al.* determined a trion lifetime of 220 ± 50 ps for CsPbBr_3_ NCs through carrier doping using double pump–probe spectroscopy.^917^ In another study, negative trions were generated in FAPbBr_3_ NCs using strong hole acceptors like CuSCN and their lifetime was estimated (∼600 ps).^918^ Considering that the trions are generally formed due to surface trapping of an electron or a hole, post-synthetic surface treatments can suppress the trion recombination process.^882,919^ Additional information on this topic can be found in a recent review.^920^

Nonradiative multiexciton annihilation processes can be avoided by below band gap multiphoton excitation and generation processes.^908^ Manzi *et al.* observed the PL centered at 523 nm from CsPbBr_3_ NCs assembly for a wide range of below band gap nonlinear excitations (Figure 110c).^908^ They noticed that the spectral shape of the emitted PL remained unchanged while the emission intensity highly depended on the excitation wavelength. PL can be observed starting at an excitation wavelength around λ_2_ = 1030 nm (photon energy *h*ν_2_ = 1.20 eV ≃ 0.50*E*_g_). The PL intensity then increases toward lower excitation energies in a nonmonotonic fashion. Two distinct peaks, located at an excitation wavelength of λ_3_ = 790 nm and λ_4_ = 700 nm (corresponding to the photon energies *h*ν_3_ = 1.57 eV ≃ 0.66*E*_g_ and *h*ν_4_ = 1.77 eV ≃ 0.75*E*_g_, respectively), have been found with the PL intensity being several orders of magnitude higher (10^3^ and 10^5^, respectively) than the signal detected in the vicinity of λ_2_. These particular energies (at λ_3_ and λ_4_) perfectly match the multiples of the exciton energy *h*ν_*x*_, suggesting a multiple photon absorption and a subsequent resonant generation of multiple excitons. A schematic representation of the combined multi photon excitation and multiexciton generation processes in CsPbBr_3_ NCs system is shown in Figure 110d,e. For the excitation wavelength λ_4_, the NCs assembly undergoes a 4-photon absorption and reaches an energy level resonant with 3*E*_*x*_ = 7.10 eV. Likewise, for the excitation wavelength λ_3_, a 3-photon absorption process occurred, giving rise to photogenerated excitons with an energy resonant with 2*E*_*x*_ = 4.73 eV.

### Charge Transfer Dynamics

Our discussion so far has been restricted to different intrinsic relaxation processes of the photogenerated charge carriers in perovskites. However, for applications like in solar cells, these photoactive materials are sandwiched between carrier harvesters. It is thus necessary to have an understanding of how charge-transfer dynamics (at the donor–acceptor interface) competes with the dynamics of intrasystem relaxation processes. In this section, we highlight some of the charge-transfer studies on various perovskite NCs with a variety of carrier acceptors.

#### Single Electron/Hole Transfer

**Figure 111:**
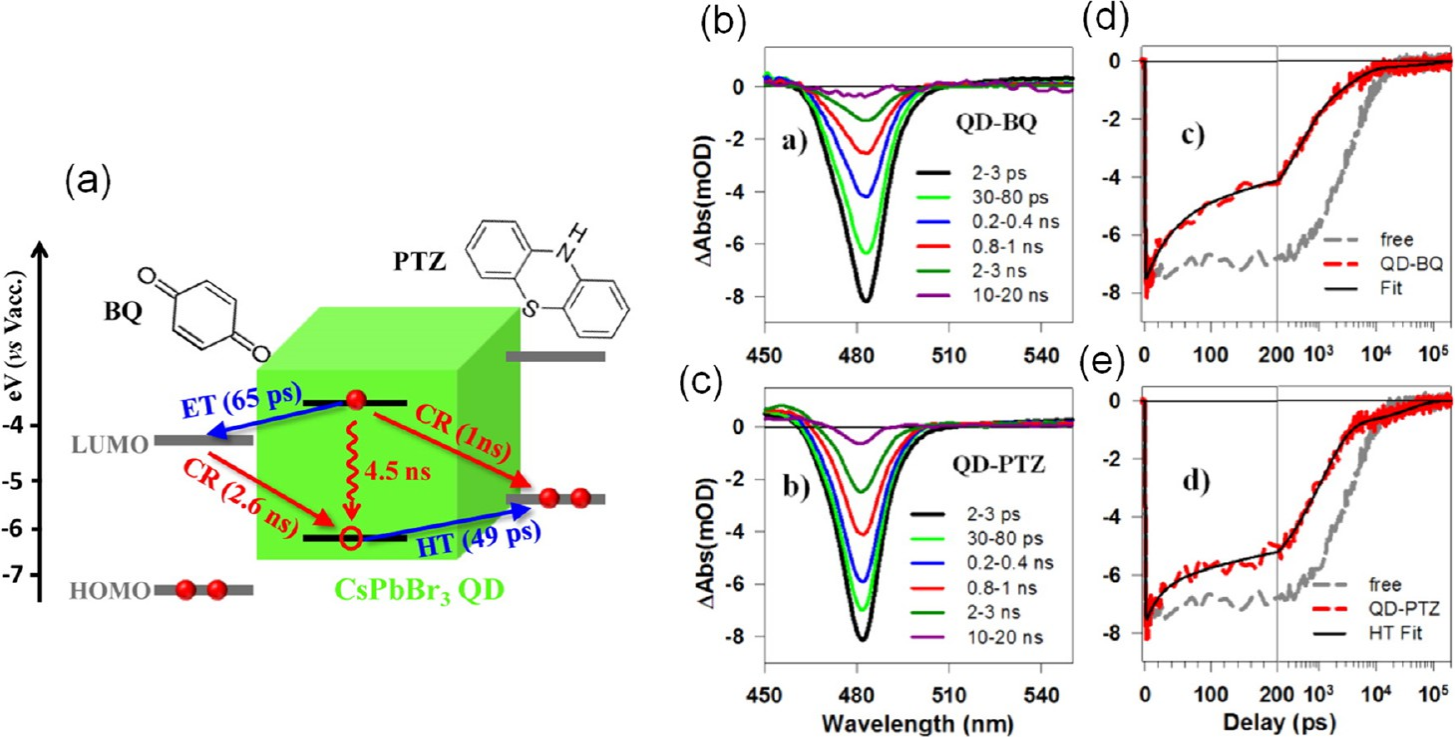
(a) Schematic energy level diagram of CsPbBr_3_ NCs-BQ/-PTZ complexes and possible charge separation and recombination channels. (b,c) TA spectra NCs-BQ and NCs-PTZ complexes at indicated time delays after 400 nm excitation. (d,e) Corresponding accelerated bleach recovery kinetics as compared with the free NCs (gray dashed line). Reproduced from ref (921). Copyright 2015 American Chemical Society.

Wu *et al.* investigated the electron and hole-transfer dynamics from CsPbBr_3_ NCs to traditional electron and hole acceptors, benzoquinone (BQ) and phenothiazine (PTZ), respectively (Figure 111a), by monitoring the bleach recovery kinetics of the NCs in presence and absence of the acceptors in ultrafast TA measurements.^921^ The bleach recovery kinetics of the CsPbBr_3_ NCs is accelerated in the presence of BQ/PTZ due to charge transfer from the perovskites (Figure 111b–e). Subsequently, several molecular acceptors such as fullerene, ferrocene, tetracyanoethylene, anthraquinones, 1-aminopyrene, *etc.* were used with a variety of perovskites.^912,922−931^ The time constants for charge transfer between different pairs are summarized in Table 2.

**Table 2:** Charge Transfer Dynamics between Various Pairs of Perovskite NCs and Molecular Acceptors Investigated through Transient Absorption Measurements (Unless Otherwise Mentioned)

system	carrier acceptor	carrier-transfer time (ps)	ref
electron transfer			
CsPbBr_3_	benzoquinone	65 ± 5 (half-life)	(921)
	benzoquinone^a^	20–50	(932)
	Rhodamine-B	600	(917)
	anthraquinone	30	(923)
	C_60_	190	(923)
CsPbI_3_	Rhodamine-B	40.6–872	(933)
	C_60_	18–45	(912)
hole transfer			
CsPbBr_3_	phenothiazine	49 ± 6 (half-life)	(921)
	phenothiazine^a^	137–166	(932)
	4,5- dibromofluorescein	1–1.25	(930)
	1-aminopyrene	∼120	(927)
	TIPS-Pc^b^	∼5	(929)
	4-mercaptophenol	∼14.1 ± 3	(934)
CsPbCl_*x*_Br_3–*x*_	tetracene carboxylic acid	7.6 ± 0.2	(935)
CsPbI_3_	1-aminopyrene	∼170	(927)

a Through terahertz (THz) measurements.

b Triisopropylsilylethynyl pentacene carboxylic acid.

The electronic coupling of the QD and acceptor orbitals influences both charge separation and charge recombination dynamics.^933^ It is shown that ∼99% photogenerated electrons can be transferred from CsPbI_3_ NCs to TiO_2_, with a size-dependent rate ranging from 1.30 × 10^10^ to 2.10 × 10^10^ s^–1^.^879^ Scheidt *et al.* investigated electron transfer between CsPbBr_3_ NCs and several metal oxides such as TiO_2_, SnO_2_, and ZnO.^936^ Formation of a long-lived (∼microseconds to milliseconds) species is observed in CsPbBr_3_/methyl viologen^2+^ system.^937^ A long-lived (5.1 ± 0.3 μs) charge-separated state for CsPbCl_*x*_Br_3–*x*_ perovskite–tetracence complex is also reported.^935^ Electron and hole transfer from CsPbBr_3_ nanoplatelets to BQ and PTZ with a time constant of 10–25 ps and a half-life time >100 ns of the charge-separated state in NPls-PTZ is also reported.^938^ To examine the dependence of the charge-transfer dynamics on the morphology of the perovskite NCs, Ahmed *et al.* studied electron transfer between tetracyanoethylene and the nanospheres, -plates, and -cubes of MAPbBr_3_.^924^ Electron transfer from photoexcited CsPbBr_3_ NCs to CdSe QDs and hole transfer from photoexcited CdSe to perovskites were studied.^939^ Charge transfer between CsPbBr_3_ NCs and CdSe QDs and NPls is also examined.^940^ The electron transfer from CsPbBr_3_ to 2D NPls is found to be faster as compared to the QDs due to larger surface area and greater density of states in 2D materials. There are also a few studies on charge-transfer dynamics between photoexcited non-perovskite semiconductors and perovskite NCs.^941−943^ Yao *et al.* studied the charge transfer and exciton diffusion process in bilayer and blend structures of CsPbBr_3_/PCBM interfaces.^944^ By varying the thickness of the CsPbBr_3_ NC film on top of the PCBM layer in the bilayer heterostructure, they determined an exciton diffusion length of 290 ± 28 nm for CsPbBr_3_ assembly. They concluded that the diffusion process in such cases is followed by an ultrafast exciton dissociation (within 200 fs) at the CsPbBr_3_/PCBM interface. Even an overall faster charge-transfer process was observed by them in the blend structures which revealed an effective charge extraction from the active layer resulting in a high photosensitivity.^944^

#### Triplet Energy Transfer

As the band-edge excitonic states of the perovskites possess both singlet and triplet characters,^945^ recent studies focused as well on harvesting the triplet exciton. The triplet exciton can be used for sensitization of molecular triplets that generates possibilities like room-temperature phosphorescence, triplet–triplet annihilation mediated photon upconversion, *etc.*(46,147,946−951) Several polyaromantic hydrocarbons with appropriate band alignment have been investigated in this regard.^952^ It is interesting to note the enhancement of triplet energy transfer (TET) efficiency with a decrease in NC size. For strongly quantum-confined (edge length of ∼3.5 nm) CsPbBr_3_ NCs, the TET efficiency is found to be as high as ∼99%, but for 11.2 nm sized NCs, no TET is observed.^952^ This is because for quantum-confined NCs, the electron and hole wave functions spread beyond the NCs surface that enhances the orbital overlap between surface-adsorbed triplet acceptors and the NCs. While direct observation of the formation of molecular triplets confirms TET, a recent study suggests that the mechanism can vary from system to system.^953^

#### Multiexciton Extraction

Extraction of multiexcitons prior to Auger recombination is an important process, which can push up PCE of the solar cells by manifolds. While extensive studies on harvesting multiexcitons from the metal chalcogenide quantum dots have been made,^954,955^ there are only a few similar studies with the perovskite NCs. Wu and co-workers demonstrated tetracene-assisted dissociation of up to 5.6 excitons per NC from CsPbCl_*x*_Br_3–*x*_ NCs.^935^ Multiexciton extraction from CsPbI_3–*y*_Cl_*y*_ using C_60_ has also been successfully achieved.^912^ In a recent study, it was shown that out of 14 excitons generated under high excitation fluence in CsPbBr_3_ NCs, approximately five electrons get transferred to surface-bound anthraquinones.^926^ As discussed earlier, Manzi *et al. showed* efficient multiexciton generation also takes place for below band gap excitation in the case of CsPbBr_3_ NCs.^908^ While this topic holds promises for further advancements, clearly it is in the early stages of development.

#### Hot Carrier Transfer

Extraction of hot charge carriers is a challenging task due to their rapid relaxation to the band-edge states. Only a few reports of HC extraction from perovskites are so far available.^850,932,934,956−958^ In an early work, transfer of hot electron and hot hole from CsPbBr_3_ NCs to BQ and PTZ was established by monitoring the photoinduced change in conductivity in time-resolved THz transmission.^932^ Li *et al.*(850) showed transfer of hot electrons from MAPbBr_3_ to 4,7-diphenyl-1,10-phenanthroline (Bphen) from the sharp drop in bleach amplitude at early time in the presence of the latter. The hot electron extraction efficiency is estimated to be ∼83% for ∼0.6 eV excess energy, and this efficiency progressively decreases with lowering of the excess energy. Hot hole extraction from MAPbI_3_ to spiro-OMeTAD has also been demonstrated.^957^ More studies on this important but challenging task are needed.

### Summary and Outlook for Optical Properties and Charge Carrier Dynamics

In conclusion, we have tried to review the fundamental optical properties in MHPs covering a broad range of topics. Though still, the stability of MHP NCs is a major issue which needs further improvement from their chemistry point of view for their future commercialization, it is also absolutely necessary to have understanding of their fundamental optical properties for their ultimate employment in the optoelectronic devices. One of the major ongoing debates in the field of MHP nanostructures is to understand the exciton fine structures which governs the radiative *versus* nonradiative rates significantly and it is essential for their light-emitting applications. Though initially it was believed that the lowest exciton state is bright in the case of MHP NCs, in later investigations, it is found to be opposite in many cases. As the transition metal ion doping in MHPs is a quickly emerging topic, it demands more in-depth understanding of the crystal field-induced splitting of bright *versus* dark excitonic states. Hence, a significant amount of research needs to be done in this direction. In addition to the lead-based MHPs, many lead-free MHPs such as double perovskites, 0D MHPs, are emerging as potential semiconducting material for white light generation from self-trapped excitons. The self-trapped exciton formation process in such materials is still not understood. The self-trapping process is highly nonlinear and strongly related with electron–phonon coupling.^809^ Hence, a considerable amount of research should be performed in this direction to understand the phonon dynamics in such material systems to unravel small polaron formations kinetics and the relevant photophysics of these systems. Hot carrier cooling in MHPs is also not fully understood where many theories like large polaron formation,^959^ acoustic to optical phonon up-conversion^960^ have been proposed so far. Recently it is shown by atomistic simulation that lattice vibrations is important in understanding the hot carrier cooling process in the case of MHPs.^961^ Thus, it is also crucial to understand the role of electron–phonon coupling for hot carrier’s extraction at the MHP/organic interfaces for the realization of hot carrier solar cells. Therefore, further research needs to be carried out in this direction. Multiexcitonic processes such as multi photon generation processes are important to increase power conversion efficiencies of solar cells by harvesting below band gap photons and to minimize above band gap excitation induced multiexcitonic annihilation processes such as Auger heating. This nonradiative process becomes dominant at high excitation densities and thus plays an important role in the nonradiative process in the case of high current driven LEDs and lasers. To get better understanding of those processes and how they control the efficiency of perovskite LEDs, such processes need to be monitored in detail in operational devices. The recent findings of ultrafast spin-relaxation dynamics in the case of MHP NCs may become beneficial for MHP-based spintronics such as spin LEDs and spin lasers. Chirally functionalized MHP shows room-temperature circular dichroism^20^ where a detailed understanding of the spin-dependent chirality transfer process in the photoexcited carriers needs more investigations.

## Optical Studies of Quantum Dots and Nano- and Microcrystals at the Single-Particle Level

### Photoluminescence Blinking in MHP Single NCs

**Figure 112:**
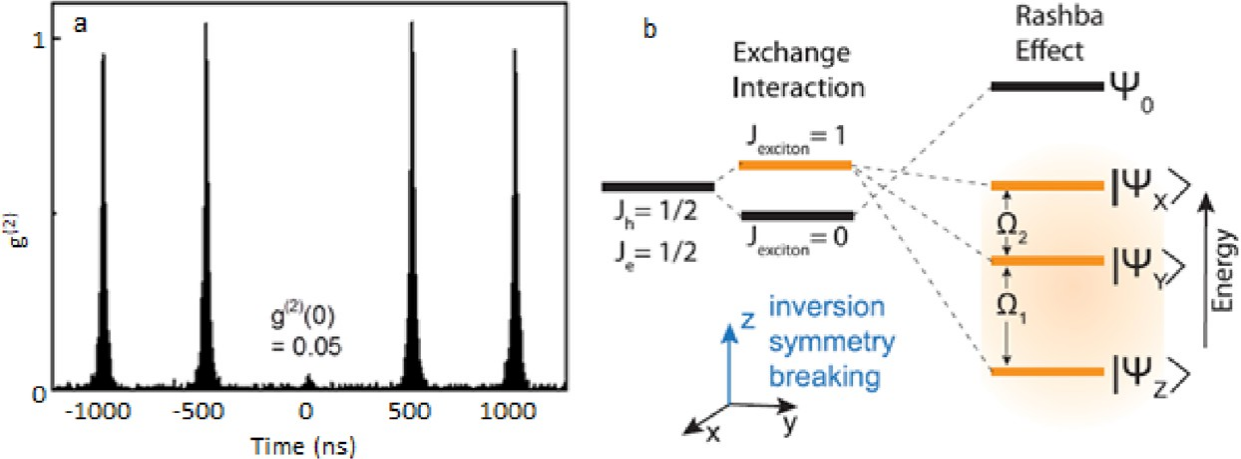
(a) Photon coincidence histogram of a CsPbI_3_ MHP QD under pulsed excitation. The low value of coincidence (0.05) at time zero represents single-photon emission. Reproduced from ref (26). Copyright 2015 American Chemical Society. (b) Splitting of exciton fine structure in a MHP QD after the breaking of the inversion symmetry of a CsPbBr_3_ QD owing to the Rashba effect, where *J*_e_ and *J*_h_ are the total angular momentum of electron and hole, Ψ_0_ is optically inactive singlet state, and |Ψ_x,y,z_⟩ are emissive triplet states. Reproduced with permission from ref (24). Copyright 2019 American Association for the Advancement of Science.

MHP NCs show properties similar to the conventional QDs based on cadmium or lead chalcogenides, such as broad absorption of light in the UV–vis–NIR region, size-tunable absorption and emission, and narrow-band, bright photoluminescence. Like conventional QDs, MHP NCs show stochastic fluctuations of PL intensity, also called PL intermittency or blinking. PL blinking varies with size, morphology, and composition of the MHP NCs, the nature and density of defects, intensity and energy of incident light, and the degeneracy of band-edge states. Quantum emitters are further characterized by the emission of a single photon within their PL lifetime. Recent studies show that the band-edge states of MHP NCs become nondegenerate due to the mixing of electron and hole states, exchange interactions of excitons and the Rashba effect.^24,147^ While the highest lying band-edge singlet state in MHP NCs is optically inactive due to inversion symmetry breaking of perovskite crystals, multiphoton emission from the low lying nondegenerate triplet states can occur.^147^ Hence, although single MHP NCs can be spatially isolated and studied, the exclusion of entangled photons from closely spaced band-edge triplet states (Ω_1_ and Ω_2_, Figure 112b) becomes necessary. Conversely, excellent antibunching (temporal separation) of photons from single MHP NCs at room temperature suggests that the degeneracy of the band-edge states increases with an increase in temperature, resulting in the maintenance of single-photon emission. Despite the complexity of the band-edge states and entangled photons, which are resolved at temperatures as low as 3.6 K,^147^ we focus in this section on the blinking behavior of single MHP NCs by referring to the intrinsic defects or traps, photoionization, and biexciton generation.

**Figure 113:**
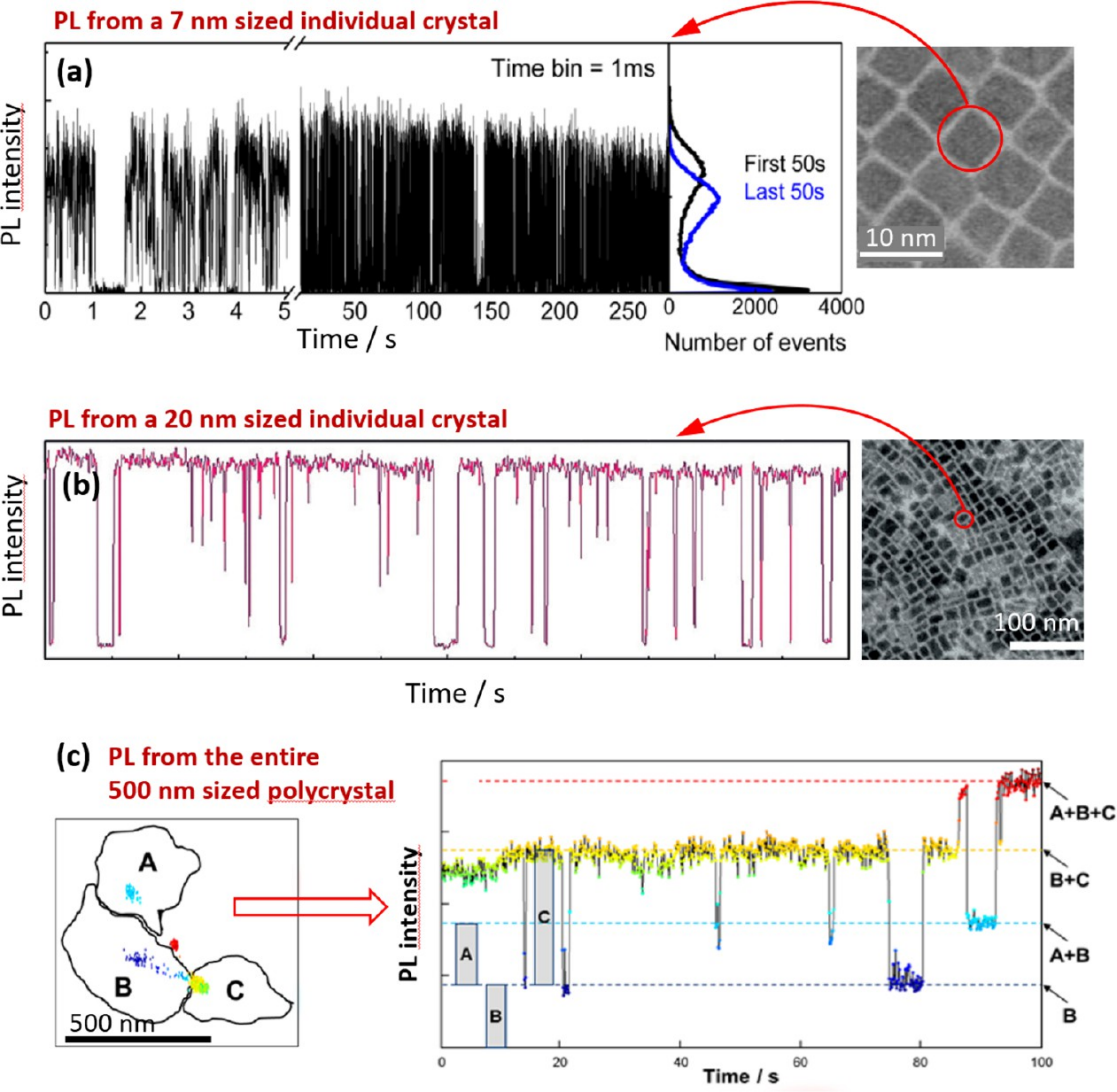
Single-particle PL intensity *vs* time of perovskite crystals with different sizes. (a) CsPbI_3_ NCs with a size of 7 nm. Reproduced from ref (26). Copyright 2015 American Chemical Society. (b) MAPbI_3_ nanocrystals with a size of 12 nm. Reproduced with permission from ref (962). Copyright 2019 Wiley-VCH Verlag GmbH & Co. KGaA, Weinheim. (c) MAPbI_3_ polycrystals with a size of 500 nm, where A, B, and C are three parts of the crystal. Reproduced from ref (963). Copyright 2017 American Chemical Society.

The strong quantum-confinement regime in NCs which are smaller than the exciton Bohr radius (<10 nm for MHPs), plays an important role in PL blinking.^24^ Hence, quantum size effects should be precisely considered during the analysis of single MHP’s PL. Differences in the MHP QD blinking behavior when compared to nano- and microcrystals are depicted in Figure 113;^26,962,963^ the multistate blinking of MHP nano- and microcrystals attributed to multiple emissive sites that are governed by metastable nonradiative recombination centers will be discussed in the next section. MHP NCs show, in addition to the distinct ON and OFF blinking behavior, also intermediate PL levels, similar to GREY states of conventional QDs.^964^

#### Blinking Mechanism

**Figure 114:**
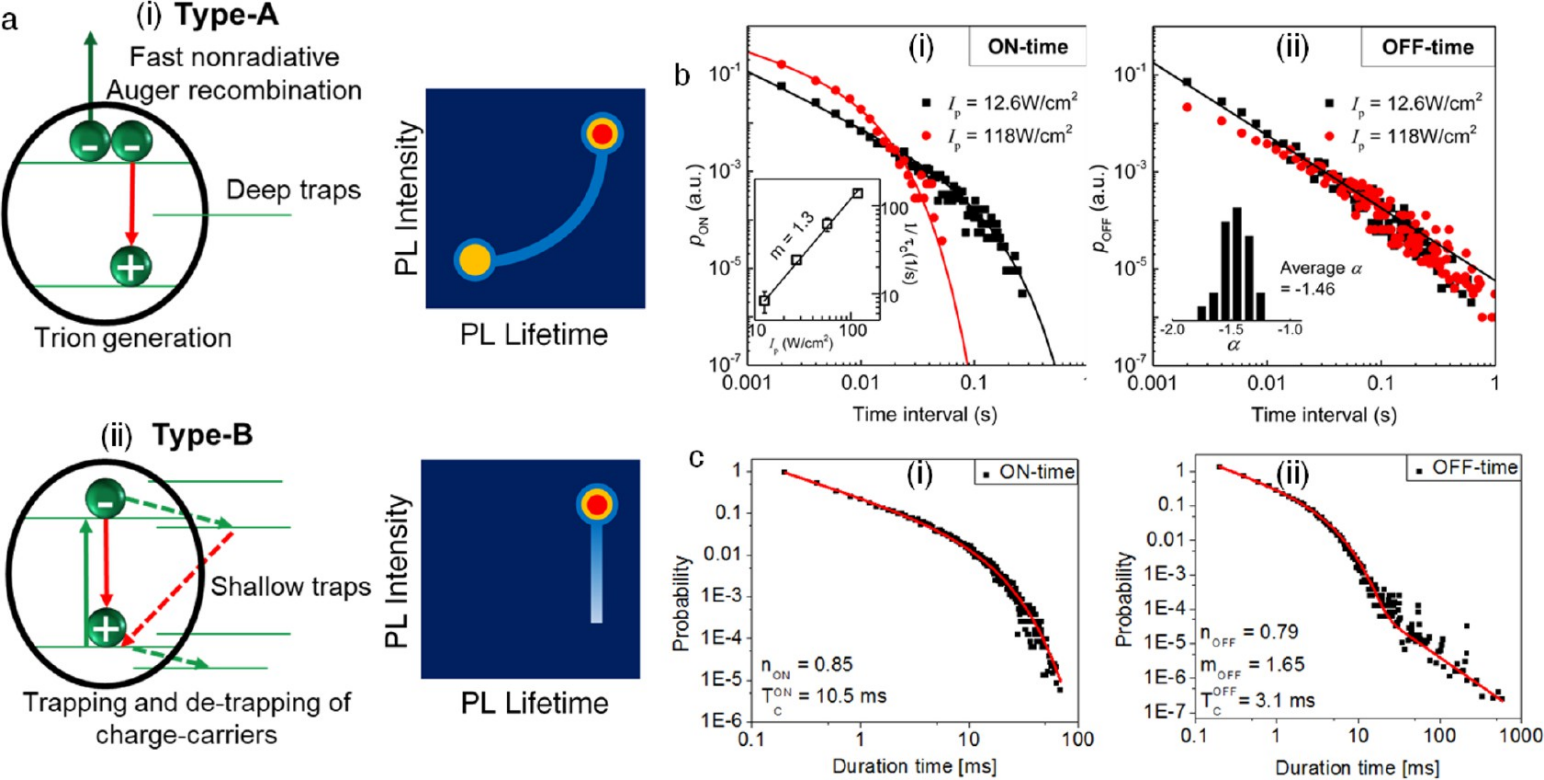
(a) Schemes correlating PL intensity and PL lifetime with the mechanisms of charge carrier dynamics for (i) type-A, (ii) type-B blinking. (b) Power-law functions showing type-A blinking of CsPbI_3_ NCs at two excitation pulse intensities. Reproduced from ref (26). Copyright 2015 American Chemical Society. (c) Power-law functions of FAPbBr_3_ NCs showing both type-A and type-B blinking. Reproduced from ref (966). Copyright 2018 American Chemical Society.

The ON and OFF periods during QD PL blinking correspond to the neutral and charged states with random switches between the states due to (dis)charging. Like conventional QDs, the blinking of MHP NCs can be assigned to type-A and type-B mechanisms,^964,965^ with random QD charging and discharging the key features of type-A blinking (Figure 114a(i)) and the activation and deactivation of trap or defect states in type-B blinking (Figure 114a(ii)). In type-A blinking, the PL lifetime decreases with a decrease in the PL intensity. ON and OFF time distributions trace the exponential power-law function, *p*_ON/OFF_ ∝ *t*^α^exp(−*t*/*t*_c_), where *t*_c_ is the truncation time and α is the power-law coefficient. In contrast, in type-B blinking, the PL lifetime does not change with variations in PL intensity, and the distributions of the ON and OFF times fit with the linear power-law function, *p*_ON/OFF_ ∝ *t*^α^.

Photoactivation of MHP QD surface defects or deep traps can produce a trion. In this scenario, after photoexcitation, a charge is transferred to the crystal shell, leaving behind a net charge with the opposite sign. Upon additional photoexcitation, the core of the QD will then contain three charges (trion state). Subsequently, the excited electron–hole pair will recombine nonradiatively by transferring their excitation energy to the extra charge *via* an Auger process instead of emitting a photon. Hence, one observes type-A blinking through repeated nonradiative Auger recombination, charge neutralization and radiative relaxation. For example, Park *et al*. have observed strong photon antibunching and type-A blinking in CsPbI_3_ NCs;^26^Figure 114b shows the ON and OFF time distributions associated to type-A blinking. Certain MHP NCs show both type-A and type-B blinking in tandem, as was shown for CsPbI_3_ QD by Yuan *et al.*(964) and FAPbBr_3_ QD by Trinh *et al.*(966) For example, the OFF time distribution of FAPbBr_3_ NCs follows an exponential behavior initially, which is the characteristic of type-A blinking (Figure 114c(ii)). After the truncation time, a linear behavior is followed, which is characteristic of type-B blinking.^966^ Type-A blinking of these NCs obeys the exponential nature of ON and OFF time distributions. The ON-time duration cutoff for FAPbBr_3_ NCs decreases with increasing excitation light intensity and saturates at ⟨*N*⟩ ≈ 1, whereas the OFF time distribution does not show such behavior. The switching from ON to OFF states takes place through either type-A or type-B pathway. However, MHP NCs turned OFF by ionization continue to be OFF until neutralized.

Trion and multiple exciton states, common to MHP NCs excited with high intensity/energy light, affect the PL quantum efficiency and induce frequent ON/OFF events in the PL trajectories. Like conventional QDs, biexcitons are generated in MHP NCs by mainly two mechanisms, (i) the absorption of two photons with an energy equal or higher than the band gap energy (*E*_*g*_) or (ii) the absorption of a photon with an energy equal or higher than 2*E*_*g*_. The biexciton can emit two photons by first going to the single exciton state and then to the ground state. The second-order correlation function depends on the PLQYs of the biexciton (*Q*_XX_) and single exciton (*Q*_X_) states. Under low intensities of excitation,  takes values close to zero, which is proportional to the ratio of the biexciton (τ_XX_) and single exciton (τ_X_) lifetimes and the ratio (β) of the corresponding radiative rates.^26,967^ Thus, the above equation can be rewritten as . Generally, if the statistics are scaled quadratically with radiative rates and the multiplicity of excitons, the value of β can be 4.

PL intensity transients of NCs show multiple intensity levels, which can be explained by the activation and deactivation of multiple recombination centres (MRC model).^968,969^ Li *et al.* described the relation of PL blinking to MRC and bright biexcitons.^967^ The activation and deactivation of MRCs govern the nonradiative recombination rate in a QD. This rate increases with an increase in the number of activated MRC, and as a result, the PL intensity and lifetime decrease. To account for the changes in PL lifetime and blinking at different intensities of excitation light, Li *et al.*(967) recorded the single MHP QD behavior at ⟨*N*⟩ = 0.02, 0.2, and 2. The PL blinking at ⟨*N*⟩ = 0.2 shows more frequent OFF states when compared with the blinking at ⟨*N*⟩ = 0.02. The blinking of an MHP QD shows the flickering effect at higher intensities of excitation light, suggesting switching between the bright and dim states. PL blinking at ⟨*N*⟩ = 2 is explained based on the activation and deactivation of MRCs and the charging and discharging of the trion state.

Apart from MRC, blinking due to nonradiative Auger recombination is correlated to the particle size. For example, the energy levels of larger MHP NCs are perturbed by the delocalization of the hole state throughout a QD.^970^ An increase in ON-time distribution with an increase in QD size suggests a low trapping rate and high de-trapping rate for larger MHP NCs. These rates can be extracted from the power-law coefficients of ON and OFF time distributions. The trapping and de-trapping rates depend on (i) photoionization of NCs, (ii) charge tunneling from a NC to a trap state, and (iii) the trapping time of electrons and holes. The nonradiative Auger recombination of trions becomes fast if an MHP NC is photoionized by trapping, which can be analyzed from the OFF time distribution and PL lifetime. The trapping and detrapping time of electrons and holes also affect the recombination rates; nonradiative recombination of the hole in a short-lived trapped state decreases the PL intensity and lifetime.

#### Blinking Suppression

**Figure 115:**
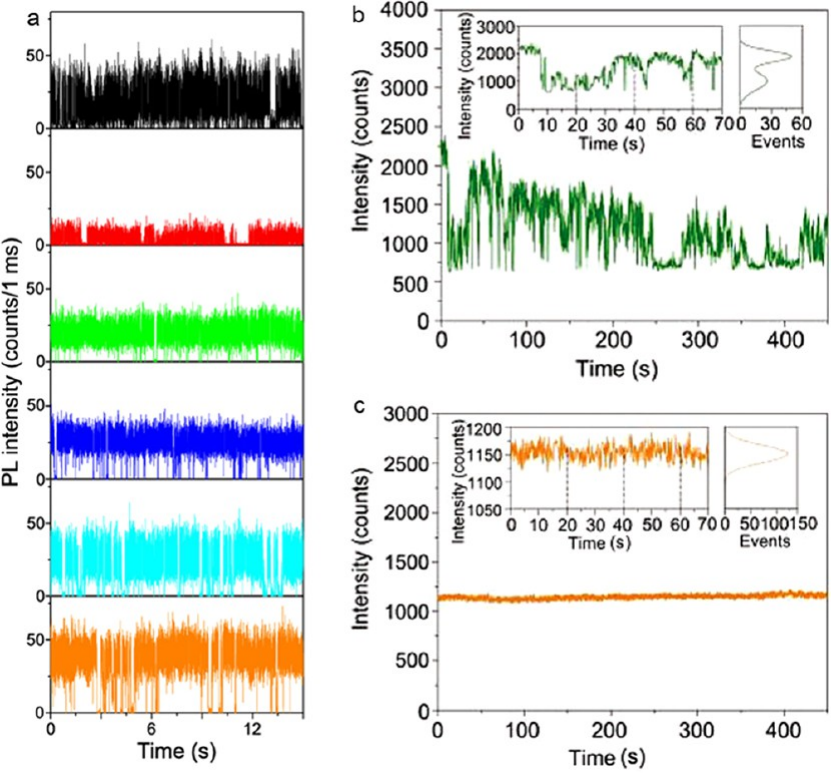
(a) PL intensity transients of a CsPbBr_3_ QD as a function of the bromide to iodide exchange reaction before (black), during (red, green, blue, cyan), and after (orange) the addition of PbI_2_ dissolved in a mixture of oleic acid and oleylamine. Reproduced from ref (972). Copyright 2020 American Chemical Society. (b,c) PL blinking of CsPbBr_3_ MHP NCs (b) without and (c) with a CdS shell. Reproduced with permission under a Creative Commons CC BY 4.0 license from ref (974). Copyright 2019 John Wiley & Sons, Inc.

The blinking behavior of MHP NCs may also depend upon the halide ion and A/B-site cation, halide vacancies, and surface defects.^882,914,971^ For example, with the exchange of bromide to iodide in CsPbBr_3_ NCs, Yoshimura *et al*. revealed a considerable increase in the ON time (Figure 115a),^972^ which should be attributed to not only the exchange of halide ions but also the filling of halide vacancies. These halide vacancy-assisted defects result in Type-A PL blinking, which can be suppressed by filling the vacancies. Chouhan *et al.*(973) demonstrated PL blinking suppression in real-time by supplementing MAPbX_3_ (X = Br, I) QDs with MAX. Also, blinking can be suppressed by the passivation of surface defects using shells. For example, Tang *et al.* demonstrated the suppression of the trap-assisted blinking in CsPbBr_3_ NCs by the preparation of CdS shells (Figure 115b,c).^974^ Here, blinking suppression is assigned to the passivation of deep electron or hole traps at the interface between the MHP QD core and CdS shell.

Although blinking of MHP NCs with different A-site cations is independently investigated by many groups, systematic single-molecule studies correlating the composition of A-site cation and blinking, are required to understand the role of A-site cation on blinking. Any differences in the blinking behavior due to differences in the composition at the A-site should be correlated with the dipole moment. Organic cations such as methylammonium (MA^+^) and formamidinium (FA^+^) ions are dipolar, whereas Cs^+^ is unipolar. When compared with Cs^+^ and FA^+^, the higher dipole moment of MA^+^ and its rapid motion within the lattice create a polaronic screening of the charge carriers. As a result, the exciton–exciton interactions are suppressed in MAPbX_3_. MA^+^ is also more susceptible to the fluctuations in the external charge and local current than Cs^+^ and FA^+^. Thus, the energy states in MAPbX_3_ or CsPbX_3_ can be modified by the quantum-confined Stark effect.^975^ Nonetheless, the exact relationship between blinking and A-site cation in an MHP QD is yet to be verified.

### Photoluminescence Blinking in MHP Single Crystals and Microcrystals

As outlined in the previous section, photoluminescence blinking on time scales up to seconds or minutes is an established phenomenon for single quantum systems such as molecules and classical QDs. Hence, the observation of blinking in larger MHP nano- and microcrystals was surprising, necessitating physical explanations beyond the mechanistic picture of blinking in quantum systems. In recent years, unraveling the underlying processes of blinking has become a topic of intense research. Even though full understanding of the physical picture is still absent, several key experiments have been carried out and yielded important information for the research on the origin of blinking. Moreover, blinking in spatially extended objects offers the distinctive opportunity to spatially resolve the intensity fluctuations and correlate them with the material’s morphology.

#### Pioneering Work and General Picture

The initial studies on blinking in MHPs emerged in 2015 and focused on larger sized MAPbI_3_ NCs and microcrystals (μCs),^700,976^ whereas MHP QD blinking was reported only a few months later.^26^ With their observation of blinking in 2–3 μm long MAPbI_3_ microrods, Zhu *et al*.^700^ reported for the time PL intermittency of MHP crystals larger than the diffraction limit of light. Tian *et al*.^976^ carried out more extensive research on the blinking phenomenon itself using polycrystalline MHP NCs. They suggested that the intensity fluctuations in such polycrystalline NCs are controlled by chemical or structural defects that trap-free charges.^976^ Due to their ability to quench the PL across surprisingly large volumes of MHP NCs and even μCs, Merdasa *et al.*(963) later termed these presumable defects “supertraps”. There is a clear analogy to large organic systems like conjugated polymers and aggregates, as in both cases the excited states are not delocalized over the whole volume (100 × 100 × 100 nm^3^ or larger); however, the excitations are very mobile and can travel over almost the entire system and potentially undergo nonradiative decay *via* an active quencher.

**Figure 116:**
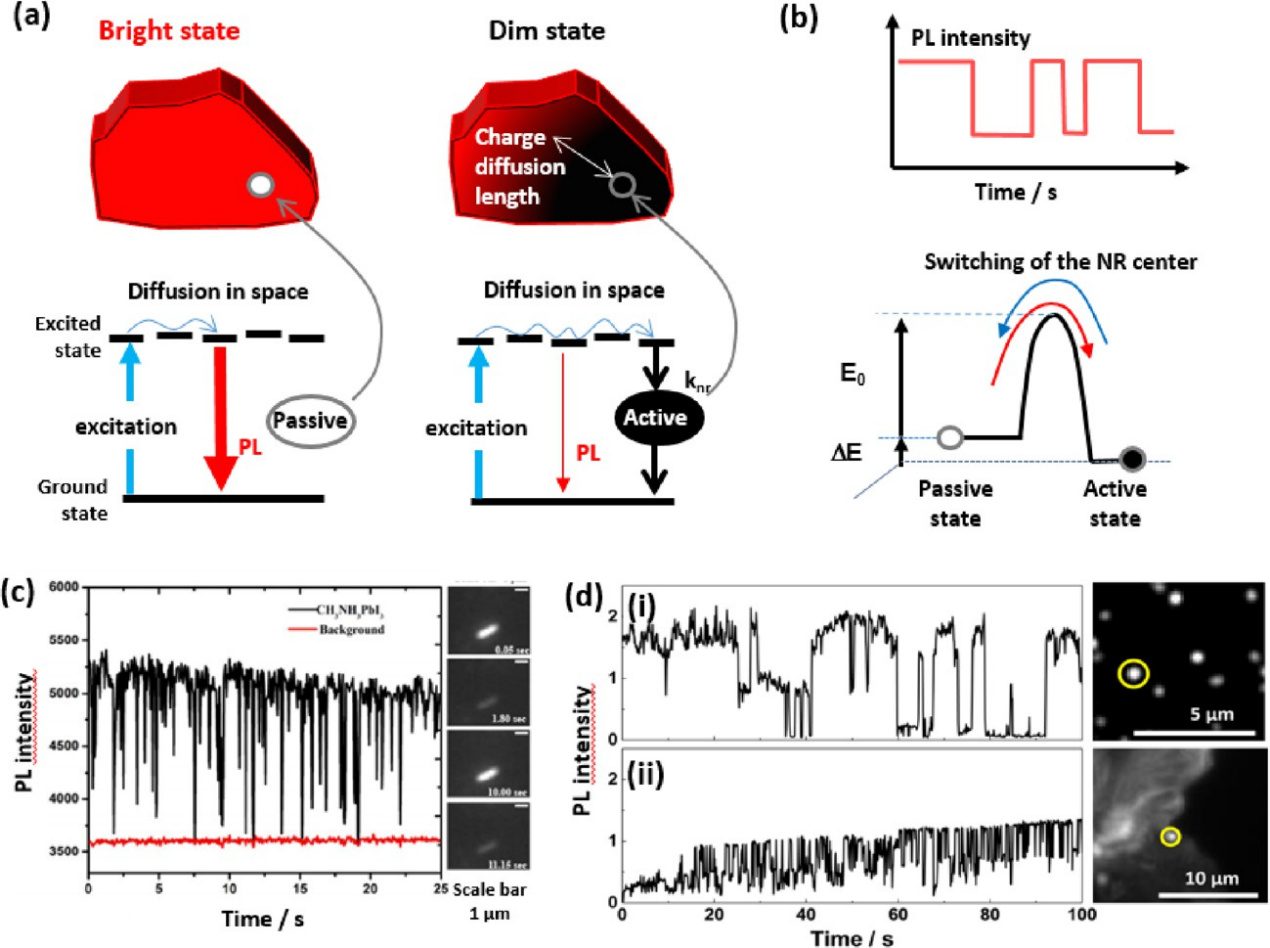
(a) Schematic illustration of the mechanism of a NR center in an MHP crystal and corresponding energy diagram schemes showing the sub-band-gap state formed by the defect. When passivated, the charge carriers freely diffuse until they recombine radiatively (bright state). When the NR center gets activated, a charge carrier can be “trapped” by the center due the typical long charge carrier diffusion lengths in MHPs, experiencing a trap-assisted nonradiative recombination (dim state). (b) Typical recorded PL transient and corresponding energy diagram illustrating the energy barrier for “on/off” switching of the NR center. (c,d) Pioneering blinking experiments showing PL intensity time traces for (c) a single MAPbI_3_ microrod (λ_exc_ = 540 nm, λ_PL_ = 700 nm). Reproduced from ref (700). Copyright 2015 American Chemical Society. (d) (i) MAPbI_3_ nanocrystal and (ii) a bright dot located on the top of a polycrystalline MAPbI_3_ crystal. Reproduced from ref (976). Copyright 2015 American Chemical Society.

Conceptually, this idea is similar to the model of MRCs proposed by Frantsuzov and Marcus.^968,969^ Originally, this model was invoked to explain the power-law distribution of switching times in QDs, which were inconsistent with the commonly accepted model of trap-assisted Auger recombination. As illustrated in Figure 116, the main idea is that the nonradiative rate fluctuates due to the ON/OFF switching of one or several metastable defects, leading to a time-dependent luminescence yield
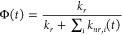
1where *k*_*r*_ denotes the radiative decay rate and *k*_*nr*,*i*_ is the time-dependent nonradiative rate constant for an active defect. In larger crystals, it is important to consider that Φ may also have some spatial dependence due to the spatial distribution of nonradiative recombination centers and a limited diffusion of photoexcited carriers toward these centers.

Tian *et al*.^976^ estimated the “quenching volume” of their polycrystalline MAPbI_3_ NCs to be >10^–16^ cm^3^ and the concentration of quenchers to be <10^16^ cm^–3^. From the saturation of blinking at high excitation power, the authors also estimated the capacity of the quenchers, *i.e.*, the maximum nonradiative recombination rate, to be 10^8^ s^–1^, corresponding to the quenching of one electron–hole pair per 10 ns. Later, similar estimates yielded quencher concentrations of 1.6 × 10^16^ cm^–3^ in the study of Gerhard *et al*.^977^ Hence, even for defect-rich polycrystalline MHPs of several hundred of nanometers in size, there is only a relatively small number of metastable quenchers per crystal.^977,978^ Note that the defect concentration is highly dependent on the synthesis procedure and the crystallinity of the formed MHP crystals, which is reflected in the variety of numbers reported here.

Similar to small NCs, Yuan *et al*.^979^ encountered power-law distributions of active and passive time periods exceeding 2 orders of magnitude upon blinking of large-sized MAPbI_3_ NCs. Moreover, Yuan *et al*.^21^ as well as Merdasa *et al*.^963^ confirmed the time fluctuations of the nonradiative rate by correlating the appearance of intermediate PL intensity levels in the blinking transient to faster PL decay. Faster PL decay in connection with a lower PL intensity is expected when the PL yield is modulated by a fluctuating nonradiative rate according to eq 1.

After introducing these NR centers, we would like to reiterate why the blinking in MHP nano- and microcrystals must have different underlying mechanisms to blinking in MHP NCs. As outlined before, PL blinking is commonly ascribed to the Auger process in colloidal QDs with sizes in the range of 2 to 7 nm. After the creation of a trion, subsequently excited electron–hole pairs will recombine nonradiatively by transferring their excitation energy to the extra charge *via* an Auger process, instead of emitting a photon. This “dark” state of the crystal lasts until the MHP QD turns back to the neutral state by recapturing the charge. Switching of the QD between the charged state and the neutral state can take several seconds and the process can therefore be easily framed.

For the Auger process to occur, the charges must be confined in a very small volume on the order of 100 nm^3^, which corresponds to a charge concentration of 10^19^ cm^–3^. This is exactly the regime of carrier concentrations when charge recombination in a bulk semiconductor is dominated by the Auger process. In MHP crystals with dimensions on the order of 100 nm length or larger, the carrier concentrations are orders of magnitude lower (10^13^ to 10^16^ cm^–3^). Even if the crystals become charged, the extra charges do not increase the carrier concentration close to the Auger regime. Reaching sufficiently high carrier densities is possible by choosing appropriate excitation conditions (> 100 W cm^–2^), however, this would not lead to “digital” switching of the nonradiative rate, because the process is masked by the high number of other recombination events. Therefore, a much larger volume requires a different mechanism to explain PL blinking. Moreover, the absence of the photon antibunching effect in MHP sub-micrometer-sized crystals recently demonstrated by Eremchev *et al*.^980^ rejects the simple Auger-based blinking mechanism. An alternative mechanism is trapping by a strong NR center, which is metastable and works at any excitation condition as long as the trap is not saturated. The only requirement is that the charge carrier should be able to diffuse over of the whole volume of the crystal to reach the center.

#### Origin of Metastable Defects

The idea of metastable NR centers has become the basis of the current understanding of PL fluctuations in MHP NCs. However, the chemical origin of the underlying defects has not yet been unraveled. It is important to note that the blinking phenomenon is not restricted to prototypical MAPbI_3_, but rather seems to occur in a wide variety of MHP compositions and morphologies.

Wen *et al*.^981^ reported blinking in local regions of a polycrystalline film comprising MAPbBr_3_ NCs, whereas intermittency in isolated NCs was suppressed. In that early work, the authors assigned the dim intervals to enhanced Auger recombination at interfaces between NCs in the film where charge carriers get accumulated in analogy to the blinking of aggregates of QDs.^26,964,966,970^ However, as we have discussed before, Auger recombination cannot be the primary origin of blinking in large crystals because of their size. Freppon *et al*.^982^ studied the PL intermittency in pure MAPbI_3_ and MAPbBr_3_ NCs, as well as in NCs with mixed-halide composition. The PL in the pure components was stable, while the mixed compounds showed pronounced blinking behavior, most likely due to (light-induced) iodide-rich and bromide-rich phase segregation. Tachikawa *et al*.^983^ on the other hand reported blinking for individual MAPbBr_3_ NCs, which was accompanied by light-induced PL enhancement. Halder *et al*.^984^ observed blinking in both pure and SCN^–^-doped MAPbI_3_ NCs, and Li *et al.*(985) demonstrated PL intermittency behavior in individual grains of mixed-halide MAPbI_3–*x*_Cl_*x*_ films. The above-mentioned studies confirm that the blinking phenomenon occurs in a plethora of MHP systems, which indicates that it is a general feature of MHP semiconductors related to the presence of a small number of metastable NR centers per grain/crystal^978^ rather than an effect, which is limited to certain material compositions or morphologies.

**Figure 117:**
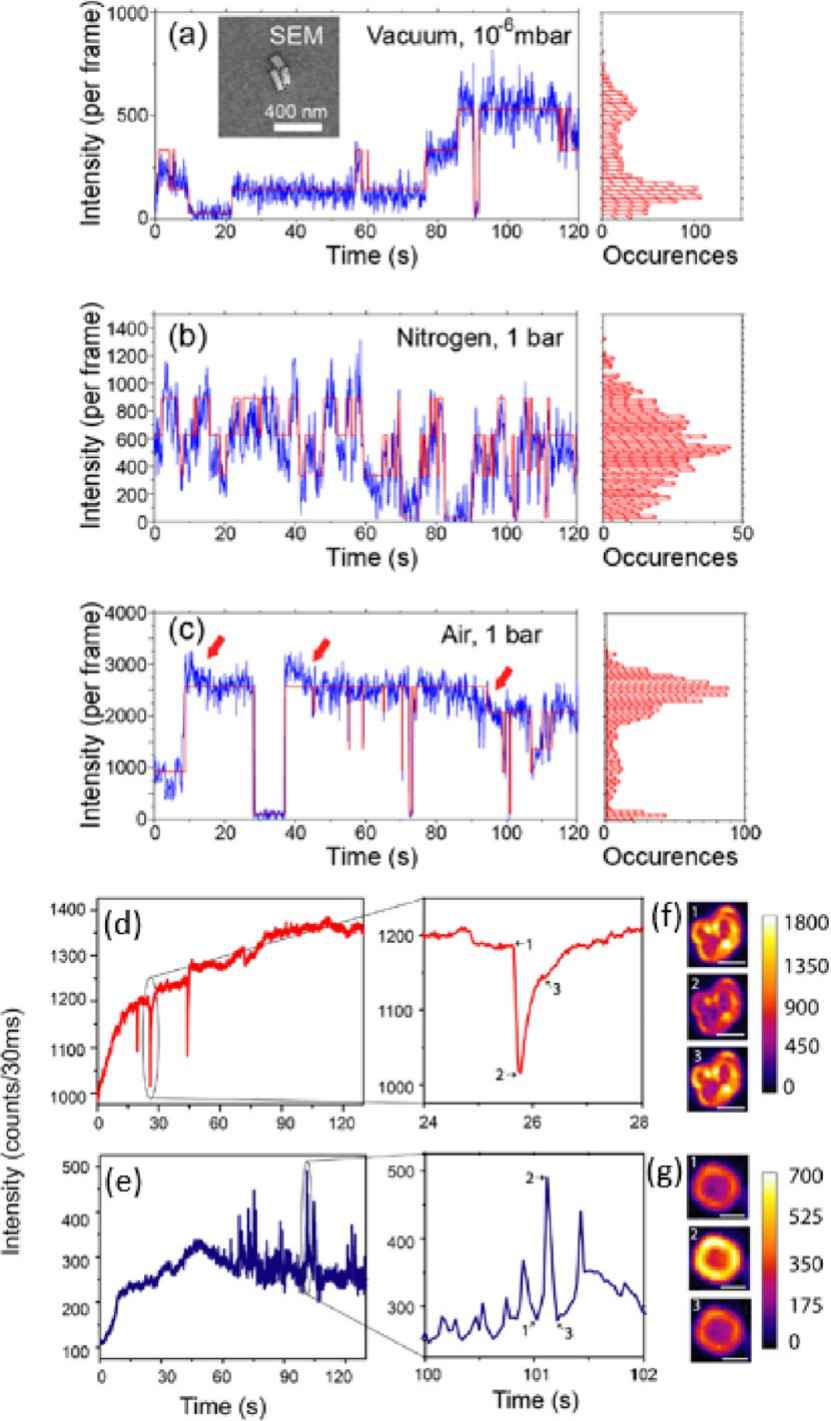
Effect of environmental conditions on PL blinking time traces of MAPbI_3_ nanorods (a) under vacuum, (b) in nitrogen under ambient pressure, and (c) in air under ambient pressure. The inset shows the scanning electron micrograph of the cluster of three perovskite nanorods. The red lines are a guide for the eye. Reproduced from ref (979). Copyright 2016 American Chemical Society. (d–g) Slow, gradual PL flickering of MAPbBr_3_ microcrystals under different humidity conditions. PL intensity time traces showing exceptional (d) sudden dim states under low humidity conditions (35–70% RH) and (e) sudden bright states under high humidity conditions (75–90% RH). (f,g) PL images of the particular microcrystals from (d,e) respectively, with timing of 1, 2, and 3. Scale bar = 1 μm. Reproduced from ref (989). Copyright 2016 American Chemical Society.

A very informative approach to comprehend the origin of metastable defects is the study of blinking in different atmospheric conditions, as they provide different reactive environments for trap formation and annihilation, in particular at the crystal surface. Yuan *et al*.^979^ investigated the environmental dependence of blinking in single-crystalline MAPbI_3_ nanorods and found pronounced differences in the blinking behavior under vacuum, nitrogen and ambient air (Figure 117). From this they concluded that most of the defects causing PL blinking must be located close to crystal surface. As potential candidates for the metastable defects they proposed under-coordinated Pb ions and MA vacancies. For the formation of the latter species, they argued that vacuum could promote detachment of MA due to its low boiling point, whereas the presence of oxygen, light and moderate humidity enable chemical reactions that promote passivation of surface defects. Passivation of defects under these atmospheric conditions has also been reported by Tian *et al*.,^986^ Tachikawa *et al*.,^983^ and Merdasa *et al*.^963^ who found an increase of the overall PL intensity and connected this to the emergence of blinking. Although, it is important to realize that fast diffusion of gases like oxygen through MHP crystals does not allow to assign the effect of atmosphere to surface modification only. The influence of surface defects on the PL are consistent with surface passivation studies leading to a significant improvement of the luminescence yield and optical stability.^983,987,988^ However, note that not all metastable defects were passivated, potentially because some of them are inherent structural defects, as pointed out by Yuan *et al*.^987^

Detailed studies on the influence of the ambient atmosphere on MAPbBr_3_ microcrystals were also carried out by Halder *et al*.^989^ In particular, they investigated the effect of high humidity and observed a lower PLQY in a humid atmosphere and the appearance of strong variations in the PL intensity looking like PL flickering. This process was found to be accelerated in the presence of oxygen. Upon the removal of moisture, the flickering disappeared, accompanied by a considerable enhancement in the overall PL intensity. It is important to note that the change in PL was usually gradual rather than abrupt, thus it cannot be explained by activation/deactivation of just one quencher. In this work, the slow PL flickering was assigned to a concerted phenomenon caused by several defects. Such defects may be induced *via* interaction with the environment, for example, transient chemical changes to the surface layer due to local fluctuations of humidity. So far, all these are pure speculations and further studies are needed to understand the nature of such large-scale gradual fluctuations. It is interesting to note that the emergence of PL flickering observed by Halder *et al*. was connected to an overall reduction of the PL intensity due to the humidity effect, whereas in the other reports mentioned above,^963,983,986^ PL blinking appeared after light-induced PL enhancement. This is a strong indication that the flickering phenomenon observed by Halder *et al*. under high humidity has a different origin from “real” blinking related to individual luminescence quenchers, which becomes more pronounced after PL enhancement potentially due to light-induced defect curing, which increases the diffusion length and with this the quenching volume of individual metastable quenchers.

Variations of the experimental conditions can also be employed to study whether blinking is a photo- or thermally activated process. Tian *et al*.^976^ and Yuan *et al*.^979^ studied the influence of optical power on the blinking characteristics. Both found a strong reduction of the relative blinking amplitudes, which was interpreted as a saturation of the metastable nonradiative center (trap filling) and an overall reduction of blinking events with increasing excitation power. Photoactivation, however, would become apparent as an increase of the switching dynamics. Hence, in recent studies there is no evidence for photoactivation of the switching process. However, trap filling effects could mask the photoactivation phenomenon and more suitable model systems, *e.g.*, smaller crystals, are needed to clarify this point.

By investigating the influence of temperature on luminescence blinking, Gerhard *et al*.^977^ provided a detailed view on the underlying mechanism of blinking in MAPbI_3_ NCs. After decreasing the temperature from 295 to 77 K, an increased time-averaged PL intensity by 1–2 orders of magnitude was observed as well as a substantial reduction in the relative magnitude of blinking below 200 K. Both the observed temperature-dependent PL intensity and the blinking dynamics were very specific from crystal to crystal and often fully repeatable in consecutive cooling–heating cycles. It was proposed that this peculiar behavior comes from the presence of several quenchers per crystal having potential barriers between active and passive states. Using a simple model, the activation energies of the switching of individual quenchers were found to be broadly distributed from 0.2 to 0.8 eV. This range matches the range of reported energy barriers for ion migration in perovskites. Therefore, it is likely that the random switching is caused by diffusing ions that can passivate or activate an NR center, whose energetic position is determined by the local environment.

Even though the above-mentioned studies revealed important insights into the processes that drive luminescence blinking, the nature of the underlying defects has not yet been defined. It is important to note that most MHPs possess defect levels close to the band-edges. Therefore, it is unlikely that they act as potent luminescence quenchers. However, as pointed out by Merdasa *et al*.,^963^ one should consider that the defects could also be complexes comprising an electron donor and an electron accepting species. This way, electrons and holes are efficiently trapped in close proximity, and their spatial overlap leads to fast nonradiative recombination. Additionally, this hypothesis seems to explain the relatively low estimated concentration of metastable NR centers.

### Super-resolution Methods to Unravel the Spatial Distribution of NR Centers

The fact that blinking in MHPs occurs in spatially extended objects offers the opportunity to obtain information about the location of quenchers and emissive sites. In this context, a particularly powerful approach is the combination of electron microscopy with super-resolution fluorescence microscopy,^963,979,987^ which allows for the direct correlation between the morphology of the material and the local emissive properties.

**Figure 118:**
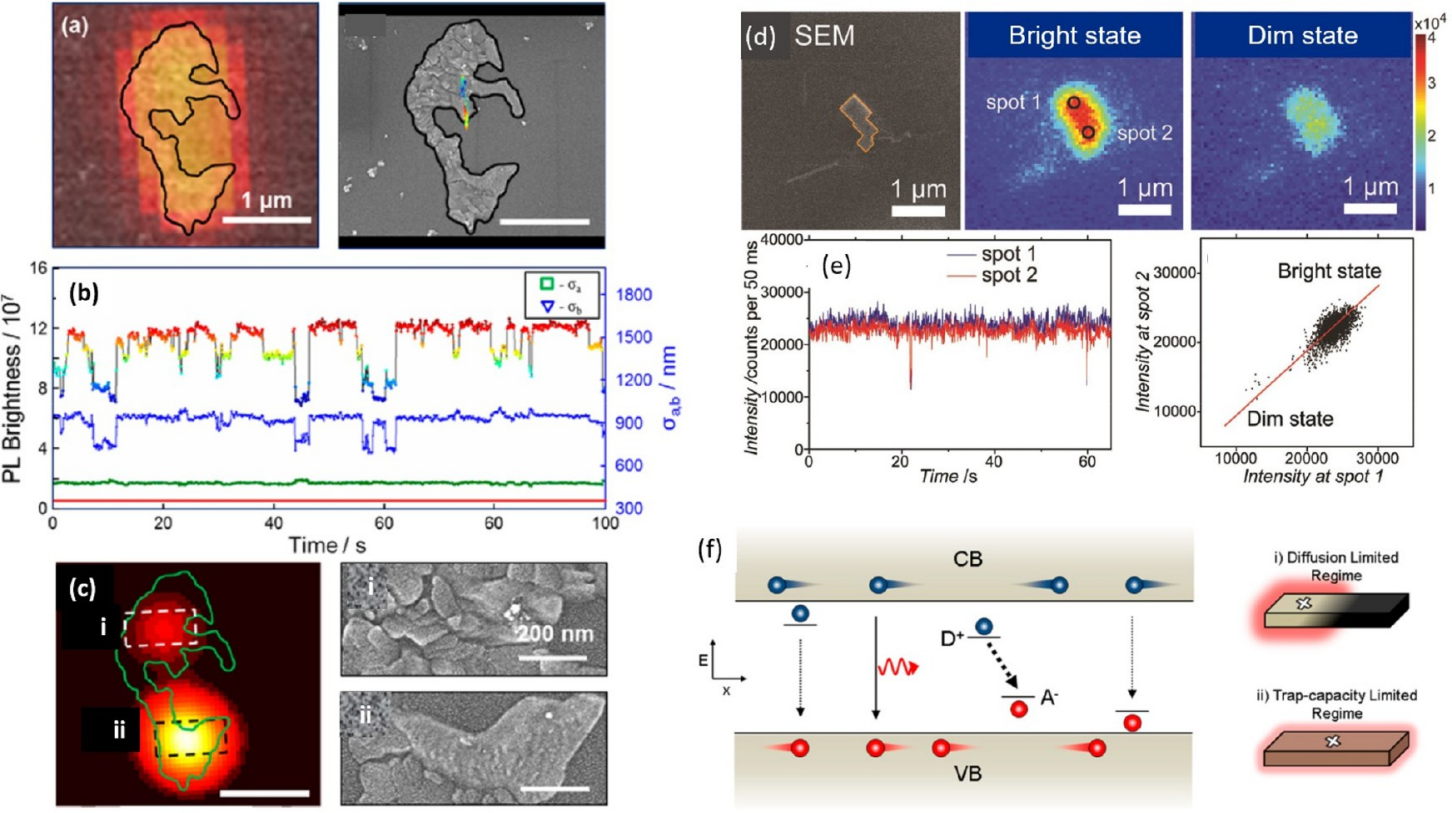
(a) PL emission profile of a large MAPbI_3_ polycrystal with its contour shown by the black line (left) and the emission localizations indicated on the corresponding SEM image (right). (b) PL intensity transient and time dependence of the Gaussian widths (σ_a,b_) indicating a fluctuating asymmetric emission profile. The red horizontal line at 360 nm is the σ_PSF_ of the microscope for λ = 760 nm. (c) SOFI image showing two well-separated spots (left) and their corresponding zoomed-in SEM images (right). Reproduced from ref (963). Copyright 2017 American Chemical Society. (d) SEM image of a polycrystalline MAPbI_3_ NC with a volume of about 9 × 10^6^ nm^3^ and the PL images in its bright state and dim state, respectively. (e) PL intensity time traces recorded at the two ends of the crystal (left) and scatter plot of the PL intensities at both ends showing a strong correlation (right).^987^ Reproduced with permission from ref (987). Copyright 2018 John Wiley & Sons, Inc. (f) Schematic illustration of a high-capacity NR center (supertrap) as a donor–acceptor pair. Left: Energy diagram schemes showing the sub-band-gap states formed by the defects. In the case of an ionized donor (D^+^) and acceptor (A^–^), the high-capacity NR center is created and efficient nonradiative recombination occurs (thick dashed line). If they are separated in space, nonradiative recombination is inefficient (thin dashed lines). Radiative recombination occurs across the band gap (solid line). Right: Different operational regimes of the high-capacity NR center as affected by its location (white crosses). Reproduced from ref (963). Copyright 2017 American Chemical Society.

In their study on monocrystalline MAPbI_3_ NCs, Yuan *et al*.^979^ recorded SEM images and employed a localization algorithm to track the center of the profile emission in the course of blinking. Interestingly, they found no change in the emission localization position upon blinking of single crystals. In contrast, for polycrystalline MHP NCs, Tian *et al.*(976) observed a clear correlation between the PL intensity fluctuations and shifts in the emission location. In the first case carrier diffusion through the whole crystal is very efficient and the extent of luminescence quenching is only limited by the capacity of the metastable defect. As a consequence, the PL of the crystal is spatially homogeneously quenched. In the second case charge carrier diffusion plays a crucial role such that quenching in some regions of the objects is more efficient than in other regions, leading to shifts of the emission location in the course of blinking. The existence of both quenching regimes was initially pointed out by Merdasa *et al*.,^963^ who presented an extensive study on PL blinking in polycrystalline MAPbI_3_ microcrystals (Figure 118a–c), as well as monocrystalline microrods up to 10 μm in length. The authors demonstrated experimentally clear examples of the diffusion-limited and the NR center capacity-limited blinking regimes, as illustrated schematically in Figure 118f. It was found that high-capacity NR centers, also termed “supertraps”, are most efficient in structurally homogeneous and large MAPbI_3_ crystals where carrier diffusion is efficient, which may pose limitations on the efficiency of perovskite-based devices. Furthermore, as can be found in Figure 118f, they have elaborated a scheme considering the high-capacity NR center or supertrap as a donor–acceptor pair.

Sharma *et al*.^990^ demonstrated electroluminescence blinking in aggregated CsPbBr_3_ NCs but noted the absence of blinking when the material was photoexcited. By employing a super-resolution technique, they found that the electroluminescence was emitted from only a few distinct spots in each aggregate. They attributed this to the fact that, in the case of electroluminescence, only a few of the agglomerated NCs are emissive due to funneling of the injected charges to the lowest energy levels. PL on the other hand resulted from collective excitation of the overall aggregates, hence, no fluctuations of individual NCs became apparent. Their work further exemplifies that a key requirement to observe blinking is a high interconnectivity between the emissive entities, or, in other words, efficient diffusion of a major fraction of the emissive population toward the quenching defect.

In addition to localization of the emission position, other techniques borrowed from the toolkit of super-resolution methods have been employed to study the blinking dynamics. Tian *et al*.^986^ utilized a differential super-resolution technique to spatially map the regions characterized by intense PL blinking in a polycrystalline MAPbI_3_ film and found that the emission predominantly stems from very localized sites of less than 100 nm in size. It was hypothesized that an emitting site can be either a small crystallite free from quenchers or a spatially localized state in a large crystal with increased radiative recombination probability. Merdasa *et al*.^963^ employed super-resolution optical fluctuation imaging (SOFI) to detect local regions with strong and frequent blinking in polycrystalline MAPbI_3_ NCs. As depicted in Figure 118d,e, Yuan *et al*.^987^ used a differential imaging approach similar to that of Tian *et al*., from which they determined a heterogeneous distribution of fluctuating quenchers in mono- and polycrystalline objects comprising MAPbI_3_. Interestingly, the authors demonstrated that even a micrometer-sized polycrystal comprising several well-defined cubic sub-micrometer crystals can generate one common PL blinking time trace which is not limited by diffusion.

The combination of SEM with super-resolution fluorescence allowed furthermore to directly correlate the location of the NR center to a specific blinking volume, allowing to precisely define the density of NR center. As such, Merdasa *et al*.^963^ estimated a 10^9^ s^–1^ recombination rate introduced by a single quencher (supertrap) and Yuan *et al*.^987^ obtained quencher densities of 8.5 × 10^13^ and 1.3 × 10^14^ cm^–3^ for monocrystalline and polycrystalline NCs, respectively. The discrepancies in the reported defect concentrations highlight once more the importance of the material quality. Additionally, the crystal morphology and size may play a role. For smaller crystals, defects with a smaller capacity cause detectable PL blinking, while in larger crystals their influence can be suppressed because they get saturated at the same excitation power due to larger number of electron–hole pair generated in the crystal.

### Electron–Phonon Coupling in Single Perovskite NCs

The intrinsic (photo)physical properties of MHP semiconductors are strongly related to the coupling of excited electronic and vibrational states.^991^ The vibrational modes in MHPs can be generally split into two branches:^992,993^ a low-energy band (20–200 cm^–1^) dominated by the inorganic [PbX_6_]^4–^ sublattice, along with the high-energy vibrations of the organic components (200–3300 cm^–1^). In all-inorganic systems, like the CsPbX_3_ perovskites, the high-energy branch is missing. Following the absorption of above band gap light,^994^ the thermalization, transport, and recombination of photogenerated carriers will depend on the underlying electron–phonon interactions. For instance, stronger electron–phonon scattering in lead-based [PbX_6_]^4–^ octahedra directly reduces carrier mobility and increases the PL emission Stokes shift and line width (*i.e.*, color purity). At relatively low temperatures (below ∼50 K), scattering from low-energy acoustic phonons is dominant, while closer to RT Fröhlich coupling with high-energy longitudinal optical phonons (*E*_LO_ = ℏωLO) is the principal scattering mechanism in polar MHPs.

Analysis of the PL fwhm between 0 K and RT has become routine for gauging the strength of electron–phonon coupling within MHPs and comparing its magnitude across different compositions.^795,995^ The temperature-dependent excitonic line width of band to band recombination within semiconductors^795,798,996^ is related to the carrier-phonon coupling by
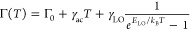
2The first term (Γ_0_) represents the intrinsic low-temperature fwhm, while the second and the third terms (Γ_ac_ and Γ_LO_) describe acoustic and LO–phonon (Fröhlich) scattering contributions, respectively, with coupling strengths γ_ac_ and γ_LO_. Below 75 K, the linear Γ_ac_ component dominates due to low-energy acoustic phonons. The LO–phonon population requires more thermal energy to become impactful, being governed by Bose–Einstein statistics, with *k*_B_ being the Boltzmann constant.

Studying single MHP NCs also allows one to investigate electron–phonon interactions beyond the bulk approximation. In the absence of strong thermal broadening close to 0 K, electron–phonon coupling in MHP NCs can manifest additional satellite peaks in the high-resolution PL spectrum, appearing as low-energy phonon replicas.^997,998^ These additional peaks correspond to weak phonon-assisted transitions and are red-shifted relative to the central zero-phonon PL (ZPL) emission. The relative intensity of phonon replicas between different NCs of different sizes will vary.^999^ Whereas low-temperature PL spectroscopic studies are widely adopted to probe electron–phonon interactions in MHPs, relatively few studies have focused on single MHP NCs. At the nanoscale, perovskite crystals tend to exhibit higher phase stability, preferring to occupy the desired perovskite structure,^3^ allowing more complete low-temperature optical studies. Furthermore, for micro-PL studies on single MHP NCs, the emission fwhm is substantially reduced^997,1000−1003^ (≤1 meV) compared to ensemble NC studies, better revealing fine energetic structure.

#### Temperature-Dependent PL

**Figure 119:**
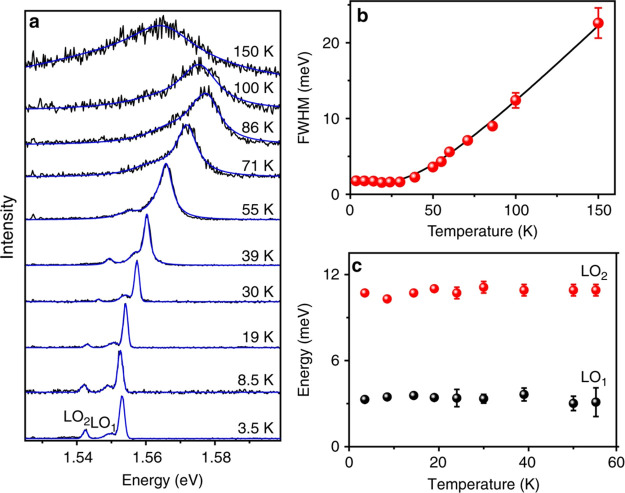
Temperature-dependent PL (a) spectral evolution and (b) zero-phonon PL (ZPL) line width (fwhm) of a single FAPbI_3_ NC. The black line is a fitting curve made using eq 2, taking into account only the low temperature line width (Γ_0_ = 1.5 meV) and Fröhlich coupling contributions (*E*_LO_ = 10.7 meV). Broadening due to acoustic phonon scattering is found to be negligible. (c) LO_1_ and LO_2_ phonon energies as a function of temperature from 3.5 to 55 K, recovered from (a). Reprinted with permission under a Creative Commons CC BY 4.0 license from ref (997). Copyright 2018 The Authors.

In Figure 119a Lounis *et al*.^997^ examine the thermal evolution of the exciton-phonon coupling phenomena in individual FAPbI_3_ NCs. Interestingly, below 30 K, they found negligible thermal broadening in the ZPL emission from a single FAPbI_3_ NC (Figure 119b), which suggests a weak electron–acoustic phonon interaction. An upper limit of γ_ac_ ∼ 5 μeV K^–1^ is extracted from eq 2 from their temperature-dependent broadening, which is found to be over 1 order of magnitude smaller than that previously reported for bulk FAPbI_3_.^795^ Thus, using a single optical phonon mode is enough to reproduce the thermal-induced broadening evolution in Figure 119b (parameters: Γ_0_ = 1.5 meV, γ_ac_ = 0 meV, γ_LO_ = 27 meV, and *E*_LO_ = 10.7 meV). While the optical phonon energy appears to be softened in the NC, the γ_LO_ broadening coefficient derived is in agreement with measurements on bulk FAPbI_3_.^795^ Due to the location of the A-site cation within the charged octahedral cavity formed by the BX_3_ sublattice, MHP NCs exhibit a soft ionic structure which endow them with so-called “crystal–liquid duality”.^1004^ More specifically, this glass character arises from the crystalline-like response of coherent band transport and a liquid-like response in the dielectric function. Hence, Fu *et al*.^997,1005^ assigned the derived smaller γ_ac_ value to the phonon glass character of the soft perovskite lattice and the larger bulk values to extrinsic influences (counterintuitive to expected confinement effects^1005^), rather than intrinsic electron–phonon interactions.

Below roughly 60 K, the appearance of additional phonon replicas are also resolved in the single FAPbI_3_ NC^997^ PL spectrum, assigned to different bundles of separated low-energy lattice modes (Figure 119c). On the basis of theoretical predictions and low-temperature vibrational studies of APbI_3_-based systems, they assign these phonon replicas to different bundles modes which are seen to be thermally stable in Figure 118c. Up to three additional satellites were resolved during their survey,^997^ being governed by different bending and stretching modes of the PbI_6_ network and motion of the organic FA cation, and by their mutual couplings.

Through PL studies of individual all-inorganic CsPbBr_3_ microwires at cryogenic temperatures (77 to 300 K), Zhao *et al*.^1006^ revealed the electron–phonon interactions arising in wires ranging from 0.5 to 5 μm thick and up to hundreds of microns long. They found that the PL spectrum exhibited a dominant green (527 nm) triplet exciton emission with an additional low-energy shoulder (∼540 nm) which became better resolved at lower temperatures, due to a replica emission. Fitting the thermal-induced broadening of the ZPL emission down to 77 K in the single CsPbBr_3_ microwires, they extracted an LO–phonon coupling constant of γ_LO_ = 66 meV^1006^ using a thermally stable phonon energy^996^ of *E*_LO_ = 19 meV, as derived from the single-crystal Raman scattering spectrum. This value is comparable to other bulk lead-bromine-based perovskites^795^ and confirms the preservation of strong Fröhlich interactions in their single CsPbBr_3_ microwires, arising from relatively weak confinement effects, *i.e.*, due to the relatively large NC dimensions.

Rainò *et al*.^1000^ reported one of the low-temperature PL studies of single MHP NCs, examining individual all-inorganic CsPbX_3_ (X = Cl/Br) NCs. Beyond the interesting blinking phenomena exhibited by the NCs, spectra measured from a single particle using sufficiently high optical excitations contained an additional low-energy peak, arising from a charged excitonic emission. Measured at what they define as intermediate excitation power,^1000^ the charged exciton line of some NCs became 2–3 times narrower than the principal exciton line, suggesting that the excitonic transition might have reduced electron–phonon coupling. At the single FAPbBr_3_ NC level, Pfingsten *et al*.^1001^ examined exciton–phonon interactions *via* temperature- and polarization-dependent PL measurements. Near 0 K, pronounced satellite PL peaks appear shifted relative to the ZPL band due to the TO_1_, TO_2_, and TO_3_/LO_1_ phonon bands, by energies of 4.3, 8.6, and 13.2 meV, respectively. Through their survey of multiple individual NCs, some extra replica peaks sometimes appeared, red-shifted by higher energies (18.3 and 37.2 meV) relative to the ZPL.^1001^ Based on the expected low-energy vibrational modes of the PbBr_6_ octahedra, they attribute these additional emission peaks to coupling of charge carriers to libration modes of the FA^+^ cations. Fitting the temperature-dependent fwhm of the ZPL with eq 2, Pfingsten *et al*.^1001^ also inferred a negligible contribution from acoustic phonon coupling (γ_ac_ < 0.1 meV) and identified thermal-induced broadening to principally arise *via* an optical phonon coupling constant (γ_LO_) of roughly 32 meV. Notably again, the optical phonon contribution is recorded here to be relatively low compared to other bulk Br-based MHP counterparts.^795^

Employing low-temperature (down to 5 K) polarized PL studies of CsPbBr_3_ single NCs (∼7 nm), Ramade *et al*.^1002^ also found that the temperature-dependent PL line width is mainly governed by the Fröhlich term (γ_LO_ = 42 ± 15 meV), being consistent with the polar nature of the bulk lead-halide perovskite.^795^ Within this regime (*i.e.*, NCs exhibiting band gaps of 2.46–2.62 eV), they found no correlation of the crystal size, for NCs in the order of the Bohr diameter, with the PL broadening due to acoustic phonon coupling. Liu *et al*.^1003^ reported single-dot PL measurements of MAPbI_3_ NCs (∼7 nm) down to 5 K, realizing the narrowest ZPL line width of ∼0.6 meV ever managed in the archetypal perovskite system. They also noted a sharp satellite peak that was red-shifted by ∼4 meV in low-temperature spectra, which varied in relative amplitude between dot to dot, pointing to variation in their exciton-phonon coupling strengths.

### Summary and Outlook for Single-Particle Studies of MHP NCs

Photoluminescence blinking of MHP NCs hampers the application of these bright luminescent crystals in quantum optical devices. Spectrally and temporally correlated single-photon counting through single-molecule microscopy and spectroscopy have been helpful for the classification of the emitting states and the blinking mechanisms. Although single NCs emit entangled photons with slightly different energies from the band-edge triplet states which become nondegenerate when cooled significantly, the degree of degeneracy increases with temperature and the second-order single-photon correlation function minimizes at room temperature. Thus, the blinking mechanism of MHP NCs is dissected at room temperature. Photo-charging followed by nonradiative Auger recombination is the primary mechanism of blinking in metal-halide perovskite NCs and metal chalcogenides. Here, blinking is due to the random switching of a NC between nonradiative and radiative cycles by charging and discharging. Also, MHP NCs show blinking due to trap-assisted nonradiative carrier recombination involving surface traps, deep traps and shallow traps, governed by ion vacancies, interstitial sites and antisites. Hence, post-synthetic chemical modification of MHP NCs allows for blinking suppression. Nonblinking MHP NCs for applications in nanophotonic quantum devices can be developed by optimizing the energy and intensity of excitation light, the nature and density of trap states, the size of quantum dots and the chemical composition of cations, halide ions, ligands, and shells.

The growing number of studies related to PL intermittency in MHPs indicates that the phenomenon is an intrinsic feature of this material class rather than an effect related to specific processing conditions or a specific environment. Furthermore, it is important to note that blinking in crystals with sizes beyond quantum confinement (up to several micrometers) goes mechanistically beyond the physics and chemistry of single quantum systems. A consistent explanation for blinking in MHP nano- and microcrystals can be given by the presence of metastable nonradiative centers. Metastability of defect states is in fact reflected in many phenomena observed in MHPs and related devices, for example PL enhancement and suppression, ion migration, self-healing after photodegradation, dropping and recovery of solar cell efficiencies^1007^ and the sensitivity of these processes to light illumination, atmospheric constituents and temperature. Thus, it is plausible that PL blinking is another manifestation of the metastable character of incorporated defect states. Except for PL blinking, however, all defect-related phenomena are ensemble observations, where contributions of individual defects are averaged out. This averaging is unavoidable because of the very large number of individual species in the volume, which can be described by the concentration *n*. Now, let us hypothetically decrease the volume of the sample to 1/*n*. Following Poissonian statistics, a crystal of this volume should contain on average 1 NR center, and its metastability becomes apparent as discrete blinking. To investigate this individual defect, methods of luminescence microscopy and in particular techniques inspired by super-resolution methods and single molecule spectroscopy are ideal tools. The resolution of optical microscopy is about 500 nm, which is equal to the typical grain size in polycrystalline films. Moreover, isolated crystals of sizes from 10 to 1000 nm can be readily investigated as model systems representing individual constituents of a perovskite film. Studying individual defects incorporated in these objects allows us to rationalize fundamental properties behind solar cells and other devices.

Furthermore, correlating PL and electron microscopy allows estimating the quenching volume and defect concentration. Taking the inverse of the concentration, we obtain the cube-shaped volume containing only one defect to range from 10^–10^ to 10^–17^ cm^3^, giving cube side lengths from 4.6 μm to 21 nm. Grain sizes in MHP films vary over the same range, hence, a number on the order of one defect per grain appears reasonable. This estimation is nicely supported by the long list of experiments discussed above where strong PL fluctuations have been reported for MHP crystals of very different sizes up to micrometers. Note that in order to observe blinking, it is not necessary to have exactly one defect per crystal. Additionally, defects with the strongest quenching capacity will be more visible in the case of many defects contributing. Increasing the number of defects, however, will make the blinking transients more complex and eventually reduce the overall modulation of the luminescence yield, such that a number much higher than one appears unfeasible. Despite the uncertainty in determining the actual concentration of metastable quenchers, the current stage of experimental work indicates that there is a high variety in densities. Likewise, literature is filled with very divergent estimations of the defect state concentrations in MHPs based on distinct techniques, ranging from 10^10^ to 10^17^ cm^–3^, which may be related to diversity from poly- to monocrystalline crystals, different detection techniques, and different methods of data analysis. However, we note that it is a remaining open question whether blinking studies and other methods are actually sensitive to the same type of defects, whether or whether not being (high-capacity) NR centers.

Despite a growing number of studies related to blinking in MHPs, several questions regarding the phenomenon of blinking in MHP NCs and μCs remain open. These include in particular the nature of the metastable quenchers and the mechanism of their activation and deactivation. It has also not yet been studied whether the switching process can be activated by light. Better understanding of the origin of blinking can open channels to passivate the quenchers permanently, which will be beneficial for the performance of MHP devices. Another interesting question is which fraction of the defect states in MHPs is metastable. The defect concentrations estimated from blinking experiments yield defect concentrations similar to the range reported from other methods, suggesting that the defects probed in blinking experiments are actually representative for a high fraction and maybe even all of the defects in the material. Furthermore, micro-photoluminescence studies on individual MHP NCs reveal high-resolution information on the nature and extent of charge carrier–phonon coupling in these systems, which are not averaged out by bulk measurements. Much deeper understanding of these photophysical processes can direct material development ensuring optimized charge dynamics with the aim to further design high-performance MHP NC-based optoelectronic devices.

## Applications

### Lasers

Since the initial observation of stimulated emission (SE) and lasing from colloidal perovskite quantum dots (perovskite NCs), there has been a surge in research activities in developing high-performance perovskite NC-based lasers because perovskite NCs combine the advantages that can be derived from both colloidal NCs and halide perovskite materials, namely facile processability from solution, band gap tunability, large absorption cross-sections and low non-radiative recombination rates.^28,1008−1018^ In general, there are two kinds of halide perovskite NCs, that is, the organic–inorganic hybrid halide perovskites (OIHP) NCs and the all-inorganic halide perovskites NCs (IHPNs). The IHPNs manifest better stability against moisture and oxygen than OIHP NCs since the organic compounds tend to dissociate when exposed to ambient conditions. Until now, both the IHPNs and OIHP NCs have shown excellent optical gain performance and were used in a variety of laser devices, including random lasers,^464,1019^ whispering-gallery-mode (WGM) lasers,^1020^ distributed feedback (DFB) lasers,^1021−1023^ vertical cavity surface emitting lasers (VCSELs),^819,1014,1024,1025^ and even high-resolution large-area laser arrays with multicolor outputs.^696,1026^

In this section, we will discuss the optical gain in perovskite NCs including the SE under one- and multi-photon pumping as well as the optical gain mechanism. After that, the recent progress in laser devices developed from perovskite NCs will be presented. Finally, we will discuss the current challenges and perspectives of developing lasers based on perovskite NCs. We believe that perovskite NC-based lasers will become an important complement to epitaxial semiconductor lasers in the near future.

#### Optical Gain in MHP NCs

**Figure 120:**
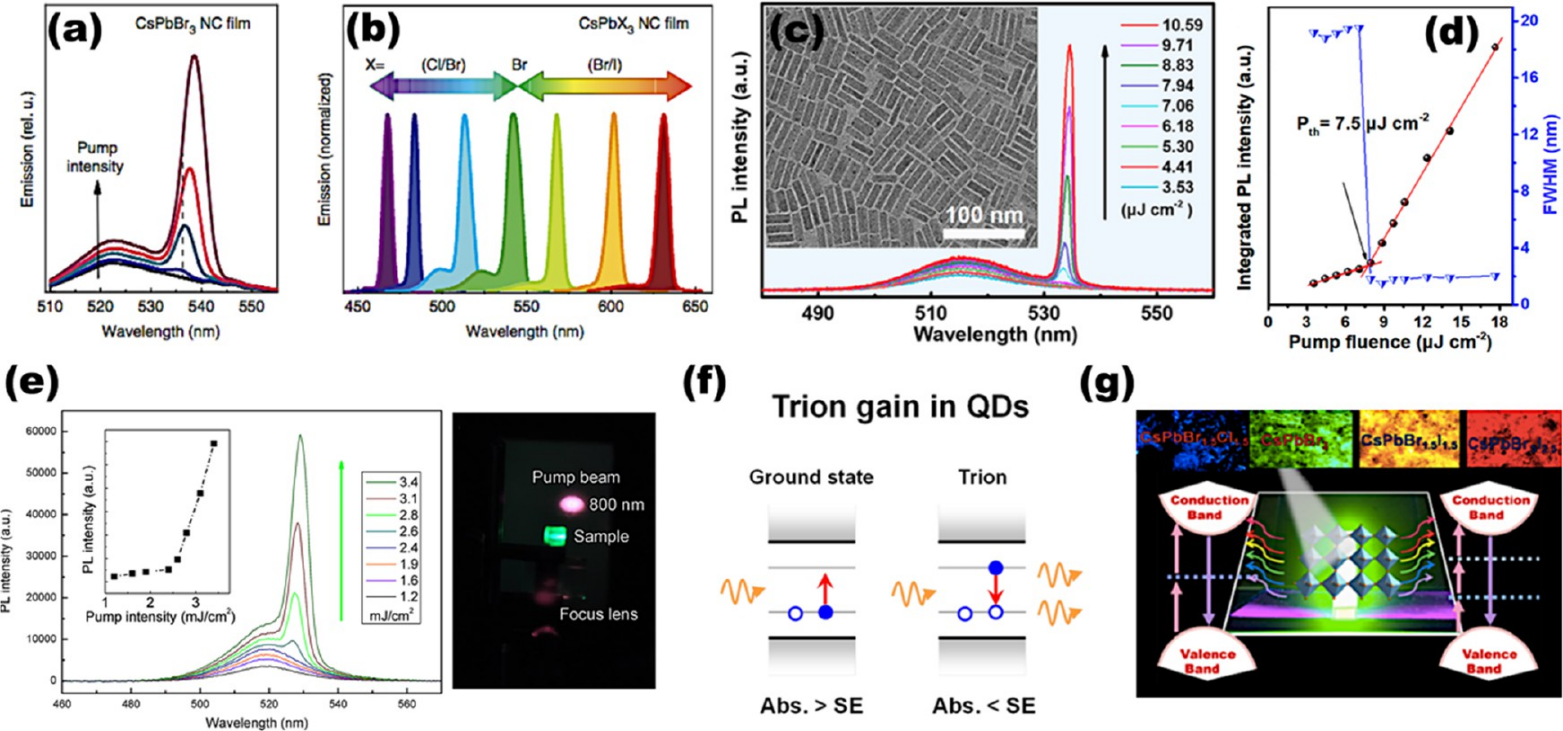
(a) Pump intensity dependence of the emission in a CsPbBr_3_ NC film (pumping intensity range was 3–25 mJ cm^–2^). (b) Spectral emission tunability of ASE *via* modulating constituents in a CsPbBr_3_ NC film. Reprinted with permission under a Creative Commons CC BY license from ref (1010). Copyright 2015 The Authors. (c) Pump-fluence-dependent emission of CsPbBr_3_ perovskite nanorods with uniform surface. Inset: Typical TEM images of low-defect CsPbBr_3_ nanorods. (d) Integrated PL intensity and line widths of CsPbBr_3_ nanorods as a function of pump fluences. Panels c and d are reprinted from ref (1027). Copyright 2019 American Chemical Society. (e) Two-photon-pumped PL spectra from CsPbBr_3_ nanocrystals at varied pump intensities. Right inset: Photograph of the stripe pumping configuration adopted to pump the CsPbBr_3_ NCs with an 800 nm laser beam with the pulse width of 100 fs and repetition rate of 1000 Hz. Reproduced from ref (1011). Copyright 2015 American Chemical Society. (f) Mechanism for trion gain in singly charged NCs with doubly degenerate band-edge states. Reproduced from ref (1028). Copyright 2018 American Chemical Society. (g) Two-photon fluorescent microscope images of different CsPbX_3_ Pe-NCs as well as the simple illustration of two- and three-photon-excited PL. Reproduced from ref (1029). Copyright 2019 American Chemical Society.

In 2014, SE was initially demonstrated in solution-processed CH_3_NH_3_PbX_3_ (X = Cl, Br, and I) perovskite thin films, indicating that the halide perovskites are not only excellent photovoltaic materials but also promising gain media for lasing.^53,176,1030,1031^ Soon after, the notable optical gain properties of IHPNs were reported by Wang *et al*. and Yakunin *et al*. nearly simultaneously in 2015.^28,1010^ Both groups demonstrated robust SE under either femtosecond or nanosecond pulsed excitation from the close-packed thin films of CsPbX_3_ IHPNs, where the thresholds were found to be much lower than those of the traditional CdSe-based NCs. The low SE threshold can be attributed to the large absorption cross section and the moderate nonradiative recombination loss (*e.g*., low rate of carrier trapping and relatively slow Auger recombination rate).^28,1014^ Leveraging on the variable stripe length technique, the modal gain in CsPbBr_3_ NCs was determined to be as high as ∼450 cm^–1^. Moreover, the SE spectrum can be easily tuned from blue, green to red region by adjusting the composition and size of IHPNs. (Figure 120a,b).^28,1010^ Later, using the intermediate monomer reservoir synthetic strategy, Wang *et al*.^1027^ fabricated rod-shaped IHPNs. Thanks to surface ligand passivation, the perovskite nanorods showed a high PLQY of up to 90% and enhanced stability in aqueous environments and at high temperature, exhibiting an extremely high gain of 980 cm^–1^ and a low SE threshold of 7.5 μJ cm^–2^ under nanosecond laser pumping, as shown in Figure 120c.^1027,1032^ In addition to the close-packed films of IHPNs, SE from the liquid solution of CsPbBr_3_ NCs has also been reported recently. The SE threshold was estimated to be 105 μJ cm^–2^, and photostability tests exhibited steady SE intensities for more than 3 h under the pump of a constant femtosecond pulsed laser beam (>10^7^ shots).^1033^ The superior gain properties of these IHPNs hold great potential for developing different classes of miniaturized laser devices.

Regarding the gain mechanism in IHPNs, Wang *et al.* performed comprehensive steady-state and time-resolved PL measurements and revealed that the optical gain might arise from the radiative recombination of biexcitons.^28^ Lately, through two-dimensional electronic spectroscopy, Zhao *et al*.^1034^ reported that the SE threshold in CsPbBr_3_ NCs is largely determined by the competition between SE from biexcitons and excited-state absorption from single exciton to biexciton states. In other words, the optical gain in CsPbBr_3_ NCs was confirmed to originate from biexcitons. The lower photon energy from biexciton recombination than single exciton transition as well as the relatively larger biexciton binding energy from NCs makes IHPNs attractive candidates as optical gain media because the red-shifted SE peak could effectively reduce the reabsorption loss in an inhomogeneous NC ensemble. In addition, trion-based optical gain in colloidal CsPbBr_3_ NCs was proposed by Wang *et al*. in 2018.^1028^ Through surface treatment with excess PbBr_2_, the trion lifetime of the CsPbBr_3_ NCs film was prolonged. At the same time, an ultralow SE threshold of 1.2 μJ cm^–2^ (the average number of excitons per nanocrystal ⟨*N*⟩ = 0.62, which is close to the theoretical threshold value of ⟨*N*⟩_th_ = 0.58 for trion-based gain) was observed, indicating the participation of trions in the optical gain. The schematic illustration of trion gain in NCs is shown in Figure 120f. Furthermore, single exciton recombination induced SE with threshold of 8–12 μJ cm^–2^ in CsPbX_3_ (X = Br, I) NCs has also been reported.^1035^ The single-exciton gain mechanism leads to low optical losses, since the nonradiative exciton–exciton annihilation (Auger recombination) can be efficiently prevented, but the reabsorption loss may be an issue. Until now, the gain mechanism in Pe-NCs remains an open question, and more comprehensive spectroscopic studies correlated with theoretical calculations are required to reach a consensus. Nevertheless, the mechanisms of stimulated emission and lasing depend on the electronic structure of the particular material because different optical processes may compete with each other. There is no universal description of the mechanism for an inhomogeneous NC ensemble.

SE induced by two-photon and even high-order multiphoton absorption in perovskite NCs has also been extensively demonstrated in recent years, which highlights the potential of these materials for nonlinear photonics and devices.^826,1011,1029,1036−1039^ Multiphoton absorption is an important branch of nonlinear optics, which features long excitation wavelengths and nonlinear excitation intensity dependent fluorescence. Hence, it brings about the advantages of deeper penetration depth, higher damage threshold, higher image contrast and fewer scattering effects.^1039^ Wang *et al*. found that the CsPbBr_3_ NCs exhibit strong nonlinear absorption and derived a two-photon absorption (2PA) cross section (σ_2_) as high as ∼1.2 × 10^5^ GM (1 GM = 10^–50^ cm^4^ s) at 800 nm for 9-nm-sized CsPbBr_3_ NCs.^1011^ It is worth mentioning that the 2PA cross section of various dye molecules is in the range of 10–10^3^ GM.^1040^ Furthermore, it was demonstrated that these close-packed thin films of CsPbBr_3_ NCs possessed low threshold of frequency-upconverted SE pumped by simultaneous two-photon absorption (800 nm, threshold ∼2.5 mJ cm^–2^) or three-photon absorption (3PA) (1200 nm, threshold ∼5.2 mJ cm^–2^) (Figure 120e), and the photostability of SE under two-photon pumping was practically favorable. Moreover, ⟨*N*⟩ can be calculated from the equation ⟨*N*⟩ = *f*^2^σ_2_/τ,^1011^ where *f* is the pump fluence (photons cm^–2^) and τ is the pulse line width; the ⟨*N*⟩ at SE threshold is estimated to be ∼1.2, which indicates that the SE in perovskite NCs arises from biexciton recombination. Soon after, a two-photon-pumped laser with high stability based on CsPbBr_3_ NCs was demonstrated in the work by Xu *et al*.^1020^Figure 120g displays two-photon fluorescent images of CsPbX_3_ perovskite NCs with different halide stoichiometries under 800 and 1064 nm excitation. It is noted that the progress in nonlinear optically pumped SE and lasing from perovskite NCs may offer additional possibilities in the development of next-generation multiphoton imaging techniques.^7,1011,1041^

**Figure 121:**
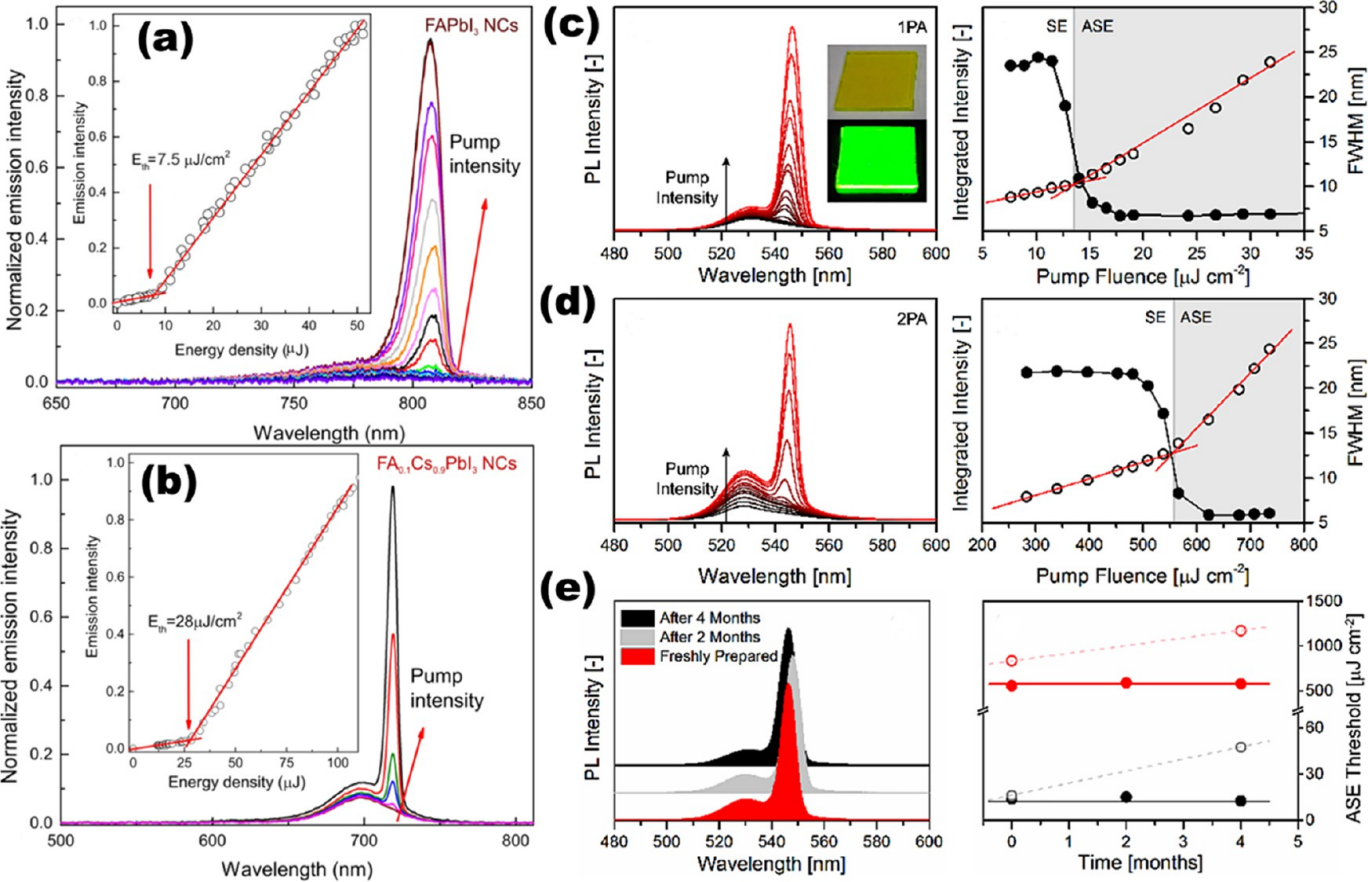
Emission spectra of (a) FAPbI_3_ and (b) FA_0.1_Cs_0.9_PbI_3_ NCs films pumped by a pulsed laser with duration of 100 fs, indicating the SE behavior with ultralow thresholds. The insets show the integrated PL intensity as a function of energy density. Reproduced from ref (69). Copyright 2017 American Chemical Society. Excited steady-state PL emission spectra of BnOH-modified MAPbBr_3_ NCs under (c) one- and (d) two-photon absorption. (e) Consistency of steady-state PL and stimulated emission peak position and the corresponding ASE threshold of different month(s) old BnOH-modified MAPbBr_3_ NC samples stored under ambient conditions. Reproduced from ref (1042). Copyright 2017 American Chemical Society.

Apart from the IHPNs, the OIHP NCs also exhibit SE with fairly low thresholds and the photostability was improved by surface ligand engineering and chemical treatment. In 2017, Protesescu *et al.* synthesized monodisperse, nearly cubic FAPbI_3_ and FA_0.1_Cs_0.9_PbI_3_ with average sizes of 10–15 nm, which extends the emission spectra into the NIR range (*e.g.*, 780 nm for FAPbI_3_ NCs).^69^ The SE threshold of 7.5 μJ cm^–2^ of FAPbI_3_ was among the lowest values of the red to near-IR emitting perovskites (5–10 μJ cm^–2^).^17,525,1010,1043^Figure 121a,b separately shows the emission spectra of FAPbI_3_ and FA_0.1_Cs_0.9_PbI_3_ NCs films pumped by a pulsed laser with pulse width of 100 fs, indicating ultralow threshold SE.^69,1044^ The integrated PL emission intensity as a function of pump fluence is plotted in Figure 121a,b, separately. It is highlighted that surface engineering can serve as an effective strategy to improve the stability where the active media are made of organic–inorganic hybrid components.^1037,1044^ The robust FAPbI_3_ NCs exhibiting low-threshold SE manifest improved ambient thermodynamic and chemical stability over pristine CsPbI_3_ analogues,^1045,1046^ making them suitable for light-emitting applications, including lasers in the red spectral region. Also, it was demonstrated that FAPbBr_3_ NCs show low SE threshold and temperature insensitive SE under both one- and two-photon pumping, benefiting from large two-photon absorption coefficient (0.76 cm GW^–1^) and high optical net gain (480 cm^–1^).^1047^ The WGM lasing from these FAPbBr_3_ NCs under two-photon excitation was achieved by inserting FAPbBr_3_ NCs into a microresonator.^1047^ In 2017, Veldhuis *et al*.^1042^ reported the high-yield synthesis of luminescent MAPbBr_3_ NCs through direct precipitation of the chemical precursors in a benzyl alcohol (BnOH)–toluene phase, where BnOH can steer the passivating ligands and maintain the ligand binding motifs on the NCs surface, resulting in improved structural stability and optoelectronic properties. They revealed ultralow SE thresholds (13.9 ± 1.3 μJ cm^–2^ under one-photon (400 nm) absorption, Figure 121c; 569.7 ± 6 μJ cm^–2^ at two-photon (800 nm) absorption, Figure 121d), high stability under ambient storage and measurement conditions (Figure 121e), as well as outstanding optical modal gain coefficient (520 cm^–1^) through the detailed ultrafast spectroscopic studies.

#### Laser Devices Developed From MHPs

A suitable feedback mechanism combined with a gain material is the key to realize a laser device, in which the light can be amplified in certain resonating frequencies.^1036,1037^ In this regard, a variety of high-quality optical resonators were employed aiming at realizing desirable coherent light output based on perovskite NCs.

**Figure 122:**
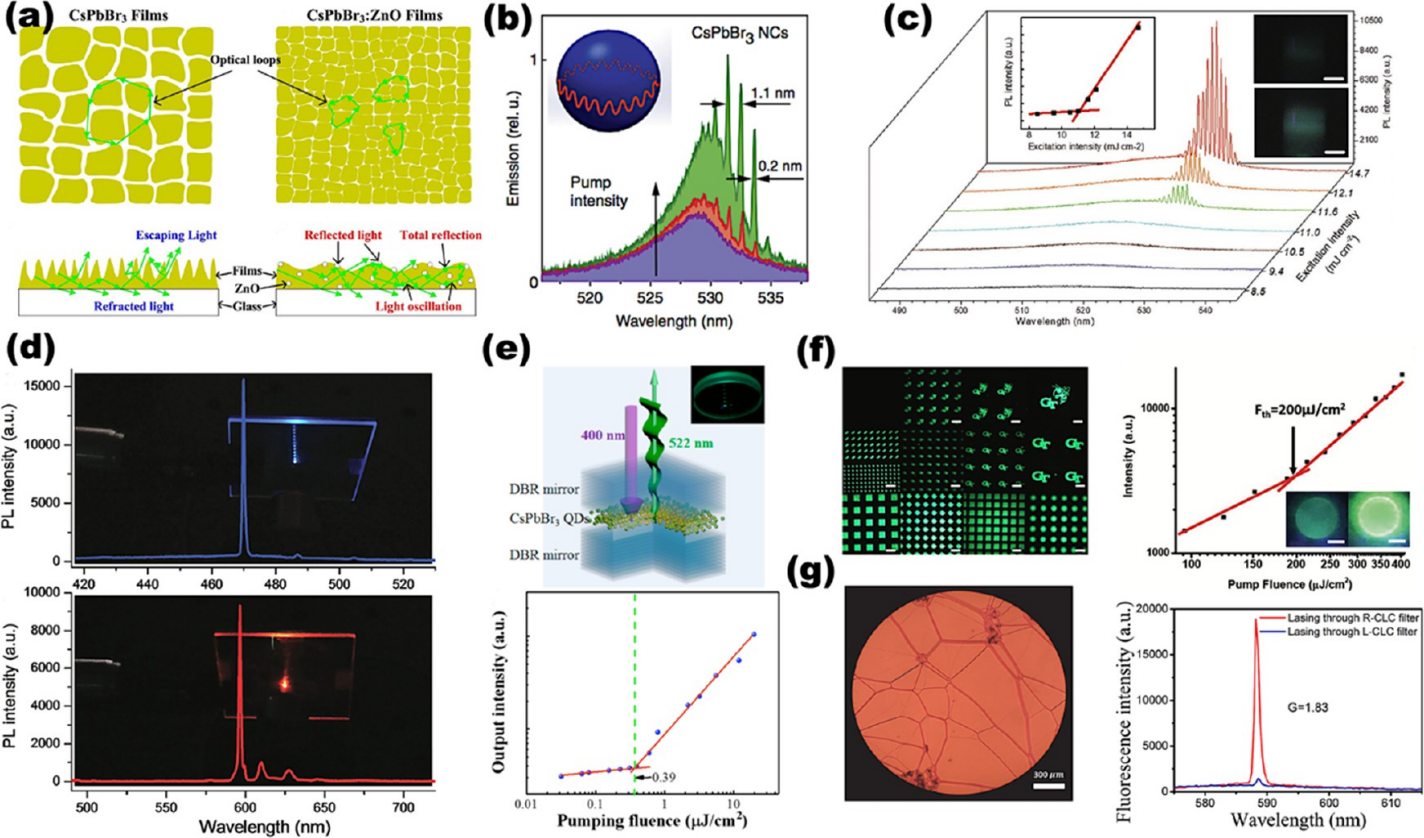
Different laser devices based on perovskite NCs. (a) Shortened optical loops and increased light oscillation in the perovskite CsPbBr_3_:ZnO films for random lasing. Reproduced with permission from ref (1049). 2017 Elsevier Ltd. (b) WGM lasing in a microsphere resonator of 15 mm in diameter, covered by a film of CsPbBr_3_ nanocrystals. Reprinted with permission under a Creative Commons CC BY license from ref (1010). Copyright 2015 The Authors. (c) WGM lasing from CsPbBr_3_ nanocrystals infiltrated into a capillary tube with inner diameter of ≈50 μm. Reproduced with permission from ref (28). Copyright 2015 John Wiley & Sons, Inc. (d) Blue and red lasing spectra of VCSELs from CsPb(Br/Cl)_3_ and CsPb(I/Br)_3_ IHPNs under pump intensity of 38.2 and 30.5 μJ cm^–2^, respectively. Reproduced with permission from ref (1014). Copyright 2017 John Wiley & Sons, Inc. (e) Schematic of CsPbBr_3_ NC-based VCSELs setup with ultralow lasing threshold (0.39 μJ cm^–2^). Reproduced from ref (1051). Copyright 2017 American Chemical Society. (f) Different arrays of CsPbBr_3_ nanocrystals patterns and lasing in arrays of microdisk lasers. Reproduced with permission from ref (696). Copyright 2018 John Wiley & Sons, Inc. (g) Single mode lasing action in CsSnI_3_-doped with CLC cavities. Reproduced from ref (582). Copyright 2018 American Chemical Society.

Random lasers are the simplest laser configuration where the optical feedback is offered by the constructive interference of the scattered light in a disordered system.^1036,1048^ In 2017, random lasing was demonstrated in the perovskite CsPbBr_3_:ZnO films. The ZnO nanoparticles were found to be able to improve the lasing performance thanks to the shortened optical loops and increased light oscillation as shown in Figure 122a. In this way, the SE thresholds of CsPbBr_3_:ZnO films were significantly reduced under both 1PA and 2PA.^1049^ Leveraging on the similar strategy, ultralow threshold random lasing was achieved by depositing MAPbBr_3_ NCs on a heterostructure of 3D graphene-sheathed SiC nanowalls.^1050^ Strong scattering of emitted photons by leachy vertical graphene networks provide the effective optical feedback to achieve random lasing. Moreover, the lasing threshold can be further lowered by the combined effect of the improved scattering cross section and plasmonic field enhancement of extra Ag/SiO_2_ particles.

Silica microspheres can naturally serve as WGM cavities. Yakunin *et al.*(1010) coated the IHPNs onto silica spheres to construct a WGM microlaser (Figure 122b, inset), in which the light propagation was total internally reflected around the circular cavity edges.^1010,1036^ WGM lasing could also be developed by infiltrating the gain media into a capillary tube.^1052,1053^ In a similar way, Wang *et al*. coated a thin film of CsPbBr_3_ NCs onto the inner wall of a capillary tube to realize high-performance WGM lasers with a quality factor (Q-factor) of ∼2000 (Figure 122c).^28^ The occurrence of evenly spaced spikes and super-linear increase of the integrated PL intensity *versus* pump fluence (inset in Figure 122c, left) confirmed the development of lasing action, and the longitudinal optical modes could be well-assigned according to the WGM model.^1053−1055^ Recently, by embedding a CsPbBr_3_–SiO_2_ spheres into a microtubule, the frequency up-converted WGM lasing over 140 min with a low lasing threshold of 430 μJ cm^–2^ has been successfully achieved under two-photon excitation. Combining the effects of natural microring resonator of SiO_2_ and high gain of CsPbBr_3_ NCs provides a promising strategy to realize frequency up-converted lasing devices with low threshold.^1056^

A DFB laser is made of a grating structure in which the active region contains a periodically varied refractive index distribution. The grating provides optical feedback for a wavelength satisfying the Bragg condition.^1037^ In 2016, DFB lasers based on MAPbI_3_ perovskites with threshold at optical pump intensities of 5 kW cm^–2^ for durations up to ∼25 ns at repetition rates exceeding 2 MHz were reported. It highlighted that using the short pulse drive would be an effective strategy to reduce the threshold in a perovskite NC-based laser diode.^1021^ After that, highly green luminescent MAPbBr_3_ perovskite film composed of large NCs were used to produce stable DFB lasers at 550 nm with a low threshold of 6 μJ cm^–2^. These DFB lasers were able to support multiple polarizations and could be switched between transverse magnetic and transverse electric mode operation through tuning of the distributed feedback grating period.^1022^

Additionally, VCSELs, basically constructed by inserting an active layer into two parallel reflecting mirrors, have been demonstrated based on perovskite NCs. In 2017, Wang *et al*.^1014^ sandwiched the CsPbX_3_ NCs between two distributed Bragg mirrors (DBRs) to achieve high-performance VCSELs. These lasers showed low threshold (9 μJ cm^–2^), directional output (beam divergence of ∼3.6°), and favorable stability. Blue-emitting CsPb(Br/Cl)_3_ IHPNs and red-emitting CsPb(I/Br)_3_ IHPNs were similarly inserted into the DBR resonators to obtain the VCSELs across the full visible spectral range (Figure 122d). In the same year, VCSELs based on CsPbBr_3_ NCs with ultralow lasing threshold (0.39 μJ cm^–2^, Figure 122e) was also reported. The schematic of the CsPbBr_3_ NCs based VCSELs is shown in Figure 122e. These VCSELs exhibited stable device operation over 5 h or 1.8 × 10^7^ optical excitation pulses at ambient condition, demonstrating the potential in practical coherent light-emitting applications.^1051^

Moreover, duplicatable and scalable microlaser arrays have been realized from CsPbX_3_ NCs relying on an orthogonal lithography approach, which is promising for integrated photonic applications.^696^ For example, Lin *et al*. fabricated large-area high-resolution arrays of microdisk lasers and multicolor (binary and ternary emission) pixels (Figure 122f).^696^ The reported orthogonal lithography method preserved the high optical gain performance of CsPbBr_3_ NCs, which was the key to achieve the WGM lasing.^696,1026^

Dynamically tunable lasers have been realized by doping CsSnI_3_ NCs into cholesteric liquid crystal (CLC) reflectors. (Figure 122g).^582^ A similar approach was employed by Stranks *et al.*(1057) to obtain robust lasing under nanosecond pumping at 532 nm (a minimum threshold of 7.6 μJ cm^–2^). A thin CLC film (∼7 μm) coupled with a metal back-reflector was adopted to construct the cavity. The use of flexible CLC reflectors provides a pivotal step toward “mirror-less” single-mode lasers on flexible substrates, which could be exploited in applications such as flexible displays and military identification.

#### MHP Nanowire Lasers

**Figure 123:**
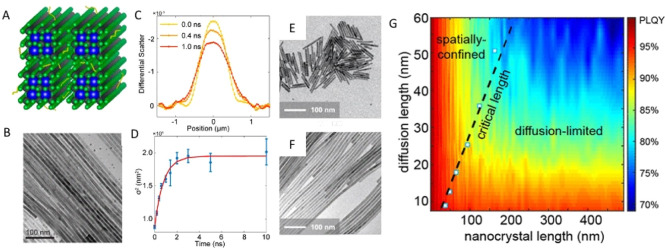
(A) Schematic diagram and (B) SEM image of perovskite nanowire bundles. (C,D) Diffusion profile of energy along the longitudinal axis of the nanowires. Reproduced from ref (1058). Copyright 2020 American Chemical Society. (E,F) TEM images of CsPbBr_3_ nanowires with different aspect ratios. (G) 2D counter plot as an illustration of the optical scaling law of PLQY as a function of NC length in both the spatially confined regime and the diffusion-limited regime Reproduced from ref (239). Copyright 2020 American Chemical Society.

Despite the fact that the carrier dynamics in perovskite NCs has been extensively studied in 0D quantum dots systems, studies on the 1D geometry of perovskite NWs also demonstrate an important role on the modification of electronic structure, carrier trapping, and exciton decay mechanisms. Carrier diffusion in one-dimensional CsPbBr_3_ NWs with 10 nm lateral widths were directly visualized from stroboSCAT (stroboscopic interferometric scattering microscopy) measurements (Figure 123A–D).^1058^ The rapidly diffusing excitons encounter less trap densities along the NWs. The qualitative study using ultrafast transient microscopy showed the anisotropy splitting of the band-edge exciton in NWs due to dielectric confinement in one dimension.^1058^

To demonstrate the charge carrier behaviors in a more controllable system, high-quality, single-crystalline 1D CsPbX_3_ NWs with aspect ratios varying from 1 to 1000 were used to construct a model platform to investigate the optical scaling laws of NCs (Figure 123E–G).^239^ NW surface ligands with tunable Lewis acidity offer control over the nonradiative rate of the perovskite NWs. Steady-state PLQY and time-resolved PL lifetime measurements have yielded valuable information on the impact of NWs aspect ratio on excitonic dynamics within the wire. The scaling laws derived from this model system are not only a phenomenological observation but unraveled the carrier dynamics of these microscopic systems in a quantitative and interpretable manner. Monte Carlo simulations with an exciton-diffusion–defect-encounter random walk model extracted an exciton diffusivity of 0.4 cm^2^/s, and together with the scaling behaviors, revealed materials dimensionality as a hidden constraint on the carrier recombination kinetics. In addition, Janker *et al.* employed the spatiotemporal dynamics of electrons and holes in aligned CsPbI_3_ NW bundles using acousto-optoelectric spectroscopy.^1059^

**Figure 124:**
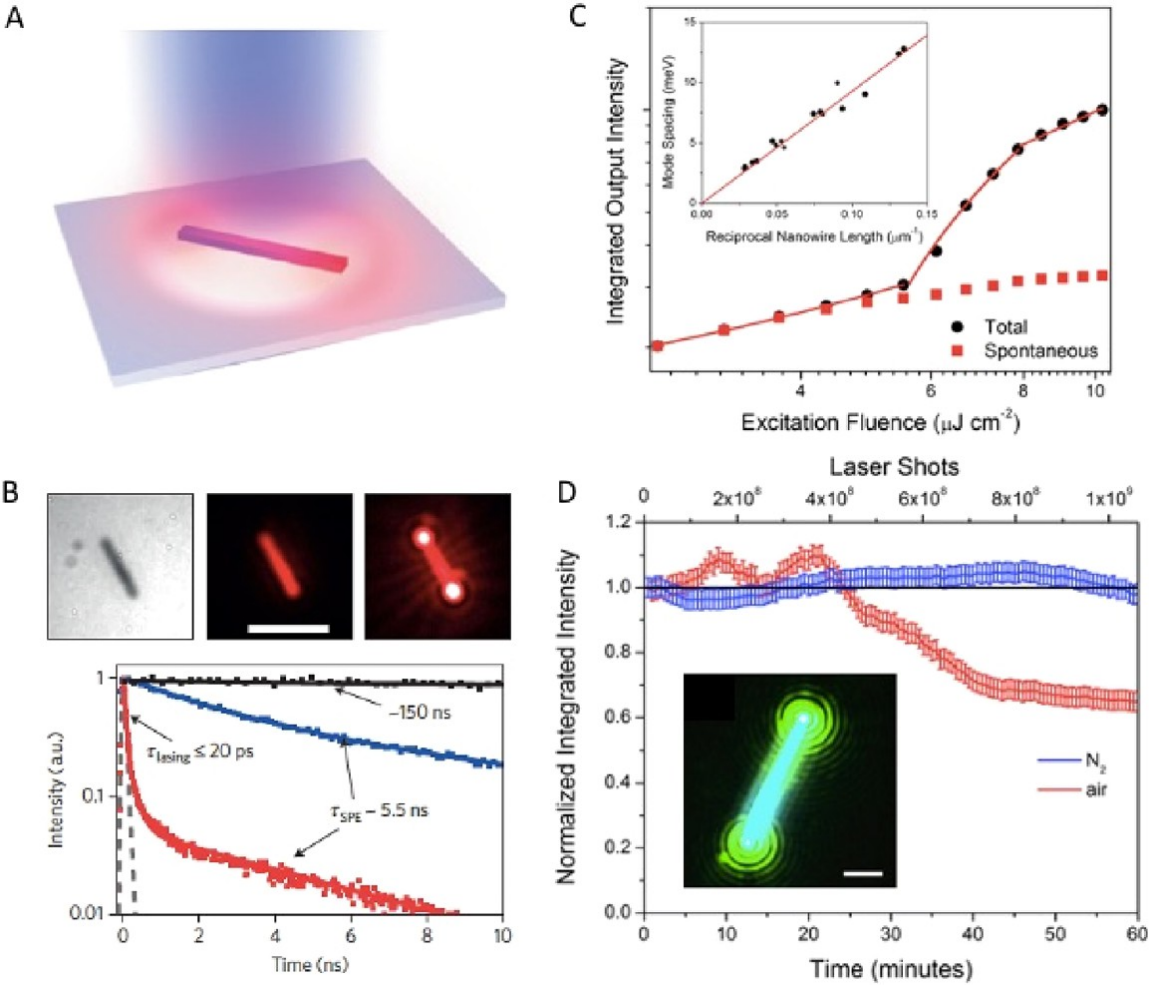
(A) Schematic diagram of perovskite nanowire pumped by a laser. (B) Optical images of a single nanowire with and without laser excitation and corresponding transient PL decay kinetics at certain excitation intensity. Reproduced with permission from ref (34). Copyright 2015 Nature Publishing Group. (C) Integrated output intensity from CsPbBr_3_ nanowire as a function of increasing excitation fluence. (D) Stability test of lasing from CsPbBr_3_ nanowire in both air and N_2_ environment. Reproduced with permission under a Creative Commons CC BY license from ref (244). Copyright 2016 National Academy of Sciences of the United States of America.

The carrier dynamics studies of perovskite NWs mentioned above paved the way for the rationally designed NW laser systems. NW lasers are ideal candidates for miniaturized light sources, providing both the optical gain medium and the resonant laser cavity that can potentially allow their facile integration into circuits. The perovskite NWs that were synthesized from colloidal methods are too thin to effectively support the photonic lasing modes. A low-temperature, solution-phase growth of single-crystalline CsPbX_3_ NWs with a few hundred nm in width and micron length scale led to the Fabry-Pérot mode lasing behavior with a low lasing threshold, high maximum quality factor, and the wavelength tunability from blue to near-IR regions of the visible spectrum (Figure 124).^34,242,244,1060,1061^ The confined exciton–polaritons in perovskite NWs and the composition-dependent Rabi splitting has been studied using high-quality in-plane aligned CsPbX_3_ (X = Cl, Br, I) NWs that were grown on the M-plane sapphire substrates.^1062^ The corresponding energy–wavevector dispersion relation of the lasing mode well agreed with the exciton–polariton model, and the Rabi splitting was extracted as ∼210 ± 13, 146 ± 9, and 103 ± 5 meV in CsPbCl_3_, CsPbBr_3_, and CsPbI_3_ NWs. Moreover, the lasing from CsPbBr_3_ NWs has been maintained for over 1 h of constant pulsed excitation in both nitrogen and ambient atmospheres (Figure 124D).^244^ This represents significant stability of inorganic perovskite NWs and demonstrates the viability of the robust, all inorganic compositions for photonic integrated circuits that require highly stable miniaturized light sources. In addition to the inorganic perovskite NWs for lasing, organic–inorganic hybrid CH_3_NH_3_PbX_3_ NWs have been grown from vapor-phase synthesis and equally show excellent optical properties with adequate gain and efficient optical feedback.^1063^ The surface plasmon effect in CH_3_NH_3_PbBr_3_/SiO_2_/Ag cavity can further enhance the strong exciton-photon interactions in perovskite NWs.^1064^ The exciton-photon coupling strength can be enhanced by ∼35%, and this is attributed to the localized excitation field redistribution from surface plasmon effect.

The origin of the lasing in halide perovskite NWs is still a controversial topic. In addition to the lasing mechanism involving exciton–polaritons as mentioned above, Schlaus, *et al.*, have proposed that under the pulsed excitation density, the excitation power would exceed the exciton Mott density, and as a result, lasing in CsPbBr_3_ NWs was originated from the stimulated emission of a nondegenerate electron–hole plasma rather than exciton–polaritons.^1065^ The changes in laser gain profile and refractive index that lead to the lasing mode distribution of NWs strongly depend on excitation density and pulse duration time. In particular, the high intrinsic PL quantum efficiency is crucial for advancing their application as light-emitting sources. It has been demonstrated that the quantum efficiency of single-crystalline CsPbBr_3_ NWs can be improved by 3 orders of magnitude upon exposure to oxygen molecules.^249^ Oxygen can passivate the perovskite surface defects originated from lead-rich surface, therefore it greatly reduces the nonradiative recombination rate.

#### Summary and Future Outlook for MHP Lasers

Perovskite NCs, including organic–inorganic hybrid and all-inorganic perovskite NCs, are emerging as a contemporary class of cost-effective and wavelength-tunable lasing materials. Although tremendous progress has been made in developing solution-processed lasers from perovskite NCs, especially in terms of understanding the fundamental physics and improving the device performance, there remain challenges with regard to developing practical and commercially available lasers utilizing the perovskite NCs. Firstly, these perovskite NCs are severely affected by chemical and environmental factors (*e.g*., oxygen, moisture, heat, and continuous light illumination) instabilities.^462,1066,1067^ Li *et al.* developed an amination-mediated nucleation strategy and demonstrated significantly improved SE stability of perovskite NCs.^1068^ In another case, Yuan *et al.* fabricated CsPbBr_3_ NCs in a glass matrix *in situ* crystallization synthesis, which not only protected the NCs from the ambient conditions, but also hindered their aggregation.^464^ In 2017, Wang *et al.* demonstrated the insertion of CsPbBr_3_ NCs into a wider-band-gap Cs_4_PbBr_6_ matrix through a low-temperature solution-phase synthesis method. It was found that the thermal stability of IHPNs is enhanced, and robust high-temperature perovskite lasers could be realized.^364^ However, most of the strategies are only applicable for the pure, green emitting CsPbBr_3_ NCs, while the stability of blue-emitting and red-emitting perovskite NCs is lagging behind. Secondly, most of the progress made on perovskite NCs lasers^168,1069−1071^ has focused on lead-containing compounds, which are toxic and their use may be restricted in the future. As a result, studying nontoxic NCs and developing heavy metal-free perovskite NCs for laser media will probably be an irreversible trend.^1072^ For example, air-stable lead-free double perovskites NCs with chemical formula A_2_MM′X_6_, where A is a monovalent cation (Cs^+^, CH_3_NH_3_^+^, etc.), M is also a monovalent cation (Ag^+^, Na^+^, etc.), M′ is a trivalent cation (In^3+^, Bi^3+^, Sb^3+^, etc.), and X is the halogen anion (Cl^−^, Br^−^, I^−^) have been recently synthesized (see dedicated sections in this review on NANOCRYSTALS OF LEAD-FREE PEROVSKITE-INSPIRED MATERIALS), and they could be promising for lead-free perovskite lasers in the near future.^571^ Thirdly, to date, only optically pumped lasing has been demonstrated in perovskite NCs, whereas electrical pumping is more practically desired.^1071,1072^ Despite significant progress in optically pumped lasers and electrically driven light-emitting diodes has been demonstrated, there is still a long way to go before realizing electrically-pumped perovskite NC lasers. In particular, the following issues have to be addressed to achieve lasing in NCs by electrical pumping. First, the Auger recombination generally limits the electrically driven lasing in perovskite NCs because the carriers are injected into perovskite NCs one-by-one.^1073^ Thus, this nonradiative Auger recombination has to be suppressed by electron–hole wave function engineering and other additional strategies. Second, the organic ligands used for the passivation of perovskite NC surfaces generally exhibit poor electrical conductivity, hampering the carrier injection and transport.^1010,1070^ Therefore, it is imperative to find ways to achieve efficient injection of charge carriers into the perovskite NC layer. The methods include the modification of the NC surface and the reduction of the thickness of the emitting layer.

### Light-Emitting Devices

Light-emitting diodes based on lead-halide perovskites emerged more than a decade ago. However, there was no electroluminescence reported at that time because of the weak light emission from LHPs.^1074−1076^ However, in the past few years, there have been significant developments and LHPs have returned to the spotlight, not only as highly efficient photon absorbers in solar cells, but also as efficient photon emitters in LEDs.^8,31,1077^ Interestingly, the external quantum efficiencies of LHP-LEDs reached the same level as organic LEDs and colloidal cadmium selenide QD LEDs of over 20% in just 5 years.^404,1078,1079^ Generally, the whole LHP-LED with a total thickness of hundreds of nanometers is deposited onto a transparent substrate coated with an indium tin oxide electrode, and functional films are also required for facilitating charge carrier injection into the LHP layer from external electrodes.^31,404,1078,1079^ Because the LHP emitter and other functional layers are deposited by solution processing, the device structures of most LHP-LEDs are simple.^31,404,1078,1079^ By changing the halide anion from chloride to iodide, the emission wavelength of LHPs can be tuned across the whole visible range (refer to Composition Control by Ion Exchange and Suppression of Exchange section).^14,53,80,1080,1081^ Moreover, nanostructured emitters are effective for confining charge carriers in the LHP layer and achieve highly efficient radiative recombination. These nanostructured emitters include 3D NCs, quasi-2D nanoplatelets, and multilayer quantum wells.^22,216,385,901,1062,1078,1082^ Apart from high EQEs, LHP-LEDs achieve narrow emission peaks with high-color purity.^53,78,404,1083^ LHP-LEDs are therefore a natural candidate for potential applications in full-color information displays. So far, bromide- and iodide-based green and near-infrared LHP-LEDs have achieved record EQEs of over 20%. However, the development of blue LHP-LEDs lags behind.^385,404,1078,1079,1084^ The synthesis of a wide variety of LHP emitters and their deposition in films can be conducted simply and quickly, even in ambient atmosphere, which is another advantage compared to their counterparts, such as CdSe QDs.^14,52,78,1083^ As a soft semiconductor emitter, the similarity in the processing LHPs and OLEDs/QD-LEDs suggests that LHPs may be compatible with the booming OLED/QD-LED industry.^1062,1078,1079^ However, LHP emitters and the resulting LEDs are still limited by the toxicity of the lead ions and rapid degradation under operation condition, and efforts to develop lead-free alternatives are discussed in the earlier section on NANOCRYSTALS OF LEAD-FREE PEROVSKITE-INSPIRED MATERIALS.^112,469,885,1080,499,1085−1088^ Details of the fundamental properties of LHPs (*e.g.*, band gap tunability, defect tolerance and carrier dynamics) are covered in previous sections, particularly the section on OPTICAL PROPERTIES.

#### Classification of Perovskite Light Emitters

**Figure 125:**
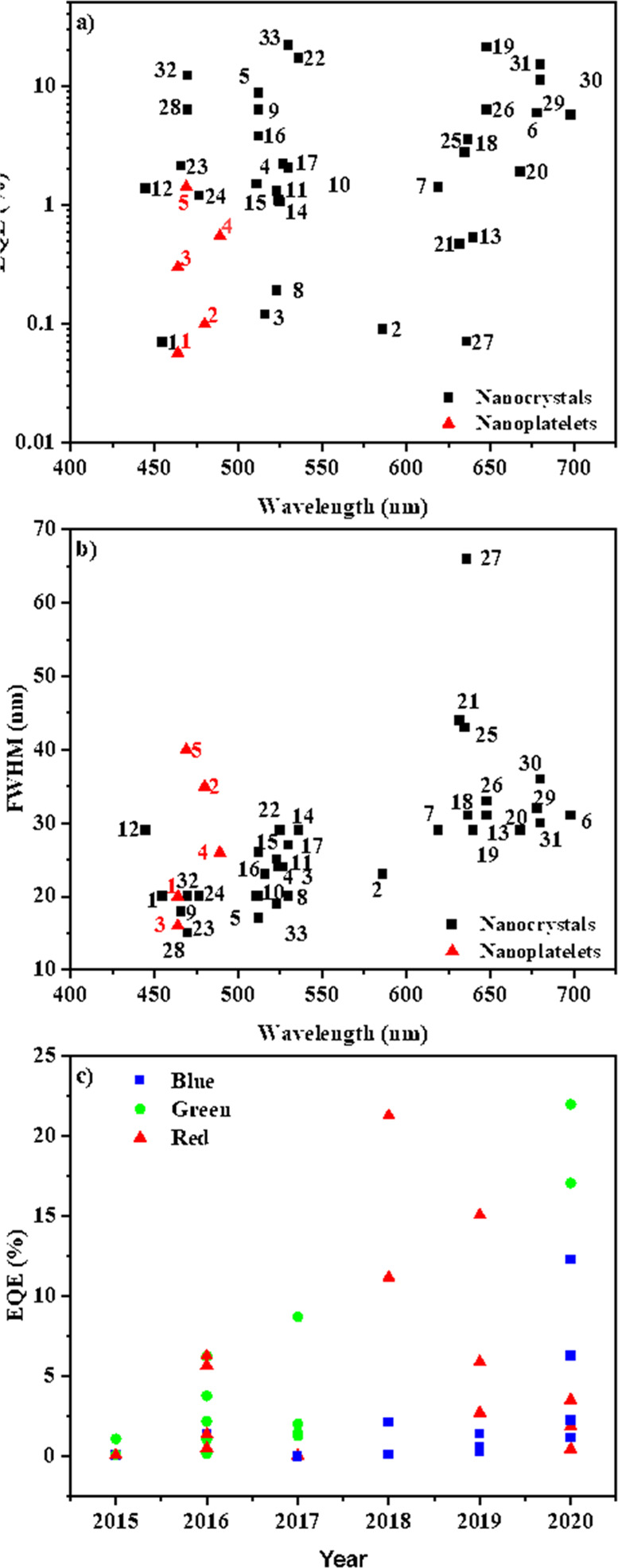
(a) EQE and (b) PL fwhm *vs* emission wavelength for different emitters and structures. The number labeled in the figure matches the label number in Table 3, and the corresponding references are also shown in Table 3. (c) Development of red, green, and blue PeLEDs over time.

Although the initial perovskite LEDs to operate at room temperature used bulk 3D perovskite thin films, the EQEs reached up to only ∼1%.^31^ An important challenge was the low exciton binding energy of a few meV in the films,^894^ necessitating the spatial confinement of charges to increase the fraction of injected carriers that radiatively recombine.^1089^ While this was initially achieved by creating a quantum well (by sandwiching the emitter between two injectors that each block one of the carriers),^31^ a more effective strategy was to create a multi-quantum-well structure through thin films comprising mixtures of perovskites with different dimensionality (3D, 2D, and quasi-2D).^385^ This has led to near-infrared perovskite LEDs with >20% EQE.^1079^ However, controlling the phase-purity and distribution of phases in these multidimensional perovskite thin films remains challenging.^1089^ An important alternative to thin films for improving the spatial confinement of charge carriers is through nanostructured perovskites. These include colloidal nanocubes, nanoplatelets, NCs embedded in 3D perovskite matrices and perovskite–polymer composites (Figure 125). Nanostructured perovskites have the advantages of higher exciton binding energy, band gap tunability, and the ability to passivate the surfaces to achieve high PLQYs near-unity.^52^ Details of the synthesis and optical properties of nanocubes and NPls are given above, while 0D nonperovskites and NCs embedded in these nonperovskites are discussed in other sections of this review. The discussion below focuses on the application of these materials in LEDs.

**Table 3:** External Quantum Efficiency and Full Width at Half-Maximum of Electroluminescence of Perovskite Nanocrystal and Nanoplatelet Light-Emitting Diodes

labels in Figure 125	emitting materials	EL peak center (nm)	EQE (%)	fwhm (nm)	ref
1	CsPbBr_*x*_Cl_3–*x*_	455	0.07	20	(27)
2	CsPbBr_*x*_I_3–*x*_	586	0.09	23	(27)
3	CsPbBr_3_	516	0.12	23	(27)
4	CsPbBr_3_/CsPb_2_Br_5_	527	2.21	24	(1066)
5	CsPbBr_3_	512	8.73	17	(190)
6	CsPbI_3_	698	5.7	31	(1067)
7	CsPbBr_0.75_I_2.25_	619	1.4	29	(1067)
8	CsPbBr_3_	523	0.19	19	(1067)
9	CsPbBr_3_	512	6.27	20	(184)
10	CsPbBr_3_:Mn^2+^	511	1.49	20	(621)
11	MA_0.8_Cs_0.2_PbB_r3_	523	1.3	25	(1090)
12	MAPb(BrCl)_3_	445	1.37	29	(1091)
13	MAPbI_3_	640	0.53	29	(1091)
14	MAPbBr_3_	525	1.06	29	(1091)
15	MAPbBr_3_	524	1.1	24	(141)
16	MAPbBr_3_	512	3.8	26	(1083)
17	FAPbBr_3_	530	2.05	27	(403)
18	CsPbI_3–*x*_Br_*x*_	637	3.55	31	(1092)
19	CsPbBr_0.6_I_2.4_	648	21.3	31	(1078)
20	CsPbI_3_	668	1.9	29	(1093)
21	CsPbBr_*x*_I_3–*x*_	632	0.47	44	(1093)
22	FAPbBr_3_	536	17.1	29	(1094)
23	CsMnyPb_1–*y*_Br_*x*_I_1–*x*_	466	2.12	17.9	(1095)
24	CsPbBr_*x*_Cl_3–*x*_	477	1.19	20	(1096)
25	MAPbBr_*x*_I_3–*x*_	635	2.75	43	(1097)
26	CsPbBr_*x*_I_3–*x*_	648	6.3	33	(1098)
27	CsPbBr_*x*_I_3–*x*_	636	0.071	66	(1099)
28	CsPbBr_*x*_Cl_3–*x*_	470	6.3	15	(1100)
29	Sr-CsPbI_3_	678	5.92	32	(627)
30	Ag-CsPbI_3_	680	11.2	36	(631)
31	CsPb_0.64_Zn_0.36_I_3_	680	15.1	30	(626)
32	CsPbBr_3_	470	12.3	20	(1101)
33	CsPbBr_3_	530	22	20	(1101)
1	CsPbBr_3_ NPl	464	0.057	20	(60)
2	CsPbBr_3_ NPl	480	0.1	35	(216)
3	CsPbBr_3_ NPl	464	0.3	16	(1102)
4	CsPbBr_3_ NPl	489	0.55	26	(1102)
5	CsPbBr_3_ NPl	469	1.42	40	(1103)

##### Nanocrystal Emitters

**Figure 126:**
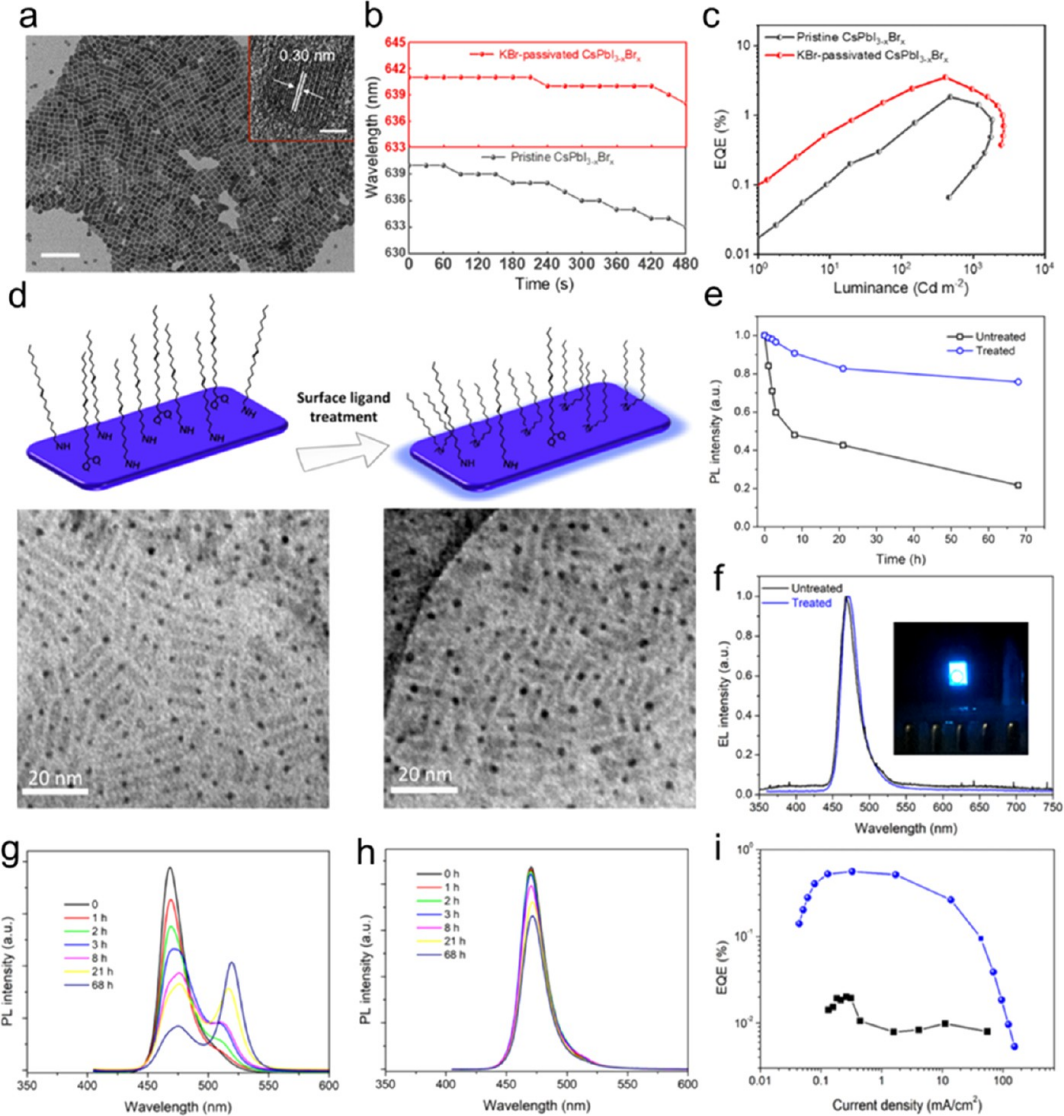
(a) TEM image of KBr-passivated CsPbI_3–*x*_Br_*x*_ NCs. The inset shows the corresponding HRTEM image. Scale bars: 100 and 5 nm. (b) PL peak position as a function of irradiation time for pristine and KBr-passivated CsPbBr_3–*x*_I_*x*_ NC films. The ensemble films were continuously excited by a laser emitting at 365 nm with a power density of 100 mW cm^–2^. (c) EQEs of LEDs based on prepared pristine and KBr-passivated CsPbI_3–*x*_Br_*x*_ NCs at different luminance. Panels a–c are reprinted from ref (1092). Copyright 2020 American Chemical Society. (d) TEM images of untreated and treated CsPbBr_3_ NPls. (e) Remnant PL intensity of treated and untreated NPls. (f) EL spectra of the untreated and treated CsPbBr_3_ NPl-based LEDs. Inset: Photograph of a working treated CsPbBr_3_ NPl-based LED at a driving voltage of 5 V. Recorded PL spectra of (g) untreated and (h) treated CsPbBr_3_ NPl/toluene solutions. (i) External quantum efficiency–current density curves of the untreated and treated CsPbBr_3_ NPl-based LEDs. Panels d–i are reprinted from ref (1103). Copyright 2019 American Chemical Society.

Efficient performance has been achieved in perovskite NC LEDs emitting across the entire visible wavelength range. The morphology of the most widely explored LHP NCs is shown in Figure 126a. Two critical strategies that have enabled this result are surface passivation and the use of dopants.

An important source of nonradiative recombination is due to uncoordinated Pb^2+^ at the surface of NCs. In red-emitting CsPbI_3_ NCs, the uncoordinated Pb^2+^ ions were passivated by introducing excess iodine to the surface. This was achieved using excess trimethylsilyl iodine as the iodine source during synthesis, which resulted in the surface I/Pb ratio reaching 4.4. Through surface passivation, the PLQY of the colloidal NCs in solution approached unity, and the device reached 1.8% EQE.^168^ Surface passivation can also be achieved post-synthesis. For example, Pan *et al.*(172) introduced 2,2′-iminodibenzoic acid to CsPbI_3_ NCs, leading to the a peak EQE increase from 2.26% to 5.02%. The improvement in performance was attributed to the bidentate ligands binding firmly to the PbI_2_-rich surface of the NCs and reducing the density of surface traps. Potassium halides have also been found to be effective surface passivation agents and were used by Yang *et al.* to passivate the surface of CsPbI_3–*x*_Br_*x*_ NCs to suppress phase separation into iodide- and bromide-rich regions, and this stabilized the PL spectra over time, as shown in Figure 126b. In doing so, they achieved electroluminescence at 637 nm wavelength, which is required for pure-red emission for displays, and increased the EQE from 1.89% (pristine NCs) to 3.55% (KBr-passivated NCs) as shown in Figure 126c.^1092^

An important challenge with CsPbI_3_ is that the cubic perovskite phase (the α-phase) is metastable at room temperature, due to the small size of the Cs^+^ cation, which leads to the Goldschmidt tolerance factor being below the range for cubic perovskites (refer to the beginning of the review).^51^ The room-temperature orthorhombic phase has a wider band gap and undesirable optoelectronic properties.^1093^ An approach to stabilize the α-phase at room temperature is to partially replace Pb^2+^ cations with smaller cations (*e.g.*, Sr^2+^, Ag^+^, and Zn^2+^), in order to increase the tolerance factor. LEDs made from these perovskites emitted at 678–690 nm, with EQEs ranging from 5.92% (Sr doping) to 15.1% (Zn doping).^626,627,631^ Another successful approach was iodide anion-exchange in CsPbBr_3_ NCs. For example, Mathews and co-workers used FAI in water as the iodide source for ion exchange. Water was used because it is not miscible with the toluene solvent for the colloidal CsPbBr_3_ quantum dots, therefore preventing ligand desorption. By tuning the concentration of FAI in the aqueous solution, either mixed Br/I or pure I-based NCs were achieved, with EL wavelengths ranging from 630 to 670 nm (pure iodide) and high PLQYs >74%. However, the EQEs only reached up to 1.9% for CsPbI_3_ NCs.^1093^ Chiba *et al.*(1078) achieved much higher EQEs, reaching 21.3%, through anion exchange using iodide-containing ligands. Starting with CsPbBr_3_ NCs, oleylammonium iodide (OAM-I) was used for halide exchange to form CsPbI_3_ by adding the ligand to the colloidal solution. In this halide-exchange process, the surface anion vacancy concentration was significantly reduced from a starting Br/Pb ratio of 2.78 to a final I/Pb ratio of 3.00. This, in part, accounts for the PLQY increase from 38% for CsPbBr_3_ to 80% for CsPbI_3_. Although the EQE of the CsPbI_3_ LEDs matched their bulk thin film counterparts, the device stability was limited, with the performance halving after only 5 min at 1.25 mA cm^–2^ current density.^1078^

Surface engineering has also been important for improving the performance of green emitters (510–530 nm wavelength). Successful strategies include: (1) eliminating labile OLA (oleylamine) from the synthesis (EQE = 0.32%),^346^ (2) treating NCs with ammonium thiocyanate (EQE = 1.2%),^1104^ (3) employing octylphosphonic acid post-synthesis (EQE = 7.74%),^1105^ (4) using didodecyldimethylammonium ligand during synthesis (EQE = 9.80%),^1106^ and (5) triple ligand–surface treatment (EQE = 11.6%).^1107^ Combining these organic ligands with inorganic passivation agents has also been shown to be effective in improving EQE. The addition of ZnBr_2_ to DDA-Br-capped NCs resulted in the improvement of the EQE of the green LEDs from 10.7 to 16.48%.^396^ Introducing excess FABr to the precursor solution was also found to be effective, with PLQYs increasing from 62% to 74% in films, and device EQEs increasing from 1.5 to 17.1%, as the FABr/PbBr_2_ molar ratio was increased from 1:1 to 2.2:1.^1108^ XPS measurements indicated that there was a reduction in the concentration of bromide and formamidinium vacancies, which may be due to these being filled by the excess FABr. There was also a lower surface ligand density, which may have resulted in improved charge transport between the NCs. The operational stability was also improved from 52 s (control (FABr/PbBr_2_ molar ratio 1:1)) to 1080 s (FABr/PbBr_2_ molar ratio 2.2:1), due to the suppression of nonradiative recombination as the excess FABr passivated the surface defects. However, it was found that this was not due to any improvements in thermal stability, which was found to be unaffected by the addition of FABr from thermogravimetric analysis.^1108^ Indeed, Dong *et al.*(1101) found that a limitation of ligand exchange is that the process results in the removal of surface bromide anions, which results in lower PLQYs. They showed that this could be overcome by mixing the NC solution with a saturated solution of isopropylammonium bromide in DMF or NaBr in DMF after multiple reprecipitation steps to heal the surface bromide vacancies. As a result, their 4 and 7 nm CsPbBr_3_ NCs exhibited near-unity PLQYs after ligand exchange, resulting in blue LEDs with 12.2% EQE (480 nm wavelength).^1101^ Beyond these surface treatments, Zheng *et al*. decorate nickel oxide on the CsPbBr_3_ NC surface through adsorption and a sequential oxidation treatment. This resulted in EQE increasing from 0.7 to 16.8% with a drop in turn-on voltage from 5.6 to 2.8 V.^1109^

There has also been increased recent focus on blue-emitting perovskites: it is a fact that the EQEs of these devices currently limit the development of perovskite-based displays and solid-state white lighting. A key challenge is the low PLQYs of Cl-based perovskite emitters. Recent efforts to address this limitation include passivation with K^+^, Cl^–^ (from CuCl_2_), Ni^2+^, and Mn^2+^ ions.^782,1095,1096^ Yang *et al.*(1096) recognized that a challenge with using oleic acid and oleylamine (the most common ligands) in the synthesis of perovskite NCs is that the protonation process between the acid and amine (*i.e*., the surface-bound ammonium ion giving back the proton to the surface bound carboxylate ion) can result in ligand desorption and the formation of surface defects. The introduction of K^+^ (through K_2_CO_3_) was found to passivate surface defects and also reduce the density of organic ligands required on the surface (as found from Fourier transform infrared spectroscopy), which improved charge transport between NCs in films. It is thought that K^+^ bound to halide ions on the NC surface can passivate dangling bonds. As a result, the PLQY of the colloidal NCs increased from 9.50% (no K^+^) to 38.4% with 8% K^+^, which correlated with increases in the EQE from 0.23% (no K^+^) to 0.82% (8% K^+^).^1096^ However, the highest EQE was achieved with 4% K^+^ (1.19% EQE) due to improved surface morphology, for which the emission wavelength was 476 nm.^1096^ With surface passivation, the LT_50_ also improved by 2.6 times up to 4.5 min with an applied bias of 4 V. Further improvements in EQE were achieved by replacing the TPBi electron injector with PO-T2T, which has higher mobility that is better matched with the poly-TPD hole injector. By also adding a layer of poly(9-vinylcarbazole) between the poly-TPD and emitter, the EQE reached a peak of 1.96%.^1096^

De *et al.*(782) demonstrated that the addition of CuCl_2_.2H_2_O to the reaction mixture during the synthesis of CsPbCl_3_ by hot injection, led to an increase of the PLQY of CsPbCl_3_ NCs from 0.5% (no doping) to 60% (1% Cu doping) at 400 nm wavelength (violet). It was also found that with Cu doping, the NCs became halide-rich rather than halide-deficient, and the improvement in PLQY is attributed to the reduction in the density of anion vacancies on the surface. Bi *et al.* reported improvements in PLQY in mixed Cl–Br NCs emitting in the 430–460 nm range, which reached 92 and 98%, respectively, after incorporating CuCl_2_. Improvements in the air-stability of the NCs were also seen, but the effect on device performance was not reported.^1110^

Hou *et al.*(1095) also demonstrated improvements in the PLQY and, consequently, the EQE of blue-emitting CsPbBr_1–*x*_Cl_*x*_ NCs through Mn^2+^ doping (by hot-injection synthesis). The PLQY improved from 9% (no Mn^2+^) to 28% (with 0.19% Mn^2+^). This correlated with improvements in the EQE from 0.50 to 2.12% at an emission wavelength of 466 nm. The emission fwhm was also narrow (18 nm). The high-performance blue LEDs were used to excite red and green perovskite NC down-converters, resulting in CIE coordinates of (0.311, 0.326), close to the values for true white emission. The white-light LED was calculated to have an EQE of 0.25%. Despite the promising device performance, the stability was limited, with the devices degrading within seconds to minutes. Another important challenge is the Mn^2+^ content, which needed to be controlled very carefully. Having Mn^2+^ content above 0.2% resulted in a reduction in the PLQY and a longer-wavelength emission from the Mn^2+^ ion center increasing in intensity significantly.^1095^ More recently, Zheng *et al.*(1100) reported the passivation of Cl^–^ vacancies using *n*-dodecylammonium thiocyanate (DAT). The thiocyanate component is a “pseudo-halide” capable of filling halide vacancies but has the important advantage of not shifting the emission peak (unlike the use of organic halides). DAT was introduced to CsPb(Br_*x*_Cl_1–*x*_)_3_ quantum dots post-synthesis because the long-chain doedecylammonium component of DAT enabled it to dissolve in toluene, the same solvent of the quantum dots. After post-treatment, the PLQY of the quantum dots increased from 83% (as-synthesized) to 100% (post-treatment), whereas the PL peak remained at the same wavelength (468 nm). The EQE improved from 3.5% (without treatment) to 6.3% (with DAT treatment), with an electroluminescence wavelength of 470 nm. DAT treatment also resulted in an improvement in device stability from 17 s (without treatment) to 99 s (with DAT treatment), and this was attributed to reduced ion migration due to a reduced concentration of Cl^–^ vacancies.

##### NPl Emitters

In addition to being grown as symmetrical, three-dimensional NCs, LHPs can also be synthesized as 2D nanoplatelets (NPls). The common morphology of NPls can be seen in Figure 126d. The thickness of these NPls can be finely tuned from one monolayer (approximately 0.6 nm) to several monolayers. These perovskite NPls exhibit quantum confinement when the thickness is smaller than the Bohr radius (typically 2–3 nm),^47^ enabling a blue shift in the emission. This is currently simpler and more reproducible than growing perovskite NCs smaller than 3 nm.^60,103^ Perovskite NPls have therefore gained significant attention for blue-emission applications, by allowing pure-bromide perovskites to emit at between 400 and 475 nm wavelength.^60^ In 3D perovskite NCs larger than the Bohr radius, achieving these blue emission wavelengths requires using Cl-based or mixed chloride–bromide perovskites.^103^ An important limitation is that Cl vacancies form deep traps that result in low PLQYs.^103,1095^ Although these limitations could be addressed through passivation, bromide-based perovskite NPls are an important alternative. However, NPls have a higher surface area to volume ratio, and exhibit pronounced surface defects. Originally, this limited the PLQYs to low values of 20% or less.^47,209^ However, Bohn *et al.* demonstrated that the PLQYs can be substantially increased up to 75% through surface passivation by adding PbBr_2_ complexed with organic ligands to the colloidal solution.^60^ Wu *et al.* also demonstrated that surface Br vacancies could be passivated using HBr, resulting in PLQYs up to 96% at a PL wavelength of ∼460 nm,^398^ which is suitable for blue-emitters in ultrahigh definition displays.^499,1111^

The use of passivation in bromide-based perovskite NPls has led to improved performance with significantly improved color purity. An early report of perovskite NPl LEDs used MAPbBr_3_ and MAPbI_3_ NPls complexed with long-chain butylammonium ligands. These NPls were denoted L_2_[MAPbX_3_]_*n*−1_PbX_4_, where X is the halide (either Br^–^ or I^–^), L the butylammonium ligand,^1112^ and *n* the number of monolayers. It is noted that other groups would refer to these as simply MAPbX_3_ NPls.^60,398^ However, the Br-based perovskites contained a mixture of NPls with different thicknesses, with electroluminescence from *n* = 2, 3, and 4 layers. The EQEs were all well below 0.01%.^1112^ Yang *et al.* subsequently developed a hot-injection approach to synthesize monodisperse CsPbBr_3_ NPls using the long-chain oleylamine, oleic acid, and octadecene as the ligands. By controlling the reaction temperature, they were able to fine-tune the number of monolayers in the NPls, with fewer layers obtained at lower reaction temperatures. Using a reaction temperature of 180 °C, CsPbBr_3_ NPls with a thickness of 3.1 nm were obtained, which gave EL in LEDs at 480 nm. In both the PL and EL spectra, only one emission peak was obtained, and according to TEM analysis, there was a narrow distribution in the NPl thicknesses. The performance of the LEDs reached 0.1%, with a maximum luminance of 25 cd m^–2^.^216^ Through passivation of the CsPbBr_3_ NPls using HBr, Wu *et al.* achieved an improvement in EQE to 0.124%, with 62 cd m^–2^ luminance. This was made possible using thinner NPls with bluer emission at 463 nm. Color-pure emission was also achieved, with the fwhm of the EL peak being only 12 nm. As such, the CIE coordinates (0.157, 0.045) fulfilled the requirements for ultrahigh definition displays.^398^ However, the EQE falls well below the near-unity PLQY. Hoye *et al.* investigated the limiting factors in CsPbBr_3_ perovskite NPl LEDs. They found that when using PEDOT:PSS as the hole injector, there was significant nonradiative decay, leading to the PLQYs of the NPls nearly halving. By adding a poly(triarylamine) layer between PEDOT:PSS and the NPl, nonradiative recombination was reduced, as found from time-resolved PL measurements. This led to an improvement in the EQEs by 2 orders of magnitude, from 0.007 to 0.3%, with 40 cd m^–2^ luminance for blue emitters (464 nm EL wavelength).^1102^ Similar results were obtained from sky-blue emitters (490 nm wavelength). Further improvements in EQE for the sky-blue emitters were achieved by adding PbBr_2_ complexed with oleylamine and oleic acid for surface passivation, as previously detailed by Bohn, Tong, *et al.*(60) However, it was found that only a small amount (10 vol %) could be added to the NPl solution to improve the LED performance of the sky-blue emitters (from 0.24 to 0.55%).^1102^ Further increases in the volume of the PbBr_2_–ligand passivating agent led to a reduction in performance. By contrast, Bohn *et al.* redispersed all of their purified perovskite NPls into a solution of PbBr_2_–ligand in order to achieve the maximum improvement in PLQY.^60^ It was also found that adding PbBr_2_–ligand to the blue-emitters led to no improvement in performance. While the reason behind the limitation in the amount of passivating agent that could be added is unknown, possibilities include the formation of an insulating shell around the NPls that make charge-injection challenging.

Another approach used to passivate surface defects in CsPbBr_3_ perovskite NPls was to use soft Lewis bases. Zhang *et al.* used DDAB to partially replace the original oleylamine ligands through liquid-phase ligand exchange of the colloidal NPls. The replacement of shorter DDAB ligand and the corresponding TEM images before and after the ligand treatment can be seen in Figure 126d. This increased the PLQY of blue-emitting perovskite NPls from 45.1 to 69.4%, with a consequent increase in the device EQEs by an order of magnitude to 0.56%. Further improvements in EQE to 1.42% (shown in Figure 126i) were achieved by adding a layer of CBP between the poly-TPD hole injector and NPls. The role of the CBP was attributed to a reduction in the hole injection barrier, owing to the higher HOMO level of 6 eV. Furthermore, the stability of the LEDs also improved, with the time for the EL to reach half the peak value increasing from 15 to 42 s at a constant current of 1 mA cm^–2^. Also, the PL stability was improved over time after ligand treatment, as shown in Figure 126e,h,g.^1103^ While short, these lifetimes are among the longest for blue perovskite NPls reported to date. Nevertheless, they are shorter than those achieved by sky-blue emitting perovskite thin films,^1113^ and significant improvements in device operation stability are needed before the NPl devices can be used commercially. It is believed that these effects are due to the DDAB ligands binding to surface bromide vacancies (XPS showed an increase in the Br/Pb ratio after adding DDAB), as well as to exposed lead cations on the surface.^1103^

An important challenge in the early development of perovskite NPl LEDs was poor knowledge of the exact band positions.^103^ This was recently addressed through the use of Kelvin probe to measurements of the work function, and through X-ray photoemission spectroscopy to measure the valence band to Fermi level offset of blue and sky-blue emitting CsPbBr_3_ NPls. According to these measurements, both emitters have deep ionization potentials of 6.8 eV (blue) and 6.5 eV (sky blue). As a result, conventional hole-injectors would give rise to a large hole-injection barrier, whereas conventional electron-injecting materials would have a lower electron affinity or LUMO than the conduction band minimum of the NPls (3.8–3.9 eV). This was found to result in significant charge imbalance, which limits the EQEs of the devices, and indicates that future efforts need to focus on developing higher hole-injection level materials.^1102^ Another alternative is to change the ligands to tune the band positions. Zhang *et al.* showed that partially substituting oleylamine for DDAB resulted in a reduction of the ionization potential of CsPbBr_3_ NPls from 7.1 to 6.8 eV. Nevertheless, the hole-injection level remained deep.^1103^

Beyond CsPbBr_3_, perovskite NPls using both Pb^2+^ and Sn^2+^ cations, and with halides ranging from I^–^ to Br^–^ to Cl^–^ have been grown, demonstrating PL emission wavelengths that can be tuned from 690 to 400 nm, although it should be noted that Cl-based NPls were not emissive.^209^ There is therefore potential to use perovskite nanoplates beyond solely blue emission (as it is in the cases that have been discussed previously), although there has been less focus on device development, since green, red, and near-infrared emitting thin films and NCs have already reached >20% EQE. Nevertheless, the ability of perovskite NPls to blue shift the emission of pure-halide materials may be advantageous in avoiding phase segregation and broadening of PL peaks that could be observed in mixed-halide perovskite thin films. However, further work is needed to improve the purity of iodide-based perovskite NPls, with recent examples demonstrating broad PL fwhm values (50 nm at 650 nm wavelength) or multiple emission peaks.^54^

##### NCs Embedded in 3D Matrices

**Figure 127:**
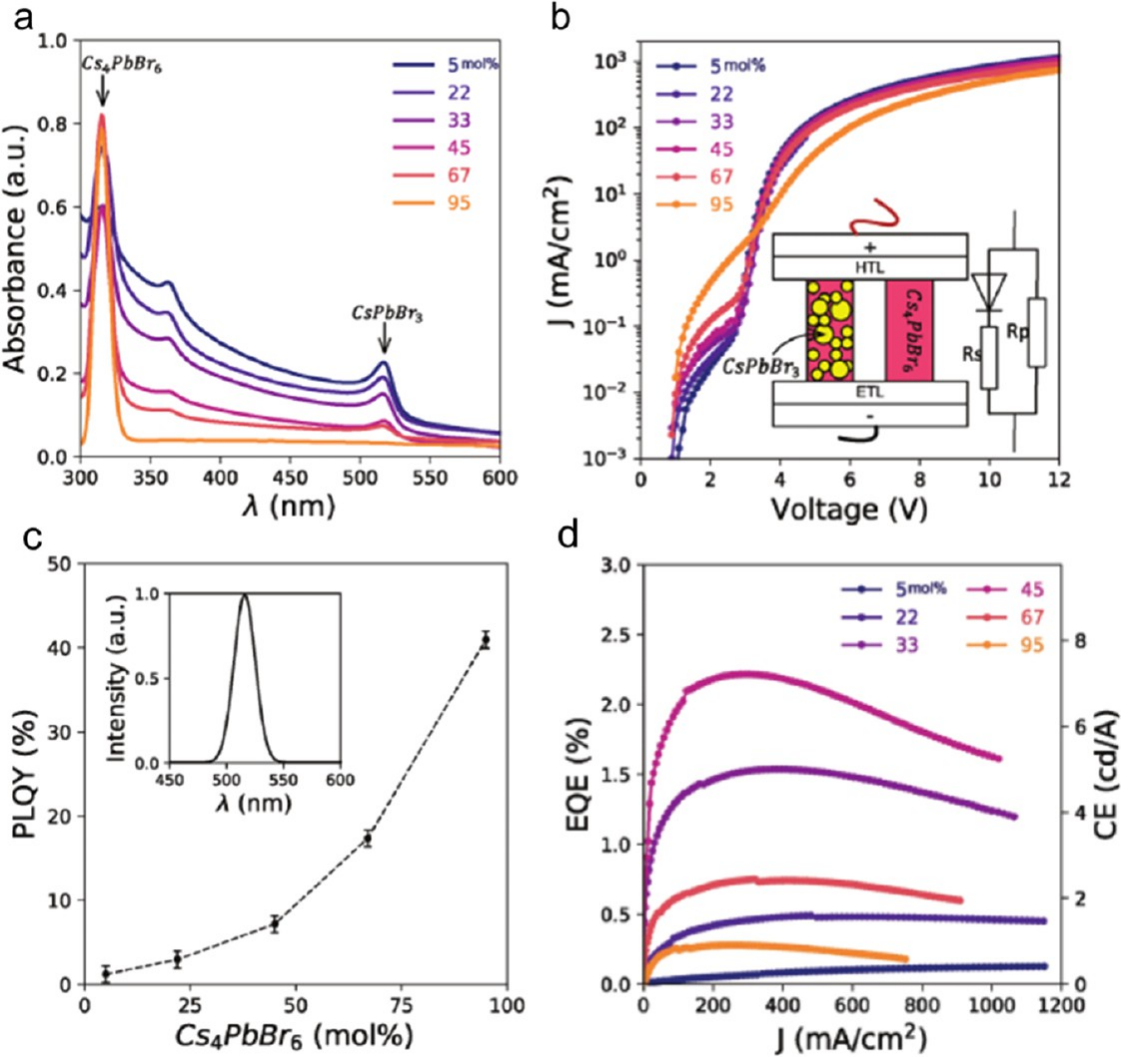
(a) Absorbance of CsPbBr_3_|Cs_4_PbBr_6_ composite with different CsBr and PbBr_2_ precursor ratios. (b) Current density for devices based on the composite perovskites with different Cs_4_PbBr_6_ molar percentages. Inset: Schematic showing two types of current conducting channels (CsPbBr_3_-rich zone and Cs_4_PbBr_6_-rich zone) through the perovskite layer in LED devices. The former channel could form a typical LED structure with a series resistor, whereas the latter one would serve as a shunt resistor due to the lack of emitter. (c) PLQY of composite films increased with an increased percentage of Cs_4_PbBr_6_. Inset: Photoluminescence spectrum of composite films showing sharp peak near 516 nm with fwhm = 20 nm. (d) EQE for devices with respect to Cs_4_PbBr_6_ molar percentage. Reproduced with permission from ref (1114). Copyright 2018 John Wiley & Sons, Inc.

3D NCs embedded within a matrix of a lower dimensionality perovskite have been demonstrated, as discussed above. This enables charges to be more effectively confined in the 3D NCs, while having a well-controlled structure. An example that has gained attention recently is CsPbBr_3_ embedded within a matrix of Cs_4_PbBr_6_,^1114^ which is a wide-band-gap 0D non-perovskite. The absorbance due to Cs_4_PbBr_6_ is shown in Figure 127a. This composite structure has been shown to result in significantly improved PLQY. For example, Lian *et al.* found that CsPbBr_3_ grown by thermal evaporation has a PLQY of 1.2%, whereas 5 mol % CsPbBr_3_ embedded in Cs_4_PbBr_6_ has a PLQY of 40% (Figure 127c). This has been attributed to spatial confinement of charges, as well as the passivation of surface defects.^1114^ In devices, this correlated with a significant improvement in device performance, from 0.13% for CsPbBr_3_ LEDs to 2.5% for the composite devices with 55 mol % CsPbBr_3_ (Figure 127d, a sketch of the device structure and device current density at different composite ratio are shown in Figure 127b).^1114^ Similar improvements in performance were also observed by Shin *et al.*, from 0.0062% EQE for CsPbBr_3_ to 0.36% for the composite, which was consistent with the improvement in the PLQY to 55% for the composite. Optical modeling found the outcoupling of these devices to be similar, between 9 and 12%, and the calculated internal quantum efficiencies were 0.072 and 2.9%, respectively. From this, it was calculated that the injection efficiency was lower for the composite, in agreement with the wide band gap of the Cs_4_PbBr_6_ host.^1115^ Both Lian *et al.* and Shin *et al.* grew the composite films through the evaporation of CsBr and PbBr_2_ in alternate layers and adjusting the ratio of the thicknesses of each layer. However, Shin *et al.* reported that a limitation with this technique is that CsPbBr_3_ formed in the Cs_4_PbBr_6_ is not stable and is affected by exposure to moisture. Indeed, they reported that the as-grown film (that was nominally Cs_4_PbBr_6_) was originally yellow-colored CsPbBr_3_ that became transparent Cs_4_PbBr_6_ with embedded CsPbBr_3_ after 15 min in ambient air. After several days in air, the film had completely become Cs_4_PbBr_6_ and no green emission was observed.^1115^ This therefore shows the limitation of Cs_4_PbBr_6_/CsPbBr_3_ composites prepared by the sequential deposition approach, even though Lian *et al.* reported that the composite was more stable under operation than CsPbBr_3_.^1114^

Composites comprising PbS quantum dots heteroeptiaxially incorporated in perovskite matrices have also been demonstrated with success.^1116^ These structures are particularly advantageous for devices emitting in the near-infrared at wavelengths (900–1560 nm) longer than achievable with pure lead perovskite emitters.^1117^ Such long wavelength emitters are important for applications in night vision, biomedical imaging, optical communications and computing,^1118^ and the ability to achieve these devices using low-cost solution-based methods could be significantly advantageous over the epitaxial structures currently used.^1117^ PbS can form heteroepitaxially in MAPbI_3_ lattices because they have strong structural affinity and similar Pb–Pb bond distances (5.97 Å for PbS, 6.26 Å for MAPbI_3_) that are within 4.6% of each other.^1116^ Further improvements in lattice matching could be achieved by alloying I with Br in the perovskite due to reductions in the lattice parameter of the perovskite.^1118^ Theoretical considerations also showed that it is possible for PbS/MAPbI_3_ interfaces to form without defects. HRTEM measurements showed a well-defined orientation between PbS and MAPbI_3_. The growth of the MAPbI_3_ matrix around the PbS quantum dots was achieved by exchanging the organic ligands for short halide ligands. By mixing with PbI_2_ dissolved in butylamine, the PbI_2_ formed a complex with the halide species on the quantum dot surface. This complex was deposited onto a surface by spin-coating, followed by soaking in a solution of methylammonium iodide in isopropyl alcohol, thus forming the MAPbI_3_ matrix. Changing the ratio of PbI_2_ and quantum dot in the precursor changed the final content of the quantum dots in the matrix from 0.2 to 29%.^1116^ Spectroscopic measurements showed that the efficiency of carrier transfer to the PbS was up to 80%.^1116^ Demonstrations of near-infrared LEDs achieved EQEs up to 5.2% at 1390 nm emission wavelength, which was significantly higher than the PbS quantum dot only control (0.03%).^1118^ Further improvements in performance were achieved by embedding PbS quantum dots in a 2D perovskite matrix, with phenethylamine used as the stabilizing agent bound to the PbS quantum dots. This was mixed into the solution containing the inorganic precursors (CsBr, PbBr_2_), which was spin-coated, with toluene dripped as the antisolvent. GISAXS measurements showed that this resulted in quantum dots that were regularly and evenly spaced (on average 4.4 nm apart). Spectroscopic measurements showed that the exciton transfer efficiency from perovskite to quantum dot was 82% at 1533 nm emission wavelength, with LEDs achieving 3.5% EQE. For 1300 nm emission wavelength, the EQE was 6%, but the highest EQE was achieved for 986 nm emission, with a peak value of 8.08%. The increases in EQE with shorter wavelength were due to increased PLQY in the quantum dots.^1117^ These devices also demonstrated improved stability compared to earlier quantum dot in perovskite versions, with the EL intensity reaching half the peak value after 1 h of operation.^1117^

##### NC–Polymer Composites

**Figure 128:**
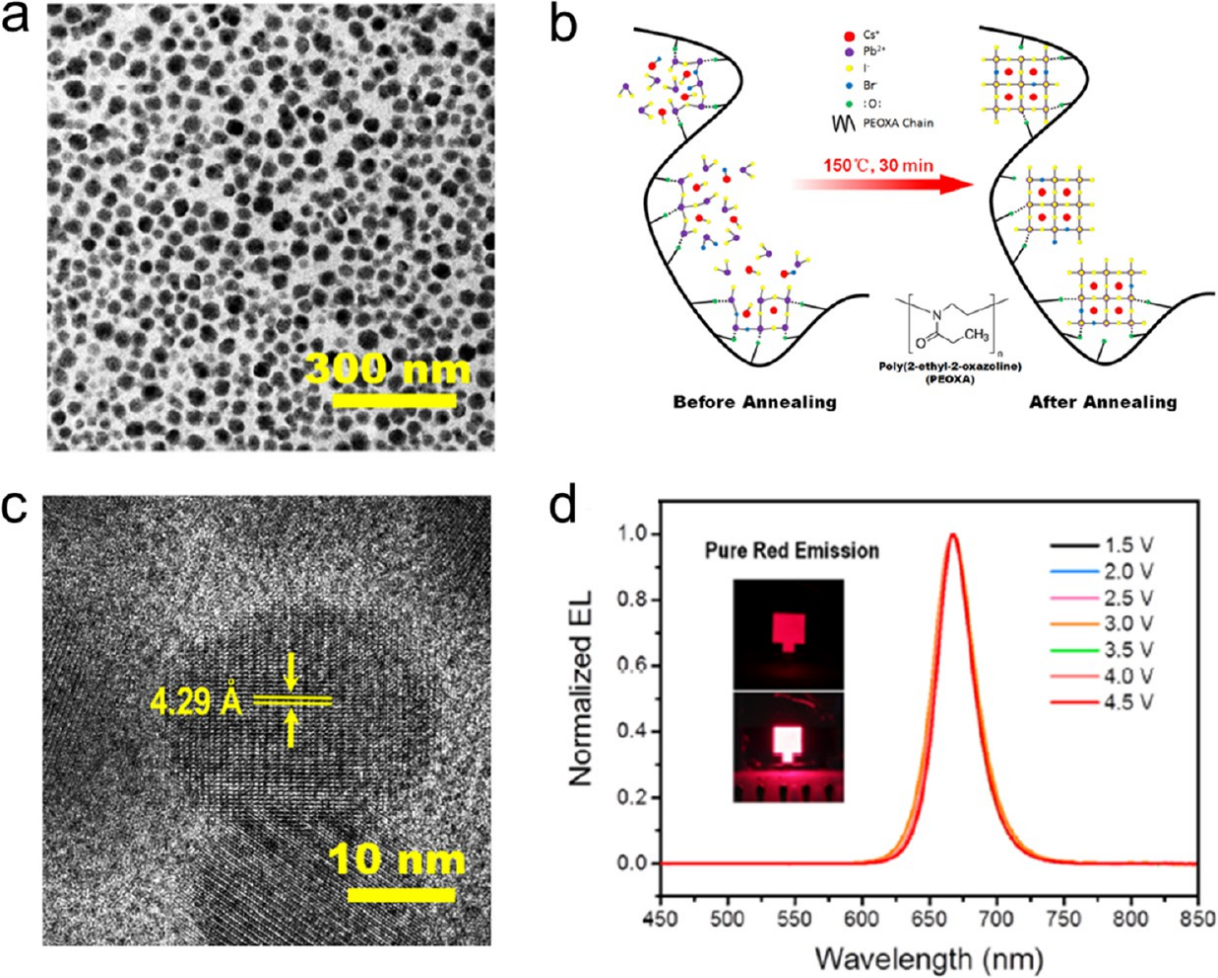
(a) TEM of CsPbBr_0.6_I_2.4_ film with 45% PEOXA. (b) Schematic diagram of polymer-induced *in situ* perovskite nanocrystal formation process. (c) High-resolution TEM image of crystals; the repeated distance of 4.29 Å indicates the (110) plane of CsPbBr_0.6_I_2.4_ lattice. (d) EL spectrum stability of the CsPbBr_0.6_I_2.4_ LED with 45% PEOXA. The inset is the photos of a light-up LED at voltage biases of 1.5 V (top) and 3.0 V (bottom). Reproduced from ref (1121). Copyright 2018 American Chemical Society.

Perovskite NC–polymer composites have been explored as a means to improve the stability of the NCs.^20,469,1119,1352^ Xin *et al.* demonstrated blends of CsPbBr_3_ NCs with the PMMA, PS, and poly(butyl methacrylate). These composites were able to maintain their quantum yield in air for more than a month.^1352^ Wang *et al*.^285^ also reported a swelling–deswelling microencapsulation strategy to fabricate MAPbBr_3_ NC/polymer composite films which were stable against moisture and heat. Perovskite–polymer composites have also been shown to result in reduced nonradiative recombination and improved device performance. Zhao *et al*. demonstrated this with perovskite thin films. They embedded a 2*D*/3D bulk perovskite into an insulating polymer matrix, resulting in near-infrared LEDs with EQEs reaching 20.1%.^1079^ The polymer component suppressed nonradiative recombination at the interfaces between the perovskite emissive layer and charge transport layers. Li *et al.* demonstrated improved performance in perovskite LEDs using perovskite NC/polymer composites. They fabricated a composite of MAPbBr_3_ NCs and an aromatic polyimide precursor (PIP). By adding the PIP polymer matrix, the EQE was increased by 2 orders of magnitude compared with pristine MAPbBr_3_ NCs in a thin film, giving an EQE of 1.2%.^1120^ Cai *et al.* blended CsPb(Br,I)_3_ NCs in different ratios with the polymer poly(2-ethyl-2-oxazoline). The TEM images are shown in Figure 128a,c. This resulted in improved EQEs in pure-red LEDs from 1.04% (0 wt % polymer) to 6.55% (45 wt % polymer).^1121^ The enhancement of EQE and stability are attributed to strong interactions between the functional group in the polymer matrix and the Pb^2+^ in NCs, which facilitates homogeneous distribution of NCs and increases the PLQY (Figure 128b). The EL spectra is stable under different operational voltage as shown in Figure 128d. In addition to homogenous distribution of NCs, Rainò *et al.* suggested the improvement of spectra stability is due to that the high hydrophobicity and efficient molecular packing of the polymer matrix with the long-chain NC surface ligands are the key factors for protecting the NCs against environmental damage.^1122^ The polymer matrix also provides excess nucleation sites during the NC recrystallization process, which leads to more uniform NC distributions in the films, resulting in a higher PLQY in thin films of the composite.^1121^ Another promising application of the perovskite NC/polymer composites is as down-converters. Through excitation with commercial blue LEDs, these down-converters efficiently produce sharp green and red photoluminescence, which is important for display applications.^278,1123^ Start-up companies are beginning to explore the commercial potential of perovskite NC/polymer composite phosphors.^1100^ However, devices are still limited by the thermal stability of the composite materials. For example, LEDs using MAPbBr_3_ NCs/Polyvinylidene fluoride composites undergo thermally induced degradation when temperature exceeds 70 °C.^278^

#### Optical Features of Perovskite Light Emitters

##### Highly Efficient Light Emission

The emergence of LHP NC systems as a novel class of light-emitting materials may offer additional technological possibilities, as reflected by the enormous enhancement of photoluminescence quantum yield in the past 5 years. The defect-mediated nonradiative losses in the bulk LHPs are often considerable, but in the NC systems, strategies including composition engineering, ligand passivation, quantum and dielectric confinement, and post-treatments of LHP thin films and NCs, have shown promise. For example, Hassan *et al*. achieved a η_PL_ of >93% in cubic MAPbI_3_ NCs synthesized by the LARP technique.^1124^ In the mixed-cation NCs, *e.g*., FA_0.5_MA_0.5_PbBr_3_, near-unity η_PL_ was also achieved.^1125^ Pan *et al*. synthesized highly luminescent red CsPbI_3_ NCs with η_PL_ of >95% using bidentate 2,2′-iminodibenzoic acid as ligands to passivate NC surfaces.^172^ Near-unity η_PL_ was also reported in CsPbI_3_ NCs,^1126^ as well as other CsPbX_3_,^78^ by stabilizing the cubic phase using trioctylphosphine lead iodide precursor and passivating the surface with alkylammonium ligands using the hot-injection methods. Additional strategies, such as selective chemical etching^1127^ and spray pyrolysis synthesis,^1128^ were also reported to significantly enhance η_PL_ to near-unity.

In quantum-confined LHP NC systems, η_PL_ enhancement generally requires more efforts. For example, the quasi-2D PEA_2_A_1.5_Pb_2.5_Br_8.5_ NCs, where A = MA and Cs, exhibit a high η_PL_ of 88%.^1082^ In the 2D (RNH_3_)_2_[MAPbBr_3_]_3_PbBr_4_ NPl system, where R is an alkyl chain, and η_PL_ in the assembled superlattices can reach 90%,^1129^ hypothetically due to a special aggregation-induced emission mechanism. An important merit for 2D material-based emitters is that the exciton transition dipole moments (TDMs) can be aligned parallel to the surface plane, guiding the emission perpendicular to the out-of-plane direction, which greatly enhances the light outcoupling efficiency in LEDs.^1125,1130^ Recent advances in 2D CsPbBr_3_ and MAPbBr_3_ NCs have shown that one can obtain a high degree of in-plane TDM ratio in their superlattices, showing promise for future photonic devices.^751,1131^

##### Narrow Emission Band

Bright and narrow-band fluorophores as primary colors emitting at pure red (R), green (G), and blue (B) wavelength regions are critical to enable next-generation displays with extremely high chromaticity. The emergence of LHP NC-based LEDs is mainly driven by their intrinsically narrow-band emission, whose fwhm ranges from 9 to 42 nm, from B to R.^23,105,411^ Notably, an extremely narrow fwhm of 11 nm had been reported in the layer-controlled 2D CsPbBr_3_ NC solutions.^60^ In LEDs, the fwhm of 14.7 nm has been realized using the mixed anion CsPbBr_3_/Cl_3_ NCs with appropriate ligand engineering.^1132^ Sim *et al*. reported bright EL based on CsPbX_3_, giving narrow fwhm values of 16, 16, and 40 nm for B, G, and R primaries, respectively.^1133^ A report demonstrated that the PL fwhm decreased from 36 to 32 nm when the CsPbI_3_ NCs were encapsulated by varying the amount of ammonium thiocyanate.^1134^ Zhang and co-workers achieved a very narrow EL fwhm of 33 nm for the R primary at 648 nm using the CsPb(Br/I)_3_ NCs.^1098^ By cross-linking the CsPbI_3_ perovskite NCs with trimethylaluminum, the fwhm further reduced to 31 nm for the R primary.^1067^ In the NIR wavelength region, by modulating the anion and cation compositions, the EL fwhm was reported to as low as 27 nm in the Cs_*x*_FA_1–*x*_Pb(Br_1–*y*_I_*y*_)_3_ NCs, optimized by an automated microfluidic platform.^1135^

**Figure 129:**
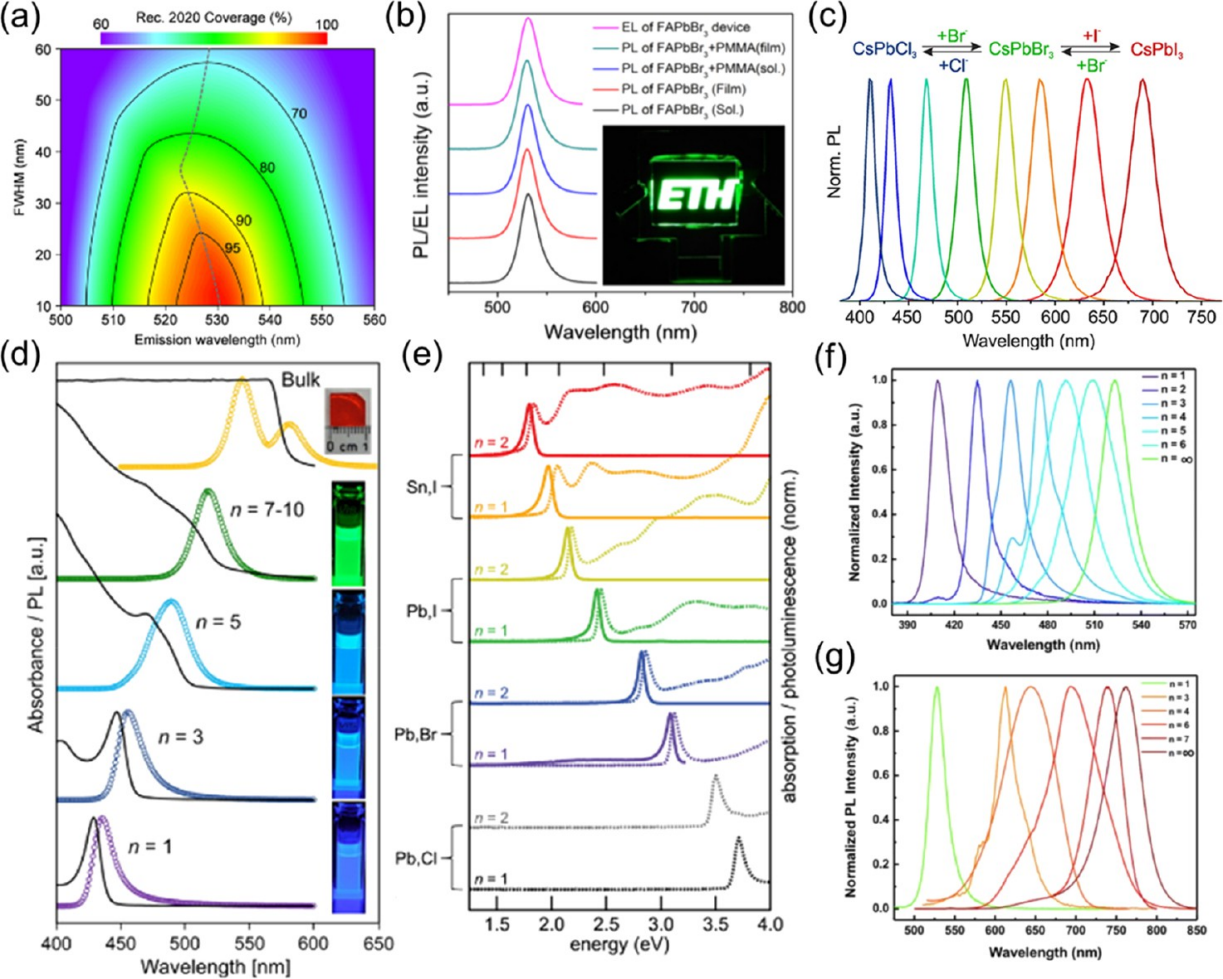
Fundamental characteristics of lead-halide perovskites. (a) Calculated Rec. 2020 color gamut coverage in CIE 1931 color space as a function of fwhm and emission wavelength for the green emitter. (b) PL and EL spectra of FAPbBr_3_ NCs that achieved Rec. 2020 gamut area coverage >97%. Reprinted from ref (900). Copyright 2017 American Chemical Society. (c) Tunable PL spectra in the colloidal CsPbX_3_, where X = Cl, Br, and I, NCs using fast anion exchange either from bromide to iodide (red shift) or bromide to chloride (blue shift). Reprinted from ref (55). Copyright 2015 American Chemical Society. (d) Absorption and tunable PL spectra of 3D bulk single-crystal and colloidal solution of 2D MAPbBr_3_ NCs with precise layer control between *n* = 7–10 and *n* = 1. Reprinted from ref (211). Copyright 2016 American Chemical Society. (e) Absorbance and highly tunable PL spectra of 2D LHPs by varying the B-site cations, Pb and Sn, and anions, Cl, Br, and I. Reprinted from ref (209). Copyright 2016 American Chemical Society. (f,g) Tunable PL spectra in the layered quasi-2D perovskite (Br-based (f) and I-based (g)) NCs. Reprinted with permission under a Creative Commons CC BY license from ref (1138). Copyright 2018 The Authors.

Although narrow electroluminescence peaks can be realized in the green-emitting CsPbBr_3_ NCs, the resulting color gamut area would only cover 90% of the recommendation (Rec.) 2020 standard, the newly defined color gamut for next-generation displays, because the emission peaks are at <520 nm wavelength.^52^ Using the colloidal 2D FAPbBr_3_ NCs with a fwhm of 22.8 nm peaking at 529 nm, a coverage of >98% Rec. 2020 has been reported (Figure 129a,b).^900,1136,1137^ We consider that the perovskite NC emitters would be the most promising candidate reaching 100% of the Rec. 2020 color gamut among all semiconductor systems.

##### Tunable Emissive Spectra

The emission spectra and corresponding optical band gaps in the LHPs are continuously tunable over the entire visible spectral region from 400 to 780 nm. A few strategies, including stoichiometric mixing and quantum confinement were utilized to tune the optical band gap of the perovskite NCs, as amply discussed in previous sections. For example, Nedelcu *et al*. demonstrated emission wavelength tunability in the CsPbX_3_ NCs by fast anion exchange at 40 °C (Figure 129c).^55^ The NCs exhibit η_PL_ of 10–80% and fwhm of 12–40 nm. A similar approach was reported by the Akkermann *et al*. by exchanging bromide anions using iodide and chloride precursors.^57^ Similar approaches were also carried out in the MAPbX_3_ and FAPbX_3_ systems.^29,163^ For the RP-phase quasi-2D NPs the PL emission can also be tuned between 410 and 523 nm for (BA)_2_(MA)_*n*−1_Pb_n_Br_3n+1_ (Br series) (Figure 129f), and between 527 and 761 nm for (BA)_2_(MA)_*n*−1_Pb_n_I_3*n*+1_ (I series) (Figure 129g).^1138^ Note that although the anion exchange enables viable band gap tunability, the high η_PL_ of the mother NCs is not always preserved.^55^ Moreover, the solubility of chloride precursors in the common polar solvents is generally low, making it more difficult to prepare blue emitters.^899^ The emission spectra can also be modulated by temperature (temperature-dependent PL studies have mainly been used to investigate the excitonic properties of LHPs).

One-dimensional quantum confinement by controlling the lattice layer number in 2D NPs is another attractive approach to enable emission blue shift.^16,18,48,743,895,1124^ Note that the 2D NPs are different from the RPPs, which are quasi-2D phases comprising stacked 2D layers. Considerable efforts have been made in the 2D MAPbX_3_, CsPbX_3_ systems using the LARP, nonsolvent crystallization, and hot-injection technique.^209,1139^ For example, the Tisdale group identified the colloidal 2D MAPbBr_3_ perovskites with layer numbers (*n*) of 4, 5, and 6 emitting at 475, 490, and 504 nm, respectively.^19^ By gradually varying the octylammonium ligand concentration between 100 and 0%, the colloidal 2D MAPbBr_3_ NCs were isolated giving emission between 427 and 519 nm for *n* = 1 to ∞.^16^ The Tisdale group also demonstrated thin layers of *n* = 1 and 2 using the nonsolvent crystallization method (Figure 129e).^209^ The Shih group reported high η_PL_ of up to 90% in the 2D NC solutions of *n* = 1, 3, 5, and 7, yielding stable room-temperature EL at 436, 456, 489, and 517 nm, respectively (Figure 129d).^899^

#### Electrical Features of Nanocrystal Perovskite Light Emitters

##### Charge Carrier Dynamics

While there has been tremendous progress in the performance of perovskite NC LEDs, future improvements will require a more in-depth understanding of the intrinsic photophysics of these materials, and also how charge carriers are transported across interfaces within the devices. The recombination rate of free carriers can be described by eq 3:^1140−1142^
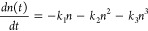
3where *t* is time, *n* is charge carrier density, *k*_1_ is recombination rate of exciton recombination or trap-related recombination, *k*_2_ is the bimolecular recombination rate of free charge carriers, and *k*_3_ is the Auger (multi charge carrier) recombination rate. By comparing the charge carrier dynamics of polycrystalline perovskite bulk thin films and perovskite NC films using steady-state and transient photoluminescence spectroscopy, Kim *et al*.^1142^ and other researchers found out that both exciton recombination and bimolecular recombination occur in bulk thin films, while exciton recombination is dominant in NC thin films.^29,1062,1142,1143^ Further details on the physics of hot carrier relaxation and exciton recombination are given in Charge Carrier Dynamics section.

As the main radiative recombination of perovskite NCs is due to exciton recombination, it is important to understand the source of band-edge exciton generation inside NCs. During photoexcitation, photons with energy higher than the band gap will create hot carriers. The interactions between carriers (carrier–carrier interactions) and the surrounding lattice (carrier–phonon interactions) play an important role in hot carrier cooling processes in perovskite NCs which generate band-edge excitons or cold carriers.^50,1144^ The radiative recombination of these single band-edge excitons is the main contribution of photon generation in a perovskite NC light-emitting diode. Another pathway to create band-edge excitons is through biexciton or multiexciton generation processes. When the incident photon energy is higher than 2*hn* during the photoexcitation process, the excess energy of the generated hot carriers can create additional excitons.^912^ Then, the bi/multiexcitons will recombine nonradiatively through an Auger process and form band-edge excitons.^872,1145^ The hot carrier cooling rate can be influenced by several factors including excitation energy,^872^ halide compositions^852^ and types of cations.^1146^ The reader can consult the OPTICAL PROPERTIES section for more details on this topic.

Carrier trapping will also influence the carrier dynamics in perovskite NCs for light-emitting applications. It occurs when the band-edge excitons do not recombine radiatively and instead migrate to a trap state which is close to the band-edge.^21,872^ As perovskite NCs still suffer from a broadening in the photoluminescence peak, it is important to understand what gives rise to this effect.^1147,1148^ Wehrenfennig *et al*.^1148^ suggested that the homogeneous PL broadening could be increased through phonon creation and annihilation which would generate side peaks, or through polaronic effects where the photogenerated electron–hole pair is strongly coupled to the surrounding lattice, causing a geometric lattice relaxation and a Stokes-shifted emission from the absorption edge. A typical Stokes shift for CsPbBr_3_ NCs with effective edge length between 4 and 13 nm ranges from 20 to 80 meV.^785^ Brennan *et al.* reported that the size-dependent Stokes shift is intrinsic to the NC electronic structure and independent from extrinsic influences such as solvents and impurities.^785^ Another factor which can influence the band structure and hence the Stokes shift is temperature. Naghadeh *et al.*(1149) reported that the PL spectra will exhibit a blue shift for small NCs (∼3.1 nm) with decreasing temperature from 300 to 20 K, while exhibiting a red shift with decreasing temperature for medium-sized (5.1 nm) and large (9.2 nm) NCs. The size of NC will also influence the carrier dynamics as the PL lifetime increases with temperature for larger NCs, and it remains the same for the small and medium-sized NCs.

The majority of investigations into perovskite NC carrier dynamics are performed on solutions or thin films under photoexcitation. Future insights into the carrier dynamics of the NCs under electrical excitation are also needed. Sharma *et al.*(990) recently demonstrated that the NCs aggregates in the thin film did not blink in PL but showed strong blinking in EL. This is because that all NCs can be photoexcited spontaneously and emit photons during the photoluminescence process. However, only a small fraction of the NCs within the aggregates can undergo electroluminescence, the majorities remain dark permanently, resulting in blinking. By investigating CsPbBr_3_ NCs system (∼16 ± 5 nm), they reported that the selective EL process is due to charge migration and selective recombination. During the electroluminescence process, the injected charges will migrate to larger NCs that have smaller band gaps. As a result, the larger NCs function as traps where the charges migrating over other NCs get accumulated and recombined. It shows that under comparable excitation rates, the intrinsic ELQY is only 36% that of the PLQY.^990^ During photoluminescence, simultaneous emissions can occur on all NCs after photoexcitation and exciton recombination. However, when injecting carriers, only a larger NC will emit as it acts as a trap center due to its lower band gap energy.

##### Role of Contact Layers and Charge Balance

**Figure 130:**
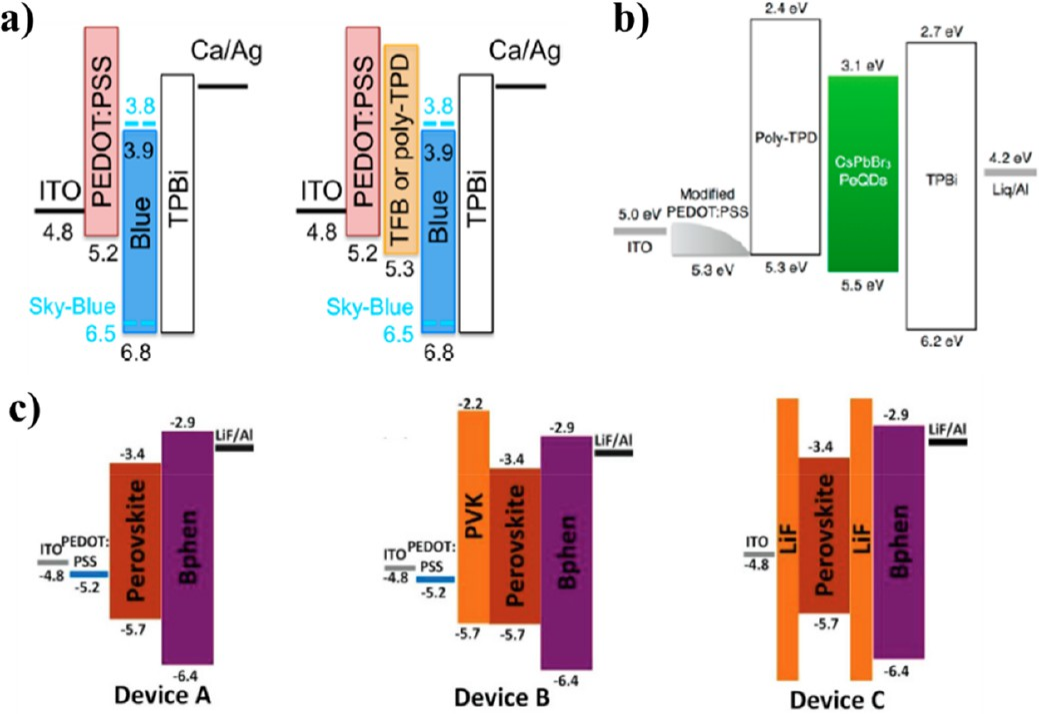
Band structure illustration of perovskite LEDs with different interfacial layers. (a) Blue and sky-blue emitting perovskite NPl LEDs with or without an interfacial layer of TFB or poly-TPD. Reprinted under a Creative Commons CC-BY license from ref (1102). Copyright 2019 American Chemical Society. (b) HTL modification: energy diagram for modified hole injection layer (Nafion blending PEDOT:PSS). Reproduced from ref (190). Copyright 2017 American Chemical Society. (c) Comparison of devices with different interfacial layers. Device A: PEDOT:PSS/perovskite/Bphen. Device B: PEDOT:PSS/PVK/perovskite/Bphen. Device C: LiF/perovskite/LiF/Bphen. Reprinted with permission ref (1163). Copyright 2018 John Wiley & Sons, Inc.

Charge balance and the charge injection barrier are two parameters that are strongly linked together because they are determined by the position of the perovskite bands relative to the band positions of the materials for injecting electrons and holes. The most common organic and inorganic charge injection materials are detailed in several reviews, *e.g.*, reference.^801,1150−1152^ From these, is evident that the most common charge injectors enable efficient electron injection over the full range of perovskite electron affinities (down to 3.1 eV for MAPbCl_3_),^1150,1153^ but hole-injection is more challenging. While the hole-injection level for typical materials is up to 5.4 eV (for TFB and poly-TPD),^1150^ the perovskite ionization potential can reach values as high as 6.8 eV for blue-emitting CsPbBr_3_ perovskite NPls.^1102^ Green-emitting perovskites also have higher ionization potentials (*e.g.*, 5.9 eV for MAPbBr_3_).^1150^ Higher hole-injection levels have been achieved through modifications in common organic materials. For example, PEDOT:PSS has been mixed with MoO_*x*_ to increase the work function from 5.20 to 5.62 eV.^1115,1154^ As another example, Nafion perfluorinated ionomer (PFI) has been used to modify the surface of TFB. The surface dipole from PFI gives rise to band bending of the TFB beneath to a higher work function, resulting in an improvement in the performance of blue-emitting CsPbBr_3–*x*_Cl_*x*_ NCs.^1155^ Similarly, Chiba *et al.* also reported using Nafion blending with PEDOT:PSS to modify the workfunction of PEDOT:PSS, as shown in Figure 130b.^190^

Charge balance is measured by constructing two single-carrier devices from the same perovskite emitter. One device has hole-injecting and hole-selective contacts on both sides (*e.g.*, ITO/PEDOT:PSS/perovskite/MoO_*x*_/Au). The other has electron-injecting and electron-selective contact which have deep ionization potentials or HOMO levels to block holes (*e.g.*, ITO/ZnO/PEI/perovskite/TPBi/Ca/Ag). By controlling the polarity of the applied bias, hole or electron injection from each of the injecting layers is measured, and the current densities for electrons and holes are compared. Unbalanced current densities would result in the recombination zone being close to the electrode with the less efficient injection. For example, a higher electron current density would imply that the recombination of injected electrons and holes occur at hole-injector interface. In such cases, it is important to ensure that the electron affinity or LUMO of the hole-injector is sufficiently low to confine carriers within the active layers in order to avoid parasitic emission from the injecting layer.

The size of the injection barriers may be inferred from the built-in potential of the device, which is measured through electroabsorption spectroscopy,^1156^ or is determined through photoemission spectroscopy measurements of the individual layers. Details and best practices of the latter approach are given in reference.^870^ It should be emphasized that owing to strong spin–orbit coupling, perovskites often have significant tailing in the density of states at the valence band maximum, and accurately determining the valence band to Fermi level offset would require fitting the density of states to the valence spectrum rather than through simple linear fits.^1102,1157^ Another approach to measure the work function is to perform Kelvin probe measurements, which has the advantage of measuring the work function of the layers under ambient conditions that may be more representative of the films in devices. Details on best practices on Kelvin probe measurements on perovskites are given in reference.^1158^

Careful choice of the charge-injection layers is necessary not only to minimize injection barriers and control charge-balance, but also to minimize nonradiative recombination at the interfaces. PEDOT:PSS is one of the most common hole-injection materials deposited beneath the perovskite active layer but has in many cases it been shown to give high rates of nonradiative recombination with both bulk 3D perovskites and perovskite NPls,^1102,1159^ leading to lower external PLQYs and fast PL decay. This is due to the semimetallic nature of PEDOT:PSS and high density of defect states that would occur at the interface.^1159^ The effects of nonradiative recombination at the interface with PEDOT:PSS has been addressed through the use of poly(triarylamine) interlayers between PEDOT:PSS and perovskite. For example, the use of TFB or poly-TPD resulted in an increase in the PL decay time of blue-emitting CsPbBr_3_ perovskite NPl thin films deposited on top, which led to the device EQE improving by 2 orders of magnitude, as shown in Figure 130a.^1102^ Similarly, it has been found that adding a 20 nm layer of poly-TPD between PEDOT:PSS and MAPbI_3_ in solar cells resulted in a significant reduction in leakage current, along with an increase in the open-circuit voltage.^1159^

Work on reducing interface recombination has also focused on passivating the perovskite, though this has to date largely been demonstrated in photovoltaic systems. This includes the use of surface passivating species such as alkali metal-halide additives and generation of 2D/3D surfaces that significantly reduce nonradiative recombination at the interfaces.^1160−1162^ Another important consideration for the device performance is the charge leakage, which refers to the escape of holes and electrons from the perovskite layer to the charge transport layer. To solve the leakage issue, Shi *et al.* proposed an LiF double insulating structure shown in Figure 130c.^1163^ The sandwiched FAPbBr_3_ perovskites are protected by LiF layers to avoid leakage, which increases the EQE to 5.53% device C, compared to that of devices A and B, which is 0.174%.

##### Ion Conductance and Hysteresis

Typically, perovskite LEDs are only measured in one voltage direction. In many cases, this is due to the device degrading toward the higher voltage end of the measurement. However, measurements of the forward and reverse sweep of nondegraded perovskite LEDs have shown hysteresis to be present,^31^ similar to observations made in perovskite solar cells. In photovoltaics, hysteresis is attributed to ion migration, owing to the high density of halide ions and vacancies present in the perovskite material.

Changing the distribution of ions at the interface impacts charge collection (in a solar cell) or injection (in an LED); in certain configurations, this may be more favorable, but typically, this creates unwanted barriers to charge movement at the interfaces.^1164^ Furthermore, these interfacial halides and vacancies may also lead to nonradiative recombination sites as the very ions or defects may introduce trap states in the band gap, particularly at surfaces.^1165^ We note that such ion migration effects can also be seen as an opportunity, as demonstrated by light-emitting electrochemical cells,^1166,1167^ in which the devices are designed such that the local distribution of ions allows for favorable injection and emission properties. However, achieving control over the ionic movement will be critical for its practical use.

**Figure 131:**
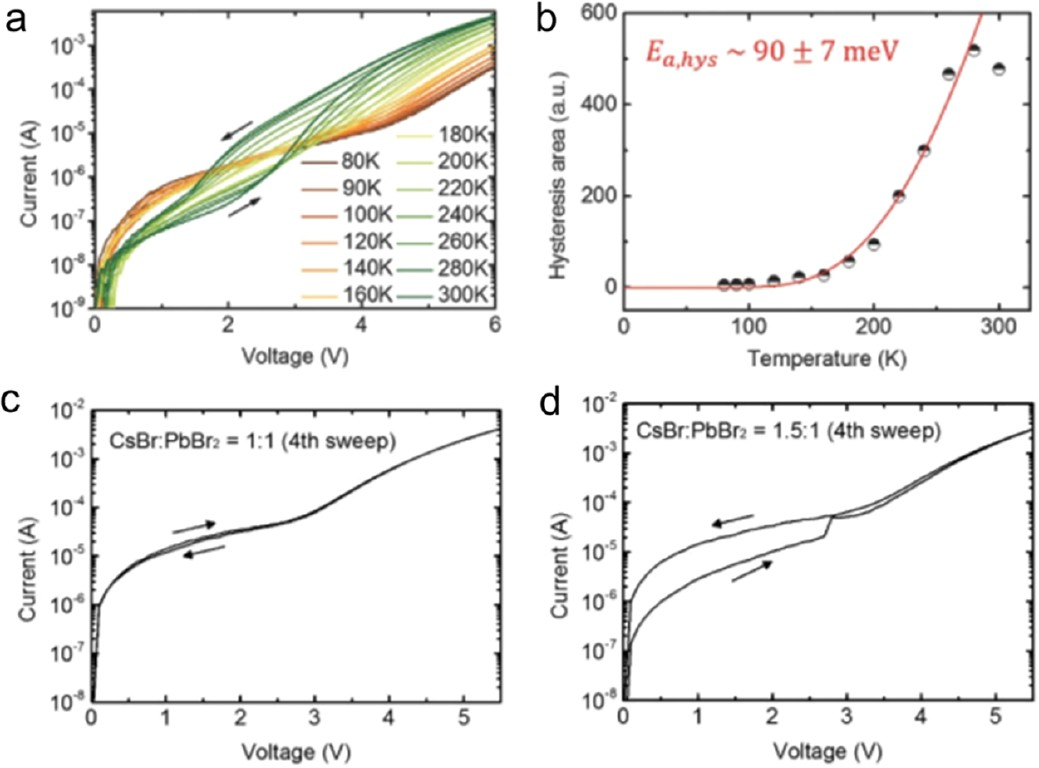
(a) Temperature-dependent current–voltage characteristics of the CsPbBr_3_ PeLED showing current hysteresis. (b) Plot of hysteresis area *versusT* with a nonlinear fitting based on Arrhenius equation. (c) Hysteresis behavior of a CsPbBr_3_ PeLED with CsBr:PbBr_2_ = 1:1 (based on Buf-HIL) at room temperature with four sweeps. (d) Hysteresis behavior of a CsPbBr_3_ PeLED with CsBr/PbBr_2_ = 1:1.5 (based on Buf-HIL) at room temperature with four sweeps. Reproduced with permission from ref (1168). Copyright 2017 John Wiley & Sons, Inc.

Work by Cho *et al.*(1168) on CsPbBr_3_ thin film LEDs showed that the degree of current hysteresis increased exponentially with temperature, following an Arrhenius relationship that had an activation energy of 90 ± 7 meV. This is close to the reported activation energy for halide anion migration in MAPbBr_3_ and it was proposed that the migration of Br^–^ accounts for the current hysteresis observed at different temperatures (Figure 131a,b). When the ratio of CsBr/PbBr_2_ was increased from 1:1 to 1.5:1 in the precursor solution, the current hysteresis from the resultant films became worse (Figure 131c,d), possibly due to an increase in trap density. With higher CsBr/PbBr_2_ ratio, the hysteresis increased up to fourth sweep compared with low CsBr/PbBr_2_ ratio.^1168^ Chen *et al.* also found that ions migrated with the application of an electric field of 0.3 V m^–1^ vertically in a MAPbBr_3_ microplatelet, enabling the formation of a p–i–n junction, which could be frozen in place by rapidly cooling to −193 °C. This operated as an LED, with negligible current hysteresis at −193 °C, but significant hysteresis at ambient temperature, which is again consistent with ion migration giving rise to the observed hysteresis.^1169^ Such ion migrations results in halide segregation in LHP NCs with mixed-halide composition. Under photoirradiation or with an applied bias, mixed-halide perovskites present a main limitation due to the segregation of the mixed phase into two phases, as initially reported by Hoke *et al*.^1170^ For example, in the ensemble film of CsPbBr_1.2_I_1.8_ NCs, Zhang *et al.*(1171) observed that the laser excitation causes a blue shift from 630 to 520 nm in the PL peak that can revert back in the dark. Interestingly, for an isolated single CsPbBr_1.2_I_1.8_ NC, the PL is also blue-shifted upon laser excitation but never returns back in the dark, revealing the fact that the presence of adjacent NCs is crucial to channel the migration of iodide ions. Furthermore, they observed blue-shifted PL when the NCs were electrically biased in the dark without the injection of excited-state charge carriers. This finding suggests that the local electric field breaks the iodide bonds that triggers the ion migration process.^1171^ Gualdrón-Reyes *et al*. found that such segregation is a size-dependent phenomena and is minimized in thin films of smaller size NCs.^1172^ Similarly, the spectral instability of the PeLEDs is observed under varying bias when mixed Cl/Br halide is used for blue EL. Wang *et al*. reported EL red shift as a function of Cl content caused by strong electrical field.^1173^ It was found that the deeper blue device appeared to be more subjected to the field-induced phase separation.

#### LEDs Exploiting Lead-Halide Perovskite Emitters

By virtue of superior features in light generations and electrical characteristics, lead-halide perovskites, especially the NCs, were supposed to be contemporary soft light emitters in flexible thin film light-emitting diodes.^396,404,499,1085^ In addition to the cost advantage endowed by cheap raw materials, facile synthesis of emitters and solution processing film deposition, LHP-LEDs also demonstrate high luminous efficiency, high-color purity, and ultrawide color gamut for prospective full-color display, white lighting, and other applications.^169,175,404,499,1083,1084,1128^ Thus, far, some impressive achievements have been reported in the few years, including a high external quantum efficiency level over 20%, ultrahigh brightness level over 100 000 cd m^–2^, a good flexibility, a facile device fabrication, but an incongruous operation stability.^396,404,1078,1117,1143,1147^ Because of the environment-friendly consideration of lead component, some lead-free metal-halide perovskite emitters were also developed and great progresses, *e.g*., high-color rendering index over 90, were achieved.^499^ However, limited by a high-quality film deposition, these emitters are more compatible with inorganic LEDs as phosphors.^499,1105^ This section mainly concentrates onto the LEDs exploiting LHP emitters.

##### Classifications

Like other solution-processed thin film devices, such as QD-LEDs and polymer solar cells, the device structures of most LHP-LEDs are simple, and their primary difference are the emitters. Thus, the classification of LHP-LEDs is mainly based on the colors, dimensions, film deposition technologies, and other features of LHP emitters.

##### Color of LHP Emitters

Normally, the EL spectra of LHP-LEDs are almost same with the photoluminescence spectra of adopted LHP emitters, with band gap being predominately determined by the halogen species.^1126,1157,1174^ For the LHPs with single halogen species, three discrete and narrow emissive bands with a width of around 20–30 nm go across the whole visible range, which means almost the whole color gamut is covered.^14,443,1175^ However, except the green bromine-based LHPs, the near-ultraviolet chlorine-based and near-infrared iodine-based LHPs are too extreme for most application of LEDs.^78,1092,1173^ Using alloyed halogen species, the emissive band of the resulting LHPs can be tuned across the whole visible range; correspondingly, their color gamut is also extended.^78,1083,1092,1173^ However, compared to the single halogen species LHP-LEDs, the EL spectra of LHP-LEDs based on alloyed halogen species LHP emitters demonstrate an irreversible shift because of the migration of halogen anions and vacancies under an applied electric field.^1078,1099,1142,1171^ To date, LEDs using single halogen species emitters, especially the APbBr_3_ green ones, still dominate the development of LHP-LEDs by virtue of high EQEs over 20% and high device operation stability.^404,1079^ As the last piece of LHP-LED jigsaw in the prospective full-color display applications, the progress of blue LHP-LEDs is still lagging behind the red and green ones, because of a low luminous efficiency and poor stability of chloride-based blue LHP emitters.^1067,1083,1096^ Alternatively, APbBr_3_ NPls and other nanostructures with a strong quantum confinement are held in great consideration as prospective blue emitters in LHP-LEDs.^216,899,1082,1113,1177^

##### Dimension of LHP Emitters

**Figure 132:**
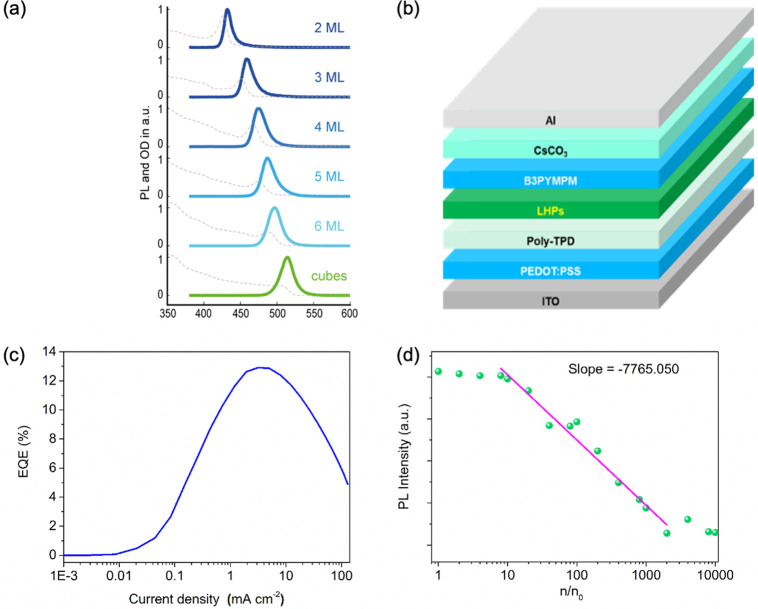
(a) PL (solid) and absorption (dashed) spectra of CsPbBr_3_ colloidal nanoplatelet with different thicknesses. (b) Scheme structure of LHP-LED. (c) EQE current density characteristics of LHP-LEDs. (d) PL intensity dependence of MAPbBr_3_ film on electron number, and *n*_0_ is the density of electron injected when the current density is 1.0 × 10^–2^ mA cm^–2^. Panel a is reprinted with permission under a Creative Commons CC-BY license from ref (60). Copyright 2018 The Authors. Panel d is reprinted from ref (1187). Copyright 2018 American Chemical Society.

Because of a low exciton binding energy (around dozens of meV), most excitons generated by photon excitation or electrically driven in bulky LHPs would dissociate into free charge carriers, leading to a low efficient radiative recombination.^894,896,1133,1178,1179^ Also, trap-assisted nonradiative recombination in polycrystalline LHPs with high density of defects additionally competes with the radiative processes.^1178,1180−1182^ Nanocrystalline LHP grains with dimension less than 10 nm, *e.g*., quantum dots, quantum well and NPls, confine charge carriers in a small volument, and this enhances exciton binding energy to hundreds of meV and facilitates exciton radiative recombination.^60,1178,1179,1183^ Moreover, the surface defects of nanocrystalline LHPs can be passivated effectively using long-chain molecule ligands. Quasi-2D LHP NPls with a strong quantum-confinement shift the emission toward high energy even by 200 meV compared to their 3D NCs counterparts.^60,1183^ By varying the number of [PbX_6_]^4–^ octahedral layers in these APbBr_3_ NPls, their emission color can be adjusted from green to deep-blue, providing an alternative pathway for blue LHP-LEDs (Figure 132a).^16,216,398,1102^ Synthesis approaches have achieved a level of control such that NPls narrow thickness distributions and characterized by narrow emission spectra, can be prepared.^60,216,398,747,1102^ The presence of long alkyl chain spacers, confers also excellent stability against ambient moisture but on the other hand it blocks the injection of charge carriers into the NPls.^49,1184−1186^

##### Deposition of LHP Emitter Films

The emissive films of most LHP-LEDs are deposited by solution processing, especially the organic–inorganic hybrid ones, which mainly includes *ex-situ* deposition using a prepared nanocrystalline LHP colloidal solution and *in situ* deposition using precursor solution.^404,1078,1079,1082,1187^ For the former, the synthesized high-quality nanocrystalline LHP, *e.g*., NPls, is dispersed into a low polarity solvent, *e.g*., toluene or tetrahydrofuran, to form a uniform colloidal solution for subsequent film deposition.^1078,1079,1187^ Normally, the concentration of these colloidal solutions must be high enough to deposit a continuous and uniform LHP film. In the meanwhile, to get a good charge carrier transport of the deposited LHP film, the amount of insulating long-chain ligands is kept at a low level, although leads to a poor stability of these colloidal solution, especially the NPl because of their propensity of self-assembly into stacks.^60,1129^ By changing the preferred orientation of LHP-NPls into random or using a semiconductive molecular spacer, the emission from the resulting LEDs can be improved.^1184,1188^ For the latter, all precursors are resolved in a polar solvent, *e.g*., dimethylformamide or dimethyl sulfoxide, to form a uniform solution for film deposition.^398,1082,1121^ Generally, an antisolvent crystallization treatment using a low polarity solvent, *e.g*., toluene, or solution is adopted during the film deposition.^189,385,1082,1084^ Moreover, an annealing post-treatment of the deposited LHP film is also required to enhance the quality of LHP films.^189,385,1082,1084^ In addition to solution processing, inorganic CsPbX_3_ film can also be deposited by vacuum thermal evaporation. However, in this case the polycrystalline film that is obtained has high density of defects, without an effective spatial confinement of excitons and charge carriers, and exhibits a much lower emissive efficiency compared to the solution processed films prepared with surface-passivated nanoscale emitters.^1189−1192^

##### Device Structures and Fabrications

The guideline of device structure design and fabrication of LHP-LEDs are developed within the framework originating from OLEDs and limited by the deposition of emissive layer, thus the device structures of most LHP-LEDs are simple. Normally, an LHP-LED contains multilayer thin films with a total thickness of around 100–200 nm sandwiched by two planar electrodes. Like other soft emitters, except rigid ITO glass, LHPs also demonstrate a good compatibility with flexible substrates.

##### Device Structures

**Figure 133:**
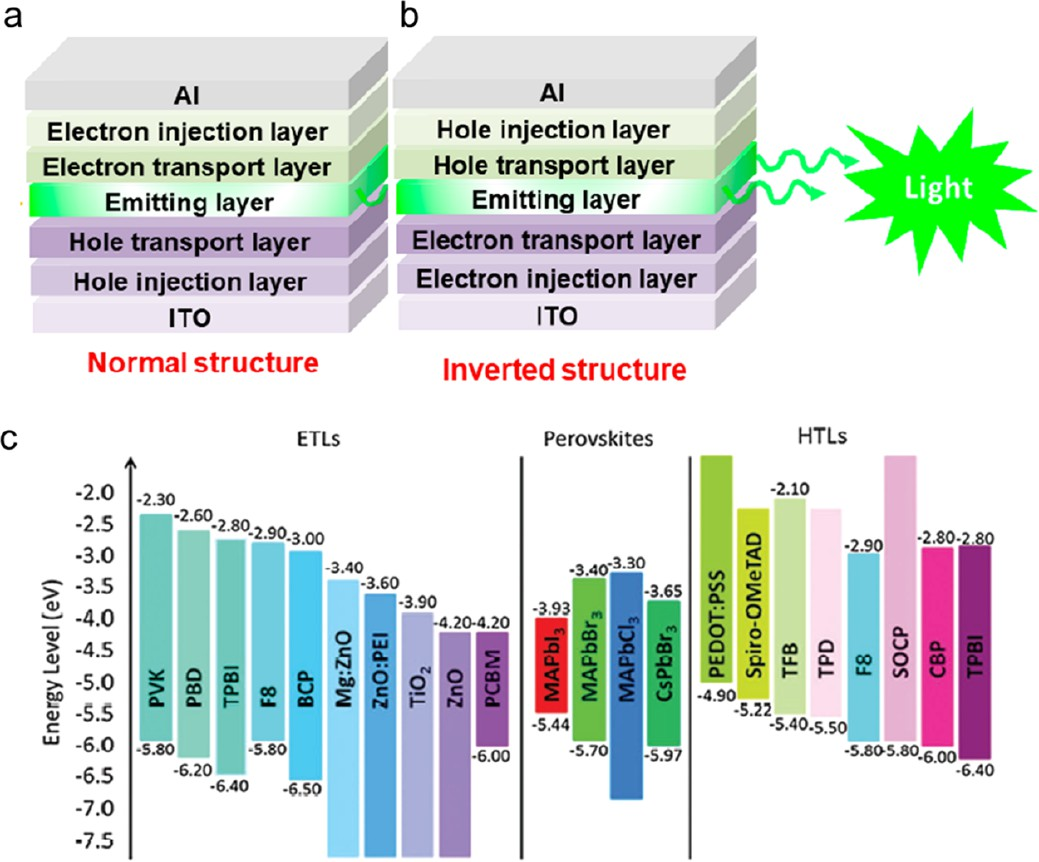
Device structures of perovskite LEDs. (a) Normal structure, (b) inverted structure. Reprinted with permission under a Creative Commons CC BY license from ref (1202). Copyright 2018 MDPI. (c) Energy level alignment of various materials used as perovskites, ETLs, and HTLs in the reported HPLEDs. Reproduced with permission from ref (1201). Copyright 2017 Elsevier.

To avoid the near-field quenching caused by electrode, in most LHP-LEDs a conductive poly(3,4-ethylenedioxythiophene):polystyrenesulfonate (PEDOT:PSS) film is selected as a spacer, which also can enhance hole injection from ITO anode (Figure 132b and Figure 133a,b).^404,1078,1082,1187^ In principle, the metallic PEDOT:PSS film is also regarded as an exciton quencher because of its highly electrical conductivity and interfacial defects.^1078,1193^ Therefore, an organic semiconductor film, *e.g*., poly(4-butylphenyldiphenylamine) (poly-TPD), with low density of charge carriers is adopted as a buffer layer to eliminate the exciton quenching caused by PEDOT:PSS.^396,1078^ Moreover, this organic hole transport film is supposed to enhance hole injection into the recombination zone because there is a large mismatch between the deep valence band of LHPs and the Fermi level of PEDOT:PSS.^396,1078,1082,1109^

To get a high EQE, a balanced charge carrier injection into the recombination zone is essential. In LHPs, holes and electrons have comparable mobilities, which helps to achieve a balanced charge carrier in LHP-LEDs.^11,1187,1194^ With consideration of the high conductivity of PEDOT:PSS, therefore, a high mobility/conductivity electron injection/transport layer, *e.g*., 2,2′,2″-(1,3,5-benzenetriyl)-tris(1-phenyl-1*H*-benzimidazole) (TPBi), is required to ensure a balanced charge carrier injected into the LHP layer.^396,1078,1082,1187^ For the cathode, a thermal evaporating deposited aluminum film with a buffer layer, *e.g*., lithium fluoride or caesium carbonate, is a popular choice.^396,1078,1082,1187^ Additionally, ITO can also work as cathode to in an inverted structure device.^1079,1195−1197^ Correspondingly, some functional layers were also required for a balanced charge carrier injection. Normally, a n-type semiconductor film, *e.g*., zinc oxide NCs, can be selected as matched electron transport layer.^1079,1195−1197^ Drawing inspiration from the PEDOT:PSS/poly-TPD combination used in normal structure devices, a polymer film, *e.g*., polyethylenimine ethoxylated, is required to modify ZnO NC film before the deposition of LHPs.^1079,1195−1197^

Without the limitation of solution processing deposition, in principle, an LHP-LED with more advanced device structure can be achieved using vacuum thermal evaporating deposition. Even in most solution processed LHP-LEDs, the deposition of metal electrode and other organic functional layers still need a vacuum thermal evaporation. In particular, using current solution processing technology, it is almost impossible to get a large-scale uniform emissive film with fine structure pattern for a LED display.

##### Device Fabrication

Generally, the solution processing deposition used for LHP films in LHP-LEDs includes spin-cast, inkjet printing and slot-die coating technologies.^1176,1187,1198^ So far, spin-cast is the most popular technology used for the solution processing film deposition in various soft material LED fabrication, including LHP-LEDs, QLEDs, and polymer LEDs.

At a practical level, for solution processing film deposition, the compatibility of film deposition plays a critical role in fabricating a successful LHP-LED. Normally, it is required that the surface energy of the deposited film must be higher than that of the solution used for subsequent film deposition. To increase the surface energy of polymer film, a charging treatment of oxygen plasma can be adopted. This however leads to the formation of surface defects that would increase the nonradiative recombination of emitters. Moreover, the deposited films are required to be highly passivated to withstand the solution processing of subsequent LHP film deposition. For example, with an annealing post-treatment, the passivation of poly-TPD and ZnO NC film is improved against subsequent solution processing on them.^396,1078,1079,1082,1109,1195−1197^ Because of their ionic crystal structure, LHPs are sensitive to high dielectric constant environment. For this reason, any processing of highly polar solvents onto LHPs are excluded from device fabrication.^1078,1199,1200^ Other functional layers, including top electrode, can also be deposited using solution processing deposition; however, their device reliability is not as good as the thermal evaporated ones.

For inorganic CsPbX_3_ LHPs, the films can also be deposited by a coevaporation of two precursors CsX and PbX_2_ or CsPbX_3_ in a high-vacuum chamber.^1189−1192^ The whole device, except some solution processing functional layers, *e.g*., PEDOT:PSS, can be deposited in a single run without breaking the vacuum, which is helpful to eliminate any potential negative influence caused by the atmosphere in the glovebox. In principle, the uniformity of LHP film and the reliability of resulting LEDs fabricated using the vacuum thermal evaporating are higher than those of the corresponding devices fabricated using solution processing technologies, especially in large-scale film deposition. Commonly used electron transport layers and hole transport layers with their corresponding energy levels are summarized in Figure 133c.^1201^

##### Luminous Efficiency Drop

A high EQE means a maximized output of photon number with respect to a minimized input of electrons number injected into devices, mainly including three factors for LHP-LEDs:
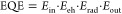
4In the expression above, *E*_in_ is the charge carrier balance factor in the recombination zone, and these injected charge carriers will form excitons with a possibility of *E*_eh_. The factor *E*_rad_ depicts the fraction of the intrinsic radiative efficiency of emitters, normally, which is equivalent to the PLQY of LHP emissive film. Though the emission of LHPs originates from exciton radiation, due to a strong spin–orbit coupling caused by heavy lead atoms, this electron transition obeys the conservation of total momenta rather than spin statistics.^147^ The last *E*_out_ determines the photon extraction efficiency of the device, which is dependent on the device structure and can be defined as 1/(2*n*^2^) (*n* is the refractive index of films).

If all charge carriers injected through electrode flow into the recombination zone, the *E*_in_ will be unity. EQE loss related to *E*_in_ is caused by leakage currents which depends on device structure and quality. In a low-quality device containing a large number of pinholes and trap states, the injected charge carriers would flow across the device *via* this bypass instead of being injected into the recombination zone. Due to an effective spatial confinement of nanocrystalline LHP domains, the charge carriers injected into the recombination zone will meet each other with a high possibility *E*_*eh*_ and form stable excitons. In a high driving current density level, the injected charge carriers would pass through the device without recombination as an overflow current, resulting in a drop in *E*_eh_ and EQE, which can be supposed to be another origin of leakage current.^1187^

The factor *E*_rad_ plays a dominating role in determining EQE of LHP-LEDs. At a low excitation intensity level, a trap-mediated nonradiative process dominates the exciton recombination, which is consistent with the low initial value of luminous efficiency, thus a high-quality LHP emissive film with a low density of defect is essential.^1187,1203−1206^ By increasing the excitation intensity, the exciton radiative recombination will dominate the trap-mediate process.^1187,1203,1205,1206^ A further increase of excitation intensity will result in a multiexciton Auger nonradiative process and luminous efficiency droop (Figure 132c).^1187,1203,1205,1206^ In the electrically driven devices, the injected charge carriers, especially the excess ones caused by imbalanced injection, will increase the probability of Auger nonradiative recombination even at a low driving current density level (Figure 132d).^1187,1207^

For almost of all planar multilayer structure LEDs, including OLEDs and QLEDs, most generated photons will be trapped inside devices by waveguide mode and substrate mode, only around 15–20% photons can be outcoupled because of the refractive index mismatch among functional layers, glass substrate and air.^1187,1208^ Similar to that with OLEDs, *E*_out_ can be enhanced using periodic nano- or microstructures, *e.g*., microlens array in this kind of multilayer planner structure LEDs.^1209^ Moreover, because of the overlap between absorption and luminescence spectra, which means an equivalently prolonged lifetime of excitons, the photons trapped inside device should have more chance to escape before annihilation by a recycling process.^1140^

In the working state of LHP-LEDs, one more factor that can result in EQE drop is the degradation of LHPs emitters caused by a considerable ion migration, which can be facilitated by applied electrical field and evidenced by a hysteresis dependence between driving current density and driving voltage in almost all electronoptic applications based on LHPs.^956,1210^

##### Stability of LHP-LEDs

Device operation stability is a very important consideration when evaluating a LED at a practical level, and achieving a good stability is still a severe challenge for LHP-LEDs.^98,469,956,1099^ Although LHP-based LEDs have a similar device structure to QLEDs the degradation is faster, and the degradation mechanisms may relate to the perovskite, as well as the interfaces between the perovskite and carrier injection layers. In general, the degradation mechanisms of perovskite LEDs are divided into four categories: (a) Ion migration, (b) interactions with surrounding moisture and oxygen, (c) electrochemical reactions, and (d) interfacial reactions.^1211^ Ion migration of halide ions in PeLEDs is intrinsically a defect migration process which is strongly related to perovskite surface chemistry and defects.^86^ It leads to defect creation (*e.g*., Frenkel defects), halide vacancy migration and lattice distortion which are detrimental to spectral stability and material stability. Halide ion migration can occur both within the perovskite emitting layer^1079,1212^ and across the organic transport layers.^1190,1213^ In addition, LHPs are sensitive to moisture, thus high-quality encapsulation is required for protecting the device against the environment.^98,469,956,1088,1099^ The heterostructure of 2D LHP-NPls and matrix-dispersed nanoscale LHPs can suppress ion migration effectively and provide additional protection for LHP emitters against environmental moisture.^189,398,1079,1189^ Moreover, as current-driven devices, the structural instability induced by mechanical stress is also a severe challenge for LHP emitters because of their ultralow thermal conductivity and Joule heating generated by devices under operation.^244,1214−1216^ Electroluminescence spectral stability is another challenge for colloidal perovskite LEDs, especially for deep blue (∼465 nm) and pure red (∼625 nm) emitters.^1217^ The instability of the EL spectra is primarily due to the halide segregation. Apart from ion migration, electrochemical reactions between migrated species from the perovskite and electrodes is another degradation pathway during device operation. Yuan *et al.* showed in bulk thin film MAPbI_3_ under electrical bias, the perovskite can react with electrodes to form I_2_ gas and PbI_2_, which makes the degradation process irreversible.^1218^ The interaction between the perovskite layer and transport layers can take place without external electrical bias, for example, the acidic nature of PEDOT:PSS layer can cause reactions with ITO over time upon direct contact, and the etched Sn and In ions can diffuse into perovskite layers and act as traps.^1219^ To suppress ion migration (halide segregation) and interfacial interactions, there are many methods that have been reported, such as compositional engineering, dimensional engineering, and defect passivation at NC surface and interfaces between the emitting and injection layers.^49^ However, currently, there is no individual strategy that can passivate all defects and suppress device degradation. It is critical to understand and utilize multiple strategies to further improve the stability of PeLEDs.

#### Summary and Outlook for Perovskite LEDs

LHP-LEDs have achieved incredible progress over the past few years, with excellent features, including highly efficient light emission, high-color purity, ultrawide color gamut, low cost of raw materials and fabrication methods, as well as good compatibility with existing OLEDs/QD-LEDs manufacturing technologies. In recent years, OLEDs, QD-LEDs, micro-LEDs, and other screenless display technologies are competing with each other. In particular, the great similarity between LHP-LEDs and CdSe QD-LEDs from device fabrication procedures to output features in a working state suggests a strong exclusiveness as prospective applications.

However, before evolving into practical products, LHP-LEDs need to overcome some critical bottlenecks, such as the concern of the toxic lead atoms, poor operation stability and large-scale panel fabrications, which has been attracting great attention, and some impressive progress has been achieved thus far. Until now, the performance of the lead-free perovskite-inspired materials have lagged behind the performance of their lead-based counterparts. The operational stability of LHP-LEDs is also a complicated issue because the device contains multilayer thin films and resulting heterogeneous interfaces. The extrinsic factors, including oxygen, moisture, *etc.* caused by ambient environment, can be fixed by following the well-established programs developed for OLEDs. The degradation of LHP emitters should be intrinsic among all possible factors, especially, which can be accelerated by applied electrical current and field in LEDs. The large-scale panel manufacturing is not an exclusive problem of LHP-LEDs, which also challenges for other solution processing LEDs, such as QD-LEDs and polymer LEDs. A nanoscale uniformity of all functional films contained in the LHP-LEDs is essential, because the pinhole and any other nonuniform morphology will lead to a highly deviated distribution of electric current flow and resulting brightness. A LHP-LED demo with spot size of square millimeters can be fabricated simply using spin-cast. However, when the spot area is increased to square centimeters level and even larger size, the deposition of such a large area film with a nanoscale uniformity is almost impossible using current technologies, including spin-cast, inkjet printing, *etc.*

Thus far, most works on LHP-LEDs have focussed on the enhancement of characteristic parameters, especially EQEs, at the technical level, however, the understanding of such enhancements are chained to the framework borrowed from OLEDs and QD-LEDs to a great extent. Actually, the performance enhancements of LHP-LEDs seem to have plateaued in the past years. Therefore, more fundamental work on LHP-LEDs is required for a better understanding of the working mechanism of such a contemporary LEDs. This would provide a guideline for the device works at the technical level and trigger a breakthrough in the device performance improvement in the future. For example, the above-mentioned stability issue of LHP-LEDs, though the same LHP emitters demonstrate a great stability under optical excitation, even in ambient atmosphere.

### Photodetectors and Field-Effect Transistors

Photodetectors convert light signals to electrical signals, which is critical for a diverse range of applications, such as sensors and optical communication devices.^1220^ Lead-halide perovskites are promising materials for photodetectors with high figure-of-merit (*e.g.*, responsivity and temporal response) owing to their strong optical absorption, high quantum efficiency, and ultralong carrier diffusion length.^994,1221,1222^ The initial reports on perovskite photodetectors were based on polycrystalline film, which indicates highest photoresponsivity of ∼3.5 A W^–1^ at 365 nm in the range of visible to the near-infrared region.^1223^ However, owing to polycrystalline structure, numerous crystal boundaries and defects exist in the perovskite film, which would serve as recombination and scattering centres in carrier dynamics, limiting the performance of the perovskite-based photodetectors.^1223,1224^ Low-dimensional perovskite NCs including nanocubes, nanowires, nanorods (1D), and nanosheets (2D) have recently been developed and tested for high-performance photodetectors. In particular, it has been demonstrated that lower defect density can be achieved than in their 3D counterparts, such as through surface passivation.

Fully inorganic CsPbX_3_ QD-based photodetectors have achieved high photocurrent on/off ratios of over 10^5^, thereby enabling effective switching.^37^ In order to increase the performance of the inorganic perovskite NC photodetectors, Kwak *et al.*(1225) and Wang *et al.*(1226) introduced conductive graphene as charge transport channel to enhance charge transfer, reaching a responsivity over ∼10^8^ A W^–1^. However, in general, perovskite NCs are coordinated with long-chain organic ligands, which could hinder charge transport and therefore lead to slow photoresponses (>1 s). With regard to fast carrier dynamics, it is crucially important to optimize ligand molecular and device configuration. In this framework, conductive nanonets made of carbon nanotubes (CNTs) in CsPbBr_3_ QD/CNT composites were used to improve charge extraction and transport, by which fast-response photodetectors with rise time of 0.016 ms have been achieved.^1227^

**Figure 134:**
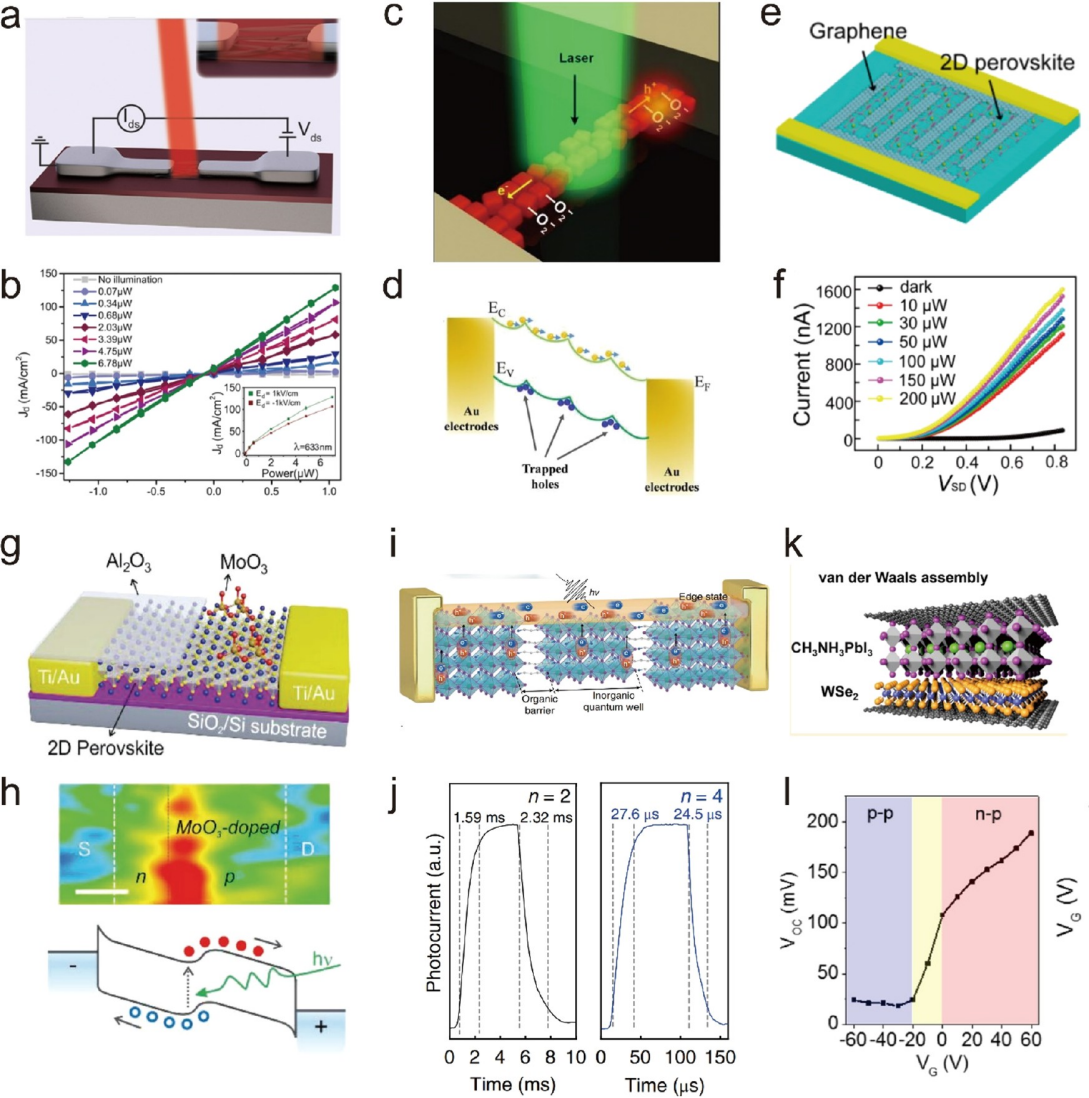
Photodetectors and field-effect transistors based on perovskite NCs. (a) Schematic diagram of 1D MAPbI_3_ wire photodetectors and (b) *I–V* curve of the MAPbI_3_ wire photodetectors under irradiation with laser wavelength of 633 nm. Reproduced from ref (1228). Copyright 2014 American Chemical Society. (c) Schematic diagram of 1D aligned CsPbX_3_ NCs photodetectors and (d) schematics of carrier dynamic in perovskite 1D NCs photodetectors under illumination. Reproduced with permission from ref (1232). Copyright 2019 Royal Society of Chemistry. (e) Schematic diagram of 2D perovskite/graphene photodetectors and (f) *I–V* curve of the 2D (C_4_H_9_NH_3_)_2_PbBr_4_/graphene heterostructure photodetectors in the dark and under various illumination intensities with a 470 nm laser irradiation. Reproduced from ref (1233). Copyright 2016 American Chemical Society. (g) Schematic MoO_3_-doped 2D perovskite nanosheet photodetector and (h) photogenerated current mapping in source–drain channel and schematic band diagram under *V*_d_ = +1 V under irradiation. Reproduced with permission from ref (1234). Copyright 2018 John Wiley & Sons, Inc. (i) Schematic diagram carrier dynamics in the single-crystalline (101)-oriented layered perovskite photodetector and (j) photoresponse of 1D-layered perovskites array with *n*= 2 and 4. Reproduced with permission from ref (1235). Copyright 2018 The Authors. (k) Schematic of CH_3_NH_3_PbI_3_/WSe_2_ heterojunction field transistor and (l) The *V*_g_–*V*_OC_ curve extracted from source and drain channel in CH_3_NH_3_PbI_3_/WSe_2_ heterojunction at 77 K. Reproduced from ref (1236). Copyright 2015 American Chemical Society.

Up to now, there have only been a few reports of MAPbI_3_-based photodetectors due to the limited stability of MAPbI_3_.^478^ However, 1D solid hybrid organic–inorganic perovskite NCs remain attractive as efficient carrier transport channels in photodetectors. Figure 134a,b presents the perovskite photodetectors based on solution-processed 1D MAPbI_3_ NWs with a responsivity of 5 mA W^–1^ and a response time of ∼0.3 ms.^1228^ However, the defects and grain boundaries in MAPbI_3_ NWs lead to scattering effects which significantly reduces the responsivity. The defect density in MAPbI_3_ NWs was reduced by surface passivation through OA soaking treatment.^1229^ As a result, larger responsivities (4.95 A W^–1^) and a shorter response times (< 0.5 ms) were achieved. To further enhance the photodetector performance, Deng *et al.* developed a blade solution-casting method to increase the crystallinity of MAPbI_3_ NWs.^1230^ As the blade moves against the MAPbI_3_ solution on the substrate, MAPbI_3_ precipitates out at the triple-phase (solid–liquid–solvent vapor) interface upon solvent evaporation and continues to self-organize to form 1D NWs along the direction in which the blade moves. The as-fabricated MAPbI_3_ NW photodetector possesses a high responsivity over 13 A W^–1^ due to the high perovskite crystal quality. Therefore, well-controlled gas–liquid–solid triple-phase contact within prepatterned substrates could be a key factor to produce large-scale high-quality NW crystals and practical perovskite NW photodetectors. Feng *et al.* developed a template-assisted method for the production of well-aligned single-crystal CsPbBr_3_ NW arrays, which enabled a surprisingly high responsivity of ∼1400 A W^–1^.^1231^ Dai *et al*. introduced an oxygen-related hole trapping state on the surface of the NCs, causing surface band bending, which results in an internal electric field that can spatially separate the photogenerated electron–hole pair, thereby suppressing the carrier recombination, as shown in Figure 134c,d. Additionally, polarized light detection can be achieved in the photodetectors based on the strict alignment of CsPbBr_3_ NW arrays along the [100] orientation.^1231^ All these pioneering works clearly demonstrate the potential of perovskite NCs in the fabrication of efficient photodetectors.

High-quality 2D perovskite NCs have been considered to be effective photoactive media for high-performance photodetectors due to their large surface area to volume ratio and potential integration with other 2D materials and conventional silicon circuits.^1237^ Essentially, there are two major working principles for photodetectors based on 2D perovskite NCs, *i.e.*, photoconductive and photovoltaic effects. A typical structure for perovskite photoconductors involves the perovskite sandwiched between two gold electrodes. 2D perovskite photoconductors typically deliver a responsivity of 22 A W^–1^ under visible laser illumination, which is superior to those photodetectors based on 3D perovskite films.^1238^ The integration of 2D perovskites with other 2D conductive materials can be an efficient approach to improve photodetector performance. In particular, heterostructure photodetectors consisting of 2D perovskite (C_4_H_9_NH_3_)_2_PbBr_4_ and interdigitated graphene electrodes were demonstrated, as shown in Figure 134e,f, in which graphene would be favorable for transporting photocarriers and improving stability in air.^1233^ This device gives a high responsivity of 2100 A W^–1^.^1233^ For devices operating based on the photovoltaic effect, one or more junctions are normally required. In this regard, Ou *et al.* fabricated a lateral junction by partially doping the n-type pristine perovskite nanosheet.^1234^ A large depletion region with a few micrometers width formed in which a lateral built-in electric field facilitates the separation and transport of photogenerated carriers. As a result, these photodetectors have a responsivity of ∼1.42 A W^–1^ and an EQE of ∼3.93% at zero bias, much higher than those of the pristine 2D perovskite device. A single-crystalline 2D Ruddlesden–Popper perovskite nanowire with a pure (101) crystallographic orientation has been used to fabricate ultrasensitive photodetectors, as shown by Figure 134i.^1235^ The organic layers act as insulating barriers which significantly reduce the dark current, whereas exposed crystalline perovskite layers function as charge conductive pathway for exciton dissociation, free-carrier conduction and charge injection, therefore giving an averaged responsivity of over 10^4^ A W^–1^ and a detectivity of over 7 × 10^15^ Jones. Apart from using dopants, the combination of 2D perovskites with other 2D semiconductors could also create a built-in electric field to form a p–i–n junction.^1236,1239^ A graphene/WSe_2_/2D MAPbI_3_/graphene device was assembled to work as a photodetector with ultrahigh on/off photocurrent ratios (>10^6^) under negative bias.

**Figure 135:**
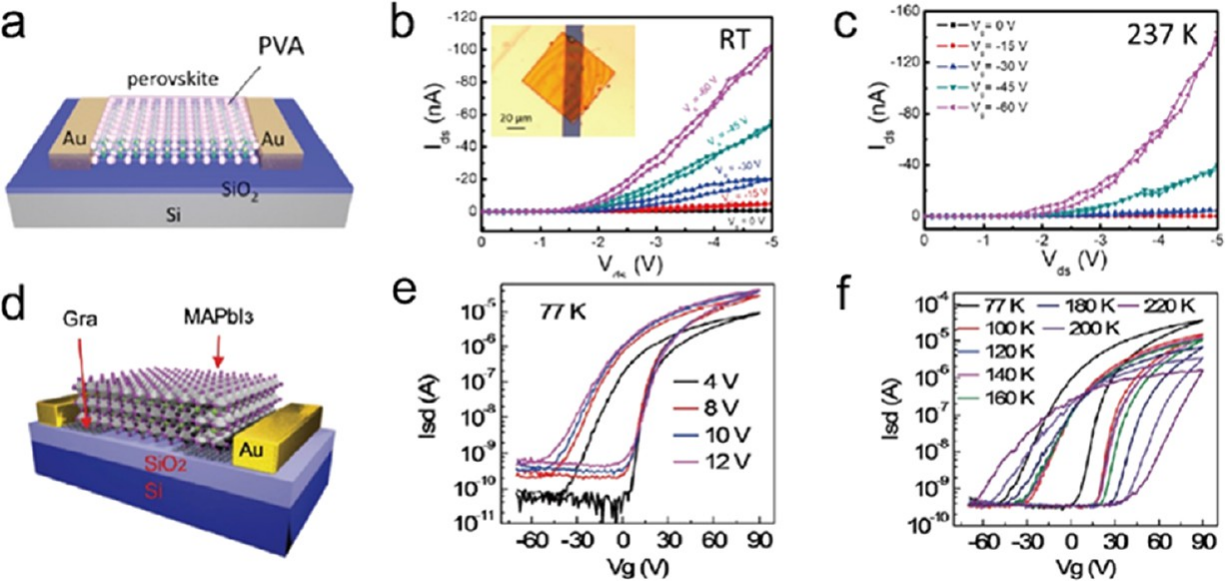
Perovskite NC-based FETs. (a) Schematic illustration of CsPbBr_3_-based FETs fabricated using the dry transfer method. Output characteristics of the as-fabricated CsPbBr_3_ FETs under gate voltages in the range from −60 to 0 V at (b) room temperature and (c) 237 K. Reproduced from ref (1245). Copyright 2017 American Chemical Society. (d) Schematic illustration of the graphene-contact MAPbI_3_ microplate-based FETs. (d) Transfer characteristics of the as-fabricated MAPbI_3_ FETs under different gate voltages from 4 to 12 V at 77 K. (e) Transfer characteristics of the as-fabricated MAPbI_3_ FETs under source–drain bias of 10 V at different temperatures from 77 to 220 K. Reproduced with permission from ref (1249). Copyright 2016 John Wiley & Sons, Inc.

Beyond photodetectors, the distinctive gate-modulated features due to the ambipolar nature of 2D perovskites under different biases underpin their great promise for transistors. The reported mobilities of hybrid perovskite film-based transistors are mostly below 1 cm^2^ V^–1^ s^–1^, which are much lower than their high intrinsic mobility ∼200 cm^2^ V^–1^ s^–1^ due to unavoidable ion migration at room temperature.^1240−1244^ In this regard, these results would suggest that perovskite NCs with lower ion vacancy and grain boundary density are promising for achieving improved performance. As shown in Figure 135a–c, Huo *et al*. developed high-quality ultrathin boundary-free CsPbBr_3_ platelets using van der Waals epitaxy and dry transfer processes, yielding FET hole mobilities of 0.32 and 1.04 cm^2^ V^–1^ s^–1^ at room temperature and 273 K, respectively.^1245^ Yu *et al.*(1246) further enhanced surface adhesion between thin single-crystal MAPbX_3_ and prepatterned FET substrates to reduce surface contamination, reaching record electron and hole mobilities of 1.5 and 4.7 cm^2^ V^–1^ s^–1^ at room temperature, respectively. Moreover, Cheng *et al.*(1236) systematically investigated transport properties of the high-quality perovskite materials with van der Waals contacts such as graphene and gold.^1236,1247−1249^ As shown in Figure 135d,e, Li *et al.* demonstrated temperature-dependent transfer characteristics of graphene-contact MAPbI_3_ microplate-based FETs with estimated electron mobilities of 4 cm^2^ V^–1^ s^–1^ at 77 K.^1249^ However, by achieving atomically flat contacts, the as-fabricated CsPbBr_3_ FETs showed Hall mobilities >2000 cm^2^ V^–1^ s^–1^ at 80 K and ultralow bimolecular recombination coefficients of 3.5 × 10^–^^15^ cm^3^ s^–1^.^1247^ Improving contacts with electrode and dielectric layers in FETs would be effective strategies to increase the performance of the perovskite NC-based FETs. However, exploration of perovskite NCs with lower ion vacancy densities will be essential for achieving practical FETs.^1250^

**Figure 136:**
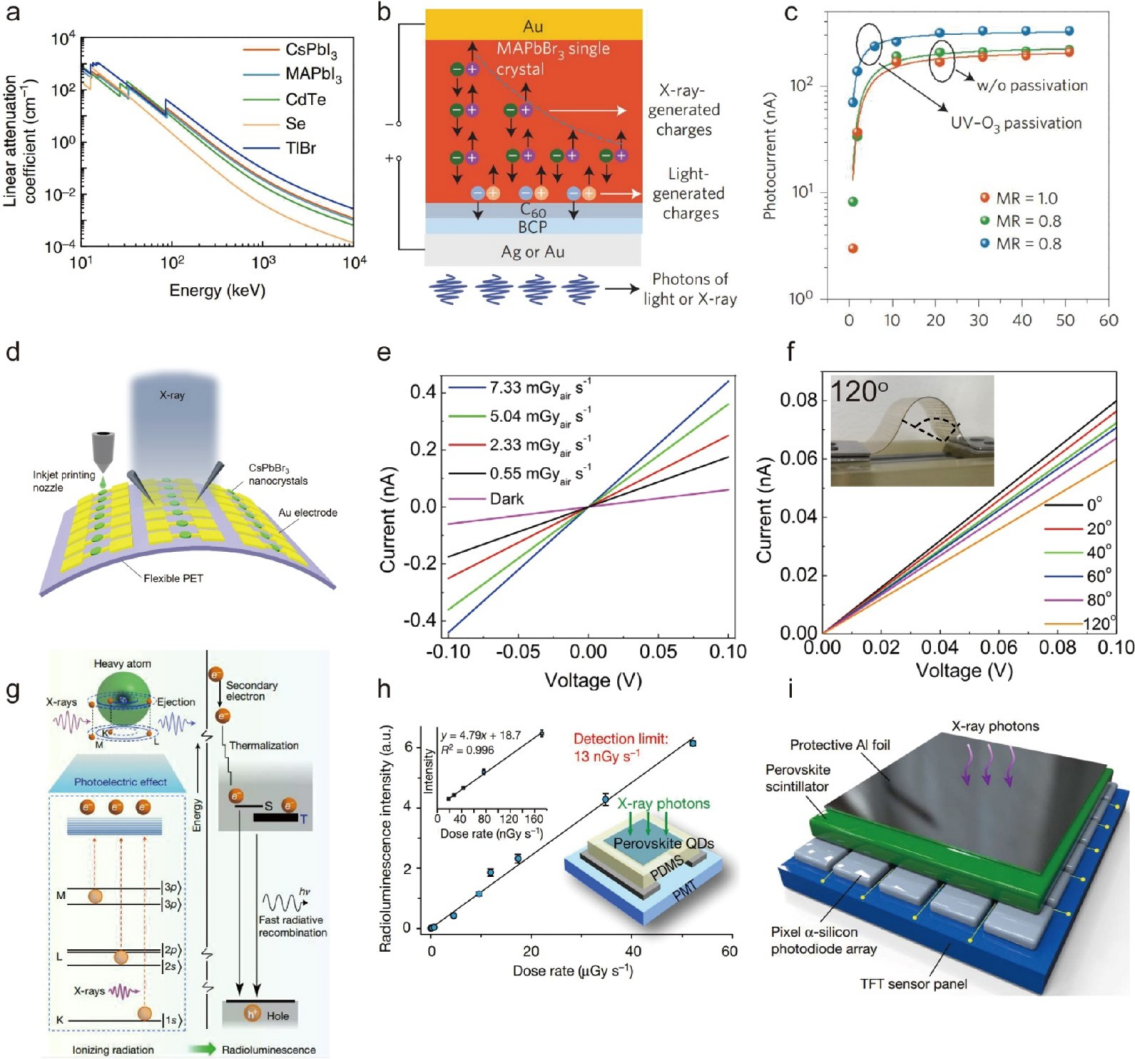
High-energy ionizing radiation detectors based on perovskite NCs. (a) Linear attenuation coefficient of MAPbI_3_, MAPbBr_3_, CdTe, Se, and TlBr in the 10–10000 keV energy range. Reprinted with permission under a Creative Commons CC BY license from ref (1255). Copyright 2019 The Authors. (b) Schematic configuration of the cross-sectional view of single-crystal X-ray detector. (c) Photocurrent of MAPbBr_3_ single-crystal devices with different molar ratios and surface passivation procedure *versus* electrical bias. Reproduced with permission from ref (1257). Copyright 2016 Nature Publishing Group. (d) Schematic diagram of the flexible X-ray detector arrays based on inkjet-printed CsPbBr_3_ NCs on PET substrate. (e) Dark current and photocurrent of the CsPbBr_3_ NCs X-ray detectors under different X-ray dose rates with 0.1 V bias voltage. (f) *I*–*V* curves of the CsPbBr_3_ NCs X-ray detectors at various bending angles with the X-ray irradiation of 7.33 mGy_air_ s^–1^ and 0.1 V bias voltage. Reproduced with permission from ref (1258). Copyright 2019 John Wiley & Sons, Inc. (g) Hypothesis of working principle of a CsPbBr_3_ NC-based X-ray scintillation. In general, photoelectric ionization, thermalization, and fast radiative recombination take place upon X-ray illumination in lead-halide perovskite NCs. (h) Radioluminescence intensity of a CsPbBr_3_-based scintillator *versus* dose rate. The inset at the top left presents radioluminescence profiles in the low dose rate range. (i) Schematic illustration of a prototype CsPbBr_3_ NCs-based flat-panel X-ray imaging system. Reproduced with permission from ref (1259). Copyright 2018 Springer Nature Limited.

Beyond visible photodetectors and FETs, metal-halide perovskites are also promising candidates for the detection of high-energy ionizing radiation, such as X-rays and γ-rays. Radiation detectors with high sensitivities and small lowest detectable dose rates can potentially be achieved with low cost due to the solution processability of the metal-halide perovskites and their high-*Z* elements.^1251−1253^ For X-ray detectors, the ability to control charge carrier movement is key to their functionality. Charge generation, transport and separation all must occur in the perovskite NCs sequentially upon X-ray irradiation.^1254,1255^ In particular, favorable optoelectronic properties, such as strong absorption, tunable band gap, long carrier diffusion length and large bulk resistivity in lead-halide perovskite NCs also contribute to improved sensitivity.^1256^Figure 136a shows the linear X-ray attenuation coefficient of different materials, suggesting that the perovskite materials are superior over current commercial materials for multiple solid-state applications.^1255^

In general, X-ray detectors could be classified as semiconductor-based direct and scintillator-based indirect devices. Solution-processed MAPbI_3_ films were initially used for X-ray detection by directly recording photogenerated current in both photovoltaic and photoconductive devices.^1260^ Owing to the heavy Z elements (Pb and I), high X-ray sensitivity and (∼25 μC mGy_air_^–1^ cm^–3^) and responsivity (1.9 × 10^4^ carriers/photon) were demonstrated, which is superior to amorphous a-Se-based X-ray detectors. Similar to visible photodetectors, the performance of X-ray detectors could be dramatically improved by interfacial engineering.^1261^ As shown in Figure 136b and c, using surface defect passivation processes, Wei *et al.* developed a hard X-ray detector using high-quality single-crystal MAPbBr_3_, which would enhance charge extraction efficiency and therefore yield a high sensitivity (∼80 μC Gy_air_^–1^ cm^–2^) and a lowest detectable dose rate (∼0.5 μC mGy_air_ s^–1^ ) at near zero bias.^1257^ The as-fabricated MAPbBr_3_ X-ray detectors provide not only a four times higher X-ray sensitivity but also ∼100-fold reduction in the lowest detectable dose rate than a-Se-based X-ray detectors.^1257^ Moreover, the record-high X-ray sensitivity could be further promoted up to ∼50000 μC Gy_air_^–1^ cm^–2^ in thick hot-pressed CsPbBr_3_ quasi-particle film with same crystal orientation and thickness of several hundreds of micrometers.^1253^ Alternatively, interface engineering would be suggested as an effective way to minimize the dark current upon X-ray irradiation. Kim *et al.* demonstrated a spin-cast MAPbI_3_-based X-ray detector comprising polyimide (PI)-MAPbI_3_ layer as the hole-transporting pathway and PI-MAPbBr_3_ as hole-blocking pathway, producing broad X-ray absorption range and a large sensitivity over 10 μC mGy_air_^–1^ cm^–2^.^1262^ Strategically, low-cost patterning perovskite NCs on flat or flexible substrates is of great importance for scale production of printable and flexible perovskite-based X-ray detectors. As shown in Figure 136b. Liu *et al.*(1258) demonstrated flexible soft X-ray detectors array based on CsPbBr_3_ NCs film using inkjet printing. Apart from a reasonably high sensitivity at low X-ray dose rate (∼17.2 μC mGy_air_ s^–1^; see Figure 136e), the as-fabricated perovskite flexible devices only lose 25% electrical signal at bending angle over 120° (see Figure 136f) and sacrifice only 12% current after 200 bending circles.

Perovskite NC scintillators have also emerged as commercially competitive indirect converters for nondestructive X-ray detectors.^1252,1263^ Chen *et al.* demonstrated fully inorganic perovskite NC-based scintillators for X-ray imaging.^1259^ Due to highly emissive triplet excited states, fast radiative recombination and high quantum efficiency from CsPbBr_3_ NCs, the as-fabricated scintillators have a rapid response time of ∼46 ns and a low X-ray detection limit of 13 nGy s^–1^ (∼400 times lower than typical X-ray diagnostics), as indicated in Figure 136g,h.^1259^ The as-fabricated prototype CsPbBr_3_ NCs-based flat-panel X-ray imaging system is desired for dynamic real-time X-ray imaging when exposed to a low X-ray dose of 15 μGy, as shown by Figure 136i. In addition, very recent reports indicated that embedding emissive CsPbBr_3_ NCs in host matrices such as Cs_4_PbBr_6_ and plastic waveguides is a very effective approach to produce stable and low optical loss scintillators for X-ray detectors.^494,1264^ Moreover, lead-free perovskites have also been used in the fabrication of X-ray detectors.^1265,1353^ For example, (C_8_H_17_NH_3_)_2_SnBr_4_ 2D-layered perovskites with absolute near-unity PLQY and a large Stokes shift have been applied in scintillators for green X-ray imaging applications.^1265^ In another work, Zhu *et al.*(1266) have demonstrated the scintillators based Cs_2_Ag_0.6_Na_0.4_In_0.85_Bi_0.15_Cl_6_ (PL lifetime = 1.3 μs) with enhanced light yield of 39000 ± 7000 photons/MeV compared to that of perovskite colloidal CsPbBr_3_ materials; however, the lead-free perovskite materials, in general, suffer from long decay time, which are required further material optimization. More importantly, most reported lead-free-based X-ray detectors are based on bulk single crystals or 2D-layered perovskites, whereas the corresponding NC-based devices are yet to be realized. In summary, the field of perovskite-based visible light and X-ray detectors is a very fast-moving research area toward the realization of various applications including integrated optoelectronic devices, sensing, and medical radiography. Among all, the scintillator-based indirect strategy is more promising in low-cost X-ray imaging system by combing current CMOS system and facile preparation methods.

#### Summary and Outlook on Perovskite Photodetectors

Owing to their strong attenuation of visible and high-energy photons, high photoluminescence quantum yields and ambipolar charge transport, lead-halide perovskites have been demonstrated as promising photodetectors, FETs and X-ray/γ-ray detectors. Among these applications, it has been shown that nanostructuring has delivered benefits in terms of performance or compatibility with flexible substrates. In photodetectors, perovskite NCs have demonstrated improved performance over 3D perovskite thin films through surface passivation to reduce nonradiative recombination. On/off ratios exceeding 10^5^ have been achieved in photodetectors based on CsPbX_3_ NCs. Blending with conducting graphene or CNTs led to high responsivities of 10^8^ A W^–1^ and fast response times of 0.016 ms by improving carrier extraction. Furthermore, by synthesizing CsPbBr_3_ NWs that are well-aligned, responsivities as high as ∼1400 A W^–1^ have been achieved, as well as polarized light detection. Future improvements in performance will depend on careful control over the interfaces between the perovskite and contacts, as well as control over the distribution of organic ligands, which could reduce dark current but could also increase response times if placed inappropriately such that they reduce charge extraction.

The ambipolar nature of charge transport in lead-halide perovskites has been taken advantage of in FET applications. A key challenge is ion migration in perovskites, which modules the field-effect mobility to well below the intrinsic mobility. Grain boundary density, as well as interfaces with contacts play an important role. Future work should focus on improved contact and dielectric layers, as well as synthesis routes to reduce the density of vacancies to reduce ion migration.

Finally, the high average atomic number in lead-halide perovskites allows them to strongly attenuate X-ray and γ-rays, and an improved performance over industry-standard amorphous selenium has been demonstrated. Although full attenuation requires the use of thick single crystals, NC-based perovskites have been shown to demonstrate reasonable performance as solid-state X-ray detectors but with the added advantage of being solution processable on flexible substrates. CsPbBr_3_ NCs have also been shown to be effective X-ray scintillators, owing to the high quantum efficiency, fast radiative recombination, and highly emissive triplet excited states.

### Perovskite Nanocrystal Solar Cells

#### Lead-Halide Perovskite NCs

Lead-halide perovskites have brought about a revolution in thin film photovoltaics. In a similar manner, lead-halide perovskite NCs have very recently also brought about a revolution in QD solar cells.^90,115,185,318,523,1267−1271^ Perovskite NCs can utilize surface energy for improving phase stability, have different, but also low cost solution-based fabrication processes, and enable a platform to better understand and engineer the properties of MHPs, such as through molecular surface/grain passivation, achieving higher PLQY, formation of perovskite heterojunctions, *etc.*(90) Quantum confinement effects, while perhaps less pronounced than in the Pb chalcogenides, are still prevalent in Pb-halide perovskites, which have Bohr radii similar to those of Cd chalcogenides. Thus, perovskite NCs with relatively large diameters (>10 nm) are best characterized as in the intermediate confinement regime.^1272,1273^

An interesting aspect of halide perovskite NCs is the role the surface energy plays in the stabilization of certain crystalline phases that are not stable in their bulk counterparts at room temperature. Perhaps a reason why researchers have broadly overlooked halide perovskites as a semiconductor system for the past 80 years is the limited number of A-site cations needed stabilize Pb-halides as a perovskite. Cs^+^ is typically too small to promote CsPbI_3_ into the octahedral corner-sharing perovskite phase, and thus a slightly larger but uncommon organic cation, such as methylammonium, is required to achieve the tolerance factor needed to accomplish the perovskite structure. For the interest of single junction solar cells, a band gap as close as possible to 1.3 eV is preferred in order to maximize the potential efficiency as predicted by the Shockley–Queisser analysis.^1274^ Thus, CsPbI_3_, MAPbI_3_, and FAPbI_3_,^1275^ are the most common conventional perovskite structures of which CsPbI_3_ and FAPbI_3_ are especially interesting. The former by the inorganic nature with higher temperature stability and the later also presenting higher stability than MAPbI_3_, and the narrowest band gap of 3D iodine perovskites. As stated above, pure composition CsPbI_3_ and FAPbI_3_ suffer from cations either too small or too large to preserve the stability of the photoactive perovskite black phase, converting into the less photoactive yellow phase at room temperature in bulk materials.^185,1275,1276^ However, by reducing the perovskite size to <20 nm, the contribution of the surface energy (namely tensile strain) can influence the stability of the phase, promoting the formation of the black perovskite phase of CsPbI_3_ and FAPbI_3_.^185,1277,1278^ Ironically, at the nanoscale, MAPbI_3_ (with the most ideal A-site cation radius for bulk compounds) has the lowest stability.^1279^ There are phase-related nuances of perovskite NCs where the transitions among the α, γ, and δ perovskite phase can be size, composition, and temperature-dependent.^1273,1280^

**Figure 137:**
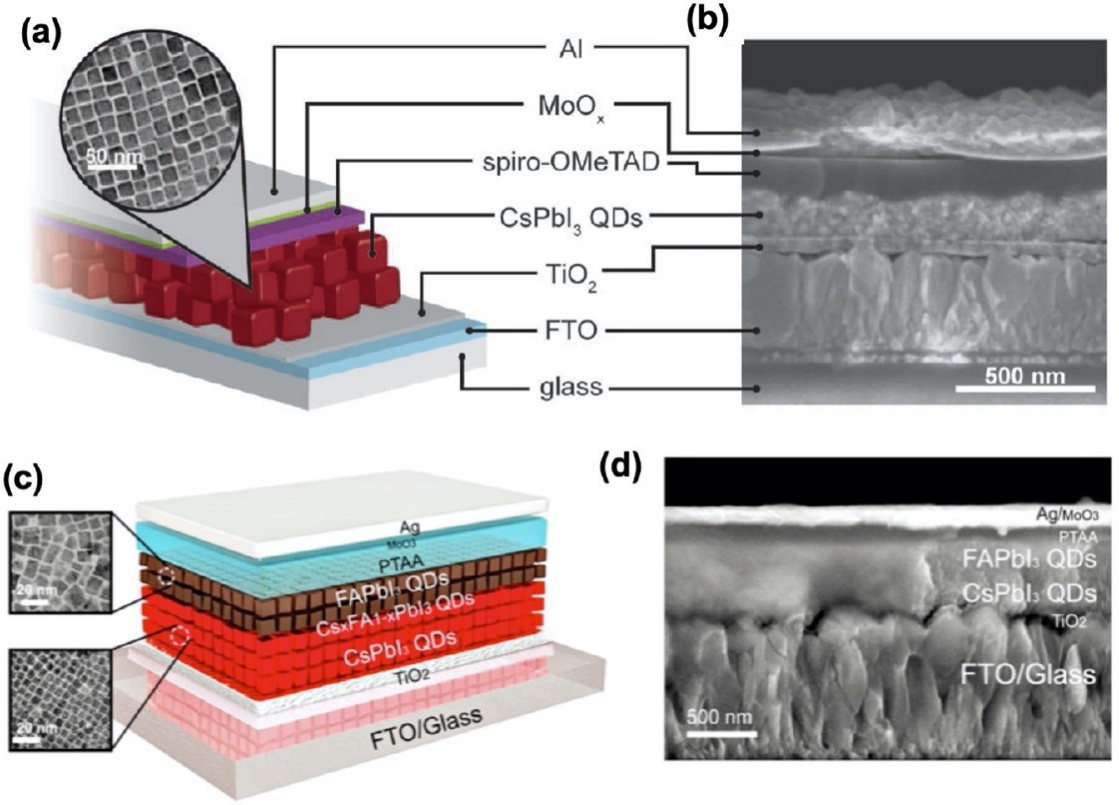
(a) Schematic of a perovskite QD solar cell with halide perovskite NCs as the light absorber and (b) corresponding SEM image of an exposed cross section. Reproduced with permission from ref (185). Copyright 2016 American Association for the Advancement of Science. (c,d) Schematic of a perovskite QD solar cell employing two compositions which have been shown to form a charge separating heterostructures (c) and the corresponding cross-sectional SEM image (d). Reproduced from ref (1283). Copyright 2019 American Chemical Society.

Beyond phase stabilization of the building blocks needed for perovskite QD solar cells, the next challenge is preparing QD films thick enough to absorb incident light, while simultaneously having sufficient transport properties to harvest the photogenerated charges. Low polarity solvents such as methyl acetate (MeOAc) or ethyl acetate (EtOAc)^185,1281^ preserve the stability while removing or replacing surface ligands^1282^ and have permitted the early report on perovskite NC solar cells which showed PCEs exceeding 10%.^185^ Here, a layer of few hundred nm of CsPbI_3_ NCs were sandwiched between TiO_2_ and spiro-OMeTAD, which act as electron and hole selective contacts respectively, see Figure 137a,b.^1283^ It was found that the CsPbI_3_ NC-based solar cell devices showed improved operational stability as well as tolerance to higher relative humidity levels.

The high crystallinity of colloidally grown perovskite NCs reduces nonradiative recombination channels, reflected by an enhancement in the PLQY, especially if the surface states of NCs are properly passivated.^1284,1285^ This property is especially attractive for the development of photocatalytic systems,^43,1286^ optoelectronic devices^1089^ and also for photovoltaic applications.^523^ The increase in PLQY to values higher than 80%, in conventional NCs, has been a process that has required a couple of decades of research.^1285^ In contrast, immediately following the report of perovskite NCs^66^ were reports with PLQY beyond 80%^14,25^ and soon after reports of NCs with PLQYs near-unity.^520^ Low nonradiative recombination is necessary for photovoltaic devices with high open circuit voltage, *V*_oc_.^1287^ Because of this, halide perovskite QD solar cells presents outstanding *V*_oc_, with several reports showing values greater than 1.25 V with up to 90% of the thermodynamically limited *V*_oc_ demonstrated.^192,1288−1290^

While low nonradiative recombination ensures a high *V*_oc_, achieving high efficiencies also require good transport properties of the photogenerated charges, along with an absorber layer thick enough to harvest all available incident light. A critical component fundamental for eliminating nonradiative recombination in colloidal NCs in general and of perovskite NCs in particular is the passivation of the NC surfaces with organic capping ligands. However, these organic ligands hinder charge transport. Therefore, a balance is required for proper passivation, such that the spacing between NCs is short, such that electron hopping can still occur. In Pb chalcogenide QD solar cells, transport properties are actively studied using a wide variety of ligand exchange strategies with many ligand head group options.^1284,1291,1292^ Perovskite NCs often have multiple ligand types (cationic and anionic species) which may be handled individually.^1281,1282^ Nevertheless, it is anticipated that with more work on designing better ligand motifs for halide NCs, as demonstrated for other NCs systems, perovskite QD solar cell performance may greatly increase.

**Figure 138:**
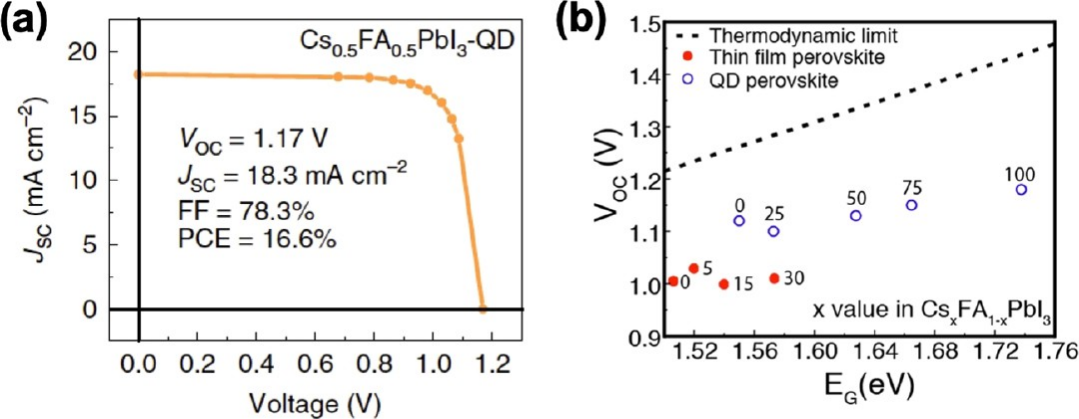
(a) Current–voltage characteristics of currently published world record QD solar cell; the device is based in Cs_*x*_FA_1–*x*_PbI_3_ NCs. Reproduced with permission from ref (1269). Copyright 2020 The Authors under exclusive licence to Springer Nature Limited. (b) Comparison between *V*_oc_ obtained for perovskite QD solar cells, standard thin film solar cell, and the maximum thermodynamic limit. Reproduced from ref (1295). Copyright 2018 American Chemical Society.

FAPbI_3_ is *a priori* more appealing for photovoltaic applications than CsPbI_3_ due to a narrower band gap.^1293^ However, due to transport limitations, the performance of perovskite QD solar cells based on FAPbI_3_ NCs has not exceeded the efficiency of CsPbI_3_ NCs. Nevertheless, the combination of CsPbI_3_ with FAPbI_3_ and/or Cs_*x*_FA_1–*x*_PbI_3_ NCs in charge separating heterostructures (see Figure 137c,d) has enabled the PCE of perovskite QD solar cells to exceed 15%.^1283,1294^ Cells based on mixed cation NCs has also shown better performance than analogous devices but based on single cation NCs.^192,1269^ Cs_*x*_FA_1–*x*_Pb(I_1–*x*_Br_*x*_)_3_-based perovskite QD solar cells with band gaps larger than 1.8 eV exhibit *V*_oc_ values nearly 100 mV higher than those of the solar cells based on CsPb(I_1–*x*_Br_*x*_)_3_ NCs.^192^ The currently published PCE record of QD solar cells of 16.6% was obtained with devices using Cs_*x*_FA_1–*x*_PbI_3_ NCs as a light-harvesting material (see Figure 138a).^1269^ In this achievement, the synthesis of the NCs with excess oleic acid ligand is reported to play a key role. In addition, it was demonstrated that the Cs_*x*_FA_1–*x*_PbI_3_ NC-based solar cell devices exhibit significantly enhanced photostability compared with their thin-film counterparts, and they retain 94% of the original PCE under continuous solar illumination for 600 h.

Halide perovskite NCs may also be attractive for the development of multijunction solar cells as the wide gap component. However, there has not been a compelling demonstration published yet. First, the NCs offer band gap control by quantum-confinement effects in addition to composition. The versatility of halide perovskites allows the band gap to be easily tuned through the halide composition.^765,1296^ In bulk thin films, halide phase segregation is readily observed in mixed-halide perovskites under illumination^1170,1297,1298^ or when electrical bias is applied,^1299^ limiting band gap stability in mixed perovskites. However, due to size constraints, phase segregation is suppressed in mixed-halide perovskites NCs in comparison with thin films.^1172,1297,1300^ This may lead to more possibilities for achieving higher voltages in devices with band gap in the 1.8–2.0 eV range. Presently, perovskite QD solar cells often exhibit higher *V*_oc_ than bulk perovskites of similar band gap and composition, in the range of 1.55–177 eV (see Figure 138b).^1295^ However, there are several key limitations of perovskite QD solar cells at this stage. One area is the development of greater versatility in terms of carrier selective contacts, such as being able to construct the cell in a p–i–n geometry instead of an n–i–p structure, or using contacts with lower thermal budgets for processing on other subcells. Another challenge is that increasing the band gap by quantum confinement or by introducing bromine has yet to produce a high efficiency solar cell with larger *V*_oc_ due to reductions in the lifetime. Likely a breakthrough in ligand exchange for improved passivation or more complex compositions that yield longer lifetimes and higher band gaps could be the key to realizing this potential in multijunction cells using high voltage perovskite NCs.

The fact that perovskite QD solar cells have now demonstrated >16% PCE is exciting in its own right; however, just having this distinct solar cell platform can enable us to learn more about metal-halide perovskites, in general. The high surface area to volume ratio enables studies of surface passivation, which could carry over to other areas in halide perovskite science. NCs can act as seeds for the nucleation of larger crystals. At this moment, it is not clearly known if both kinds of devices fully share the same working principles or if there are significant differences in effective carrier concentrations, junction characteristics, *etc*. Recent studies point to similar optoelectronic behavior as the impedance spectroscopy analysis highlights.^1301^ Furthermore, several groups have demonstrated improved characteristics in devices using heterojunctions containing a thin film layer and a QD layer.^1279,1302,1303^ For these reasons, perovskite QD solar cells offer us many more possibilities with high potential.

#### Lead-Free Perovskite NCs

As discussed in the NANOCRYSTALS OF LEAD-FREE PEROVSKITE-INSPIRED MATERIALS section, there has been extensive work in developing lead-free analogues to LHP NCs. Beyond lighting applications, these materials have also been investigated for photovoltaics. For example, tin-halide perovskite solar cells have achieved high photocurrents as it has a low band gap, high absorption coefficient and a symmetric perovskite crystal structure with disperse bands.^1304,1305^ The highest efficiency currently reported of bulk Sn-based perovskite solar cell is reported by Jokar *et al.*(1306) using a mixed cation (guanidinium (GA^+^), formamidinium (FA^+^)), tin triiodide perovskite with ethylenediammonium diiodide (EDAI_2_) as an additive. The PCE of the device is 9.6% with a *J*_sc_ of 21.2 mA cm^–2^. Sn-based perovskite quantum dot solar cells have achieved comparable PCEs. For example, CH_3_NH_3_SnBr_3–*x*_I_*x*_ NC solar cells using mesoscopic TiO_2_ anode has a PCE of 8.79%, *V*_oc_ of 0.758 V, *J*_sc_ of 17.06 mA cm^–2^, and fill factor of 68.1%.^1307^ Tin-based perovskite QRs have also been synthesized and investigated for photovoltaics. Chen *et al*. reported a CsSnX_3_ QR solar cell with a PCE of 12.96% for CsSnI_3_. They also reported CsSnBr_3_ and CsSnCl_3_ QRs with 10.46% for and 9.66% efficiency, respectively.^517^ Similar work has also been reported by Chen *et al.* for CsGeX_3_ QRs with a peak PCE of 4.92%.^538^ The bottleneck for tin-halide perovskite NC solar cells are low open circuit voltages. The average *V*_OC_ of Sn-based perovskite solar cells is around 0.5 V, which is significantly below their band gap of 1.2-1.4 eV^1304,1308^ This is due to the facile and undesirable oxidation from Sn^2+^ to Sn^4+^, which leads to p-type doping and an increase in the dark current density and photocarrier recombination.^1308,1309^ The PCEs of Sn-based perovskite solar cells are currently well below their Shockley–Queisser limit of 33%.^1308^ Unlike lead-based perovskites, tin-based perovskites do not have inactive lone pair, which could provide oxidative resistivity. As a result, tin-based perovskites are extremely sensitive to oxidation induced self-doping, which leads to perovskite degradation. The future challenges include stabilizing the tin oxidation state to improve the defect-tolerant properties of Sn-based perovskite and solar cell performance. Apart from methods like partial substitution, addictive engineering and addition of deoxidizer,^1268^ developing low-dimensional structures, such as quantum dots, could be another approach to stabilize Sn-based perovskites, as NCs have less intrinsic defects caused by large surface to volume ratios and automatic elimination of volume defects.

Apart from isovalent substitution of lead, other lead-free perovskite NC solar cells have been investigated, including A_2_B(I)B(III)X_6_ double perovskite NCs, 0D A_3_B(III)_2_X_9_ and 0D A_2_B(IV)X_6_ perovskite-inspired materials.^95,352,1310^ Cho *et al.*(1311) recently reported a Cs_2_AgBiBr_6_ double perovskite NC solar cell using semiconductor oxides such as TiO_2_ or ZnO as the ETL. By depositing multiple layers (20 deposition cycles, 225 nm) of the QD film, the device achieved an open-circuit voltage of 0.92 V. Although this is similar to the *V*_OC_ of LHP solar cells, it is well below the ∼2.1 eV band gap of Cs_2_AgBiBr_6_. Furthermore, the efficiency was only 0.13%. The low PCE cannot be further improved by simply increasing the thickness of the absorber layer as the material can only absorb light with wavelength below 550 nm due to the wide band gap.^1312^ Also, the low fill factor (32%) indicated the QD films to have high series resistance.^352,1311^ Vacancy-ordered double perovskite A_2_B(IV)X_6_ is considered a 0D perovskite-inspired materials due to the absence of connectivity between BX_6_ octahedra.^352^ Many A_2_B(IV)X_6_ compounds have been investigated for potential photovoltaic applications, including MA_2_SnI_6_,^1313^ Cs_2_TiBr_6_^1314^ (champion efficiency of 3.3%) and Cs_2_PdBr_6_.^1310^ However, there are no reported quantum dots solar cell for these materials yet. Recently, Zhou *et al.* successfully synthesized Cs_2_PdBr_6_ NCs with single unit cell thickness and high stability.^1310^ The NCs demonstrated a measured photocurrent density of 1.2 μA cm^–2^ under an applied potential of 0.65 V_Ag/AgCl_ with simulated solar light (AM1.5G, 150 mW/cm^2^, compared to lead-free perovskite thin-film NCs). It would be interesting to see if the development of A_2_B(IV)X_6_ NC materials can further improve the performance of the solar cell, such as using 0D Cs_2_TiBr_6_, as low-dimension quantum dots have larger surface to volume ratio and less volume defects. In general, comparing with lead-halide perovskite quantum dots solar cell, the research of lead-free perovskite NC solar cell is still at the beginning stage. There is a strong incentive to synthesis high-quality NC materials and fabricating more efficient lead-free quantum dots for solar cells applications, even though the current PCE of the cell remains low. The motivation would be it has been shown that NCs can stabilize thermodynamically unstable phase.^1315^ For example, the bulk perovskite cubic α-CsPbI_3_ black phase is unstable under room temperature, and the phase becomes metastable in the form of NCs and can survive for days in solution.^185^

#### Conclusions and Outlook for MHP NC-Based Solar Cells

Currently, one of the main challenges for lead-free perovskite-inspired NCs is to achieve high efficiency and stability simultaneously. Sn-based NC solar cells have achieved the highest efficiencies among these materials, but the materials still suffer from instability issues. By contrast, stable materials such as bismuth-based (Bi) and antimony-based (Sb) double perovskites NCs, have low power conversion efficiency (less than 5%). The degradation mechanism has been discussed previously in the Light-Emitting Devices section. Although surface ligands are expected to stabilize the metastable phases of perovskites, the ligands are often removed either by washing or annealing for improving the charge transport across the films. Therefore, it is still unclear what density of ligands is required on the NC surface to promote phase stability. Currently, extensive studies have been made on improving the stability of solar cells made with perovskite bulk thin films.^1316−1318^ By following a similar logic, more works are required on quantum-dot-based solar cells, such as utilizing compositional engineering or using doped NCs. Furthermore, the candidates for solving the toxicity of lead-based perovskite should not be limited to perovskites materials; other perovskite-inspired materials such as chalcogenide NCs should also be explored, and these materials are detailed in reference.^1319^

### Photocatalysis Using Perovskite NCs

Chemical fuels have significantly higher energy storage capacity than the batteries due to the very high specific energy of the former, which can be released by combustion.^1320,1321^ Harvesting the energy from chemical fuels through the use of solar radiation can enable the clean and efficient storage or renewable solar energy.^1322^ The common chemical fuels generated are hydrogen and oxygen (from water splitting), or methane (from CO_2_ reduction).^1323,1324^ Photons in the UV and visible wavelength regions have sufficient energy to drive these photochemical reactions.^1325^ Owing to their large specific surface area, NCs offer the possibility to both absorb solar radiation and drive the desired solar fuel generating reaction without any external bias.^1326,1327^ The attractive optical properties of halide perovskite NCs (for example, high absorption coefficients in the UV–visible region, a tunable band gap, and high PLQY) make them suitable candidates for solar-driven photocatalytic applications. While recent progress in the halide perovskite NCs leads to successful use in different optoelectronic filed, there use in the field of photocatalysis remains a challenge due to their instability in aqueous media.^1328,1329^ Here, we will provide the current development of perovskite NCs toward photocatalytic dye degradation, H_2_ evolution as well as CO_2_ reduction. First, we will discuss the photocatalytic activity of the Pb-based and Pb-free perovskite NCs, followed by photocatalytic activity of halide perovskite-based heterostructures.

#### Photocatalysis with Pb-Based Perovskites

**Figure 139:**
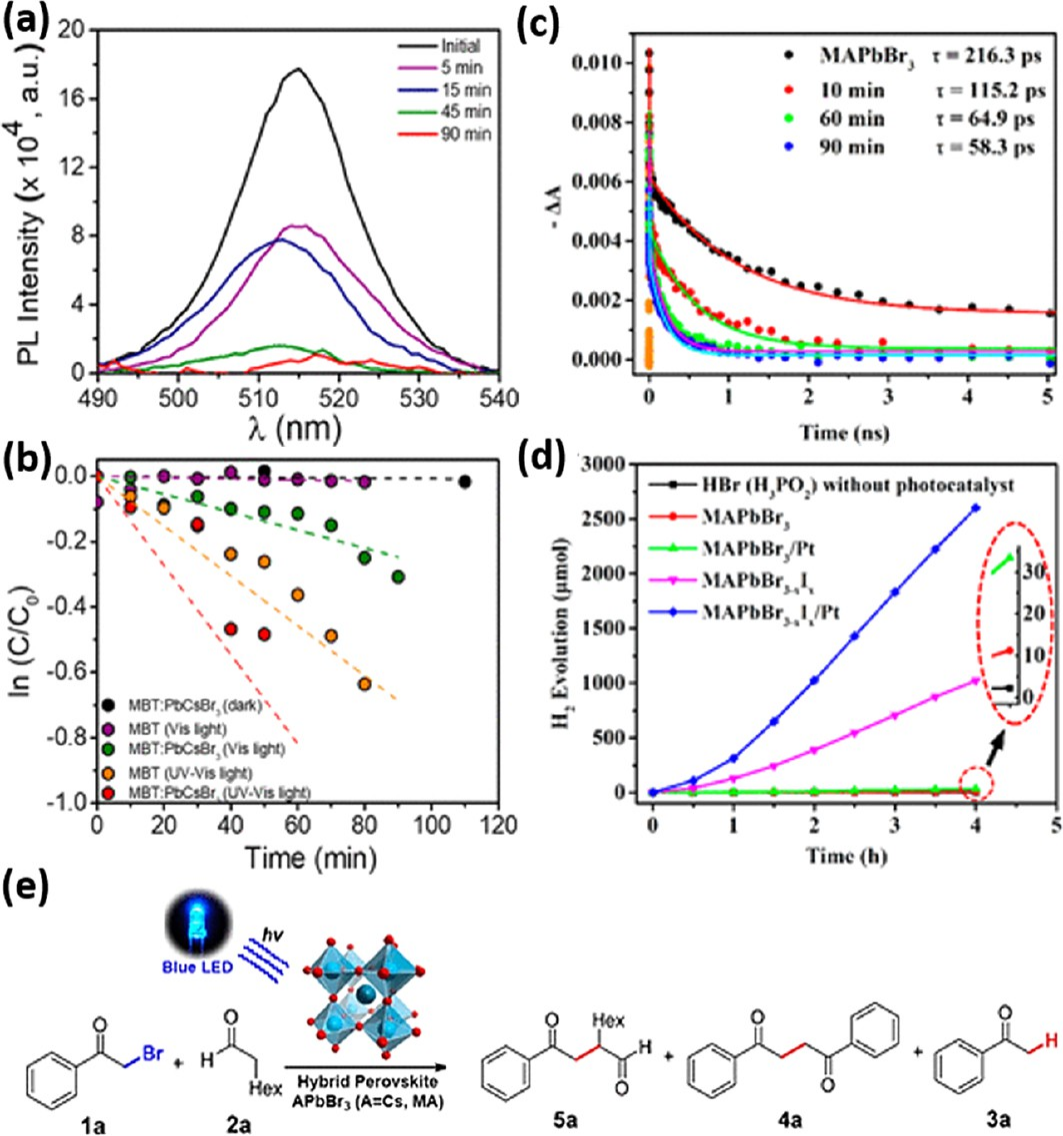
(a) PL spectra of CsPbBr_3_ NCs in the absence and presence of 2-mercaptobenzothiazole, under 100 mW cm^–2^ irradiation with UV filter. (b) Relative change in MBT concentration (without and with CsPbBr_3_) with time under visible and UV–visible light. (c) Bleach recovery kinetics of MAPbBr_3_ (0 min) and MAPbBr_3–*x*_I*_x_* at different time (10 to 90 min) of ion-exchange reaction as observed from transient absorption spectroscopy. (d) Photocatalytic activity of the H_2_ evolution without and with different photocatalysts (MAPbBr_3_, MAPbBr_3_/Pt, MAPbBr_3–*x*_I*_x_*, and MAPbBr_3–*x*_I*_x_*/Pt). (e) APbBr_3_ (A= Cs or MA) NCs for photocatalytic α-alkylation of aldehydes. Panels a and b are reproduced from ref (1286). Copyright 2019 American Chemical Society. Panels c and d are reproduced from ref (1330). Copyright 2018 American Chemical Society. Panel e is reproduced from ref (1331). Copyright 2019. American Chemical Society.

Most of the developed waste water treatment strategies primarily separate only organic contaminants from water. However, it is necessary to convert these contaminants to nontoxic substances. The outstanding optoelectronic properties of lead-halide perovskites, *e.g*., CsPbBr_3_ NCs can be used for the photocatalytic degradation of organic pollutants and convert them to nontoxic substances. The photocatalytic degradation of a common organic pollutant 2-mercaptobenzothiazole (MBT) in the presence of CsPbBr_3_ NCs has been investigated systematically.^1286^ MBT is a poorly biodegradable heterocyclic organic compound which causes severe toxicity in the aqueous solution. As has been shown in Figure 139a, the PL intensity of CsPbBr_3_ NCs reduces drastically in the presence of MBT. The energy level alignment between CsPbBr_3_ NCs and MBT suggests a photoinduced hole transfer from the perovskite NCs to MBT, which results in PL quenching. This leads to the oxidation of MBT in the presence of lead-halide perovskite NCs and results complete degradation of the pollutants. Time-resolved PL measurements further support the hole-transfer phenomenon.^1286^ To unambiguously determine the role of lead-halide perovskite NCs in MBT photodegradation, several control experiments were carried out and shown as relative concentration of the contaminant with time in Figure 139b. It is evident from the experiments that in the absence of the perovskite NCs, only UV light is effective for the degradation of MBT. However, in the presence of the CsPbBr_3_ NCs, significantly faster photodegradation of MBT takes place under both visible and UV irradiation. The photodegradation rate constant for MBT has been calculated from the linear plot of ln(*C*/*C*_0_) *versust*, assuming a pseudo-first-order reaction (Figure 139b). The calculated rate constant suggests, although in the presence of UV irradiation, the photodegradation rate of MBT doubled with CsPbBr_3_ NCs; however, in the presence of visible irradiation, the rate becomes 6-fold faster with CsPbBr_3_ NCs. The zero response toward photodegradation of MBT in the presence of CsPbBr_3_ NCs in the dark eliminates the possibility of any competing mechanism.

Employing a light-assisted halide exchange method in aqueous HBr/HI solution, mixed-halide MAPbBr_3–*x*_I_*x*_ perovskite has been synthesized from pristine MAPbBr_3_, which processes a band gap–funnel structure.^1330^ Such a structure results in an iodine concentration gradient within the perovskite, where the iodine concentration increases gradually from the core to the surface of the NC. This enhances the charge transport properties toward the surface, which is beneficial for photocatalytic reactions at the surface of the perovskites. The photogenerated electron–holes thus can migrate toward the surface through such band gap-funneled perovskite and can initiate the photocatalytic reaction. To understand the charge carrier dynamics induced by the halide-exchange reaction, ultrafast transient absorption spectroscopy has been performed. The TA spectrum of pristine MAPbBr_3_ shows a ground-state bleach at 526 nm due to photoinduced phase-space filling from electrons and holes.^1330^ However, the 90 min iodine exchange perovskite sample shows only a 10 nm red shift in the bleach signal, suggesting the TA spectrum is dominated by Br ions in the MAPbBr_3–*x*_I_*x*_. In other words, the bromide ions inside the particle are only partially replaced by the iodide ions which supports the band gap–funnel structure. However, comparing the ground-state bleach recovery kinetics of MAPbBr_3_ and MAPbBr_3–*x*_I_*x*_ at different time of the ion-exchange reaction (Figure 139c) reveals significantly faster bleach recovery signal for longer time iodide exchange perovskites. This indicates on increasing iodine content at the surface (at longer time ion-exchange reaction), charge transport toward surface increases significantly due to the band gap–funnel effect, which results in faster recovery of the bromide-rich photobleach signal. To corroborate the enhanced charge transport property toward better photocatalytic performance in the band gap-funnel MAPbBr_3–*x*_I_*x*_ perovskite, photocatalytic H_2_ evolution reaction has been performed under visible-light irradiation. The pristine MAPbBr_3_ shows poor photocatalytic H_2_ evolution performance (2.8 mmol/h) which improves to 8.4 mmol/h after loading on Pt (Figure 139d). Surprisingly, after introducing the band gap-funneled MAPbBr_3–*x*_I_*x*_ perovskite, the activity increases significantly to 255.3 mmol/h. Expectedly, on loading with Pt, a further 2.5-fold (651.2 mmol/h) enhancement was observed as a result of efficient separation of the photogenerated electron–hole.

Zhu *et al.* demonstrated the C–C bond coupling organic reactions using APbBr_3_ (A = Cs or MA) as photocatalysts.^1331^ As shown in Figure 139e, under visible-light (λ = 450 nm) irradiation, APbBr_3_ NCs can selectively produce several products, including dehalogenated acetophenone **3a** (yield 76%), sp^3^ C coupling product **4a** (8%), and α-alkylation product **5a** (7%). In addition, the broad reaction scope of this important organic transformation, especially the tolerance of sophisticated biorelevant functional groups, indicates the feasibility of employing halide perovskites for photo-driven pharmaceutical molecule synthesis. In another work,^1332^ they further demonstrated the halide perovskites NCs can catalyze a series of organic reactions, such as C–C bond formations *via* C–H activation, C–N bond formations *via* N-heterocyclizations and C–O bond formations *via* aryl esterifications. In this work, the impacts of reaction conditions (*e.g*., the size of NCs, solvent types, acid/base, and air tolerance, *etc.*) on the performance of CsPbX_3_ (X = Cl, Br, I) NCs were systematically investigated, which provide important guidance for expanding the application of halide perovskite-driven organic reactions. Another interesting example of the use of perovskites in photocatalysis is that of CsPbBr_3_ nanoparticles as photosensitizers for a demanding photoredox catalytic homo- and cross-coupling of alkyl bromides at room temperature by merely using visible light and an electron donor, as demonstrated by Pérez-Prieto and co-workers.^348^ The building of a high concentration of the generated radical anions in the NC surface eventually facilitated the exergonic C–C bond formation, thus demonstrating the cooperative action between the nanoparticle surface and the organic capping.

#### Photocatalysis Using Pb-Free Perovskites

**Figure 140:**
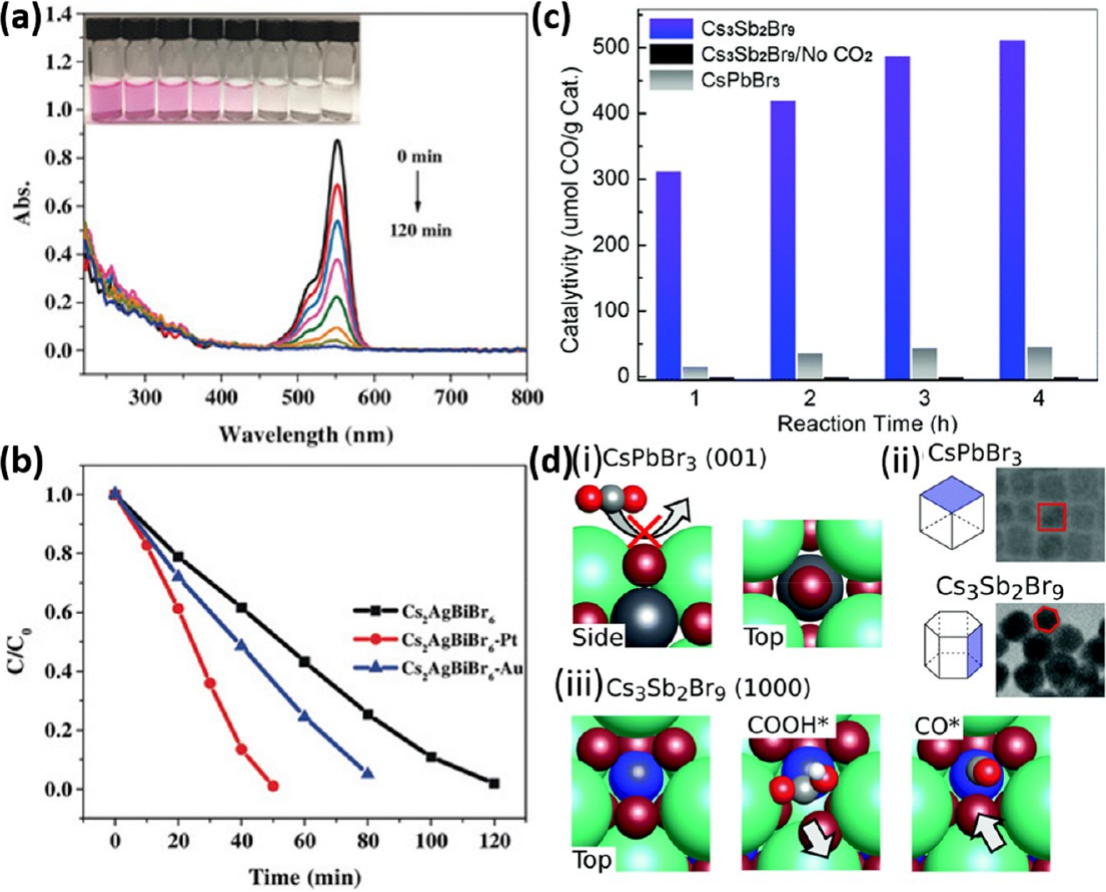
(a) UV–vis absorption spectra of Rhodamine-B in the presence of Cs_2_AgBiBr_6_ at different irradiation times (between 0 and 120 min). Inset: Digital photographs of the corresponding photocatalyst at different irradiation times. (b) *C*/*C*_0_ plot as a function of irradiation time for photodegradation of RhB in the presence of Cs_2_AgBiBr_6_, Cs_2_AgBiBr_6_–Pt, and Cs_2_AgBiBr_6_–Au. Reproduced with permission from ref (1333). Copyright 2019 John Wiley & Sons, Inc. (c) Photocatalytic activity toward production of CO by Cs_3_Sb_2_Br_9_ NCs compared to CsPbBr_3_ NCs. (d) (i) Inaccessiblile Pb atoms as shown on the CsPbBr_3_ (001) surface. (ii) TEM image showing cubic CsPbBr_3_ and hexagonal Cs_3_Sb_2_Br_9_ NCs, along with the planes of (001) for CsPbBr_3_ and (1000) for Cs_3_Sb_2_Br_9_. (iii) Reactivity of highly exposed Cs_3_Sb_2_Br_9_ NCs (1000) surface *via* partial displacement of one of the Br atoms. Reproduced with permission from ref (1335). Copyright 2020 Royal Society of Chemistry.

While lead-halide perovskites demonstrate significant potential toward different optoelectronic properties including photocatalysis, the toxic nature of Pb limits its large-scale application. Furthermore, metal centers (*e.g*., Bi, Sb) other than Pb may allow higher activity and better selectivity toward photocatalysis. An alcohol-based Pb-free Cs_2_AgBiBr_6_ double perovskite has been developed recently which shows a great promise toward dye degradation under visible-light irradiation with high chemical stability.^1333^ Cs_2_AgBiBr_6_ has been studied for photocatalytic degradation of Rhodamine-B (RhB), a common organic contaminant, under visible-light irradiation (Figure 140a), which shows up to 98% degradation of the dye upon a continuous irradiation for 120 min. The photocatalytic activity of Cs_2_AgBiBr_6_ was enhanced after depositing Au and Pt on the surface (Figure 140b), which improves the charge transport efficiency and has been verified using steady-state and time-resolved PL quenching measurements.

Although the photocatalytic activity of lead-free double perovskites is promising, the stability of this material remains challenging. In this respect, lead-free Cs_3_Sb_2_X_9_ and Cs_3_Bi_2_X_9_ defect-ordered perovskites are promising and have greater thermal stability.^544,1334^ The photocatalytic activity of Cs_3_Sb_2_Br_9_ perovskite for CO_2_ reduction reaction has been explored recently.^1335^ Unlike the commonly used solvent ethyl acetate or acetonitrile, in this work high boiling-point octadecene was chosen due to its larger CO_2_ solubility. Figure 140c compares the photocatalytic activity toward CO_2_ reduction of Cs_3_Sb_2_Br_9_ and CsPbBr_3_ perovskite NCs. Over the course of 4 h irradiation, CsPbBr_3_ NCs produces 50 mmol/g CO, which is higher than that in previous reports. This has been attributed to increased CO_2_ solubility as well as reduced degradation of perovskite NCs in octadecene compared to that with commonly used acetonitrile or ethyl acetate for photocatalytic reactions. Surprisingly, the activity of Cs_3_Sb_2_Br_9_ NCs to CO_2_ reduction is more than 10-fold higher, producing a total of 510 mmol/g CO after 4 h irradiation (Figure 140c). The control experiments in the absence of CO_2_ shows no CO production which confirms the result of CO generation is not from the degradation of ligands or solvent. The activity of both the catalysts was found to be reduced over the multiple reaction cycles. However, the Cs_3_Sb_2_Br_9_ NCs still showed a 5–10-fold larger activity than Pb-based CsPbBr_3_ NCs. Density functional theory calculations were performed to unravel the underlying cause for such enhanced activity in Cs_3_Sb_2_Br_9_ NCs.^1335^ No intermediate COOH*-bound states were observed on the CsPbBr_3_ NC (001) surface from the calculations. This is because the Pb atom is completely isolated from the surface by the Cs and Br atoms, as shown in Figure 140d(i), which restricts any direct interaction with COOH*. The (1000) and (0001) surfaces of Cs_3_Sb_2_Br_9_ NCs, however, have high exposure due to the hexagonal structure (Figure 140d(ii)). Here, the Sb atom is only partially shielded by three Br atoms (Figure 140d(iii)). An Sb–C bound state is observed in the DFT calculation for both (1000) and (0001) surfaces for COOH*, where one of the ionic Br ions displaces slightly to allow the formation of the Sb–COOH* bond. The smaller size of CO* allows the shifted Br to return to its initial position during the evolution of CO. Thus, DFT calculations show that the mechanism for the enhanced photocatalytic activity of Cs_3_Sb_2_Br_9_ NCs is due to the effective binding sites on the (1000) and (0001) surfaces for COOH* and CO* intermediates.

#### Photocatalytic Activity of Perovskite-Based Heterostructures

**Figure 141:**
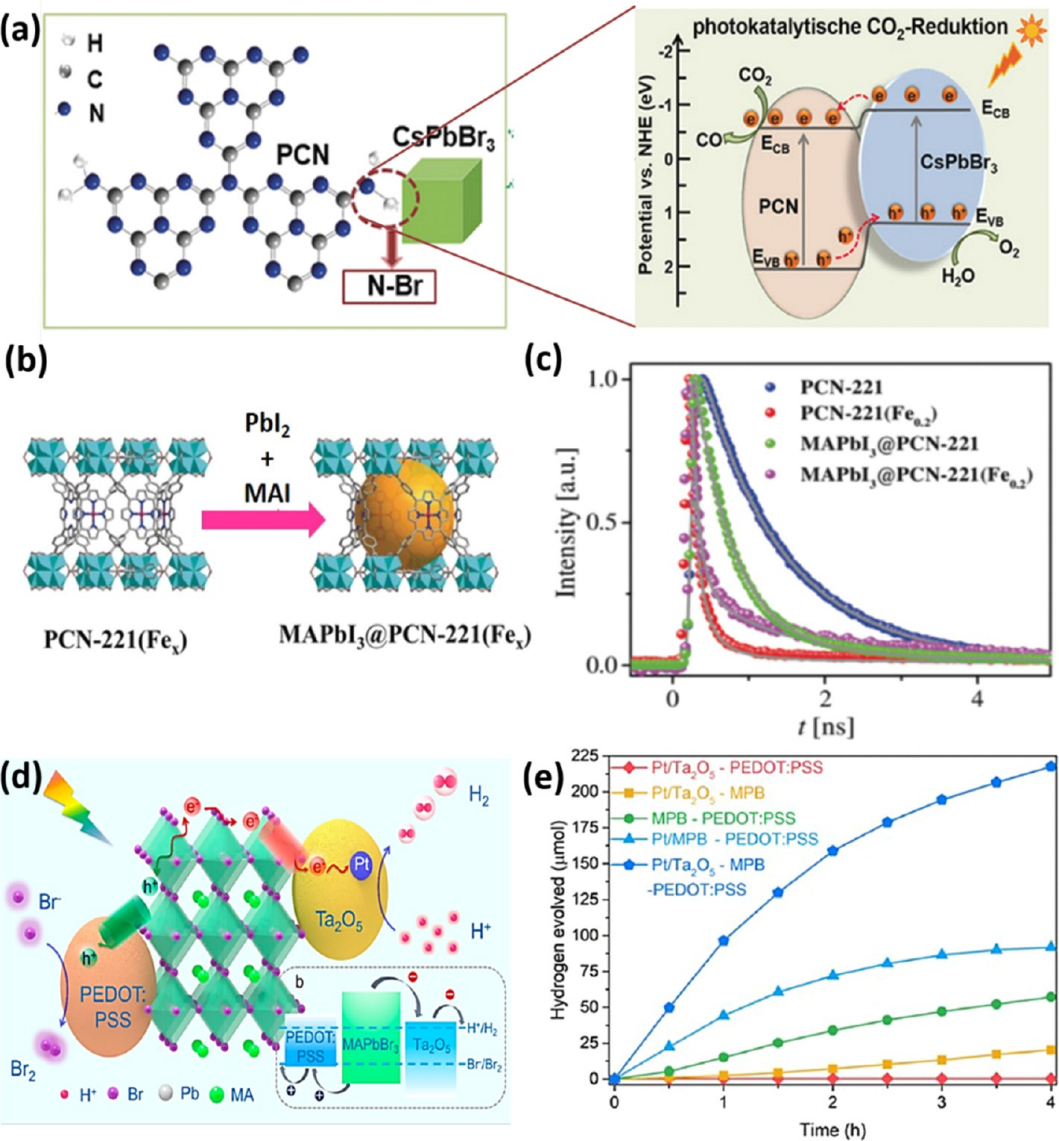
(a) Schematic illustrations for the interfacial interaction and band alignment within CsPbBr_3_ NCs/g-C_3_N_4_ heterojunction. Reproduced with permission from ref (1337). Copyright 2018 John Wiley and Sons. (b) Schematic illustration for the synthesis of MAPbI_3_@PCN-221(Fe_*x*_). (c) TRPL decays of different samples. Reproduced with permission from ref (1338). Copyright 2018 John Wiley & Sons, Inc. (d) Schematic illustration of the mechanism of photocatalytic HER over Pt/Ta_2_O_5_-MAPbBr_3_–PEDOT:PSS heterojunction. (e) Comparison of the H_2_ evolution activities of Pt/Ta_2_O_5_–PEDOT:PSS, Pt/Ta_2_O_5_-MAPbBr_3_, Pt/MAPbBr_3_–PEDOT: PSS, MAPbBr_3_–PEDOT:PSS, and Pt/Ta_2_O_5_-MAPbBr_3_–PEDOT:PSS. Reproduced from ref (1340). Copyright 2018 American Chemical Society.

The high absorption coefficient, defect-tolerance and tunable band positions of halide perovskites are strongly beneficial for photocatalysis. In addition to efficient charge separation and transfer, photocatalysts also require a high density of active sites, good stability and recyclability. Generally, it is difficult to satisfy all these requirements for a single-component halide perovskite photocatalyst. Owing to the synergistic properties induced by the interactions among different components, heterostructures of diverse functional materials into a single system with precise design is a commonly employed strategy to enhance the performance of semiconductors.^1336^ Halide perovskite-based heterostructures have therefore demonstrated improved performance. For instance, based on a facile self-assembly method, Ou *et al*.^1337^ prepared CsPbBr_3_ NCs anchored on porous g-C_3_N_4_ nanosheet heterojunctions for CO_2_ photoreduction. The intimate interface interaction enable by N–Br chemical bonding as well as the matched band alignment between CsPbBr_3_ and g-C_3_N_4_ semiconductors effectively facilitate the separation and transport of photogenerated carriers (Figure 141a). As a result, the optimal CsPbBr_3_/g-C_3_N_4_ heterojunction exhibits enhanced stability and CO production compared to CsPbBr_3_ NCs and g-C_3_N_4_ alone.

Metal-organic frameworks are also promising CO_2_ catalysts due to their porous crystalline framework offering a large specific surface area and highly active metal centers for selective CO_2_ absorption/activation. Recently, Wu *et al.*(1338) prepared Fe-based MOF-coated MAPbI_3_ perovskites (*i.e.*, MAPbI_3_@PCN-221(Fe_*x*_)) *via* a sequential deposition method (Figure 141b). TRPL measurements in Figure 141c suggest that the electron transfer from MAPbI_3_ to Fe-based MOFs reaction sites greatly promotes efficient charge separation. The MAPbI_3_@PCN-221(Fe_*x*_) can serve as efficient photocatalysts for CO_2_ reduction with the highest yield of 19.5 μmol g^–1^ h^–1^ for solar fuel production (CH_4_ + CO). In addition to MOFs, other porous materials such as silica matrixes,^1339^ TiO_2_^1293^, and graphene^38^ have also been employed as support to stabilize and disperse halide perovskite, thus tuning their photocatalytic performance.

As shown in Figure 141d, a multicomponent halide perovskite-based hybrid consisting of MAPbBr_3_ modified with Pt/Ta_2_O_5_ as electron transport layers and poly(3,4-ethylenedioxythiophene):polystyrenesulfonate as hole transport layers were reported by Wang *et al*.^1340^ The photocatalytic H_2_ evolution rate of this catalyst reached 105 μmol h^–1^, which was drastically increased about 52-fold over the pristine MAPbBr_3_ (Figure 141e). However, the photoactivity of this system decreased gradually with prolonged reaction times, indicating poor stability of the reaction. Sá and co-workers developed a photocatalytic reaction system that employed CsPbBr_3_ as the light-absorber and Ru@TiO_2_ nanoparticles as the proton reductant catalyst.^1341^ Stable H_2_ production was observed, which suggest that this reaction system can be a feasible platform for fundamental investigations on halide perovskites photoactivity and stability.

### Summary and Outlook for Perovskite Photocatalysis

Inspired by these pioneering works, various halide perovskite materials with tunable size, morphology and crystal structure have been prepared by a range of methods. These halide perovskites can also be incorporated with metal nanostructures,^1315^ semiconductors^1342^ and carbon-based materials^1343,1344^ to form heterojunction photocatalysts. Recent advances of halide perovskite in photocatalysis fields show that these materials can be used to drive H_2_ evolution, CO_2_ reduction, degradation of organic pollutants, and selective organic synthesis. Thus, it may be concluded that the renaissance of halide perovskites in the photovoltaic and optoelectronic fields has also sparked considerable interest in their photocatalytic applications. Currently, the highest CO_2_ to solar fuel (CO + CH_4_) production rate has reached 431 μmol g^–1^ h^–1^ with transition metal Ni complex modified CsPbBr_3_,^1345^ and the maximum H_2_ generation rate of 7.3 mmol g^–1^ h^–1^ was gained with BP/MAPbI_3_ heterojunctions.^1342^ Despite the exciting progress, the field is still at its infancy and there is great room for the design of target reaction systems, enhancing the stability and efficiency and eliminating toxicity of the halide systems for solar to chemical energy conversion.

The future development of halide perovskite-based photocatalysts can be divided into the following aspects:

1. The reaction type and scope of metal-halide perovskites can be expanded by fine-tuning the structural composition which may lead to efficient manipulation of the band gap and alignment. For example, doping Sn in the B-site of MAPbI_3_ lead to reduction in the band gap which may lead to enhanced light absorption. Such engineering may lead to the development of different types of photocatalysts with improved charge-transfer efficiency.

2. The photocatalytic performance of metal-halide perovskites can be improved by structural engineering toward stability, reactivity and selectivity, *e.g*., by ligand engineering, doping, surface modification with cocatalysts, surface passivation layers. This may result in increasing stability as well as enhanced reactivity and boost in the photocatalytic performance. For example, the stability can be enhanced using bulky organic ligands (*e.g*., butylamine) which may reduce the dimension of 3D perovskite to 2D perovskite. The diffusion length can be enhanced in MAPbI_3_ by A-site (X-site) doping of MA (I) with FA (Br) and suppress electron–hole recombination, leads to increased reactivity. For selective charge-extraction, several electron and hole transporting materials (*e.g*., GO, MOF, *etc.*) can be used in combination with the perovskite, as discussed above.

3. Furthermore, development of efficient eco-friendly Pb-free metal-halide perovskites by replacing Pb with other transition metals (*e.g*., Sn, Sb, Bi, Ag, *etc.*) is necessary, though the Pb-free perovskites suffer from reduced activity. Thus, development of such Pb-free photocatalysts should occur in combination with several improving strategies.

## Overall Summary and Outlook

Over the last few years, perovskite NCs have quickly emerged as an important class of semiconductors. Research into perovskite NCs has been sparked not only because of their intriguing fundamental optical and electronic properties, but also by their appeal in many semiconductor-based technologies. This review has covered most of the lines of research that are being carried on perovskite NCs. Most of these lines of research only started a few years ago, and range from synthesis to self-assembly and characterization, through to applications. Tremendous research progress has been made in these various research areas in a short span of time, yet there are many open questions and challenges to be addressed to move the field forward.

### Shape/Composition-Controlled Synthesis and Self-Assembly of MHP NCs

A wide range of synthetic methods have been developed for the preparation of perovskite NCs on a large scale using different precursors and ligands. Various morphologies include nanocubes, NPls, NWs, and NRs. The size and shape of the perovskite NCs is usually controllable by the reaction temperature, the ratio of acid–base ligands, precursor ratio (A to B), the alkyl chain length of the ligands, and the thermodynamic equilibrium of the reaction.^18,47,60,145,150^ However, the level of shape control in MHP NCs is far from what has been achieved for metal nanoparticles and classical colloidal quantum dots. Most of the synthesis methods reported for MHP NCs generally yield nanocubes or cuboid morphologies.^36,52^ The crystallization of perovskite NCs is an extremely fast process, which makes it difficult to probe their growth mechanisms. Therefore, it is still challenging to understand the nucleation and growth processes of perovskite NCs for a precise control of their morphology. An approach that could be used to slow-down the reaction speed is using precursors that react at a lower rate. In general, fast nucleation and growth result in isotropic NCs, while slow growth processes lead to anisotropic colloidal NCs. This is indeed the case for metal NPs. However, in the case of perovskite NCs, it is still unclear how 2D NPls are formed from an isotropic crystal lattice and homogeneous reaction environment. It is most likely that the symmetry breaks as soon as nucleation occurs and the NCs grow into 2D shapes rather than 3D. Another possibility is that the ligands could bind to specific facets of the nucleus more strongly than others and restrict growth, resulting in growth being anisotropic. To prove these speculations, in-depth studies on growth mechanisms are needed. On the other hand, it has been revealed that the formation of perovskite NWs occurs through the oriented-attachment of nanocubes rather than a seed-mediated growth process.^22,186,233,1346^ Although this is well-understood for thick (10–12 nm thickness) nanowires, the growth mechanism of ultrathin (2–3 nm) NWs is still unclear. Despite decent progress in inorganic perovskite NWs, controlling their length scales is still challenging. One possible way to better control the shape of perovskite NCs is to further elaborate on the use of preformed, sub-nanometer perovskite clusters, as those developed by Peng *et al.* and employed for the synthesis of perovskite NCs of different shapes.^316^ These clusters are expected to be less reactive than the direct metal and halide precursors and are already capped by ligands, providing at the same time all what is needed for the synthesis of NCs and preventing a massive nucleation of NCs.

Also, the level of control over the shape and polydispersity achieved in inorganic perovskite NCs has not been realized in OIHP NCs. In fact, researchers have paid more attention toward inorganic perovskite NCs due to their higher stability and shape purity compared to OIHP NCs. Despite the poor stability caused by the organic component, thin films of OIHPs have been shown to be potential candidates for photovoltaics. Therefore, it would be interesting to pay more attention to OIHP NCs in the future and compare their properties with inorganic perovskite NC.

One of the most interesting properties of perovskite NCs is their tunable PL by the constituted halide composition. Halide ion exchange in perovskites is relatively easy and it takes place at room temperature due to spontaneous halide ion migration, and has been applied to LHP NCs of different morphologies to tune their emission color. However, spontaneous halide exchange is a problem for the fabrication of white LEDs based on all-perovskite NCs. A few reports demonstrate the prevention of halide exchange between perovskite NCs of different halide components, but then the surface coatings used for preventing halide exchange can be a problematic for charge carrier transport. Therefore, this issue needs further attention in the future. In addition, cation exchange reactions have also been applied to obtain mixed cation perovskite NCs with distinct optical properties as compared to either all-inorganic or OIHP NCs. However, this has been mostly applied to nanocubes. It would be important to determine if anisotropic NCs such as NPls, NWs and NRs retain their shape after cation exchange. More importantly, the mechanism of cation exchange is not yet well-understood. There is still an open question regarding whether the addition of cations can lead to re-nucleation or to an actual, topotactic replacement of the original cations of the crystal lattice. There is also a considerable work to be done on the transformations involving cesium-halide NCs and their interconversions.^1347^ In this list, we consider CsX, Cs_4_PbX_6_, the perovskite phase of CsPbX_3_, and CsPb_2_Br_5_. It has been recently shown by Toso *et al.*(1347) that there is a common thread linking all these materials, that is, the Cs^+^ cation substructure: this substructure is expanded/contracted and/or twisted when one material of this class converts into another material of the same class, but it is not destroyed. This helps to rationalize the observation of hepitaxial interfaces (some in NCs, other in bulk films), for example, CsBr/CsPbBr_3_, Cs_4_PbBr_6_/CsPbBr_3_, and CsPbBr_3_/CsPb_2_Br_5_, in which there is a continuity of the Cs^+^ substructure across the interface. This mechanism of preservation of the large “A” cation might be more general and expandable to a broad series of metal halides, and it would be interesting to the study other possible transformations in perovskite and perovskite-related materials.

The soft and highly dynamic nature of the perovskite crystal lattice results in liquid-like properties. This property makes the aggregated perovskite NCs perfectly single-crystalline. For instance, it has been shown that CsPbBr_3_ nanocubes and NPls can transform into single-crystalline NWs and nanobelts, respectively.^22,234^ Similarly, it has been often observed that the nanocubes on TEM grids connect with their neighboring nanocubes either side-by-side or corner-to-corner.^319^ Very interestingly, most connected NCs appear to be single-crystalline, suggesting the liquid crystalline behavior of the lattice. However, it is still unclear how the lattice restructures at the connected joints. Further investigation will be required through high-resolution electron microscopy into what happens at the connected joints of the NCs.^22^

### Surface Chemistry and Surface Passivation/Coating of MHP NCs

There has been significant progress in the understanding of the surface chemistry of perovskite NCs through NMR studies, which were aimed to explore how the ligands could bind and stabilize the NC surface. It has often been stated that the ligands control the growth process, but we have only limited knowledge of how the ligands control the nucleation and growth of perovskite NCs. The studies suggest that bidentate and tridentate ligands are more suited to stabilizing the NC surface compared to the routinely used OLA/OA system. However, the chemistry behind ligand coordination to the NC surface remains unclear. It has been stated that the ligands are weakly bound to the surface of perovskite NCs and that this binding is highly dynamic due to the ionic character of such binding. The ligands are easy to detach from the NC surface during washing with polar solvents, and this creates surface defects, which affects their PLQY. A large number of studies have been focused on surface passivation of LHP NCs using various ligand molecules and metal halides to recover their PLQY. However, we know very little about the surface passivation mechanism at the atomic level. It is still unclear whether the ligand molecules alone can passivate the surface or if metal halides are compulsory to fill Pb and halide vacancies. More importantly, it is worth mentioning that there are differences in the trap energies and the interactions of ligands with perovskites of different halide compositions. Therefore, we cannot generalize the surface passivation mechanism for all halides. Until now, most reported studies into surface passivation have focused on the CsPbBr_3_ NC system to improve their PLQY to near-unity.

One of the important problems associated with perovskite NCs is their instability in water. To address this issue, LHP NCs have been coated with various shell materials such as bulky organic ligands, TiO_2_, SiO_2_, Al_2_O_3_, and block copolymers. However, these shells affect charge transport in corresponding optoelectronic devices. Therefore, more research efforts are needed to find conductive shells for perovskite NCs to improve their stability in water, but without affecting charge transport.

### Future Prospects of 0D Non-perovskite NCs

We have summarized the recent developments in the synthesis, phase transformation, and optical features of Cs_4_PbBr_6_ NCs, particularly focusing on the material’s molecular behavior, the origin of green emission, and optoelectronic applications. However, there are still many challenges and possibilities lie ahead for exploring these class of materials in optoelectronics. Here, we share a few future prospects for the advancement of fundamental understanding of Cs_4_PbBr_6_ NCs as well as the development of additional 0D NCs, which would facilitate their applications.

1. Although different synthesis methods have been developed for Cs_4_PbX_6_ NCs, other 0D A_4_PbX_6_ NCs, such as Rb_4_PbBr_6_, have not yet been reported. Thus, there is a large scope for the development of synthesis methods that allow precise control over the size and phase of 0D NCs with different A-site cations, and for uncovering the relationship between A-site cations and the optical properties of A_4_PbX_6_ NCs.

2. The origin of green emission in Cs_4_PbBr_6_ NCs is still under debate. It is attributed to the presence of 3D CsPbBr_3_ impurities as well as defect-related emission. Therefore, sophisticated synthesis and characterization methods are needed to identify their emissive centers. For example, to confirm the role of defect-induced emission, low-dose HRTEM and data processing methods can be used to image the point defects in Cs_4_PbBr_6_ nanoplates of thickness less than 2 nm.

3. Developing lead-free 0D NCs for optoelectronic applications is another important research direction regarding this class of materials. For instance, Cs_4_SnBr_6_ NCs were recently synthesized, and they exhibit the characteristic green emission with enhanced air stability in the form of both colloidal suspensions and thin films.^800^ Furthermore, it was demonstrated that the lead-free Cu(I)-based 0D NCs (*i.e.*, Cs_3_Cu_2_X_5_) display efficient luminescence and improved stability compared to that of Pb-based 0D NCs.^558,1348^

4. Like Pb-free perovskite NCs, stability is also a major concern for Pb-free 0D NCs. To address the issue related to oxidation of Sn(II)- and Cu(I)-based 0D NCs, core/shell strategy can be used. Theoretical studies have predicted that A_4_SnX_6_/A_4_PbX_6_ core/shell-type NCs exhibit type-I energy level alignment for promoting the energy transfer from shell to the core and thus boosting the emission of A_4_SnX_6_ core.^1349^ However, additional innovations in synthesis methods are required to realize 0D core/shell NCs.

### Outstanding Questions into the Doping of MHP NCs

Recently, there has been an explosion of research into the doping of perovskites with various metal ions, with the aim of improving stability, improving PLQY, and tuning the emission wavelength. Despite great progress into the doping of perovskite NCs, there are still a number of transition and inner transition metals that remain unexplored. With different dopants, additional optical and magnetic properties may be achieved. While B-site doping is largely explored, there should be more focus on A-site doping and on the influence on the stability and properties of the NCs. One of the important and open questions in the doping of perovskites is the exact location of the dopant sites in perovskite NCs. In most studies, it has been speculated that the dopants occupy the A-site or B-site regardless of their sizes. However, one should know that if the size of the dopant ion is too different from that of the cations of the host matrix, there may be phase segregation or the dopants destabilize the perovskite crystal structure. It is still remains unexplored whether the dopants are substitutional in the crystal lattice or they simply stay on the surface of the crystal lattice.

### Pb-Free Perovskite NCs

Beyond lead-containing perovskite NCs, a wide range of lead-free alternatives have been explored. These are termed perovskite-inspired materials (PIMs) because the main motivation is to find materials that could replicate the exceptional optoelectronic properties of the lead-halide perovskites. PIMs include halide perovskites based on Sn and Ge (ABX_3_), Cu-based materials, Sb- and Bi-based vacancy-ordered perovskites (A_3_B_2_X_9_), double perovskites (A_2_B(I)B(III)X_6_), and vacancy-ordered double perovskites (A_2_B(IV)X_6_). The synthesis routes are similar to those for lead-halide perovskites. Although the performances (such as PLQY, narrowness of the PL line width) of these materials have not matched the lead-based perovskites, they have given rise to distinctive applications. These include blue phosphors (namely, with A_3_B_2_X_9_ compounds), which lead to white-light emission when combined with conventional yellow phosphors. Other materials (namely, double perovskites) have demonstrated promise as white-light phosphors. This emission is attributed to self-trapped excitons. The key advantage of these phosphors is that the materials demonstrate improved ambient and thermal stability over lead-halide perovskites. However, it is currently rare to find examples of lead-free NCs used in electrically driven applications. Promising results have so far been obtained from Cs_3_Cu_2_I_5_ and Cs_3_Sb_2_Br_9_ NC LEDs, and there has also been the demonstrations of direct injection into self-trapped excitons in tin-based perovskites. Further work on improving the properties of lead-free NCs and developing these materials for electrically driven applications is still needed.

### Morphological and Structural Characterization

The characterization of perovskite NCs by TEM and X-ray scattering techniques is important for understanding their structure-property relationships. Perovskites are highly sensitive to the high-energy electron beam, which can lead to structural damage or phase transitions. NCs are particularly susceptible because Pb degradation products preferentially form at edges and corners. In particular, OIHP NCs are very difficult to characterize by high-resolution TEM because of the rapid degradation of the organic component. Using instruments with higher sensitivity has enabled reduced dosing of perovskites during characterization. As an example, this made it possible for MAPbBr_3_ NCs to be measured with atomic resolution by TEM. However, unlike metal NPs, electron microscopy has not been utilized with its full potential in the characterization of perovskite NCs due to its beam sensitivity issue. Therefore, there are many open questions to be addressed by electron microscopy. For example, it has been proposed that perovskites undergo phase changes at certain temperatures, and to probing such phase changes at the atomic level with *in situ* characterization at the single-particle level will provide important insights. Another important question to be addressed is the 3D atomic imaging of perovskite NCs and this can solve the issues associated with the crystallinity of perovskite NCs. In addition, electron microscopy could play an important role in identifying the location of dopants in doped perovskite NCs. On the author hand, X-ray scattering techniques have been used extensively to characterize the crystallinity of bulk and NC perovskites. X-rays have previously been used to study the nucleation and growth mechanism of metal NCs. Extending such studies to perovskite NCs would improve the understanding of their growth process. In addition, small-angle X-ray scattering techniques could help us unfold the assembly of ligand molecules on the surface of perovskite NCs.

### Outstanding Questions in Optical Properties of MP NCs

Perovskites have become popular for their interesting properties. Unlike classical QDs, perovskite NCs exhibit extremely high PLQY without having any shell on their surface. This is attributed to the shallow character of the defect-related energy states, which enables defect tolerance, that is, low nonradiative recombination rates despite high densities of defects. However, recent studies have shown that the surface traps generated by the detachment of ligands and surface atoms from perovskite NCs can have a drastic effect on their PLQY. It appears therefore that the nature of surface traps is not yet fully understood. The energy and nature of the traps created by the detached ligands need to be assessed, especially since the traps created may not follow thermodynamic predictions. In addition, the role of ligands on the optical properties of perovskite NCs has not been investigated in detail. In particular, ligands can significantly influence the optical properties of thinner nanostructures such as NPls and ultrathin NWs. One of the ongoing debates about light emission in LHP NCs is the exciton fine structure, which governs the radiative *versus* nonradiative recombination rates significantly. Although initially it was believed that the lowest exciton state of LHP NCs is bright, while the highest exciton state is dark, later investigations suggested the opposite. As transition metal ion doping in LHP NCs has been gaining increasing attention, more in-depth understanding is needed on how the crystal field resulting from doping induces the splitting of bright *versus* dark excitonic states. On the other hand, 0D non-perovskites, and Pb-free perovskites are emerging as potential semiconducting materials for white light generation from self-trapped excitons. However, the formation of self-trapped excitons and the photophysics in such materials is still not well-understood. Very recently, chiral perovskite NCs have been receiving significant attention due to their polarized emission. In most cases, chirality in perovskites is induced by chiral ligands. However, the origin of the induced chirality in perovskites is still under debate. Several mechanisms, such as chiral molecules-induced symmetry breaking in the crystal lattice, dipolar interactions between chiral molecules and perovskites, and spin–orbit coupling, have been proposed for the origin of chirality, and these need further in-depth investigations in the future.

Another important phenomenon of MHP NCs that requires further understanding is photoluminescence intermittency, which is also known as “blinking”. This limits the application of these materials in quantum optical devices. Single-particle investigations of MHP NCs suggest that this effect is intrinsic to the materials, rather than the effect of the processing route, and it has been found that blinking occurs not only in quantum-confined systems but also in microcrystals. Several mechanisms have been put forward to explain how blinking occurs. These include the effects of photocharging and Auger recombination, or the effects of nonradiative recombination centers that could be metastable. However, further work is needed to understand how metastable defects could be activated/deactivated, and whether light could play a role. The density of these metastable defects also needs to be more reliably measured. In addition to blinking, single-particle investigations have also shown that electron–phonon coupling in MHP NCs affects the emission spectra, leading to extra PL peaks. However, there is debate in the literature as to the degree of coupling between electrons and optical *versus* acoustic phonons. Furthermore, understanding into these phenomena could lead to insights of how charge transport could be improved.

### Applications

MHP NCs have gained significant attention for applications involving optical emission and absorption. These include lasing, in which MHP NCs could lead to cost-effective solid-state lasers with emissions wavelengths that can be easily tuned. Here, we foresee three key challenges. First, the MHP NCs are unstable to heat and environmental stress, and require encapsulation strategies (*e.g.*, NCs embedded in a glass matrix or in a Cs_4_PbBr_6_ matrix). Second, most work has been on Pb-based materials, and nontoxic alternatives that are air-stable need to be developed. These include double perovskites, but the PLQY in many lead-free alternatives have not matched the near-unity values the Pb-based NCs can be routinely obtained (as discussed above). Third, lasing in MHPs has only been achieved through optical pumping. Electrical pumping has been elusive, due to Auger recombination at high injection rates and the long-chain ligands used with NCs.

On the other hand, electrically driven spontaneous emission from MHP NCs has been achieved, and efficient LEDs based on lead-halide perovskite NCs have been demonstrated, with EQEs exceeding 20% after only 5 years of development. Perovskite LEDs also have the advantages of high-color purity, ultrawide color gamut, potential for low materials and fabrication costs, as well as compatibility with the existing manufacturing technology for OLEDs/QD-LEDs. Thus far, most efforts have focused on improving the EQEs of perovskite LEDs. However, it is also important to develop an understanding behind these improvements in performance, which will be important for rationally achieving further increases in efficiency. It will also be important to scale-up perovskite LEDs from the mm-level to large-area displays with nanometer-level uniformity in terms of NC size and emission wavelength. The stability of perovskite LEDs needs to be improved, particularly under operation. Furthermore, the development of perovskite LEDs has been focused on green, red, and near-infrared emitters, which have achieved the highest EQEs (of >20%). More recently, there have been significant efforts to develop blue emitters, owing to their importance for full-color displays, but both the EQE and stability lag behind their green and near-infrared counterparts. Beyond these challenges, it will also be important to replicate the high performance of lead-halide perovskites in lead-free alternatives. Currently, this is challenging because Sn- and Ge-based perovskites are less stable than the Pb-based perovskites, and many of other materials that have been proposed as alternatives have indirect band gaps and low PLQYs.

MHPs are promising for photodetectors and radiation detectors due to their high optical absorption coefficients, high *Z* numbers (ensuring strong attention of radiation) and long diffusion lengths. In photodetectors, NCs with reduced defect density have been achieved, leading to devices with high on/off ratios for the photocurrent exceeding 10^5^. Nanostructured perovskites have also been realized in 1D and 2D structures and combined with carbon nanotubes or 2D materials to demonstrate enhanced performance. In radiation detectors, NCs have shown promise for X-ray scintillators, which rapid response times and low X-ray detection limits demonstrated.

Furthermore, perovskites have been explored for FETs, where the ambipolar nature of charge transport could offer interesting possibilities. However, one the important challenges to overcome is the low field-effect mobility, which arises in part from ion migration. Passivating surface defects in NCs may aid in addressing this.

Perovskite NCs have also demonstrated significant promise in solar cells, with PCEs >16% achieved, which represents the highest efficiencies for any QD-based solar cell. NCs offer the advantage of stabilizing metastable phases, such as the α-phase of CsPbI_3_, which led to >10% efficient devices. NCs in particular offer the important advantage of high PLQYs, which result in low nonradiative losses. The open-circuit voltages of NC perovskite solar cells have therefore been closer to the radiative limits than bulk thin film perovskites. The NCs are also amenable to alloying, and the most efficient NC perovskite solar cells use a mixture of Cs and FA in the A-site, which leads to a smaller band gap than pure Cs-based perovskites. Finally, perovskite NCs have just started to receive significant attention as photosensitizers in photocatalysis. Perovskite photocatalysis has already been demonstrated for H_2_ evolution, CO_2_ reduction, the degradation of organic pollutants and selective organic synthesis. However, the field is still young, and there are still many possibilities that remain to be explored. Some of the challenges include enhancing stability and performance as well as developing more effective encapsulation strategies.

## Supporting Information Available

Movie S1: Synthesis of MAPbBr_3_ NCs by ligand-assisted reprecipitation method (MP4)
Movie S2: Degradation of a CsPbBr_3_ nanocube upon continuous scanning of the electron beam (MPG)
Movie S3: Large-scale synthesis of CsPbBr_3_ nanocubes (MP4)
HAADF-STEM images of CsPbBr_3_ nanocubes acquired under continuous electron beam illumination (PDF)

## Supplementary Material

nn0c08903_si_001.mp4

nn0c08903_si_002.mpg

## Vocabulary

**halide perovskites**: a family of materials with the basic chemical formula ABX_3_, in which A and B are cations, and X is a halide ion; the perovskite family can be extended by combining two different A-site cations or two B-site cations or by combining vacancies (giving rise to vacancy-ordered perovskites)
**quantum confinement**: localization of electrons and holes into a narrow space with dimension comparable to or smaller than the Bohr radius, leading to the increased coupling between electron–hole pairs and their energy levels becoming discrete and controllable by the size of the material; quantum confinement can be achieved along one dimension (nanoplatelets), two dimensions (nanowires), and three dimensions (quantum dots)
**zwitterionic ligand**: chemical species (usually comprising a carbon backbone) with both positively and negatively charged functional groups that can bind to the surface of NCs
**surface passivation**: a surface treatment process that can reduce unwanted carrier recombination process by eliminating defects on the surface of the materials for improving their optoelectronic properties such as photoluminescent quantum yield and carrier lifetime
**superlattice or supercrystals**: long-range-ordered array of nano- and microparticles
**photoluminescence blinking (or intermittency)**: stochastic fluctuations in photoluminescence intensity
**electroluminescence**: spontaneous emission obtained through the injection of electrons and holes into an emissive layer
