# Supplementary material for: State of the Art and Prospects for Halide Perovskite Nanocrystals
Source: ACS Nano. 2021 Jun 17;15(7):10775–981. doi: 10.1021/acsnano.0c08903 (PMC8482768; doi:10.1021/acsnano.0c08903)
Supplement: Supplementary file 4 — nn0c08903_si_004.pdf [file nn0c08903_si_004.pdf]

SUPPORTING INFORMATION

State of the Art and Prospects for Halide Perovskite Nanocrystals

Amrita Dey,[†] Junzhi Ye,[‡] Apurba De,[§] Elke Debroye,[∥] Seung Kyun Ha,[#] Eva Bladt,[⊥,7] Anuraj S. Kshirsagar,[8] Ziyu Wang,[9] Jun Yin,[10] Yue Wang,[11] Li Na Quan,[12,13] Yan Fei,[14] Mengyu Gao,[13,15] Xiaoming Li,[11] Javad Shamsi,[‡] Tushar Debnath,[†] Muhan Cao,[16] Manuel A. Scheel,[17] Sudhir Kumar,[18] Julian A. Steele,[19] Marina Gerhard,[20] Lata Chouhan,[21] Ke Xu,[22, 23] Xian-gang Wu,[24] Yanxiu Li,[25] Yangning Zhang,[26] Anirban Dutta,[27] Chuang Han,[28] Ilka Vincon,[†] Andrey L. Rogach,[25] Angshuman Nag,[8] Anunay Samanta,[§] Brian A. Korgel,[26] Chih-Jen Shih,[18] Daniel R. Gamelin,[29] Dong Hee Son,[30] Haibo Zeng,[11] Haizheng Zhong,[24] Handong Sun,[31] Hilmi Volkan Demir,[14,31,32] Ivan G. Scheblykin,[20] Iván Mora-Seró,[33] Jacek K. Stolarczyk,[†] Jin Z. Zhang,[22] Jochen Feldmann,[†] Johan Hofkens,[∥,34] Joseph M. Luther,[35] Julia Pérez-Prieto,[36] Liang Li,[37] Liberato Manna,[38] Maryna I. Bodnarchuk,[39,40] Maksym V. Kovalenko,[39,40] Maarten B.J. Roeffaers,[19] Narayan Pradhan,[27] Omar F. Mohammed,[10] Osman M. Bakr,[41] Peidong Yang,[12, 13, 15, 42] Peter Müller-Buschbaum,[17,43] Prashant V Kamat,[44] Qialiang Bao,[45] Qiao Zhang,[16] Roman Krahne,[46] Raquel E. Galian,[36] Samuel D Stranks,[‡, 47] Sara Bals,[⊥,7] Vasudevanpillai Biju,[21] William A. Tisdale,[#] Yong Yan,[28] Robert LZ Hoye[48*] and Lakshminarayana Polavarapu[†,49*]

[†] Chair for Photonics and Optoelectronics, Nano-Institute Munich, Department of Physics, Ludwig-Maximilians-Universität (LMU), Königinstrasse 10, 80539 Munich, Germany

[‡] Cavendish Laboratory, University of Cambridge, 19 JJ Thomson Avenue, Cambridge CB3 0HE, United Kingdom

[§]School of Chemistry, University of Hyderabad, Hyderabad 500 046, India

[∥]Department of Chemistry, KU Leuven, 3001 Leuven, Belgium

[#]Department of Chemical Engineering, Massachusetts Institute of Technology, Cambridge, Massachusetts 02139, United States

[⊥]EMAT, University of Antwerp, Groenenborgerlaan 171, 2020 Antwerp, Belgium

[7]NANOlab Center of Excellence, University of Antwerp, 2020 Antwerp, Belgium.

[8]Department of Chemistry, Indian Institute of Science Education and Research (IISER), Pune 411008, India

[9]School of Science and Technology for Optoelectronic Information ,Yantai University, Yantai,

Shandong Province 264005, P. R. China

[10]Advanced Membranes and Porous Materials Center, KAUST Catalysis Center, Division of Physical Science and Engineering, King Abdullah University of Science and Technology, Thuwal 23955-6900, Kingdom of Saudi Arabia

[11]MIIT Key Laboratory of Advanced Display Materials and Devices, Institute of Optoelectronics & Nanomaterials, College of Materials Science and Engineering, Nanjing University of Science and Technology, Nanjing 210094, China

[12]Department of Chemistry, University of California, Berkeley, Berkeley, California 94720, United States

[13]Materials Sciences Division, Lawrence Berkeley National Laboratory, Berkeley, California 94720, United States

[14]LUMINOUS! Center of Excellence for Semiconductor Lighting and Displays, TPI-The Photonics Institute, School of Electrical and Electronic Engineering, Nanyang Technological University, Singapore, 639798 Singapore

[15]Department of Materials Science and Engineering, University of California, Berkeley, California 94720, United States

[16]Institute of Functional Nano & Soft Materials (FUNSOM), Jiangsu Key Laboratory for Carbon-Based Functional Materials and Devices, Soochow University, 215123, Suzhou, China

[17]Lehrstuhl für Funktionelle Materialien, Physik Department, Technische Universität München, James-Franck-Str. 1, 85748 Garching, Germany

[18] Institute for Chemical and Bioengineering, Department of Chemistry and Applied Biosciences, ETH-Zurich, CH-8093 Zürich, Switzerland

[19]MACS Department of Microbial and Molecular Systems, KU Leuven, 3001 Leuven, Belgium

[20]Chemical Physics and NanoLund, PO Box 124, Lund University, 22100 Lund, Sweden

[21]Graduate School of Environmental Science and Research Institute for Electronic Science, Hokkaido University, Sapporo, Hokkaido 001-0020, Japan

[22]Department of Chemistry and Biochemistry, University of California, Santa Cruz, CA 95064, United States of America

[23]Multiscale Crystal Materials Research Center, Shenzhen Institute of Advanced Technology, Chinese Academy of Sciences, Shenzhen 518055, China

[24]Beijing Key Laboratory of Nanophotonics and Ultrafine Optoelectronic Systems, School of Materials Science & Engineering, Beijing Institute of Technology, 5 Zhongguancun South Street, Haidian District, Beijing 100081, China

[25]Department of Materials Science and Engineering, and Centre for Functional Photonics (CFP), City University of Hong Kong, 83 Tat Chee Avenue, Kowloon, Hong Kong S.A.R.

[26]McKetta Department of Chemical Engineering and Texas Materials Institute, The University of Texas at Austin, Austin, TX 78712-1062, USA

[27]School of Materials Sciences, Indian Association for the Cultivation of Science, Kolkata 700032, India

[28]Department of Chemistry and Biochemistry, San Diego State University, San Diego, California 92182 USA

[29]Department of Chemistry, University of Washington, Seattle, Washington 98195, United States

[30]Department of Chemistry, Texas A&M University, College Station, Texas 77843, United States

[31]Division of Physics and Applied Physics, School of Physical and Mathematical Sciences and Centre for Disruptive Photonic Technologies (CDPT), Nanyang Technological University, Singapore 637371, Singapore

[32]Department of Electrical and Electronics Engineering, Department of Physics, UNAM-Institute of Materials Science and Nanotechnology, Bilkent University, Ankara, 06800 Turkey

[33]Institute of Advanced Materials (INAM), Universitat Jaume I, 12071 Castelló, Spain.

[34]Max Planck Institute for Polymer Research, Mainz 55128, Germany

[35]National Renewable Energy Laboratory, Golden, Colorado 80401, United States.

[36]Institute of Molecular Science, University of Valencia, c/ Catedrático José Beltrán 2, Paterna, Valencia 46980, Spain

[37]School of Environmental Science and Engineering, Shanghai Jiao Tong University, Shanghai 200240, China

[38]Nanochemistry Department, Istituto Italiano di Tecnologia, Via Morego 30, Genova 16163, Italy

[39]Institute of Inorganic Chemistry and § Institute of Chemical and Bioengineering, Department of Chemistry and Applied Bioscience, ETH Zurich, Vladimir Prelog Weg 1, CH-8093 Zürich, Switzerland

[40]Laboratory for Thin Films and Photovoltaics, Empa−Swiss Federal Laboratories for Materials Science and Technology, Überlandstrasse 129, CH-8600 Dübendorf, Switzerland

[41]KAUST Catalysis Center, Division of Physical Science and Engineering, King Abdullah

University of Science and Technology, Thuwal 23955-6900, Kingdom of Saudi Arabia

[42]Kavli Energy NanoScience Institute, Berkeley, California 94720, United States

[43]Heinz Maier-Leibnitz Zentrum (MLZ), Technische Universität München, Lichtenbergstr. 1, Garching, Germany

[44]Notre Dame Radiation Laboratory, Department of Chemistry and Biochemistry, University of Notre Dame, Notre Dame, IN, 46556, United States

[45]Department of Materials Science and Engineering and ARC Centre of Excellence in Future Low-Energy Electronics Technologies (FLEET), Monash University, Clayton, Victoria 3800, Australia

[46]Istituto Italiano di Tecnologia, Via Morego 30, 16163 Genova, Italy

[47]Department of Chemical Engineering and Biotechnology, University of Cambridge, Cambridge, CB3 0AS, UK

[48]Department of Materials, Imperial College London, Exhibition Road, London SW7 2AZ, UK

[49]CINBIO, Universidade de Vigo, Materials Chemistry and Physics group, Departamento de Química Física, Campus Universitario As Lagoas, Marcosende, 36310 Vigo, Spain

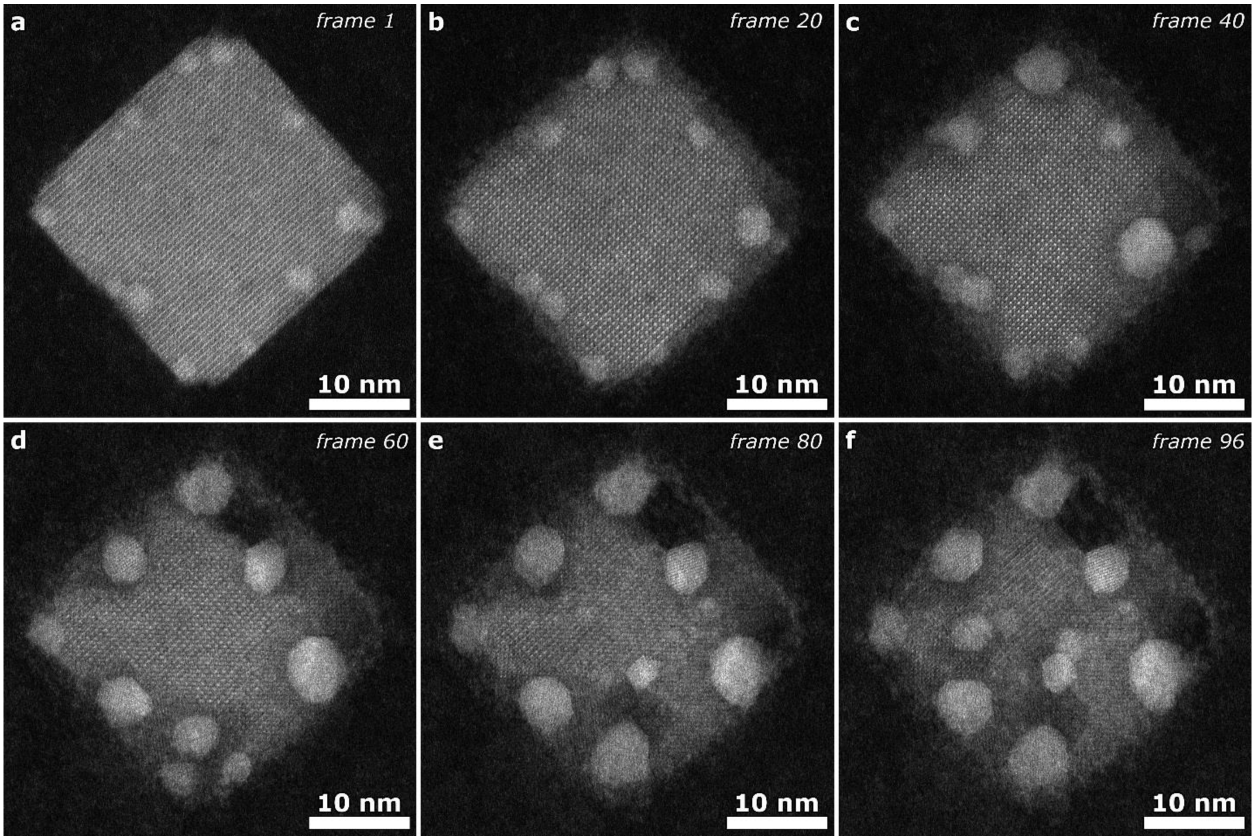

Figure S1. A few selected high resolution HAADF-STEM frames of movie S3. The dwell time used for a single frame is 2 us.

Supporting movie legends

Movie S1: Synthesis of $MAPbBr_3$ NCs by ligand-assisted reprecipitation (LARP) method

Movie S2: Degradation of a $CsPbBr_3$ nanocube upon continuous scanning of the electron beam.

Movie S3: Large-scale synthesis of $CsPbBr_3$ nanocubes. The hot injection is realized here by creating a reduced pressure in flask, and opening the valve to the dropping funnel.
